# Changing N^C^O to C^N^O Coordination in Cyclometalated Pt(II) and Pd(II) Complexes Modulates Stability and Emission Characteristics: Toward Brighter Versatility

**DOI:** 10.1002/chem.70911

**Published:** 2026-05-28

**Authors:** Joschua Lüke, Yokari Godinez‐Loyola, Alexander Hepp, Jutta Kösters, Cristian A. Strassert

**Affiliations:** ^1^ Institut für Anorganische und Analytische Chemie Münster Germany; ^2^ CiMIC, SoN, CeNTech Universität Münster Münster Germany

**Keywords:** palladium(II), photophysics, pincer ligands, platinum(II), triplet emitters

## Abstract

In this work, we present a new class of cyclometalated Pt(II) complexes bearing tridentate luminophoric chelators. Inspired by picolinato units usually acting as ancillary ligands for phosphorescent Pt(II) and Ir(III) complexes with high‐lying triplet states, we herein incorporate this coordination pattern into chelating luminophores toward tridentate pincer ligands (C^N^O) and compared them with a benzoate‐derived motif (N^C^O). Both frameworks exhibit excellent photoluminescence efficiencies with quantum yields (*Φ*_L_) reaching up to 76% (N^C^O) and 75% (C^N^O). The versatile variation of the substitution patterns at the C^N^O motif was found to significantly affect the photophysical properties, yielding tunable emission maxima ranging from 475 to 693 nm. With the introduction of electron‐donating substituents, it was demonstrated that an electron‐rich cyclometalating ring at the luminophoric backbone enables the realization of Pd(II) complexes that are emissive in solution at room temperature, if combined with strong *σ*‐donors (like *N*‐heterocyclic carbenes) as co‐ligands. This comparative study demonstrates that both coordination patterns yield highly efficient emitters with excellent solubilities in common organic solvents. Notably, the C^N^O‐based motif offers distinct advantages for future applications, owing to its greater synthetic versatility and enhanced chemical accessibility.

## Graphical abstract

A new class of cyclometalated metal complexes with pincer luminophores (C^N^O and N^C^O) is reported. They unite high photoluminescence efficiencies in liquid solutions at r.t. with tunable colors across the visible spectrum and convenient synthetic accessibility.

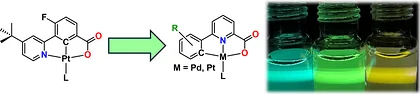

## 1 Introduction

**FIGURE 1:**
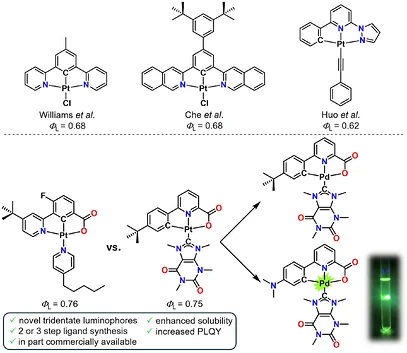
Top: Overview of Pt(II) complexes developed by Williams et al. [23], Che et al. [27], and Huo et al. [29]. Bottom: Comparison of N^C^O‐ and C^N^O‐coordinated Pt(II) complexes (left) and distinction of the substitution pattern leading to Pd(II)‐based coordination compounds that are emissive in liquid solution at room temperature (right).

Coordination compounds with late transition metal ions featuring *d* ^6^ [1, 2]_,_ *d* ^8^ [3, 4], and *d* ^10^ [5, 6, 7] electronic configurations are of great importance in photochemistry and photophysics. In addition to their applications in photocatalysis [8, 9] and bioimaging [10, 11], such complexes have also been used for the production of organic light‐emitting diodes (OLEDs) [12, 13, 14]. The advantages of these compounds over pure organic backbones rely on their partially improved photostability, extended excited state lifetimes (*τ*), and high photoluminescence quantum yields (PLQY, *Φ*_L_), both in solution and in solid state, even at room temperature. While there are several examples of (pseudo‐)octahedral Ir(III) and Re(I) complexes with *d* ^6^‐configured metal centers, Au(III)‐, Pt(II)‐, and Pd(II)‐based species are renowned for their *d* ^8^ electronic configuration with planar geometries. In this context, Pt(II) complexes have gained a lot of attraction for optoelectronic applications [15, 16]. As a heavy metal ion, it has not only an intrinsically high ligand field splitting (LFS), but also an efficient spin‐orbit coupling (SOC) which facilitates rapid intersystem crossing (ISC) after photoexcitation while enabling high phosphorescence rates. Hence, the right set of ligand configuration and donor atoms is extremely important for the design of efficient emitters. In addition to bidentate [17, 18] and tetradentate [19, 20] chelators, tridentate [21] ligands also play a crucial role in the synthesis of functional transition metal complexes. Herein, we designed two hitherto unexplored coordination patterns optimized for planar metal complexes with high rigidity and good solubility (Figure 1). A significant advantage of these pincer ligands is the fourth coordination site that remains readily available for the choice of monodentate co‐ligands, which enables precise modulation of relevant characteristics such as solubility, photophysical properties, and functionality through ligand exchange toward variable degrees of LFS. As previously mentioned, a high LFS is crucial as it raises the energy of dissociative metal‐centered (MC) excited states. Thus, it prevents thermally activated deactivation processes that involve fast radiationless pathways and otherwise lead to shortened excited state lifetimes and decreased efficiency of radiative mechanisms. In many cases, luminophoric chelators that carry at least one cyclometalating aryl unit acting as strong *σ*‐donors lead to a high LFS [22]. Various groundbreaking luminophores have already been developed for these types of compounds, whereby the pioneering work on this field was done by Williams et al. [23, 24] (N^C^N), Che et al. [25, 26, 27], and Hou et al. [28, 29] (C^N^N). Their notorious achievements constitute the benchmarks for the present study, while introducing a particularly straightforward yet versatile coordination‐chemical strategy.

While nitrogen and carbon atoms are the most common donors binding to metal cations, other elements, such as sulfur [30, 31], phosphorus [32, 33, 34], and oxygen [35, 36], can be implemented in the main chelator at the coordination site. For the latter, alcohols and carboxylic acids [35, 36] are the predominant structural features. Examples of picolinato units acting as ancillary ligands have been widely explored for the synthesis of Pt(II) and Ir(III) complexes with energetically high‐lying triplet states. Although some complexes with this structural motif have been developed, only emitters with bi‐ or tetradentate luminophores have been reported so far for complexes with a square‐planar coordination geometry [37]. Thus, we decided to compare triplet emitters bearing tridentate pincer luminophores providing N^C^O and C^N^O chelation patterns. It had been indicated previously that X^C^X motifs, with the cyclometalating position *trans* to the ancillary ligand, showed particularly promising features [23, 24].

## 2 Results and Discussion

### 2.1 Synthesis

**SCHEME 1:**
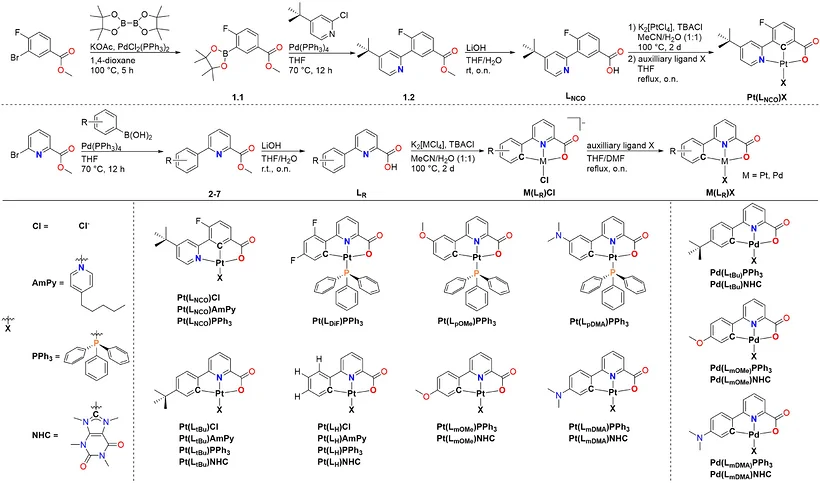
Top: Synthetic route toward N^C^O‐coordinated Pt(II) complexes as well as C^N^O‐chelated Pt(II)‐ and Pd(II)‐based species. Bottom: Set of Pt(II) and Pd(II) complexes presented in this study.

To achieve an N^C^O‐binding ligand precursor, we started by subjecting methyl‐3‐bromo‐4‐fluorobenzoate to a Miyaura‐type borylation to yield the corresponding boronic ester. The reaction with 4‐*tert*‐butylpyridine in a Suzuki cross‐coupling reaction and subsequent saponification with LiOH leads to the desired precursor **L_NCO_** (Scheme 1). The fluorine atom was inserted as a blocking position to avoid the formation of a bidentate‐coordinated Pt(II) complex. Notably, the corresponding Pd(II) complexes were not accessible under comparable reaction conditions, and no clean products were successfully obtained. A complete description of the synthetic pathways and the characterization methods is shown in the Supporting Information.

The co‐ligand exchange was surprisingly difficult, due to the fact that coordination compounds bearing strong *σ*‐donors as co‐ligands (*e.g*., isonitriles, *N*‐heterocyclic carbenes) tend to be unstable; hence, the strong *trans* influence of the cyclometalated position stemming from the N^C^O pattern results in comparatively long Pt‐ancillary ligand bond distances (see Table S3). However, this challenge was avoided when the pyridine and phenyl units were permuted toward a C^N^O pattern. Due to the versatile synthetic accessibility of C^N^O‐chelating ligands, a number of substituents showcasing electron‐donating (−OMe, −NMe_2_) and electron‐withdrawing (−F) effects were strategically introduced to assess their influence on the excited state properties of the complexes. For this purpose, the C^N^O‐chelating luminophore was accessed via a two‐step synthesis, starting with a Suzuki cross‐coupling reaction while combining methyl‐6‐bromopicolinate and substituted phenylboronic esters, followed by saponification with LiOH and yielding **L_n_** (Scheme 1), whereas the lead ligand precursor **L_H_** is commercially available. The corresponding Pt(II) and Pd(II) complexes were then prepared by reaction with the protonated ligands and K_2_[MCl_4_] in a mixture of acetonitrile and water. The synthesis and characterization details of all complexes are described in detail in the Supporting Information.

### 2.2 Photophysical Characterization

The UV–visible absorption spectra of all the metal complexes share intense bands (*ε* ∼ 1.5 10^4^ M^−1^ cm^−1^) around 250 and 300 nm. This band can be attributed to transitions into ligand‐centered (LC) singlet states with a π−π^∗^ character that stems from the aromatic moieties in the tridentate chelators. Furthermore, a band of lower intensity (*ε* ∼ 0.5 10^4^ M^−1^ cm^−1^) between 300 and 360 nm was also observed, which can be attributed to excitation into metal‐to‐ligand charge‐transfer (MLCT) singlet states, as it involves the occupied *d*‐orbitals from the metal (with admixture of π‐character from the ligand) as well as empty π^∗^ orbitals from the tridentate unit [38]. Among the series of compounds, a significant bathochromic shift can be observed while increasing the electron‐donating character of the substituents, due to a progressive HOMO destabilization.

As previously discussed, it has been demonstrated that highly efficient triplet emitters based on Pt(II) complexes can be accessed when the cyclometalation site is positioned *trans* to the ancillary ligand, even for weakly splitting ligands species such as chlorido units [23, 39]. Interestingly, **Pt(L_NCO_)Cl** showed a moderately good *Φ*_L _reaching 44%, with a comparably short *τ* of 1.7 µs. Both properties were improved by exchanging the ancillary co‐ligand, as demonstrated with **Pt(L_NCO_)AmPy** while achieving an enhanced *Φ*_L_ and longer *τ* (76% and 9.4 µs, respectively). This can be explained by the higher LFS of the pyridine co‐ligand as compared to the chlorido species, thus raising the nonradiative MC states to higher energies [40]. Although the phosphane‐decorated complex **Pt(L_NCO_)PPh_3_** was isolated, a proper photophysical characterization was not feasible due to fast photodegradation even upon brief exposure to light. This correlates with the extended Pt−P distance of 2.36 Å while demonstrating the weak Pt‐ancillary ligand bond (Figure S275, CCDC‐No. 2422728). Compared with the relatively short Pt─C bond of 1.94 Å, it shows the strong *trans*‐influence of the cyclometalated position. Thus, permutation of the phenyl and the pyridine moieties from the N^C^O ligand toward the C^N^O framework results in a relative advantage. To facilitate a direct comparison, the commercially available ligand precursor **L_H,_** as well as the synthetically derived **L_tBu_** species, were selected. Due to the change of the cyclometalating site, the complexes with chlorido ancillary ligands were expected to have inferior photophysical properties [27, 39]. However, an intense photoluminescence was observed with *Φ*_L_ values reaching 56% for **Pt(L_H_)Cl** and 60% for **Pt(L_tBu_)Cl**. The ligand exchange to insert 4‐amylpyridine or triphenylphosphane led to comparable performances with respect to their chlorido precursors. Moreover, the robust C^N^O motif tolerates stronger ancillary ligands, such as the caffeine‐based NHC derivative. Notably, improved *Φ*_L_ values reaching up to 70% for **Pt(L_H_)NHC** and 75% **Pt(L_tBu_)NHC** were achieved. These can be mainly attributed to the substantially reduced nonradiative decay rates observed by these complexes.

**FIGURE 2:**
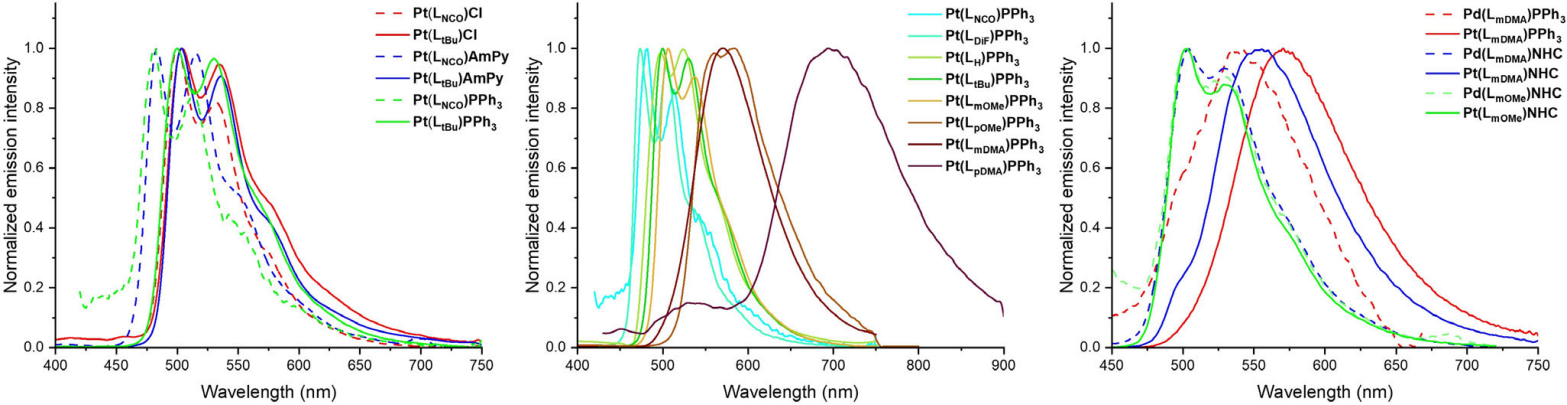
Left: Room‐temperature (RT) photoluminescence emission spectra of selected N^C^O‐ and C^N^O‐coordinated Pt(II) complexes with various ancillary ligands measured in liquid Ar‐purged DCM at RT. Center: RT emission spectra of the triphenylphosphane‐decorated Pt(II) complexes measured in liquid Ar‐purged DCM. Right: RT emission spectra of selected Pt(II) and Pd(II) complexes measured in liquid Ar‐purged DCM. All compounds were measured with *λ*_exc_ = 350 nm.

Even though the exchange of the ancillary ligand does not significantly affect the emission maximum (± 6 nm) relative to **Pt(L_tBu_)Cl** (Figure 2), the substitution pattern on the phenyl ring does have a far greater influence on the color. To eliminate potential aggregation effects and accurately investigate the impact of these substitution patterns on the emission properties, triphenylphosphane was selected as the model co‐ligand to compare the different substitution patterns that are versatilely accessible with C^N^O luminophores. Notably, a hypsochromic shift occurs for electron‐withdrawing substituents, as shown by **Pt(L_DiF_)PPh_3_** emitting at 475 nm [41]. Additionally, a bathochromic shift is observed for electron‐donating groups in *meta*‐position to the Pt−C bond (*i.e*., **Pt(L_mOMe_)PPh_3_** with *λ*_max_  =  506 nm and **Pt(L_mDMA_)PPh_3_** with *λ*_max_  =  550 nm, Figure 2). Interestingly, *para‐*substituents drastically red‐shifted the emission maxima with respect to their *meta*‐substituted analogs: switching the methoxy substitution leads to a 280 meV drop when going from **Pt(L_mOMe_)PPh_3_** to **Pt(L_pOMe_)PPh_3_** (*λ*_max_  =  572 nm). Applying this change to the dimethylamino substituent leads to a significant red shift of 480 meV when going from **Pt(L_mDMA_)PPh_3_** to **Pt(L_pDMA_)PPh_3_** (*λ*_max_  =  693 nm), which agrees with reports on comparable compounds [24, 42, 43, 44]. The change in substitution pattern also leads to a reduction in both *Φ*_L_ and *τ* (Table 1), mainly due to a faster nonradiative relaxation.

**TABLE 1:** Photophysical properties of the triphenylphosphane‐substituted Pt(II) complexes at room temperature in Ar‐purged solutions of DCM.

	*λ*_max_ (nm)	*τ* (µs)	*Φ*_L_ (%)	*k*_r_ (10^4 ^s^−^^1^)^c^	*k*_nr_ (10^4 ^s^−^^1^)^c^
**Pt(L_NCO_)PPh_3_**	481	n.d.	n.d.	n.d.	n.d.
**Pt(L_H_)PPh** ^a^	497	6.961 ± 0.005	42 ± 4	6.0 ± 0.3	8.3 ± 0.3
**Pt(L_tBu_)PPh_3_** ^a^	500	12.785 ± 0.005	60 ± 3	15.2 ± 0.6	10.1 ± 0.1
**Pt(L_DiF_)PPh_3_** ^a^	475	0.551 ± 0.001	5 ± 2	9 ± 4	172 ± 4
**Pt(L_mOMe_)PPh_3_** ^a^	506	18.49 ± 0.03	54 ± 3	2.9 ± 0.1	2.5 ± 0.1
**Pt(L_pOMe_)PPh_3_** ^a^	572	16.69 ± 0.01	46 ± 5	2.8 ± 0.1	3.2 ± 0.1
**Pt(L_mDMA_)PPh_3_** ^b^	550	4.58 ± 0.03	4 ± 2	0.7 ± 0.3	16.7 ± 0.4
**Pt(L_pDMA_)PPh_3_** ^a^	693	0.542 ± 0.002	< 2	< 3.7	180.8 − 184.5

^a^ Lifetimes correspond to monoexponential decays.

^b^ Amplitude‐weighted average ifetimes are given for biexponential decays.

^c^ The average rate constants were estimated according to *k*_r_ = *Φ*_L_/*τ* and *k*_nr_ = (1‐*Φ*_L_)/*τ* employing amplitude‐weighted average lifetimes [45].

One of the major challenges in enhancing the emissive properties of mononuclear Pd(II) complexes in solution at room temperature lies in the intrinsically lower ligand‐field splitting of the Pd(II) center. This leads to a faster population of thermally accessible metal‐centered states, wherefrom radiationless decay occurs while rendering the emissive processes less competitive. While room temperature emission from mononuclear Pd(II) complexes bearing tetradentate luminophores has already been documented in the literature by our group [20, 46], the tridentate chelators discussed by other researchers typically exhibit excimeric luminescence [47, 48]. It has been shown that the influence of electron‐shifting groups can also improve the emission efficiency of Pd(II) complexes [35]. A comparison of the herein reported Pd(II) complexes showed that room‐temperature phosphorescence in solution can be achieved in the absence of triplet dioxygen (^3^O_2_). Hence, the highest efficiency is attained by the combination of a strong electron donor (−NMe_2_) in *meta*‐position and a strong *σ*‐donor as the ancillary ligand (NHC) in the case of **Pd(L_mDMA_)NHC**. In contrast, the corresponding complexes with a *tert*‐butyl substituent at the phenyl ring did not show any phosphorescence in liquid solutions at room temperature, while methoxy‐substituted species only showed luminescence when the cyclometalation occurs in *meta*‐position with respect to the substituent, while bearing a strong NHC co‐ligand (**Pd(L_mOMe_)NHC**). However, compared to their Pt(II) analogues, none of the Pd(II) complexes could reach *Φ*_L_ values above the experimental uncertainty (*i.e*., lying below 2%). If compared with their Pt(II)‐based homologues, the spectra show the characteristic hypsochromic shift for Pd(II) complexes, which indicates an emission from the monomeric species (Figure 2, right). This comparison also shows that the luminescent Pd(II) complexes bearing NHC co‐ligands possess slightly shorter excited state lifetimes for **Pd(L_mOMe_)NHC** (*τ*  =  10.9 µs) and **Pd(L_mDMA_)NHC** (*τ*  =  11.3 µs), whereas an even shorter value is observed for **Pd(L_mDMA_)PPh_3_** (*τ*  =  3.16 µs).

## 3 Conclusions

In conclusion, two unprecedented luminophore motifs with N^C^O and C^N^O coordination frameworks were implemented in combination with diverse ancillary co‐ligands. It was shown that the use of a carboxylato unit as a coordinating donor in the resulting metal complexes can serve as a good alternative to cyclometalation or azolato moieties. The record quantum yields reported for N^C^N‐chlorido‐coordinated Pt(II) complexes (Williams et al., *Φ*_L_  =  68%) [23] were nearly matched by **Pt(L_NCO_)Cl** and also overachieved employing a pyridine derivative as the ancillary ligand (**Pt(L_NCO_)AmPy**, *Φ*_L_ = 76%). Notably, X‐ray diffractometry on single crystals showed an extended Pt−P bond length for **Pt(L_NCO_)PPh_3_**, posing limitations regarding the co‐ligand stability for the N^C^O‐motif, which relates to the strong *trans*‐influence of the cyclometalated ring. Importantly, permutation of the phenyl and pyridyl units toward the C^N^O‐pattern expanded the range of compatible co‐ligands, and **Pt(L_tBu_)NHC** achieved comparable quantum yields of *Φ*_L_  =  75%, if contrasted with the most efficient N^C^O exemplar. The highly versatile variation of substitution patterns led to a hypsochromic shift for electron‐withdrawing groups and a bathochromic shift for donors, respectively. With reference to the cyclometalation site on the phenyl moieties, a drastic bathochromic shift is exhibited when going from the *meta*‐decorated compounds to the *para*‐substituted complexes **Pt(L_pOMe_)PPh_3_** (+ 66 nm) and **Pt(L_pDMA_)PPh_3_** (+ 143 nm). Comparable bathochromic shifts have already been demonstrated by our group for electron‐poor Pt(II) centers when going from *meta*‐ to *para*‐substitution (as evidenced by the chemical shifts in ^195^Pt‐NMR spectroscopy) [49]. For the Pd(II) complexes, a higher brightness is achieved with electron donors at the cyclometalating ring, highlighting **Pd(L_mDMA_)NHC** as the most emissive exemplar in liquid solution at room temperature. Based on this comparative study, the C^N^O coordination mode provided excellent performances, particularly if compared to the N^C^O pattern, but with a substantially simplified synthetic route toward higher chemical stability regarding the choice of co‐ligands, while leading to a broad scope of accessible decoration motifs for the C^N^O luminophores. For optoelectronics, higher radiative rate constants and a slower deactivation must be achieved, thus constituting particular challenges for Pd(II) complexes. Hence, for future work, *meta*‐substituted C^N^O‐chelated complexes with Ni(II), Pd(II), Pt(II), and Au(III) centers could be combined with even stronger ancillary co‐ligands toward efficient phosphorescence at room temperature. In this sense, electrochemical, theoretical, and solid‐state photophysical studies will help elucidate the fundamental aspects to be addressed. Applications in multiscale‐multimodal bioimaging can be envisioned by the insertion of proper targeting moieties in the periphery of the luminophoric chelator while achieving solubility in water with the choice of ancillary units that act as monodentate co‐ligands to provide polar groups or ionic charges.

## Conflicts of Interest

The authors have no conflict of interest to declare.

## Supporting information




**Supporting File**: The authors have cited additional references within the Supporting Information [50–53].
