# Supplementary material for: Changing N^C^O to C^N^O Coordination in Cyclometalated Pt(II) and Pd(II) Complexes Modulates Stability and Emission Characteristics: Toward Brighter Versatility
Source: Chemistry. 2026 May 28;32(34):e70911. doi: 10.1002/chem.70911 (PMC13569946; doi:10.1002/chem.70911)
Supplement: Supplementary file 1 [file CHEM-32-e70911-s001.pdf]

Supporting Information

Changing N^C^O to C^N^O Coordination in Cyclometalated Pt(II) and Pd(II) Complexes Modulates Stability and Emission Characteristics: Towards Brighter Versatility

Joschua Lüke,[a,b] Yokari Godinez-Loyola,[a,b] Jutta Kösters,[a] Alexander Hepp,[a] and Cristian A. Strassert[a,b]*

[a] Institut für Anorganische und Analytische Chemie, Universität Münster, Corrensstraße 28/30, 48149 Münster, Germany

[b] CiMIC, SoN, CeNTech, Universität Münster, Heisenbergstraße 11, 48149 Münster, Germany

Table of Contents

Section S1: Methods and materials S2
Section S2: Synthesis and characterization S4
Section S3: NMR and mass spectra S40
Section S4: X-ray diffractometry data S177
Section S5: Photophysical characterization S198
Section S6: References S240

Section S1: Methods and materials

For all reactions, chemicals were used as purchased without further purification. 9-Methylcaffeine-ium Iodide was synthesized in a slightly modified procedure.[1] For column chromatography silica, gel 60 (0.063 – 0.200 mm) was used, purchased from Merck.

S1.1 NMR spectroscopy and mass spectrometry

NMR spectra were recorded at the Institut für Anorganische und Analytische Chemie (Univ. Münster) using a Bruker Avance III 400 or a Bruker Avance Neo 500. All NMR measurements were performed at 300 K. The chemical shifts (δ) of the spectra are given in parts per million (ppm) and the signals are referenced to the residual proton signal in deuterated methylene chloride DCM-d_2 (^{1}H = 5.32 ppm / ^{13}C = 54 ppm). The signal multiplicities are given as s (singlet), d (doublet), t (triplet), q (quartet) and m (multiplet). The mass spectrometry was carried out at the Organisch-Chemisches Institut (Univ. Münster) using a LTQ Orbitrap LTQXL (Thermo Fisher Scientific, Bremen, Germany) with electron spray ionization (ESI) to obtain exact mass (EM) spectra.

S1.2 Single crystal X-ray diffraction measurements

The single-crystal X-ray diffraction measurements for crystalline samples of **Pt(L_{NCO})PPh_3**, **Pt(L_{Ph})PPh_3**, **Pt(L_{Ph})AmPy**, **Pt(L_{tBu})AmPy**, **Pt(L_{DiFl})PPh_3**, **Pt(L_{pOMe})PPh_3**, **Pd(L_{pOMe})PPh_3**, **Pt(L_{pDMA})PPh_3** and **Pt(L_{pDMA})PPh_3** were carried out at the Institut für Anorganische und Analytische Chemie (Univ. Münster) using a Venture single crystal X-ray diffractometer (Bruker AXS, Karlsruhe, Germany). The X-ray diffractometric data reduction was carried out using SAINT+.[2] The crystal structures were dissolved by using SHELXTL.[3] The molecular structures in the crystal and the unit cells were created with Mercury.[4] All details of the crystallographic data for the above-mentioned compounds are available from the Cambridge Crystallographic Data Center (CCDC) using the corresponding depository number.

S1.3 UV-vis absorption and time-resolved photoluminescence (micro)spectroscopies

UV-visible absorption spectra were measured with a Shimadzu UV-Vis spectrophotometer UV-1900i equipped with a 20 W halogen lamp and a deuterium lamp, a silicon photodiode detector, a monochromator with a spectral bandwidth of 1 nm in the range of 190 nm to 1100 nm (a LO-RAY-LIGHT-grade blazed holographic grating in Czerny-Turner mounting), and employing LabSolutions UV-Vis software.

Photoluminescence quantum yields were measured with a Hamamatsu Photonics absolute PL quantum yield measurement system (C9920-02) equipped with a L9799-01 CW Xe light source

(150 W), a monochromator, a C7473 photonic multi-channel analyser, an integrating sphere and employing U6039-05 software (Hamamatsu Photonics, Ltd., Shizuoka, Japan).

Steady-state excitation and emission spectra were recorded on a FluoTime 300 spectrometer from PicoQuant equipped with a 300 W ozone-free Xe lamp (200-1100 nm), a 10 W Xe flash-lamp (200-1100 nm, pulse width ca. 1 µs) with repetition rates of 1 – 300 Hz, a single-grating excitation monochromator (Czerny-Turner type, grating with 1200 lines/mm, blaze wavelength: 300 nm), diode lasers (pulse width < 20 ps) operated by a computer-controlled laser driver PDL-820 “Sepia II” (repetition rate up to 80 MHz, burst mode for slow and weak decays), two emission monochromators (Czerny-Turner, selectable between single-grating blazed at 500 nm with 2.7 nm/mm dispersion and 1200 lines/mm, or single-grating blazed at 1250 nm with 5.4 nm/mm dispersion and 600 lines/mm) with adjustable slit width between 20 µm and 4 mm, Glan-Thompson polarizers for excitation (after the Xe-lamps) and emission (after the sample). A Peltier-thermostatized sample holder from Quantum Northwest (-40 °C – 105 °C), along two detectors (namely a PMA Hybrid-40 from PicoQuant with transit time spread FWHM < 120 ps, 300 – 720 nm, or a R5509-43 NIR detector with transit time spread FWHM 1.5 ns, 300-1400 nm from Hamamatsu) were used. Steady-state spectra and photoluminescence lifetimes were recorded in TCSPC mode by a PicoHarp 300 (minimum base resolution 4 ps) or in MCS mode by a TimeHarp 260 (where up to several ms can be traced). Emission and excitation spectra were corrected for source intensity (lamp and grating) by standard correction curves. For samples with lifetimes in the ns order, an instrument response function calibration (IRF) was performed using a diluted Ludox® dispersion. Lifetime analysis was performed using the commercial EasyTau 2 software (PicoQuant). The quality of the fit was assessed by minimizing the reduced chi squared function (χ^2) and visual inspection of the weighted residuals and their autocorrelation. All solvents used were of spectrometric grade (Uvasol®, Merck).

Section S2: Synthesis and characterization

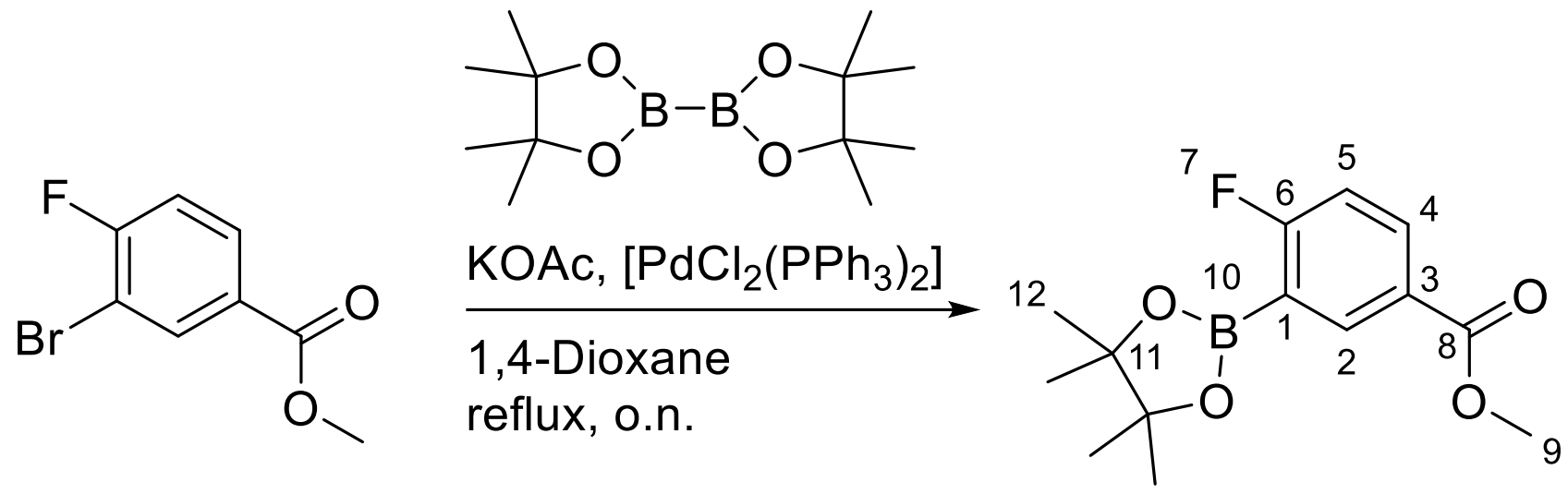

1.1

Intermediate compound 1.1. Methyl-3-bromo-4-fluorobenzoate (3 g, 12.9 mmol, 1 eq.), Bis(pinacolato)diboron (4.90 g, 19.3 mmol, 1.5 eq.), potassium acetate (2.53 g, 25.8 mmol, 2 eq.), and $[PdCl_2(PPh_3)_2]$ (452 mg, 0.65 mmol, 0.05 eq.) were dissolved in 1,4-dioxane (40 mL), the mixture was purged with argon for 5 minutes and then refluxed overnight. The reaction mixture was allowed to cool down to room temperature, then diluted with ethyl acetate and filtrated. The solvent was removed under reduced pressure, and the crude product was purified by flash column chromatography (SiO_2, cyclohexane/ethyl acetate = 95:5) yielding **1.1** (2.61 g, 9.32 mmol, 72 %) as a white solid.

EM-MS-ESI (MeOH, $C_{14}H_{18}O_4BFNa$): calcd. [**1.1**+Na]$^+$ = 303.11720, found [**1.1**+Na]$^+$ = 303.11770.

^{1}H-NMR (400 MHz, $CDCl_3$): δ (ppm) = 8.43 (dd, $^3J_{HF}$ = 5.9 Hz, $^4J_{HH}$ = 2.3 Hz, 1H, H_2), 8.12 (ddd, $^3J_{HH}$ = 8.7 Hz, $^3J_{HF}$ = 5.3 Hz, $^4J_{HF}$ = 2.2 Hz, 1H, H_4), 7.07 (t, $^3J_{HH}$ = 8.7 Hz, $^3J_{HF}$ = 8.7 Hz, 1H, H_5), 3.90 (s, 3H, H_9), 1.36 (s, 12H, H_{12}).

^{13}C{^{1}H}-NMR (101 MHz, $CDCl_3$): δ (ppm) = 170.0 (d, $^1J_{CF}$ = 251.3 Hz, C_6), 166.3 (C_8), 139.0 (d, $^3J_{CF}$ = 9.8 Hz, C_2), 135.0 (d, $^3J_{CF}$ = 10.4 Hz, C_4), 126.0 (d, $^4J_{CF}$ = 3.0 Hz, C_3), 115.6 (d, $^2J_{CF}$ = 24.9 Hz, C_1), 84.2 (C_{11}), 52.1 (C_9), 24.8 (C_{12}).

^{19}F{^{1}H}-NMR (376 MHz, $CDCl_3$): δ (ppm) = - 95.5 (F_7).

^{11}B{^{1}H}-NMR (128 MHz, $CDCl_3$): δ (ppm) = 30.0 (B_{10}).

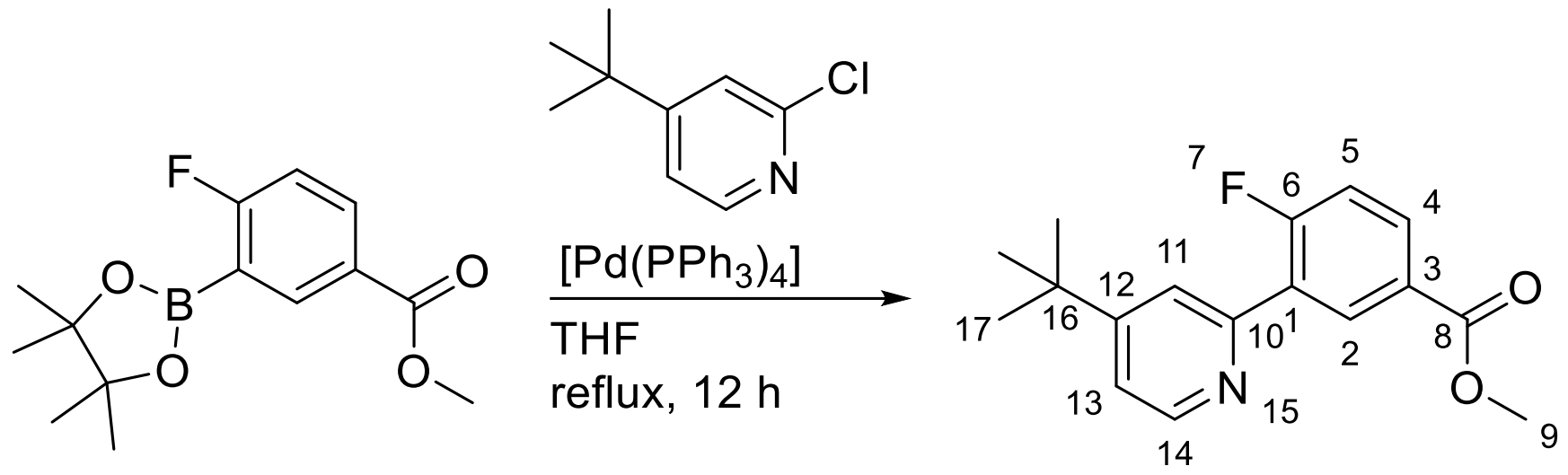

1.1 **1.2**

Intermediate compound 1.2. Compound **1.1** (600 mg, 2.14 mmol, 1.2 eq.), 4-(*tert*-butyl)-2-chloropyridine (302 mg, 288 µL, 1.78 mmol, 1.0 eq.) and $[Pd(PPh_3)_4]$ (205 mg, 0.18 mmol, 0.1 eq.) were dissolved in tetrahydrofuran (THF, 30 mL). The mixture was purged with argon at room temperature for 5 minutes before the addition of a potassium carbonate solution (2 M, 5 mL), followed by an additional 5 minutes argon purging. The solution was refluxed for 12 hours. The reaction was allowed to cool down to room temperature and was washed with distilled water (15 mL). The phases were separated, the aqueous phase was extracted with ethyl acetate (3 × 25 mL) and the combined organic phases were dried over anhydrous sodium sulfate. The solvent was removed under reduced pressure, and the crude product was purified by flash column chromatography (SiO_2, cyclohexane/ethyl acetate = 9:1) yielding **1.2** (332 mg, 1.35 mmol, 73 %) as a white solid.

EM-MS-ESI (MeOH, $C_{24}H_{27}Br_2N_3O_2$): calcd. [**1.2**+H]$^+$ = 288.13810, found [**1.2**+H]$^+$ = 288.13943.

^{1}H-NMR (500 MHz, DCM-d_2): δ (ppm) = 8.62 (m, 2H, $H_{2/14}$), 8.05 (ddd, $^3J_{HH}$ = 8.6 Hz, $^4J_{HF}$ = 4.8 Hz, $^4J_{HH}$ = 2.3 Hz, 1H, H_4), 7.79 (td, $^4J_{HH}$ = 2.0 Hz, $^5J_{HH}$ = 0.8 Hz, 1H, H_{11}), 7.31 (dd, $^3J_{HH}$ = 5.3 Hz, $^4J_{HH}$ = 1.9 Hz, 1H, H_{13}), 7.24 (dd, $^3J_{HH}$ = 8.6 Hz, $^2J_{HF}$ = 10.8 Hz, 1H, H_5), 3.91 (s, 3H, H_9), 1.36 (s, 9H, H_{17}).

^{13}C{^{1}H}-NMR (126 MHz, DCM-d_2): δ (ppm) = 166.4 (C_8), 163.6 (C_6), 161.0 (C_{12}), 152.7 (C_{14}), 150.1 (C_{10}), 132.0 (C_4), 128.7 (C_1), 127.3 (C_5), 122.0 (C_{11}), 120.5 (C_{13}), 116.9 (C_5), 35.16 (C_9), 30.69 (C_{16}).

^{19}F{^{1}H}-NMR (471 MHz, DCM-d_2): δ (ppm) = - 111.0 (F_7).

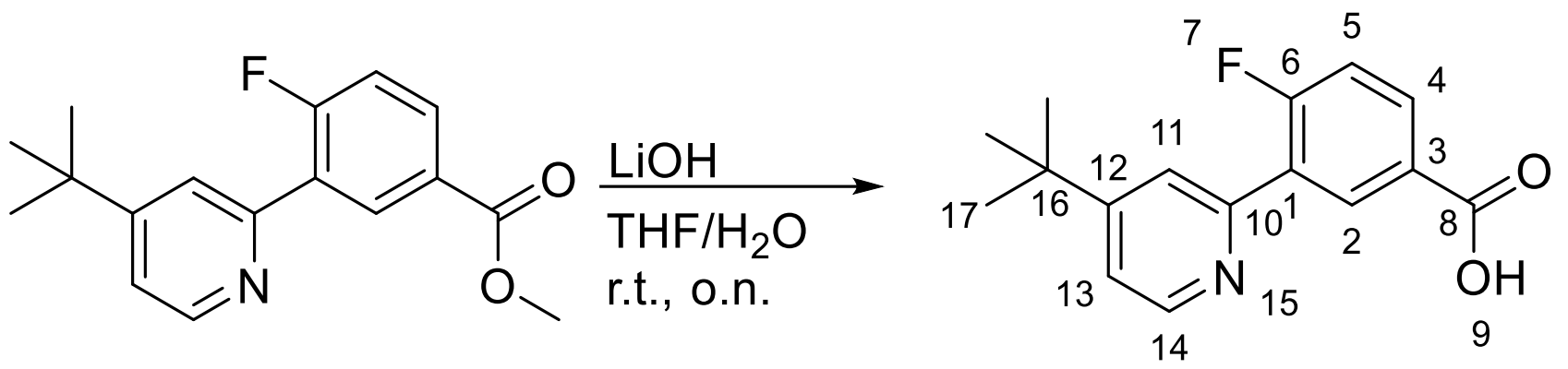

1.2 **L_{NCO}**

***Ligand precursor L_{NCO}*.** Compound **1.2** (400 mg, 1.39 mmol, 1.0 eq.) was dissolved in THF (10 mL) and lithium hydroxide (64 mg, 2.78 mmol, 2.0 eq.) was dissolved in distilled water (10 mL). The mixture of both solutions was stirred at room temperature overnight and subsequently the THF was removed under reduced pressure. The remaining solvent was neutralized with HCl (1 M) and the resulting precipitate was filtered through a glass frit and finally was washed with distilled water. The product was dried *in vacuo* to yield **L_{NCO}** (294 mg, 1.08 mmol, 77 %) as a white solid.

EM-MS-ESI (MeOH, $C_{16}H_{17}N_1O_2F$): calcd. [L_{NCO}+H]$^+$ = 274.12284, found [L_{NCO}+H]$^+$ = 274.12378; calcd. [L_{NCO}+Na]$^+$ = 296.10468, found [L_{NCO}+Na]$^+$ = 296.10573.

^{1}H-NMR (500 MHz, DMSO-d_6): δ (ppm) = 13.15 (s, 1H, H_9), 8.63 (dd, $^3J_{HH}$ = 5.2 Hz, $^4J_{HH}$ = 0.8 Hz, 1H, H_{14}), 8.52 (dd, $^4J_{HH}$ = 2.4 Hz, $^4J_{HF}$ = 7.7 Hz, 1H, H_2), 8.03 (ddd, $^3J_{HH}$ = 8.6 Hz, $^4J_{HH}$ = 2.3 Hz, $^4J_{HF}$ = 4.8 Hz, 1H, H_4), 7.79 (td, $^4J_{HH}$ = 1.9 Hz, $^5J_{HH}$ = 0.8 Hz, 1H, H_{11}), 7.43 (m, 2H, $H_{5/13}$), 1.31 (s, 9H, H_{17}).

^{13}C{^{1}H}-NMR (126 MHz, DMSO-d_6): δ (ppm) = 166.2 (C_8), 162.3 (C_6), 160.2 (C_{12}), 151.5 (C_{10}), 149.7 (C_{10}), 132.5 (C_2), 131.8 (C_4), 127.5 (C_3), 127.4 (C_1), 121.0 (C_{11}), 120.23 (C_{13}), 116.8 (C_5), 34.5 (C_{16}), 30.1 (C_{17}).

^{19}F{^{1}H}-NMR (376 MHz, DMSO-d_6): δ (ppm) = - 111.1 ppm (F_7).

^{15}N-NMR (51 MHz, DMSO-d_6): δ (ppm) = 307 (N_2).

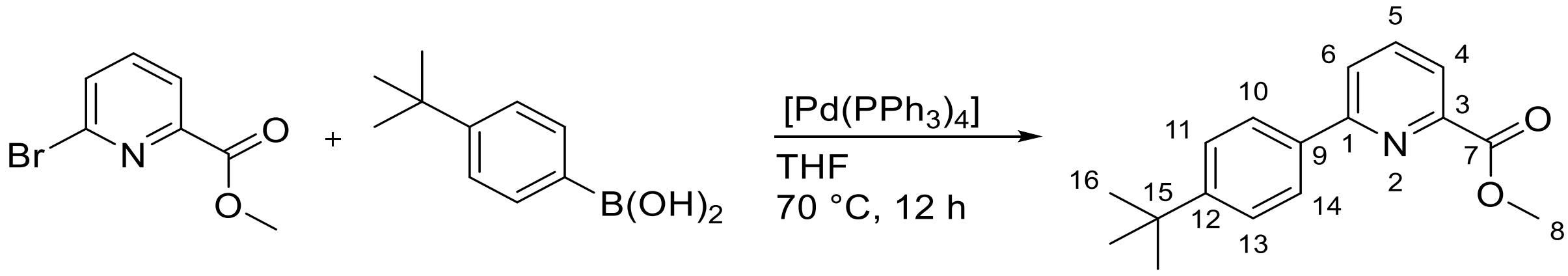

2

***Intermediate compound 2*.** Methyl-6-bromopicolinate (2.0 g, 9.30 mmol, 1 eq.), 4-(*tert*-butyl)phenylboronic acid (1.98 g, 11.10 mmol, 1.0 eq.), and [Pd(PPh_3)$_4$] (540 mg, 0.46 mmol, 0.05 eq.) were dissolved in THF (30 mL). The mixture was purged with argon at room temperature for 5 minutes before the addition of a potassium carbonate solution (2 M,

5 mL), followed by an additional 5 minutes of argon purging. The solution was refluxed for 12 hours. The reaction was allowed to cool down to room temperature and was washed with distilled water (15 mL). The phases were separated, the aqueous phase extracted with ethyl acetate (3 × 25 mL) and the combined organic phases were dried over anhydrous sodium sulfate. The solvent was removed under reduced pressure, and the crude product was purified by flash column chromatography (SiO_2, cyclohexane/ethyl acetate = 9:1) yielding **2** (1.69 g, 6.27 mmol, 67 %) as a white solid.

EM-MS-ESI (MeOH, $C_{17}H_{20}N_1O_2$): calcd. [**2**+H]$^+$ = 270.14863, found [**2**+H]$^+$ = 270.14886; calcd. [**2**+Na]$^+$ = 292.13048, found [**2**+Na]$^+$ = 292.13080.

^{1}H-NMR (400 MHz, DCM-d_2): δ (ppm) = 8.00 (m, 3H, H_{4-6}), 7.90 (m, 2H, H_{10}), 7.53 (m, 2H, H_{11}), 3.99 (s, 3H, H_8), 1.37 (s, 9H, H_{14}).

^{13}C{^{1}H}-NMR (101 MHz, DCM-d_2): δ (ppm) = 166.3 (C_7), 157.8 (C_3), 153.2 (C_{12}), 148.5 (C_1), 138.0 (C_{15}), 136.1 (C_9), 127.1 (C_{10}), 126.2 (C_{11}), 123.6 (C_4), 123.4 (C_6), 35.0 (C_{13}), 31.4 (C_{14}).

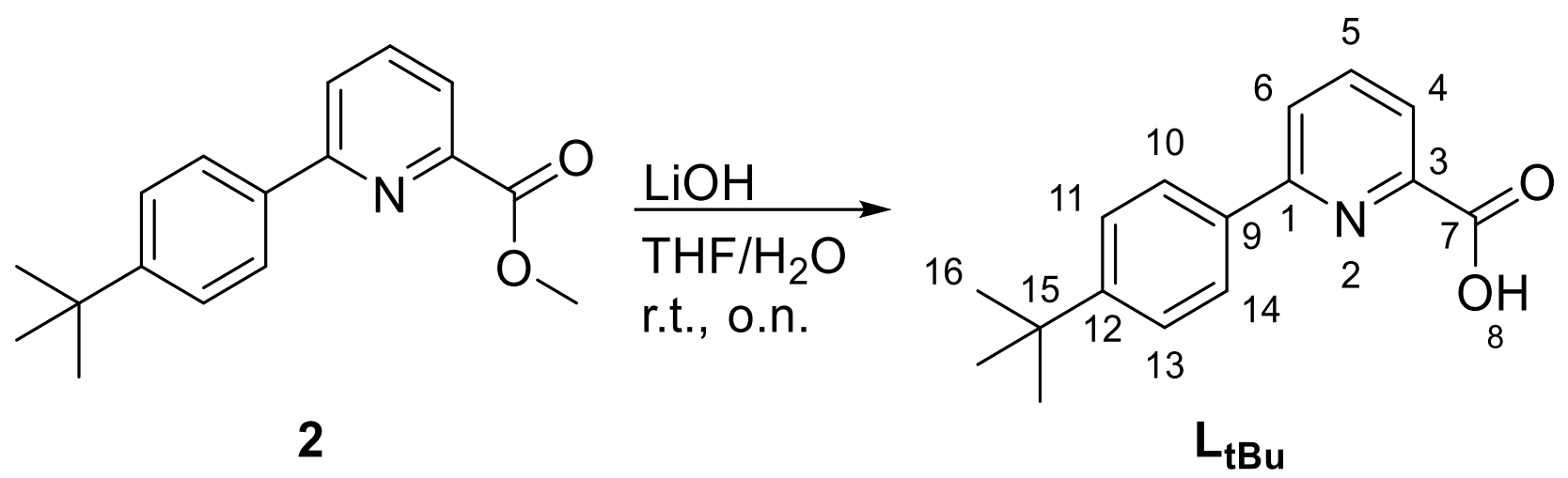

***Ligand precursor L_{tBu}*.** Compound **2** (357 mg, 1.40 mmol, 1.0 eq.) was dissolved in THF (10 mL) and lithium hydroxide (67 mg, 2.80 mmol, 2.0 eq.) was dissolved in distilled water (10 mL). The mixture was stirred at room temperature overnight and subsequently the THF was removed under reduced pressure. The remaining solvent was neutralized with HCl (1 M) and the resulting precipitate was filtered through a glass frit and finally was washed with distilled water. The product was dried *in vacuo* to yield **L_{tBu}** (294 mg, 1.08 mmol, 77 %) as a white solid.

EM-MS-ESI (MeOH, $C_{16}H_{18}N_1O_2$): calcd. [**L_{tBu}**+H]$^+$ = 256.13321, found [**L_{tBu}**+H]$^+$ = 256.1332; calcd. [**L_{tBu}**+Na]$^+$ = 278.11479, found [**L_{tBu}**+Na]$^+$ = 278.11515.

^{1}H-NMR (500 MHz, DMSO-d_6): δ (ppm) = 7.97 (dd, $^3J_{HH}$ = 7.6 Hz, $^4J_{HH}$ = 1.2 Hz, 1H, H_4), 7.90 (t, $^3J_{HH}$ = 7.7 Hz, 1H, H_5), 7.84 (m, 2H, $H_{10/14}$), 7.76 (dd, $^3J_{HH}$ = 7.8 Hz, $^4J_{HH}$ = 1.2 Hz, 1H, H_6), 7.37 (m, 2H, $H_{11/13}$), 1.27 (s, 9H, H_{14}).

^{13}C{^{1}H}-NMR (126 MHz, DMSO-d_6): δ (ppm) = 168.0 (C_7), 156.4 (C_3), 155.9 (C_1), 151.2 (C_{12}), 138.1 (C_5), 136.1 (C_9), 127.0 (C_{10}), 125.2 (C_{11}), 122.0 (C_4), 121.9 (C_6), 34.2 (C_{13}), 30.9 (C_{14}).

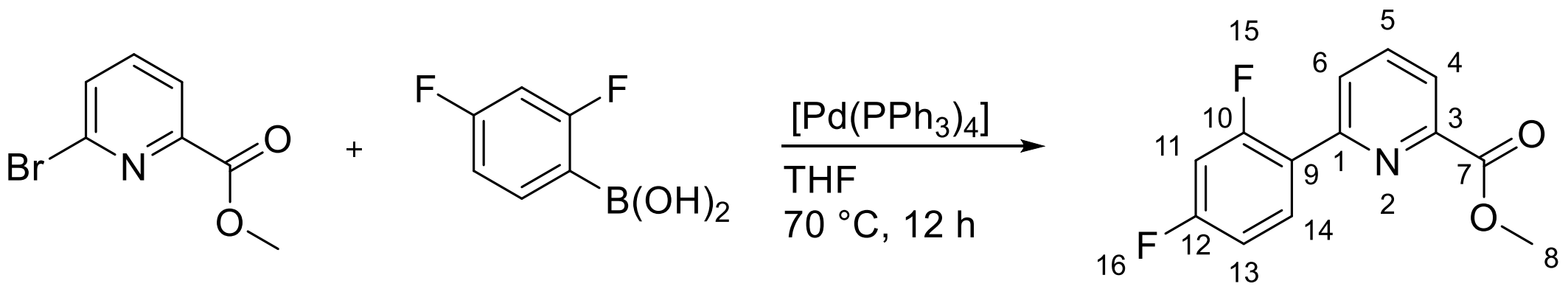

3

Compound 3. Methyl-6-bromopicolinate (300 mg, 1.39 mmol, 1.0 eq.), 2,4-difluorophenylboronic acid (263 mg, 1.67 mmol, 1.2 eq.) and $[Pd(PPh_3)_4]$ (160 mg, 0.14 mmol, 0.1 eq.) were dissolved in THF (20 mL). The mixture was purged with argon at room temperature for 5 minutes before the addition of a potassium carbonate solution (2 M, 5 mL), followed by an additional 5 minutes of argon purging. The solution was refluxed for 12 hours. The reaction was allowed to cool down to room temperature and was washed with distilled water (15 mL). The phases were separated, the aqueous phase extracted with ethyl acetate (3 × 15 mL) and the combined organic phases were dried over anhydrous sodium sulfate. The solvent was removed under reduced pressure, and the crude product was purified by flash column chromatography (SiO_2, cyclohexane/ethyl acetate = 9:1) yielding **3** (293 mg, 1.18 mmol, 85 %) as a white solid.

EM-MS-ESI (MeOH, $C_{13}H_9NO_2F_2$): calcd. [**3**+Na]$^+$ = 272.04936, found [**3**+Na]$^+$ = 272.04928.

^{1}H-NMR (400 MHz, DCM-d_2): δ (ppm) = 8.06 (m, 2H, $H_{5/14}$), 7.88 (m, 2H, $H_{4/6}$), 6.99 (dddd, $^4J_{HH}$ = 2.5 Hz, $^4J_{HH}$ = 1.0 Hz, $^3J_{HF}$ = 8.8 Hz, $^3J_{HF}$ = 7.8 Hz, 1H, H_{11}), 6.88 (ddd, $^3J_{HH}$ = 8.8 Hz, $^3J_{HF}$ = 11.3 Hz, $^5J_{HF}$ = 2.5 Hz, 1H, H_{13}), 3.98 (s, 3H, H_8).

^{13}C{^{1}H}-NMR (101 MHz, DCM-d_2): δ (ppm) = 163.4 (dd, $^1J_{CF}$ = 251.7 Hz, $^3J_{CF}$ = 12.2 Hz, C_{12}), 160.7 (dd, $^1J_{CF}$ = 252.7 Hz, $^3J_{CF}$ = 12.0 Hz, C_{10}), 152.7 (d, $^3J_{CF}$ = 2.7 Hz, C_1), 148.1 (C_3), 137.4 (C_5), 132.5 (dd, $^3J_{CF}$ = 9.8 Hz, $^3J_{CF}$ = 4.3 Hz, C_{14}), 127.2 (d, $^4J_{CF}$ = 9.8 Hz, C_6), 123.6 (C_4), 112.0 (dd, $^2J_{CF}$ = 21.0 Hz, $^4J_{CF}$ = 3.6 Hz, C_{13}), 104.2 (dd, $^2J_{CF}$ = 27.3 Hz, $^2J_{CF}$ = 26.3 Hz, C_{11}), 52.8 (C_8).

^{19}F{^{1}H}-NMR (376 MHz, DCM-d_2): δ (ppm) = - 108.3 (d, $^4J_{FF}$ = 8.9 Hz, F_{16}), - 112.9 (d, $^4J_{FF}$ = 8.8 Hz, F_{15}).

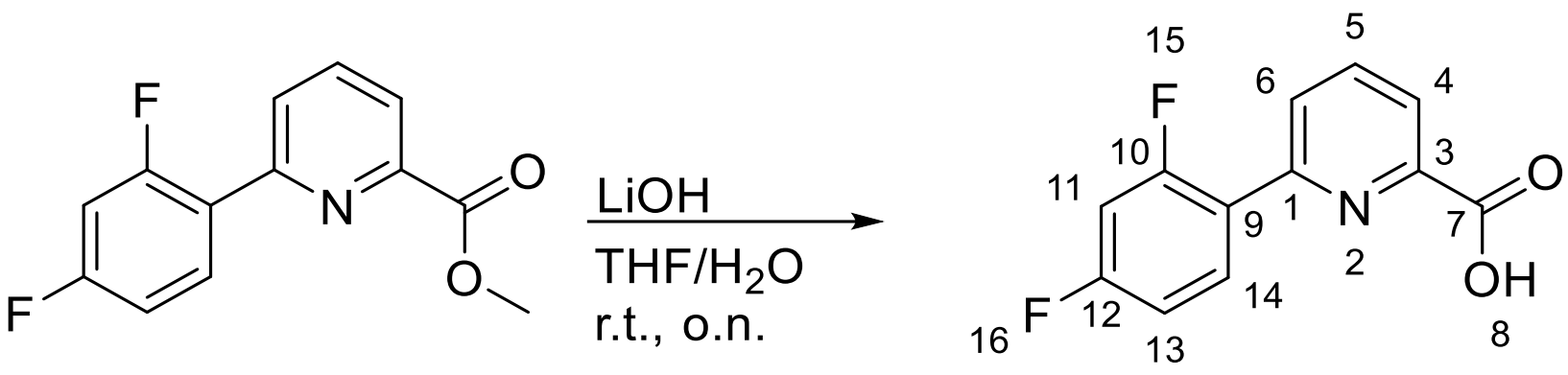

3 **L_{DiF}**

Ligand precursor L_{DiF}. Compound **3** (250 mg, 1.0 mmol, 1.0 eq.) was dissolved in THF (10 mL) and lithium hydroxide (48 mg, 2.0 mmol, 2.0 eq.) was dissolved in distilled water (10 mL). The mixture was stirred at room temperature overnight and subsequently the THF was removed under reduced pressure. The remaining solvent was neutralized with HCl (1 M) and the resulting precipitate was filtered through a glass frit and finally was washed with distilled water. The product was dried *in vacuo* to yield **L_{DiF}** (213 mg, 0.91 mmol, 91 %) as a white solid.

EM-MS-ESI (MeOH, $C_{12}H_7N_1O_2F_2$): calcd. [**L_{DiF}**-H]$^-$ = 234.03611, found [**L_{DiF}**-H]$^-$ = 234.03715.

^{1}H-NMR (500 MHz, DMSO-d_6): δ (ppm) = 13.27 (s, 1H, H_8), 8.10 (m, 2H, $H_{5/14}$), 8.04 (dd, $^3J_{HH}$ = 7.7 Hz, $^4J_{HH}$ = 1.1 Hz, 1H, H_4), 7.98 (ddd, $^3J_{HH}$ = 7.8 Hz, $^4J_{HH}$ = 1.2 Hz, $^5J_{HF}$ = 2.3 Hz, 1H, H_6), 7.42 (ddd, $^4J_{HH}$ = 2.6 Hz, $^3J_{HF}$ = 11.7, $^3J_{HF}$ = 9.3 Hz, 1H, H_{11}), 7.28 (m, 1H, H_{13}).

^{13}C{^{1}H}-NMR (126 MHz, DMSO-d_6): δ (ppm) = 165.9 (C_7), 162.7 (dd, $^1J_{CF}$ = 249.2 Hz, $^3J_{CF}$ = 12.5 Hz, C_{10}), 160.0 (dd, $^1J_{CF}$ = 251.5 Hz, $^3J_{CF}$ = 12.5 Hz, C_{12}), 151.4 (d, $^3J_{CF}$ = 2.5 Hz, C_1), 148.5 (C_3), 138.4 (C_5), 132.6 (C_{14}), 126.9 (d, $^4J_{CF}$ = 8.9 Hz, C_6), 123.5 (C_4), 122.8 (dd, $^2J_{CF}$ = 11.6 Hz, $^4J_{CF}$ = 3.7 Hz, C_9), 112.1 (C_{13}), 104.6 (C_{11}).

^{19}F{^{1}H}-NMR (471 MHz, DMSO-d_6): δ (ppm) = - 108.5 ($^4J_{FF}$ = 9.2 Hz, F_{16}), - 112.6 ($^4J_{FF}$ = 9.2 Hz, F_{15}).

^{15}N-NMR (51 MHz, DMSO-d_6): δ (ppm) = 313 (N_2).

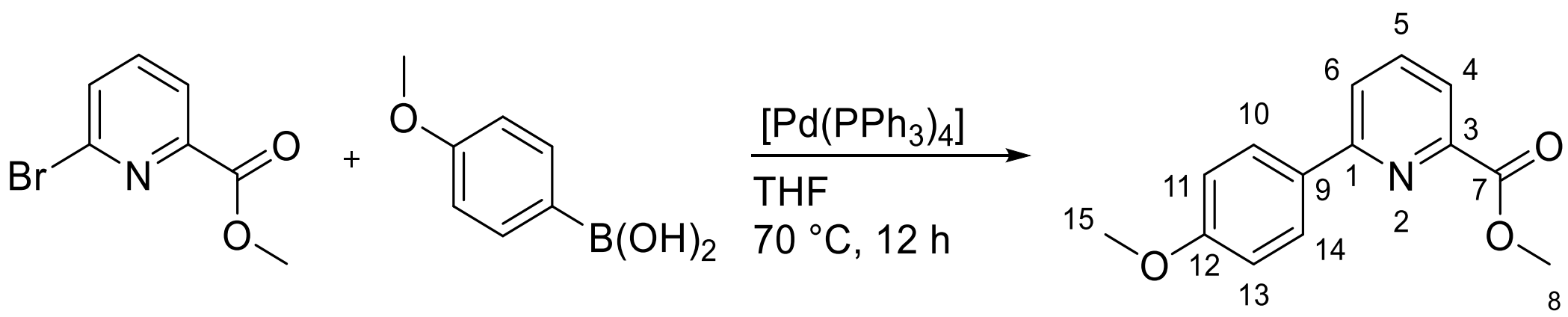

4

Intermediate compound 4. Methyl-6-bromopicolinate (300 mg, 1.39 mmol, 1.0 eq.), 4-methoxyphenylboronic acid (253 mg, 1.67 mmol, 1.2 eq.), and [Pd(PPh_3)$_4$] (80 mg, 0.07 mmol, 0.05 eq.) were dissolved in THF (30 mL). The mixture was purged with argon at

room temperature for 5 minutes before adding potassium carbonate solution (2 M, 4 mL), while purging with argon for additional 5 minutes. The solution was refluxed for 12 hours. The reaction was allowed to cool down to room temperature and was washed with distilled water (15 mL). The phases were separated, the aqueous phase extracted with ethyl acetate (3 × 20 mL) and the combined organic phases were dried over anhydrous sodium sulfate. The solvent was removed under reduced pressure, and the crude product was purified by flash column chromatography (SiO_2, cyclohexane/ethyl acetate = 9:1) yielding **4** (315 mg, 1.29 mmol, 93 %) as a white solid.

EM-MS-ESI (MeOH, $C_{14}H_{13}NO_3$): calcd. [**4**+Na]$^+$ = 266.07876, found [**4**+Na]$^+$ = 266.07876.

^{1}H-NMR (400 MHz, $CDCl_3$): δ (ppm) = 8.00 (m, 3H, $H_{6/11/13}$), 7.84 (m, 2H, $H_{4/5}$), 7.00 (m, 2H, $H_{10/14}$), 4.01 (s, 3H, H_8), 3.86 (s, 3H, H_{15}).

^{13}C{^{1}H}-NMR (101 MHz, $CDCl_3$): δ (ppm) = 166.2 (C_7), 161.0 (C_{12}), 157.5 (C_1), 148.0 (C_3), 137.7 (C_5), 131.2 (C_9), 128.7 ($C_{11/13}$), 123.0 (C_6), 122.8 (C_4), 114.3 ($C_{10/14}$), 55.5 (C_8), 52.9 (C_{15}).

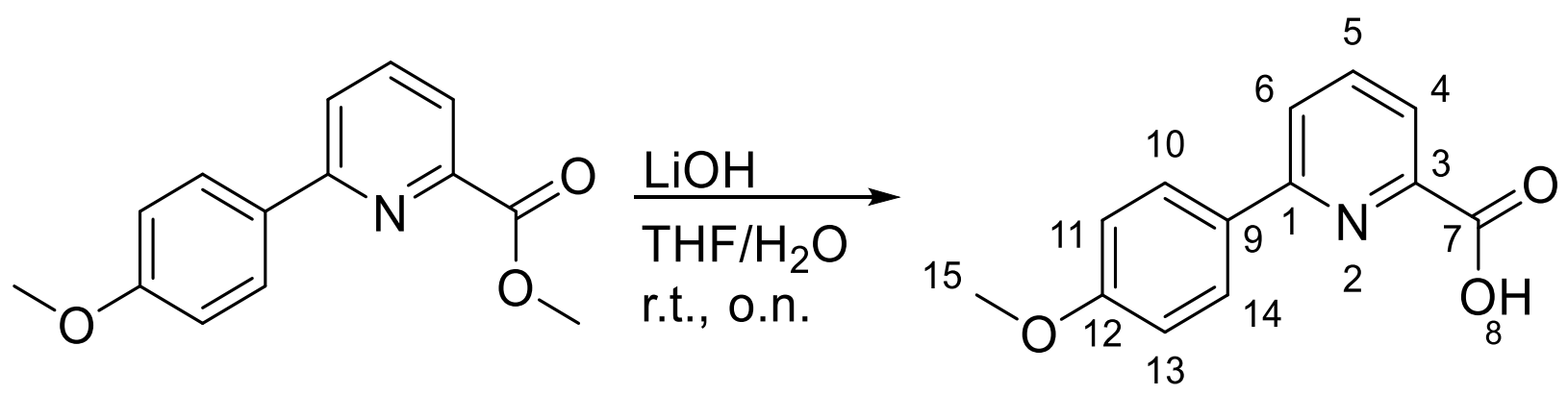

***Ligand precursor L_{mOMe}*.** Compound **4** (300 mg, 1.23 mmol, 1.0 eq.) was dissolved in THF (10 mL) and lithium hydroxide (88 mg, 3.70 mmol, 3.0 eq.) was dissolved in distilled water (10 mL). The mixture was stirred at room temperature overnight and subsequently the THF was removed under reduced pressure. The remaining solvent was neutralized with HCl (1 M) and the resulting precipitate was filtered through a glass frit and finally was washed with distilled water. The product was dried *in vacuo* to yield **L_{mOMe}** (235 mg, 0.87 mmol, 84 %) as a white solid.

EM-MS-ESI (MeOH, $C_{13}H_{11}NO_3$): calcd. [**L_{mOMe}**+H]$^+$ = 230.08117, found [**L_{mOMe}**+H]$^+$ = 230.08108; calcd. [**L_{mOMe}**+Na]$^+$ = 252.06311, found [**L_{mOMe}**+Na]$^+$ = 252.06294.

^{1}H-NMR (400 MHz, DMSO-d_6): δ (ppm) = 13.10 (s, 1H, H_8), 8.15 (m, 3H, $H_{6/10/14}$), 8.01 (t, $^3J_{HH}$ = 7.8 Hz, 1H, H_5), 7.92 (dd, $^3J_{HH}$ = 7.6 Hz, $^4J_{HH}$ = 1.0 Hz, 1H, H_4), 7.07 (m, 2H, $H_{11/13}$), 3.83 (s, 3H, H_{15}).

^{13}C{^{1}H}-NMR (101 MHz, DMSO-d_6): δ (ppm) = 166.2 (C_7), 160.5 (C_{12}), 155.8 (C_1), 148.1 (C_3), 138.5 (C_5), 130.2 (C_9), 128.3 ($C_{10/14}$), 122.5 (C_6), 122.4 (C_4), 114.2 ($C_{11/13}$), 55.3 (C_{15}).

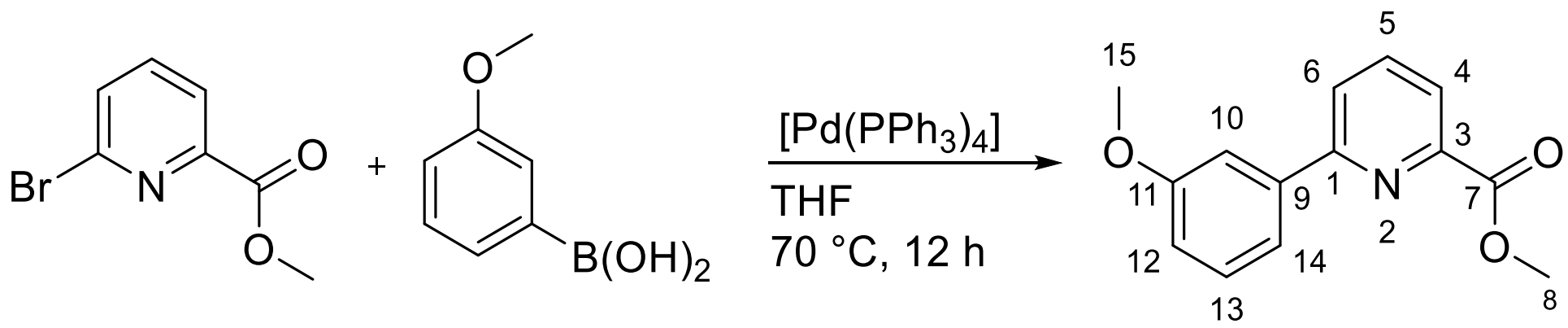

5

Intermediate compound 5. Methyl-6-bromopicolinate (350 mg, 1.62 mmol, 1.0 eq.), 3-methoxyphenylboronic acid (294 mg, 1.94 mmol, 1.2 eq.), and [Pd(PPh$_3$)$_4$] (185 mg, 0.16 mmol, 0.05 eq.) were dissolved in THF (30 mL). The mixture was purged with argon at room temperature for 5 minutes before the addition of a potassium carbonate solution (2 M, 5 mL), followed by an additional 5 minutes of argon purging. The solution was refluxed for 12 hours. The reaction was allowed to cool down to room temperature and was washed with distilled water (15 mL). The phases were separated, the aqueous phase extracted with ethyl acetate (3 × 20 mL) and the combined organic phases were dried over anhydrous sodium sulfate. The solvent was removed under reduced pressure, and the crude product was purified by flash column chromatography (SiO_2, cyclohexane/ethyl acetate = 9:1) yielding **5** (259 mg, 1.06 mmol, 67 %) as a white solid.

EM-MS-ESI (MeOH, $C_{14}H_{13}NO_3$): calcd. [**5**+H]$^+$ = 245.1001, found [**5**+H]$^+$ = 244.0972; calcd. [**5**+Na]$^+$ = 266.0788, found [**5**+Na]$^+$ = 266.0795.

^{1}H-NMR (400 MHz, $CDCl_3$): δ (ppm) = 8.05 (dd, $^3J_{HH}$ = 6.0 Hz, $^4J_{HH}$ = 2.7 Hz, 1H, H_6), 7.87 (m, 2H, $H_{4/5}$), 7.43 (m, 1H, H_{10}), 7.35 (m, 2H, $H_{12/13}$), 6.83 (dd, $^3J_{HH}$ = 5.9 Hz, $^4J_{HH}$ = 3.4 Hz, 1H, H_{14}), 4.02 (s, 3H, H_8), 3.90 (s, 3H, H_{15}).

^{13}C{^{1}H}-NMR (101 MHz, $CDCl_3$): δ (ppm) = 166.1 (C_7), 158.7 (C_1), 151.1 (C_{11}), 147.9 (C_3), 139.4 (C_9), 137.5 (C_5), 129.5 (C_{13}), 124.1 (C_6), 123.2 (C_4), 115.8 (C_{12}), 113.8 (C_{14}), 111.4 (C_{10}), 52.8 (C_8), 40.8 (C_{15}).

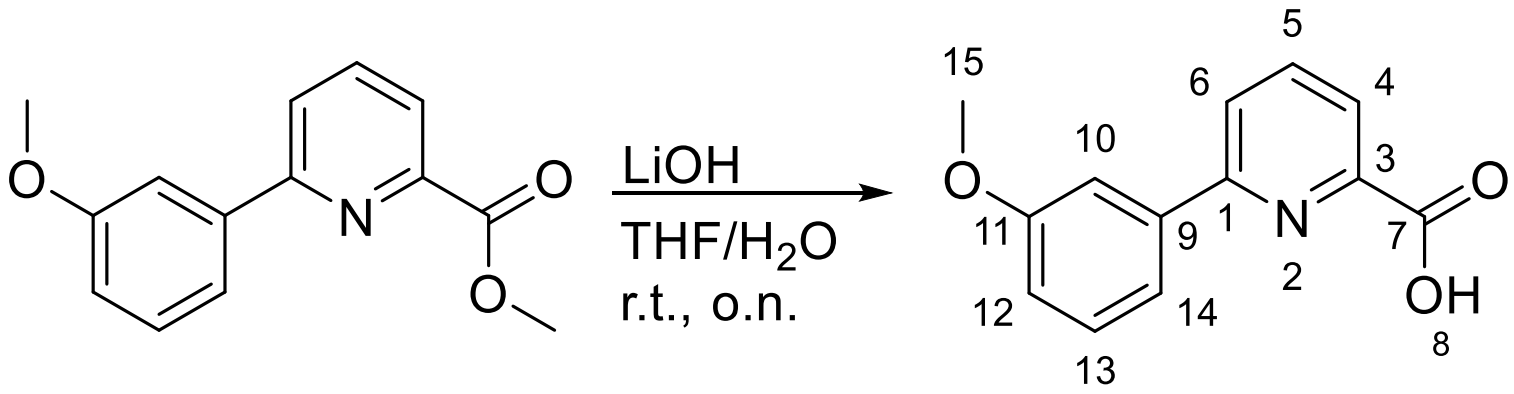

5

L$_{pOMe}$

Ligand precursor L$_{pOMe}$. Compound **5** (240 mg, 0.99 mmol, 1.0 eq.) was dissolved in THF (10 mL) and lithium hydroxide (71 mg, 2.96 mmol, 3.0 eq.) was dissolved in distilled water (10 mL). The mixture was stirred at room temperature overnight and subsequently the THF

was removed under reduced pressure. The remaining solvent was neutralized with HCl (1 M) and the resulting precipitate was filtered through a glass frit and finally was washed with distilled water. The product was dried *in vacuo* to yield $\mathbf{L_{pOMe}}$ (199 mg, 0.87 mmol, 88 %) as a white solid.

EM-MS-ESI (MeOH, $C_{13}H_{11}NO_3$): calcd. $[\mathbf{L_{pOMe}}+Na]^+$ = 252.06311, found $[\mathbf{L_{pOMe}}+Na]^+$ = 252.06304.

^{1}H-NMR (400 MHz, DMSO-d_6): δ (ppm) = 13.17 (s, 1H, H_8), 8.20 (dd, $^3J_{HH}$ = 7.8 Hz, $^4J_{HH}$ = 1.2 Hz, 1H, H_6), 8.06 (t, $^3J_{HH}$ = 7.7 Hz, 1H, H_5), 8.00 (dd, $^3J_{HH}$ = 7.7 Hz, $^4J_{HH}$ = 1.1 Hz, 1H, H_4), 7.75 (m, 2H, $H_{10/14}$), 7.44 (t, $^3J_{HH}$ = 8.2 Hz, 1H, H_{13}), 7.05 (ddd, $^3J_{HH}$ = 8.2 Hz, $^4J_{HH}$ = 2.5 Hz, $^4J_{HH}$ = 1.1 Hz, 1H, H_{12}), 3.85 (s, 3H, H_{15}).

^{13}C{^{1}H}-NMR (101 MHz, DMSO-d_6): δ (ppm) = 166.1 (C_7), 159.7 (C_{11}), 155.7 (C_1), 148.1 (C_3), 139.2 (C_9), 138.5 (C_5), 129.8 (C_{13}), 123.5 (C_4), 123.3 (C_6), 119.1 (C_{14}), 115.1 (C_{12}), 112.2 (C_{10}), 55.2 (C_{15}).

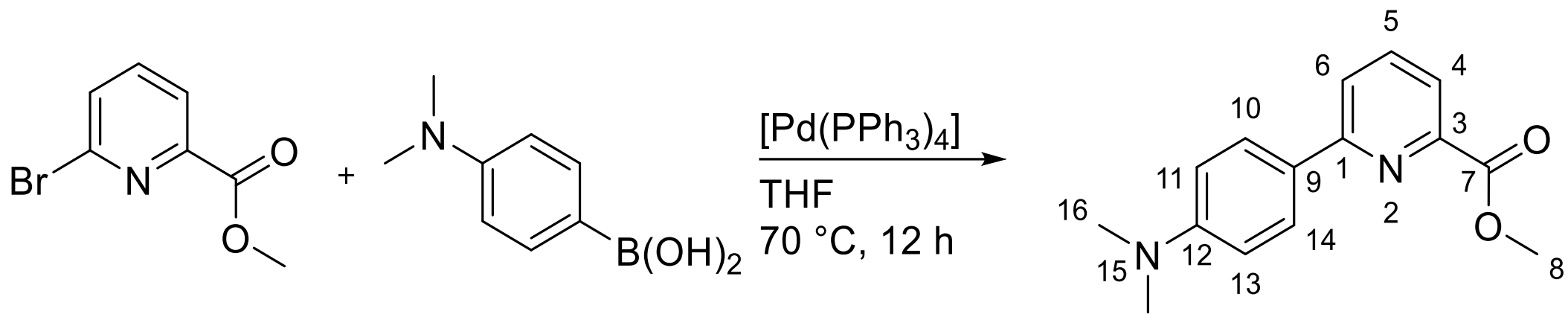

6

Intermediate compound 6. Methyl-6-bromopicolinate (300 mg, 1.41 mmol, 1.0 eq.), 4-dimethylaminophenylboronic acid (278 mg, 1.69 mmol, 1.2 eq.), and $[Pd(PPh_3)_4]$ (81 mg, 0.071 mmol, 0.05 eq.) were dissolved in THF (20 mL). The mixture was purged with argon at room temperature for 5 minutes before the addition of a potassium carbonate solution (2 M, 5 mL), followed by an additional 5 minutes of argon purging. The solution was refluxed for 12 hours. The reaction was allowed to cool down to room temperature and was washed with distilled water (15 mL). The phases were separated, the aqueous phase extracted with ethyl acetate (3 × 15 mL) and the combined organic phases were dried over anhydrous sodium sulfate. The solvent was removed under reduced pressure, and the crude product was purified by flash column chromatography (SiO_2, cyclohexane/ethyl acetate = 9:1) yielding **6** (237 mg, 0.92 mmol, 66 %) as a white solid.

EM-MS-ESI (MeOH, $C_{15}H_{16}N_2O_2$) calcd. $[\mathbf{6}+H]^+$ = 257.1285, found $[\mathbf{6}+H]^+$ = 257.1284.

^{1}H-NMR (400 MHz, $CDCl_3$): δ (ppm) = 7.98 (m, 2H, $H_{11/13}$), 7.90 (dd, $^3J_{HH}$ = 6.7 Hz, $^4J_{HH}$ = 1.8 Hz, 1H, H_6), 7.78 (m, 2H, $H_{4/5}$), 6.77 (m, 2H, $H_{10/14}$), 4.00 (s, 3H, H_8), 3.00 (s, 6H, H_{16}), 2.98 (s, 6H, H_{16}).

^{13}C{^{1}H}-NMR (101 MHz, $CDCl_3$): δ (ppm) = 166.3 (C_7), 157.8 (C_1), 151.3 (C_{12}), 147.7 (C_3), 137.2 (C_9), 137.5 (C_5), 122.1 (C_6), 121.8 (C_4), 112.2 ($C_{11/13}$), 52.7 (C_8), 40.3 (C_{16}).

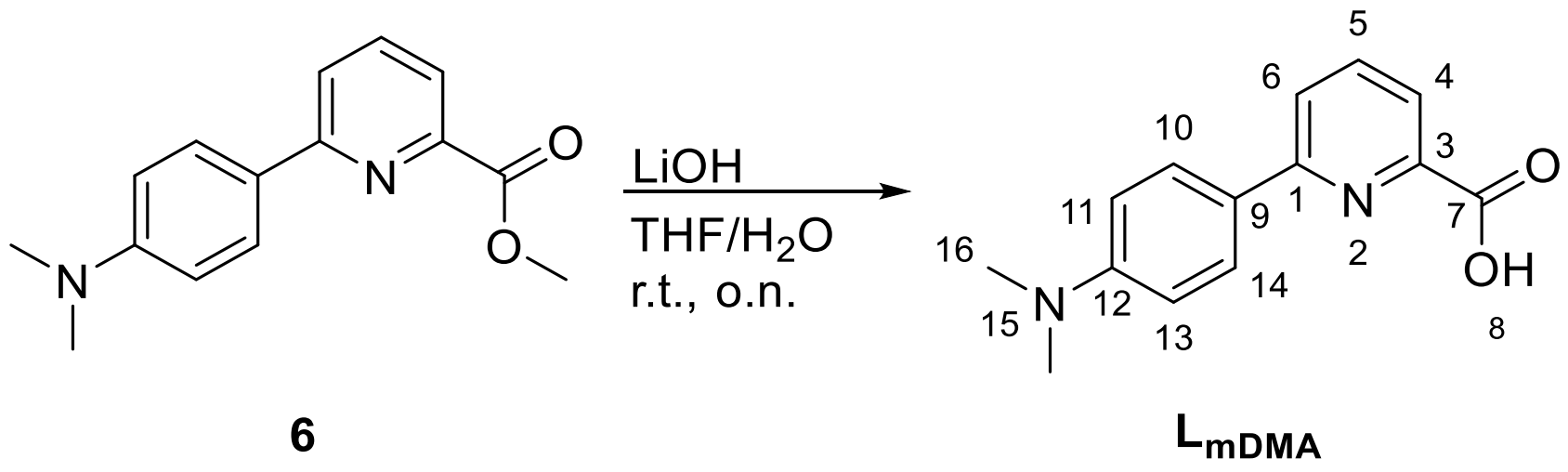

***Ligand precursor L_{mDMA}*.** Compound **6** (250 mg, 0.98 mmol, 1.0 eq.) was dissolved in THF (10 mL) and lithium hydroxide (47 mg, 1.95 mmol, 2.0 eq.) was dissolved in distilled water (10 mL). The mixture was stirred at room temperature overnight and subsequently the THF was removed under reduced pressure. The remaining solvent was neutralized with HCl (1 M) and the resulting precipitate was filtered through a glass frit and finally was washed with distilled water. The product was dried *in vacuo* to yield $\mathbf{L_{mDMA}}$ (213 mg, 0.91 mmol, 91 %) as a white solid.

EM-MS-ESI (MeOH, $C_{14}H_{14}N_2O_2$): calcd. [$\mathbf{L_{mDMA}}$-H]$^-$ = 241.09715, found [$\mathbf{L_{mDMA}}$-H]$^-$ = 241.09767.

^{1}H-NMR (400 MHz, DMSO-d_6): δ (ppm) = 8.20 (dd, $^3J_{HH}$ = 7.8 Hz, $^4J_{HH}$ = 1.2 Hz, 1H, H_6), 8.06 (t, $^3J_{HH}$ = 7.7 Hz, 1H, H_5), 8.00 (dd, $^3J_{HH}$ = 7.6 Hz, $^4J_{HH}$ = 1.1 Hz, 1H, H_4), 7.75 (m, 2H, $H_{10/14}$), 7.44 (t, $^3J_{HH}$ = 8.2 Hz, 1H, H_{13}), 7.05 (ddd, $^3J_{HH}$ = 8.2 Hz, $^4J_{HH}$ = 2.5 Hz, $^4J_{HH}$ = 1.1 Hz, 1H, H_{12}).

^{13}C{^{1}H}-NMR (101 MHz, DMSO-d_6): δ (ppm) = 166.1 (C_7), 159.7 (C_{11}), 155.7 (C_1), 148.1 (C_3), 139.2 (C_9), 138.5 (C_5), 129.8 (C_{13}), 123.5 (C_4), 123.3 (C_6), 119.1 (C_{14}), 115.1 (C_{12}), 112.2 (C_{10}), 55.16 (C_{15}).

^{15}N-NMR (41 MHz, DMSO-d_6): δ (ppm) = 307 (N_2).

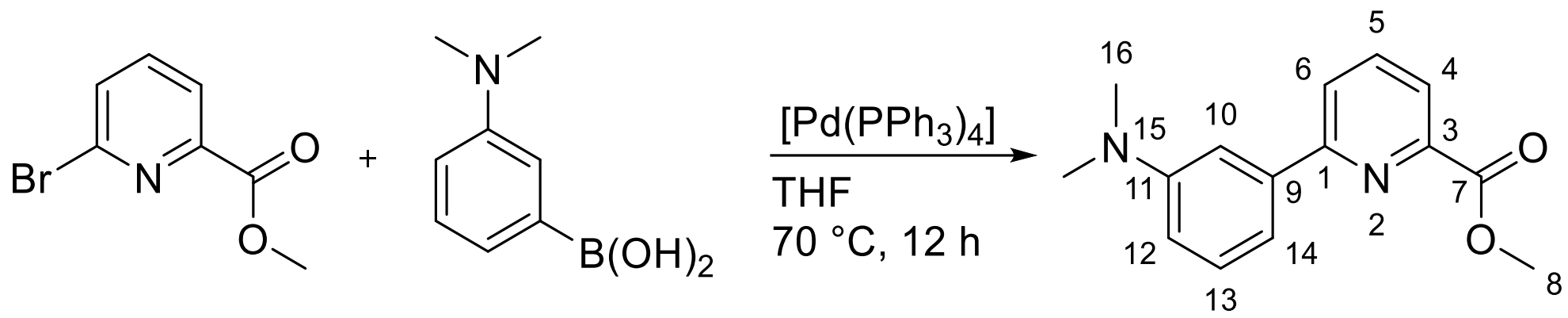

7

Intermediate compound 7. Methyl-6-bromopicolinate (300 mg, 1.41 mmol, 1.0 eq.), 3-dimethylaminophenylboronic acid (278 mg, 1.69 mmol, 1.2 eq.), and [Pd(PPh$_3$)$_4$] (81 mg, 0.071 mmol, 0.05 eq.) were dissolved in THF (20 mL). The mixture was purged with argon at room temperature for 5 minutes before the addition of a potassium carbonate solution (2 M, 5 mL), followed by an additional 5 minutes of argon purging. The solution was refluxed for 12 hours. The reaction was allowed to cool down to room temperature and was washed with distilled water (15 mL). The phases were separated, the aqueous phase extracted with ethyl acetate (3 × 15 mL) and the combined organic phases were dried over anhydrous sodium sulfate. The solvent was removed under reduced pressure, and the crude product was purified by flash column chromatography (SiO_2, cyclohexane/ethyl acetate = 9:1) yielding **7** (276 mg, 1.08 mmol, 76 %) as a white solid.

EM-MS-ESI (MeOH, $C_{15}H_{16}N_2O_3$): calcd. [**7**+H]$^+$ = 257.1285, found [**7**+H]$^+$ = 257.1289.

^{1}H-NMR (400 MHz, $CDCl_3$): δ (ppm) = 8.05 (dd, $^3J_{HH}$ = 6.0 Hz, $^4J_{HH}$ = 2.7 Hz, 1H, H_6), 7.87 (m, 2H, $H_{4/5}$), 7.43 (m, 1H, H_{10}), 7.35 (m, 2H, $H_{12/13}$), 6.83 (dd, $^3J_{HH}$ = 5.9 Hz, $^4J_{HH}$ = 3.4 Hz, 1H, H_{14}).

^{13}C{^{1}H}-NMR (101 MHz, $CDCl_3$): δ (ppm) = 166.1 (C_7), 158.7 (C_1), 151.1 (C_{11}), 147.9 (C_3), 139.4 (C_9), 137.5 (C_5), 129.5 (C_{13}), 124.1 (C_6), 123.2 (C_4), 115.8 (C_{12}), 113.8 (C_{14}), 111.4 (C_{10}), 52.8 (C_8), 40.8 (C_{16}).

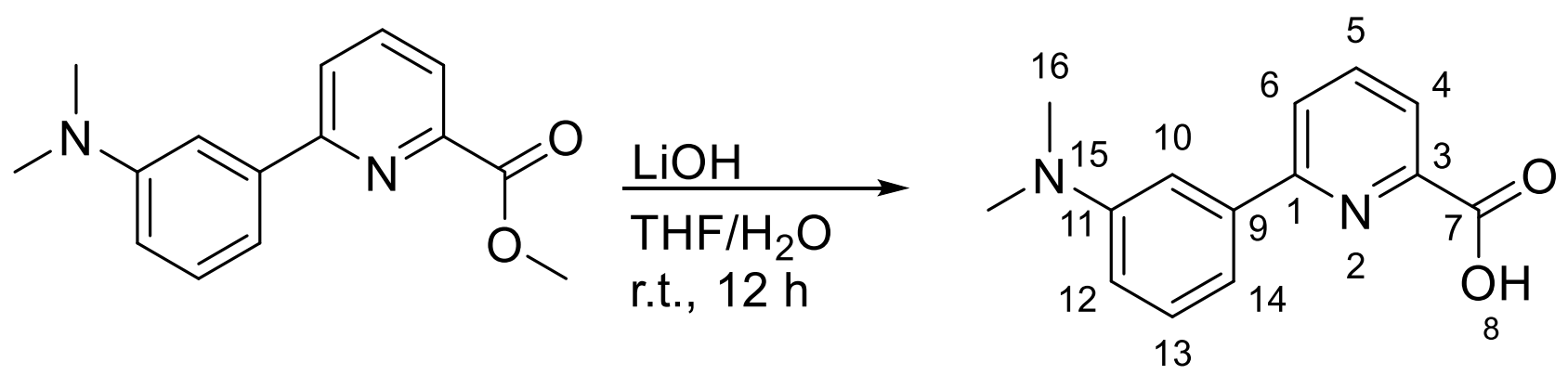

7 $\mathbf{L_{pDMA}}$

Ligand precursor L_{pDMA}. Compound **7** (250 mg, 0.98 mmol, 1.0 eq.) was dissolved in THF (10 mL) and lithium hydroxide (47 mg, 1.95 mmol, 2.0 eq.) was dissolved in distilled water (10 mL). The mixture was stirred at room temperature overnight and subsequently the THF was removed under reduced pressure. The remaining solvent was neutralized with HCl (1 M) and the resulting precipitate was filtered through a glass frit and finally was washed with

distilled water. The product was dried *in vacuo* to yield **L$_{pDMA}$** (223 mg, 0.92 mmol, 94 %) as a white solid.

EM-MS-ESI (MeOH, $C_{14}H_{14}N_2O_2$): calcd. [**L$_{pDMA}$**+H]$^+$ = 243.11280, found [**L$_{pDMA}$**+H]$^+$ = 241.11285.

^{1}H-NMR (400 MHz, DMSO-d_6): δ (ppm) = 8.11 (dd, $^3J_{HH}$ = 7.8 Hz, $^4J_{HH}$ = 1.3 Hz, 1H, H$_6$), 8.02 (t, $^3J_{HH}$ = 7.7 Hz, 1H, H$_5$), 7.97 (dd, $^3J_{HH}$ = 7.6 Hz, $^4J_{HH}$ = 1.2 Hz, 1H, H$_4$), 7.44 (dd, $^4J_{HH}$ = 2.7 Hz, $^4J_{HH}$ = 1.6 Hz, 1H, H$_{10}$), 7.39 (dd, $^3J_{HH}$ = 7.7 Hz, $^4J_{HH}$ = 1.2 Hz, 1H, H$_{12}$), 7.31 (t, $^3J_{HH}$ = 7.9 Hz, 1H, H$_{13}$), 6.83 (dd, $^3J_{HH}$ = 8.2 Hz, $^4J_{HH}$ = 2.6 Hz, $^4J_{HH}$ = 1.0 Hz, 1H, H$_{14}$)), 2.98 (s, 6H, H$_{16}$).

^{13}C{^{1}H}-NMR (101 MHz, DMSO-d_6): δ (ppm) = 166.3 (C$_7$), 156.8 (C$_1$), 150.8 (C$_{11}$), 138.6 (C$_9$), 138.3 (C$_5$), 129.2 (C$_{13}$), 123.4 (C$_6$), 122.8 (C$_4$), 115.0 (C$_{12}$), 113.4 (C$_{14}$), 110.7 (C$_{10}$), 40.2 (C$_{15}$).

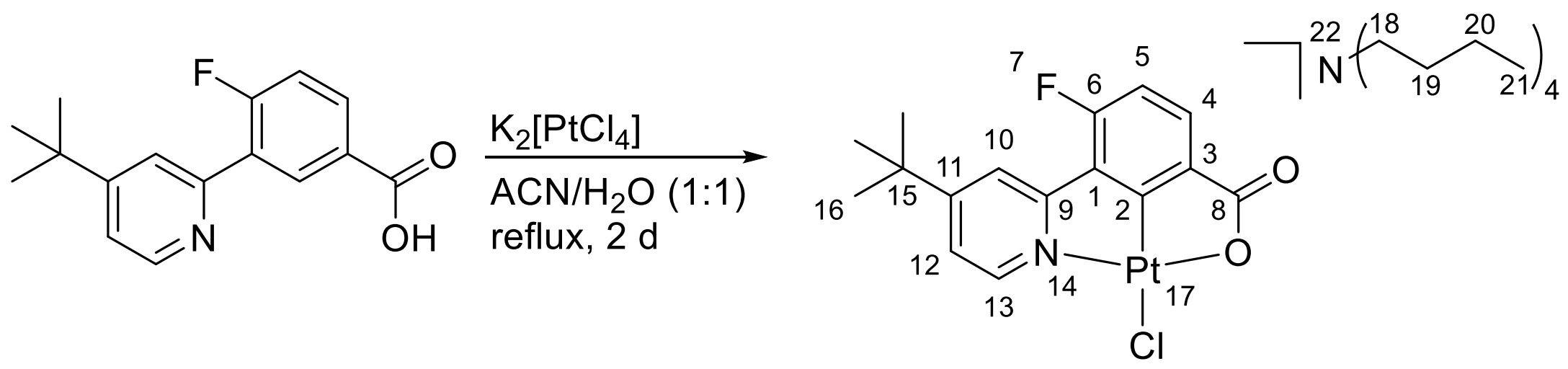

L$_{NCO}$ **Pt(L$_{NCO}$)Cl**

Complex Pt(L$_{NCO}$)Cl. $K_2[PtCl_4]$ (152 mg, 0.36 mmol, 1.0 eq.), **L$_{NCO}$** (100 mg, 0.36 mmol, 1.0 eq.), and a catalytic amount of tetrabutylammonium chloride (TBACl) were suspended in a mixture of acetonitrile/water (20 mL; 1:1 v/v). The solution was subsequently purged with argon for 10 minutes and then stirred at 100 °C for two days. The reaction mixture was cooled to room temperature and the acetonitrile was removed under reduced pressure. The resulting precipitate was filtered off and washed with water. Recrystallization from dichloromethane (DCM) with *n*-hexane yielded **Pt(L$_{NCO}$)Cl** (72 mg, 0.097 mmol, 27 %) as a yellow solid.

EM-MS-ESI (MeOH, $C_{16}H_{14}NO_2ClFPt$): calcd. [**Pt(L$_{NCO}$)Cl**]$^-$ = 501.03520, found [**Pt(L$_{NCO}$)Cl**]$^-$ = 501.03522.

^{1}H-NMR (400 MHz, DMSO-d_6): δ (ppm) = 9,01 (d, $^3J_{HH}$ = 6.0 Hz, 1H, H$_{13}$), 7.79 (m, 1H, H$_{10}$), 7.52 (d, $^3J_{HH}$ = 6.0 Hz, 1H, H$_{12}$), 7.19 (d, $^3J_{HH}$ = 8.0 Hz, $^4J_{HF}$ = 4.0 Hz, 1H, H$_4$), 6.90 (dd, $^3J_{HH}$ = 8.0 Hz, $^3J_{HF}$ = 12.0 Hz, 1H, H$_5$), 3.16 (m, 8H, H$_{18}$), 1.56 (m, 8H, H$_{19}$), 1.30 (m, 8H, H$_{20}$), 0.92 (t, $^3J_{HH}$ = 7.3 Hz, 12H, H$_{21}$).

^{13}C{^{1}H}-NMR (101 MHz, DMSO-d_6): δ (ppm) = 181.2 (C$_8$), 162.6 (C$_{9/11}$), 159.7 (d, $^1J_{CF}$ = 263.0 Hz, C$_6$), 156.2 (C$_2$), 150.8 (C$_{13}$), 131.8 (C$_3$), 130.0 (d, $^3J_{CF}$ = 8,5 Hz, C$_4$), 126.6 (C$_1$), 121.0

(C_{12}), 118.9 (C_{10}), 108.3 (d, $^2J_{CF}$ = 19.7 Hz, C_5), 54.7 (C_{18}), 35.1 (C_{15}), 29.7 (C_{16}), 23.0 (C_{19}), 19.1 (C_{20}), 13.4 (C_{21}).

^{19}F{^{1}H}-NMR (376 MHz, DMSO-*d*$_6$): δ (ppm) = - 113.0 (F_7).

^{195}Pt-NMR (107 MHz, DMSO-*d*$_6$): δ (ppm) = - 3182 (Pt_{17}).

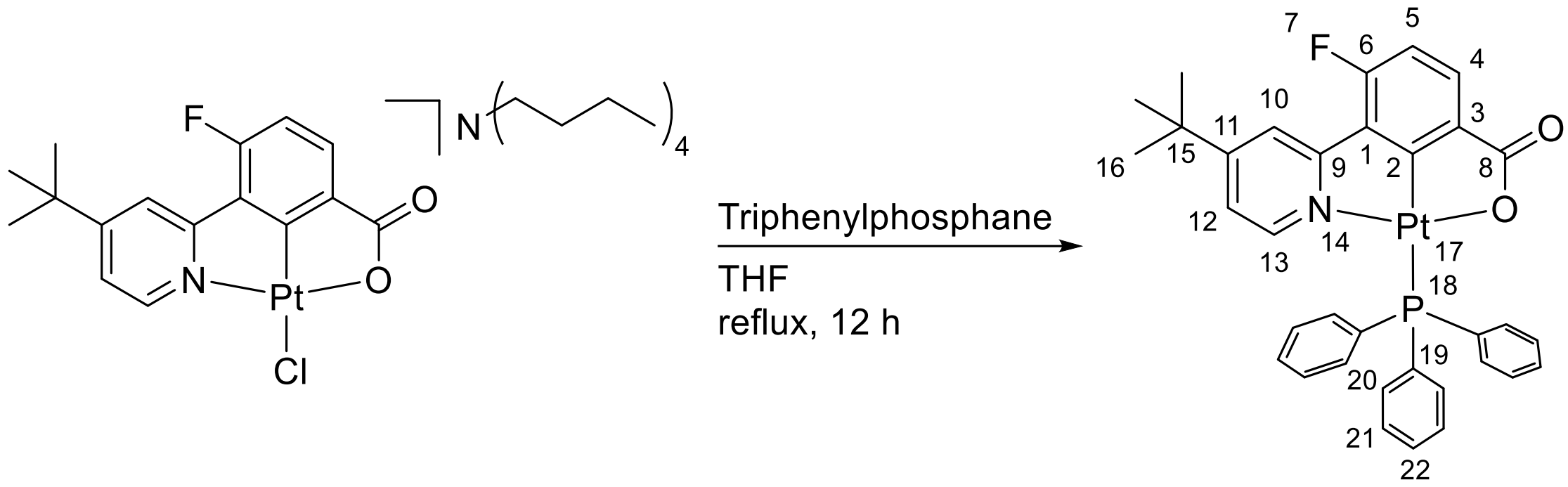

Pt(L$_{NCO}$)Cl **Pt(L$_{NCO}$)PPh$_3$**

Complex Pt(L$_{NCO}$)PPh$_3$. Complex **Pt(L$_{NCO}$)Cl** (100 mg, 0.19 mmol, 1.0 eq.) and triphenylphosphane (52 mg, 0.19 mmol, 1.0 eq.) were dissolved in THF (20 mL). The solution was purged with argon for 5 minutes and subsequently refluxed overnight. The reaction mixture was allowed to cool down to room temperature and the THF was removed under reduced pressure. The crude product was purified by flash column chromatography (SiO_2, DCM/methanol = 99:1) yielding **Pt(L$_{NCO}$)PPh$_3$** (89 mg, 0.12 mmol, 64 %) as a yellow crystalline solid.

EM-MS-ESI (MeOH, $C_{34}H_{30}NOPFPt$): calcd. [**Pt(L$_{NCO}$)PPh$_3$**+H]$^+$ = 729.16437, found [**Pt(L$_{NCO}$)PPh$_3$**+H]$^+$ = 729.16507; calcd. [**Pt(L$_{NCO}$)PPh$_3$**+Na]$^+$ = 751.14631, found [**Pt(L$_{NCO}$)PPh$_3$**+Na]$^+$ = 751.14674.

^{1}H-NMR (400 MHz, DCM-*d*$_2$): δ (ppm) = 8.00 (dd, $^4J_{HH}$ = 2.4 Hz, $^5J_{HF}$ = 1.4 Hz, 1H, H_{10}), 7.73 (m, 6H, H_{20}), 7.52 (m, 1H, H_{13}), 7.51 (m, 3H, H_{22}), 7.49 (m, 1H, H_4), 7.48 (m, 6H, H_{21}), 6.96 (dd, $^3J_{HH}$ = 8.2 Hz, $^3J_{HF}$ = 12.1 Hz, 1H, H_5), 6.62 (dd, $^3J_{HH}$ = 6.4 Hz, $^4J_{HH}$ = 2.4 Hz, 1H, H_{12}), 30.2 (s, 9H, H_{16}).

^{13}C{^{1}H}-NMR (101 MHz, DCM-*d*$_2$): δ (ppm) = 183.1 (d, $^3J_{CF}$ = 11.2 Hz, C_8), 167.3 (d, $^2J_{CP}$ = 92.5 Hz, C_2) 165.8 (dd, $^3J_{CF/CP}$ = 7.7 Hz, $^3J_{CF/CP}$ = 8.0 Hz, C_9) 165.0 (C_{11}), 160.4 (d, $^1J_{CF}$ = 246.0 Hz, C_6), 150.1 (d, $^3J_{CP}$ = 3.6 Hz, C_{13}), 134.9 (d, $^3J_{CP}$ = 12.9 Hz, C_{20}), 131.9 (d, $^3J_{CP}$ = 43.3 Hz, C_{19}), 131.2 (d, $^4J_{CP}$ = 2.1 Hz, C_{10}), 131.0 (dd, $^3J_{CF}$ = 8.2 Hz, $^4J_{CP}$ = 5.5 Hz, C_4), 129.3 (d, $^3J_{CP}$ = 10.1 Hz, C_{21}), 128.7 (d, $^2J_{CF}$ = 7.7 Hz, C_1), 121.4 (d, $^3J_{CF}$ = 12.0 Hz, C_{10}), 121.0 (C_{12}), 112.9 (d, $^2J_{CF}$ = 23.4 Hz, C_5), 35.7 (C_{15}), 30.2 (C_{16}).

^{19}F{^{1}H}-NMR (376 MHz, DCM-d_2): δ (ppm) = - 112.1 ($^4J_{FPt}$ = 28.3Hz, $^5J_{FP}$ = 8.7 Hz, F_7).

^{31}P{^{1}H}-NMR (162 MHz, DCM-d_2): δ (ppm) = 30.2 ($^1J_{PPt}$ = 2328.8 Hz, $^5J_{PF}$ = 8.7 Hz, P_{18}).

^{15}N-NMR (41 MHz, DCM-d_2): δ (ppm) = 190 (N_{14}).

^{195}Pt-NMR (86 MHz, DCM-d_2): δ (ppm) = - 3520 ($^1J_{PtP}$ = 2328.8 Hz, Pt_{17}).

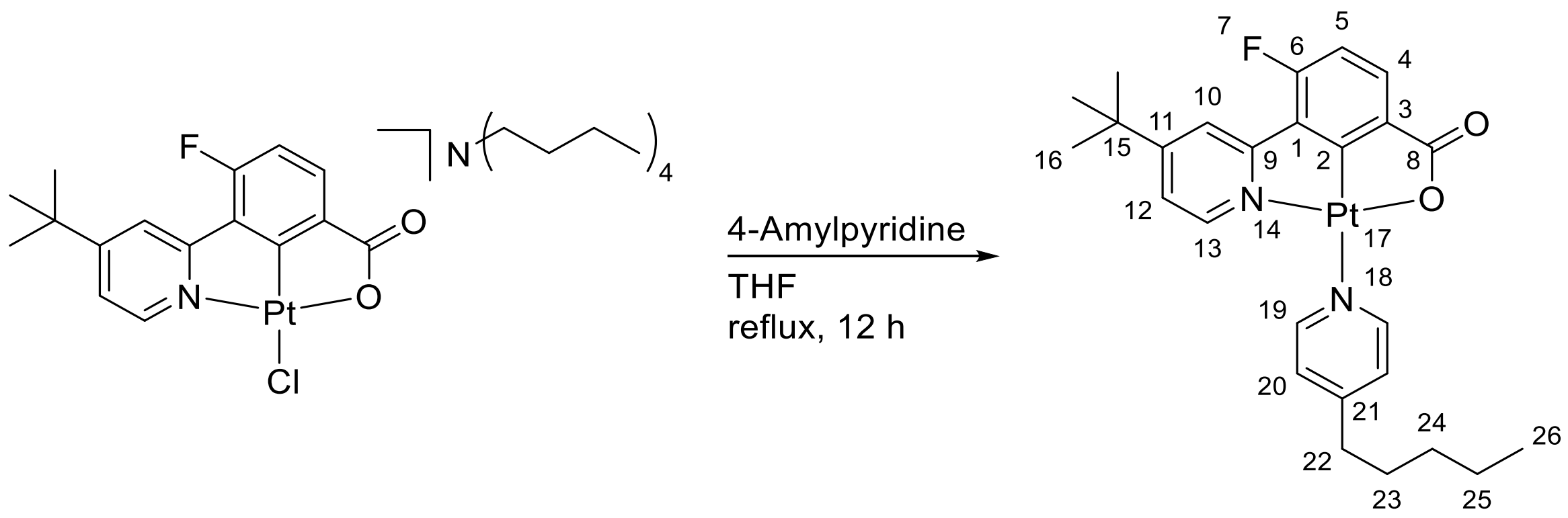

Pt(L_{NCO})Cl **Pt(L_{NCO})AmPy**

Complex Pt(L_{NCO})AmPy. Complex **Pt(L_{NCO})Cl** (70 mg, 0.14 mmol, 1.0 eq.) and 4-amylpyridine (21 mg, 23 µL, 0.14 mmol, 1.0 eq.) were dissolved in THF (15 mL). The solution was purged with argon for 5 minutes and subsequently refluxed overnight. The reaction mixture was allowed to cool down to room temperature and the THF was removed under reduced pressure. The crude product was purified by flash column chromatography (SiO_2, DCM/methanol = 98:2) yielding **Pt(L_{NCO})AmPy** (63 mg, 0.10 mmol, 73 %) as a yellow crystalline solid.

EM-MS-ESI (MeOH, $C_{26}H_{30}N_2O_2FPt$): calcd. [**Pt(L_{NCO})AmPy**+H]$^+$ = 616.19361, found [**Pt(L_{NCO})AmPy**+H]$^+$ = 616.19388.

^{1}H-NMR (500 MHz, DCM-d_2): δ (ppm) = 8.76 (m, 2H, H_{19}), 7.97 (d, $^3J_{HH}$ = 6.1 Hz, 1H, H_{13}), 7.70 (m, 1H, H_{10}), 7.24 (m, 2H, H_{20}), 7.09 (dd, $^3J_{HH}$ = 6.1 Hz, $^4J_{HH}$ = 2.1 Hz, 1H, H_{12}), 6.97 (dd, $^3J_{HH}$ = 8.1 Hz, $^4J_{HF}$ = 4.2 Hz, 1H, H_4), 6.68 (dd, $^3J_{HH}$ = 8.1 Hz, $^3J_{HF}$ = 12.0 Hz, 1H, H_5), 2.61 (m, 2H, H_{22}), 1.62 (m, 2H, H_{23}), 1.38 (s, 6H, H_{16}), 1.36 (m, 4 H, $H_{24/25}$), 0.92 (t, $^3J_{HH}$ = 7.1 Hz, 4H, H_{26}).

^{13}C{^{1}H}-NMR (126 MHz, DCM-d_2): δ (ppm) = 181.3 (C_8), 164.0 (d, $^3J_{CF}$ = 6.7 Hz, C_9), 163.6 (C_{11}), 159.7 (d, $^1J_{CF}$ = 263.0 Hz, C_6), 156.7 (d, $^3J_{FC}$ = 2.4 Hz, C_2), 155.0 (C_{21}), 151.6 (C_{19}), 150.4 (C_{13}), 132.6 (d, $^4J_{CF}$ = 2.6 Hz, C_3), 130.5 (d, $^3J_{CF}$ = 8.5 Hz, C_4), 128.3 (d, $^2J_{CF}$ = 8.1 Hz, C_1), 126.1 (C_{20}), 121.2 (d, $^3J_{CF}$ = 10.0 Hz, C_{10}), 121.1 (C_{12}), 110.4 (d,$^2J_{CF}$ = 23.2 Hz, C_5), 35.7 (C_{15}), 35.6 (C_{22}), 31.7 (C_{24}), 30.4 (C_{16}), 30.0 (C_{23}), 22.8 (C_{25}), 14.1 (C_{26}).

^{19}F{^{1}H}-NMR (471 MHz, DCM-d_2): δ (ppm) = - 112.6 (F_7).

^{15}N-NMR (51 MHz, DCM-*d*$_2$): δ (ppm) = 253 (N_{18}), 195 (N_{14}).

^{195}Pt-NMR (107 MHz, DCM-*d*$_2$): δ (ppm) = - 3291 (Pt_{17}).

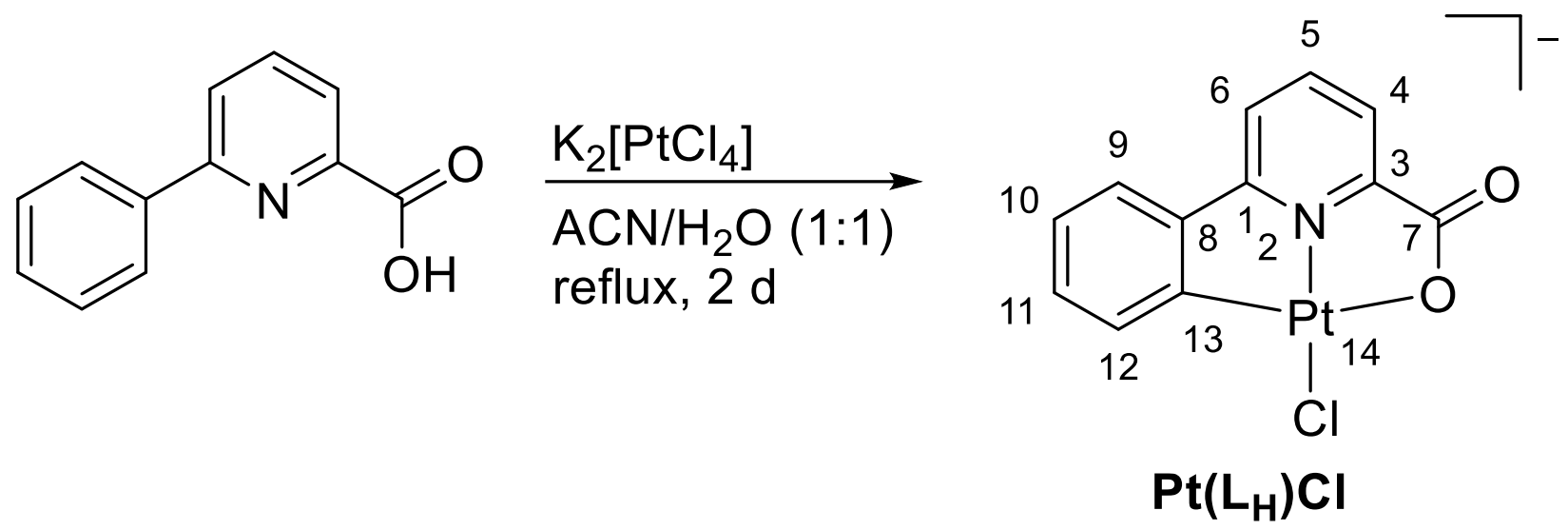

***Complex Pt(L$_H$)Cl*.** 6-Phenylpicolinic acid (75 mg, 0.38 mmol, 1.0 eq.), $K_2[PtCl_4]$ (156 mg, 0.38 mmol, 1.0 eq.) and a catalytic amount of TBACl were suspended in a mixture of acetonitrile/water (30 mL; 1:1 v/v). The solution was purged with argon for 10 minutes and subsequently stirred at 100 °C for two days. The reaction mixture was cooled to room temperature and the acetonitrile was removed under reduced pressure. The resulting precipitate was filtered off and washed with water. The crude product was purified by flash column chromatography (SiO_2, DCM/methanol = 96:4) yielding **Pt(L$_H$)Cl** (127 mg, 0.29 mmol, 78 %) as a yellow crystalline solid.

EM-MS-ESI (MeOH, $C_{12}H_7N_1O_2ClPt$): calcd. [**Pt(L$_H$)Cl**+H]$^+$ = 427.98104, found [**Pt(L$_H$)Cl**+H]$^+$ = 427.98137.

^{1}H-NMR (500 MHz, DCM-*d*$_2$): δ (ppm) = 7.74 (t, $^3J_{HH}$ = 7.9 Hz, 1H, H_5), 7.49 (td, $^3J_{HH}$ = 6.8 Hz, $^4J_{HH}$ = 1.9 Hz, 1H, H_4), 7.32 (dd, $^3J_{HH}$ = 8.2 Hz, $^3J_{HH}$ = 2.2 Hz, 1H, H_6), 7.18 (d, $^3J_{HH}$ = 7.6 Hz, 1H, H_9), 6.98 (td, $^3J_{HH}$ = 7.4 Hz, $^4J_{HH}$ = 1.5 Hz, 1H, H_{10}), 6.87 (m, 2H, H_{11-12}).

^{13}C{^{1}H}-NMR (126 MHz, DCM-*d*$_2$): δ (ppm) = 174.1 (C_7), 164.8 (C_1), 150.3 (C_3), 144.6 (C_8), 140.5 (C_5), 133.2 (C_{12}), 131.1 (C_{11}), 124.7 (C_9), 124.6 (C_{10}), 122.8 (C_4), 120.5 (C_6).

^{195}Pt-NMR (86 MHz, DCM-*d*$_2$): δ (ppm) = - 3466 (Pt_{14}).

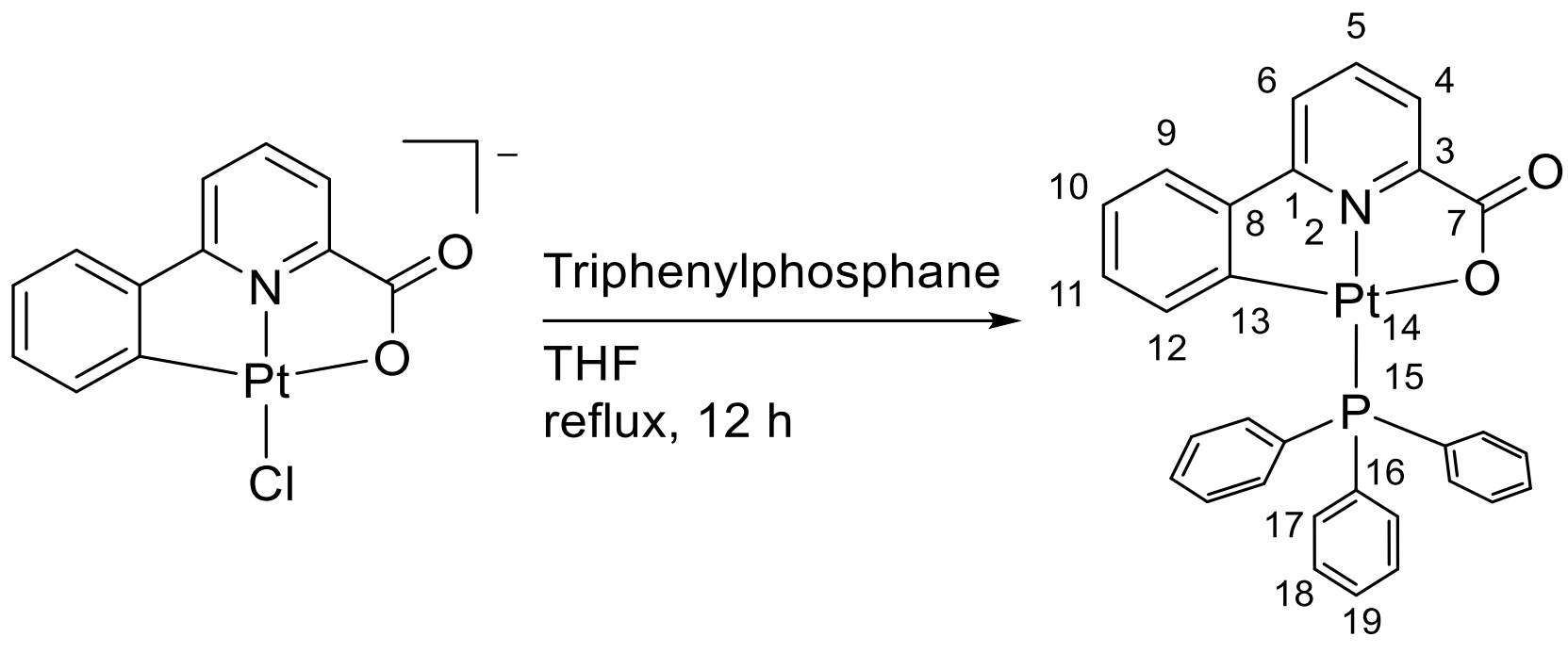

Complex Pt(L$_H$)PPh$_3$. Complex **Pt(L$_H$)Cl** (100 mg, 0.23 mmol, 1.0 eq.) and triphenylphosphane (61 mg, 0.23 mmol, 1.0 eq.) were dissolved in THF (10 mL). The solution was purged with argon for 5 minutes and subsequently refluxed overnight. The reaction mixture was allowed to cool down to room temperature and the THF was removed under reduced pressure. The crude product was purified by flash column chromatography (SiO_2, DCM/methanol = 99:1) yielded **Pt(L$_H$)PPh$_3$** (133 mg, 0.20 mmol, 88 %) as a yellow crystalline solid.

EM-MS-ESI (MeOH, $C_{30}H_{23}NO_2PPt$): calcd. [**Pt(L$_H$)PPh$_3$**+H]$^+$ = 655.11116, found [**Pt(L$_H$)PPh$_3$**+H]$^+$ = 655.11173; calcd. [**Pt(L$_H$)PPh$_3$**+Na]$^+$ = 677.09310, found [**Pt(L$_H$)PPh$_3$**+Na]$^+$ = 677.09342.

^{1}H-NMR (500 MHz, DCM-d_2): δ (ppm) = 8.02 (t, $^3J_{HH}$ = 7.9 Hz, 1H, H$_5$), 7.80 (dt, $^3J_{HH}$ = 8.1 Hz, $^4J_{HH}$ = 1.3 Hz, 1H, H$_6$), 7.75 (dt, $^3J_{HH}$ = 7.6 Hz, $^4J_{HH}$ = 1.2 Hz, 1H, H$_4$), 7.71 (m, 6H, H$_{17}$), 7.52 (m, 4H, H$_{9/19}$), 7.45 (m, 6H, H$_{18}$), 7.01 (td, $^3J_{HH}$ = 7.5 Hz, $^4J_{HH}$ = 1.2 Hz, 1H, H$_{10}$), 6.66 (td, $^3J_{HH}$ = 7.6 Hz, $^3J_{HH}$ = 1.5 Hz, 1H, H$_{11}$), 6.48 (dt, $^3J_{HH}$ = 7.8 Hz, $^3J_{HH}$ = 1.5 Hz, 1H, H$_{12}$).

^{13}C{^{1}H}-NMR (126 MHz, DCM-d_2): δ (ppm) = 173.7 (d, $^3J_{CP}$ = 4.3 Hz, C$_7$), 162.5 (d, $^3J_{CP}$ = 1.7 Hz, C$_1$), 150.6 (d, $^3J_{CP}$ = 1.7 Hz, C$_3$), 146.2 (d, $^3J_{CP}$ = 1.6 Hz), 141.3 (C$_5$), 139.0 (d, $^3J_{CP}$ = 4.9 Hz, C$_{12}$), 135.2 (d, $^2J_{CP}$ = 11.9 Hz, C$_{17}$), 132.7 (d, $^2J_{CP}$ = 7.1 Hz, C$_{13}$), 131.6 (d, $^4J_{CP}$ = 2.5 Hz, C$_{19}$), 130.9 (d, $^4J_{CP}$ = 1.7 Hz, C$_{11}$), 130.0 (d, $^1J_{CP}$ = 59.5 Hz), 129.0 (d, $^3J_{CP}$ = 11.1 Hz, C$_{18}$), 124.6 (C$_{9/10}$), 123.2 (d, $^4J_{CP}$ = 3.0 Hz, C$_4$), 120.3 (d, $^4J_{CP}$ = 3.2 Hz, C$_6$).

^{31}P{^{1}H}-NMR (162 MHz, DCM-d_2): δ (ppm) = 23.2 ppm ($^1J_{PPt}$ = 4089.4 Hz, P$_{15}$).

^{15}N-NMR (51 MHz, DCM-d_2): δ (ppm) = 245 (N$_2$).

^{195}Pt-NMR (107 MHz, DCM-d_2): δ (ppm) = - 3996 ($^1J_{PtP}$ = 4089.4 Hz, Pt$_{14}$).

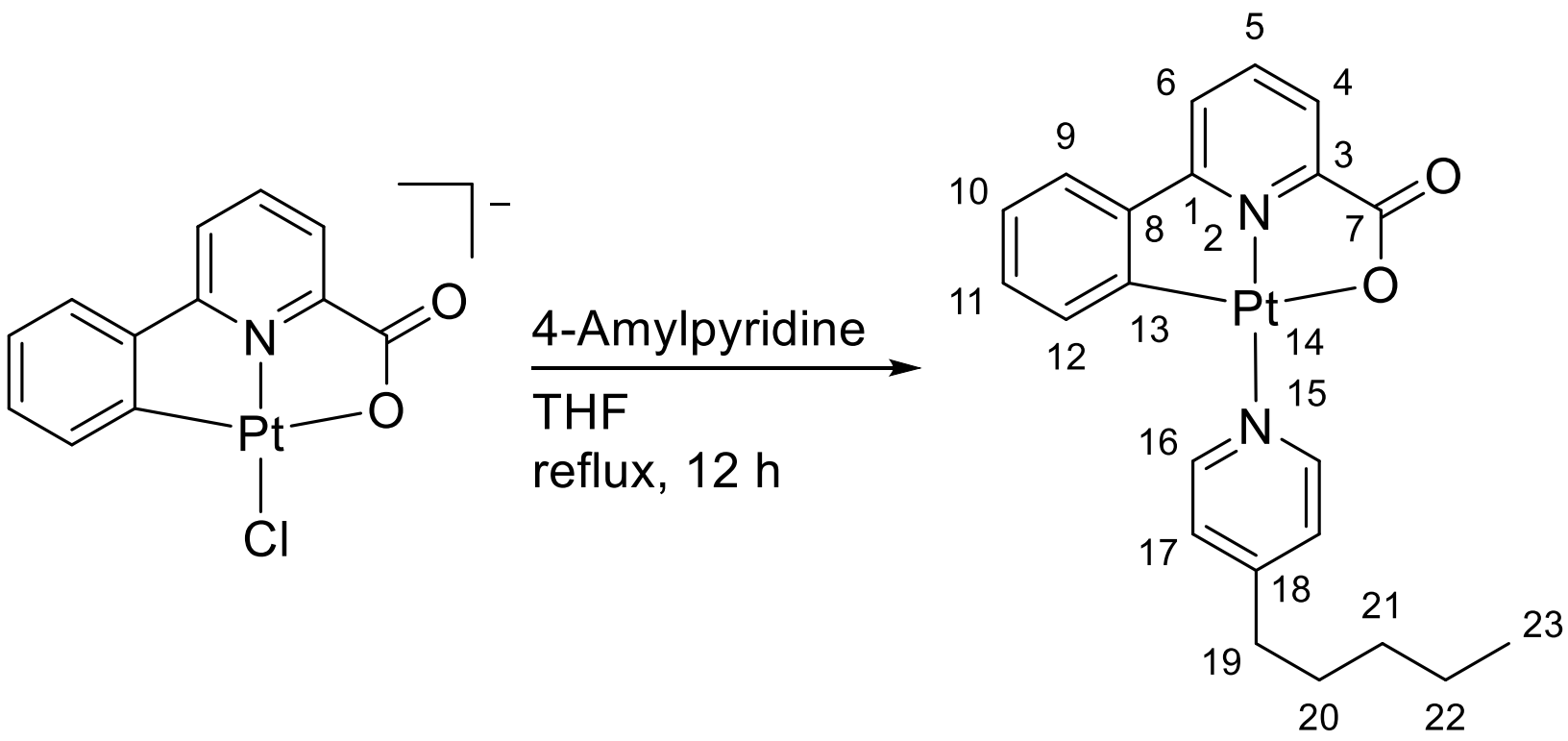

Pt(L$_H$)Cl **Pt(L$_H$)AmPy**

***Complex Pt(L$_H$)AmPy*.** **Pt(L$_H$)Cl** (100 mg, 0.23 mmol, 1.0 eq.) and 4-amylpyridine (35 mg, 38 µL, 0.23 mmol, 1.0 eq.) were dissolved in THF (10 mL). The solution was purged with argon for 5 minutes and subsequently refluxed overnight. The reaction mixture was allowed to cool down to room temperature and the THF was removed under reduced pressure. The crude product was purified by flash column chromatography (SiO_2, DCM/methanol = 99:1) yielding **Pt(L$_H$)AmPy** (92 mg, 0.17 mmol, 73 %) as a yellow crystalline solid.

EM-MS-ESI (MeOH, $C_{22}H_{23}N_2O_2Pt$): calcd. [**Pt(L$_H$)AmPy** +H]$^+$ = 542.14039, found [**Pt(L$_H$)AmPy**+H]$^+$ = 542.14037; calcd. [**Pt(L$_H$)AmPy**+Na]$^+$ = 564.12234, found [**Pt(L$_H$)AmPy**+Na]$^+$ = 564.12225.

^{1}H-NMR (500 MHz, DCM-d_2): δ (ppm) = 8.75 (m, 2H, H_{16}), 7.86 (t, $^3J_{HH}$ = 7.9 Hz, 1H, H_5), 7.57 (d, $^3J_{HH}$ = 8.2 Hz, 1H, H_6), 7.53 (d, $^3J_{HH}$ = 7.7 Hz, 1H, H_4), 7.40 (m, 1H, H_9), 7.31 (m, 2H, H_{17}), 7.05 (m, 1H, H_{11}), 7.04 (m, 1H, H_{10}), 6.63 (m, 1H, H_{12}), 2.69 (m, 2H, H_{19}), 1.68 (m, 2H, H_{20}), 1.35 (m, 4H, $H_{21/22}$), 0.90 (t, $^3J_{HH}$ = 7.0 Hz, 3H, H_{23}).

^{13}C{^{1}H}-NMR (126 MHz, DCM-d_2): δ (ppm) = 174.3 (C_7), 164.5 (d, $^2J_{CPt}$ = 115 Hz, C_1), 156.1 (C_{18}), 152.7 (C_{16}), 150.8 (C_3), 145.8 (d, $^2J_{CPt}$ = 77.0 Hz, C_8), 140.1 (C_5), 134.4 (C_{13}), 132.3 (d, $^2J_{CPt}$ = 48 Hz, C_{12}), 130.8 (C11), 126.8 (d, $^3J_{CPt}$ = 42 Hz, C_{17}), 124.9 (C_9), 122.9 (C_4), 124.5 (C_{10}), 120.5 (C_6), 35.6 (C_{19}), 31.7 (C_{21}), 29.9 (C_{20}), 22.8 (C_{22}), 14.0 (C_{23}).

^{15}N-NMR (51 MHz, DCM-d_2): δ (ppm) = 217 (N_2), 213 (N_{17}).

^{195}Pt-NMR (107 MHz, DCM-d_2): δ (ppm) = - 3246.4 (Pt_{14}).

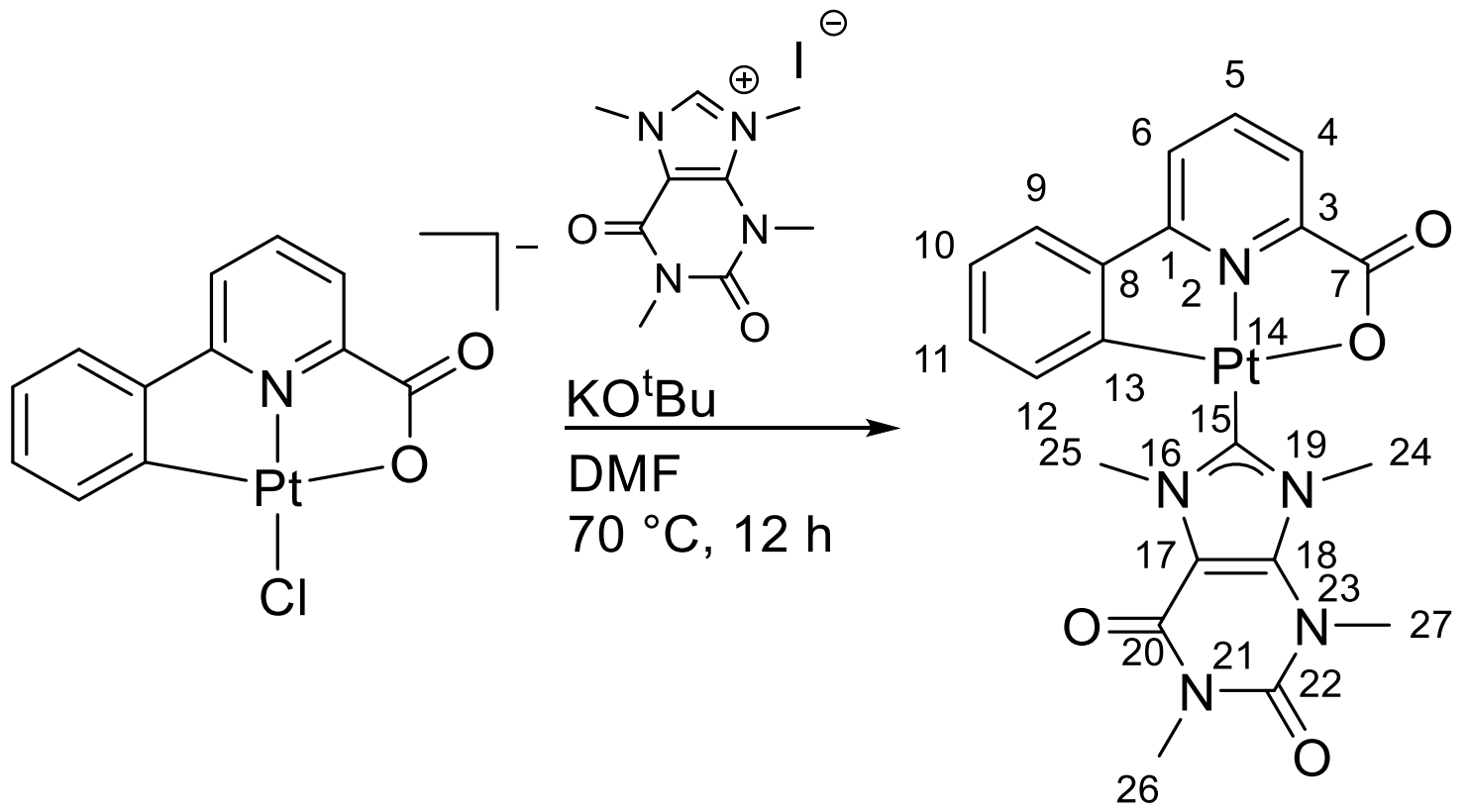

Pt(L$_H$)Cl **Pt(L$_H$)NHC**

***Complex Pt(L$_H$)NHC*.** Complex **Pt(L$_H$)Cl** (87 mg, 0.20 mmol, 1.0 eq.), 9-methylcaffeine-ium iodide (101 mg, 0.30 mmol, 1.5 eq.) and KO^{t}Bu (34 mg, 0.30 mmol, 1.5 eq.) were dissolved in N,N-dimethylformamide (DMF, 15 mL). The solution was purged with argon for 5 minutes and subsequently stirred for 12 hours at 70 °C. The reaction mixture was allowed to cool down to room temperature and the DMF was removed under reduced pressure. The crude product was purified by flash column chromatography (SiO_2, cyclohexane/acetone = 1:1) yielding **Pt(L$_H$)NHC** (58 mg, 0.096 mmol, 48 %) as a yellow crystalline solid.

EM-MS-ESI (MeOH, $C_{21}H_{20}N_5O_4Pt$): calcd. [**Pt(L$_H$)NHC**+H]$^+$ = 601.11591, found [**Pt(L$_H$)NHC**+H]$^+$ = 601.11641; calcd. [**Pt(L$_H$)NHC**+Na]$^+$ = 623.09786, found [**Pt(L$_H$)NHC**+Na]$^+$ = 623.09813.

^{1}H-NMR (500 MHz, DCM-d_2): δ (ppm) = 8.01 (t, $^3J_{HH}$ = 7.9 Hz, 1H, H$_5$), 7.75 (m, 2H, H$_{4/6}$), 7.54 (dd, $^3J_{HH}$ = 7.7 Hz, $^4J_{HH}$ = 1.5 Hz, 1H, H$_9$), 7.10 (td, $^3J_{HH}$ = 7.5 Hz, $^4J_{HH}$ = 1.3 Hz, 1H, H$_{10}$), 6.98 (td, $^3J_{HH}$ = 7.5 Hz, $^4J_{HH}$ = 1.5 Hz, 1H, H$_{11}$), 6.62 (dd, $^3J_{HH}$ = 7.5 Hz, $^4J_{HH}$ = 1.2 Hz, 1H), 4.36 (s, 3H, H$_{24}$), 4.19 (s, 3H, H$_{25}$), 3.81 (s, 3H, H$_{27}$), 3.38 (s, 3H, H$_{26}$).

^{13}C{^{1}H}-NMR (126 MHz, DCM-d_2): δ (ppm) = 174.5 (C$_{17}$), 173.0 (C$_{15}$), 162.6 (C$_1$), 153.7 (C$_{20}$), 151.1 (C$_{21}$), 149.8 (C$_3$), 145.6 (C$_8$), 140.5 (C$_5$), 136.5 (C$_{12}$), 133.7 (C$_{13}$), 131.3 (C$_{11}$), 124.8 (C$_9$), 124.4 (C$_{10}$), 123.8 (C$_4$), 120.2 (C$_6$), 110.1 (C$_{17}$), 39.2 (C$_{24}$), 37.8 (C$_{25}$), 32.3 (C$_{29}$), 28.8 (C$_{27}$).

^{15}N-NMR (51 MHz, DCM-d_2): δ (ppm) = 240 (N$_2$), 166 (N$_{19}$), 159 (N$_{16}$), 150 (N$_{23}$), 108 (N$_{21}$).

^{195}Pt-NMR (107 MHz, DCM-d_2): δ (ppm) = - 3657.5 (Pt$_{14}$).

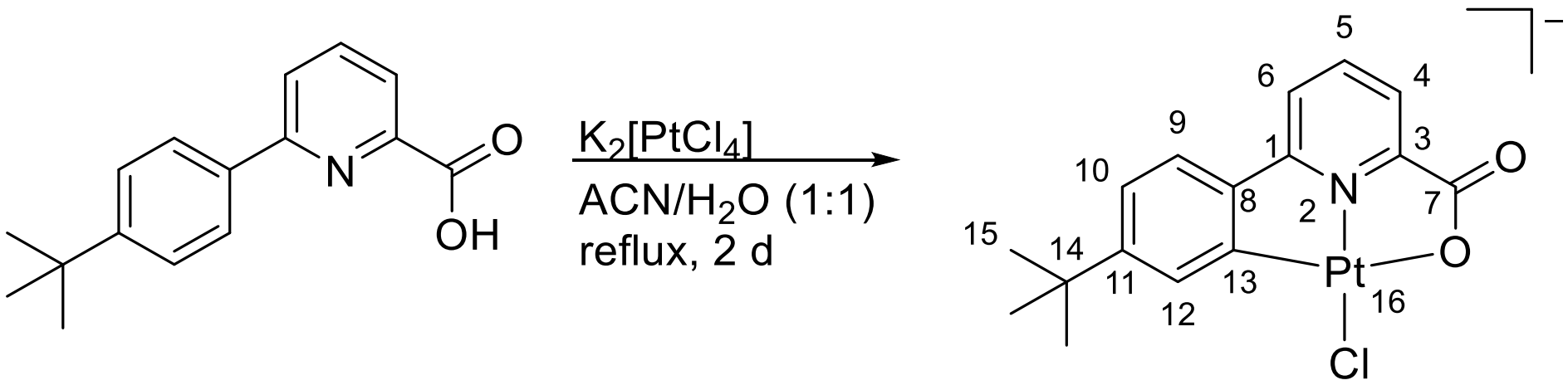

L$_{tBu}$ **Pt(L**$_{tBu}$**)Cl**

Complex Pt(L$_{tBu}$***)Cl.*** $K_2[PtCl_4]$ (81 mg, 0.20 mmol, 1.0 eq.), **L**$_{tBu}$ (50 mg, 0.20 mmol, 1.0 eq.), and a catalytic amount of TBACl were suspended in a mixture of acetonitrile/water (20 mL; 1:1 v/v). The solution was purged with argon for 10 minutes and subsequently stirred at 100 °C for two days. The reaction mixture was cooled to room temperature and the acetonitrile was removed under reduced pressure. The resulting precipitate was filtered off and washed with water. The crude product was purified by flash column chromatography (SiO_2, DCM/methanol = 96:4) yielding **Pt(L**$_{tBu}$**)Cl** (63 mg, 0.10 mmol, 73 %) as a yellow crystalline solid.

EM-MS-ESI (MeOH, $C_{16}H_{15}NO_2ClPt$): calcd. [**Pt(L**$_{tBu}$**)Cl**]$^-$ = 483.04462, found [**Pt(L**$_{tBu}$**)Cl**]$^-$ = 483.04414.

^{1}H-NMR (500 MHz, DCM-d_2): δ (ppm) = 7.84 (t, $^3J_{HH}$ = 7.9 Hz, 1H, H_5), 7.56 (dd, $^3J_{HH}$ = 7.7 Hz, $^4J_{HH}$ = 1.1 Hz, 1H, H_4), 7.50 (dd, $^3J_{HH}$ = 8.1 Hz, $^4J_{HH}$ = 1.1 Hz, 1H, H_6), 7.32 (d, $^3J_{HH}$ = 8.1 Hz, 1H, H_9), 7.17 (dd, $^3J_{HH}$ = 8.1 Hz, $^4J_{HH}$ = 1.9 Hz, 1H, H_{10}) 7.13 (d, $^4J_{HH}$ = 1.9 Hz, 1H, H_{12}), 1.34 (s, 9H, H_{15}).

^{13}C{^{1}H}-NMR (101 MHz, DCM-d_2): δ (ppm) = 173.2 (C_7), 164.8 (C_1), 15.8 (C_{14}), 151.1 (C_3), 142.0 (C_8), 140.4 (C_5), 133.2 (C_{13}), 130.3 (C_{12}), 124.4 (C_9), 122.4 (C_4), 122.1 (C_{10}), 119.9 (C_6), 32.3 (C_{14}), 31.4 (C_{15}).

^{195}Pt-NMR (86 MHz, DCM-d_2): δ (ppm) = - 3291 (Pt_{16}).

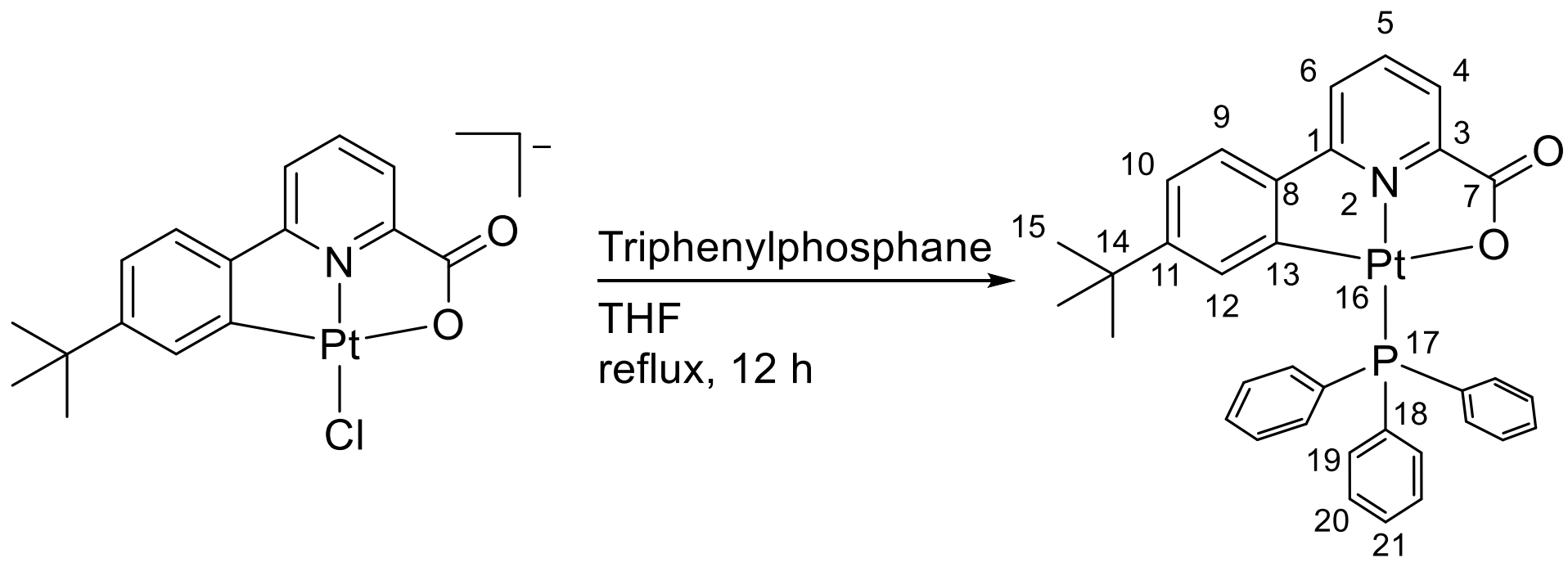

Pt(L$_{tBu}$)Cl **Pt(L$_{tBu}$)PPh$_3$**

Complex Pt(L$_{tBu}$)PPh$_3$. Complex **Pt(L$_{tBu}$)Cl** (97 mg, 0.20 mmol, 1.0 eq.) and triphenylphosphane (52 mg, 0.20 mmol, 1.0 eq.) were dissolved in THF (15 mL). The solution was purged with argon for 5 minutes and subsequently refluxed overnight. The reaction mixture was allowed to cool down to room temperature and the THF was removed under reduced pressure. The crude product was purified by flash column chromatography (SiO_2, DCM/methanol = 98:2), followed by re-crystallization from *n*-hexane in DCM, yielding **Pt(L$_{tBu}$)PPh$_3$** (120 mg, 0.17 mmol, 85 %) as a yellow crystalline solid.

EM-MS-ESI (MeOH, $C_{34}H_{30}NO_2PPt$): calcd. [**Pt(L$_{tBu}$)PPh$_3$**+H]$^+$ = 711.17379, found [**Pt(L$_{tBu}$)PPh$_3$**+H]$^+$ = 711.17415; calcd. [**Pt(L$_{tBu}$)PPh$_3$**+Na]$^+$ = 733.15574, found [**Pt(L$_{tBu}$)PPh$_3$**+Na]$^+$ = 733.15585.

^{1}H-NMR (500 MHz, DCM-*d*$_2$): δ (ppm) = 8.02 (t, $^3J_{HH}$ = 7.9 Hz, 1H, H$_5$), 7.80 (dd, $^3J_{HH}$ = 8.1 Hz, $^4J_{HH}$ = 1.0 Hz, 1H, H$_6$), 7.77 (m, 6H, H$_{19}$), 7.53 (m, 10H, H$_{9/20/21}$), 7.08 (dd, $^3J_{HH}$ = 8.1 Hz, $^4J_{HH}$ = 1.9 Hz, 1H, H$_{10}$), 6.70 (d, $^4J_{HH}$ = 1.8 Hz, 1H, H$_{12}$), 0.84 (s, 9H, H$_{15}$).

^{13}C{^{1}H}-NMR (101 MHz, DCM-*d*$_2$): δ (ppm) = 173.8 ($^3J_{CP}$ = 4.3 Hz, C$_7$), 162.4 ($^2J_{CPt}$ = 3.8 Hz, C$_1$), 154.4 (C$_{11}$), 150.6 (C$_3$), 143.3 (C$_8$), 141.2 (C$_5$), 137.6 ($^3J_{CP}$ = 5.3 Hz, C$_{12}$), 135.2 ($^2J_{CP}$ = 12.0 Hz, C$_{19}$), 132.1 ($^2J_{CP}$ = 5.2 Hz, C$_{13}$), 131.6 ($^4J_{CP}$ = 2.5 Hz, C$_{21}$), 130.1 ($^1J_{CP}$ = 59.7 Hz, C$_{18}$), 129.0 ($^3J_{CP}$ = 11.0 Hz, C$_{20}$), 124.1 (C$_9$), 122.7 ($^4J_{CP}$ = 4.0 Hz, C$_4$), 121.4 (C$_{10}$), 120.1 ($^4J_{CP}$ = 3.1 Hz, C$_6$), 34.8 (C$_{14}$), 30.8 (C$_{15}$).

^{31}P{^{1}H}-NMR (162 MHz, DCM-*d*$_2$): δ (ppm) = 23.9 ($^1J_{PPt}$ = 4089.2 Hz, P$_{17}$).

^{15}N-NMR (41 MHz, DCM-*d*$_2$): δ (ppm) = 242 (N$_2$).

^{195}Pt-NMR (86 MHz, DCM-*d*$_2$): δ (ppm) = - 3996 ($^1J_{PtP}$ = 4089.2 Hz, Pt$_{16}$).

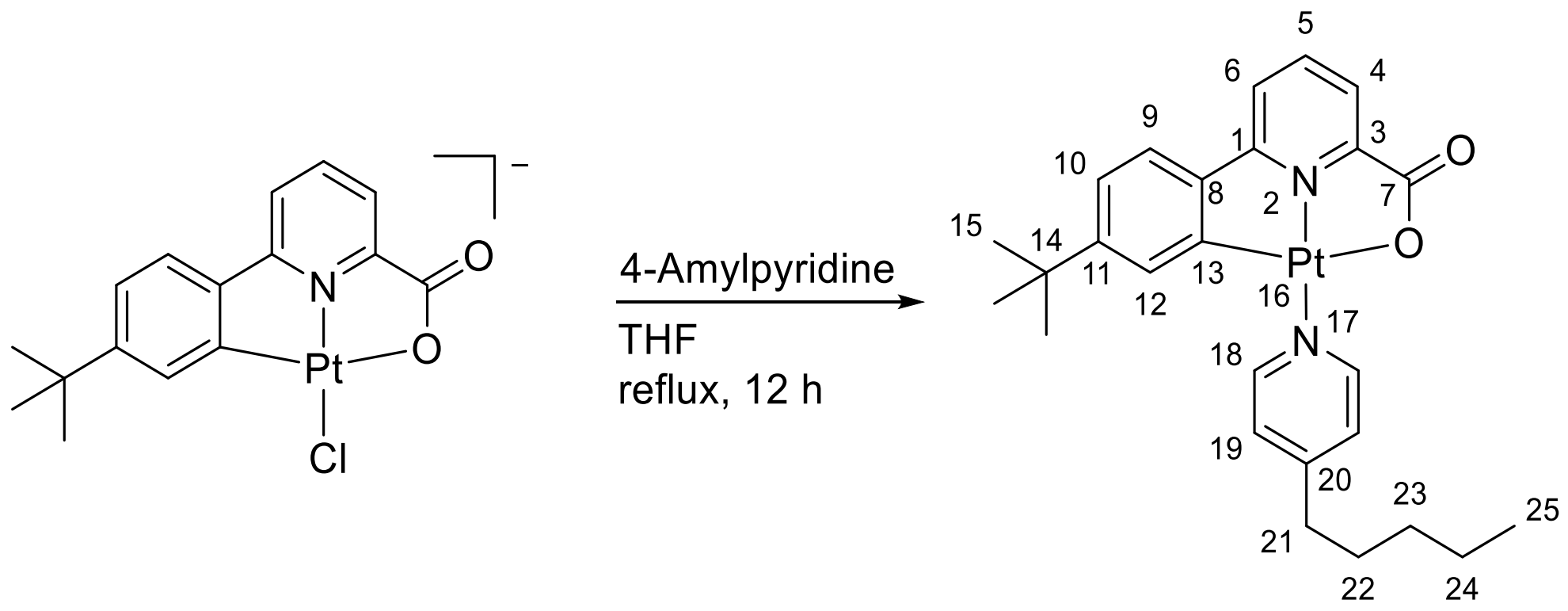

Pt(L$_{tBu}$)Cl **Pt(L$_{tBu}$)AmPy**

***Complex Pt(L$_{tBu}$)AmPy*.** Complex **Pt(L$_{tBu}$)Cl** (97 mg, 0.20 mmol, 1.0 eq.) and 4-amylpyridine (30 mg, 32 µL, 0.20 mmol, 1.0 eq.) were dissolved in THF (15 mL). The solution was purged with argon for 5 minutes and subsequently refluxed overnight. The reaction mixture was allowed to cool down to room temperature and the THF was removed under reduced pressure. The crude product was purified by flash column chromatography (SiO_2, DCM/methanol = 99:1) yielding **Pt(L$_{tBu}$)AmPy** (52 mg, 0.089 mmol, 44 %) as a yellow crystalline solid.

EM-MS-ESI (MeOH, $C_{26}H_{31}N_2O_2Pt$): calcd. [**Pt(L$_{tBu}$)AmPy**+H]$^+$ = 598.20303, found [**Pt(L$_{tBu}$)AmPy**+H]$^+$ = 598.20273; calcd. [**Pt(L$_{tBu}$)AmPy**+Na]$^+$ = 620.18497, found [**Pt(L$_{tBu}$)AmPy**+Na]$^+$ = 620.18444.

^{1}H-NMR (500 MHz, DCM-*d*$_2$): δ (ppm) = 8.83 (m, 2H, H$_{18}$), 7.88 (t, $^3J_{HH}$ = 7.9 Hz, 1H, H$_5$), 7.59 (m, 2H, H$_{4/6}$), 7.42 (d, $^3J_{HH}$ = 8.1 Hz, 1H, H$_9$), 7.35 (m, 2H, H$_{19}$) 7.17 (dd, $^3J_{HH}$ = 8.1 Hz, $^4J_{HH}$ = 1.9 Hz, 1H, H$_{10}$), 6.76 (d, $^4J_{HH}$ = 1.9 Hz, 1H, H$_{12}$), 2.74 (m, 2H, H$_{21}$), 1.72 (m, 2H, H$_{22}$), 1.37 (m, 4H, H$_{23/24}$), 1.25 (s, 9H, H$_{15}$), 0.92 (t, $^3J_{HH}$ = 7.1 Hz, 3H, H$_{25}$).

^{13}C{^{1}H}-NMR (101 MHz, DCM-*d*$_2$): δ (ppm) = 173.2 (C$_7$), 164.4 (C$_1$), 156.0 (C$_{20}$), 154.3 (C$_{11}$), 152.7 (C$_{18}$), 151.7 (C$_3$), 143.2 (C$_8$), 139.8 (C$_5$), 135.0 (C$_{13}$), 129.3 (d, $^2J_{CPt}$ = 24 Hz, C$_{12}$), 126.7 (C$_{19}$), 124.4 (C$_9$), 122.4 (C$_6$), 121.5 (C$_{10}$), 119.7 (C$_4$), 35.7 (C$_{21}$), 35.5 (C$_{14}$), 31.6 (C$_{23}$), 31.3 (C$_{15}$), 29.9 (C$_{22}$), 22.8 (C$_{24}$), 14.1 (C$_{25}$).

^{15}N-NMR (41 MHz, DCM-*d*$_2$): δ (ppm) = 216 (N$_2$), 214 (N$_{17}$).

^{195}Pt-NMR (86 MHz, DCM-*d*$_2$): δ (ppm) = - 3226 (Pt$_{16}$).

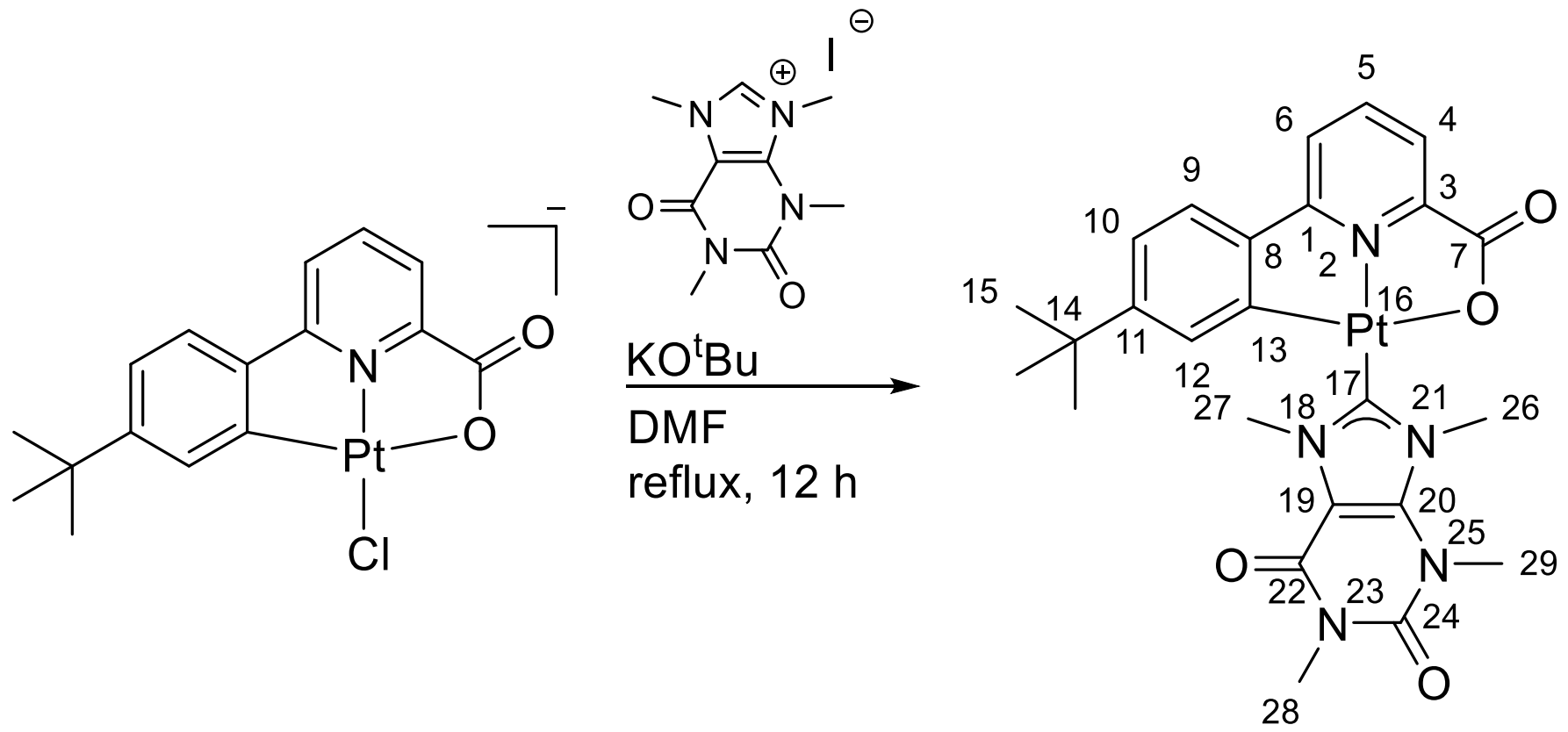

Pt(L$_{tBu}$)Cl **Pt(L$_{tBu}$)NHC**

Complex Pt(L$_{tBu}$)NHC. Complex **Pt(L$_{tBu}$)Cl** (80 mg, 0.16 mmol, 1.0 eq.), 9-methylcaffeine-ium iodide (81 mg, 0.24 mmol, 1.5 eq.) and KO^{t}Bu (28 mg, 0.24 mmol, 1.5 eq.) were dissolved in DMF (15 mL). The solution was purged with argon for 5 minutes and subsequently stirred for 12 hours at 70 °C. The reaction mixture was allowed to cool down to room temperature and the DMF was removed under reduced pressure. The crude product was purified by flash column chromatography (SiO_2, DCM/methanol = 99:1) yielding **Pt(L$_{tBu}$)NHC** (52 mg, 0.079 mmol, 49 %) as a yellow crystalline solid.

EM-MS-ESI (MeOH, $C_{25}H_{28}N_5O_4Pt$): calcd. [**Pt(L$_{tBu}$)NHC**+H]$^+$ = 657.17965, found [**Pt(L$_{tBu}$)NHC**+H]$^+$ = 657.17861; calcd. [**Pt(L$_{tBu}$)NHC**+Na]$^+$ = 679.16159, found [**Pt(L$_{tBu}$)NHC**+Na]$^+$ = 679.16039.

^{1}H-NMR (400 MHz, DCM-d_2): δ (ppm) = 7.98 (t, $^3J_{HH}$ = 7.9 Hz, 1H, H$_5$), 7.71 (m, 2H, H$_{4/6}$), 7.46 (d, $^3J_{HH}$ = 8.1 Hz, 1H, H$_9$), 7.15 (dd, $^3J_{HH}$ = 8.1 Hz, $^4J_{HH}$ = 2.0 Hz, 1H, H$_{10}$), 6.67 (d, $^4J_{HH}$ = 1.9 Hz, 1H, H$_{12}$), 4.37 (s, 3H, H$_{26}$), 4.19 (s, 3H, H$_{27}$), 3.83 (s, 3H, H$_{28}$), 3.38 (s, 3H, H$_{29}$).

^{13}C{^{1}H}-NMR (101 MHz, DCM-d_2): δ (ppm) = 174.7 (C$_7$), 173.1 (C$_{17}$), 162.6 (C$_1$), 154.9 (C$_{11}$), 153.6 (C$_{25}$), 151.2 (C$_{23}$), 149.5 (C$_3$), 140.6 (C$_{19}$), 140.5 (C$_5$), 133.5 (C$_{12}$), 133.0 (C$_{13}$), 124.5 (C$_9$), 122.6 (C$_4$), 121.5 (C$_{10}$), 120.1 (C$_6$), 110.0 (C$_{20}$), 39.2 (C$_{26}$), 37.8 (C$_{27}$), 34.8 (C$_{14}$), 32.3 (C$_{28}$), 31.2 (C$_{15}$), 28.7 (C$_{29}$).

^{15}N-NMR (41 MHz, DCM-d_2): δ (ppm) = 239 (N$_2$), 166 (N$_{21}$), 159 (N$_{18}$), 150 (N$_{25}$), 108 (N$_{23}$).

^{195}Pt-NMR (86 MHz, DCM-d_2): δ (ppm) = - 3658 (Pt$_{16}$).

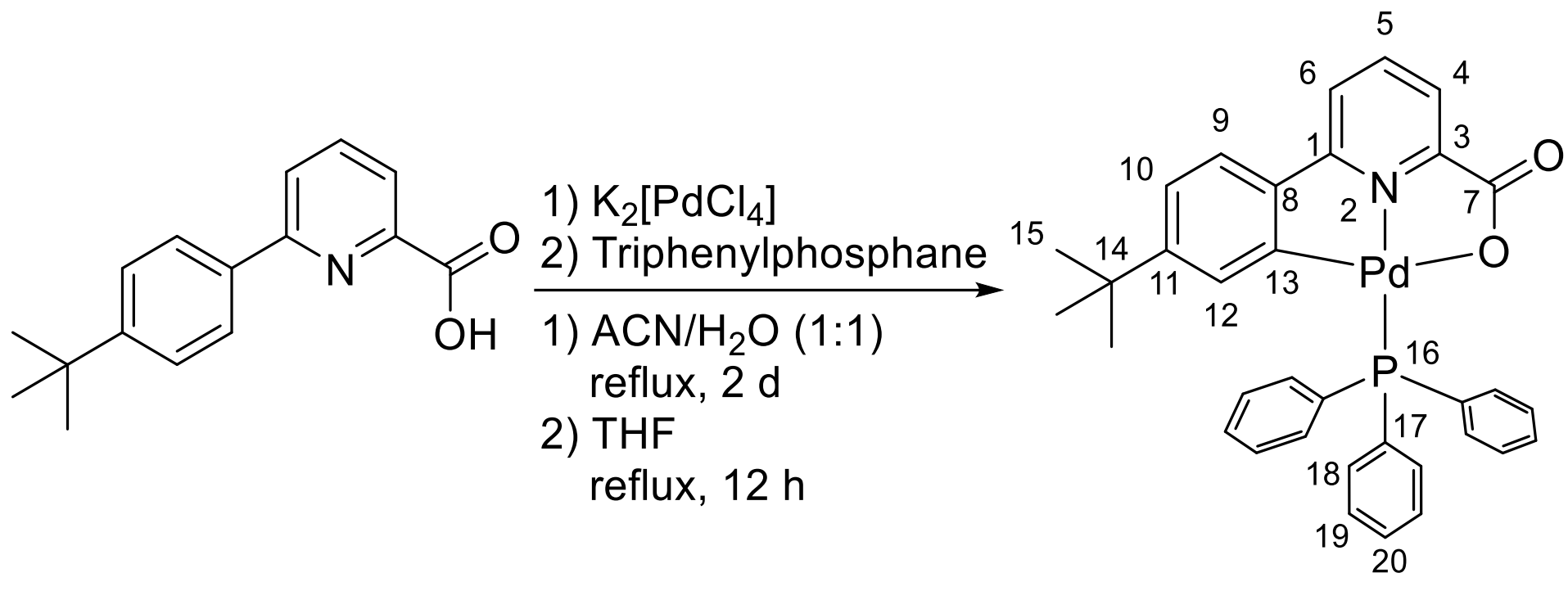

L_{tBu} **$Pd(L_{tBu})PPh_3$**

***Complex $Pd(L_{tBu})PPh_3$*.** $K_2[PdCl_4]$ (64 mg, 0.20 mmol, 1.0 eq.), **L_{tBu}** (50 mg, 0.20 mmol, 1.0 eq.) and a catalytic amount of TBACl were suspended in a mixture of acetonitrile/water (20 mL; 1:1 v/v). The solution was purged with argon for 10 minutes and subsequently stirred at 100 °C for two days. The reaction mixture was cooled to room temperature and the acetonitrile was removed under reduced pressure. The resulting precipitate was filtered off and washed with water. The crude product was used without further purification and dissolved in THF (15 mL) with triphenylphosphane (51 mg, 0.20 mmol, 1.0 eq.). The solution was purged with argon for 5 minutes and subsequently refluxed overnight. The reaction mixture was allowed to cool down to room temperature and the THF was removed under reduced pressure. The crude product was purified by flash column chromatography (SiO_2, DCM/methanol = 98:2), followed by re-crystallization from *n*-hexane in DCM, yielding **$Pd(L_{tBu})PPh_3$** (85 mg, 0.14 mmol, 70 %) as a yellow crystalline solid.

EM-MS-ESI (MeOH, $C_{34}H_{30}NO_2PPd$): calcd. [**$Pd(L_{tBu})PPh_3$**+H]$^+$ = 621.11428, found [**$Pd(L_{tBu})PPh_3$**+H]$^+$ = 621.11414; calcd. [**$Pd(L_{tBu})PPh_3$**+Na]$^+$ = 643.09620, found [**$Pd(L_{tBu})PPh_3$**+Na]$^+$ = 643.09608.

^{1}H-NMR (400 MHz, DCM-*d*$_2$): δ (ppm) = 7.95 (t, $^3J_{HH}$ = 7.9 Hz, 1H, H_5), 7.73 (m, 8H, $H_{4/6/18}$), 7.54 (m, 3H, H_{21}), 7.50 (m, 1H, H_9), 7.48 (m, 6H, H_{20}), 7.07 (dd, $^3J_{HH}$ = 8.1 Hz, $^4J_{HH}$ = 1.8 Hz, 1H, H_{10}), 6.66 (dd, $^4J_{HH}$ = 1.8 Hz, $^4J_{HP}$ = 4.7 Hz, 1H, H_{12}), 0.80 (s, 9H, H_{15}).

^{13}C{^{1}H}-NMR (101 MHz, DCM-*d*$_2$): δ (ppm) = 173.1 ($^3J_{CP}$ = 3.7 Hz, C_7), 162.1 ($^2J_{CPt}$ = 2.3 Hz, C_1), 153..9 ($^4J_{CP}$ = 3.6 Hz, C_{11}), 152.5 (C_3), 150.6 ($^2J_{CP}$ = 5.5 Hz, C_{13}), 145.9 (C_8), 140.9 (C_5), 138.5 ($^3J_{CP}$ = 9.7 Hz, C_{12}), 135.4 ($^2J_{CP}$ = 12.9 Hz, C_{18}), 131.8 ($^4J_{CP}$ = 2.5 Hz, C_{20}), 130.4 ($^1J_{CP}$ = 48.2 Hz, C_{17}), 129.3 ($^3J_{CP}$ = 10.6 Hz, C_{19}), 124.4 (C_9), 122.7 ($^4J_{CP}$ = 2.5 Hz, C_4), 122.3 (C_{10}), 119.9 ($^4J_{CP}$ = 2.7 Hz, C_6), 35.1 (C_{14}), 30.9 (C_{15}).

^{31}P{^{1}H}-NMR (162 MHz, DCM-*d*$_2$): δ (ppm) = 36.4 (P_{16}).

^{15}N-NMR (41 MHz, DCM-*d*$_2$): δ (ppm) = 251 (N_2).

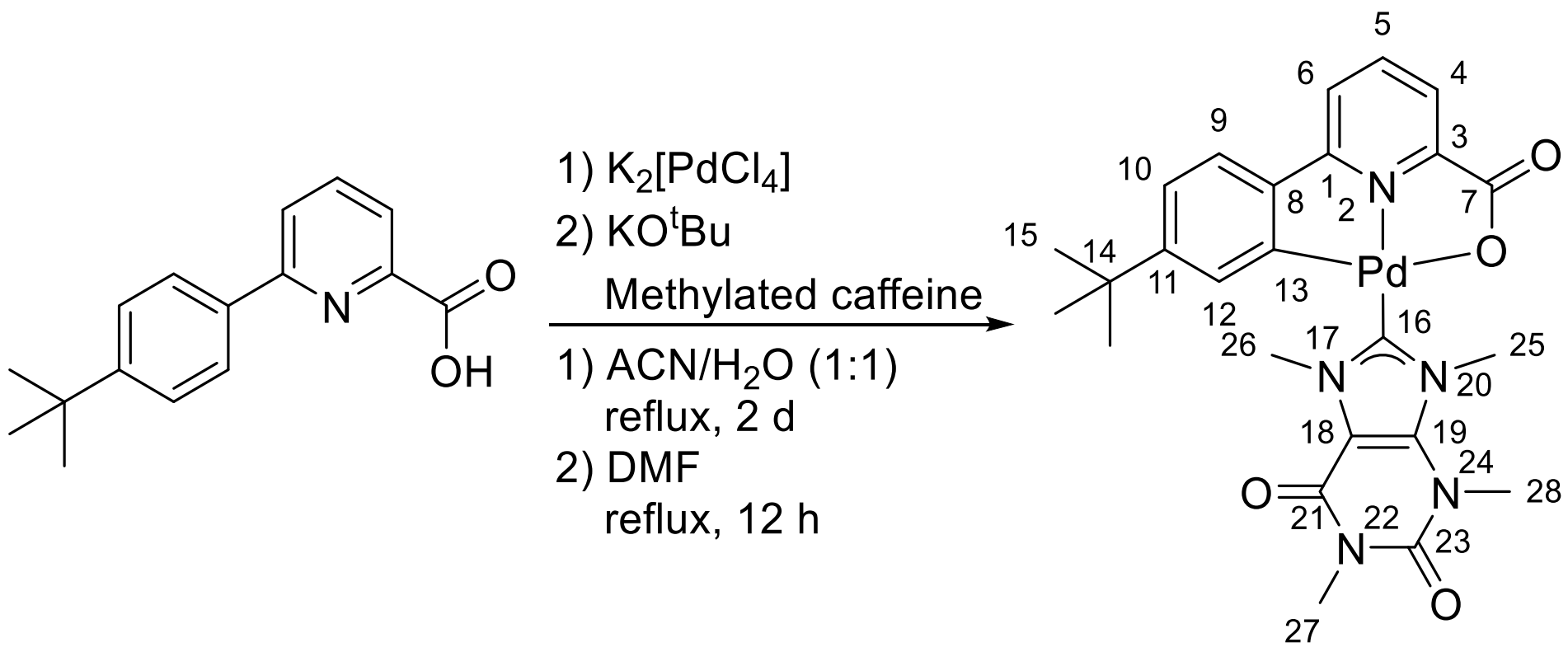

L_{tBu} **Pd(L_{tBu})NHC**

Complex Pd(L_{tBu})NHC. $K_2[PdCl_4]$ (64 mg, 0.20 mmol, 1.0 eq.), **L_{tBu}** (50 mg, 0.20 mmol, 1.0 eq.) and a catalytic amount of TBACl were suspended in a mixture of acetonitrile/water (20 mL; 1:1 v/v). The solution was purged with argon for 10 minutes and subsequently stirred at 100 °C for two days. The reaction mixture was cooled to room temperature and the acetonitrile was removed under reduced pressure. The resulting precipitate was filtered off and washed with water. The crude product was used without further purification, and 9-methylcaffeine-ium iodide (101 mg, 0.30 mmol, 1.5 eq.) and KO^{t}Bu (34 mg, 0.30 mmol, 1.5 eq.) were added to the solution, which was then purged with argon for 5 minutes and subsequently stirred for 12 hours at 70 °C. The reaction mixture was allowed to cool down to room temperature and the DMF was removed under reduced pressure. The crude product was purified by flash column chromatography (SiO_2, DCM/methanol = 99:1) yielding **Pd(L_{tBu})NHC** (52 mg, 0.079 mmol, 49 %) as a yellow crystalline solid.

EM-MS-ESI (MeOH, $C_{25}H_{27}N_5O_4Pd$): calcd. [**Pd(L_{tBu})NHC**+H]$^+$ = 568.11804, found [**Pd(L_{tBu})NHC**+H]$^+$ = 568.11702; calcd. [**Pd(L_{tBu})NHC**+Na]$^+$ = 590.09998, found [**Pd(L_{tBu})NHC**+Na]$^+$ = 590.09905.

^{1}H-NMR (500 MHz, DCM-d_2): δ (ppm) = 7.95 (t, $^3J_{HH}$ = 7.8 Hz, 1H, H_5), 7.68 (m, 2H, $H_{4/6}$), 7.49 (d, $^3J_{HH}$ = 8.1 Hz, 1H, H_9), 7.16 (dd, $^3J_{HH}$ = 8.1 Hz, $^4J_{HH}$ = 1.9 Hz, 1H, H_{10}), 6.55 (d, $^4J_{HH}$ = 1.9 Hz, 1H, H_{12}), 4.45 (s, 3H, H_{25}), 4.27 (s, 3H, H_{26}), 3.83 (s, 3H, H_{28}), 3.37 (s, 3H, H_{27}), 1.19 (s, 9H, H_{15}).

^{13}C{^{1}H}-NMR (121 MHz, DCM-d_2): δ (ppm) = 184.2 (C_{16}) 172.1 (C_7), 162.6 (C_1), 154.2 (C_{11}), 153.5 (C_{21}), 151.4 (C_3), 151.1 (C_{21}), 150.6 (C_{13}), 140.6 (C_{19}), 140.3 (C_5), 134.6 (C_{12}), 124.5 (C_9), 122.3 (C_{10}), 122.2 (C_4), 119.6 (C_6), 110.3 (C_{18}), 39.7 (C_{25}), 38.4 (C_{26}), 35.0 (C_{14}), 32.3 (C_{28}), 31.2 (C_{15}), 28.7 (C_{27}).

^{15}N-NMR (51 MHz, DCM-d_2): δ (ppm) = 247 (N_2), 169 (N_{17}), 160 (N_{20}), 150 (N_{22}), 108 (N_{24}).

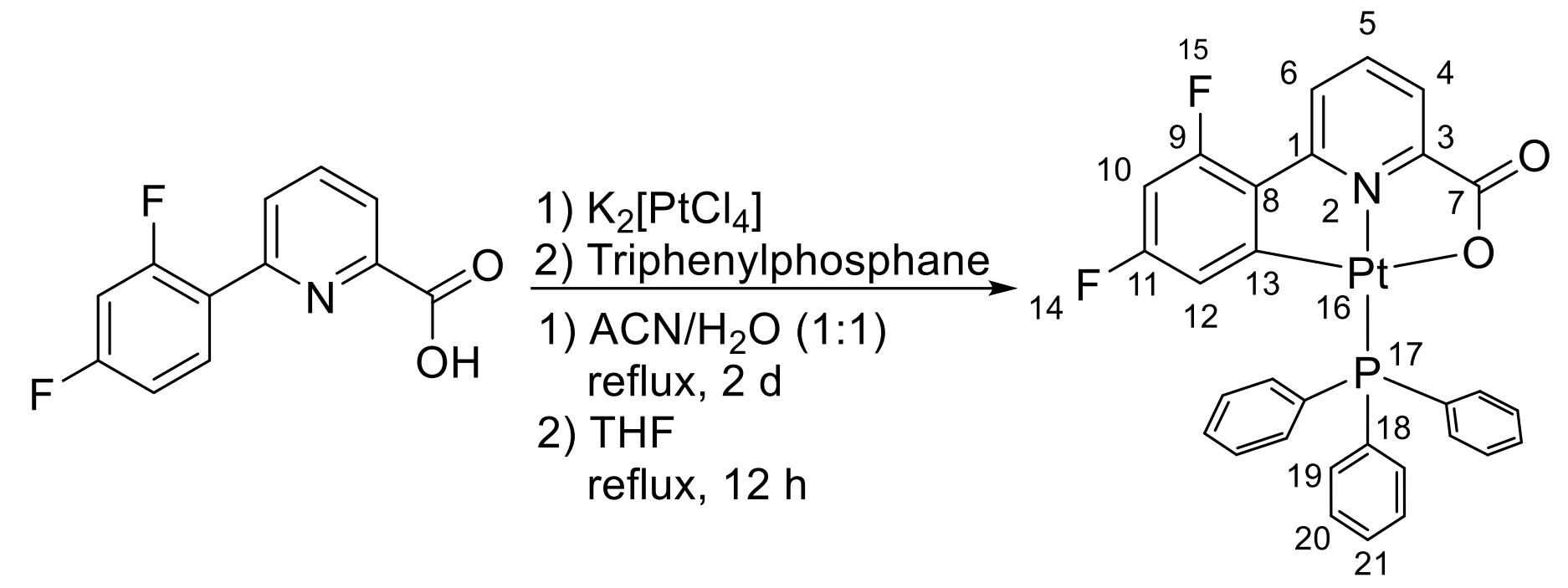

L_{DiF} **$Pt(L_{DiF})PPh_3$**

***Complex $Pt(L_{DiF})PPh_3$*.** $K_2[PtCl_4]$ (88 mg, 0.21 mmol, 1.0 eq.), **L_{DiF}** (50 mg, 0.21 mmol, 1.0 eq.) and a catalytic amount of TBACl were suspended in a mixture of acetonitrile/water (20 mL; 1:1 v/v). The solution was purged with argon for 10 minutes and subsequently stirred at 100 °C for two days. The reaction mixture was allowed to cool down to room temperature and the acetonitrile was removed under reduced pressure. The resulting precipitate was filtered off and washed with water. The crude product was used without further purification, and was dissolved in THF (15 mL) with triphenylphosphane (56 mg, 0.21 mmol, 1.0 eq.). The solution was purged with argon for 5 minutes and subsequently refluxed overnight. The reaction mixture was allowed to cool down to room temperature and the THF was removed under reduced pressure. The crude product was purified by flash column chromatography (SiO_2, DCM/methanol = 98:2), followed by re-crystallization from *n*-hexane in DCM. yielding **$Pt(L_{DiF})PPh_3$** (50 mg, 0.072 mmol, 34 %) as a yellow amorphous solid.

EM-MS-ESI (MeOH, $C_{30}H_{20}NO_2PFPt$): calcd. [**$Pt(L_{DiF})PPh_3$**+H]$^+$ = 691.09288, found [**$Pt(L_{DiF})PPh_3$**+H]$^+$ = 691.09288; calcd. [**$Pt(L_{DiF})PPh_3$**+Na]$^+$ = 713.07426, found [**$Pt(L_{DiF})PPh_3$**+Na]$^+$ = 713.07470.

^{1}H-NMR (500 MHz, DCM-d_2): δ (ppm) = 8.09 (m, 1H, H_6), 8.06 (t, $^3J_{HH}$ = 7.8 Hz, 1H, H_5), 7.78 (dt, $^3J_{HH}$ = 7.2 Hz, $^4J_{HH}$ = 1.5 Hz, 1H, H_7), 7.69 (m, 6H, H_{19}), 7.55 (m, 3H, H_{21}), 7.47 (m, 6H, H_{20}), 6.52 (ddd, $^3J_{HF}$ = 12.0 Hz, $^3J_{HF}$ = 8.8 Hz, $^4J_{HH}$ = 2.4 Hz, 1H, H_{10}), 5.93 (dt, $^3J_{HF}$ = 9.8 Hz, $^4J_{HH}$ = 2.4 Hz, 1H, H_{12}).

^{13}C{^{1}H}-NMR (126 MHz, DCM-d_2): δ (ppm) = 173.8 (d, $^3J_{CF}$ = 4.5 Hz, C_7), 163.5 (dd, $^1J_{CF}$ = 257.0 Hz, $^3J_{CF}$ = 13.8 Hz, $^4J_{CP}$ = 1.3 Hz, C_{11}), 160.5 (dd, $^1J_{CF}$ = 257.8 Hz, $^3J_{CF}$ = 13.1 Hz, C_9), 159.2 (d, $^3J_{CF}$ = 5.5 Hz, C_1), 150.4 (d, $^3J_{CP}$ = 2.2 Hz), 141.8 (C_5), 136.6 (t, $^2J_{CP}$ = 7.4 Hz, $^3J_{CF}$ = 7.4 Hz, C_{13}), 135.2 (d, $^2J_{CP}$ = 11.9 Hz, C_{19}), 131.9 (d, $^4J_{CP}$ = 2.7 Hz), 129.5 (d, $^1J_{CP}$ = 63.1 Hz, C_{18}), 129.1 (d, $^3J_{CP}$ = 11.1 Hz, C_{20}), 124.2 (dd, $^4J_{CF}$ = 17.3 Hz, $^4J_{CP}$ = 3.2 Hz, C_6), 123.2 (d, $^4J_{CP}$ = 2.9 Hz, C_4), 121.2 (ddd, $^2J_{CF}$ = 20.1 Hz, $^3J_{CP}$ = 2.9 Hz, $^4J_{CF}$ = 5.2 Hz, C_{12}), 100.34 (t, $^2J_{CP}$ = 2.9 Hz, C_{10}).

$^{19}F\{^1H\}$-NMR (471 MHz, DCM-d_2): δ (ppm) = - 106.5 ($^3J_{FF}$ = 6.9 Hz F_{15}), - 109.8 ($^4J_{FF}$ = 5.7 Hz, F_{14}).

$^{31}P\{^1H\}$-NMR (202 MHz, DCM-d_2): δ (ppm) = 22.1 ($^1J_{PPt}$ = 4008.1 Hz, P_{17}).

^{15}N-NMR (51 MHz, DCM-d_2): δ (ppm) = 241 (N_2).

^{195}Pt-NMR (107 MHz, DCM-d_2): δ (ppm) = - 3996 ($^1J_{PtP}$ = 4008.1 Hz, Pt_{16}).

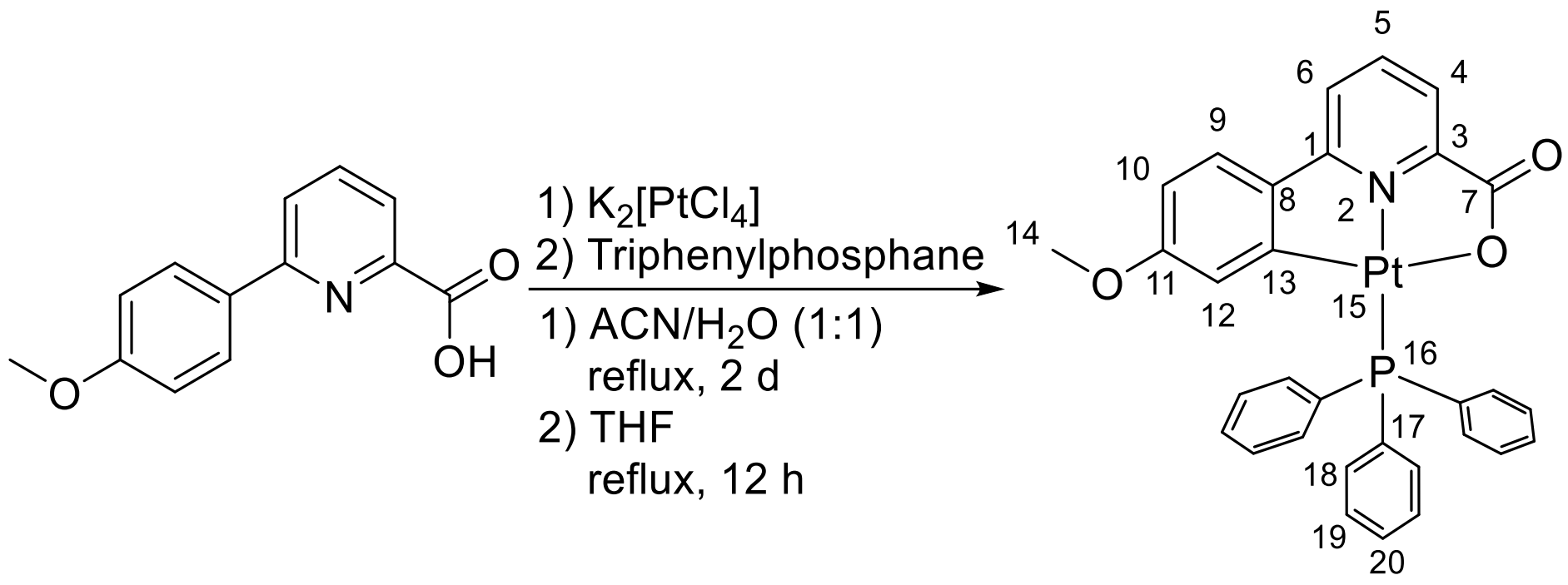

L$_{mOMe}$ **Pt(L$_{mOMe}$)PPh$_3$**

Complex Pt(L$_{mOMe}$)PPh$_3$. $K_2[PtCl_4]$ (91 mg, 0.22 mmol, 1.0 eq.), **L$_{mOMe}$** (50 mg, 0.22 mmol, 1.0 eq.) and a catalytic amount of TBACl were suspended in a mixture of acetonitrile/water (20 mL; 1:1 v/v). The solution was purged with argon for 10 minutes and subsequently stirred at 100 °C for two days. The reaction mixture was allowed to cool to room temperature and the acetonitrile was removed under reduced pressure. The resulting precipitate was filtered off and washed with water. The crude product was used without further purification and was dissolved in THF (15 mL) with triphenylphosphane (58 mg, 0.22 mmol, 1.0 eq.). The solution subsequently was purged with argon for 5 minutes and was refluxed overnight. The reaction mixture was allowed to cool down to room temperature and the THF was removed under reduced pressure. The crude product was purified by flash column chromatography (SiO_2, cyclohexane/acetone = 1:1) and re-crystallization from *n*-hexane in DCM yielded **Pt(L$_{mOMe}$)PPh$_3$** (42 mg, 0.067 mmol, 30 %) as a yellow amorphous solid.

EM-MS-ESI (MeOH, $C_{31}H_{24}NO_3PPt$): calcd. [**Pt(L$_{mOMe}$)PPh$_3$**+H]$^+$ = 685.12173, found [**Pt(L$_{mOMe}$)PPh$_3$**+H]$^+$ = 685.12248; calcd. [**Pt(L$_{mOMe}$)PPh$_3$**+Na]$^+$ = 707.10368, found [**Pt(L$_{mOMe}$)PPh$_3$**+Na]$^+$ = 707.10444.

^1H-NMR (400 MHz, DCM-d_2): δ (ppm) = 7.95 (t, $^3J_{HH}$ = 7.5 Hz, 1H, H_5), 7.68 (m, 8H, $H_{4/6/18}$), 7.53 (m, 3H, H_{20}), 7.45 (m, 6H, H_{19}), 6.54 (dd, $^3J_{HH}$ = 8.5 Hz, $^4J_{HH}$ = 2.5 Hz, 1H, H_{10}), 6.02 (dd, $^4J_{HH}$ = 2.5 Hz, $^4J_{HP}$ = 1.9 Hz, 1H, H_{12}), 3.10 (s, 3H, H_{14}).

$^{13}C\{^1H\}$-NMR (101 MHz, DCM-d_2): δ (ppm) = 174.0 (C_7), 162.4 (d, $^3J_{CP}$ = 1.5 Hz, C_1), 161.1 (d, $^4J_{CP}$ = 1.4 Hz, C_{11}), 150.3 (d, $^3J_{CP}$ = 1.5 Hz, C_3), 141.1 (C_5), 138.5 (d, $^3J_{CP}$ = 1.3 Hz, C_8), 135.2 (d, $^2J_{CP}$ = 12.0 Hz, C_{18}), 131.7 (d, $^4J_{CP}$ = 2.5 Hz, C_{20}), 130.0 (d, $^1J_{CP}$ =59.0 Hz, C_{17}), 129.0 (d, $^3J_{CP}$ = 11.0 Hz, C_{19}), 125.8 (C_{10}), 123.4 (d, $^2J_{CP}$ = 5.5 Hz, C_{12}), 121.9 (d, $^4J_{CP}$ = 3.0 Hz, C_6), 119.3 (d, $^4J_{CP}$ = 3.2 Hz, C_4), 111.6 (C_9), 55.1 (C_{14}).

$^{31}P\{^1H\}$-NMR (162 MHz, DCM-d_2): δ (ppm) = 23.4 ($^1J_{PPt}$ = 4069.2 Hz, P_{16}).

^{15}N-NMR (41 MHz, DCM-d_2): δ (ppm) = 242 (N_2).

^{195}Pt-NMR (86 MHz, DCM-d_2): δ (ppm) = - 3978 ($^1J_{PtP}$ = 4069.2 Hz, Pt_{15}).

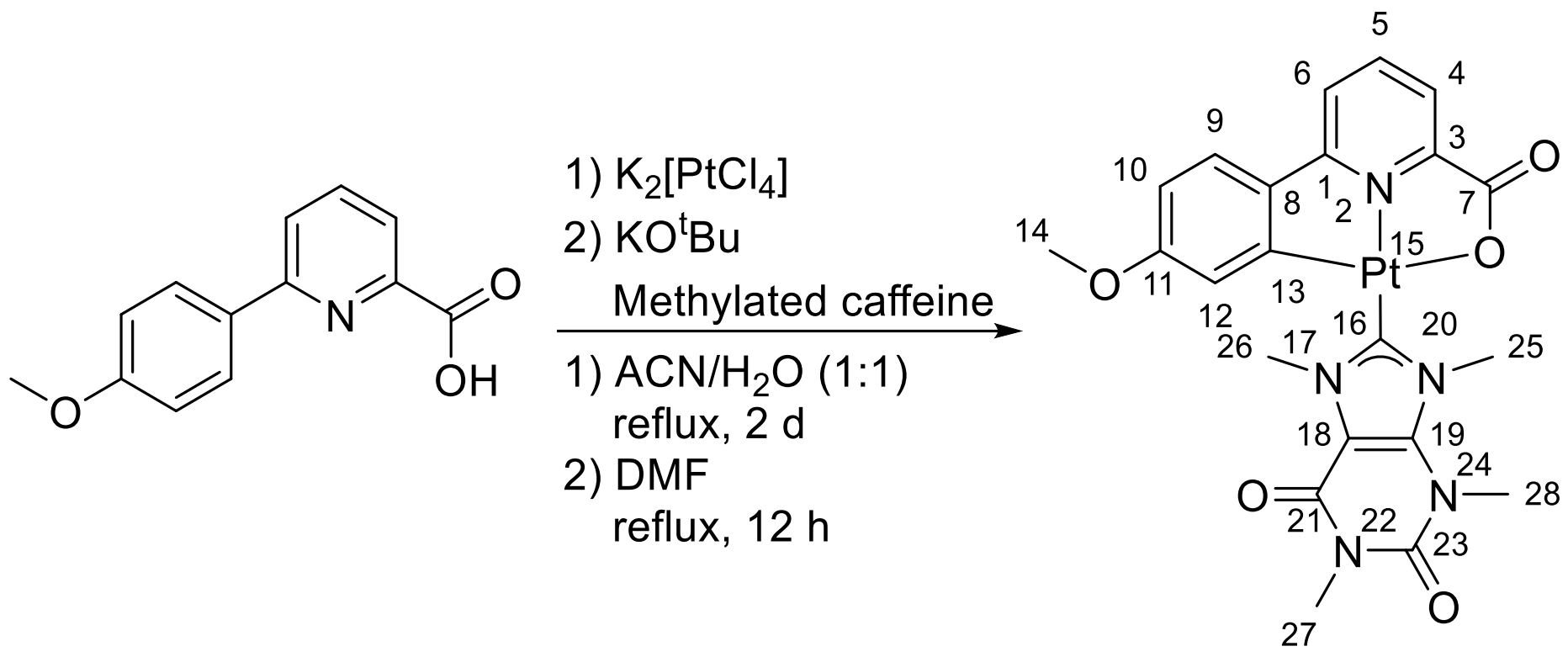

L_{mOMe} **Pt(L_{mOMe})NHC**

Complex Pt(L_{mOMe})NHC. $K_2[PtCl_4]$ (91 mg, 0.22 mmol, 1.0 eq.), **L_{mOMe}** (50 mg, 0.22 mmol, 1.0 eq.) and a catalytic amount of TBACl were suspended in acetonitrile/water (20 mL; 1:1 v/v). The solution was subsequently purged with argon for 10 minutes and was stirred at 100 °C for two days. The reaction mixture was allowed to cool down to room temperature and the acetonitrile was removed under reduced pressure. The resulting precipitate was filtered off and washed with water. The crude product was used without further purification and was dissolved in dry DMF (10 mL). 9-methylcaffeine-ium iodide (111 mg, 0.33 mmol, 1.5 eq.) and KO^{t}Bu (37 mg, 0.33 mmol, 1.5 eq.) were added to the solution, which was then purged with argon for 5 minutes and stirred for 12 hours at 70 °C. The reaction mixture was allowed to cool down to room temperature and the DMF was removed under reduced pressure. The crude product was purified by flash column chromatography (SiO_2, DCM/methanol = 99:1) yielding **Pt(L_{mOMe})NHC** (42 mg, 0.067 mmol, 30 %) as a yellow crystalline solid.

EM-MS-ESI (MeOH, $C_{22}H_{21}N_5O_5Pt$): calcd. [**Pt(L_{mOMe})NHC**+H]$^+$ = 631.12649, found [**Pt(L_{mOMe})NHC**+H]$^+$ = 631.12690; calcd. [**Pt(L_{mOMe})NHC**+Na]$^+$ = 653.10843, found [**Pt(L_{mOMe})NHC**+Na]$^+$ = 653.10871.

^{1}H-NMR (400 MHz, DCM-d_2): δ (ppm) = 7.94 (t, $^3J_{HH}$ = 7.9 Hz, 1H, H_5), 7.64 (m, 2H, $H_{4/6}$), 7.50 (d, 1H, $^3J_{HH}$ = 8.5 Hz, H_9), 6.64 (dd, $^3J_{HH}$ = 8.5 Hz, $^4J_{HH}$ = 2.6 Hz, 1H, H_{10}), 6.15 (d, $^3J_{HH}$ = 2.6 Hz, 1H, H_{12}), 4.36 (s, 3H, H_{25}), 4.19 (s, 3H, H_{26}), 3.81 (s, 3H, H_{28}), 3.38 (s, 3H, H_{27}), 3.72 (s, 3H, H_{14}).

^{13}C{^{1}H}-NMR (101 MHz, DCM-d_2): δ (ppm) = 174.7 (C_7), 173.1 (C_{16}), 162.4 (C_1), 161.9 (C_{11}), 153.7 (C_{21}), 151.1 (C_{23}), 149.5 (C_3), 140.5 (C_{19}), 140.3 (C_5), 135.4 (C_{13}), 126.2 (C_9), 123.3 (C_{12}), 121.9 (C_4), 119.6 (C_6), 110.1 (C_{18}), 107.9 (C_{10}), 55.5 (C_{14}), 39.2 (C_{25}), 37.7 (C_{26}), 32.5 (C_{28}), 28.7 (C_{27}).

^{15}N-NMR (41 MHz, DCM-d_2): δ (ppm) = 237 (N_2), 167 (N_{17}), 157 (N_{20}), 150 (N_{22}), 108 (N_{24}).

^{195}Pt-NMR (86 MHz, DCM-d_2): δ (ppm) = - 3637 (Pt_{15}).

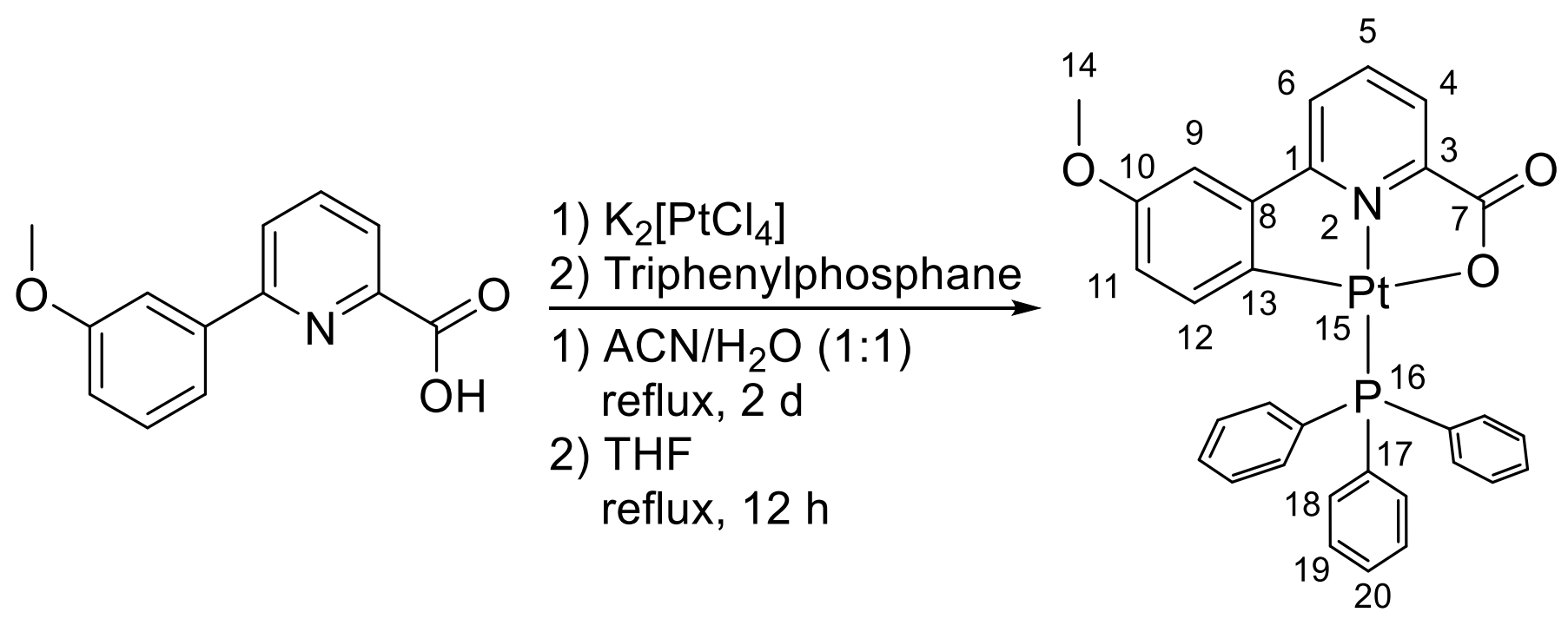

L_{pOMe} **$Pt(L_{pOMe})PPh_3$**

Complex $Pt(L_{pOMe})PPh_3$**.** $K_2[PtCl_4]$ (91 mg, 0.22 mmol, 1.0 eq.), **L_{pOMe}** (50 mg, 0.22 mmol, 1.0 eq.) and a catalytic amount of TBACl were suspended in a mixture of acetonitrile/water (20 mL; 1:1 v/v). The solution was purged with argon for 10 minutes and subsequently stirred at 100 °C for two days. The reaction mixture was allowed to cool down to room temperature and the acetonitrile was removed under reduced pressure. The resulting precipitate was filtered off and washed with water. The crude product was used without further purification and was dissolved in THF (15 mL) with triphenylphosphane (58 mg, 0.22 mmol, 1.0 eq.). The solution was purged with argon for 5 minutes and subsequently refluxed overnight. The reaction mixture was allowed to cool down to room temperature and the THF was removed under reduced pressure. The crude product was purified by flash column chromatography (SiO_2, cyclohexane/acetone = 1:1), followed by re-crystallization from *n*-hexane in DCM. yielding **$Pt(L_{pOMe})PPh_3$** (29 mg, 0.042 mmol, 19 %) as a yellow amorphous solid.

EM-MS-ESI (MeOH, $C_{31}H_{24}N_2O_2PPt$): calcd. [**$Pt(L_{pOMe})PPh_3$**+H]$^+$ = 685.12173, found [**$Pt(L_{pOMe})PPh_3$**+H]$^+$ = 685.12165.

^{1}H-NMR (400 MHz, DCM-*d*$_2$): δ (ppm) = 8.02 (t, $^{3}J_{HH}$ = 7.9 Hz, 1H, H_5), 7.77 (m, 2H, $H_{4/6}$), 7.68 (m, 6H, H_{18}), 7.48 (m, 3H, H_{20}), 7.42 (m, 6H, H_{19}), 7.11 (d, $^{4}J_{HH}$ = 2.6 Hz, 1H, H_9), 6.34 (m, 2H, $H_{11/12}$), 3.73 (s, 3H, H_{14}).

^{13}C{^{1}H}-NMR (101 MHz, DCM-*d*$_2$): δ (ppm) = 173.9 (C_7), 162.4 (d, $^{3}J_{CP}$ = 1.5 Hz, C_1), 157.8 (C_{10}), 150.8 (d, $^{3}J_{CP}$ = 1.7 Hz, C_3), 146.9 (d, $^{3}J_{CP}$ = 1.3 Hz, C_8), 141.4 (C_5), 139.5 (d, $^{3}J_{CP}$ = 4.6 Hz, C_{12}), 139.4 (d, $^{3}J_{CP}$ = 4.7 Hz, C_{18}), 135.2 (d, $^{2}J_{CP}$ = 12.0 Hz, C_{18}), 131.8 (d, $^{4}J_{CP}$ = 2.5 Hz, C_{20}), 130.2 (d, $^{1}J_{CP}$ =59.6 Hz, C_{17}), 129.1 (d, $^{3}J_{CP}$ = 11.2 Hz, C_{19}), 123.5 (d, $^{4}J_{CP}$ = 3.0 Hz, C_4), 122.1 (d, $^{2}J_{CP}$ = 7.4 Hz, C_{13}), 120.5 (d, $^{4}J_{CP}$ = 3.1 Hz, C_6), 116.3 (d, $^{4}J_{CP}$ = 1.7 Hz, C_{11}), 111.2 (C_9), 55.8 (C_{14}).

^{31}P{^{1}H}-NMR (162 MHz, DCM-*d*$_2$): δ (ppm) = 23.1 ($^{1}J_{PPt}$ = 4078.2 Hz, P_{16}).

^{15}N-NMR (41 MHz, DCM-*d*$_2$): δ (ppm) = 244 (N_2).

^{191}Pt-NMR (86 MHz, DCM-*d*$_2$): δ (ppm) = - 4009 ($^{1}J_{PtP}$ = 4078.2 Hz, Pt_{15}).

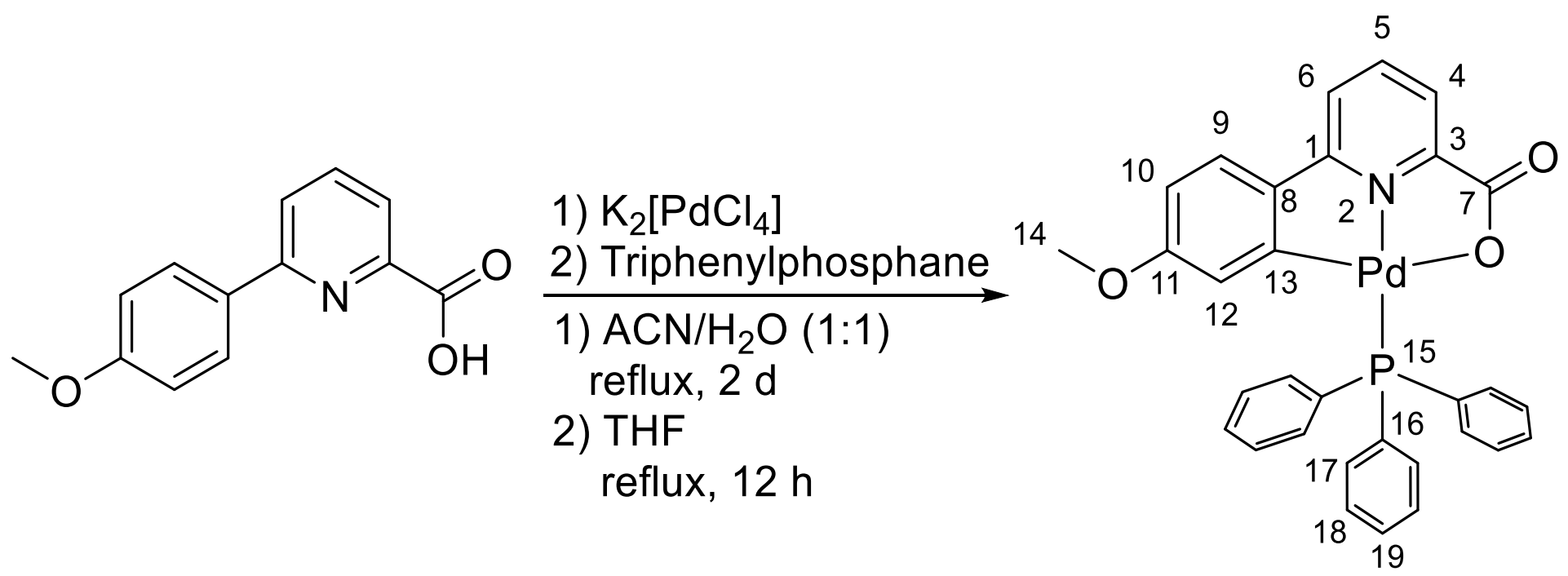

L$_{mOMe}$ **Pd(L$_{mOMe}$)PPh$_3$**

***Complex Pd(L$_{mOMe}$)PPh$_3$*.** $K_2[PdCl_4]$ (71 mg, 0.22 mmol, 1.0 eq.), **L$_{mOMe}$** (50 mg, 0.22 mmol, 1.0 eq.) and a catalytic amount of TBACl were suspended in a mixture of acetonitrile/water (20 mL; 1:1 v/v). The solution was purged with argon for 10 minutes and subsequently stirred at 100 °C for two days. The reaction mixture was allowed to cooldown to room temperature and the acetonitrile was removed under reduced pressure. The resulting precipitate was filtered off and washed with water. The crude product was used without further purification and was dissolved in THF (15 mL) with triphenylphosphane (58 mg, 0.22 mmol, 1.0 eq.). The solution was purged with argon for 5 minutes and was refluxed overnight. The reaction mixture was allowed to cool down to room temperature and the THF was removed under reduced pressure. The crude product was purified by flash column chromatography (SiO_2, cyclohexane/acetone = 1:1), followed by re-crystallization from *n*-hexane in DCM, yielding **Pd(L$_{mOMe}$)PPh$_3$** (38 mg, 0.063 mmol, 29 %) as a yellow amorphous solid.

EM-MS-ESI (MeOH, $C_{31}H_{24}NO_3PPd$): calcd. [**Pd(L$_{mOMe}$)PPh$_3$**+H]$^+$ = 596.06133, found [**Pd(L$_{mOMe}$)PPh$_3$**+H]$^+$ = 596.05976; calcd. [**Pd(L$_{mOMe}$)PPh$_3$**+Na]$^+$ = 617.04427, found [**Pd(L$_{mOMe}$)PPh$_3$**+Na]$^+$ = 617.04402.

^{1}H-NMR (500 MHz, DCM-d_2): δ (ppm) = 7.94 (t, $^3J_{HH}$ = 7.9 Hz, 1H, H_5), 7.65 (m, 8H, $H_{4/6/17}$), 7.51 (m, 3H, H_{19}), 7.43 (m, 7H, $H_{9/18}$), 6.55 (dd, $^3J_{HH}$ = 8.5 Hz, $^4J_{HH}$ = 2.5 Hz, 1H, H_{10}), 6.00 (dd, $^4J_{HH}$ = 2.5 Hz, 1H, H_{12}), 3.07 (s, 3H, H_{14}).

^{13}C{^{1}H}-NMR (126 MHz, DCM-d_2): δ (ppm) = 173.2 (C_7), 162.3 (d, $^3J_{CP}$ = 2.8 Hz, C_1), 160.3 (d, $^3J_{CP}$ = 4.6 Hz, C_{11}), 152.0 (d, $^2J_{CP}$ = 5.5 Hz, C_{13}), 151.3 (d, $^3J_{CP}$ = 0.9 Hz, C_3), 141.1 (C_5), 140.9 (C_8), 135.3 (d, $^2J_{CP}$ = 12.7 Hz, C_{17}), 131.9 (d, $^4J_{CP}$ = 2.3 Hz, C_{19}), 130.0 (d, $^1J_{CP}$ =48.6 Hz, C_{16}), 129.3 (d, $^3J_{CP}$ = 10.7 Hz, C_{18}), 125.9 (C_9), 124.2 (d, $^3J_{CP}$ = 10.1 Hz, C_{12}), 121.9 (d, $^4J_{CP}$ = 2.4 Hz, C_4), 120.1 (d, $^4J_{CP}$ = 2.2 Hz, C_6), 112.5 (C_{10}), 55.1 (C_{14}).

^{31}P{^{1}H}-NMR (162 MHz, DCM-d_2): δ (ppm) = 36.7 (P_{15}).

^{15}N-NMR (51 MHz, DCM-d_2): δ (ppm) = 249 (N_2).

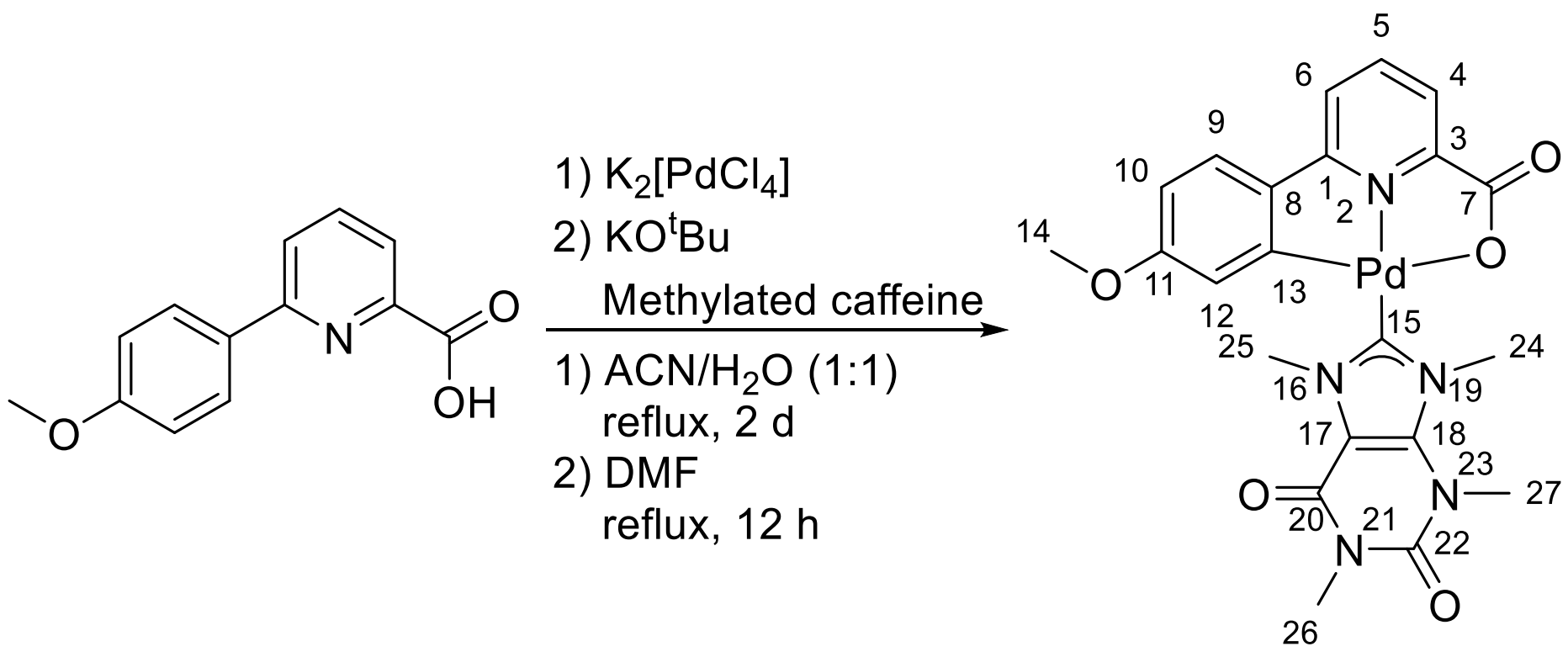

L$_{mOMe}$ **Pd(L$_{mOMe}$)NHC**

Complex Pd(L$_{mOMe}$)NHC. $K_2[PdCl_4]$ (71 mg, 0.22 mmol, 1.0 eq.), **L$_{mOMe}$** (50 mg, 0.22 mmol, 1.0 eq.) and a catalytic amount of TBACl were suspended in a mixture of acetonitrile/water (20 mL; 1:1 v/v). The solution was purged with argon for 10 minutes and subsequently stirred at 100 °C for two days. The reaction mixture was allowed to cool down to room temperature and the acetonitrile was removed under reduced pressure. The resulting precipitate was filtered off and washed with water. The crude product was used without further purification and was dissolved in dry DMF (10 mL). 9-methylcaffeine-ium iodide (111 mg, 0.22 mmol, 1.5 eq.) and KO^{t}Bu (37 mg, 0.26 mmol, 1.5 eq.) were added to the solution, which was then purged with argon for 5 minutes and stirred for 12 hours at 70 °C. The reaction mixture was allowed to cool down to room temperature and the DMF was removed under reduced pressure. The

crude product was purified by flash column chromatography (SiO_2, DCM/methanol = 99:1) yielding **Pd(L$_{mOMe}$)NHC** (45 mg, 0.083 mmol, 37 %) as a yellow crystalline solid.

EM-MS-ESI (MeOH, $C_{22}H_{21}N_5O_5Pd$): calcd. [**Pd(L$_{mOMe}$)NHC**+H]$^+$ = 542.06590, found [**Pd(L$_{mOMe}$)NHC**+H]$^+$ = 542.06459; calcd. [**Pd(L$_{mOMe}$)NHC**+Na]$^+$ = 564.04785, found [**Pd(L$_{mOMe}$)NHC**+Na]$^+$ = 564.04671.

^{1}H-NMR (400 MHz, DCM-d_2): δ (ppm) = 8.08 (t, $^3J_{HH}$ = 7.8 Hz, 1H, H_5), 7.98 (d, $^3J_{HH}$ = 8.1 Hz, 1H, H_6), , 7.74 (d, 1H, $^3J_{HH}$ = 8.6 Hz, H_9), 7.56 (d, 1H, $^3J_{HH}$ = 7.5 Hz, H_4), 6.71 (dd, $^3J_{HH}$ = 8.6 Hz, $^4J_{HH}$ = 2.5 Hz, 1H, H_{10}), 6.15 (d, $^3J_{HH}$ = 2.6 Hz, 1H, H_{12}), 4.36 (s, 3H, H_{25}), 4.19 (s, 3H, H_{26}), 3.81 (s, 3H, H_{28}), 3.38 (s, 3H, H_{27})¸ 3.72 (s, 3H, H_{14}).

^{13}C{^{1}H}-NMR (101 MHz, DCM-d_2): δ (ppm) = 181.0 (C_{16}), 174.7 (C_7), 160.9 (C_1), 160.0 (C_{11}), 152.9 (C_{20}), 151.7 (C_{13}), 150.4 (C_{22}), 150.3 (C_3), 140.7 (C_{18}), 140.6 (C_5), 139.6 (C_8), 125.9 (C_9), 123.7 (C_{12}), 120.9 (C_4), 119.5 (C_6), 109.2 (C_{17}), 108.8 (C_{10}), 54.9 (C_{14}), 38.8 (C_{24}), 37.7 (C_{25}), 31.4 (C_{27}), 28.1 (C_{26}).

^{15}N-NMR (41 MHz, DCM-d_2): δ (ppm) = 244 (N_2), 167 (N_{16}), 162 (N_{19}), 150 (N_{21}), 112 (N_{23}).

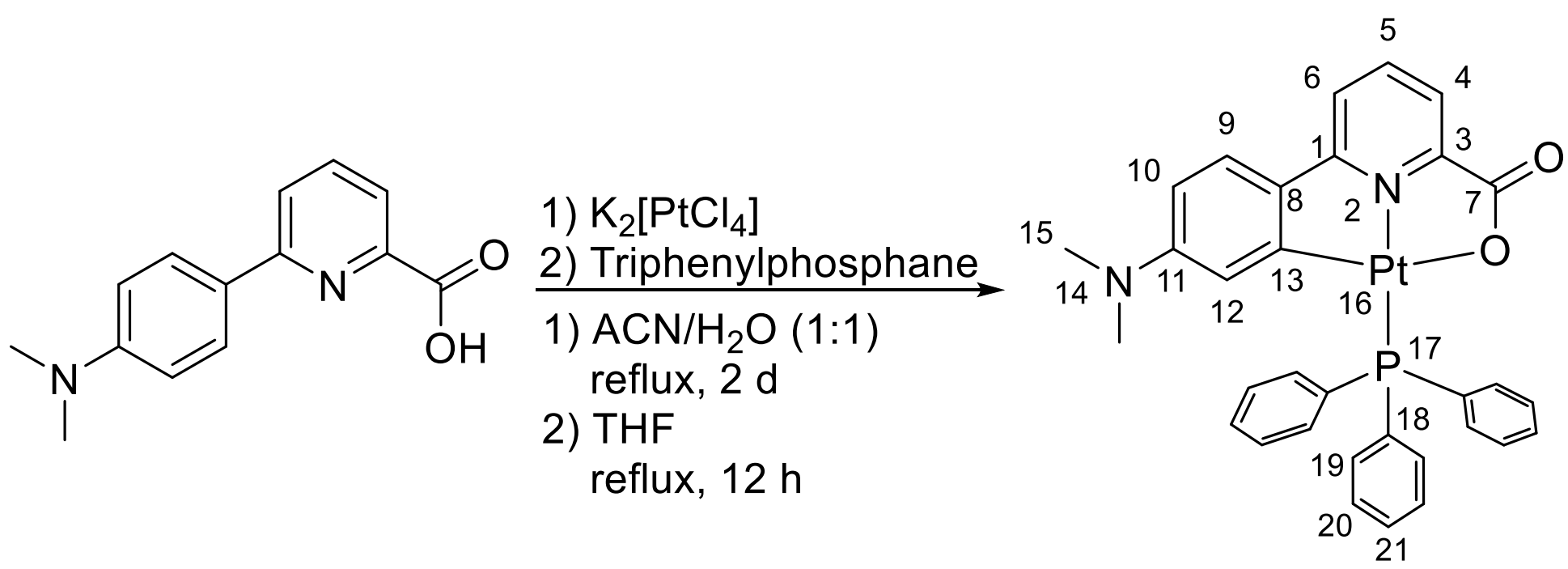

L$_{mDMA}$ **Pt(L$_{mDMA}$)PPh$_3$**

Complex Pt(L$_{mDMA}$)PPh$_3$. $K_2[PtCl_4]$ (86 mg, 0.21 mmol, 1.0 eq.), **L$_{mDMA}$** (50 mg, 0.21 mmol, 1.0 eq.) and a catalytic amount of TBACl were suspended in a mixture of acetonitrile/water (20 mL; 1:1 v/v). The solution was purged with argon for 10 minutes and subsequently stirred at 100 °C for two days. The reaction mixture was allowed to cool down to room temperature and the acetonitrile was removed under reduced pressure. The resulting precipitate was filtered off and washed with water. The crude product was used without further purification and was dissolved in THF (15 mL) with triphenylphosphane (54 mg, 0.21 mmol, 1.0 eq.). The solution was purged with argon for 5 minutes and subsequently was refluxed overnight. The reaction mixture was allowed to cool down to room temperature and the THF was removed under reduced pressure. The crude product was purified by flash column chromatography

(SiO_2, DCM/methanol = 98:2), followed by re-crystallization from *n*-hexane in DCM, yielding **Pt(L_{mDMA})PPh_3** (59 mg, 0.085 mmol, 40 %) as a red amorphous solid.

EM-MS-ESI (MeOH, $C_{32}H_{27}N_2O_2PPt$): calcd. [**Pt(L_{mDMA})PPh_3**+H]$^+$ = 698.15336, found [**Pt(L_{mDMA})PPh_3**+H]$^+$ = 698.15400; calcd. [**Pt(L_{mDMA})PPh_3**+Na]$^+$ = 720.13531, found [**Pt(L_{mDMA})PPh_3**+Na]$^+$ = 720.13588.

^{1}H-NMR (400 MHz, DCM-d_2): δ (ppm) = 7.83 (dd, $^3J_{HH}$ = 8.2 Hz, $^3J_{HH}$ = 8.2 Hz, 1H, H_5), 7.68 (m, 6H, H_{19}), 7.54 (m, 2H, $H_{4/6}$), 7.50 (m, 3H, H_{21}), 7.45 (m, 6H, H_{20}), 7.33 (d, $^3J_{HH}$ = 8.7 Hz, 1H, H_9), 6.32 (dd, $^3J_{HH}$ = 8.7 Hz, $^4J_{HH}$ = 2.6 Hz, 1H, H_{10}), 5.81 (t, $^4J_{HH}$ = 2.5 Hz, $^4J_{HP}$ = 2.5 Hz, 1H, H_{12}), 2.43 (s, 6H, H_{15}).

^{13}C{^{1}H}-NMR (101 MHz, DCM-d_2): δ (ppm) = 174.3 (C_7), 163.1 (d, $^3J_{CP}$ = 1.3 Hz, C_1), 151.7 (d, $^4J_{CP}$ = 1.4 Hz, C_{11}) 150.2 (d, $^3J_{CP}$ = 1.3 Hz, C_3), 140.6 (C_6), 135.2 (d, $^3J_{CP}$ = 11.7 Hz, C_{19}), 134.0 (d, $^2J_{CP}$ = 7.3 Hz, C_{13}), 132.9 (d, $^3J_{CP}$ = 1.4 Hz, C_8), 131.6 (d, $^2J_{CP}$ = 12.0 Hz, C_{19}), 131.6 (d, $^4J_{CP}$ = 2.6 Hz, C_{21}), 130.4 (d, $^1J_{CP}$ = 58.5 Hz, C_{18}), 129.0 (d, $^3J_{CP}$ = 10.9 Hz, C_{20}), 125.8 (C_9), 123.4 (d, $^3J_{CP}$ = 5.7 Hz, C_{12}), 120.2 (d, $^4J_{CP}$ = 3.4 Hz, C_6), 118.8 (d, $^4J_{CP}$ = 3.0 Hz, C_4), 107.3 (C_{10}), 110.3 (C_9), 39.9 (C_{15})..

^{31}P{^{1}H}-NMR (162 MHz, DCM-d_2): δ (ppm) = 24.1 ($^1J_{PPt}$ = 4088.7 Hz, P_{17}).

^{15}N-NMR (41 MHz, DCM-d_2): δ (ppm) = 239 (N_2), 53 (N_{14}).

^{195}Pt-NMR (86 MHz, DCM-d_2): δ (ppm) = - 3985 ($^1J_{PtP}$ = 4101.4 Hz, Pt_{16}).

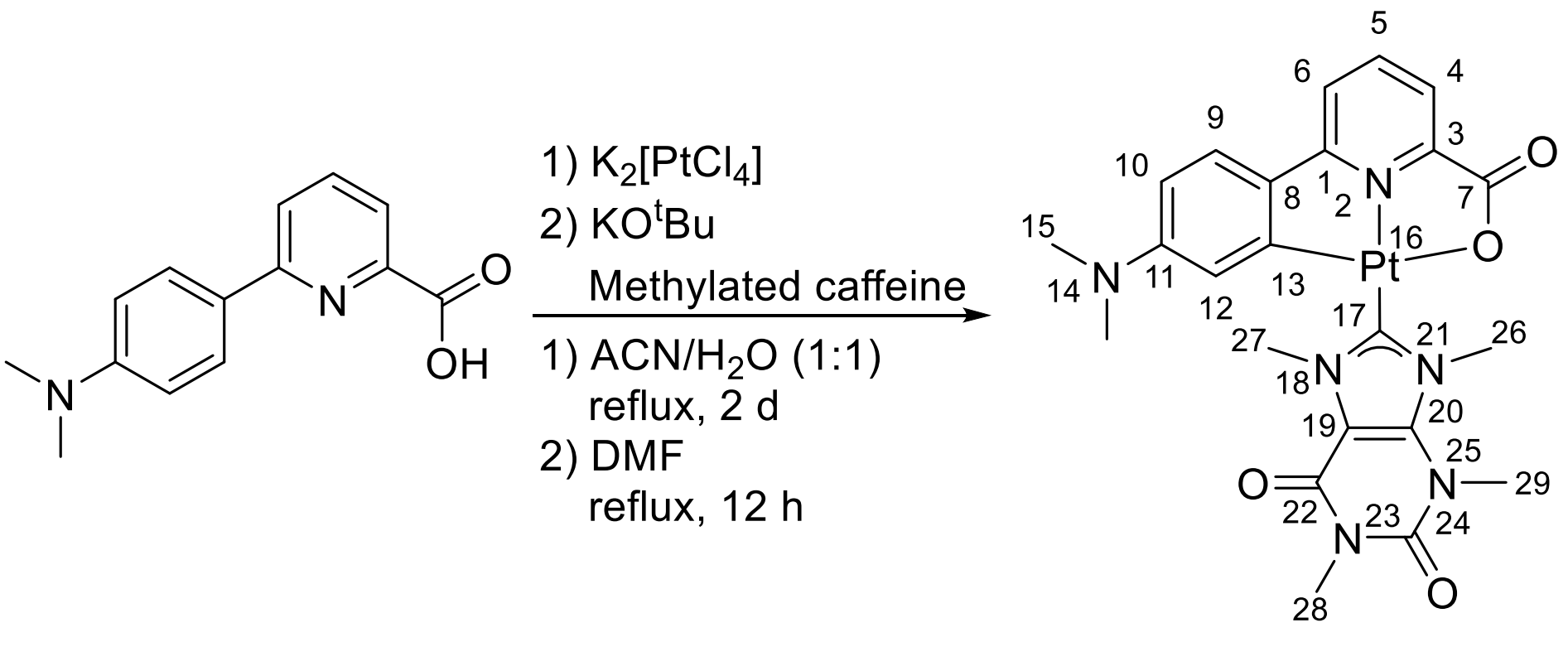

L_{mDMA} **Pt(L_{mDMA})NHC**

Complex Pt(L_{mDMA})NHC. $K_2[PtCl_4]$ (91 mg, 0.22 mmol, 1.0 eq.), **L_{mDMA}** (50 mg, 0.22 mmol, 1.0 eq.) and a catalytic amount of TBACl were suspended in a mixture of acetonitrile/water (20 mL; 1:1 v/v). The solution was purged with argon for 10 minutes and subsequently stirred at 100 °C for two days. The reaction mixture was allowed to cool down to room temperature and the acetonitrile was removed under reduced pressure. The resulting precipitate was

filtered off and washed with water. The crude product was used without further purification and was dissolved in DMF (10 mL). 9-Methylcaffeine-ium iodide (111 mg, 0.33 mmol, 1.5 eq.) and KO^tBu (37 mg, 0.33 mmol, 1.5 eq.) were added to the solution, which was then purged with argon for 5 minutes and stirred for 12 hours at 70 °C. The reaction mixture was allowed to cool down to room temperature and the DMF was removed under reduced pressure. The crude product was purified by flash column chromatography (SiO_2, DCM/methanol = 99:1) yielding **Pt(L_{mDMA})NHC** (59 mg, 0.085 mmol, 40 %) as a red crystalline solid.

EM-MS-ESI (MeOH, $C_{32}H_{27}N_2O_2PPt$): calcd. [**Pt(L_{mDMA})NHC**+Na]$^+$ = 666.14006, found [**Pt(L_{mDMA})NHC**+Na]$^+$ = 666.14053.

^{1}H-NMR (500 MHz, DCM-d_2): δ (ppm) = 7.84 (t, $^3J_{HH}$ = 7.8 Hz, 1H, H_5), 7.53 (m, 2H, $H_{4/6}$), 7.36 (d, $^3J_{HH}$ = 8.6 Hz, 1H, H_9), 6.43 (dd, $^3J_{HH}$ = 8.7 Hz, $^4J_{HH}$ = 2.6 Hz, 1H, H_{10}), 5.94 (d, $^4J_{HH}$ = 2.6 Hz, 1H, H_{12}), 4.39 (s, 3H, H_{26}), 4.22 (s, 3H, H_{27}), 3.82 (s, 3H, H_{29}), 3.39 (s, 3H, H_{28}), 2.89 (s, 6H, H_{15}).

^{13}C{^{1}H}-NMR (126 MHz, DCM-d_2): δ (ppm) = 175.1 (C_7), 174.5 (C_{17}), 163.3 (C_1), 153.8 (C_{22}), 152.7 (C_{11}), 151.4 (C_{24}), 149.5 (C_3), 140.7 (C_{20}), 140.0 (C_5), 135.3 (C_{13}), 132.9 (C_8) 126.2 (C_9), 120.4 (C_4), 119.7 (C_{12}), 118.9 (C_6), 110.2 (C_{19}), 107.5 (C_{10}), 40.5 (C_{15}), 39.5 (C_{27}), 38.2 (C_{26}), 32.5 (C_{29}), 28.9 (C_{28}).

^{15}N-NMR (51 MHz, DCM-d_2): δ (ppm) = 234 (N_2), 166 (N_{17}), 159 (N_{20}), 150 (N_{22}), 108 (N_{24}), 53 (N_{14}).

^{195}Pt-NMR (107 MHz, DCM-d_2): δ (ppm) = - 3643 (Pt_{16}).

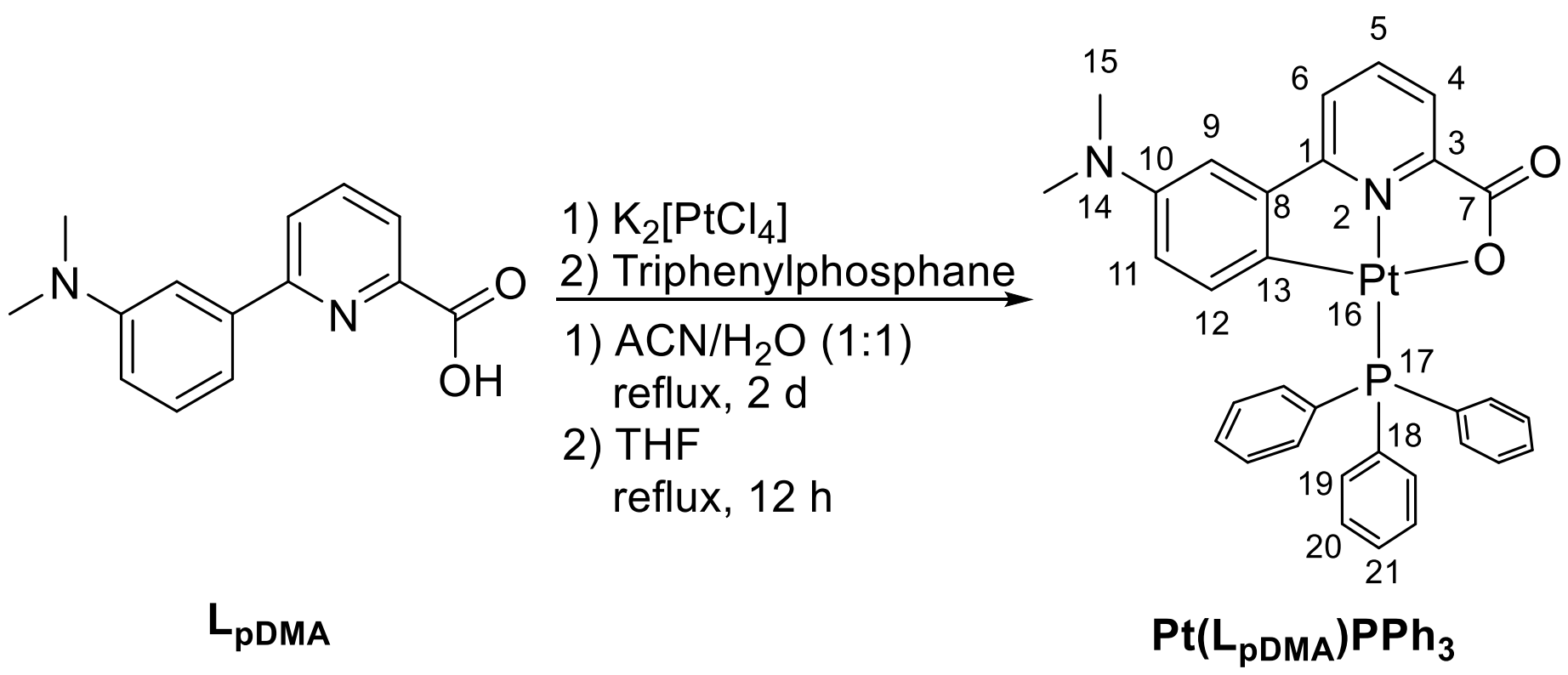

***Complex Pt(L_{pDMA})PPh$_3$*.** $K_2[PtCl_4]$ (86 mg, 0.21 mmol, 1.0 eq.), **L_{pDMA}** (50 mg, 0.21 mmol, 1.0 eq.) and a catalytic amount of TBACl were suspended in a mixture of acetonitrile/water (20 mL; 1:1 v/v). The solution was purged with argon for 10 minutes and subsequently stirred at 100 °C for two days. The reaction mixture was cooled to room temperature and the

acetonitrile was removed under reduced pressure. The resulting precipitate was filtered off and washed with water. The crude product was used without further purification and dissolved in THF (15 mL) with triphenylphosphane (54 mg, 0.21 mmol, 1.0 eq.). The solution was purged with argon for 5 minutes and was refluxed overnight. The reaction mixture was allowed to cool down to room temperature and the THF was removed under reduced pressure. The crude product was purified by flash column chromatography (SiO_2, DCM/methanol = 98:2), followed by re-crystallization from n-Hexane in DCM, yielding **Pt(L_{pDMA})PPh_3** (47 mg, 0.067 mmol, 32 %) as a yellow amorphous solid.

EM-MS-ESI (MeOH, $C_{32}H_{27}N_2O_2PPt$): calcd. [**Pt(L_{pDMA})PPh_3**+H]$^+$ = 698.15336, found [**Pt(L_{pDMA})PPh_3**+H]$^+$ = 698.15323.

^{1}H-NMR (500 MHz, DCM-d_2): δ (ppm) = 8.06 (t, $^3J_{HH}$ = 7.9 Hz, 1H, H_5), 7.87 (d, $^3J_{HH}$ = 8.1 Hz, 1H, H_6), 7.74 (d, $^3J_{HH}$ = 7.6 Hz, 1H, H_4), 7.69 (m, 6H, H_{19}), 7.53 (m, 3H, H_{21}), 7.46 (m, 6H, H_{20}), 6.99 (d, $^4J_{HH}$ = 2.8 Hz, 1H, H_9), 6.27 (d, $^3J_{HH}$ = 8.6 Hz, 1H, H_{12}), 6.21 (dd, $^3J_{HH}$ = 8.7 Hz, $^3J_{HH}$ = 2.9 Hz, 1H, H_{11}), 2.89 (s, 6H, H_{15}).

^{13}C{^{1}H}-NMR (126 MHz, DCM-d_2): δ (ppm) = 175.3 (d, $^3J_{CP}$ = 1.4 Hz, C_7), 163.2 (C_1), 150.1 (C_3), 148.9 (C_{10}), 146.2 (C_8), 141.4 (C_5), 139.2 (d, $^3J_{CP}$ = 4.7 Hz, C_{12}), 135.2 (d, $^2J_{CP}$ = 12.0 Hz, C_{19}), 131.6 (d, $^4J_{CP}$ = 2.6 Hz, C_{21}), 130.2 (d, $^2J_{CP}$ = 59.6 Hz, C_{18}), 129.0 (d, $^3J_{CP}$ = 11.0 Hz, C_{20}), 123.2 (d, $^4J_{CP}$ = 3.0 Hz, C_4), 120.8 (d, $^4J_{CP}$ = 3.1 Hz, C_6), 116.40 (d, $^2J_{CP}$ = 7.2 Hz, C_{13}), 116.0 (C_{11}), 110.3 (C_9), 40.9 (C_{15}).

^{31}P{^{1}H}-NMR (202 MHz, DCM-d_2): δ (ppm) = 23.9 ($^1J_{PPt}$ = 4101.4 Hz, P_{17}).

^{15}N-NMR (51 MHz, DCM-d_2): δ (ppm) = 247 (N_2).

^{191}Pt-NMR (107 MHz, DCM-d_2): δ (ppm) = - 4032 ($^1J_{PtP}$ = 4101.4 Hz, Pt_{16}).

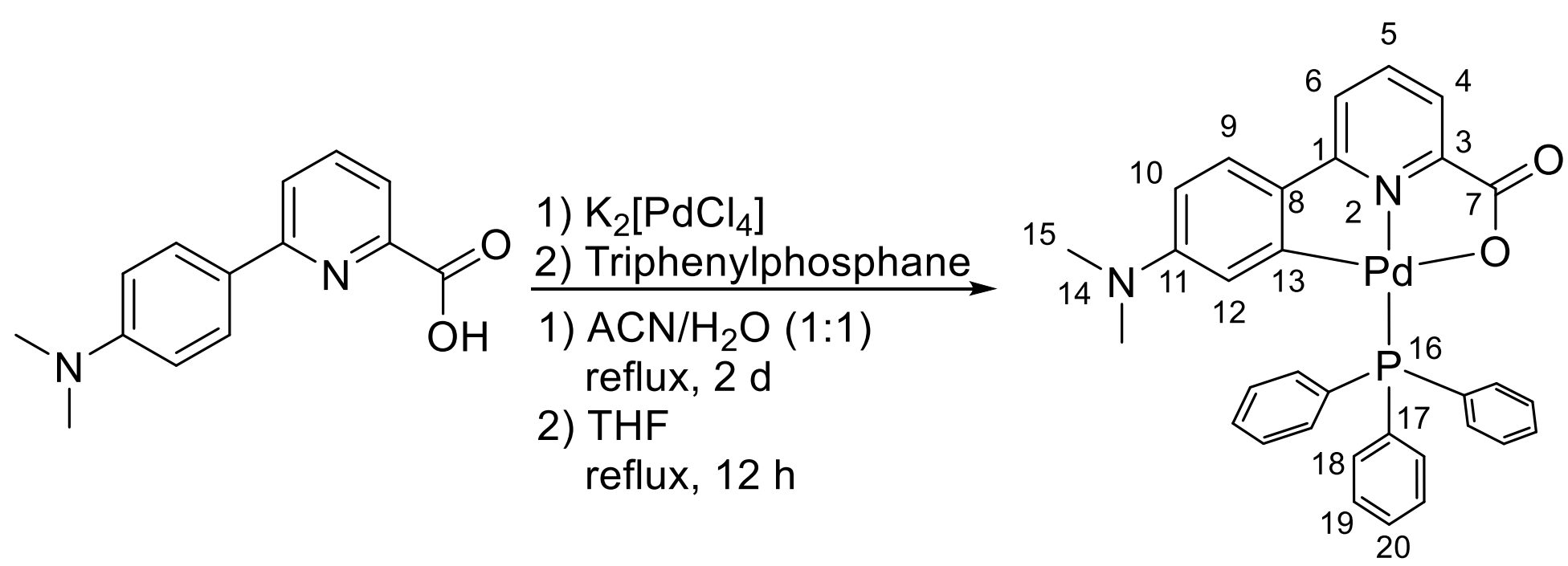

L_{mDMA} **Pd(L_{mDMA})PPh_3**

***Complex Pd(L_{mDMA})PPh_3*.** $K_2[PdCl_4]$ (69 mg, 0.21 mmol, 1.0 eq.), **L_{mDMA}** (50 mg, 0.21 mmol, 1.0 eq.) and a catalytic amount of TBACl were suspended in a mixture of acetonitrile/water

(20 mL; 1:1 v/v). The solution was purged with argon for 10 minutes and subsequently stirred at 100 °C for two days. The reaction mixture was allowed to cool down to room temperature and the acetonitrile was removed under reduced pressure. The resulting precipitate was filtered off and washed with water. The crude product was used without further purification and was dissolved in THF (15 mL) with triphenylphosphane (55 mg, 0.21 mmol, 1.0 eq.). The solution was purged with argon for 5 minutes and was refluxed overnight. The reaction mixture was allowed to cool down to room temperature and the THF was removed under reduced pressure. The crude product was purified by flash column chromatography (SiO_2, DCM/methanol = 98:2), followed by re-crystallization from *n*-hexane in DCM, yielding **Pd(L_{mDMA})PPh_3** (61 mg, 0.10 mmol, 48 %) as a yellow amorphous solid.

EM-MS-ESI (MeOH, $C_3H_{27}N_2O_2PPd$): calcd. [**Pd(L_{mDMA})PPh_3**+H]$^+$ = 609.09299, found [**Pd(L_{mDMA})PPh_3**+H]$^+$ = 609.09127; calcd. [**Pd(L_{mDMA})PPh_3**+Na]$^+$ = 631.07493, found [**Pd(L_{mDMA})PPh_3**+Na]$^+$ = 631.07350.

^{1}H-NMR (500 MHz, DCM-d_2): δ (ppm) = 7.81 (t, $^3J_{HH}$ = 7.8 Hz, 1H, H_5), 7.68 (m, 6H, H_{17}), 7.51 (m, 5H, $H_{4/6/20}$), 7.45 (m, 6H, H_{19}), 7.35 (d, $^3J_{HH}$ = 8.6 Hz, H_9), 6.31 (dd, $^3J_{HH}$ = 8.6 Hz, $^4J_{HH}$ = 2.5 Hz, 1H, H_{10}), 5.81 (dd, $^4J_{HH}$ = 2.5 Hz, $^4J_{HP}$ = 4.9 Hz, 1H, H_{12}), 2.39 (s, 3H, H_{14}).

^{13}C{^{1}H}-NMR (126 MHz, DCM-d_2): δ (ppm) = 172.9 (d, $^3J_{CP}$ = 3.6 Hz, C_7), 162.9 (d, $^3J_{CP}$ = 2.5 Hz, C_1), 152.3 (d, $^2J_{CP}$ = 5.5 Hz, C_{13}), 152.2 (d, $^3J_{CP}$ = 0.8 Hz, C_3), 151.0 (d, $^4J_{CP}$ = 4.1 Hz, C_{11}), 140.3 (C_5), 135.5 (C_8), 135.5 (d, $^2J_{CP}$ = 12.8 Hz, C_{18}), 131.8 (d, $^4J_{CP}$ = 2.5 Hz, C_{20}), 130.7 (d, $^1J_{CP}$ =47.5 Hz, C_{17}), 129.3 (d, $^3J_{CP}$ = 10.8 Hz, C_{19}), 125.7 (C_9), 124.0 (d, $^3J_{CP}$ = 10.8 Hz, C_{12}), 120.4 (d, $^4J_{CP}$ = 2.6 Hz, C_4), 118.7 (d, $^4J_{CP}$ = 2.8 Hz, C_6), 108.5 (C_{10}), 40.1 (C_{14}).

^{31}P{^{1}H}-NMR (202 MHz, DCM-d_2): δ (ppm) = 36.1 (P_{16}).

^{15}N-NMR (51 MHz, DCM-d_2): δ (ppm) = 250 (N_2), 54 (N_2).

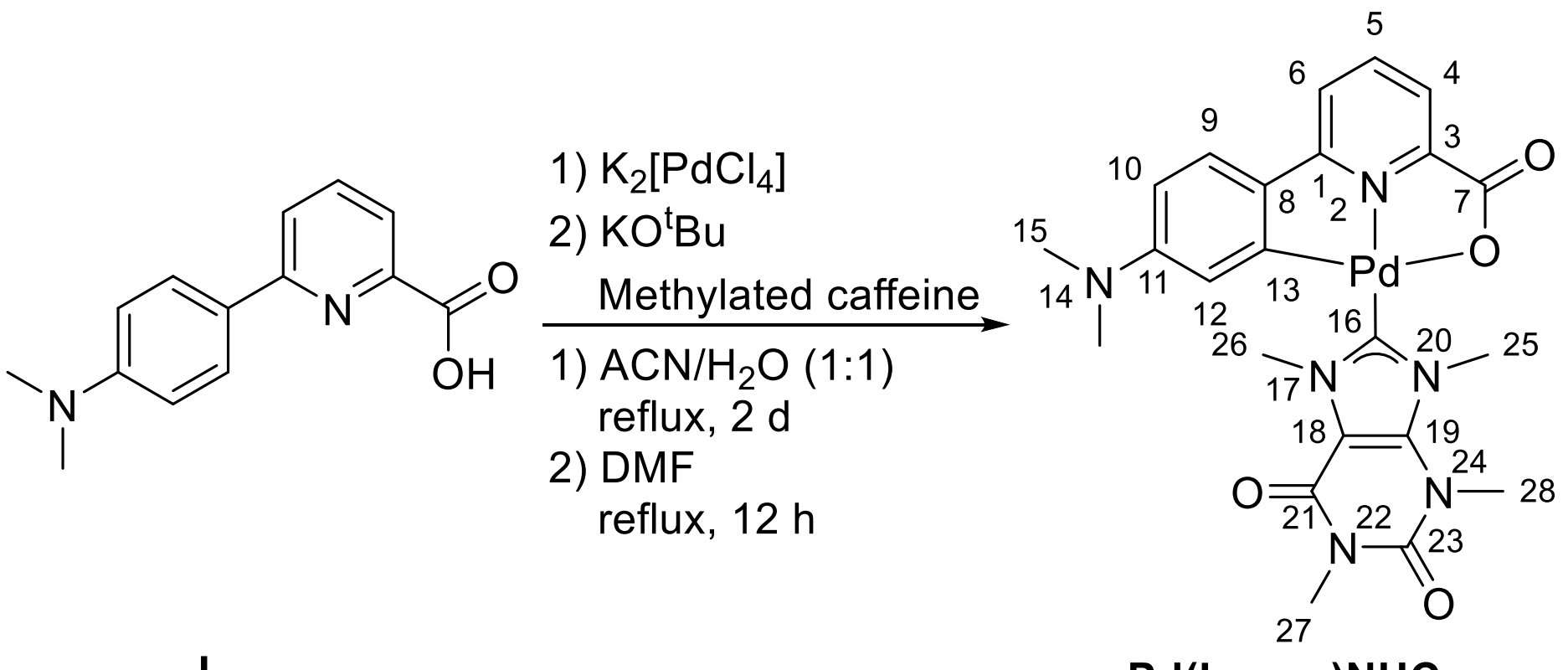

Complex Pd(L$_{mDMA}$)NHC. $K_2[PdCl_4]$ (71 mg, 0.22 mmol, 1.0 eq.), **L$_{mDMA}$** (50 mg, 0.22 mmol, 1.0 eq.) and a catalytic amount of TBACl were suspended in a mixture of acetonitrile/water (20 mL; 1:1 v/v). The solution was purged with argon for 10 minutes and subsequently stirred at 100 °C for two days. The reaction mixture was allowed to cool down to room temperature and the acetonitrile was removed under reduced pressure. The resulting precipitate was filtered off and washed with water. The crude product was used without further purification and was dissolved in DMF (10 mL). 9-Methylcaffeine-ium iodide (111 mg, 0.33 mmol, 1.5 eq.) and KO^{t}Bu (37 mg, 0.33 mmol, 1.5 eq.) were added to the solution, which was then purged with argon for 5 minutes and stirred for 12 hours at 70 °C. The reaction mixture was allowed to cool down to room temperature and the DMF was removed under reduced pressure. The crude product was purified by flash column chromatography (SiO_2, DCM/methanol = 99:1) yielding **Pd(L$_{mDMA}$)NHC** (52 mg, 0.079 mmol, 49 %) as a yellow crystalline solid.

EM-MS-ESI (MeOH, $C_{23}H_{24}N_5O_5Pd$): calcd. [**Pd(L$_{mDMA}$)NHC**+Na]$^+$ = 577.07951, found [**Pd(L$_{mDMA}$)NHC**+Na]$^+$ = 577.07819.

^{1}H-NMR (400 MHz, DCM-d_2): δ (ppm) = 7.82 (t, $^3J_{HH}$ = 7.8 Hz, 1H, H$_5$), 7.52 (t, $^3J_{HH}$ = 7.6 Hz, $^4J_{HH}$ = 0.8 Hz, 1H, H$_4$), 7.52 (t, $^3J_{HH}$ = 7.6 Hz, $^4J_{HH}$ = 0.8 Hz, 1H, H$_4$), 7.38 (d, $^3J_{HH}$ = 8.7 Hz, 1H, H$_9$), 6.42 (dd, $^3J_{HH}$ = 8.7 Hz, $^4J_{HH}$ = 2.6 Hz, 1H, H$_{10}$), 5.85 (d, $^4J_{HH}$ = 2.6 Hz, 1H, H$_{12}$), 4.45 (s, 3H, H$_{25}$), 4.31 (s, 3H, H$_{26}$), 3.81 (s, 3H, H$_{28}$), 3.39 (s, 3H, H$_{27}$), 2.86 (s, 6H, H$_{15}$).

^{13}C{^{1}H}-NMR (101 MHz, DCM-d_2): δ (ppm) = 185.0 (C$_{16}$), 172.6 (C$_7$), 162.7 (C$_1$), 153.7 (C$_{21}$), 152.6 (C$_{13}$), 151.8 (C$_{11}$), 151.3 (C$_3$), 151.3 (C$_{23}$), 140.8 (C$_{19}$), 139.9 (C$_5$), 135.3 (C$_8$), 125.7 (C$_9$), 120.7 (C$_{12}$), 120.4 (C$_4$), 118.5 (C$_6$), 110.6 (C$_{18}$), 108.5 (C$_{10}$), 40.5 (C$_{14}$), 38.9 (C$_{25}$), 38.8 (C$_{26}$), 32.4 (C$_{28}$), 28.9 (C$_{27}$).

^{15}N-NMR (41 MHz, DCM-d_2): δ (ppm) = 243 (N$_2$), 167 (N$_{17}$), 160 (N$_{20}$), 150 (N$_{22}$), 108 (N$_{24}$).

Section S3: NMR and mass spectra

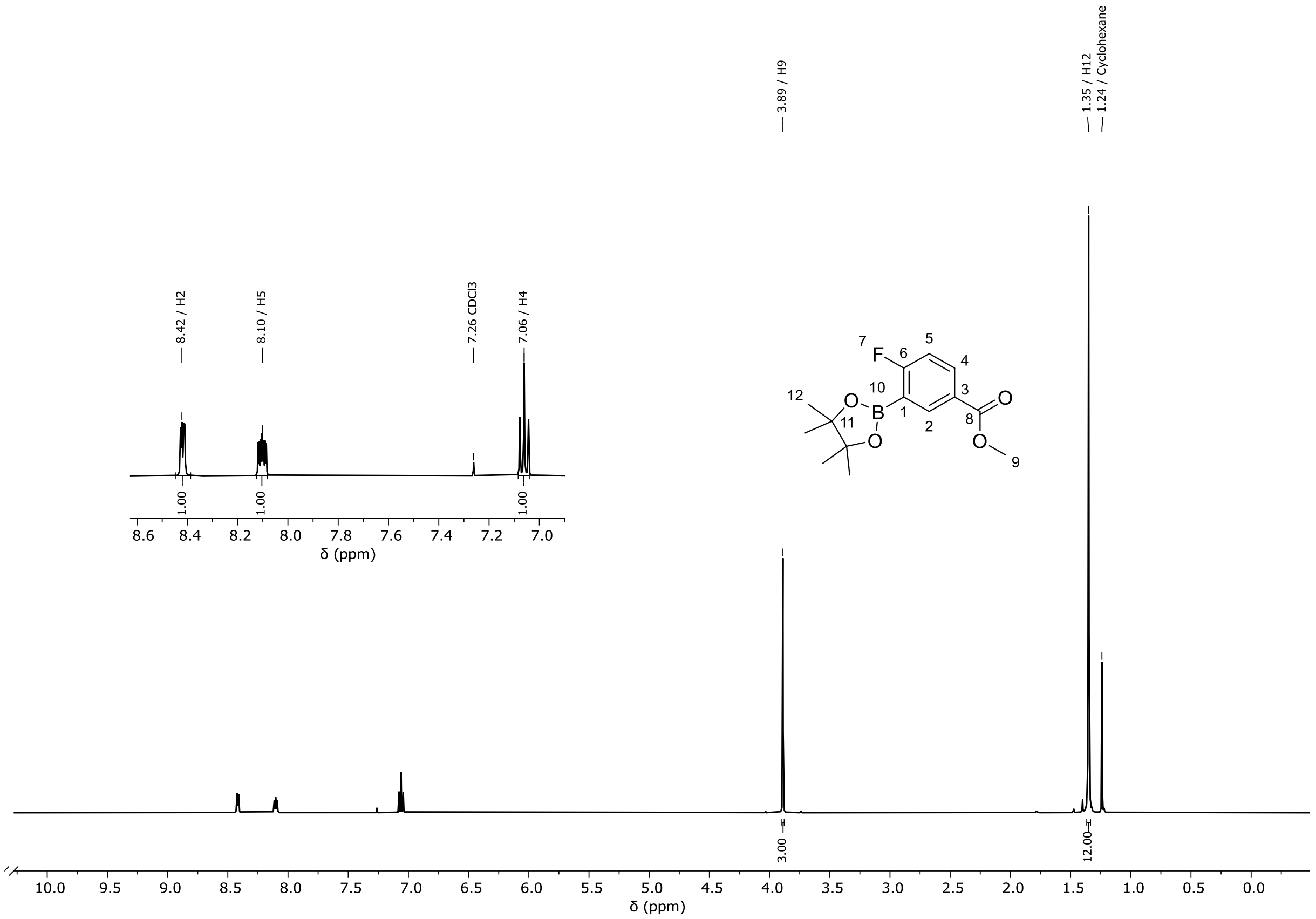

Figure S-1. ^{1}H-NMR spectrum (500 MHz, $CDCl_3$) of **1.1**.

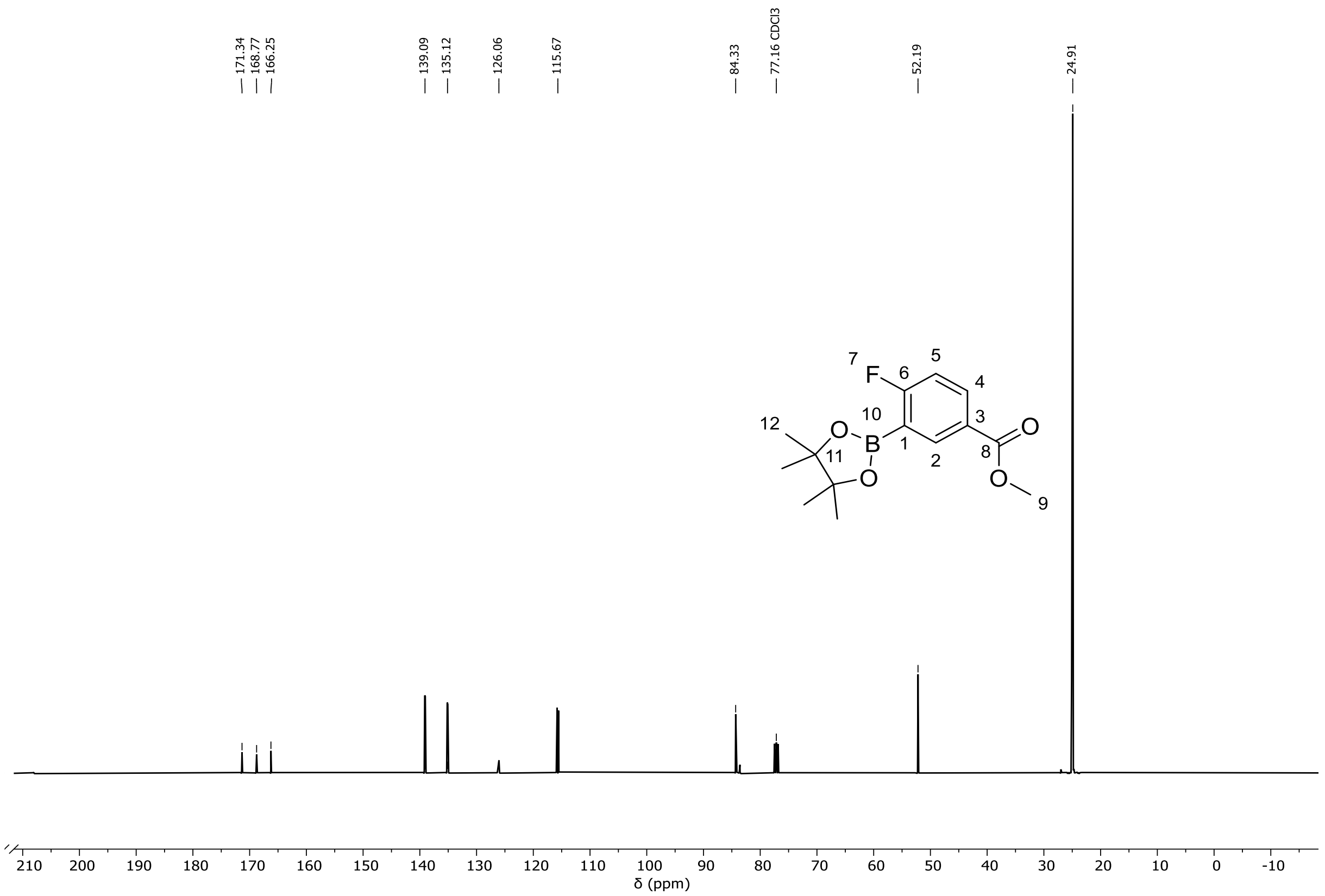

Figure S-2: $^{13}C\{^{1}H\}$-NMR spectrum (126 MHz, $CDCl_3$) of **1.1**.

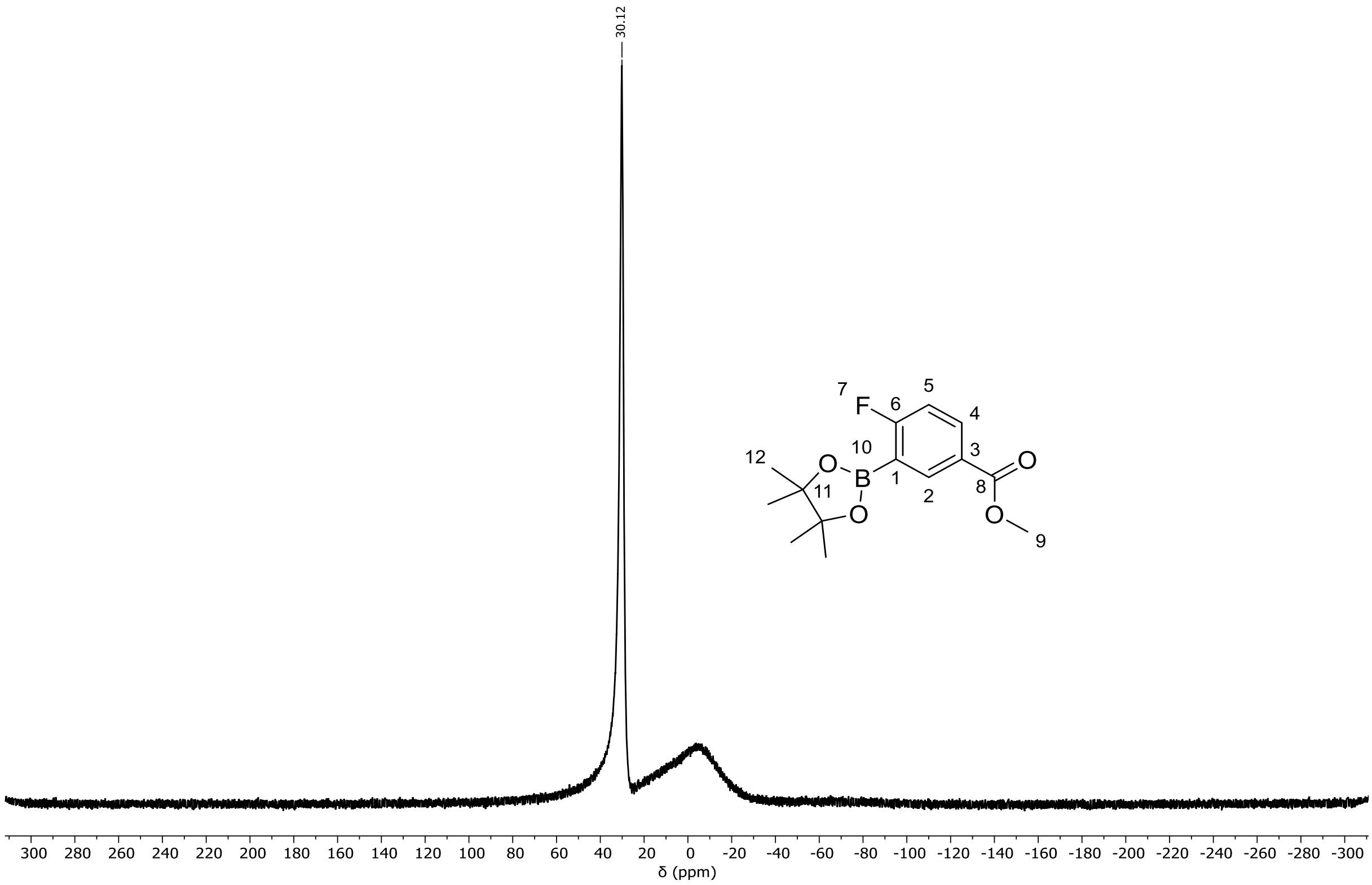

Figure S-3. ^{11}B-NMR spectrum (16 MHz, $CDCl_3$) of **1.1**.

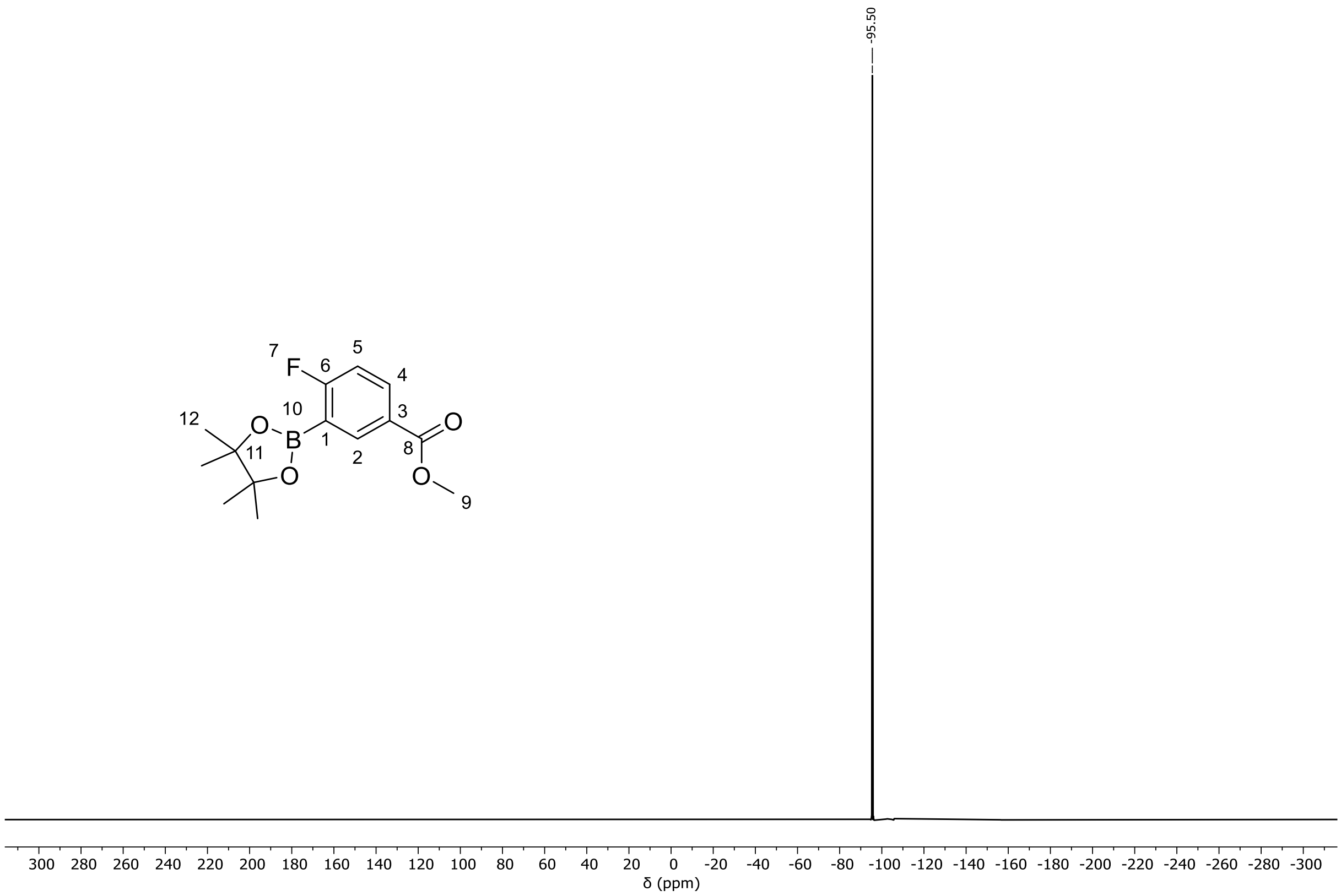

Figure S 4. $^{19}F\{^1H\}$-NMR spectrum (376 MHz, $CDCl_3$) of **1.1**.

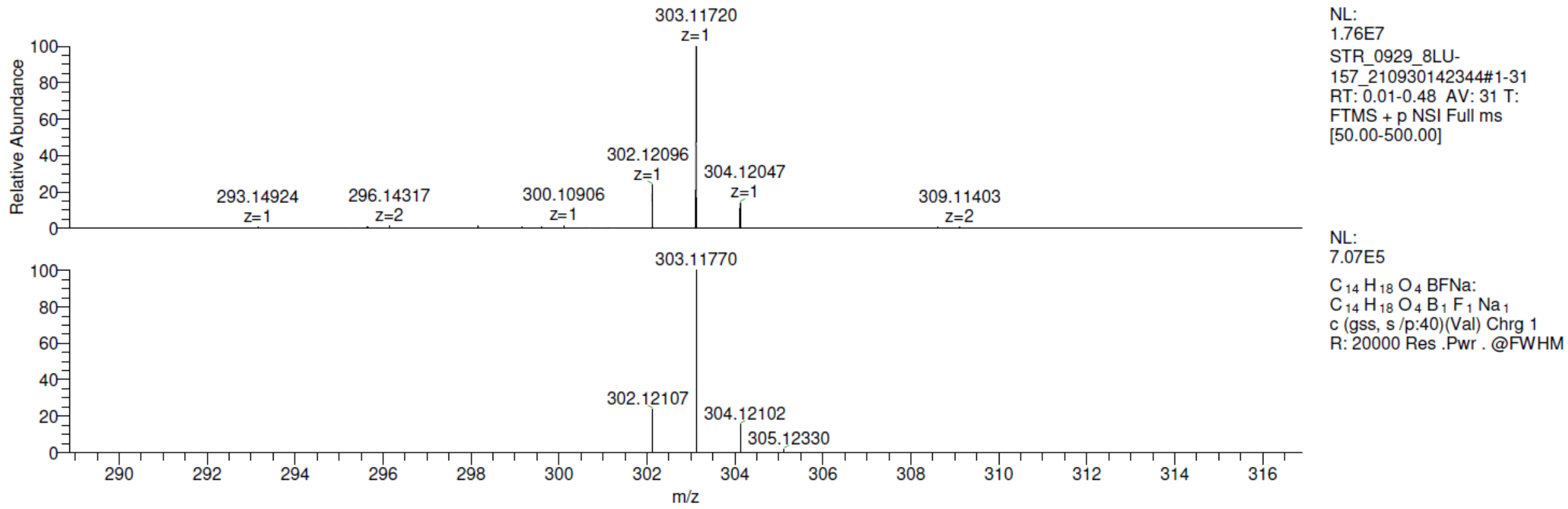

Figure S-5. Mass spectrum of **1.1** (MeOH). Additional simulation of the [**1.1**+Na]$^+$ adduct.

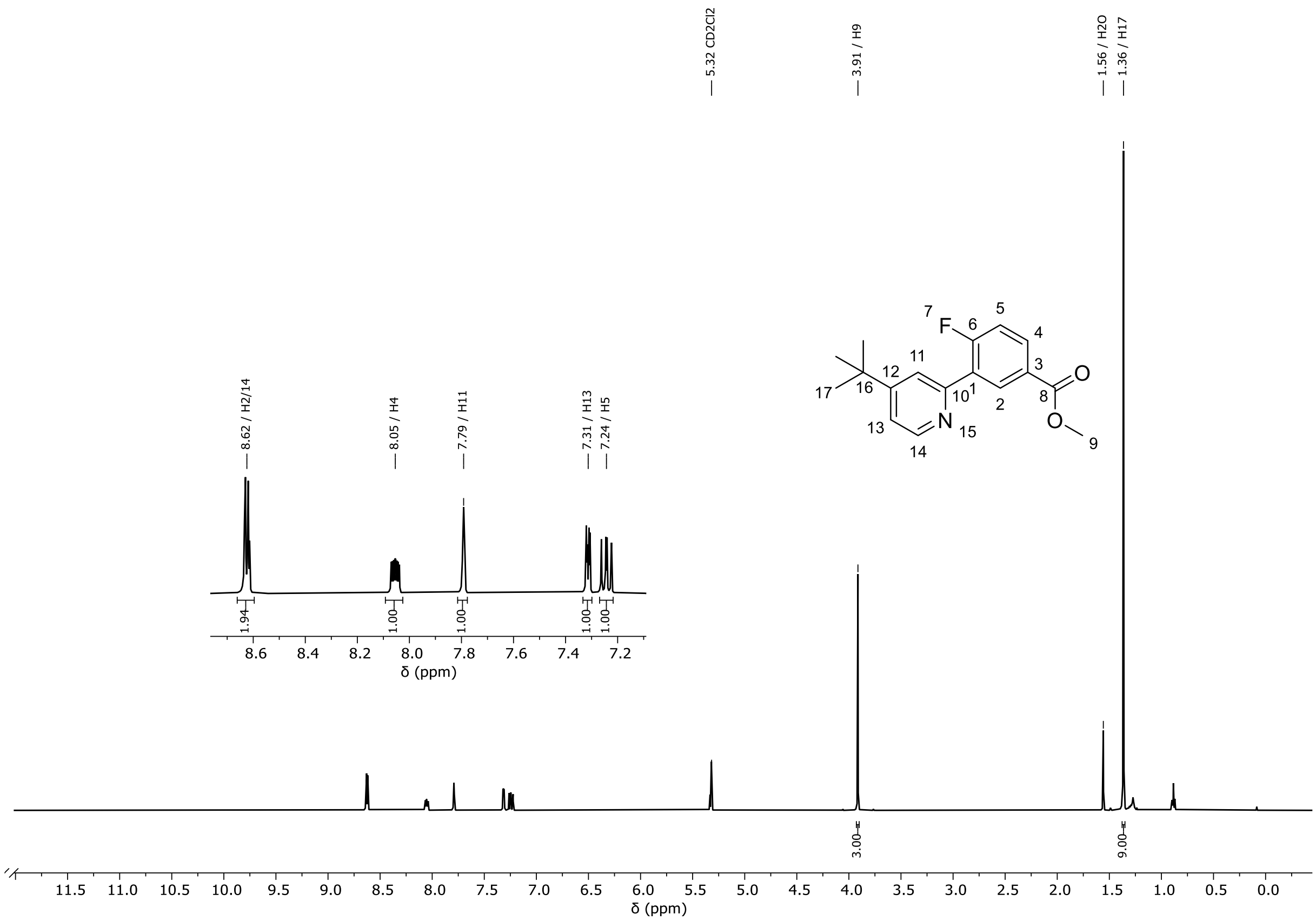

Figure S-6. ^{1}H-NMR spectrum (500 MHz, DCM-d_2) of **1.2**.

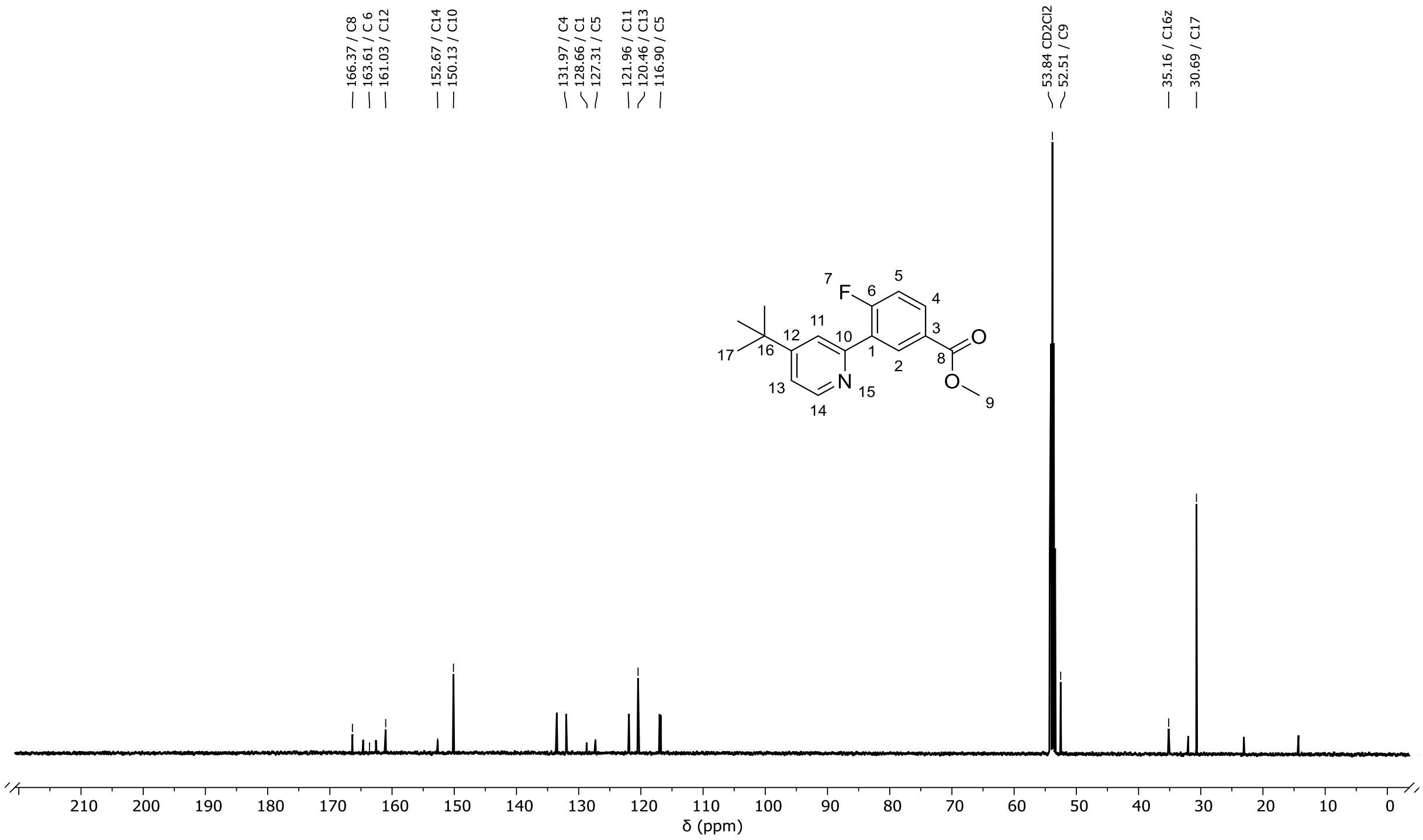

Figure S-7. $^{13}C\{^1H\}$-NMR spectrum (126 MHz, DCM-d_2) of **1.2**.

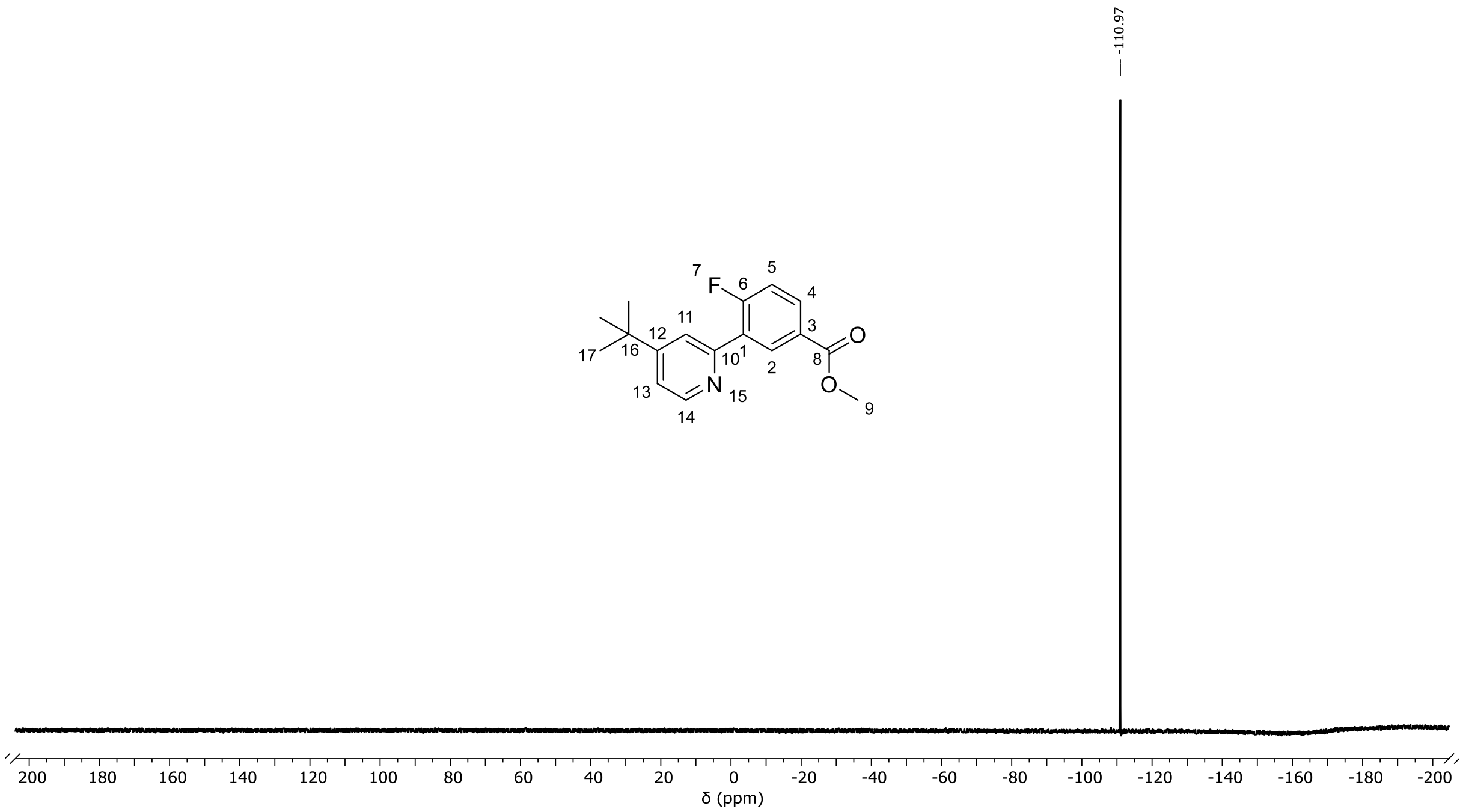

Figure S-8. $^{19}F\{^1H\}$-NMR spectrum (376 MHz, DCM-d_2) of **1.2**.

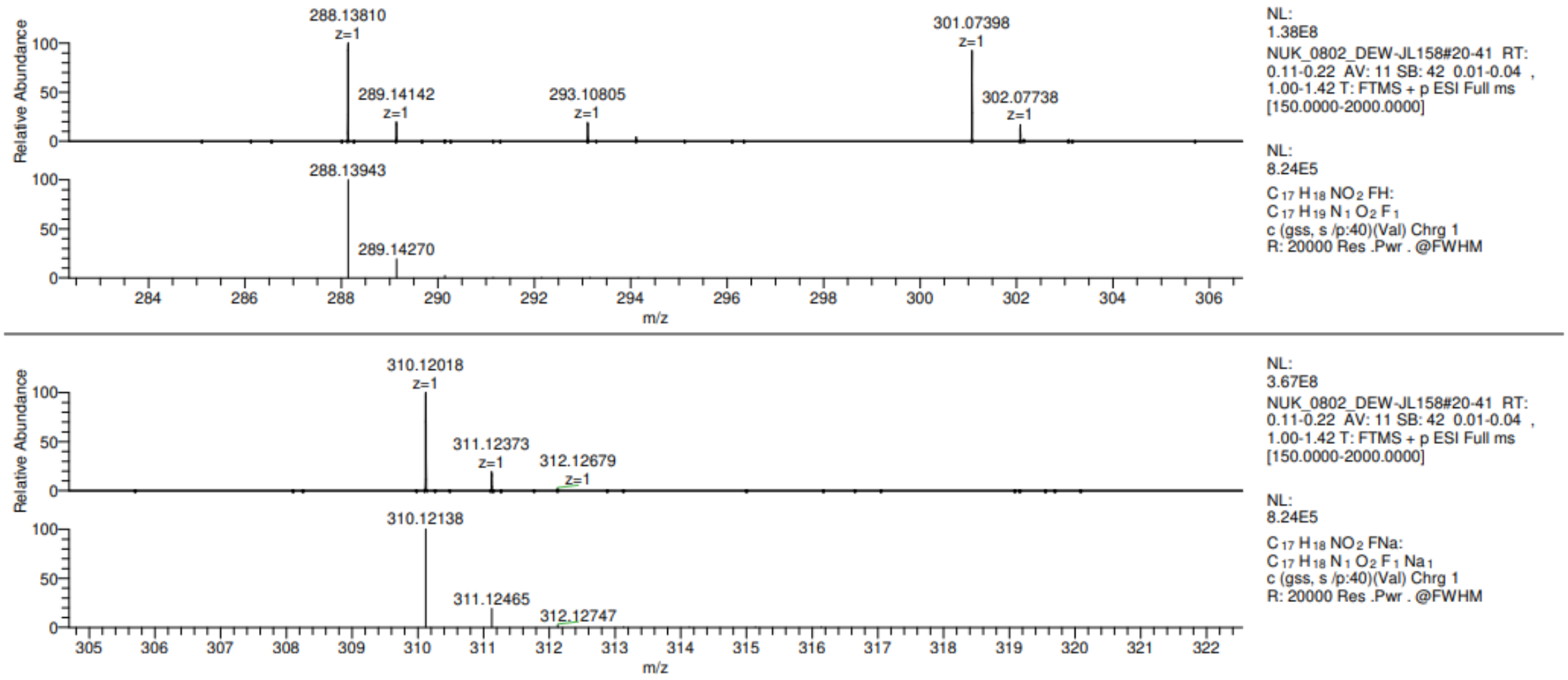

Figure S-9. Mass spectrum of **1.2** (MeOH). Additional simulation of the [**1.2**+H]$^+$ and the [**1.2**+Na]$^+$ adduct.

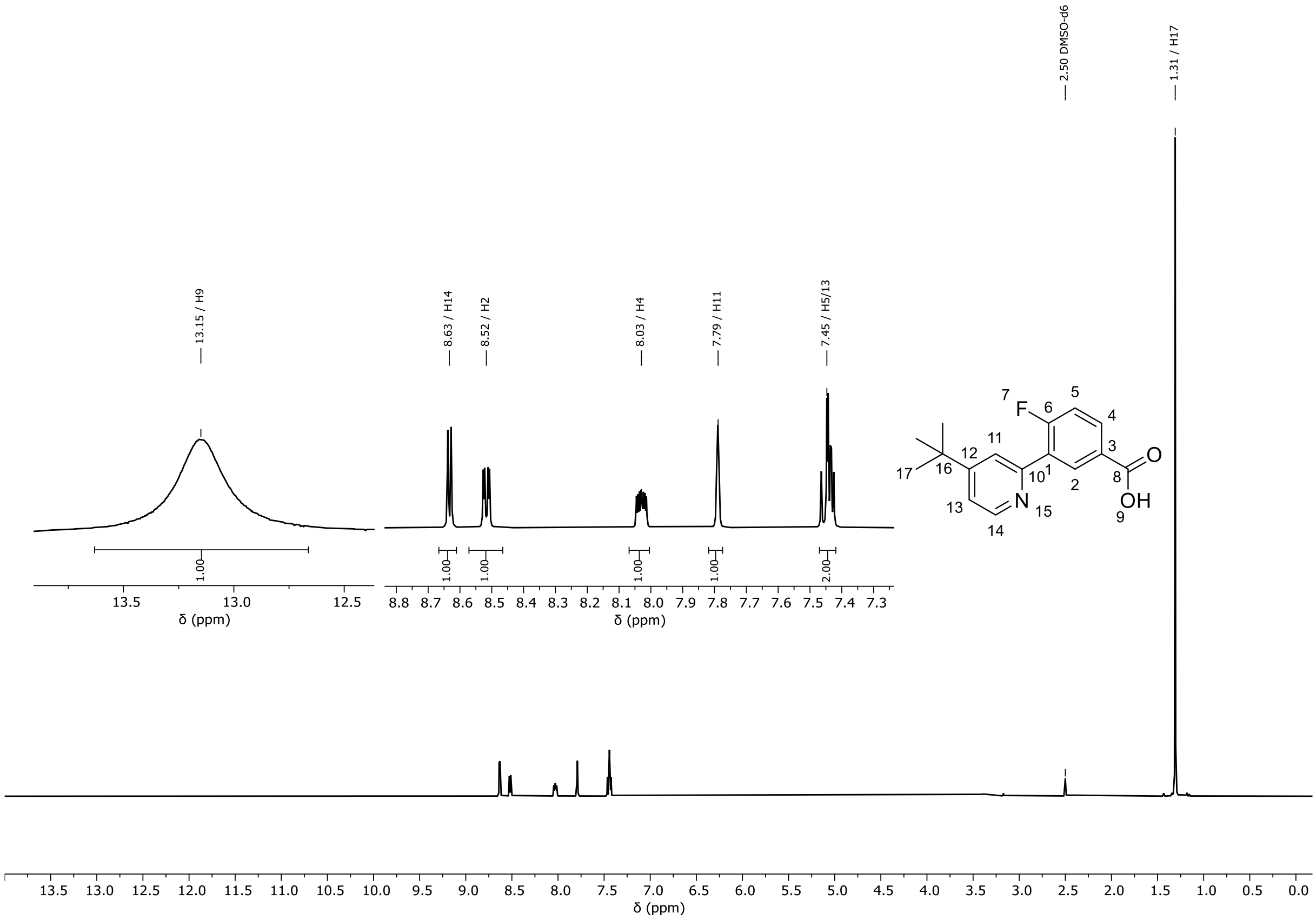

Figure S-10. ^{1}H-NMR spectrum (500 MHz, DMSO-d_6) of **L$_{NCO}$**.

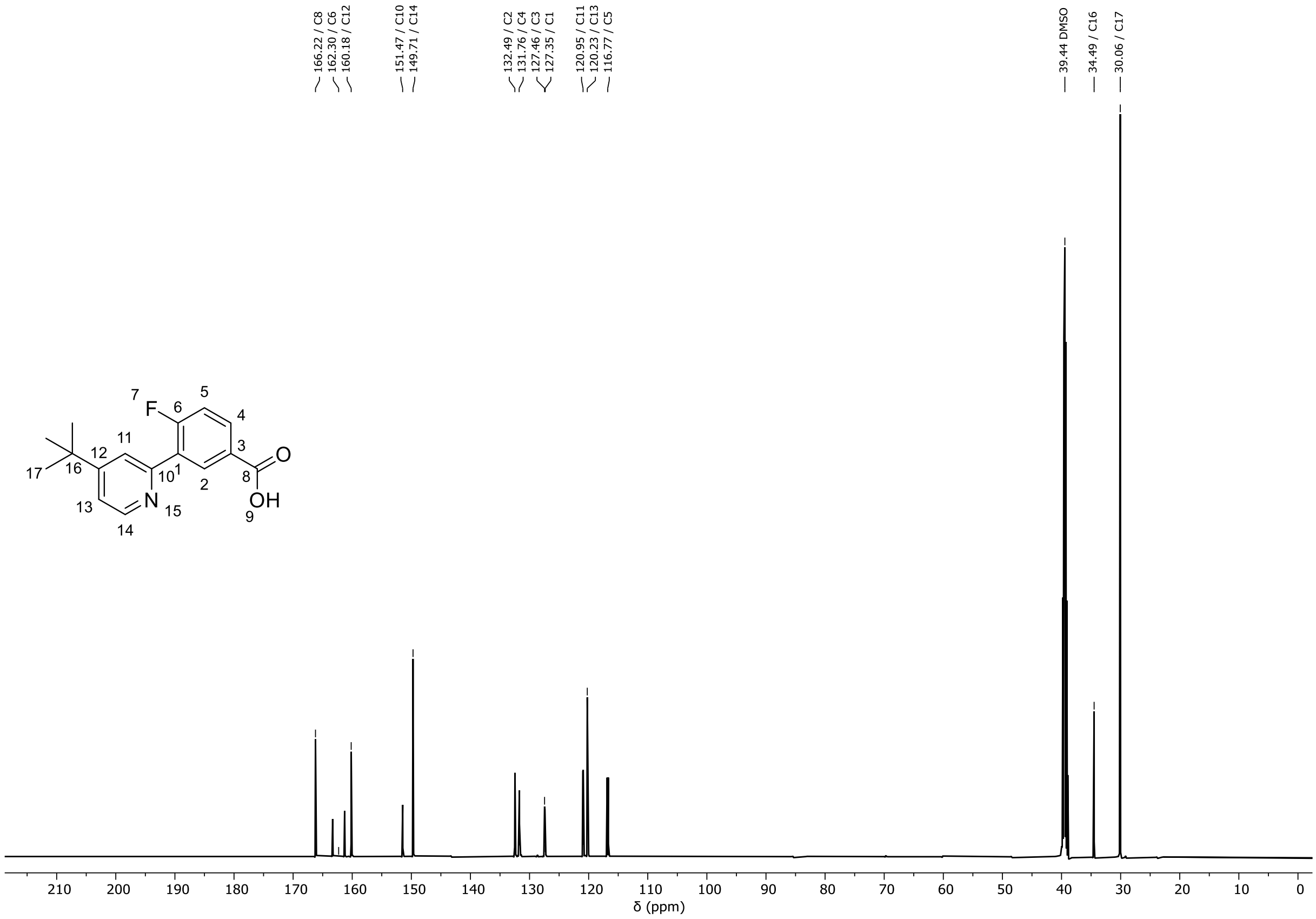

Figure S-11. $^{13}C\{^{1}H\}$-NMR spectrum (126 MHz, DMSO-d_6) of $\mathbf{L_{NCO}}$.

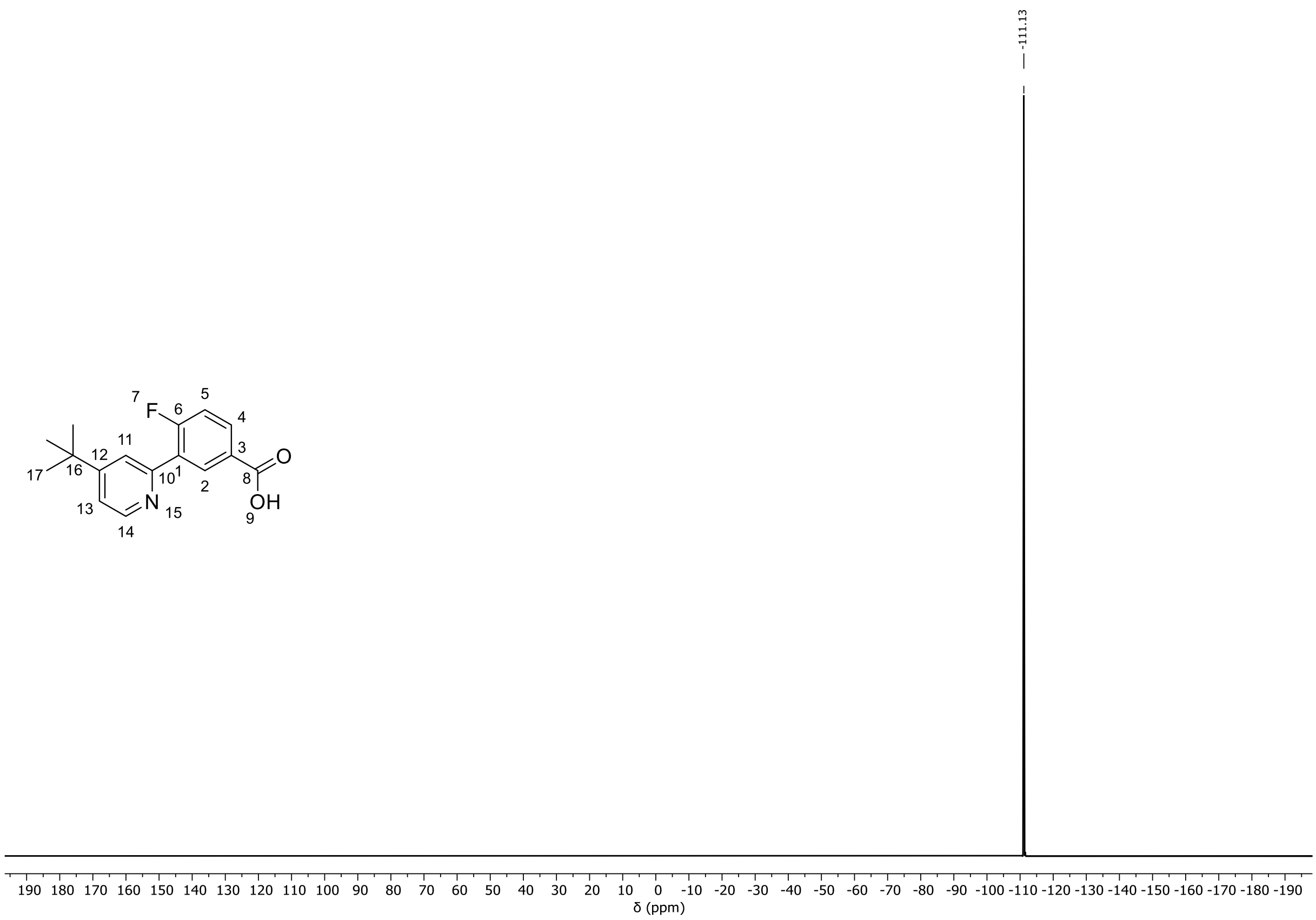

Figure S-12. $^{19}F\{^{1}H\}$-NMR spectrum (376 MHz, DMSO-d_6) of $\mathbf{L_{NCO}}$.

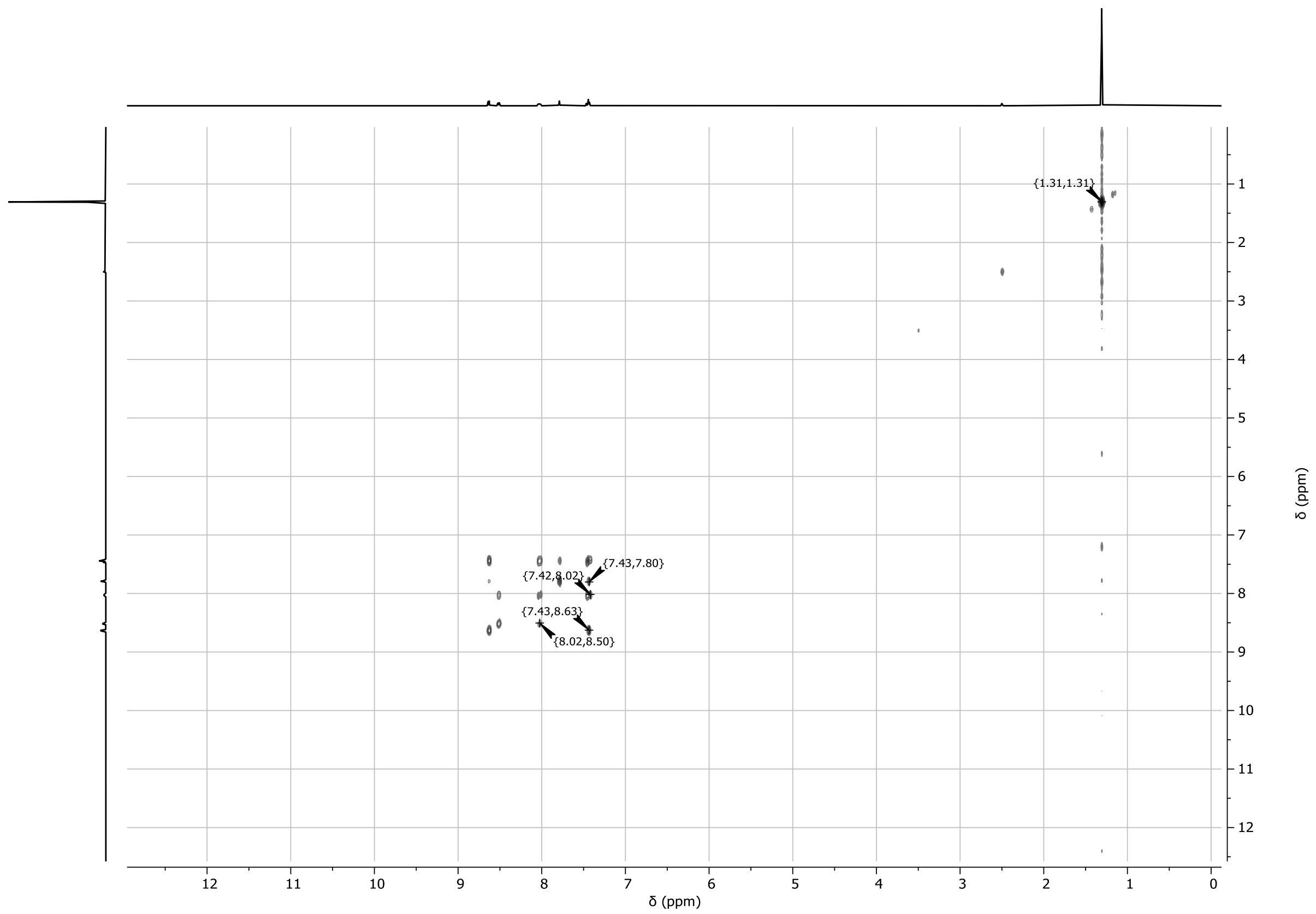

Figure S-13. HH-COSY-NMR spectrum (500 MHz, DMSO-d_6) of $\mathbf{L_{NCO}}$.

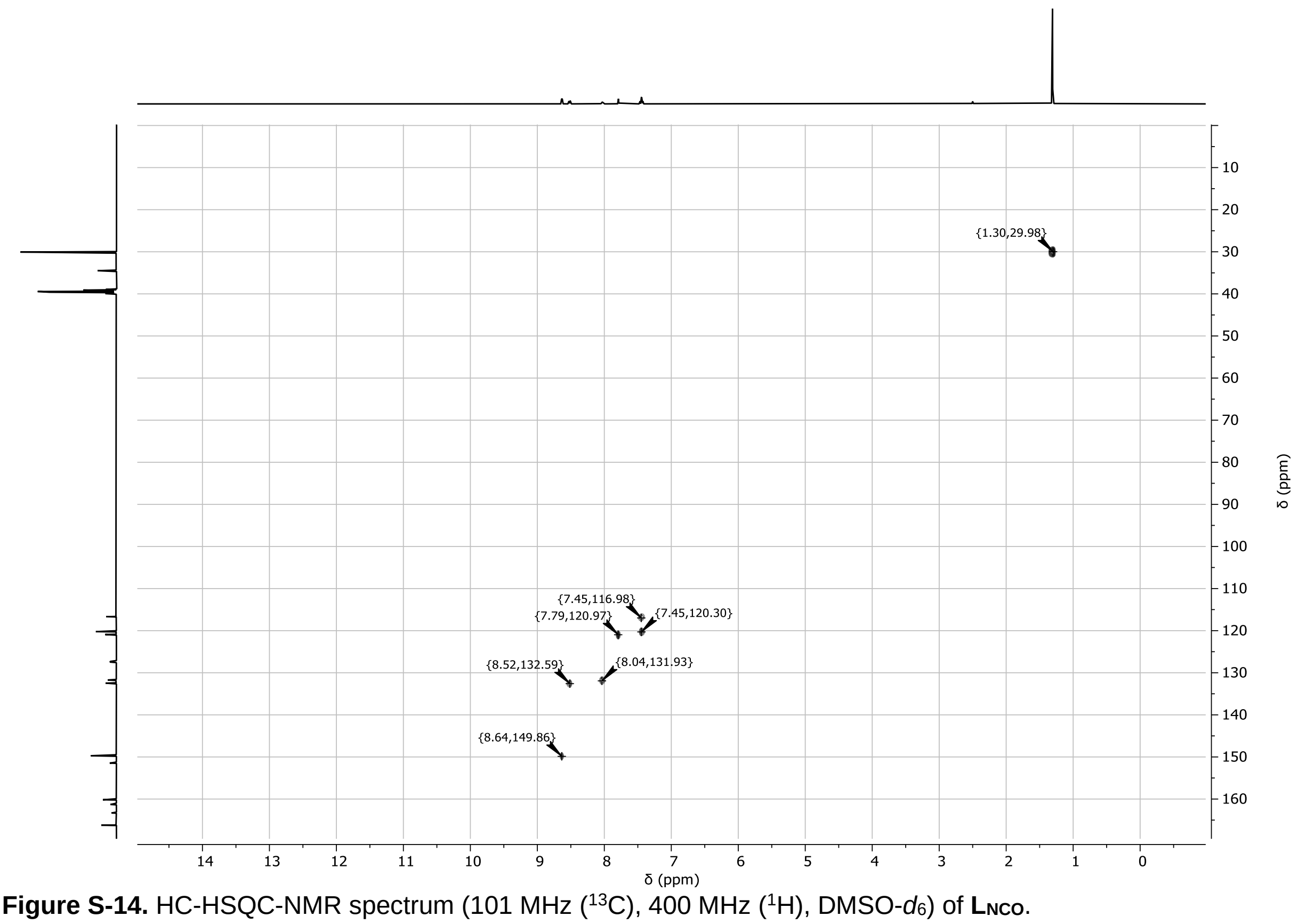

Figure S-14. HC-HSQC-NMR spectrum (101 MHz (^{13}C), 400 MHz (^{1}H), DMSO-d_6) of $\mathbf{L_{NCO}}$.

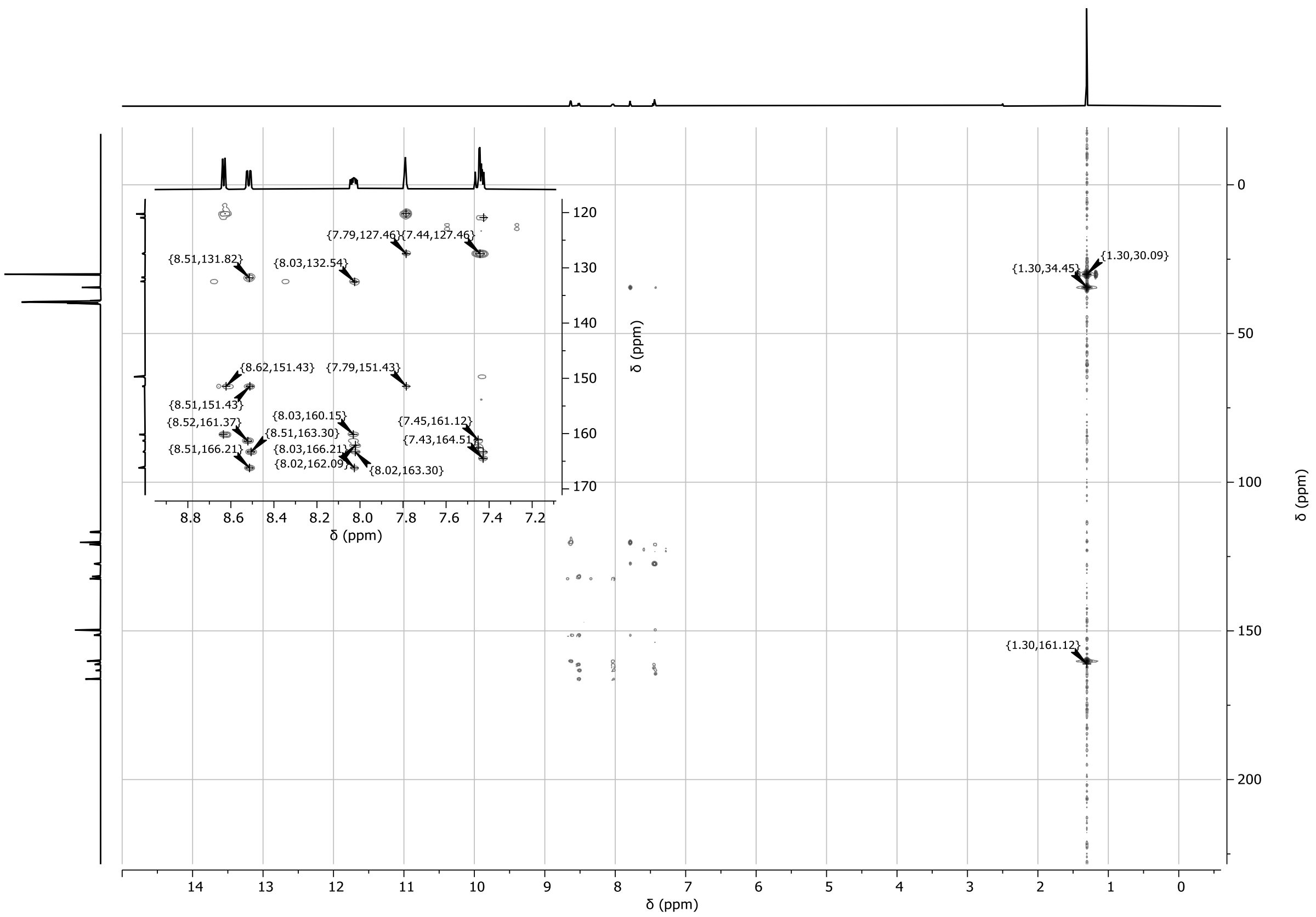

Figure S-15. HC-HMBC-NMR spectrum (101 MHz (^{13}C), 400 MHz (^{1}H), DMSO-d_6) of $\mathbf{L_{NCO}}$.

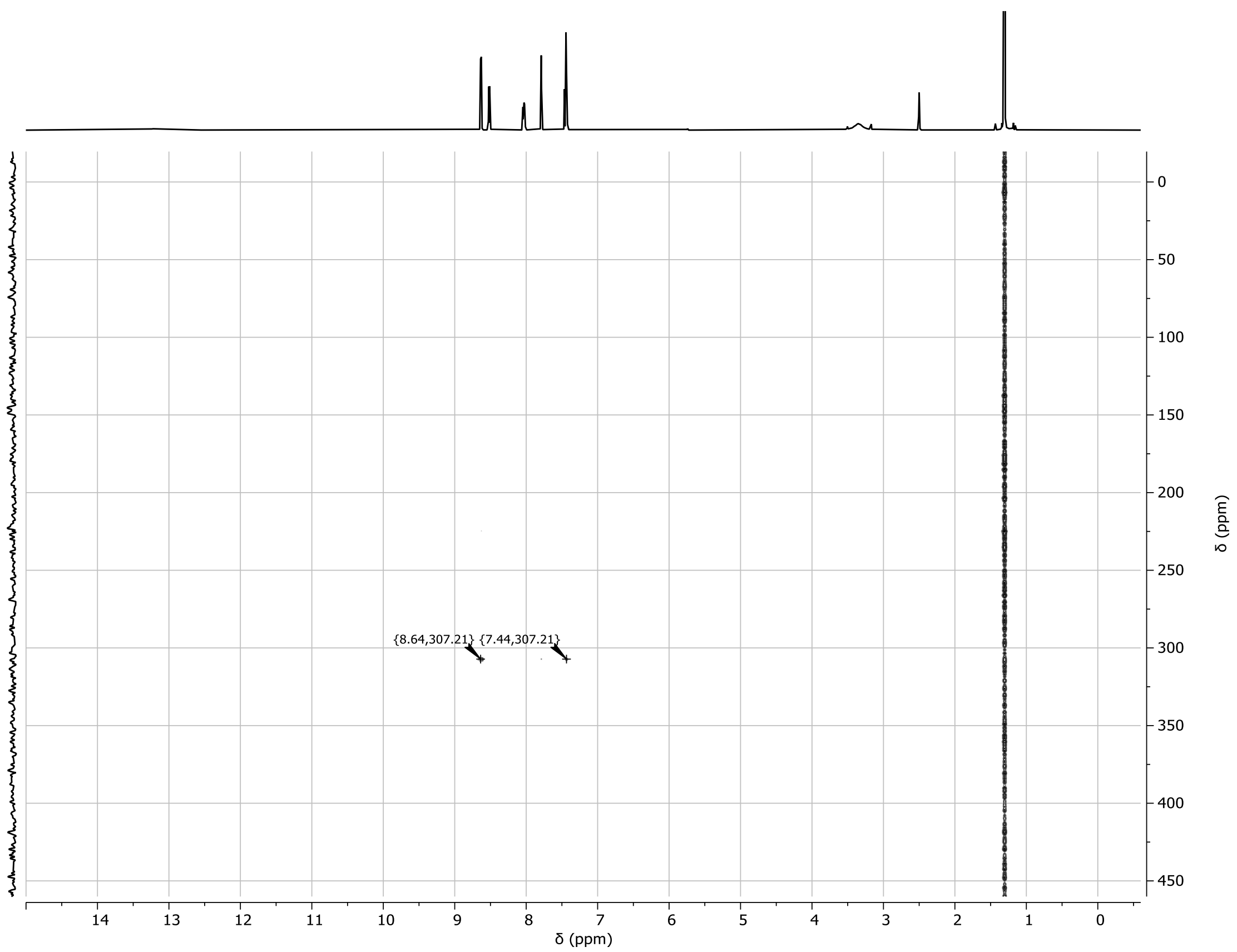

Figure S-16. HN-HMBC-NMR spectrum (51 MHz (^{13}C), 500 MHz (^{1}H), DMSO-d_6) of $\mathbf{L_{NCO}}$.

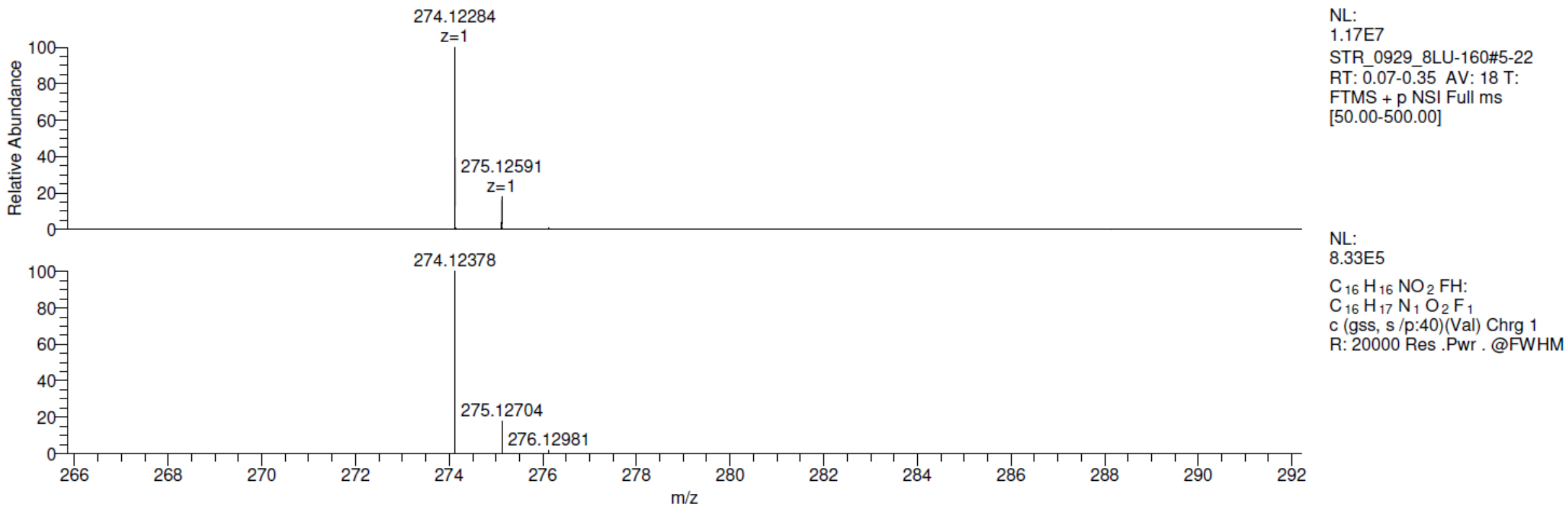

Figure S-17. Mass spectrum of $\mathbf{L_{NCO}}$ (MeOH). Additional simulation of the $[\mathbf{L_{NCO}}+H]^+$ adduct.

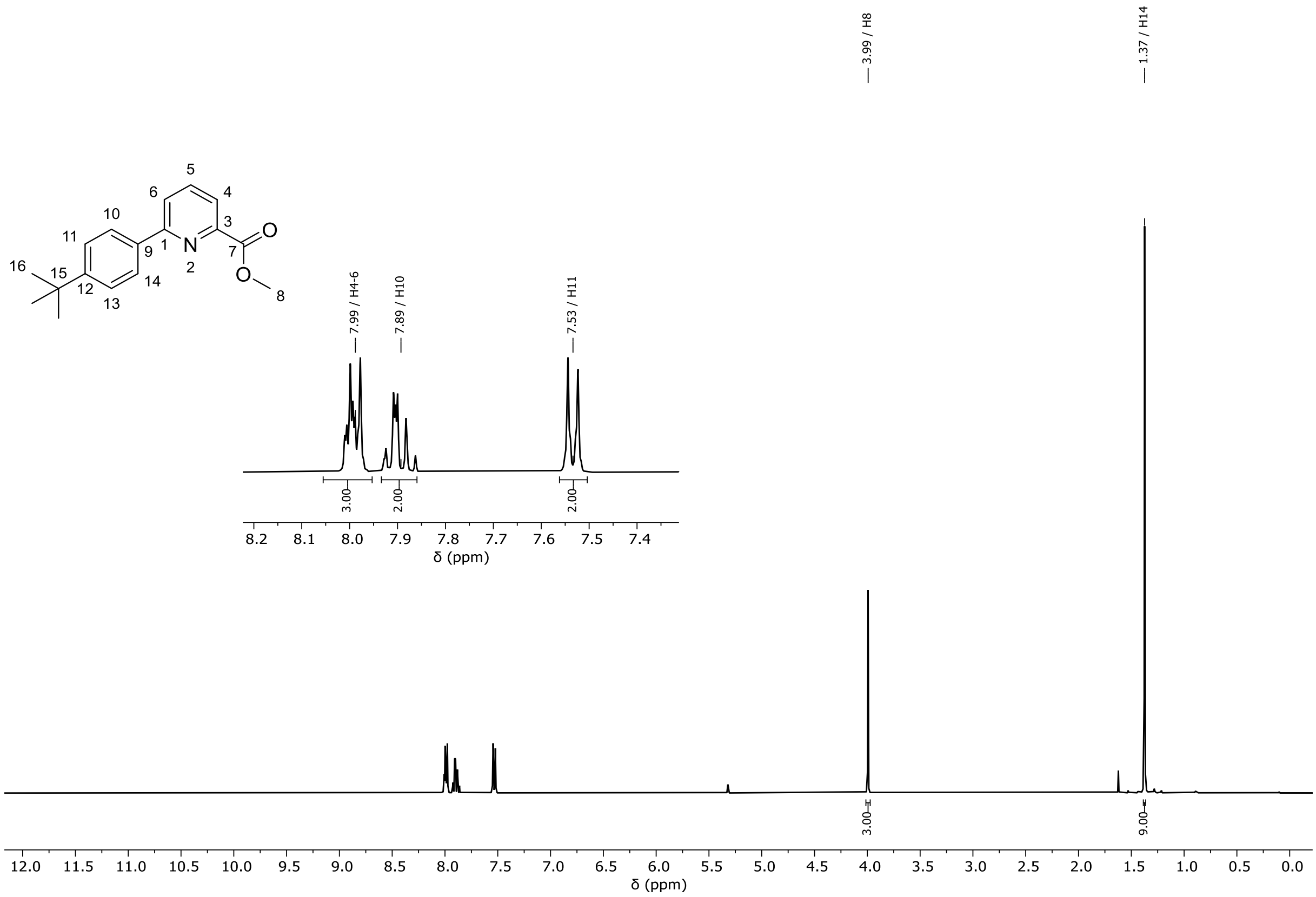

Figure S 18. ^{1}H-NMR spectrum (400 MHz, DCM-d_2) of **2**.

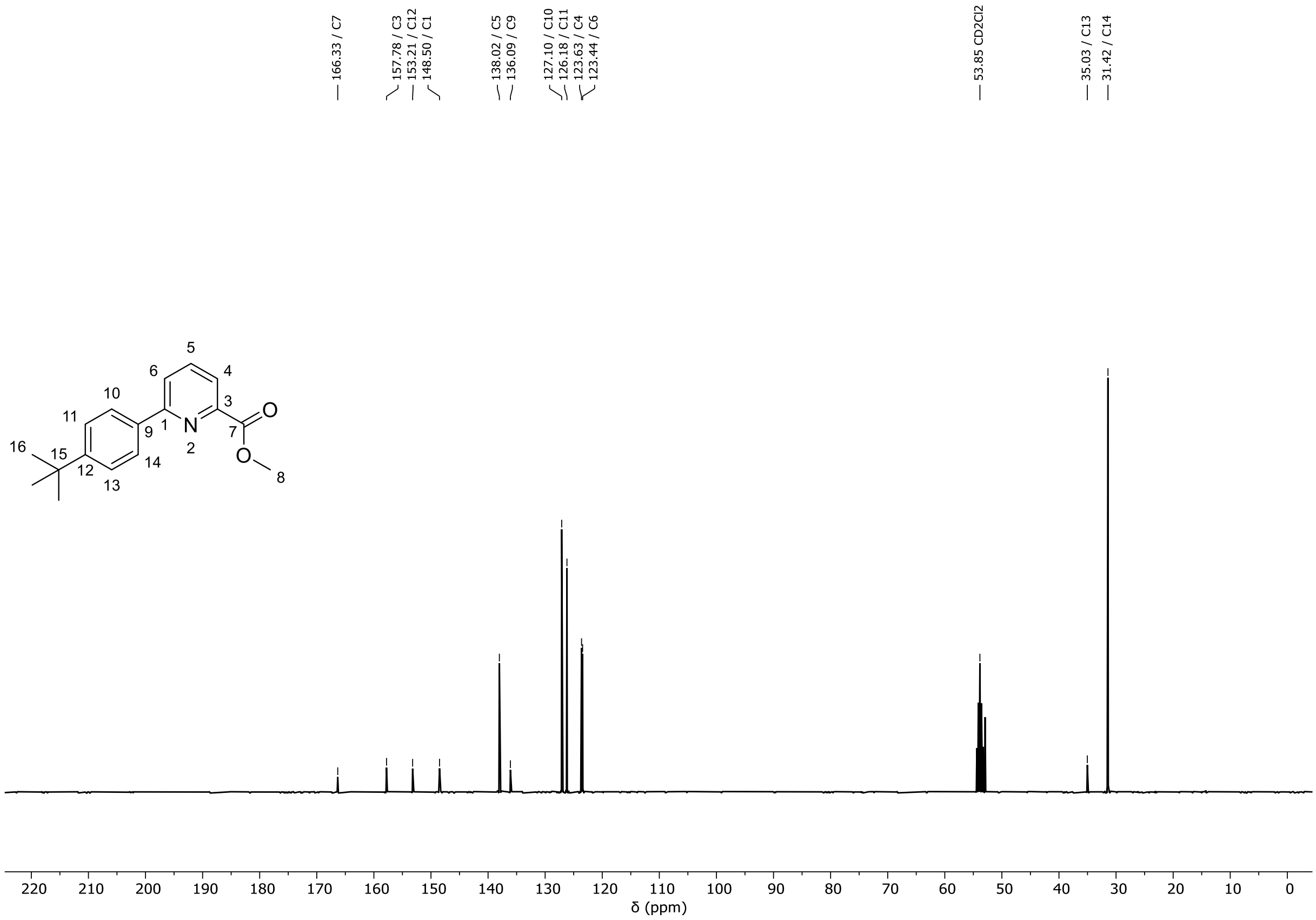

Figure S-19. $^{13}C\{^{1}H\}$-NMR spectrum (101 MHz, DCM-d_2) of **2**.

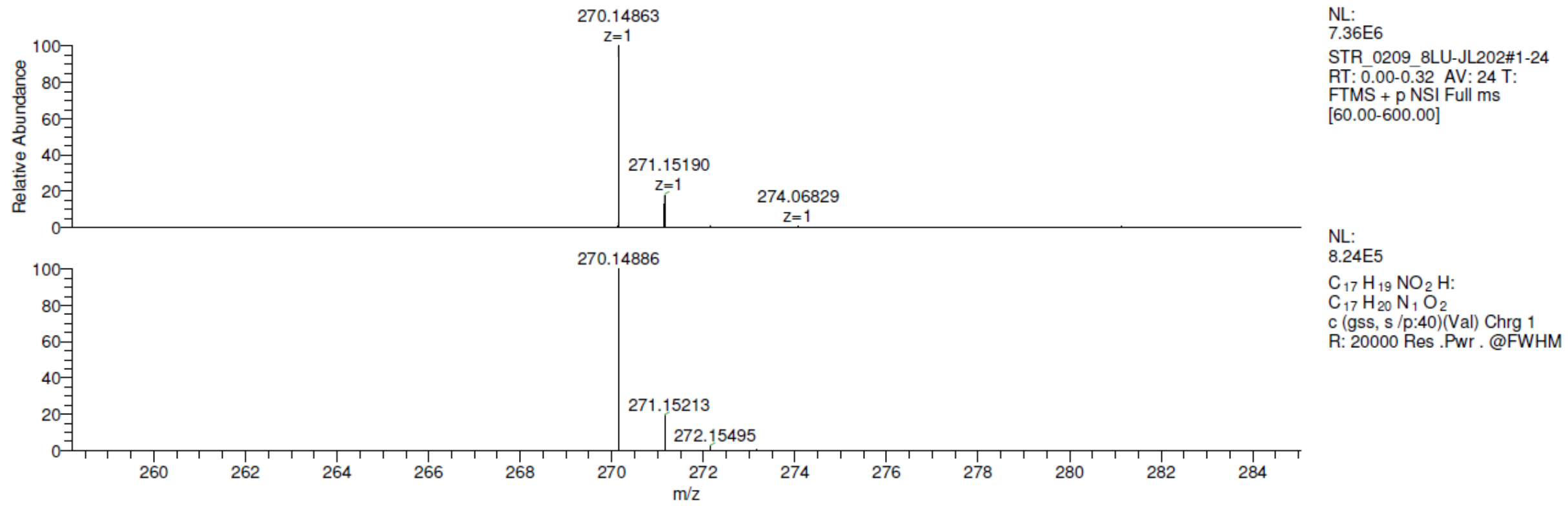

Figure S-20. Mass spectrum of **2** (MeOH). Additional simulation of the [**2**+H]$^+$ adduct.

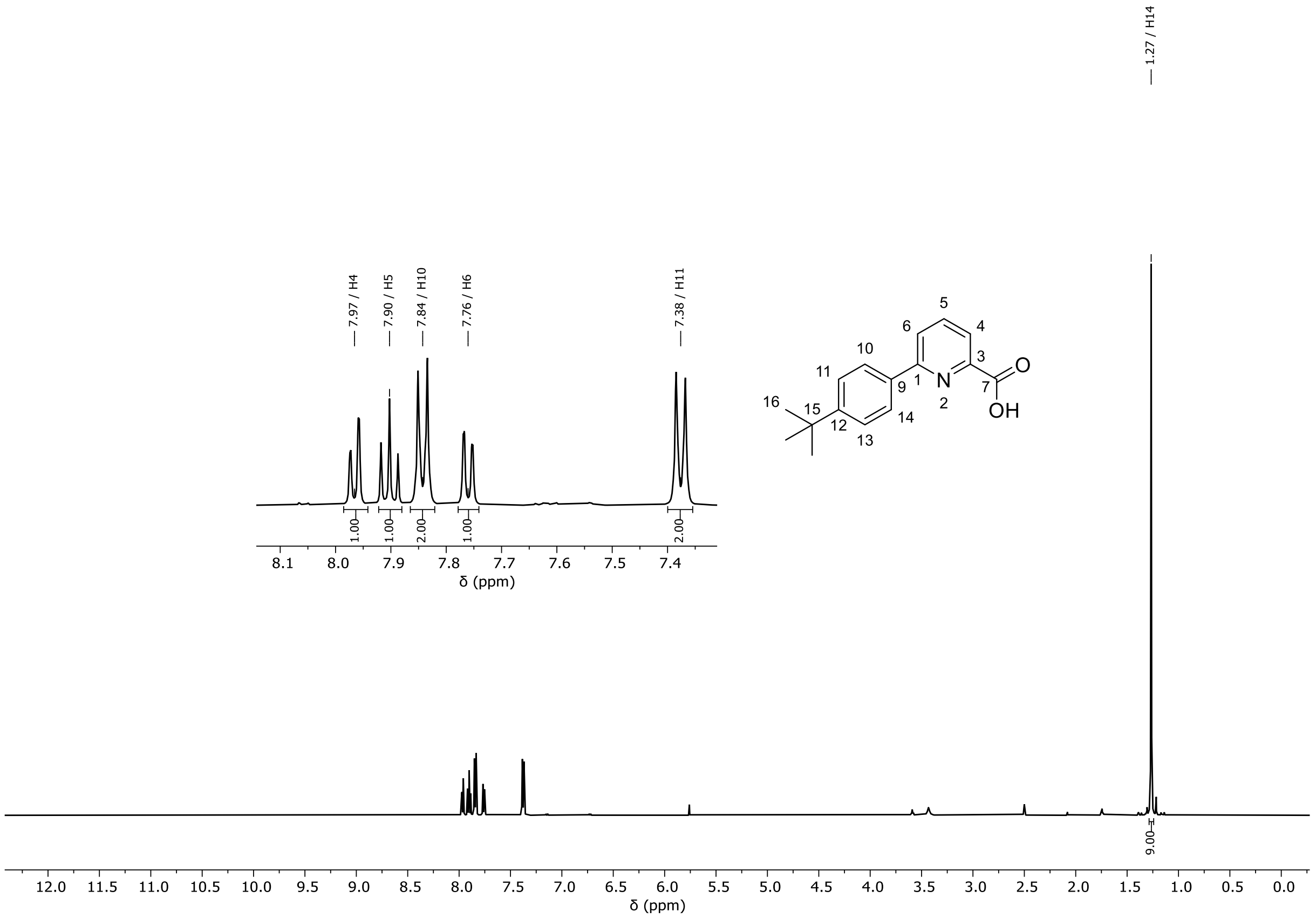

Figure S-21. ^{1}H-NMR spectrum (500 MHz, DMSO-d_6) of $\mathbf{L_{tBu}}$.

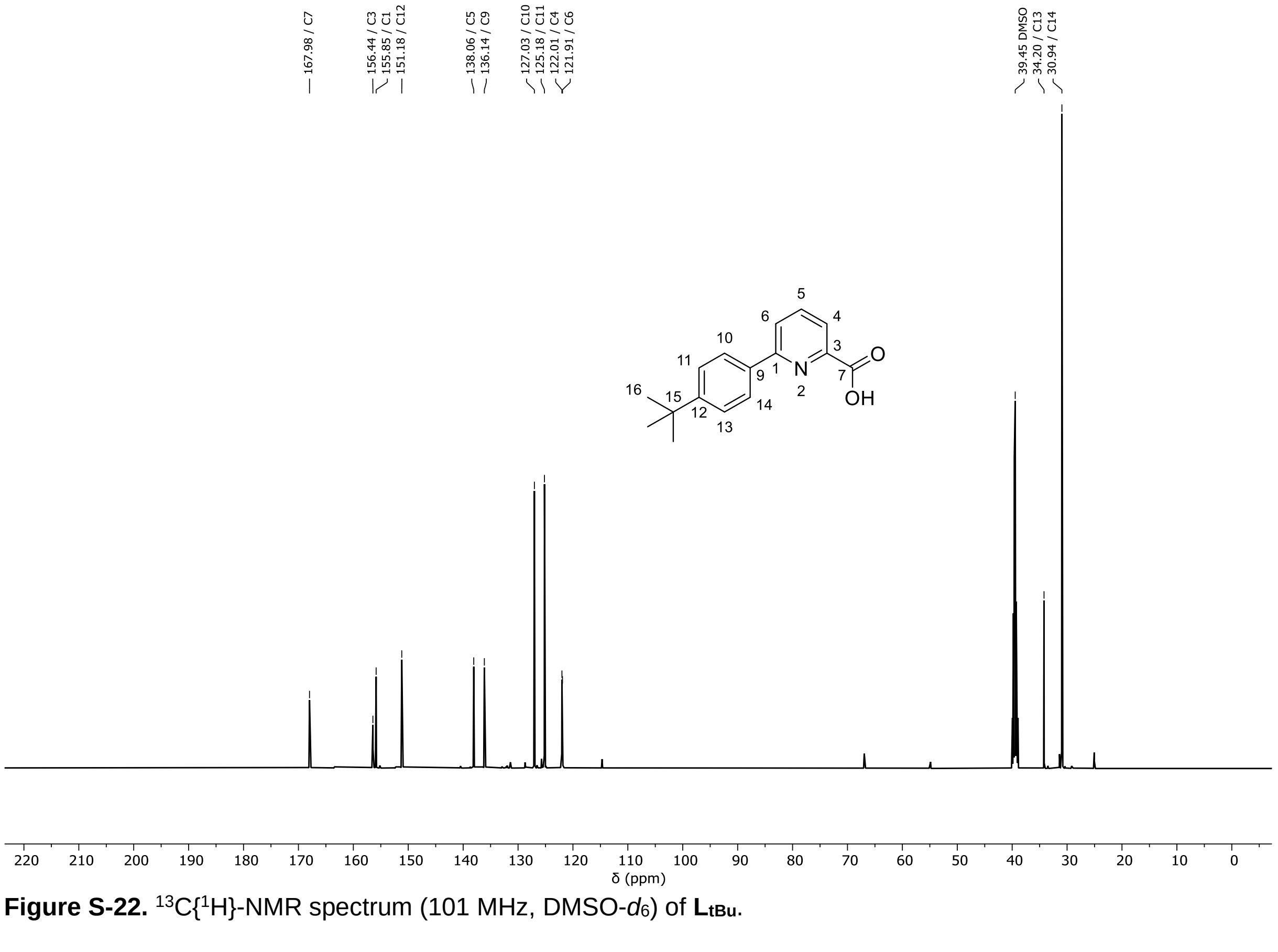

Figure S-22. ^{13}C{^{1}H}-NMR spectrum (101 MHz, DMSO-d_6) of $\mathbf{L_{tBu}}$.

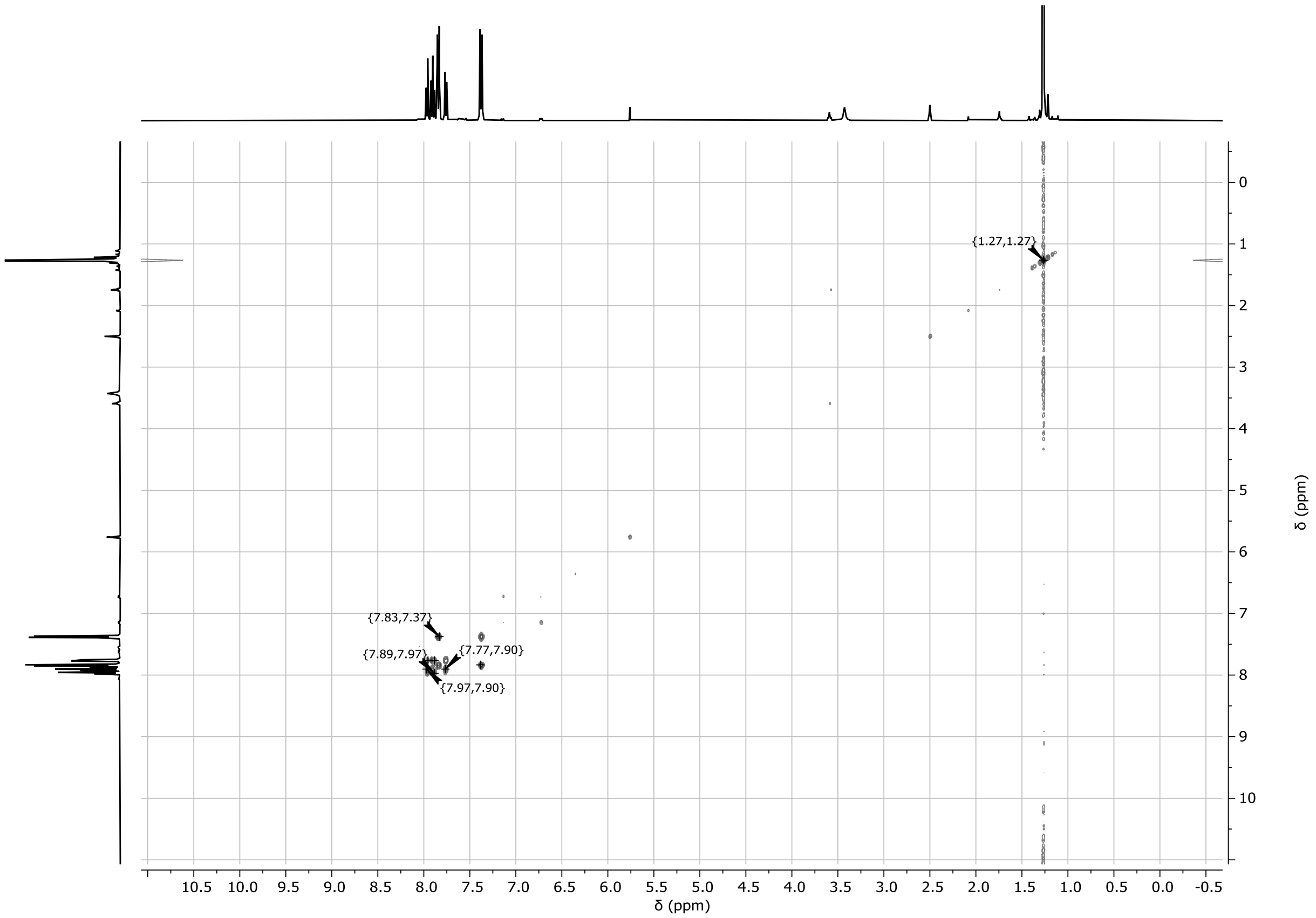

Figure S-23. HH-COSY-NMR spectrum (500 MHz, DMSO-d_6) of $\mathbf{L_{tBu}}$.

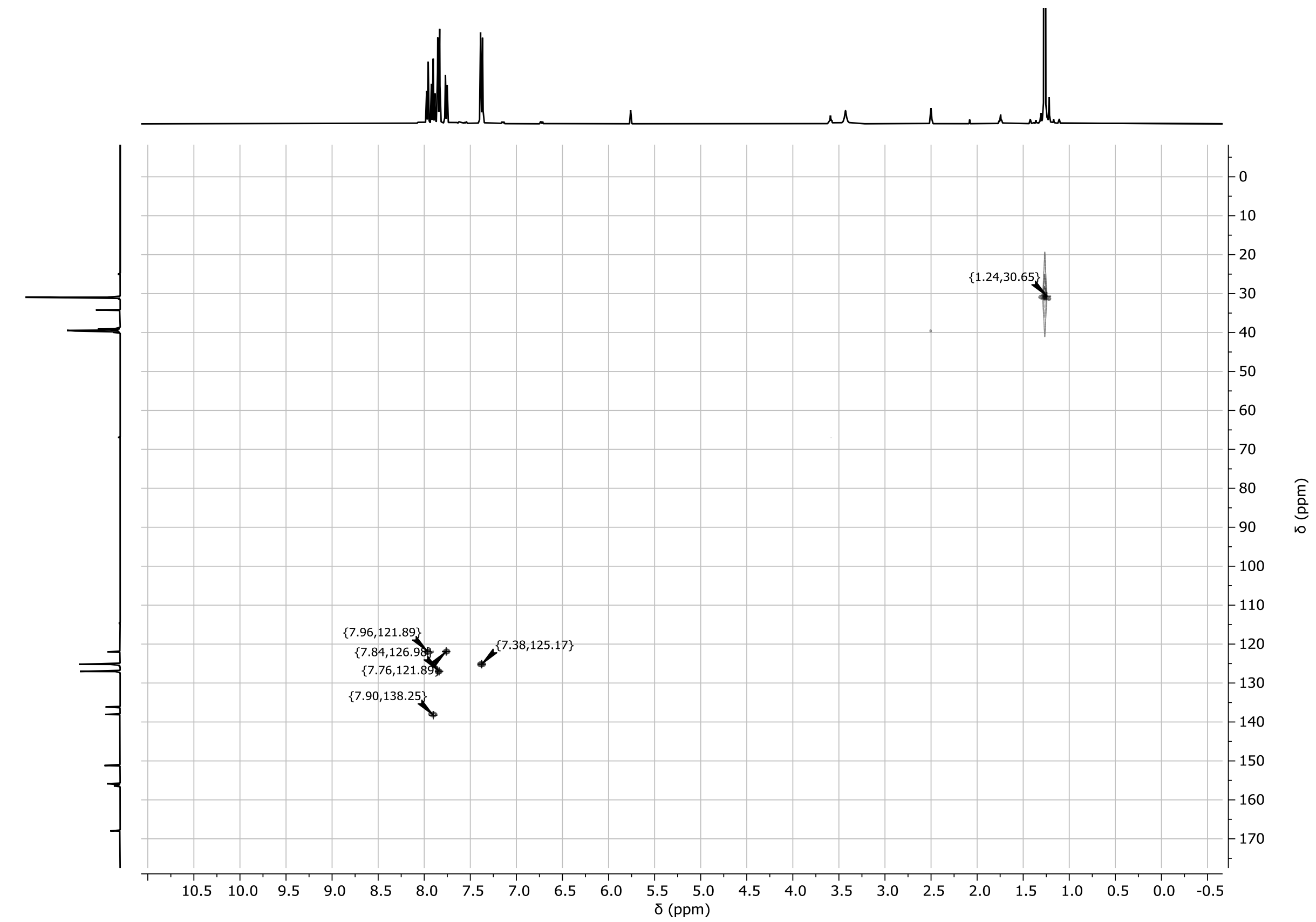

Figure S-24. HC-HSQC-NMR spectrum (121 MHz (^{13}C), 500 MHz (^{1}H), DMSO-d_6) of $\mathbf{L_{tBu}}$.

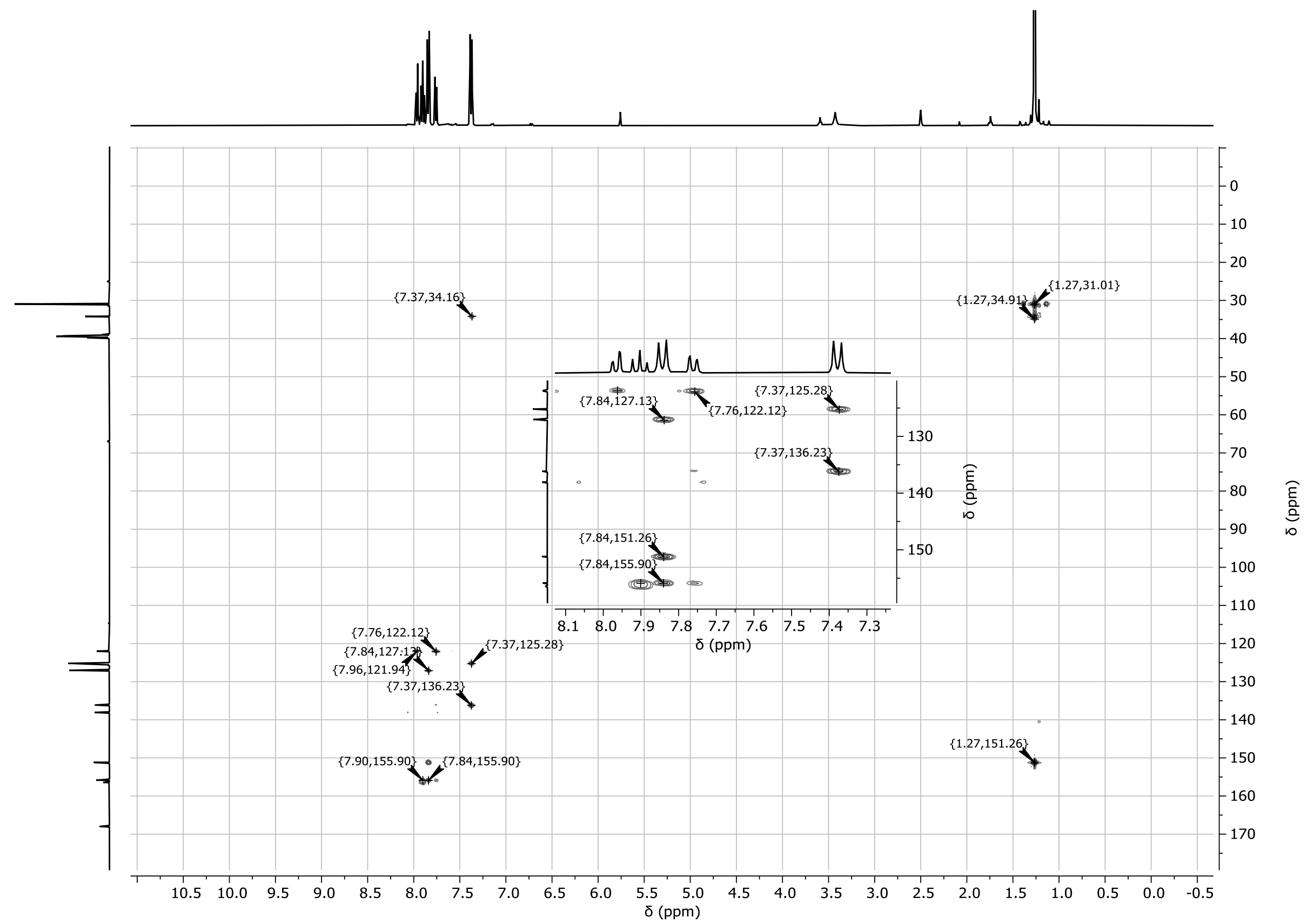

Figure S-25. HC-HSQC-NMR spectrum (121 MHz (^{13}C), 500 MHz (^{1}H), DMSO-d_6) of **L$_{tBu}$**.

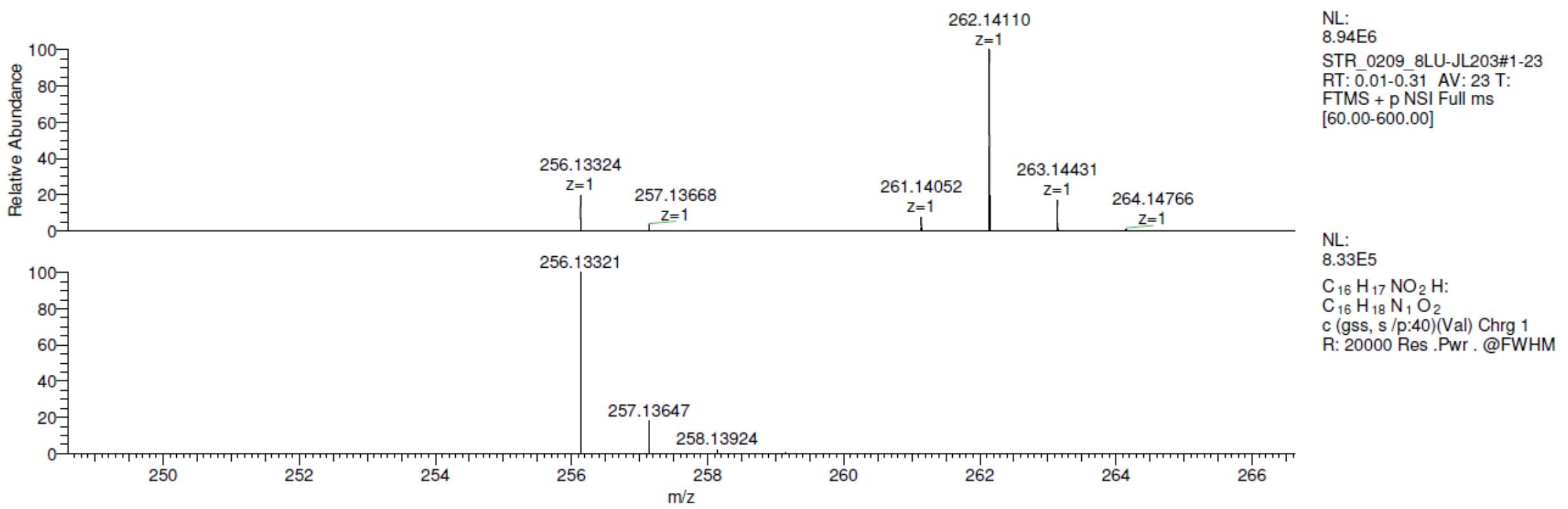

Figure S-26. Mass spectrum of **L$_{tBu}$** (MeOH). Additional simulation of the [**L$_{tBu}$**+H]$^+$ adduct.

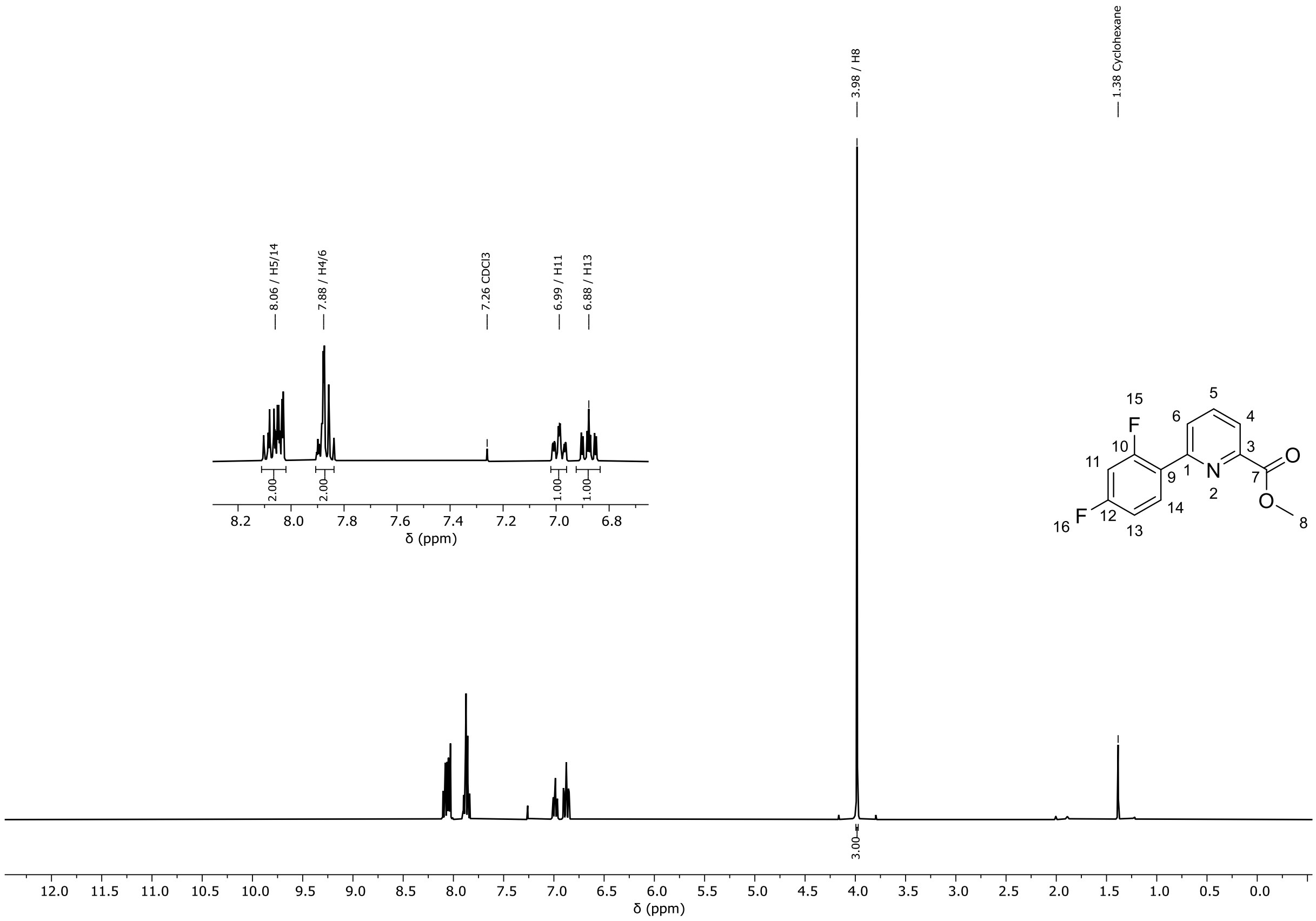

Figure S-27. ^{1}H-NMR spectrum (400 MHz, $CDCl_3$) of **3**.

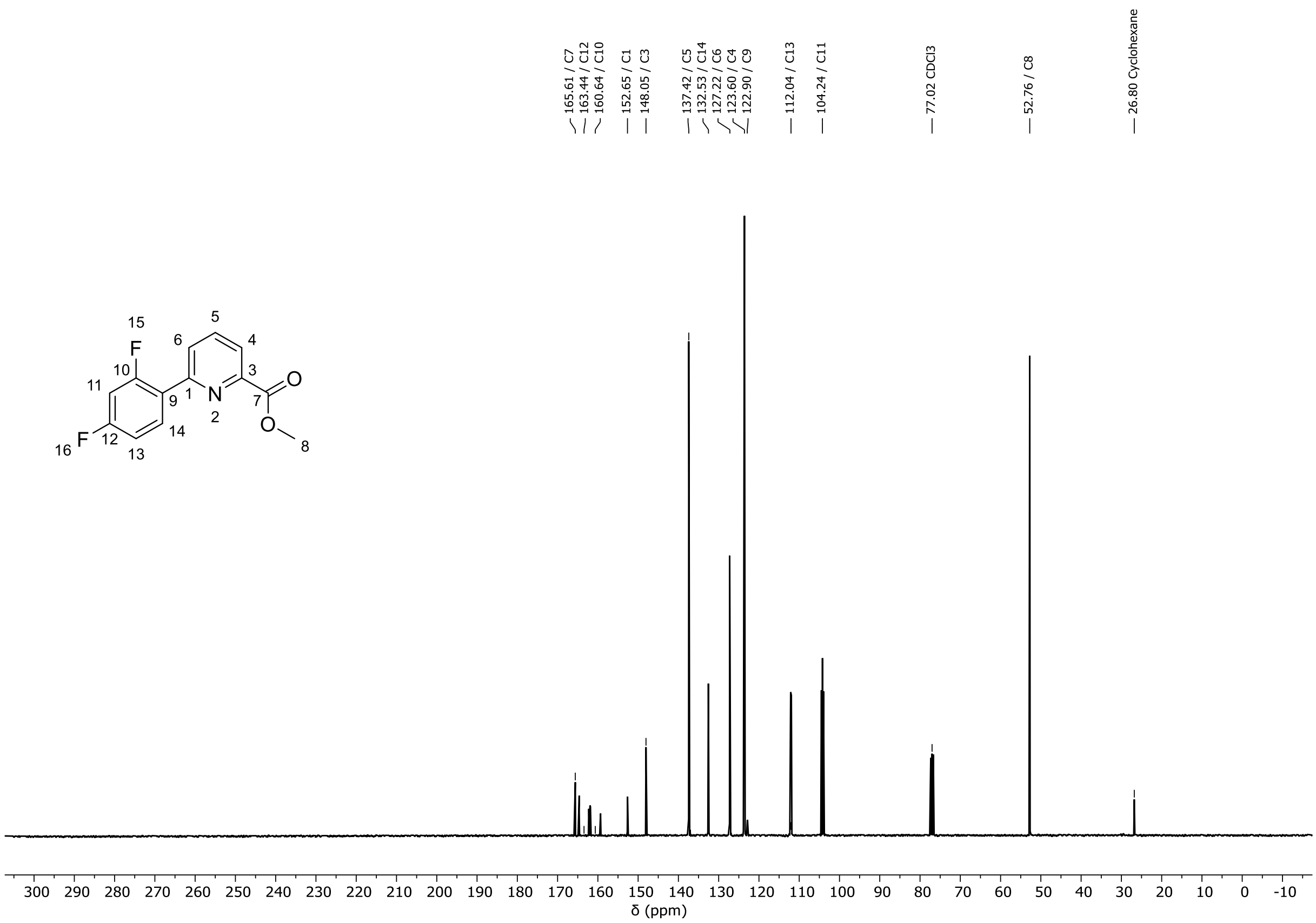

Figure S-28. $^{13}C\{^{1}H\}$-NMR spectrum (101 MHz, $CDCl_3$) of **3**.

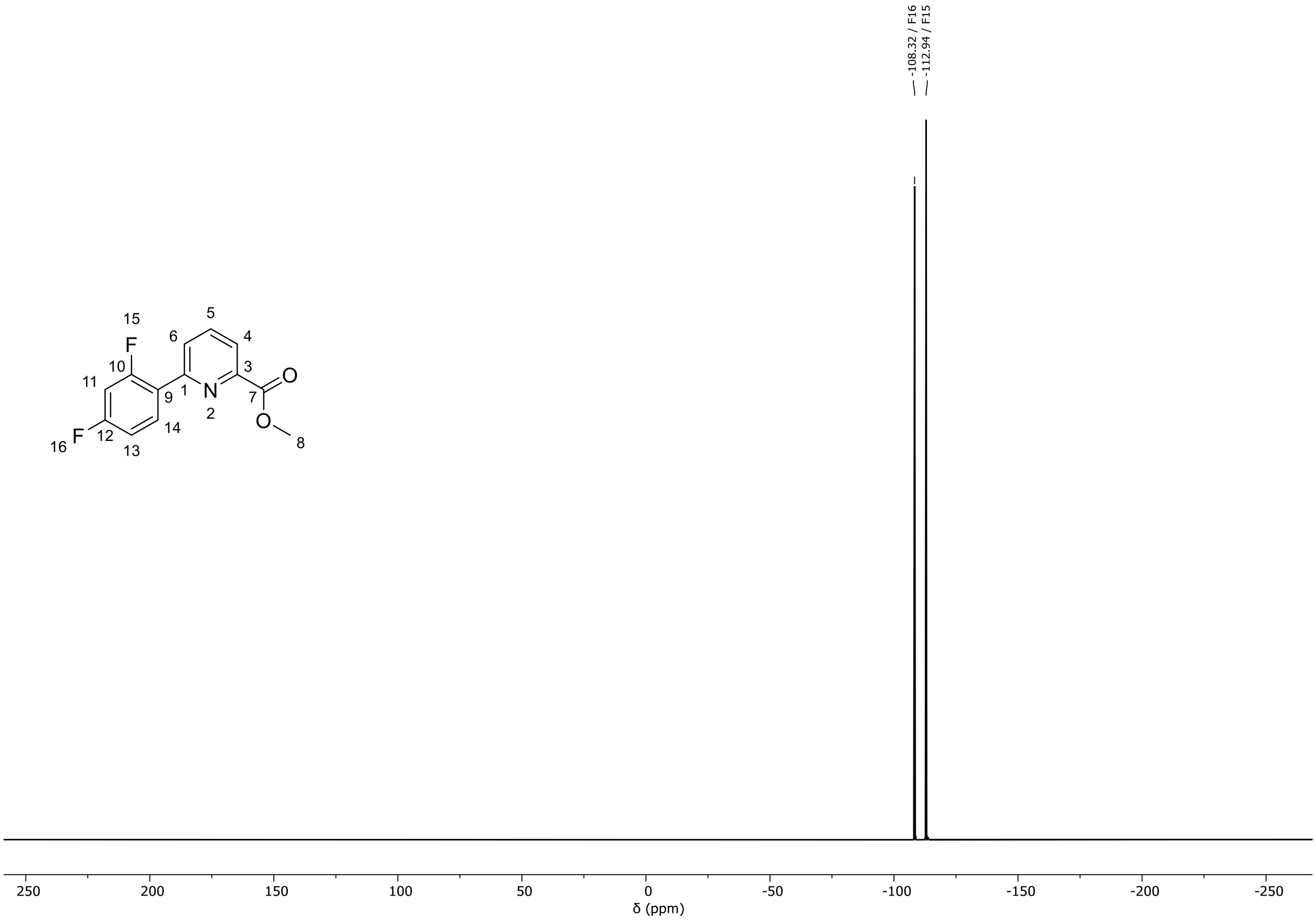

Figure S-29. $^{19}F\{^{1}H\}$-NMR spectrum (376 MHz, $CDCl_3$) of **3**.

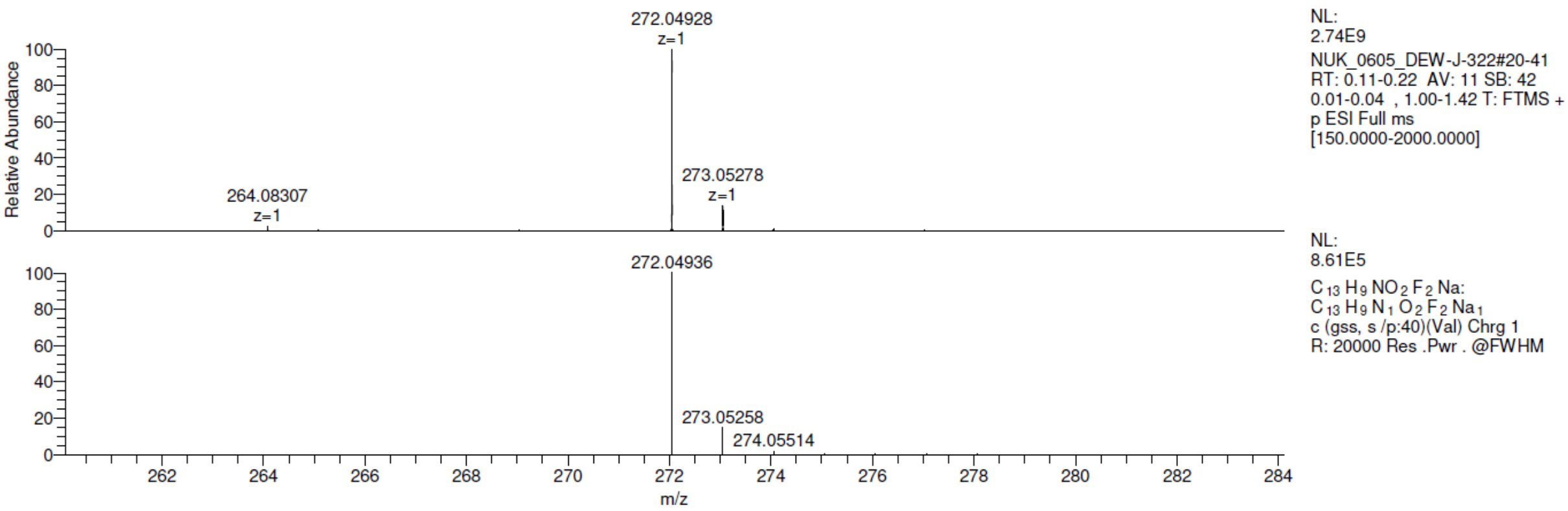

Figure S-30. Mass spectrum of **3** (MeOH). Additional simulation of the $[3+Na]^+$ adduct.

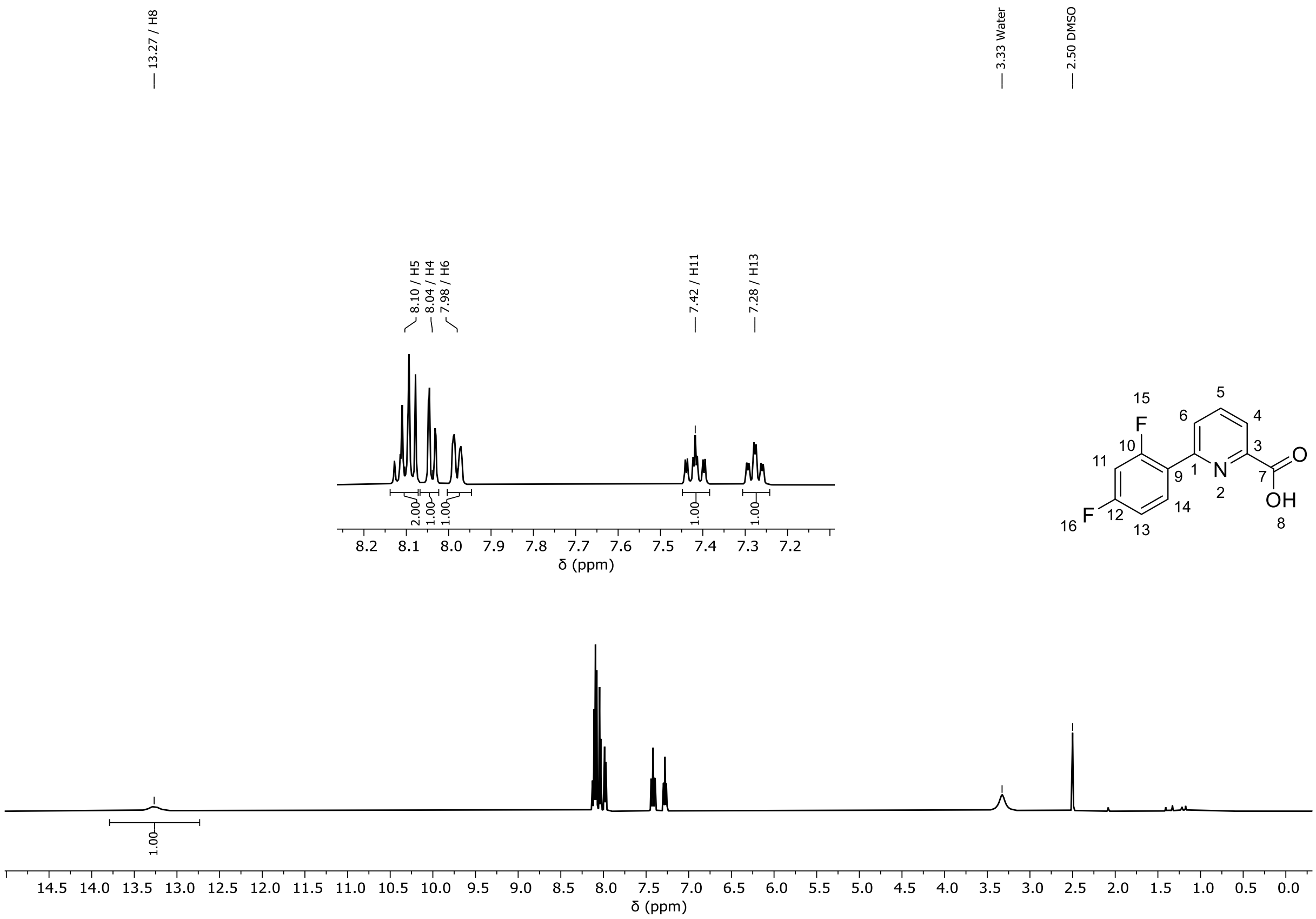

Figure S -31. ^{1}H-NMR spectrum (500 MHz, DMSO-d_6) of **L$_{DiF}$**.

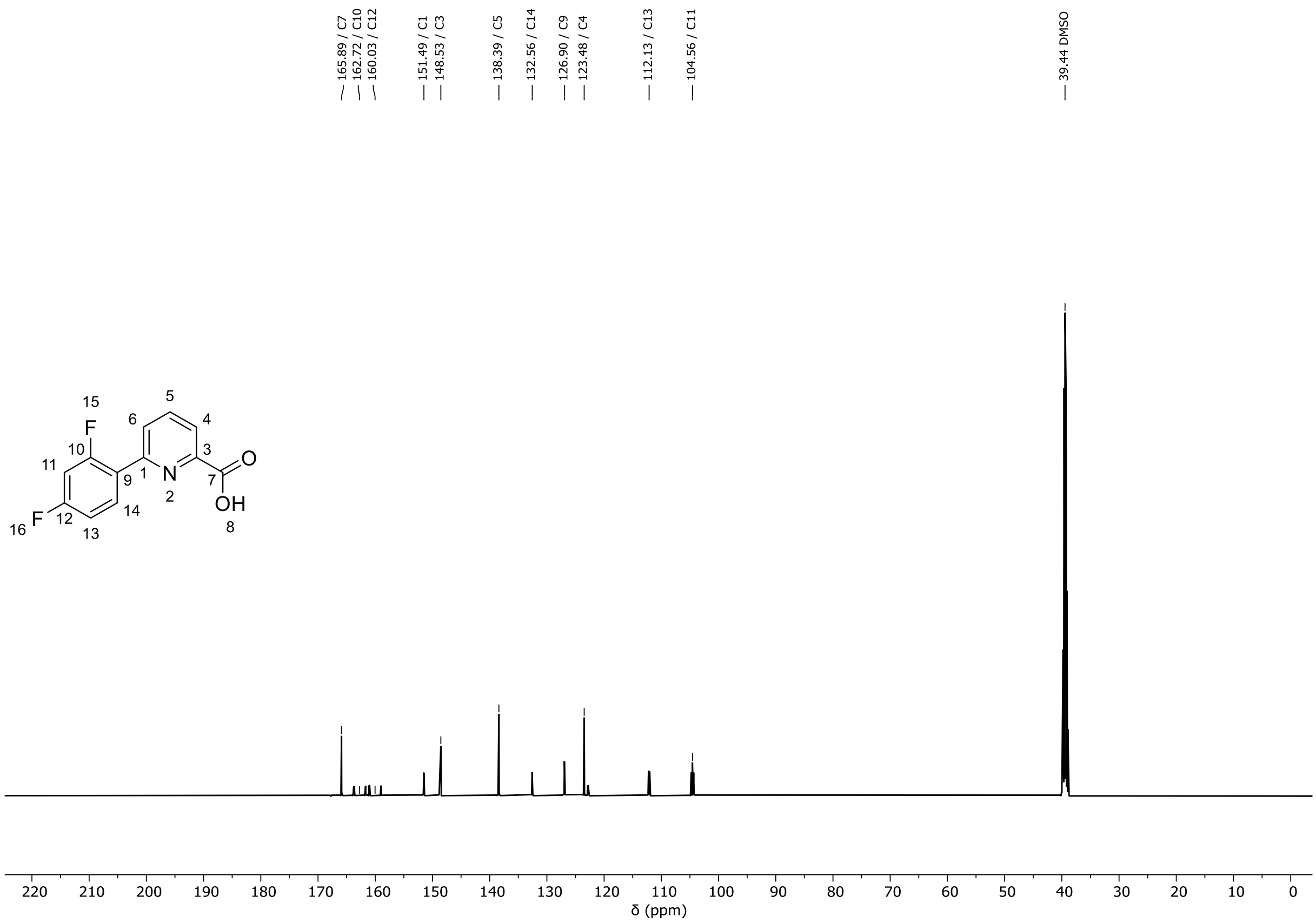

Figure S-32. ^{13}C{^{1}H}-NMR spectrum (126 MHz, DMSO-d_6) of **L$_{DiF}$**.

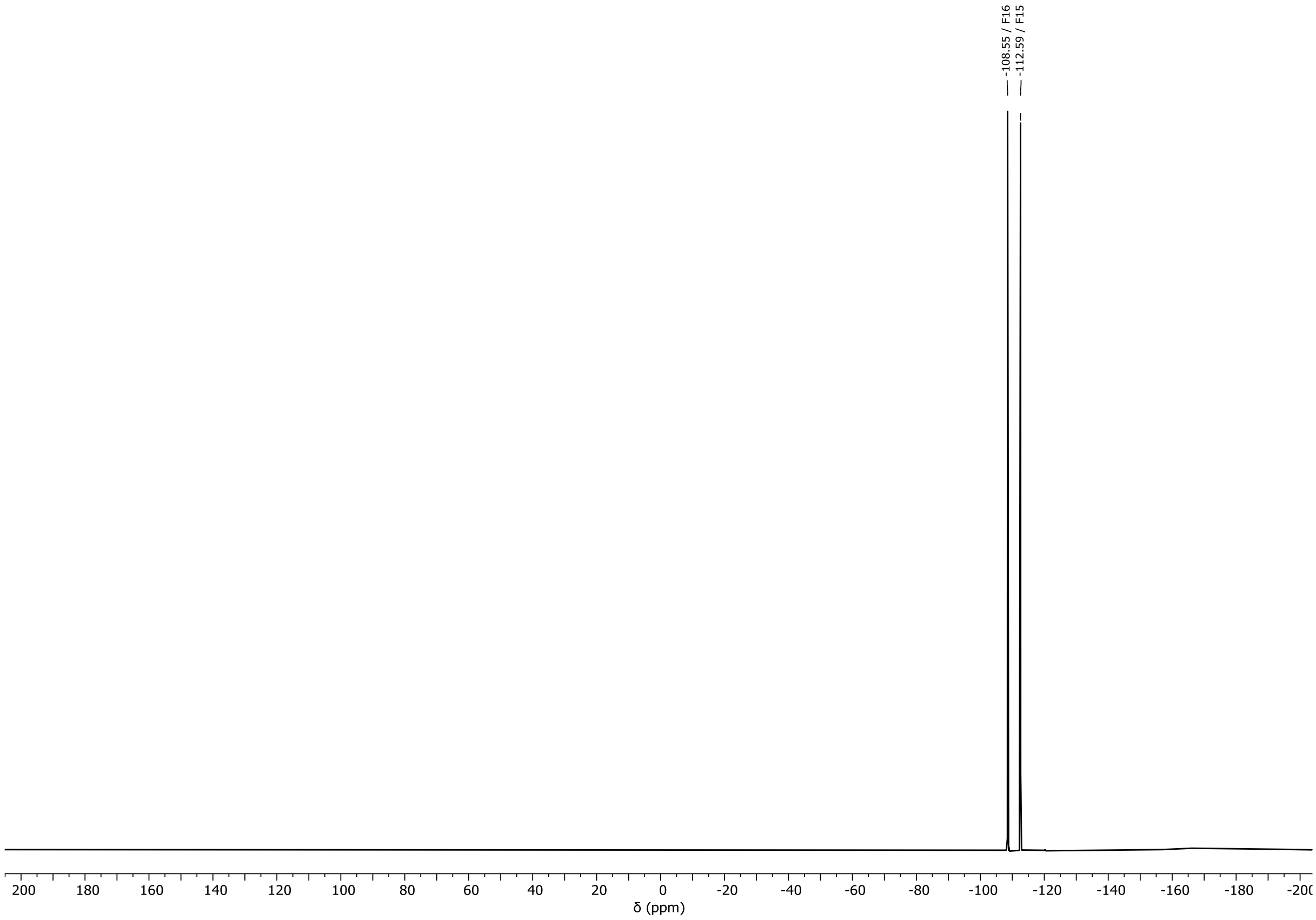

Figure S 33. $^{19}F\{^{1}H\}$-NMR spectrum (471 MHz, DMSO-d_6) of $\mathbf{L_{DiF}}$.

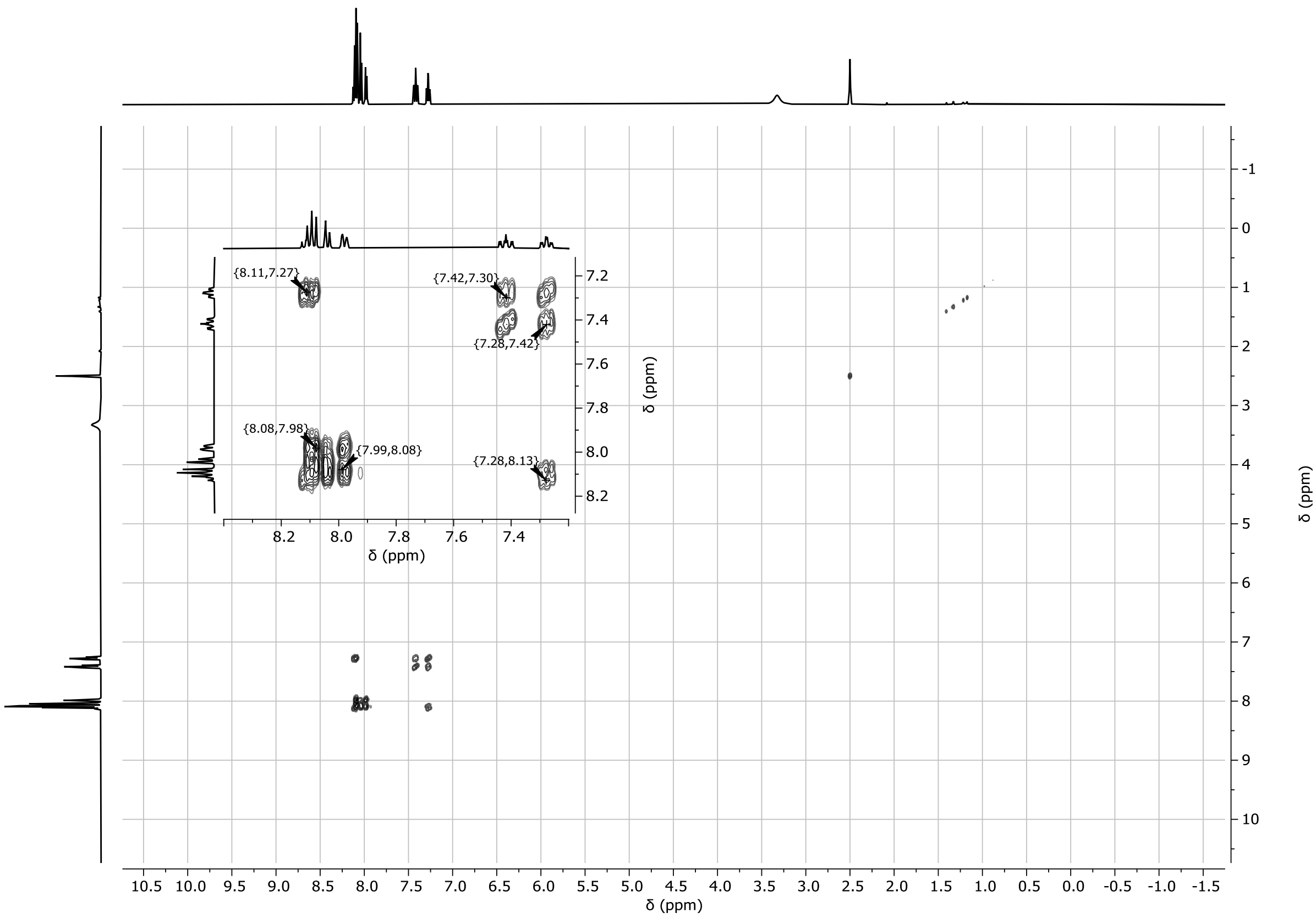

Figure S-34. HH-COSY-NMR spectrum (500 MHz, DMSO-d_6) of $\mathbf{L_{DiF}}$.

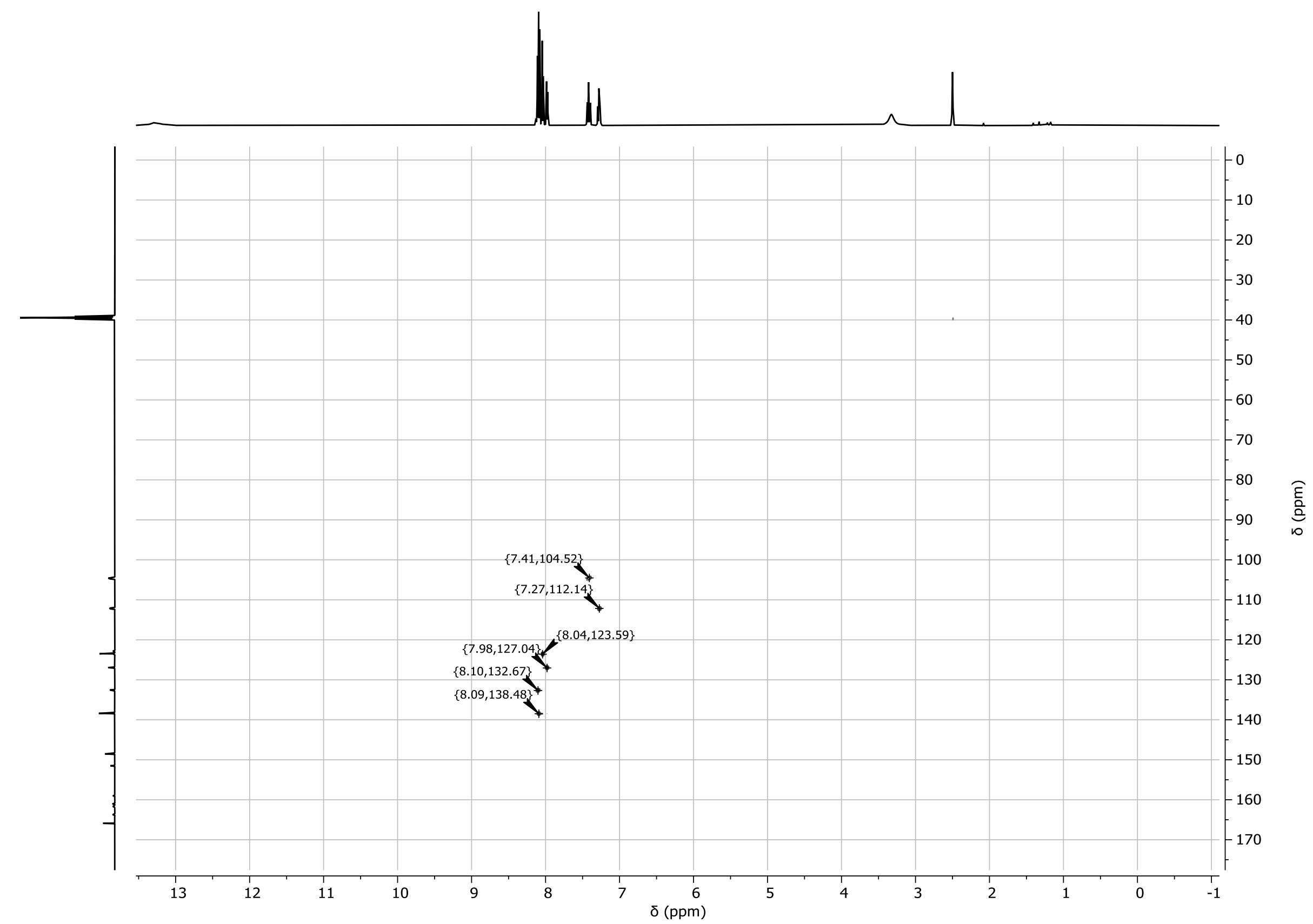

Figure S-35. HC-HSQC-NMR spectrum (126 MHz (^{13}C), 500 MHz (^{1}H), DMSO-d_6) of $\mathbf{L_{DiF}}$.

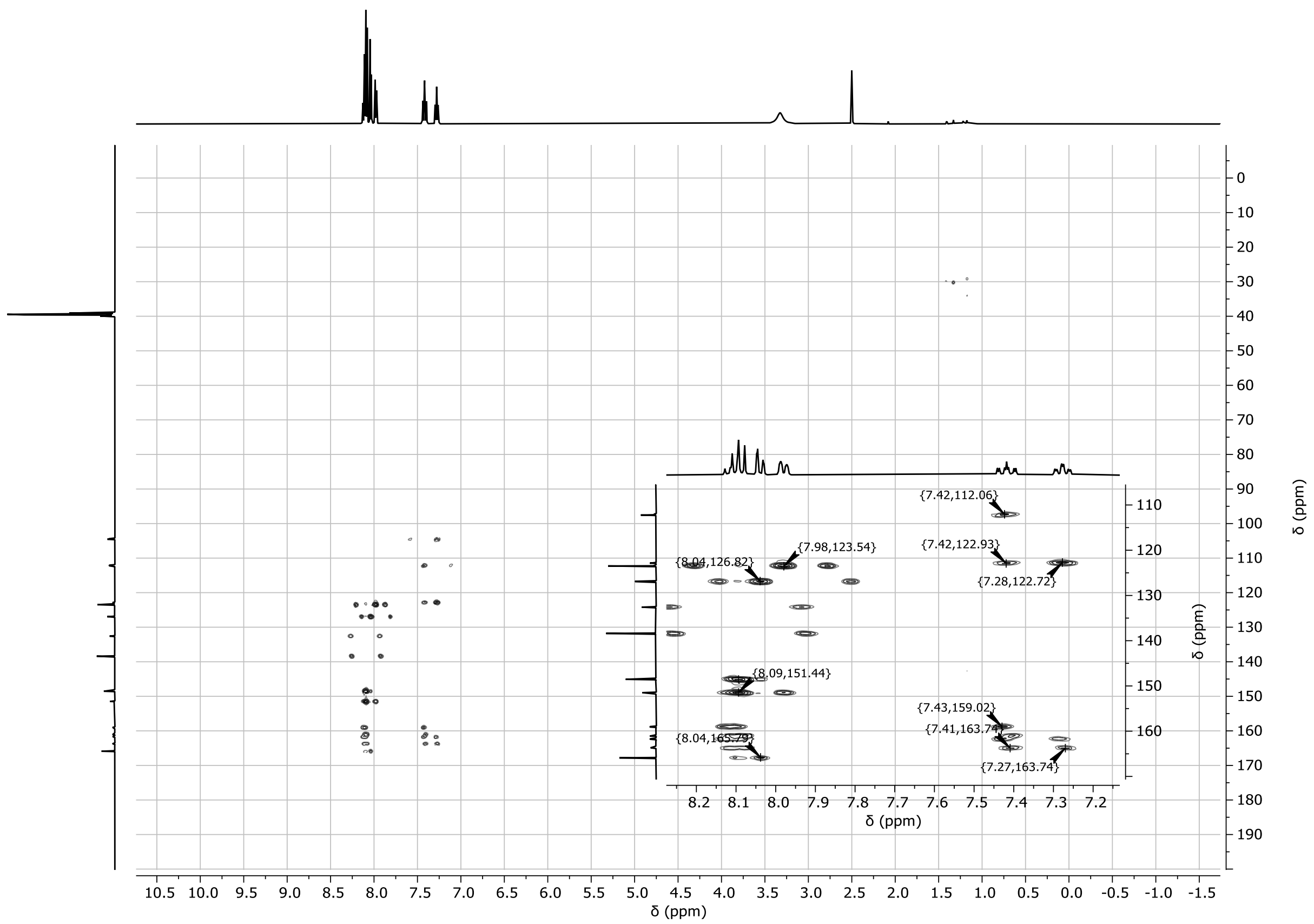

Figure S-36. HC-HMBC-NMR spectrum (126 MHz (^{13}C), 500 MHz (^{1}H), DMSO-d_6) of $\mathbf{L_{DiF}}$.

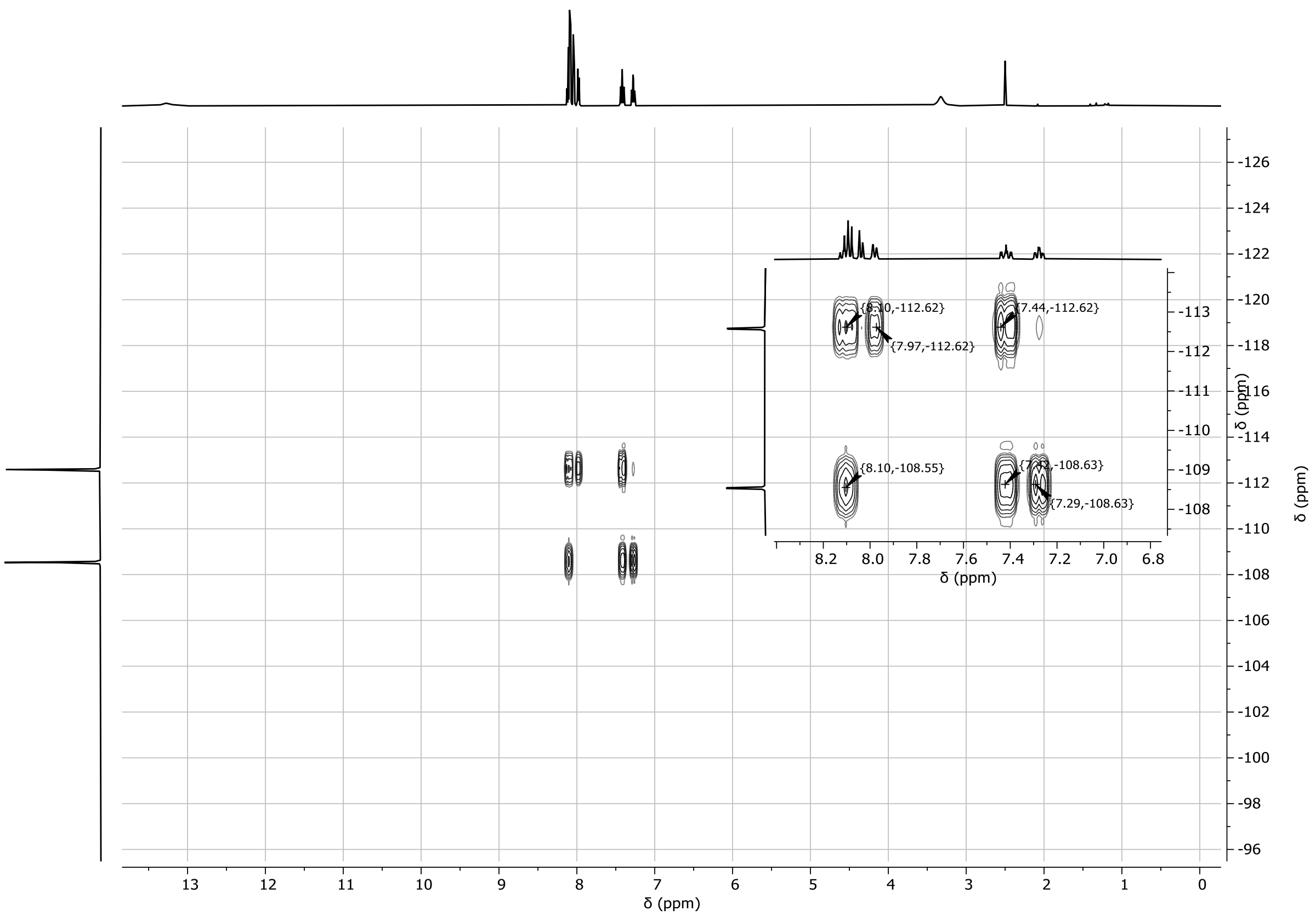

Figure S-37. HF-COSY-NMR spectrum (500 MHz (^{1}H), 471 MHz (^{19}F), DMSO-d_6) of $\mathbf{L_{DiF}}$.

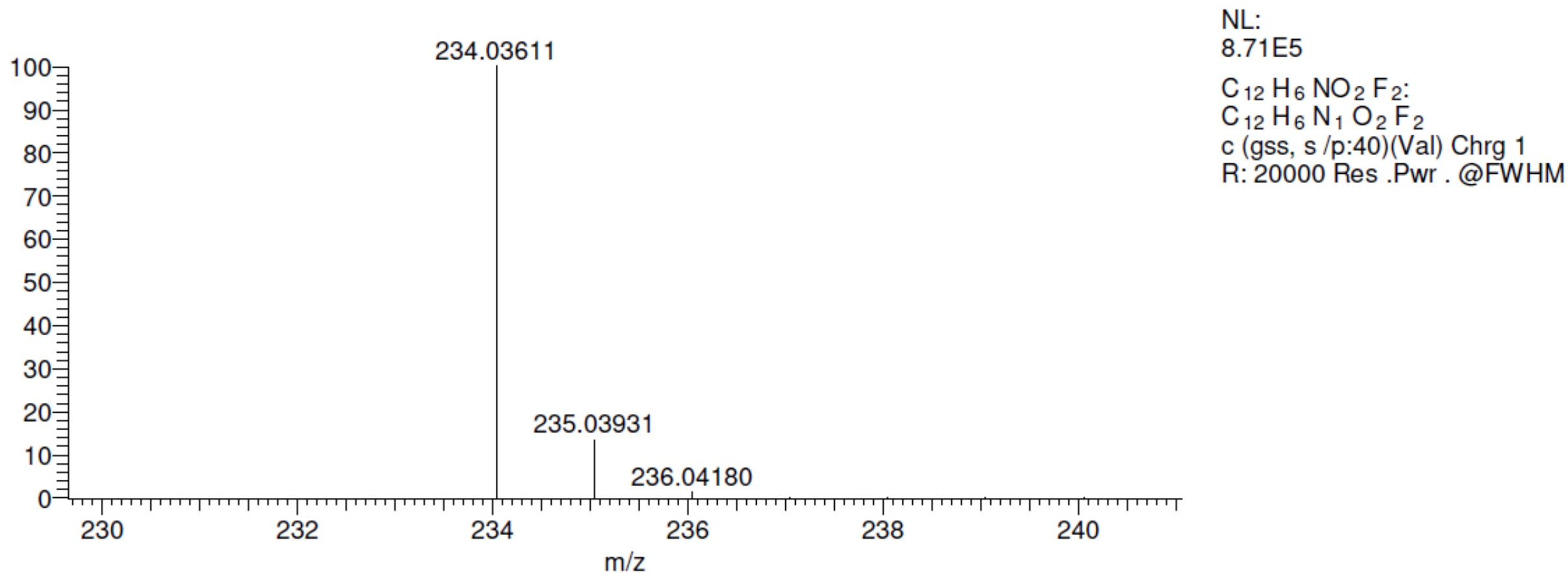

Figure S-38. Mass spectrum of $\mathbf{L_{DiF}}$ (MeOH). Shown is the adduct [$\mathbf{L_{DiF}}$+H]$^{+}$.

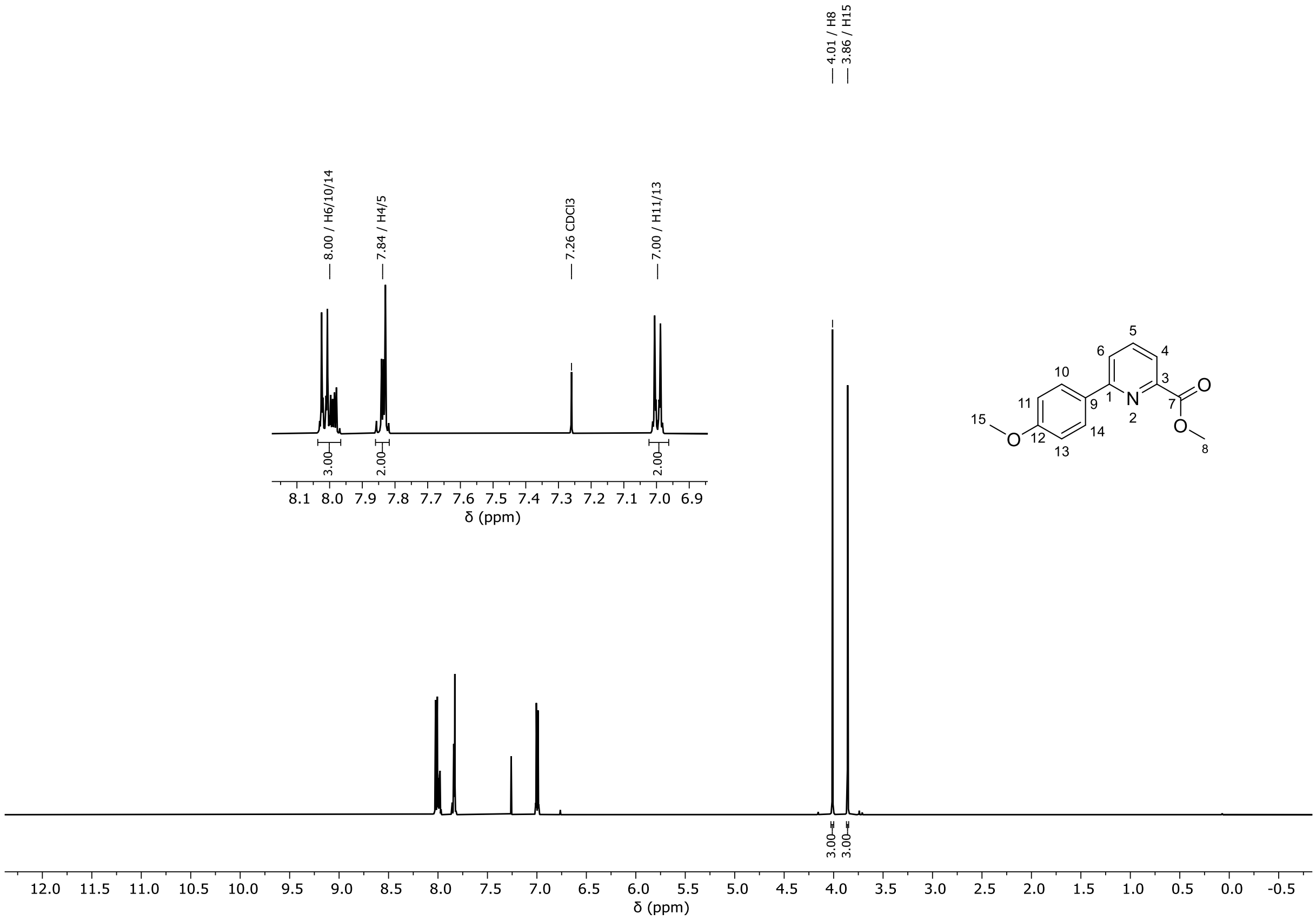

Figure S-39. ^{1}H-NMR spectrum (400 MHz, $CDCl_3$) of **4**.

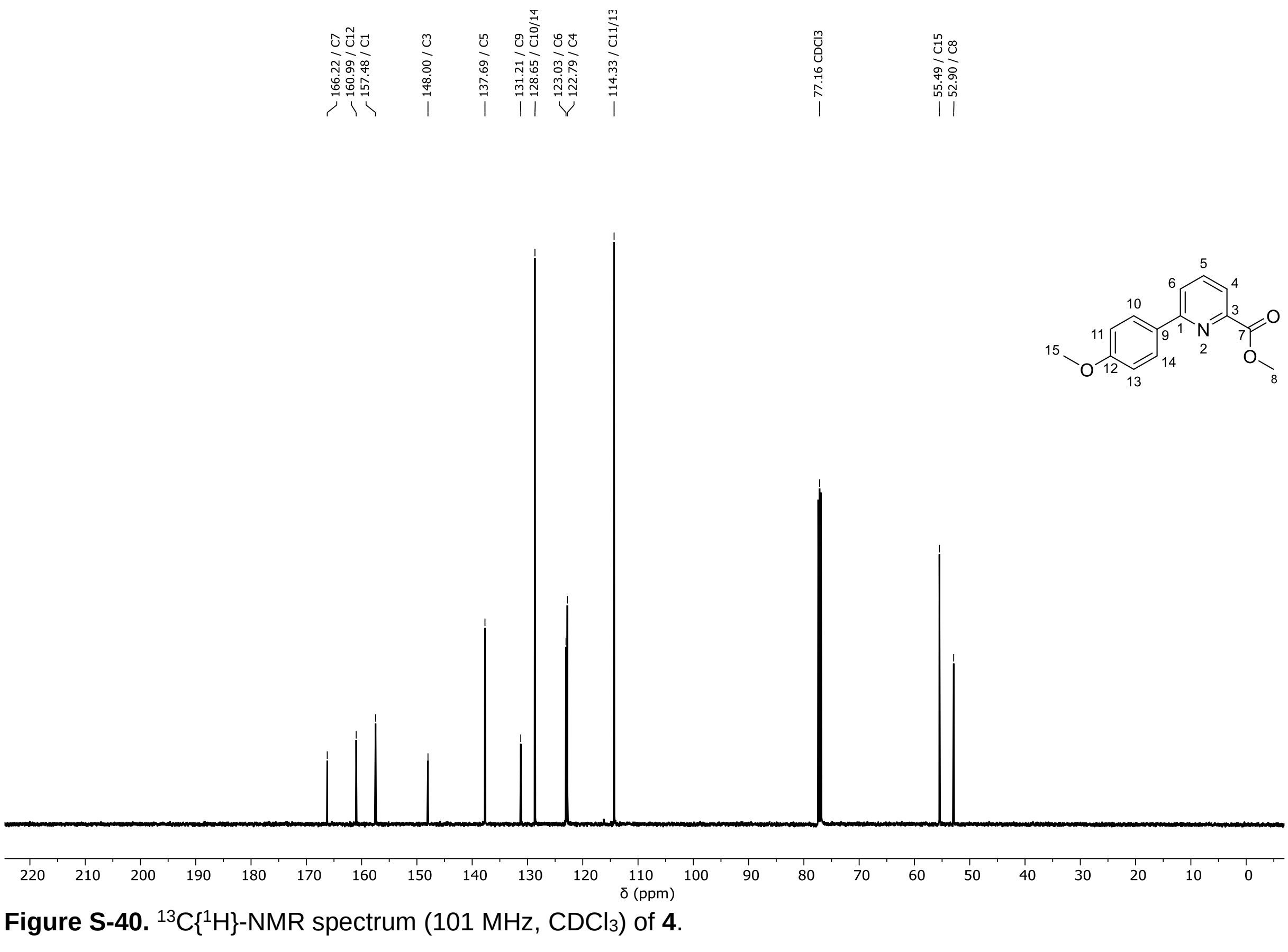

Figure S-40. $^{13}C\{^{1}H\}$-NMR spectrum (101 MHz, $CDCl_3$) of **4**.

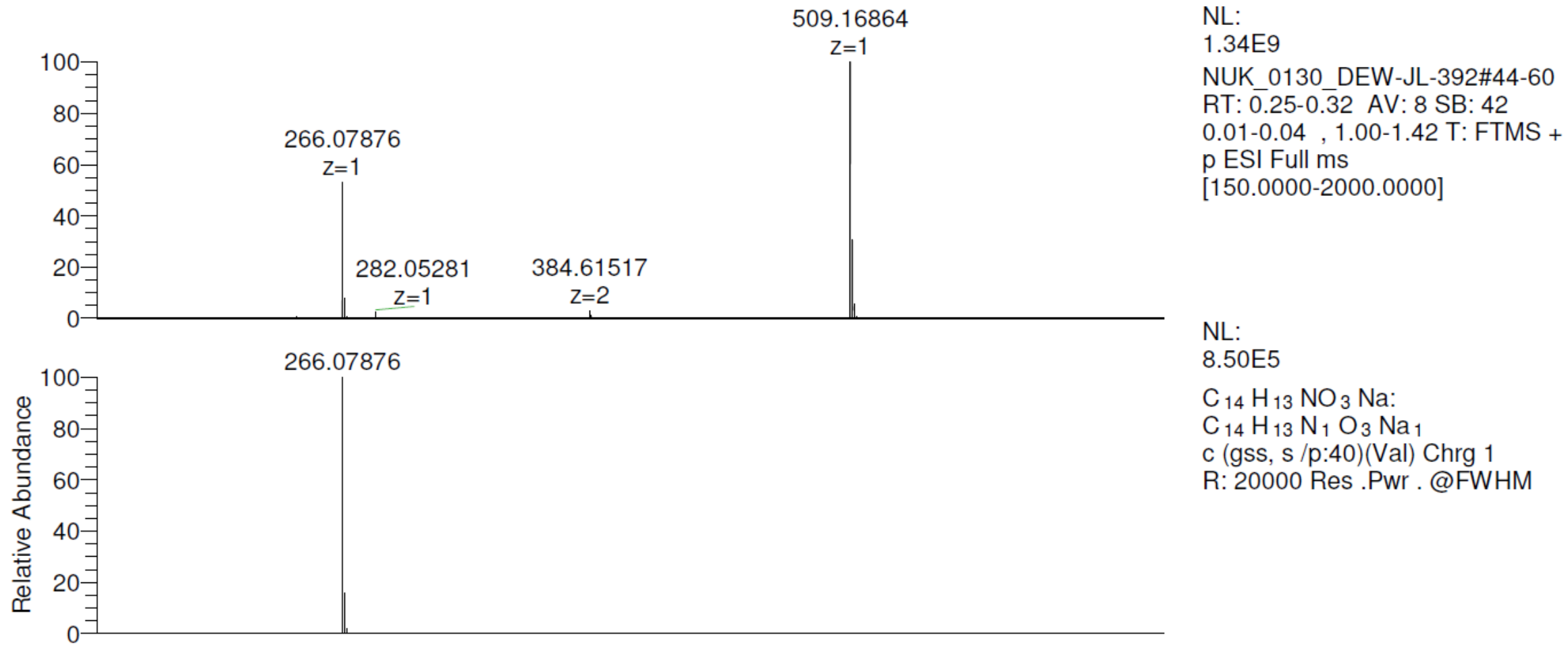

Figure S-41. Mass spectrum of **4** (MeOH). Additional simulation of the [**4**+Na]+ adduct.

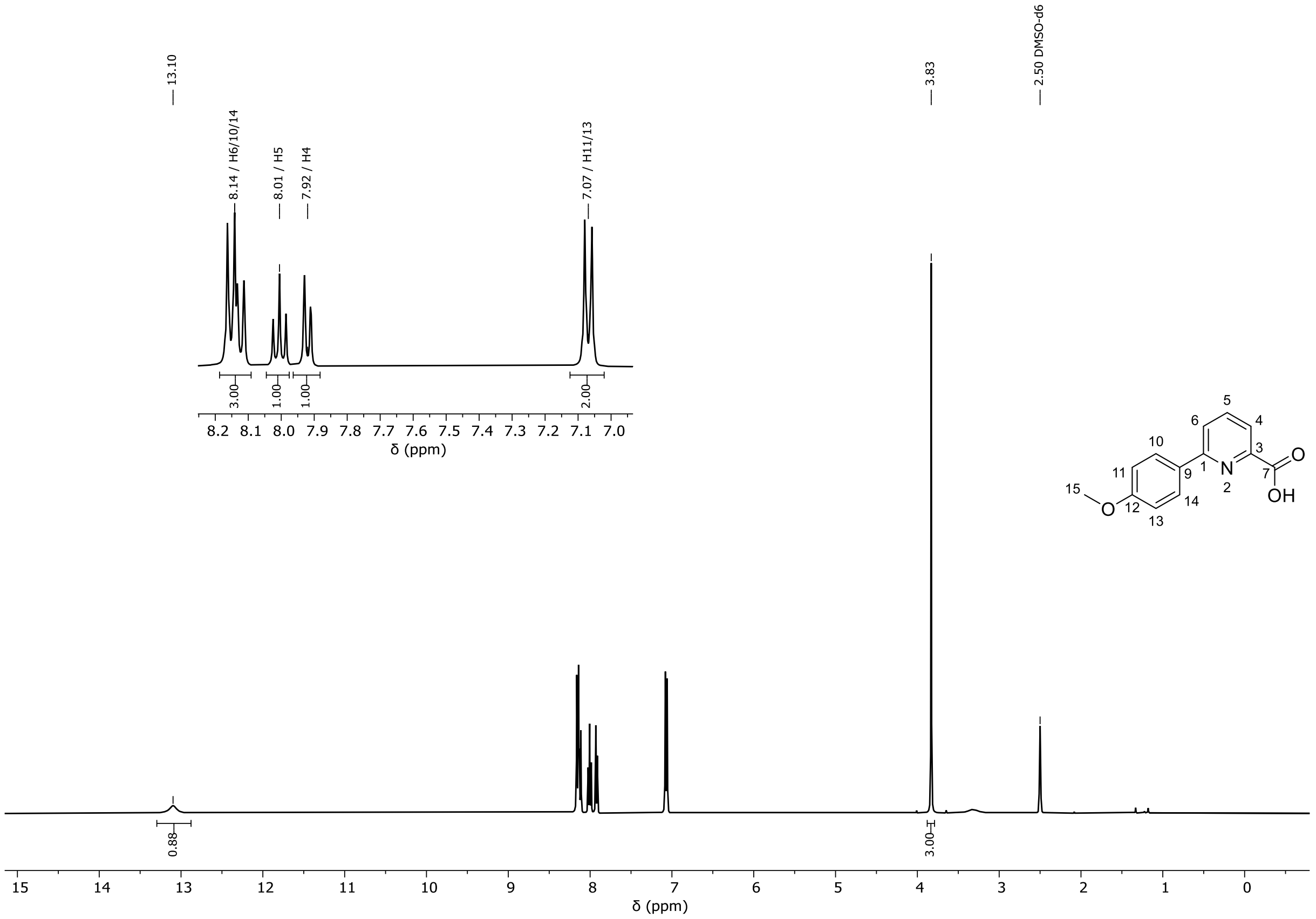

Figure S-42. ^{1}H-NMR spectrum (400 MHz, DMSO-d_6) of **L$_{mOMe}$**.

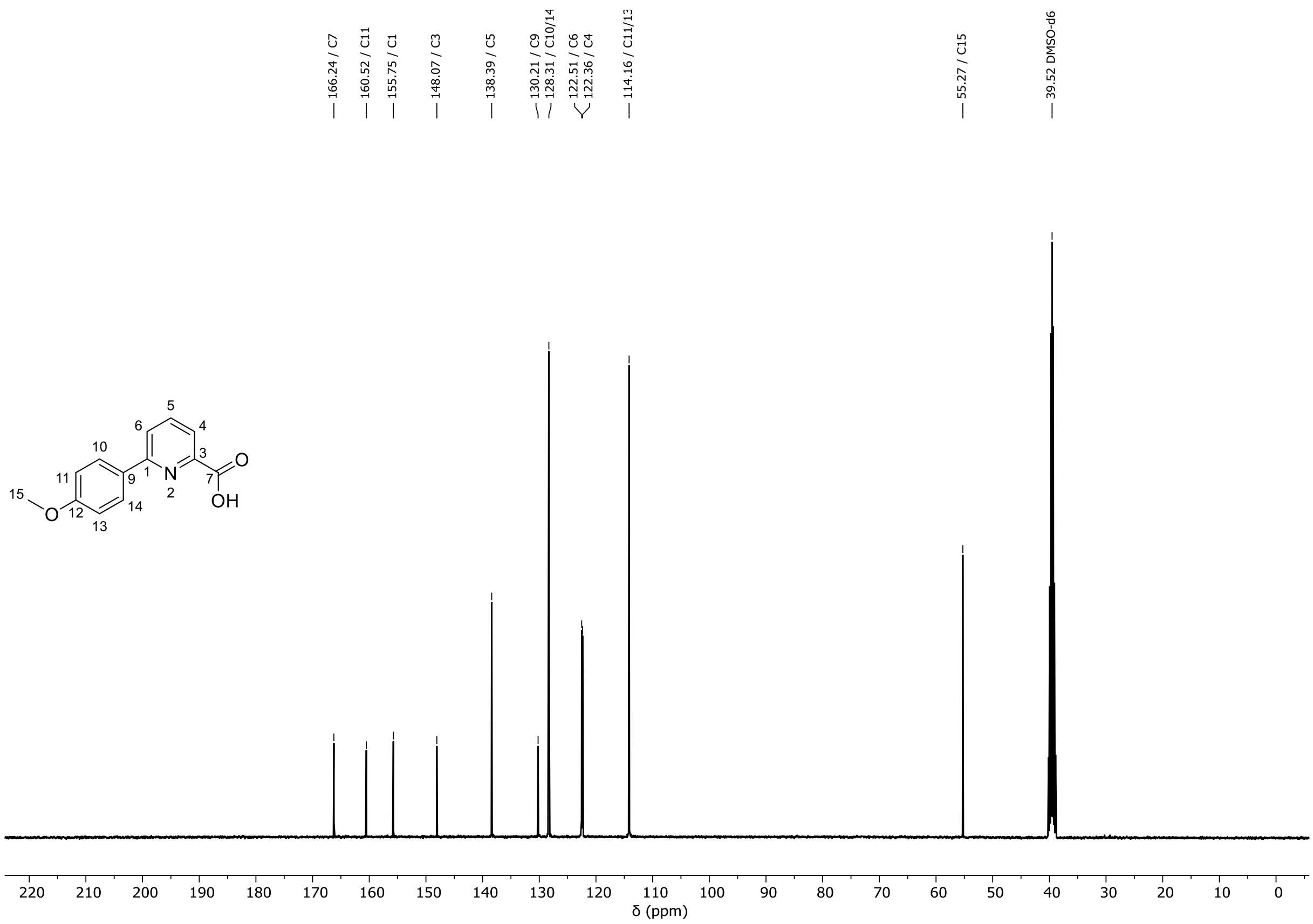

Figure S-43. $^{13}C\{^{1}H\}$-NMR spectrum (101 MHz, DMSO-d_6) of $\mathbf{L_{mOMe}}$.

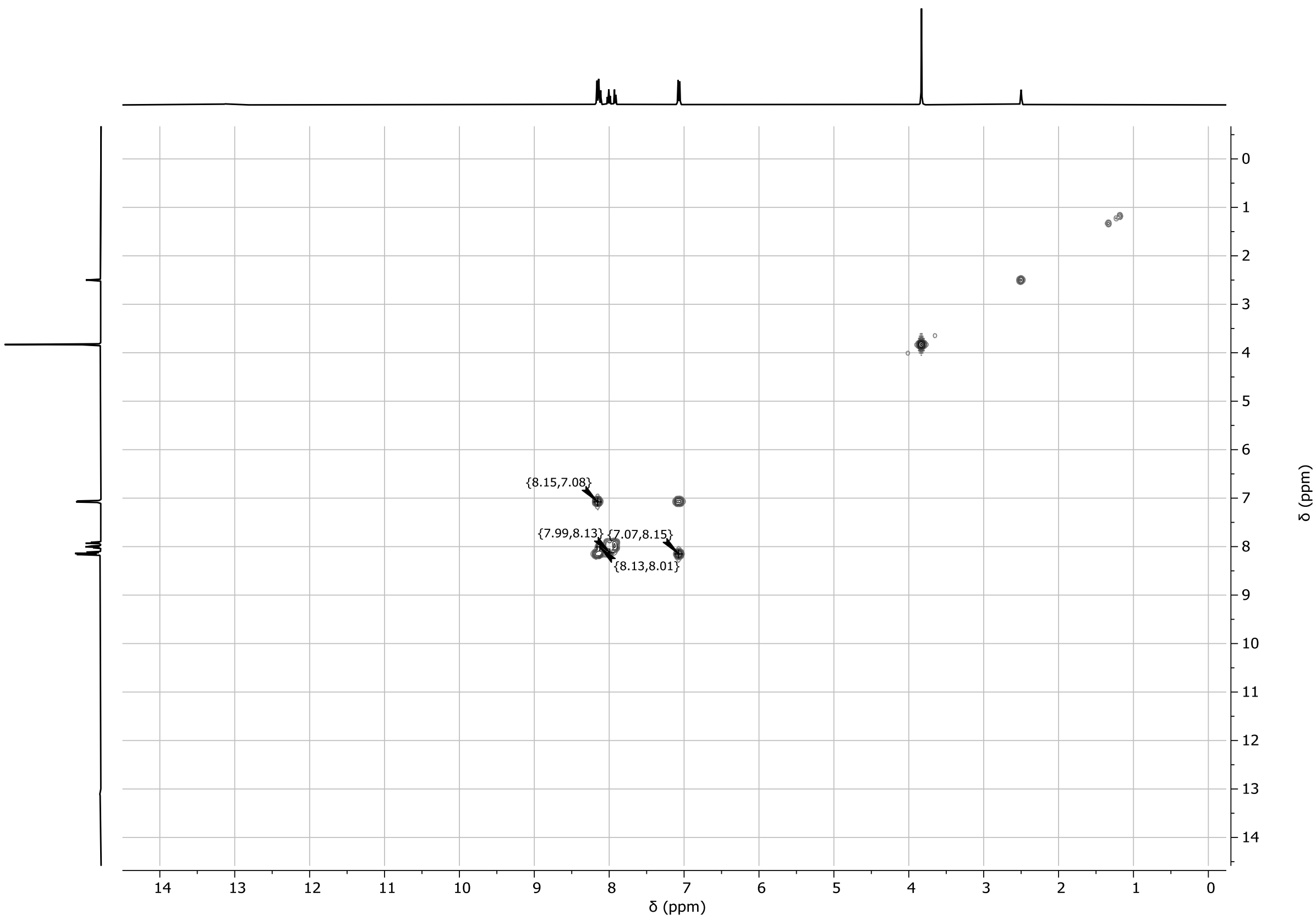

Figure S-44. HH-COSY-NMR spectrum (400 MHz, DMSO-d_6) of $\mathbf{L_{mOMe}}$.

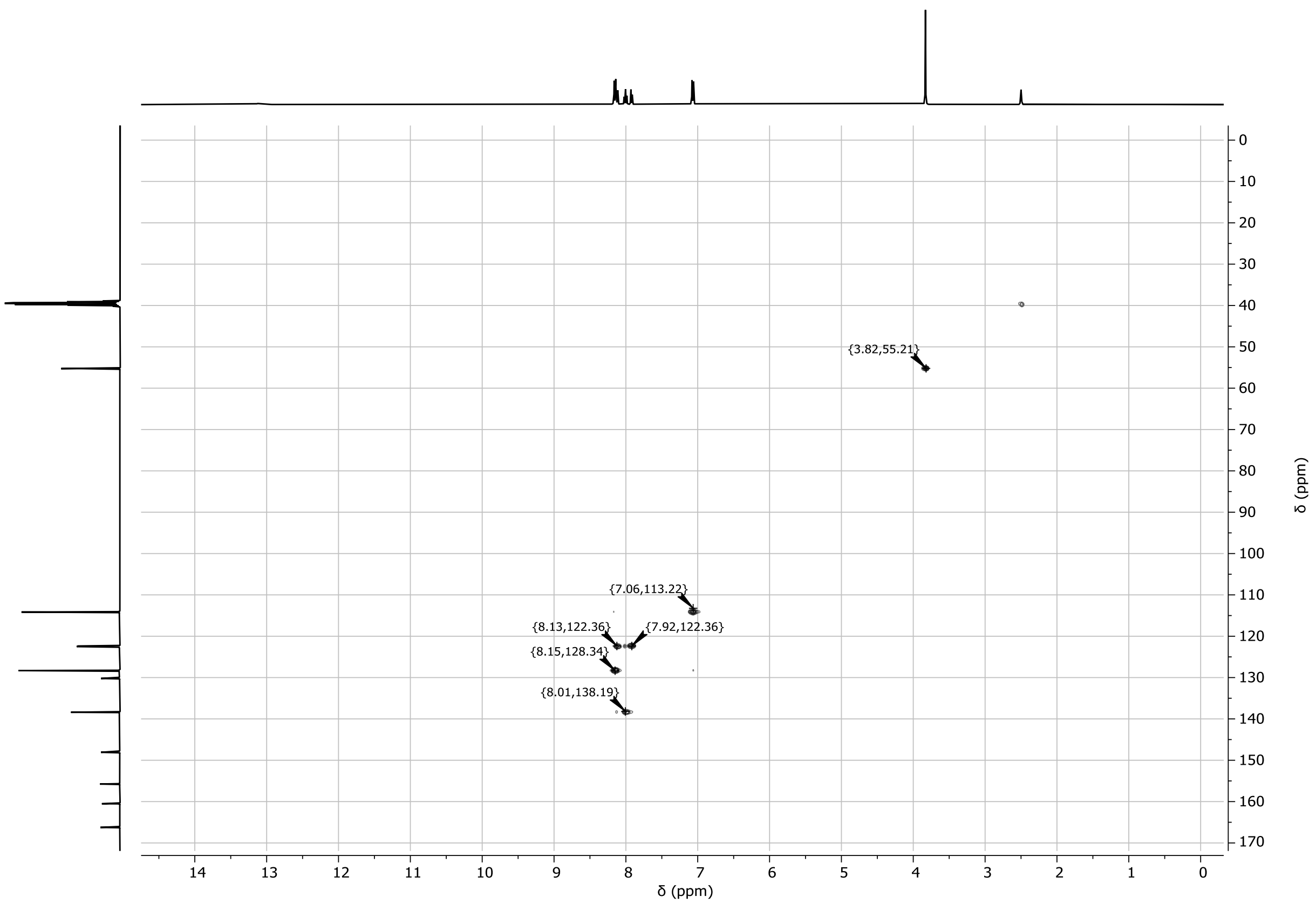

Figure S-45. HC-HSQC-NMR spectrum (101 MHz (^{13}C), 400 MHz (^{1}H), DMSO-d_6) of $\mathbf{L_{mOMe}}$.

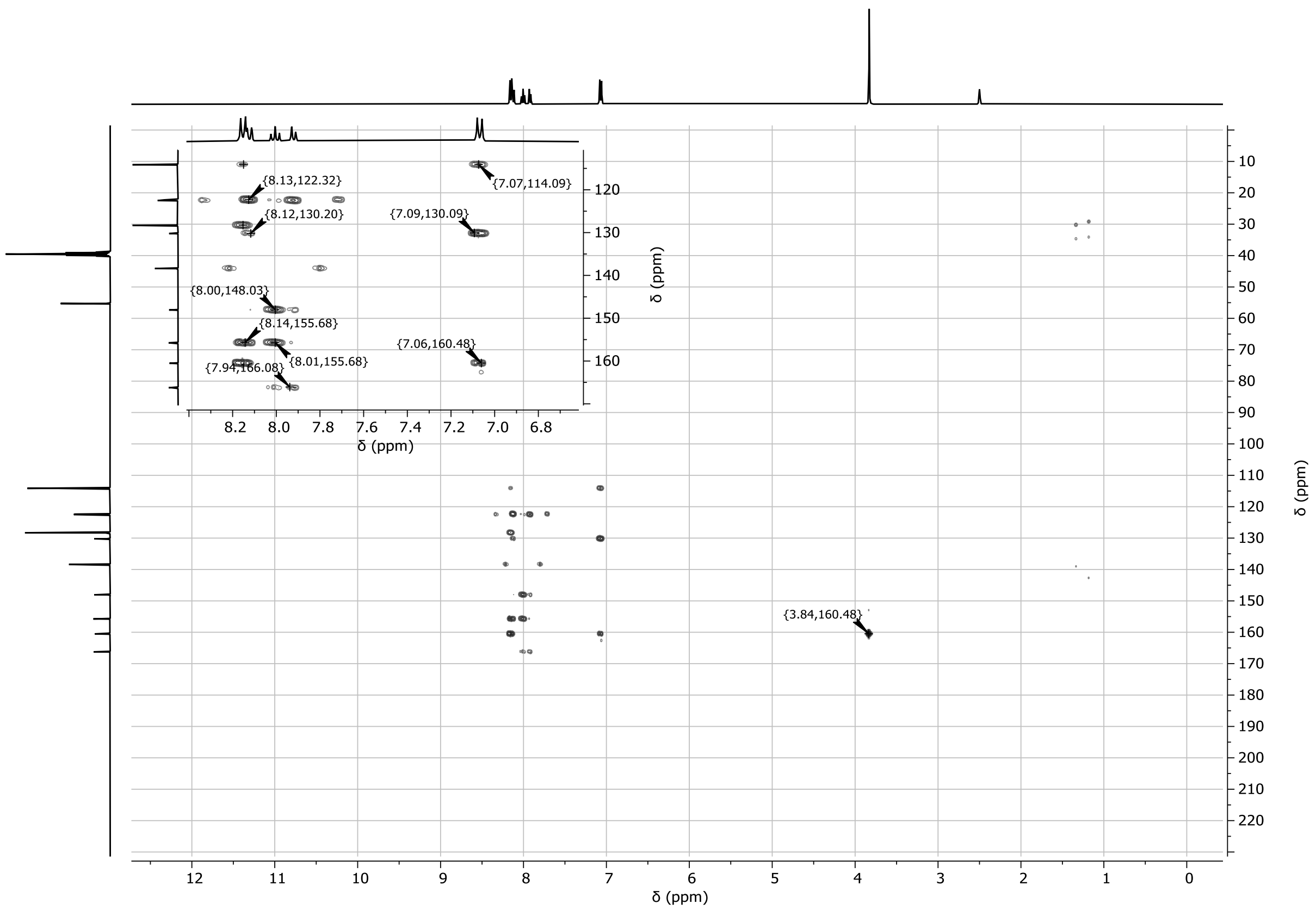

Figure S-46. HC-HMBC-NMR spectrum (101 MHz (^{13}C), 400 MHz (^{1}H), DMSO-d_6) of $\mathbf{L_{mOMe}}$.

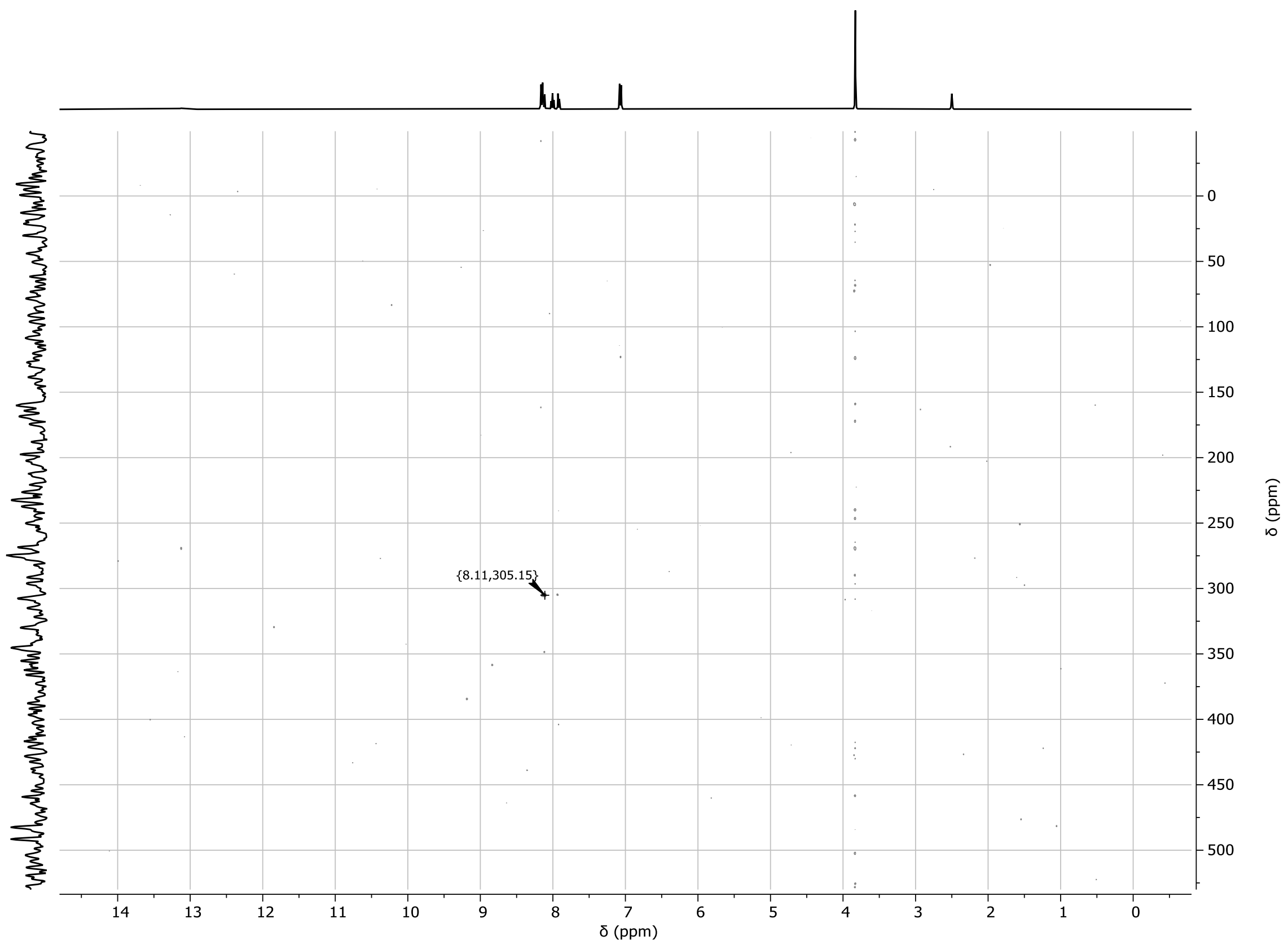

Figure S-47. HN-HMBC-NMR spectrum (41 MHz (^{15}N, 400 MHz (^{1}H), DMSO-d_6) of $\mathbf{L_{mOMe}}$.

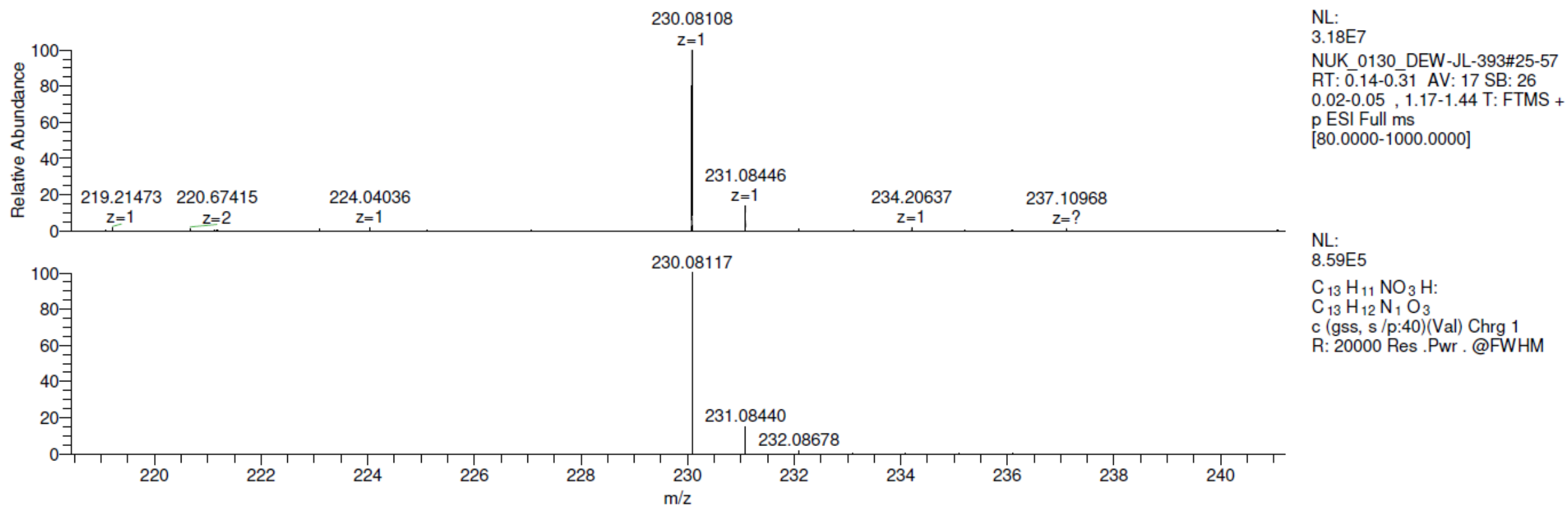

Figure S-48. Mass spectrum of $\mathbf{L_{mOMe}}$ (MeOH). Additional simulation of the [$\mathbf{L_{mOMe}}$+H]$^+$ adduct.

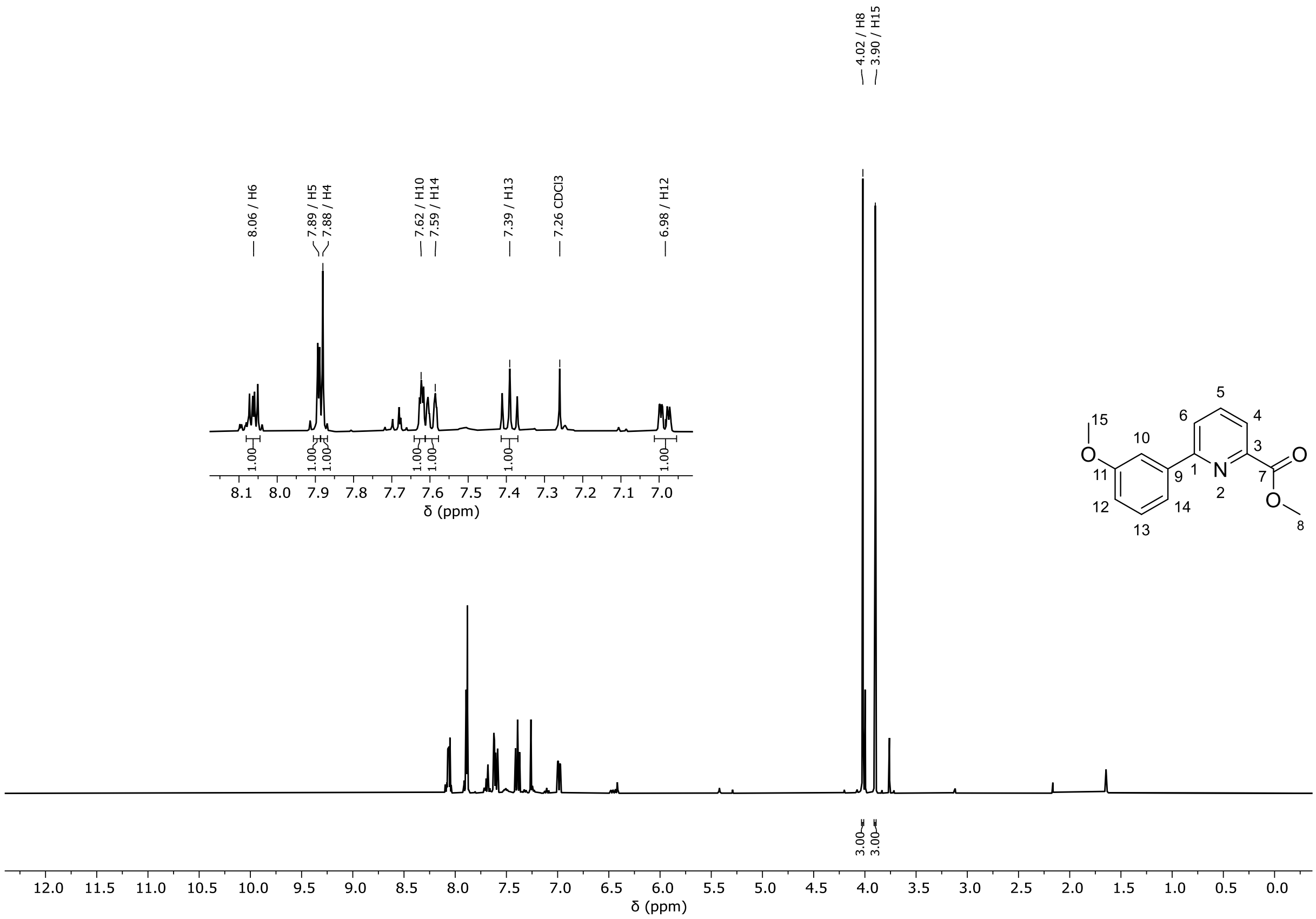

Figure S-49. ^{1}H-NMR spectrum (400 MHz, $CDCl_3$) of **5**.

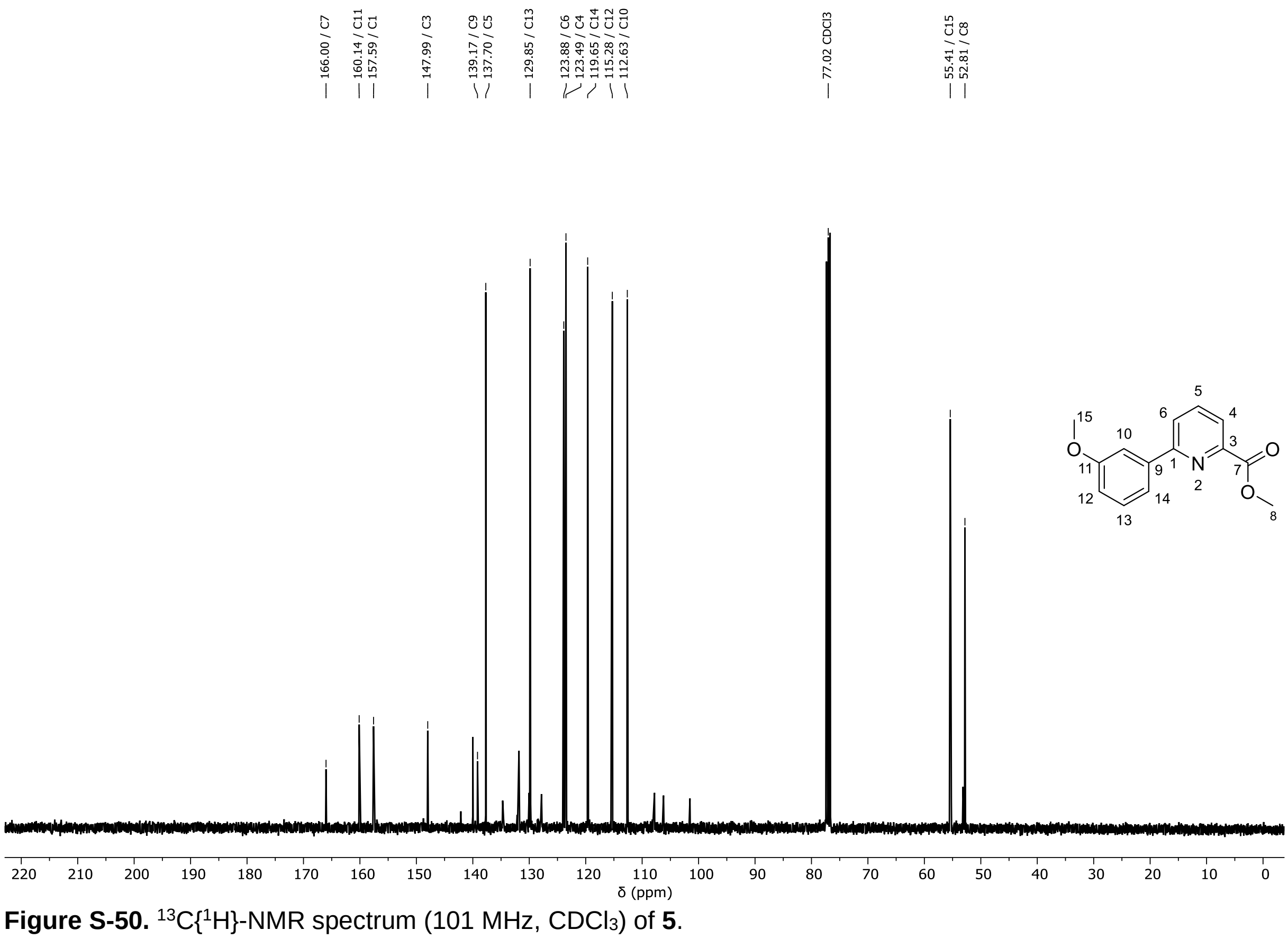

Figure S-50. $^{13}C\{^{1}H\}$-NMR spectrum (101 MHz, $CDCl_3$) of **5**.

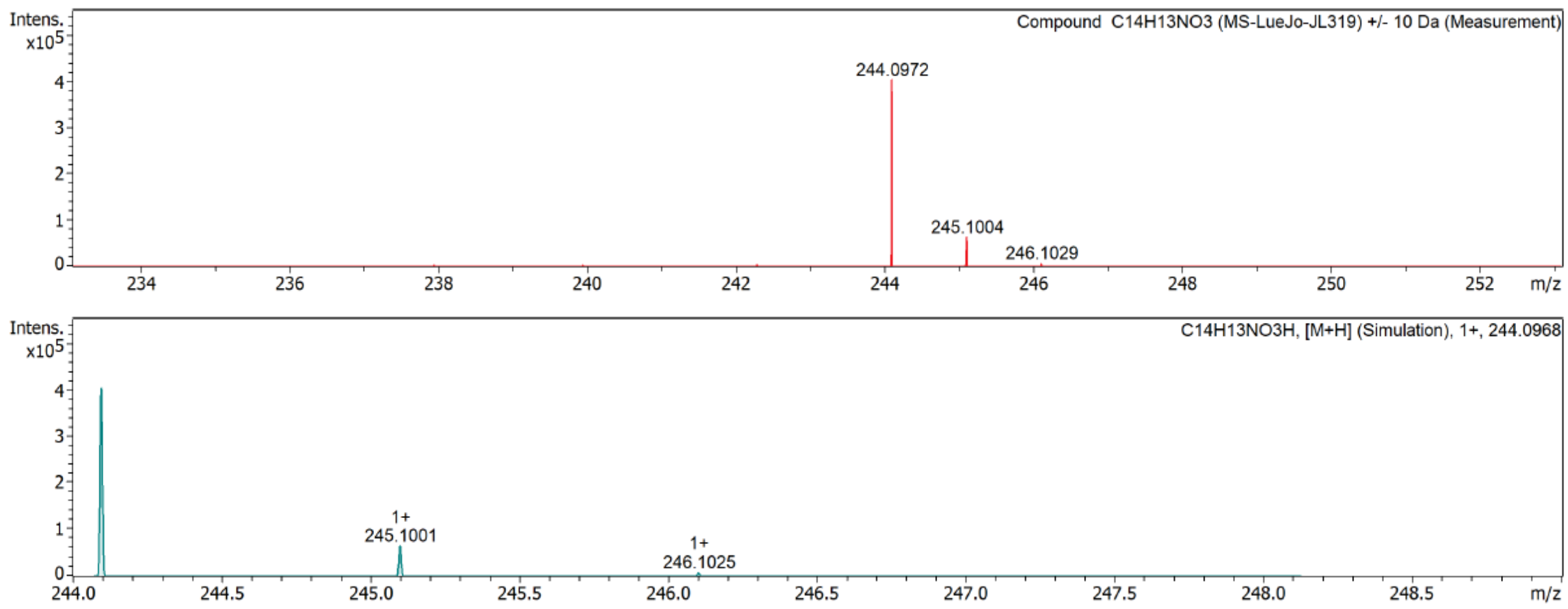

Figure S-51. Mass spectrum of **5** (MeOH). Additional simulation of the [**5**+H]+ adduct.

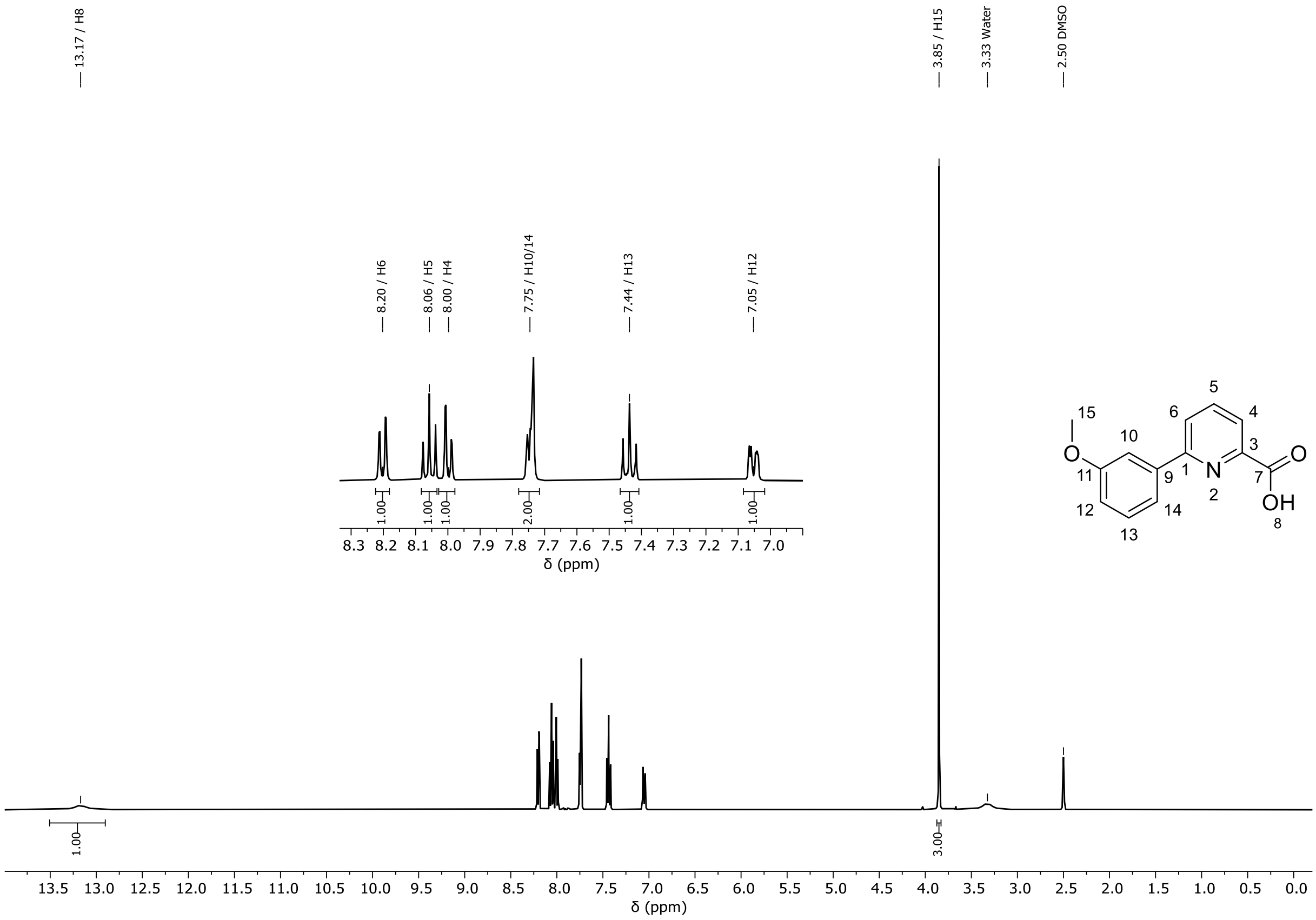

Figure S-52. ^{1}H-NMR spectrum (400 MHz, DMSO-d_6) of **L$_{pOMe}$**.

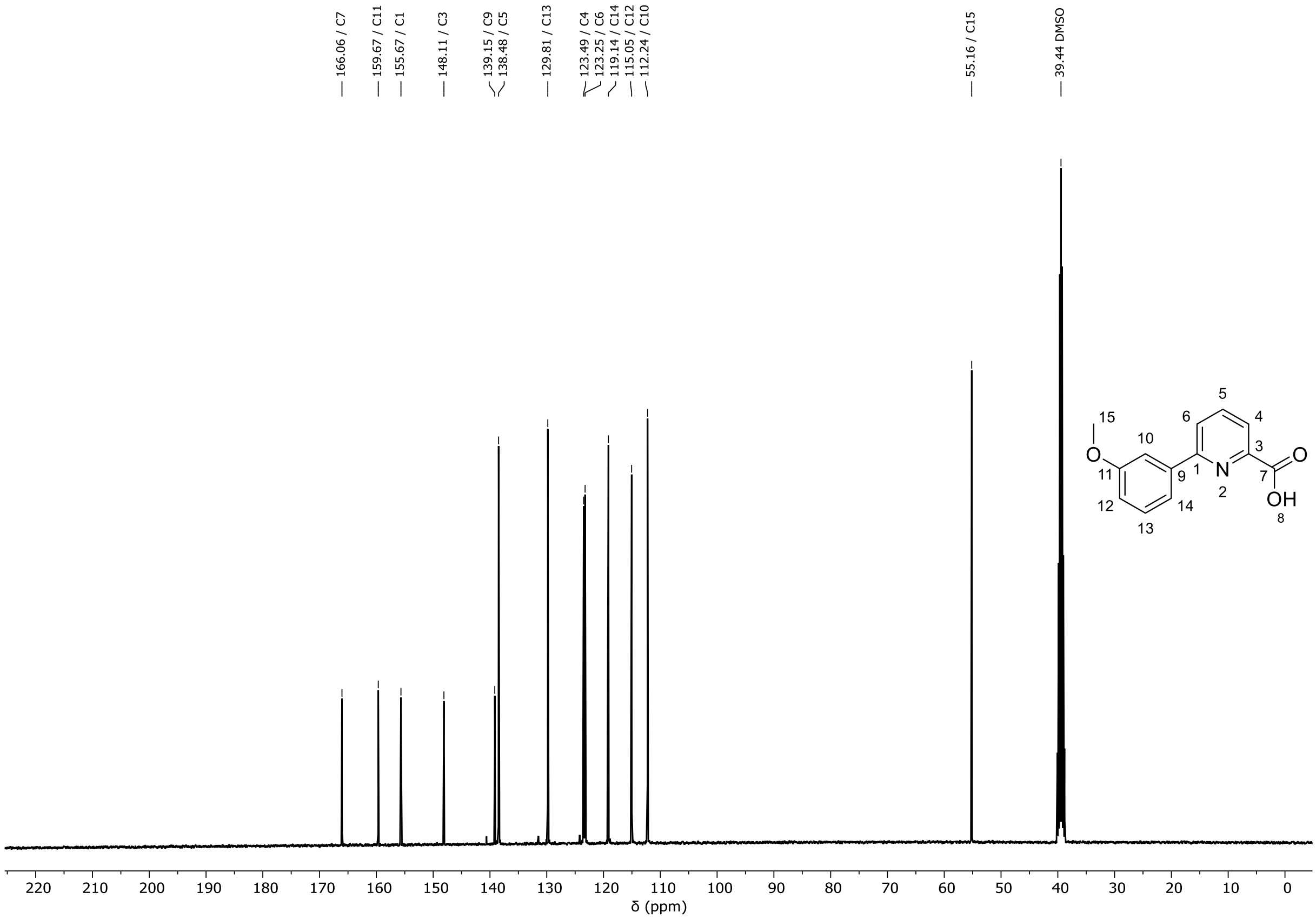

Figure S-53. $^{13}C\{^{1}H\}$-NMR spectrum (101 MHz, DMSO-d_6) of **L$_{pOMe}$**.

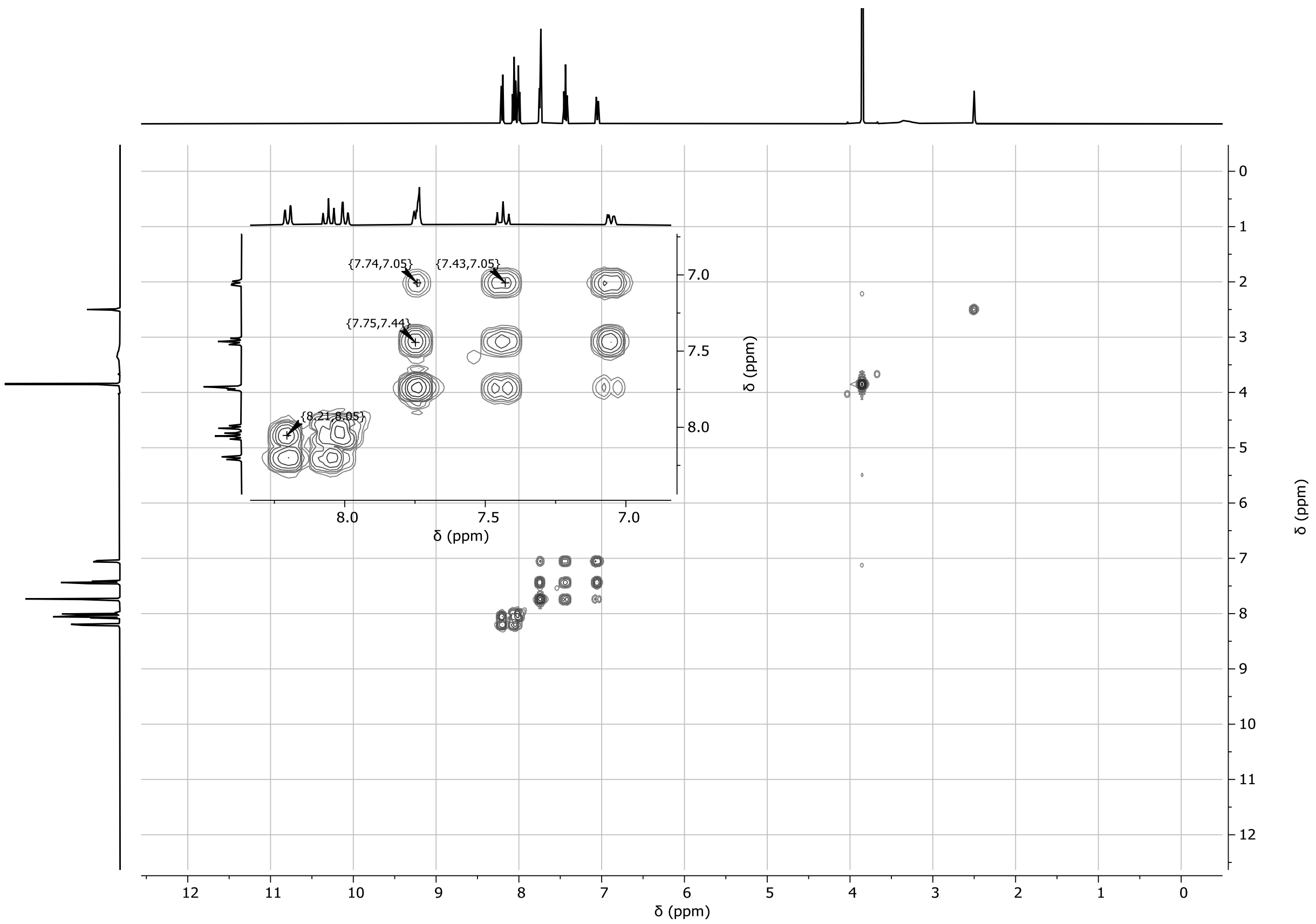

Figure S-54. HH-COSY-NMR spectrum (400 MHz, DMSO-d_6) of **L$_{pOMe}$**.

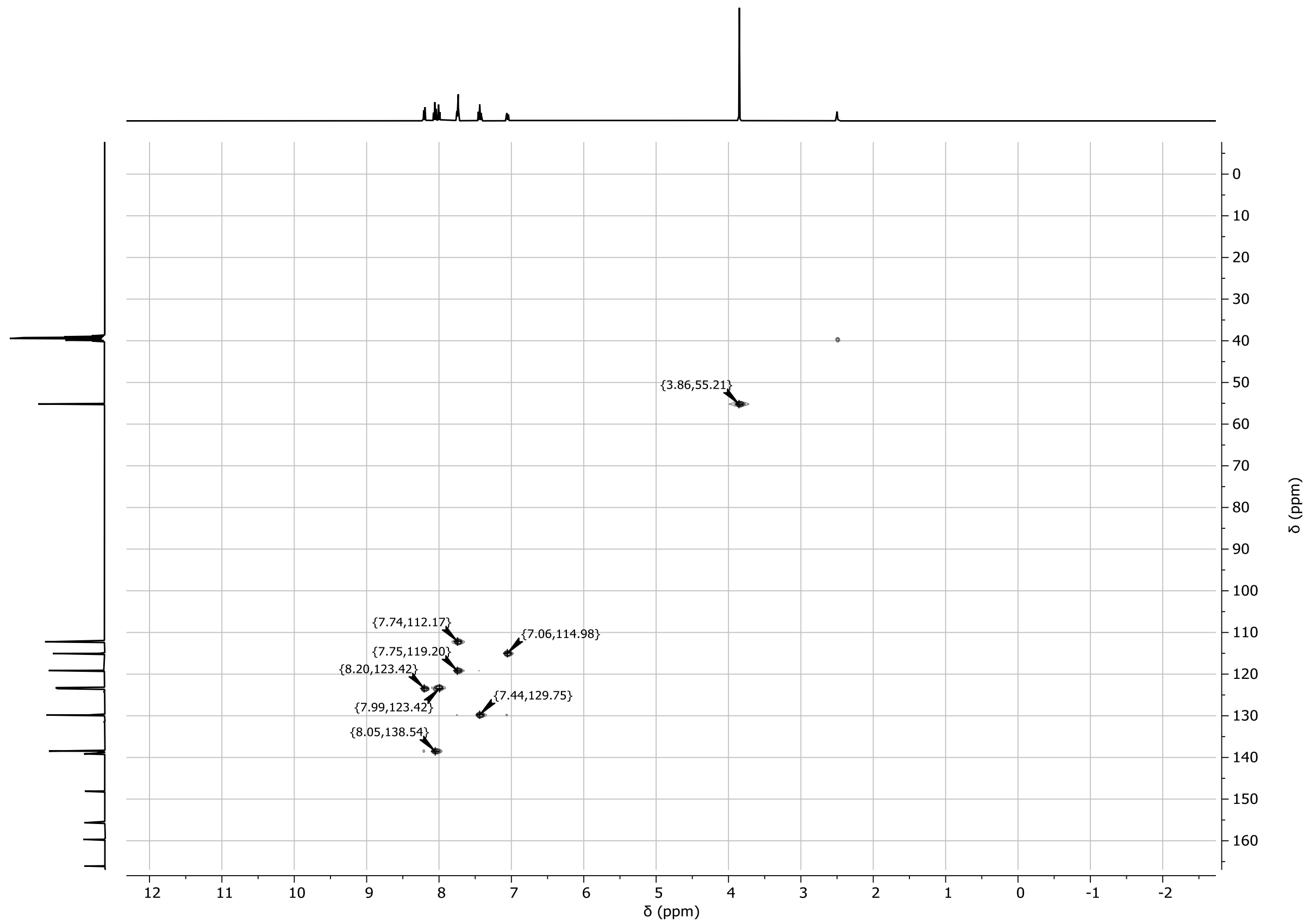

Figure S-55. HC-HSQC-NMR spectrum (101 MHz (^{13}C), 400 MHz (^{1}H), DMSO-d_6) of $\mathbf{L_{pOMe}}$.

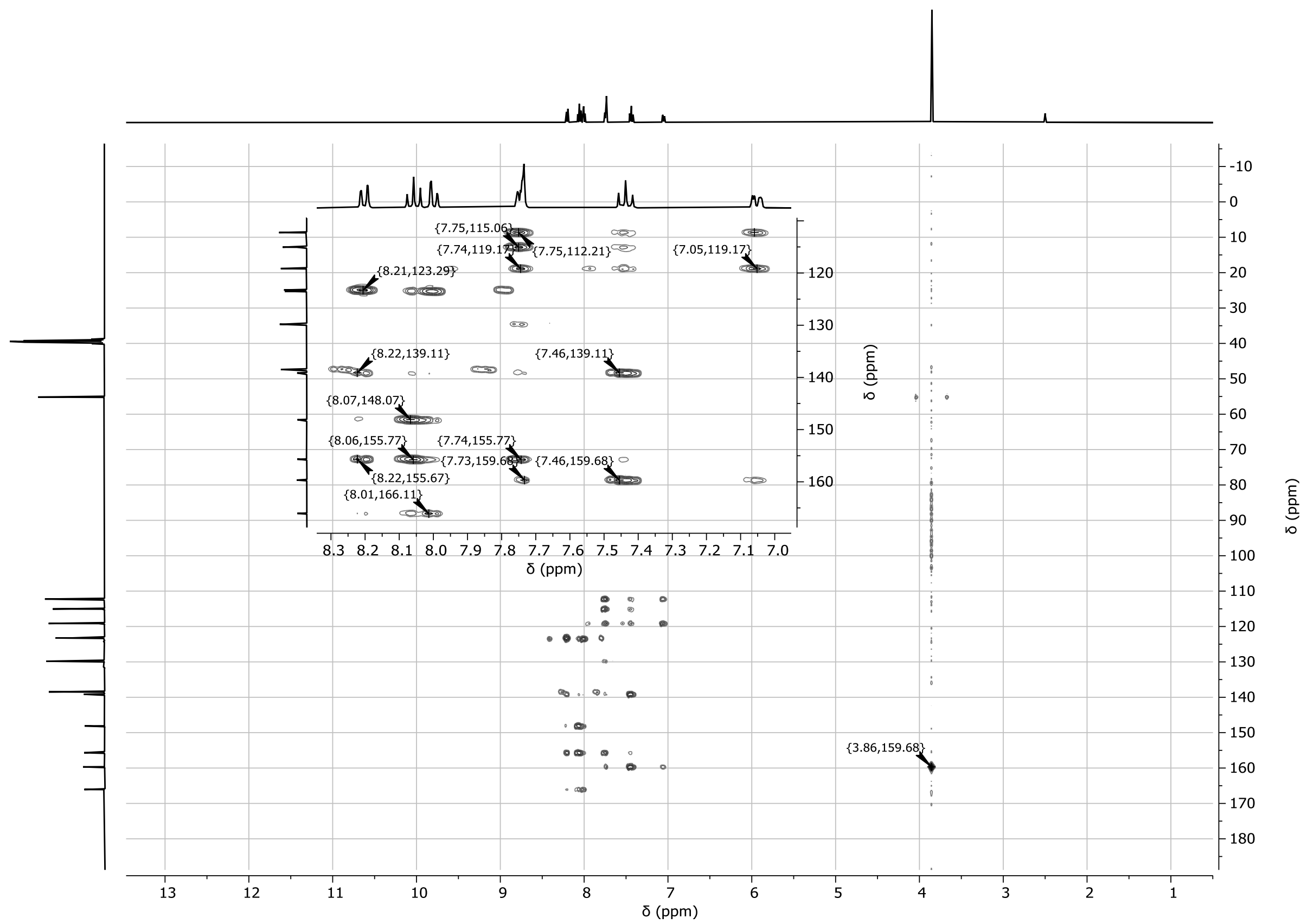

Figure S-56. HC-HMBC-NMR spectrum (101 MHz (^{13}C), 400 MHz (^{1}H), DMSO-d_6) of $\mathbf{L_{pOMe}}$.

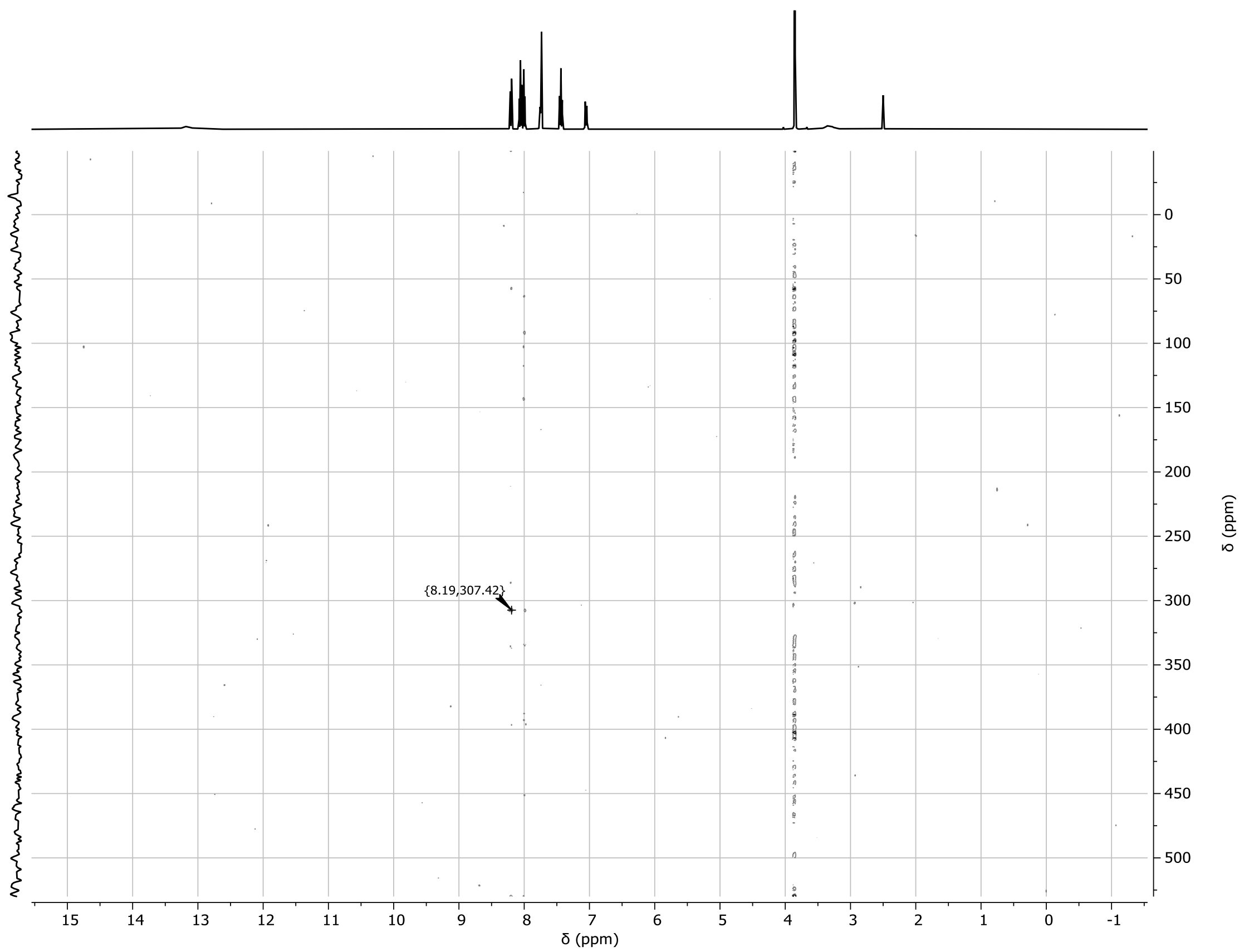

Figure S-57. HN-HMBC-NMR spectrum (41 MHz (^{13}C), 400 MHz (^{1}H), DMSO-d_6) of **L$_{\mathbf{pOMe}}$**.

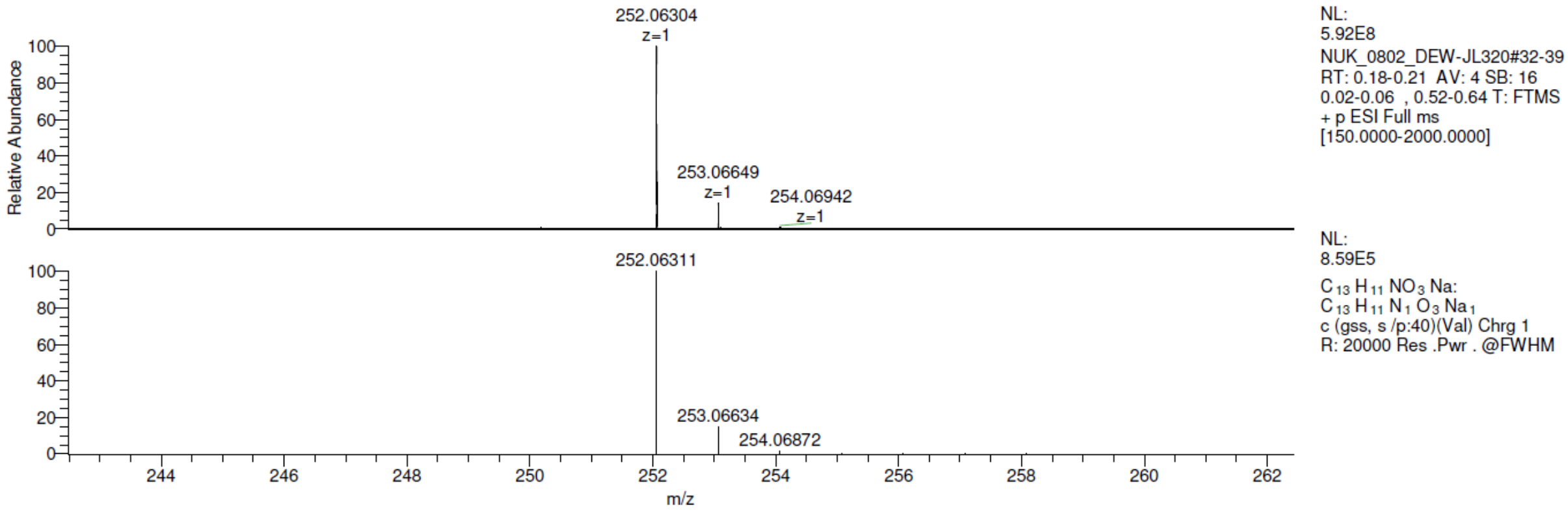

Figure S-58. Mass spectrum of **L$_{\mathbf{pOMe}}$** (MeOH). Additional simulation of the [**L$_{\mathbf{pOMe}}$**+H]$^+$ adduct.

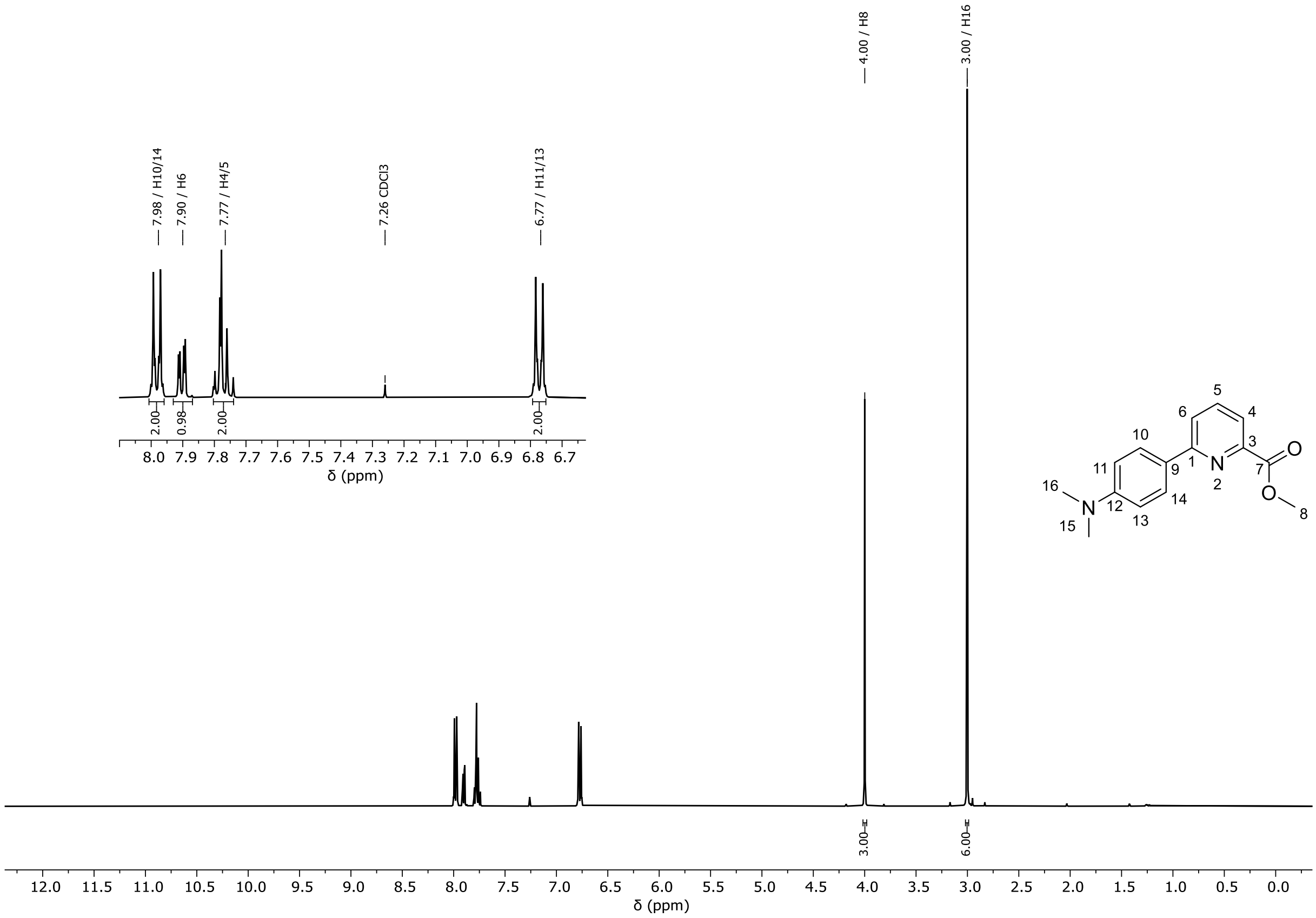

Figure S-59. ^{1}H-NMR spectrum (400 MHz, $CDCl_3$) of **6**.

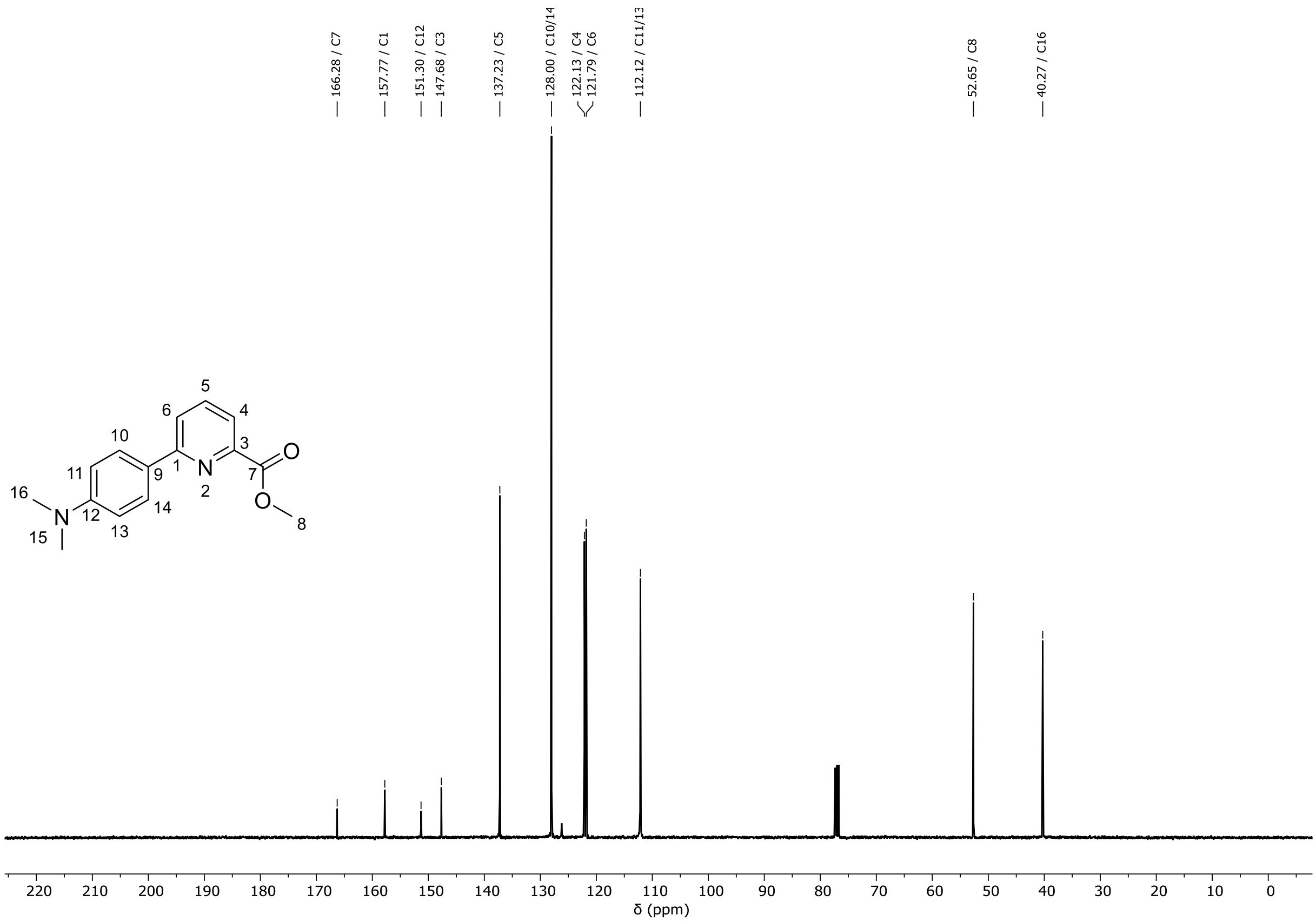

Figure S-60. $^{13}C\{^{1}H\}$-NMR spectrum (101 MHz, $CDCl_3$) of **6**.

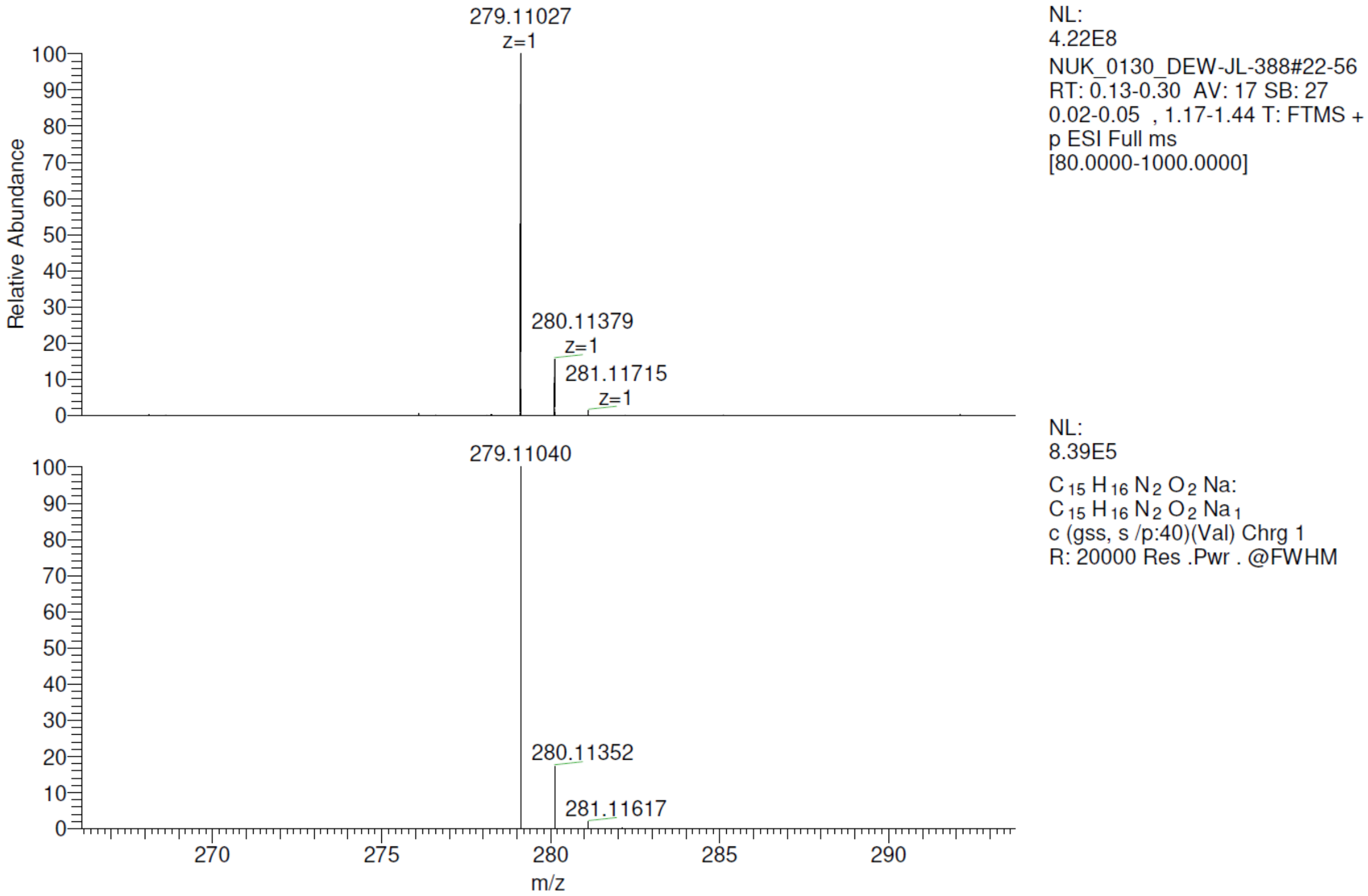

Figure S-61. Mass spectrum of **6** (MeOH). Additional simulation of the [**6**+Na]$^+$ adduct.

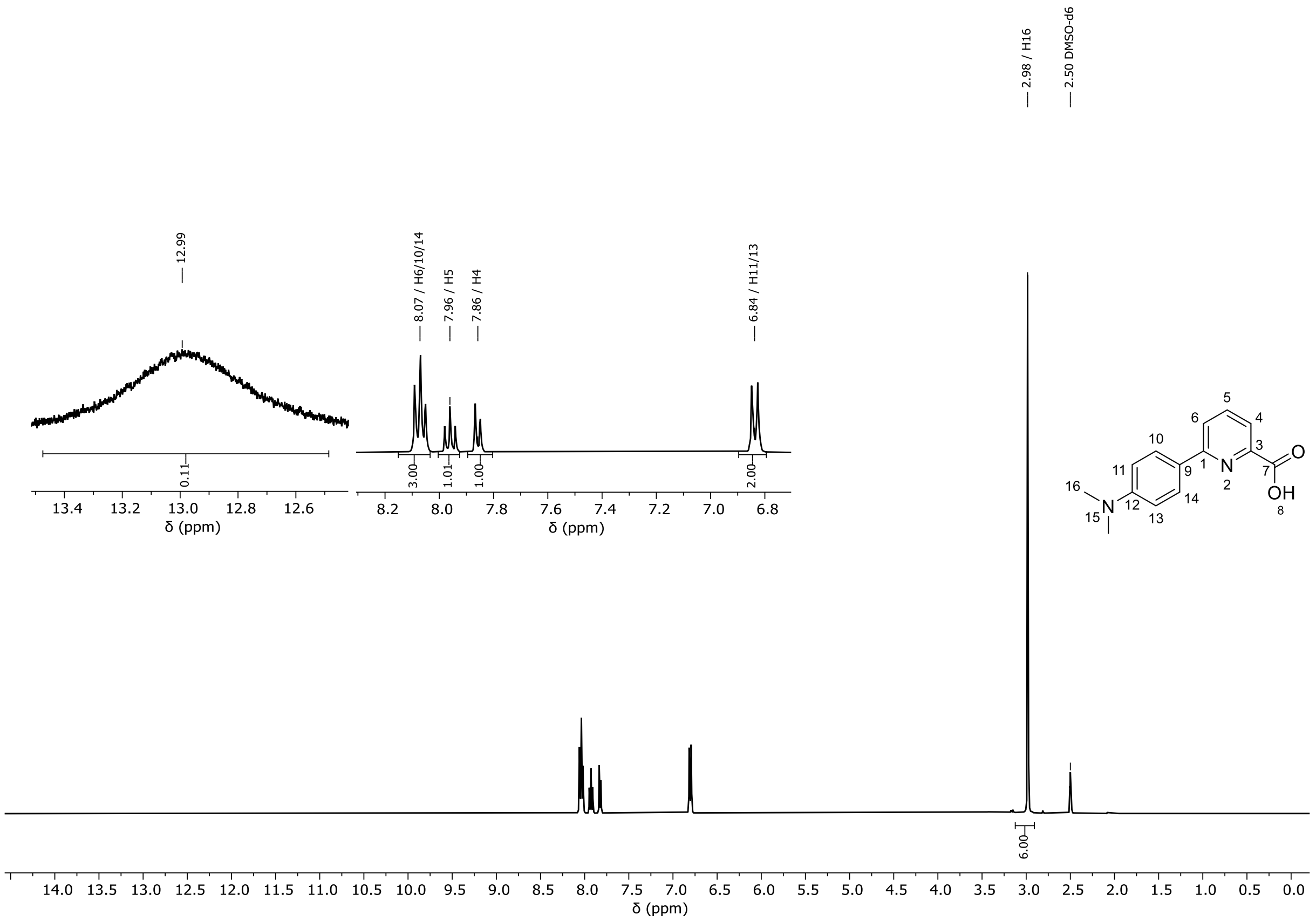

Figure S-62. ^{1}H-NMR spectrum (400 MHz, DMSO-d_6) of **L$_{mDMA}$**.

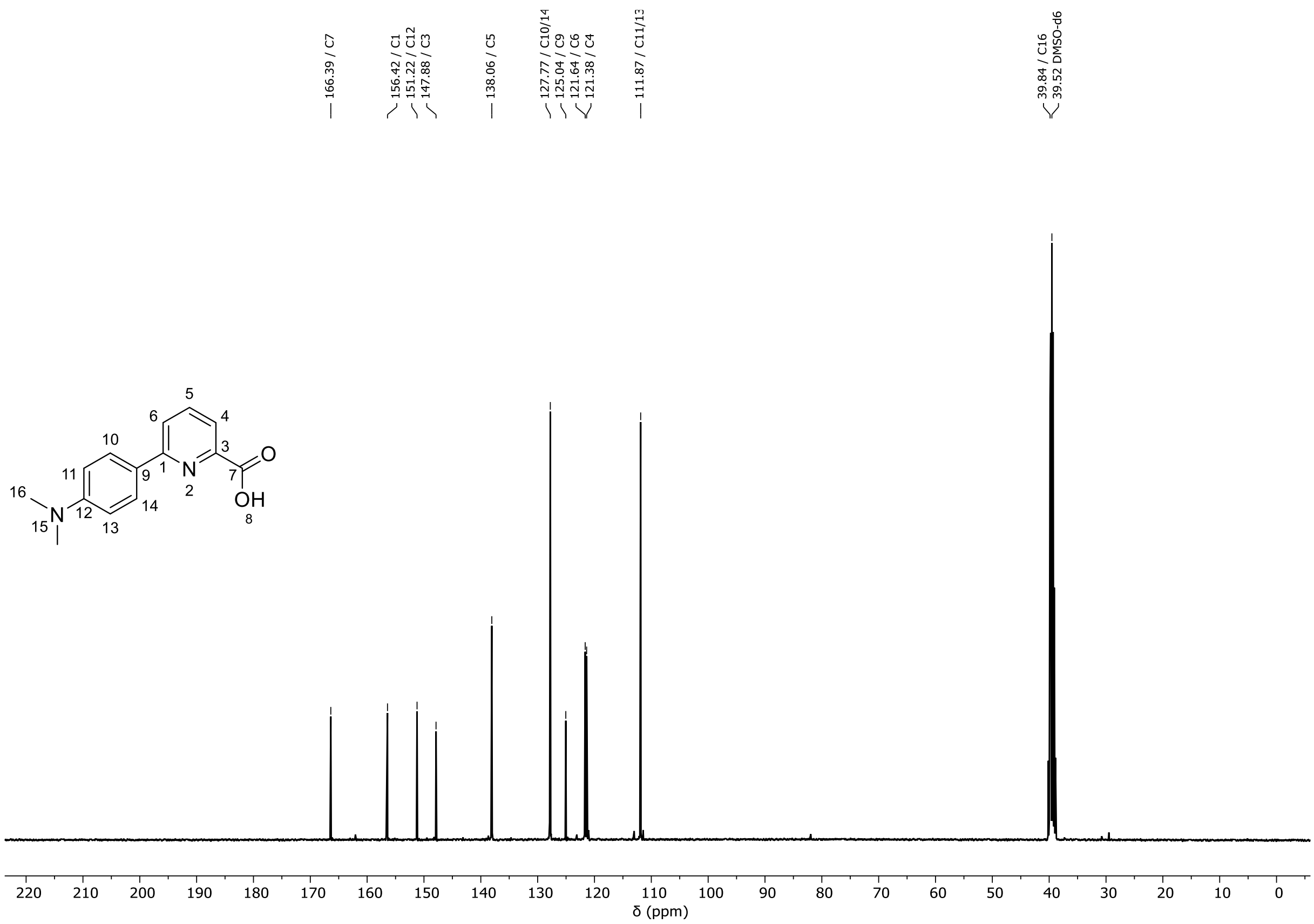

Figure S-63. $^{13}C\{^{1}H\}$-NMR spectrum (101 MHz, DMSO-d_6) of $\mathbf{L_{mDMA}}$.

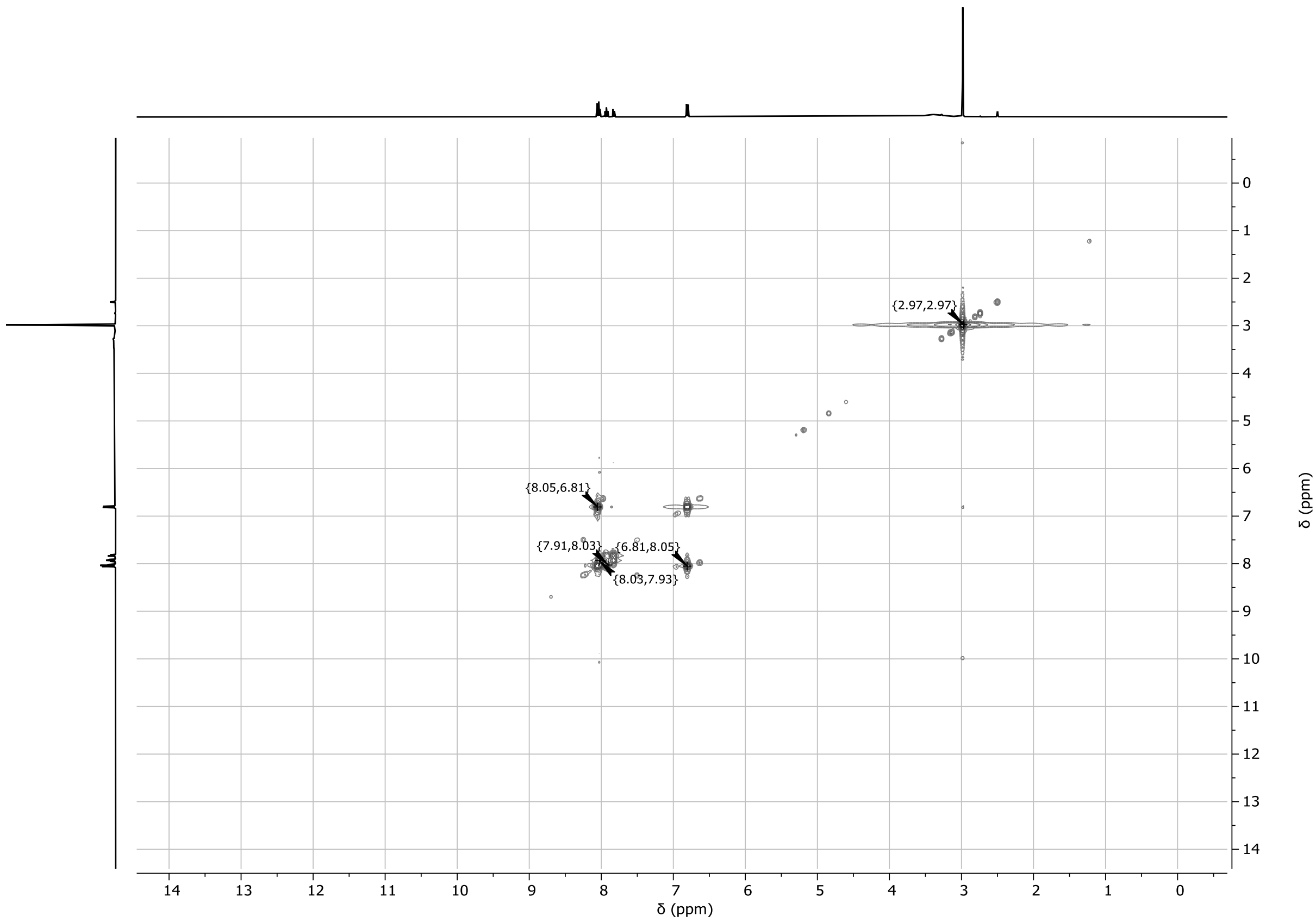

Figure S-64. HH-COSY-NMR spectrum (400 MHz (^{1}H), DMSO-d_6) of $\mathbf{L_{mDMA}}$.

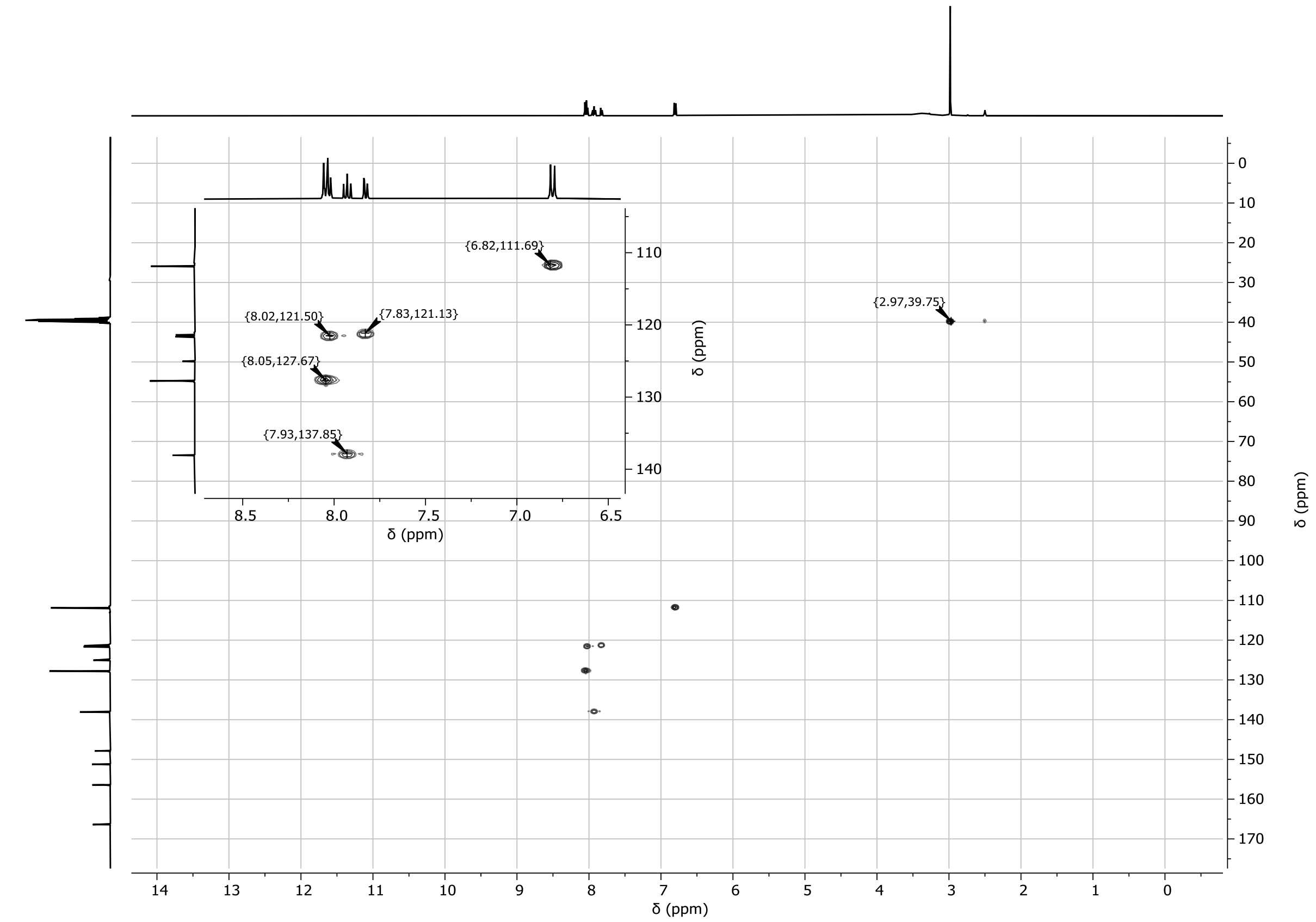

Figure S-65. HC-HSQC-NMR spectrum (101 MHz (^{13}C), 400 MHz (^{1}H), DMSO-d_6) of $\mathbf{L_{mDMA}}$.

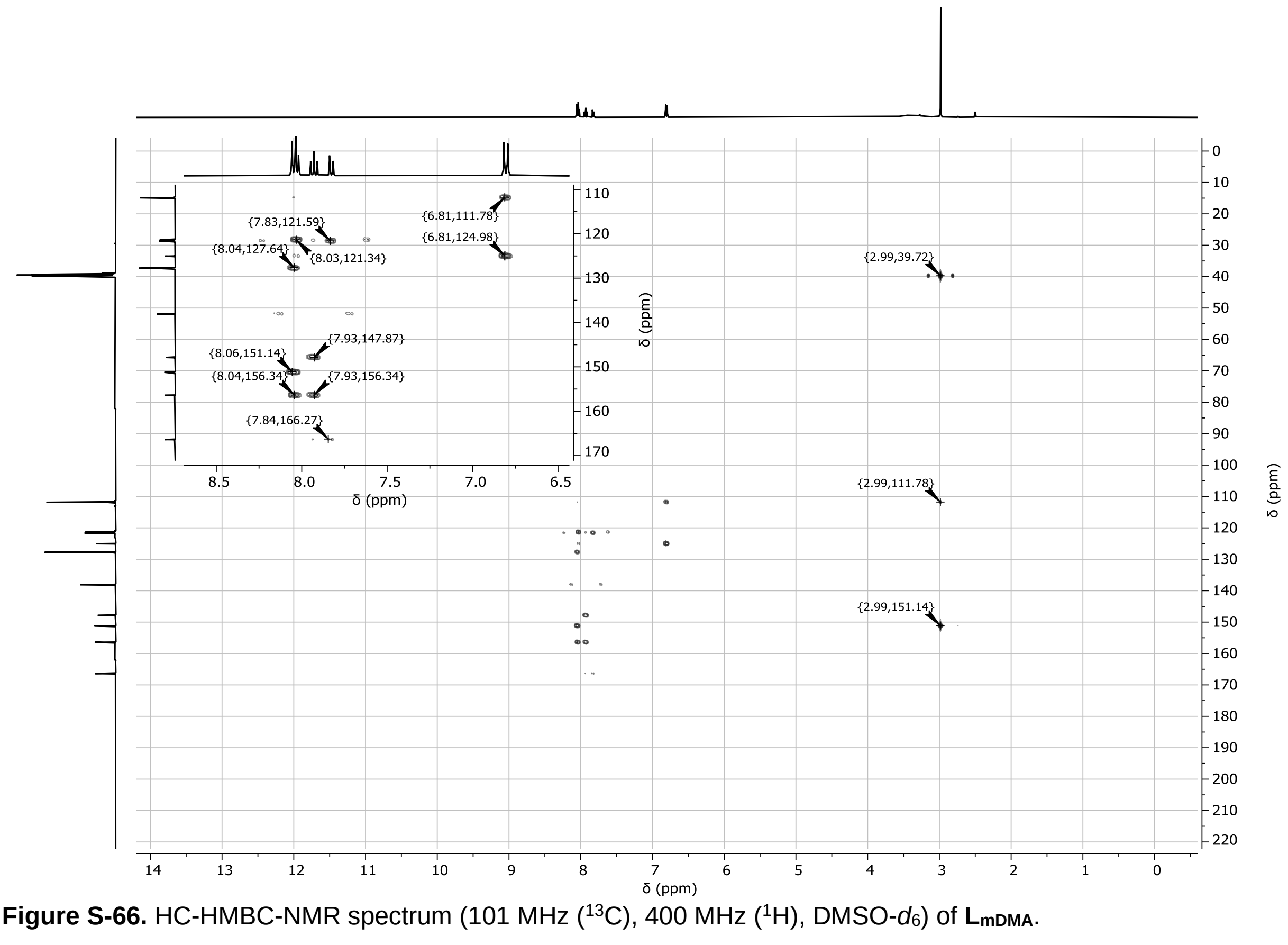

Figure S-66. HC-HMBC-NMR spectrum (101 MHz (^{13}C), 400 MHz (^{1}H), DMSO-d_6) of $\mathbf{L_{mDMA}}$.

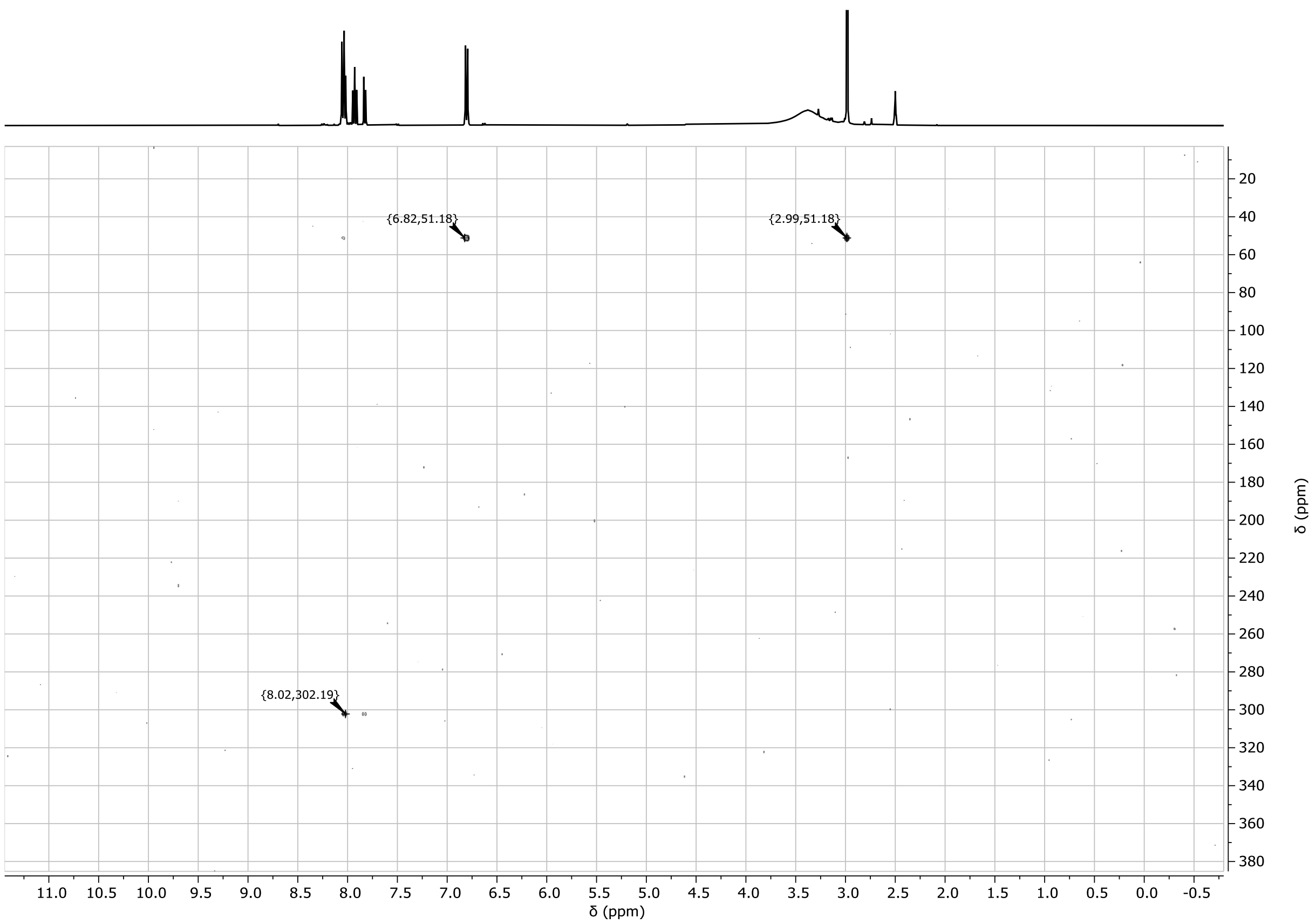

Figure S-67. HN-HMBC-NMR spectrum (41 MHz (^{15}N), 400 MHz (^{1}H), DMSO-d_6) of $\mathbf{L_{mDMA}}$.

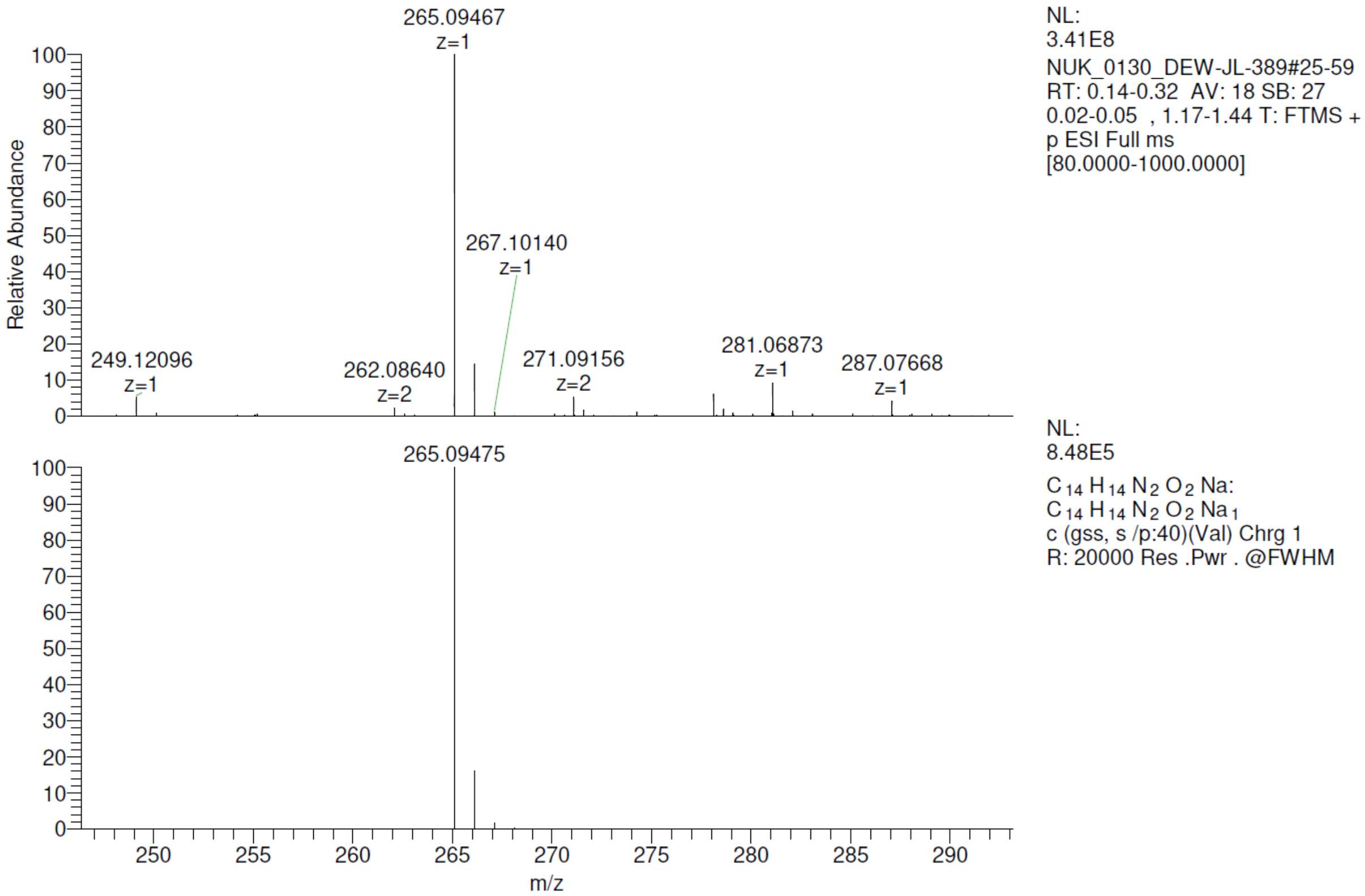

Figure S-68. Mass spectrum of $\mathbf{L_{mDMA}}$ (MeOH). Additional simulation of the [$\mathbf{L_{mDMA}}$+Na]$^+$ adduct.

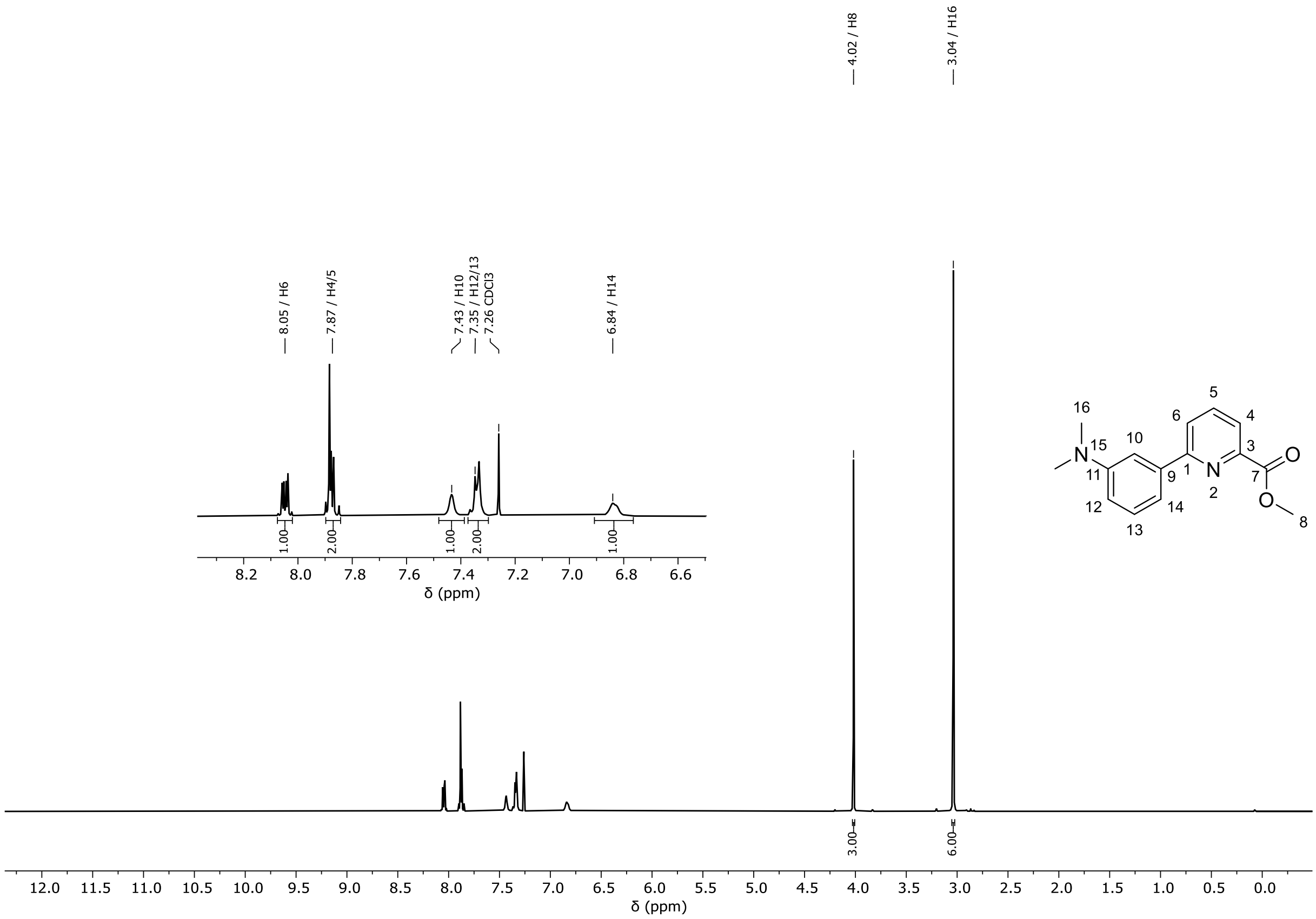

Figure S-69. ^{1}H-NMR spectrum (400 MHz, $CDCl_3$) of **7**.

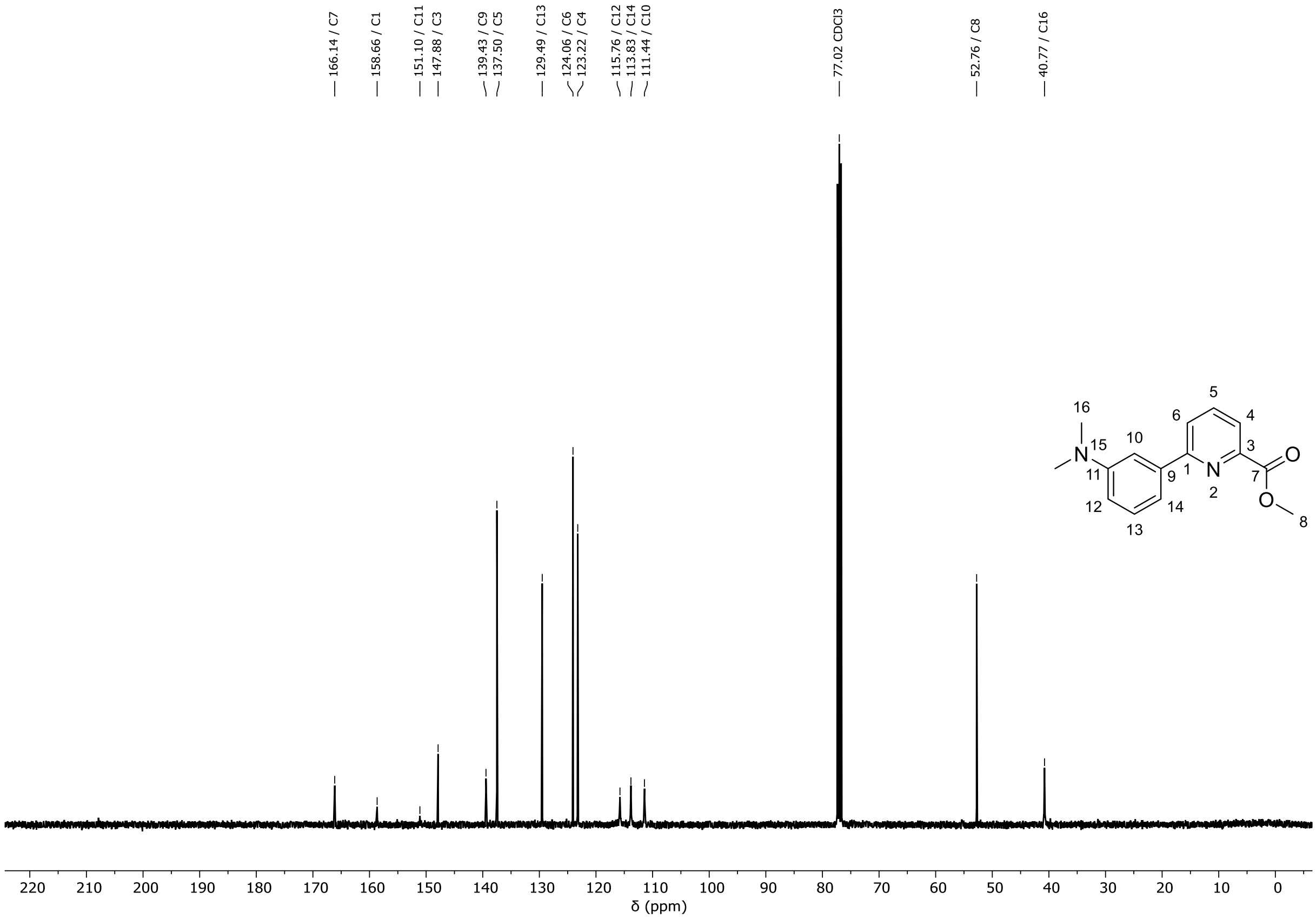

Figure S-70. $^{13}C\{^{1}H\}$-NMR spectrum (101 MHz, $CDCl_3$) of **7**.

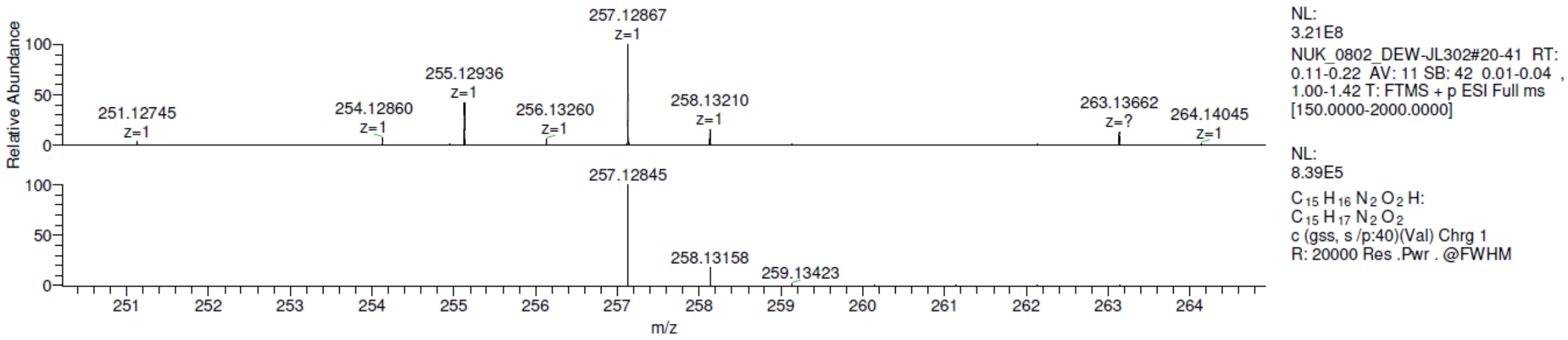

Figure S-71. Mass spectrum of **7** (MeOH). Additional simulation of the [**7**+H]$^+$ adduct.

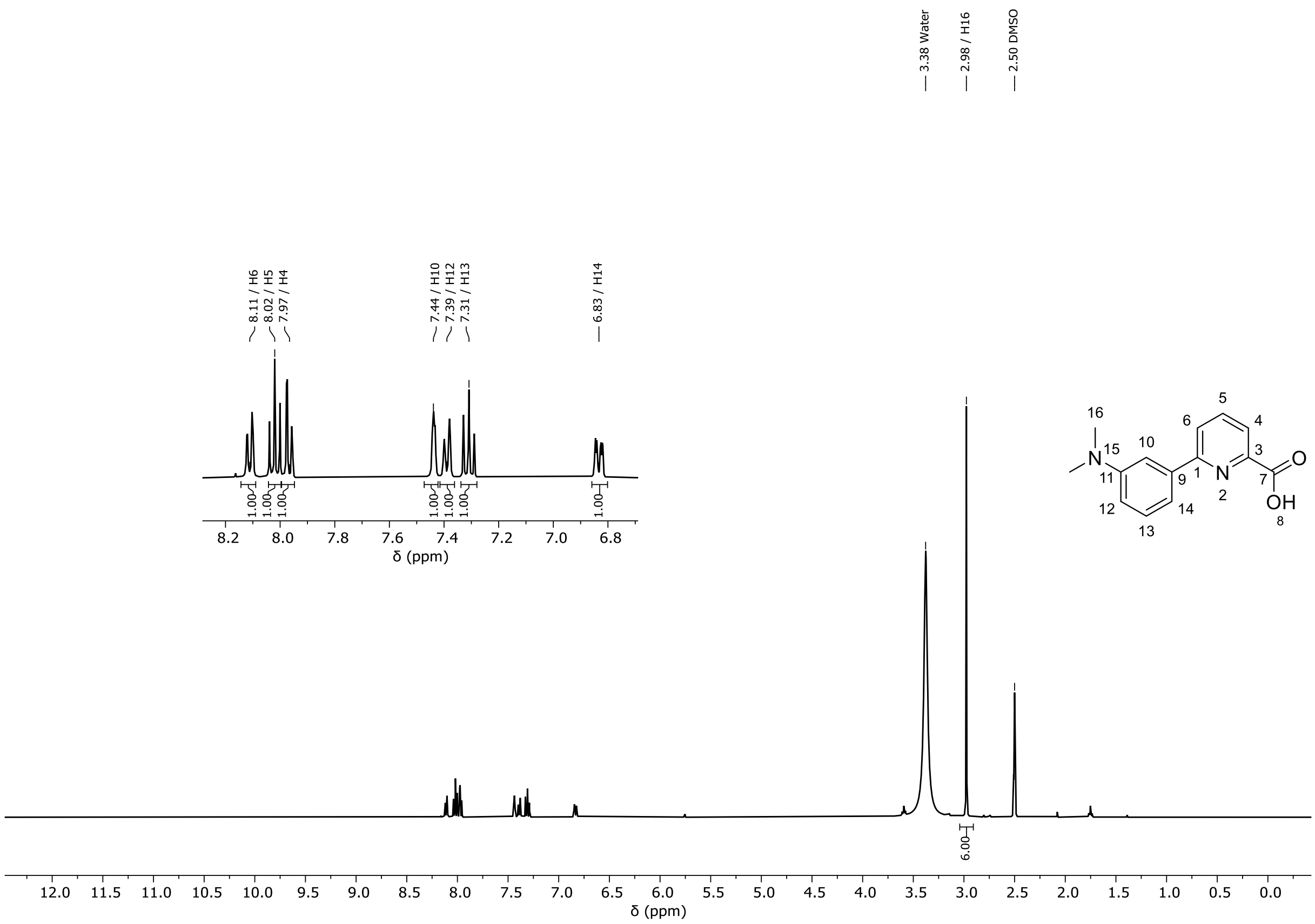

Figure S-72. ^{1}H-NMR spectrum (400 MHz, DMSO-d_6) of $\mathbf{L_{pDMA}}$.

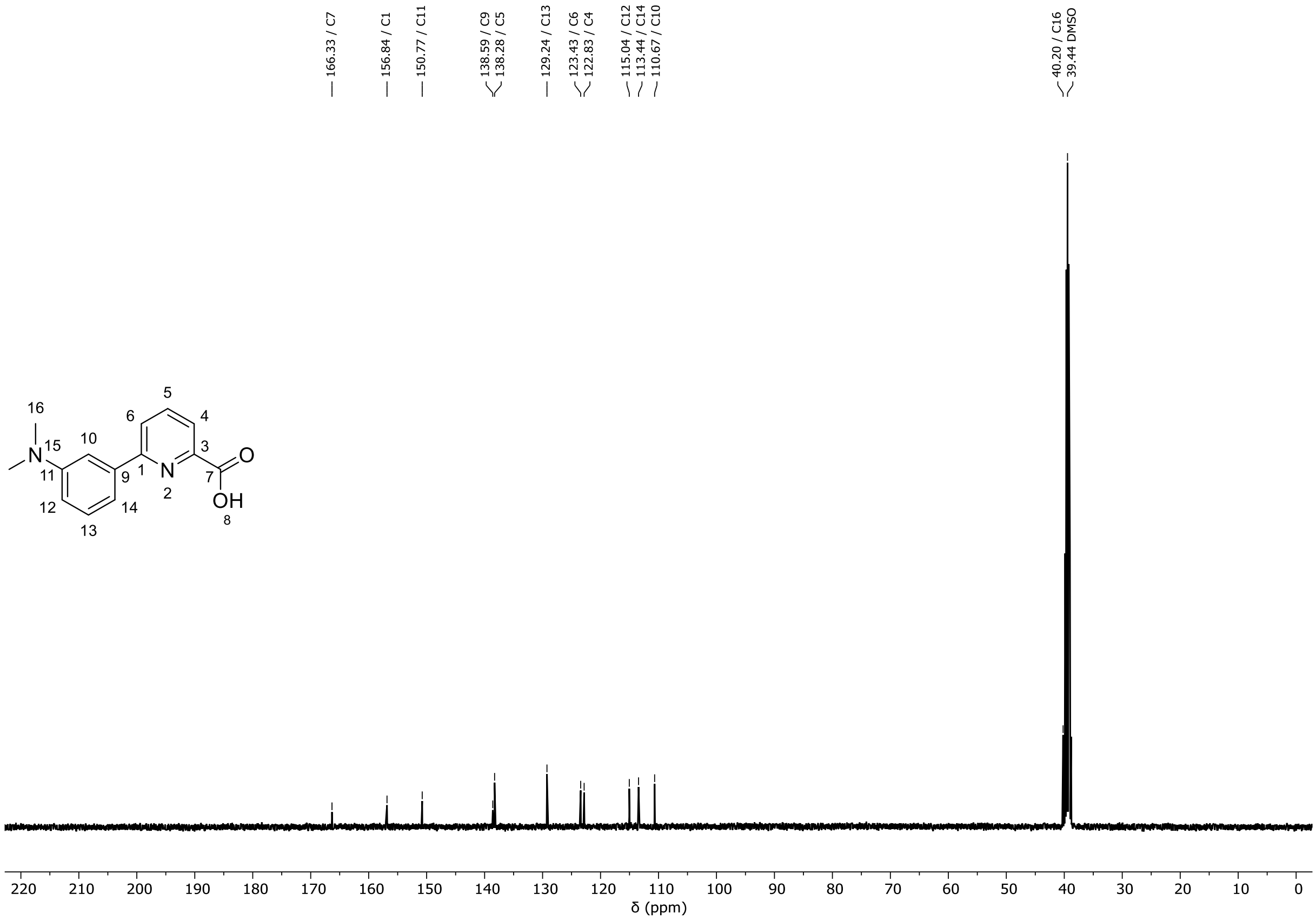

Figure S-73. $^{13}C\{^{1}H\}$-NMR spectrum (101 MHz, DMSO-d_6) of $\mathbf{L_{pDMA}}$.

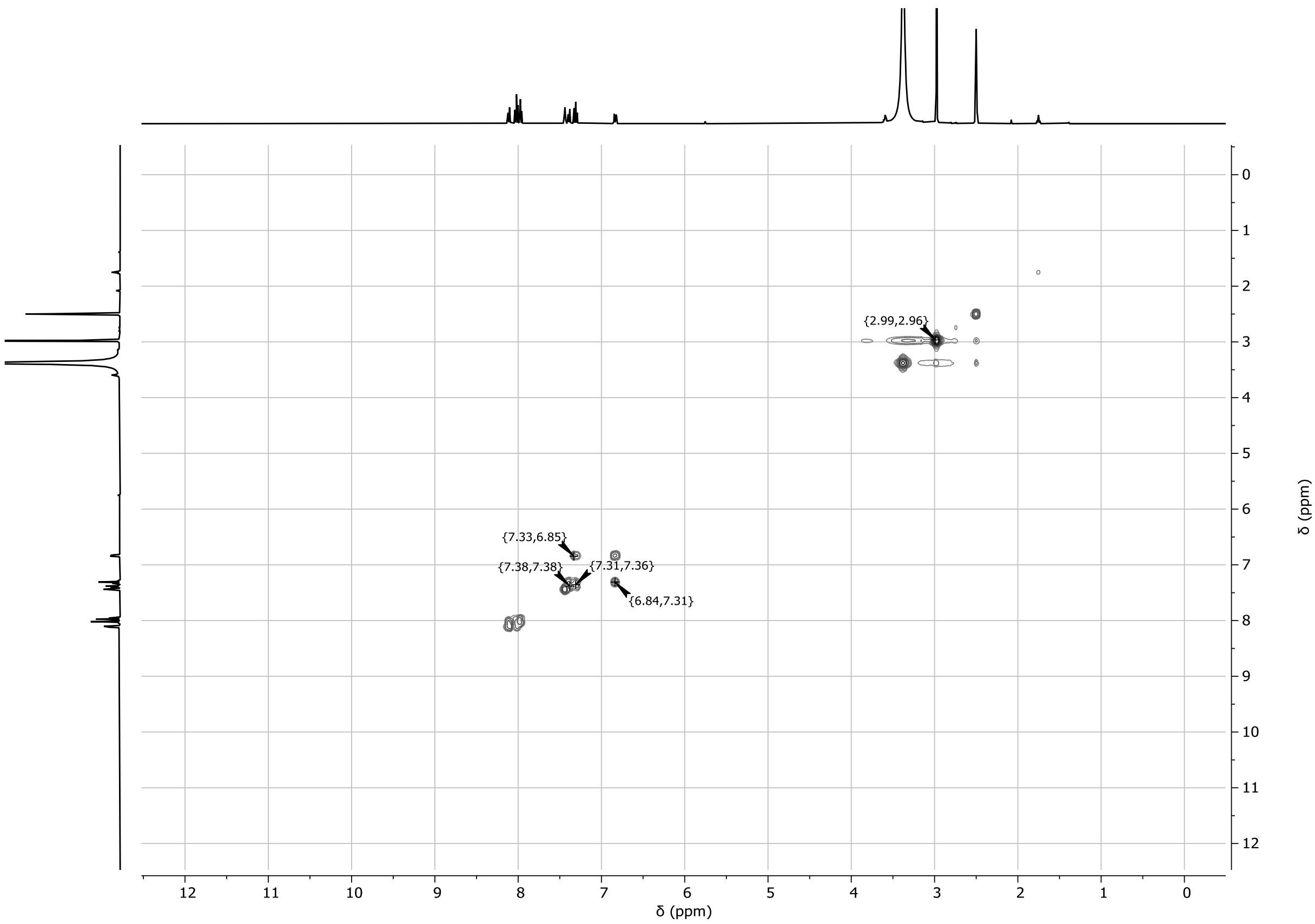

Figure S-74. HH-COSY-NMR spectrum (400 MHz, DMSO-d_6) of $\mathbf{L_{pDMA}}$.

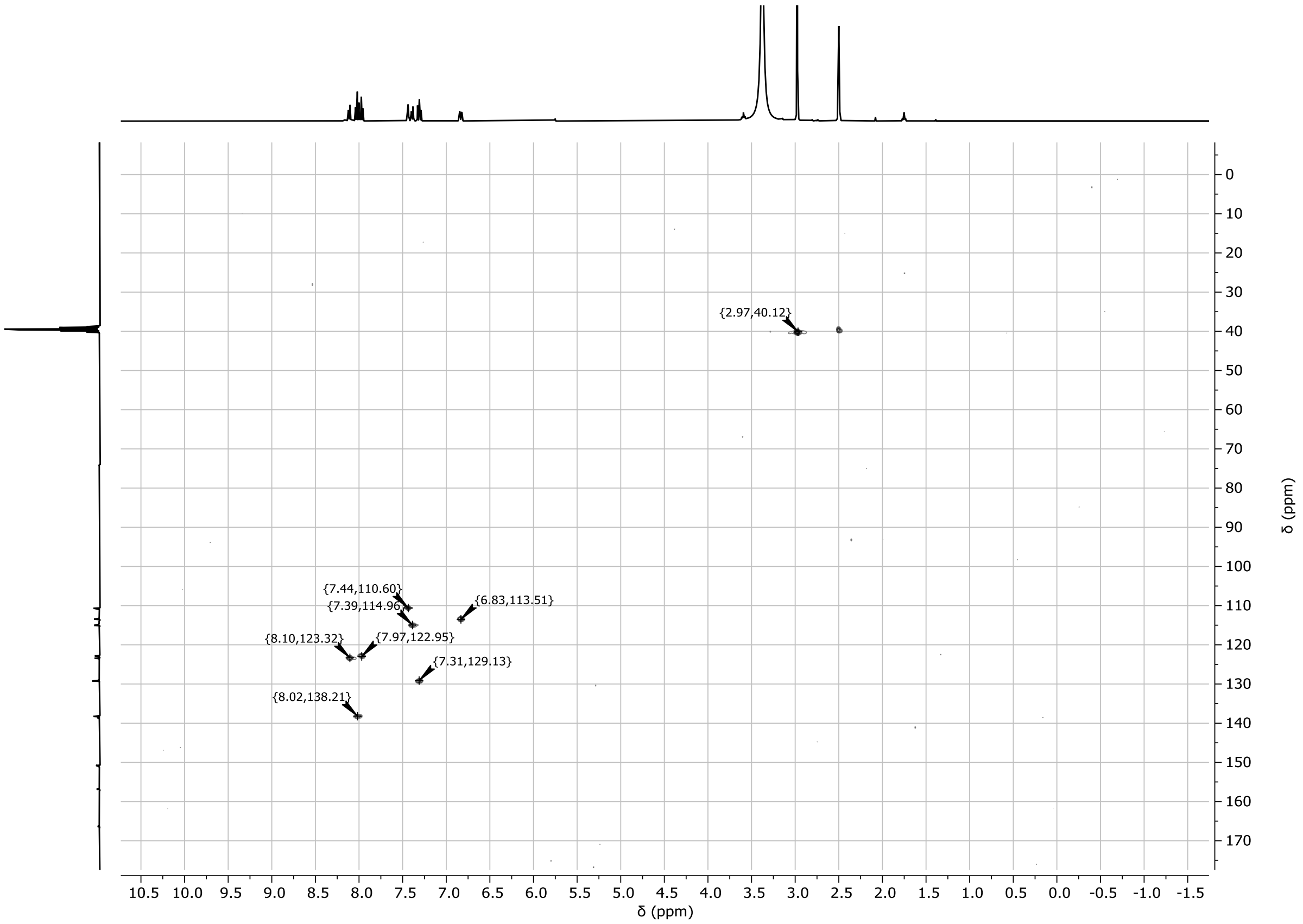

Figure S-75. HC-HSQC-NMR spectrum (101 MHz (^{13}C), 400 MHz (^{1}H), DMSO-d_6) of $\mathbf{L_{pDMA}}$.

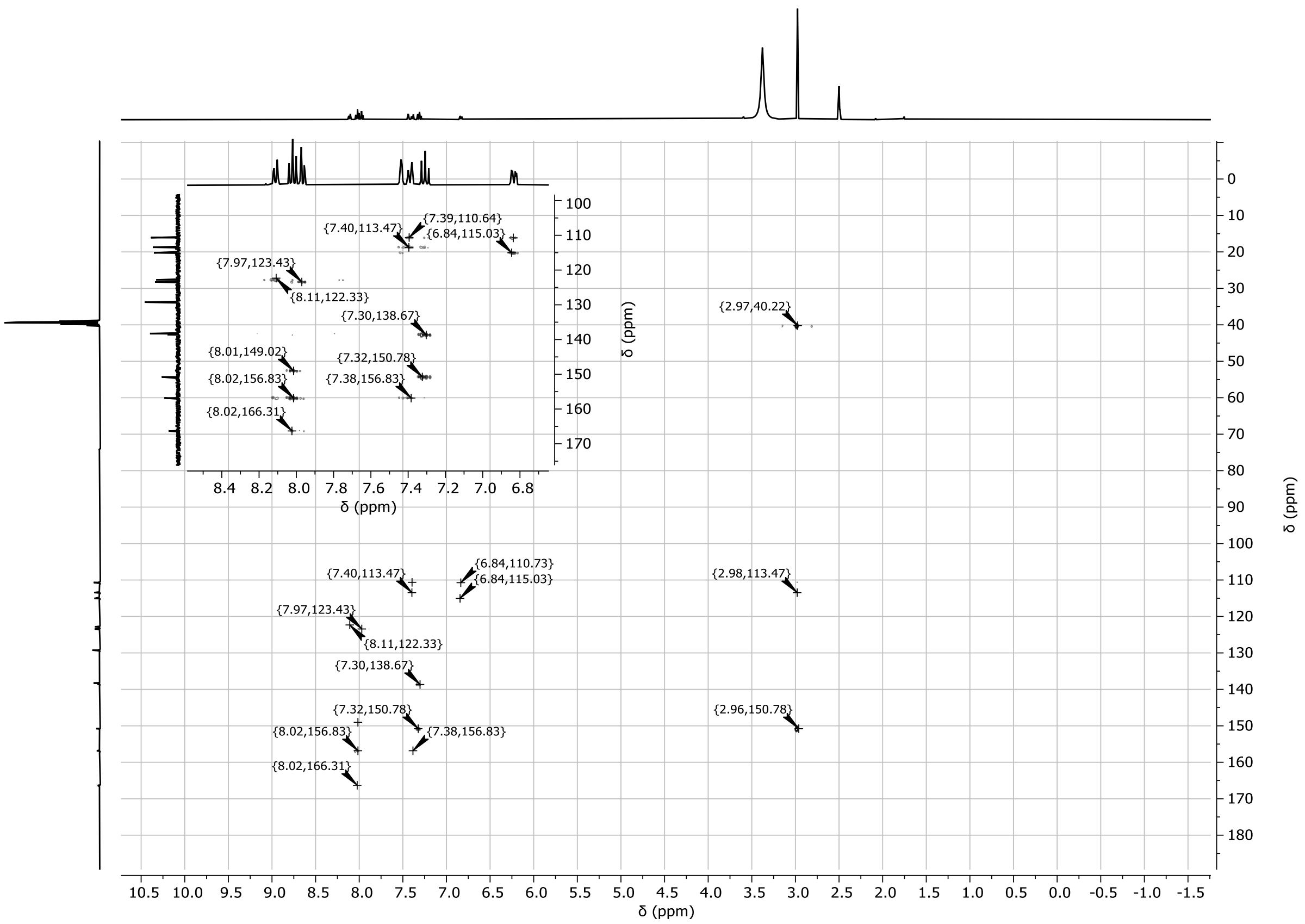

Figure S-76. HC-HMBC-NMR spectrum (101 MHz (^{13}C), 400 MHz (^{1}H), DMSO-d_6) of $\mathbf{L_{pDMA}}$.

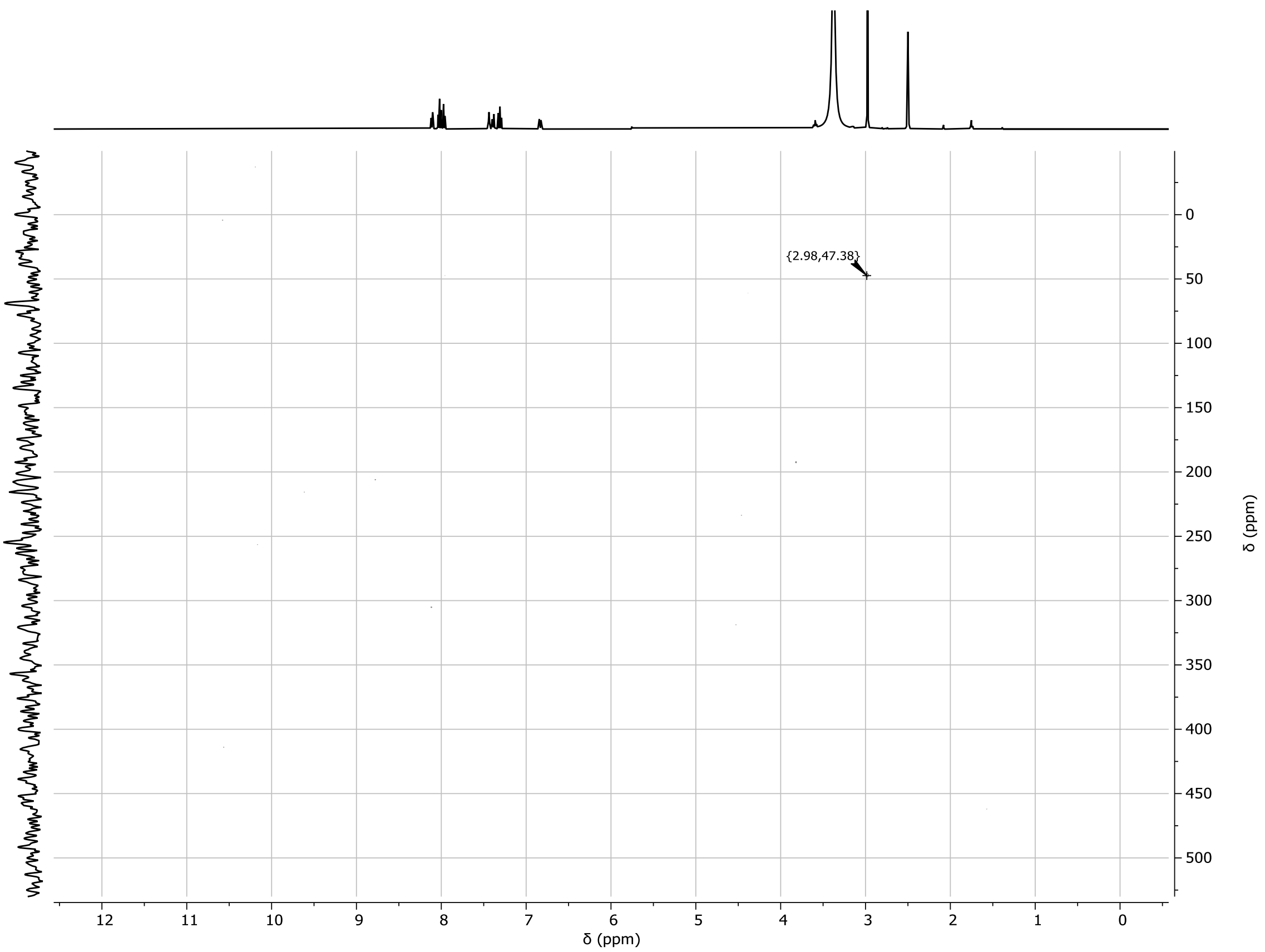

Figure S-77. HN-HMBC-NMR spectrum (41 MHz (^{15}N), 400 MHz (^{1}H), DMSO-d_6) of **L_{pDMA}**.

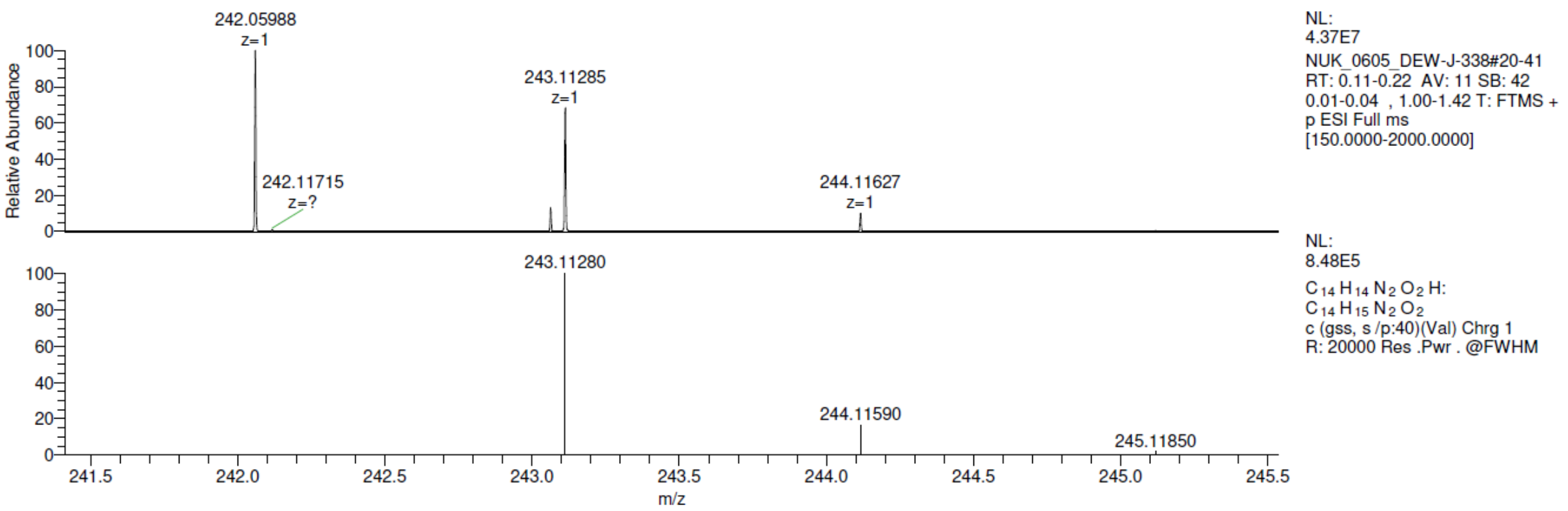

Figure S-78. Mass spectrum of **L_{pDMA}** (MeOH). Additional simulation of the [**L_{pDMA}**+H]$^+$ adduct.

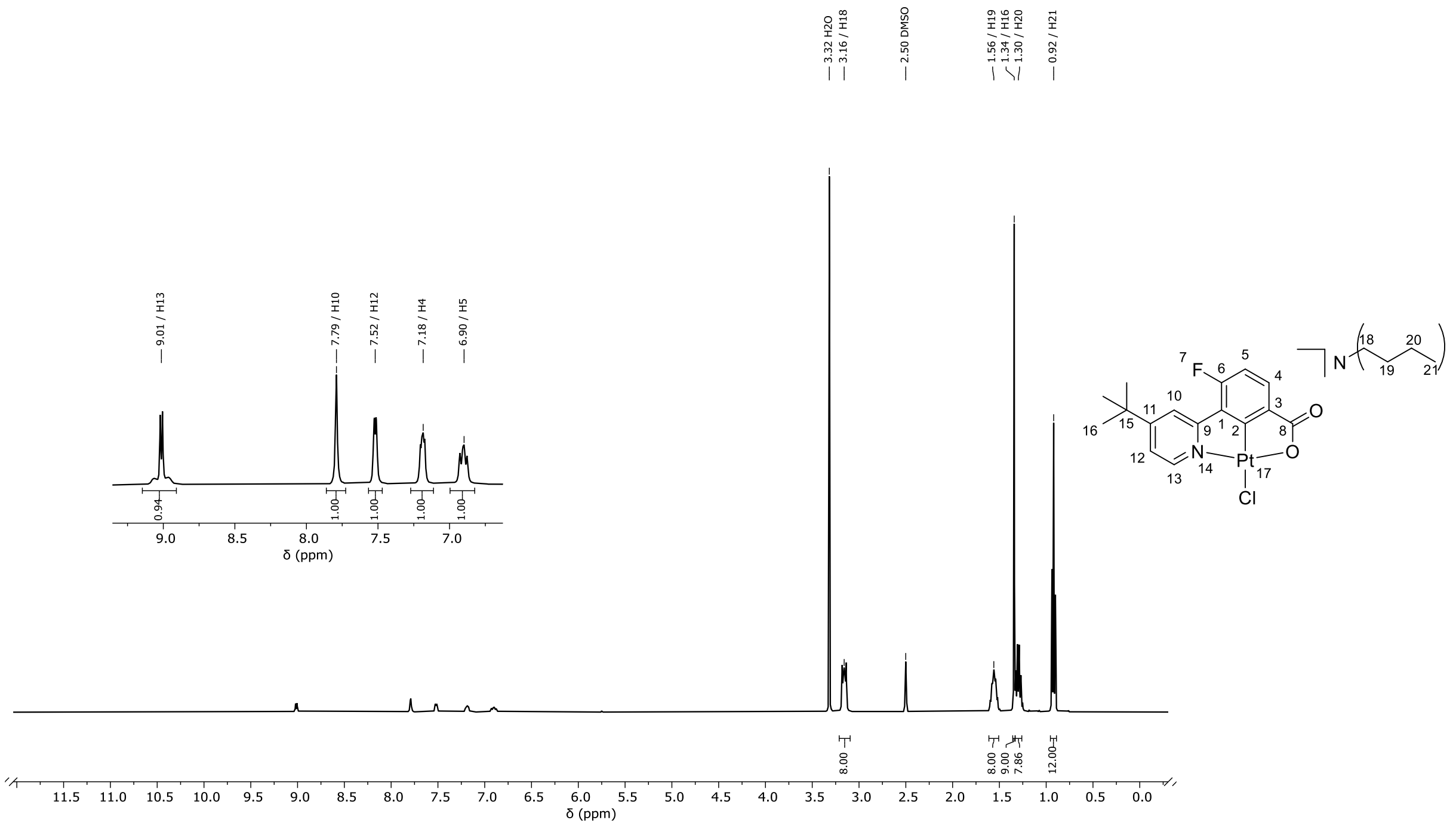

Figure S-79. ^{1}H-NMR spectrum (400 MHz, DMSO-d_6) of **Pt(L$_{NCO}$)Cl**.

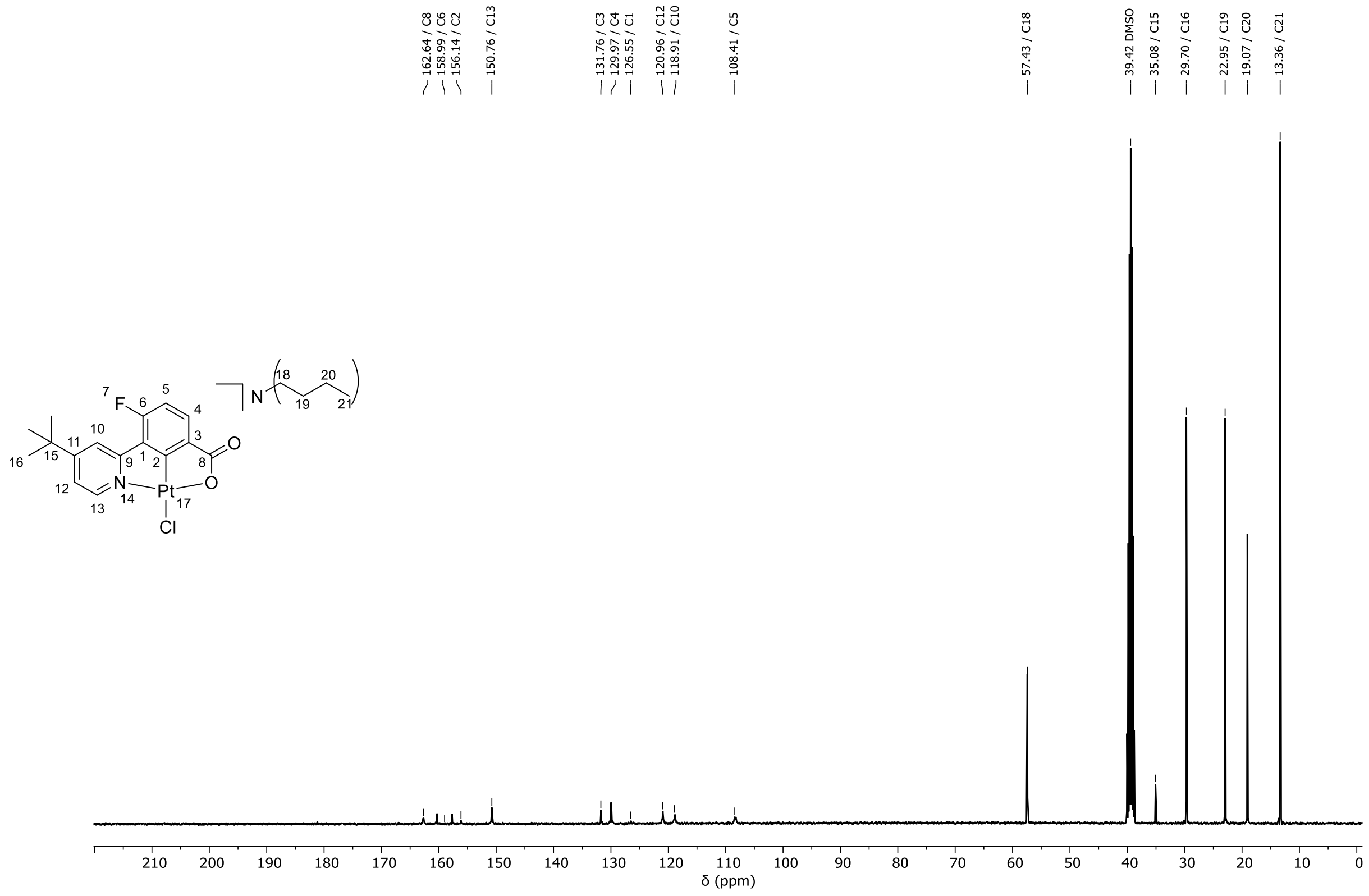

Figure S-80. ^{13}C{^{1}H}-NMR spectrum (101 MHz, DMSO-d_6) of **Pt(L$_{NCO}$)Cl**.

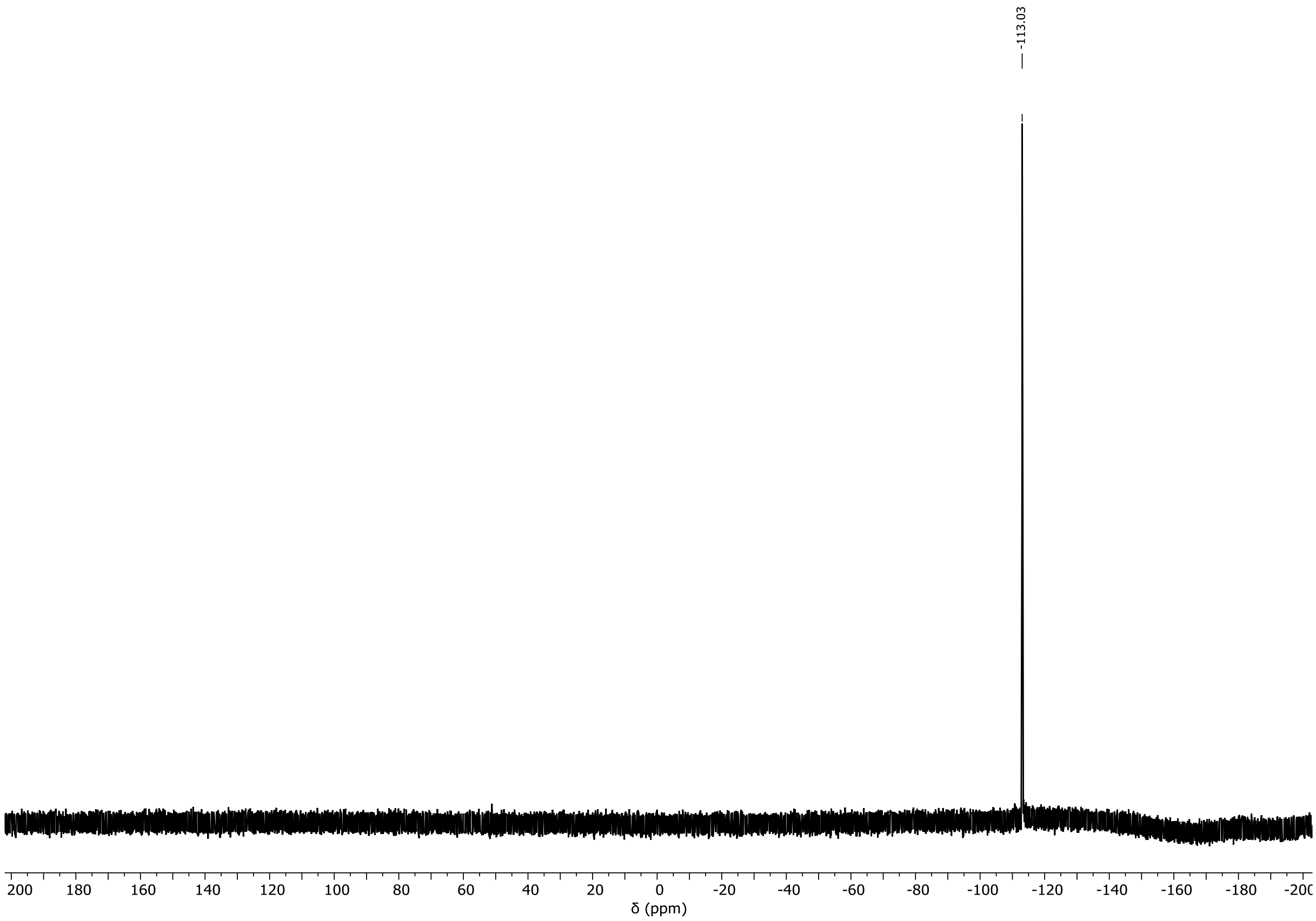

Figure S-81. $^{19}F\{^{1}H\}$-NMR spectrum (376 MHz, DMSO-d_6) of **Pt(L_{NCO})Cl**.

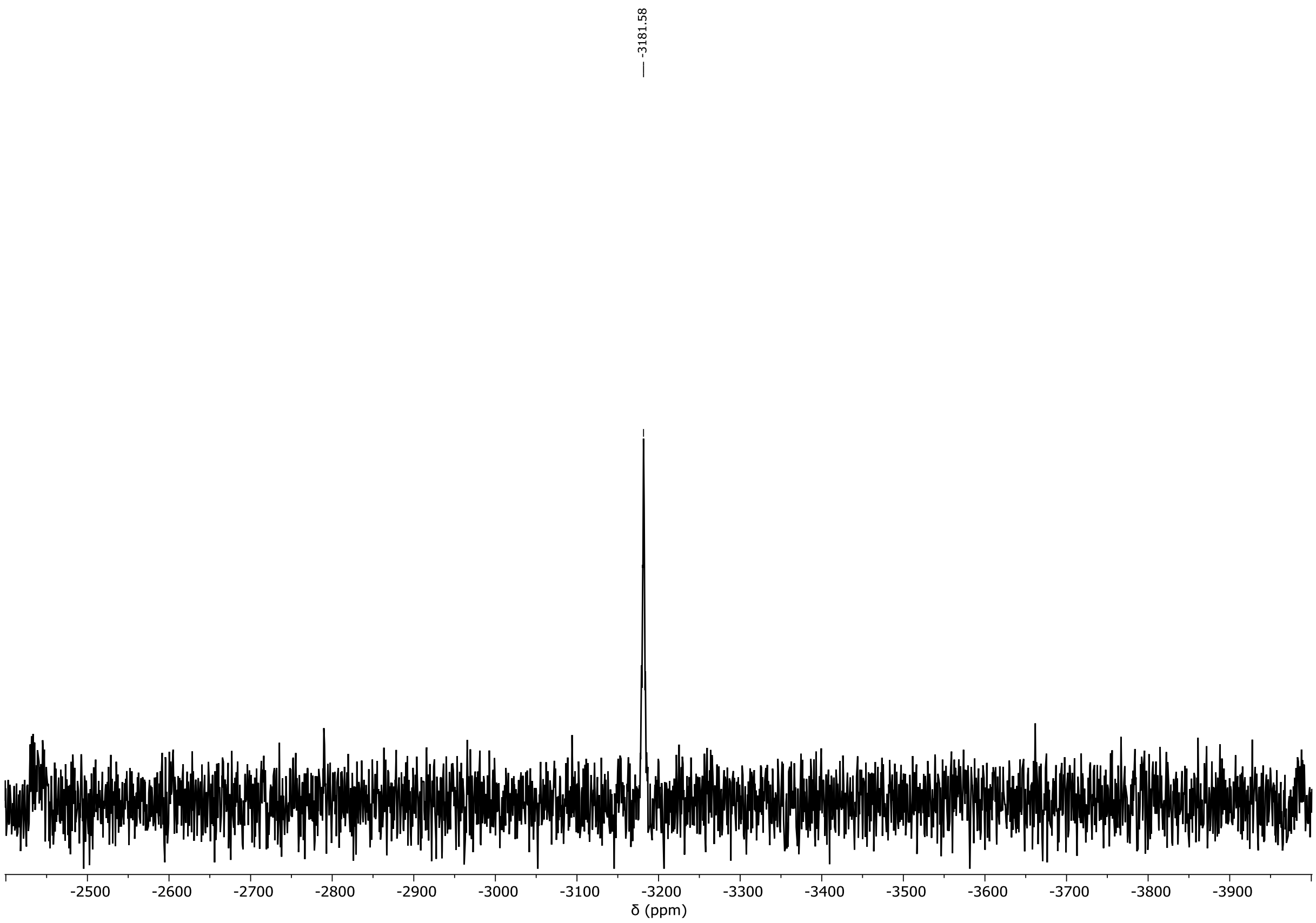

Figure S-82. ^{195}Pt-NMR spectrum (107 MHz, DMSO-d_6) of **Pt(L_{NCO})Cl**.

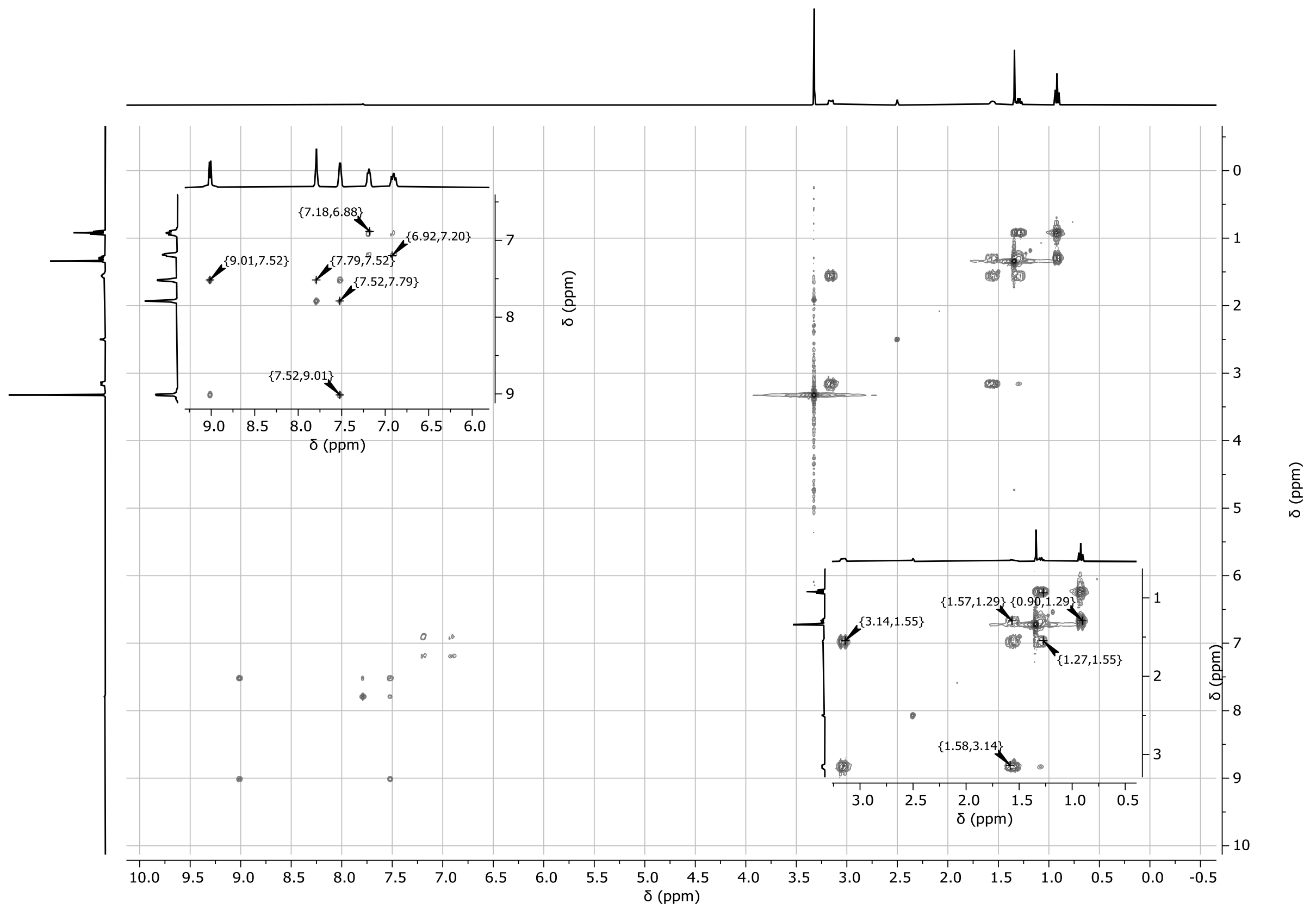

Figure S-83. HH-COSY-NMR spectrum (400 MHz, DMSO-d_6) of **Pt(L$_{NCO}$)Cl**.

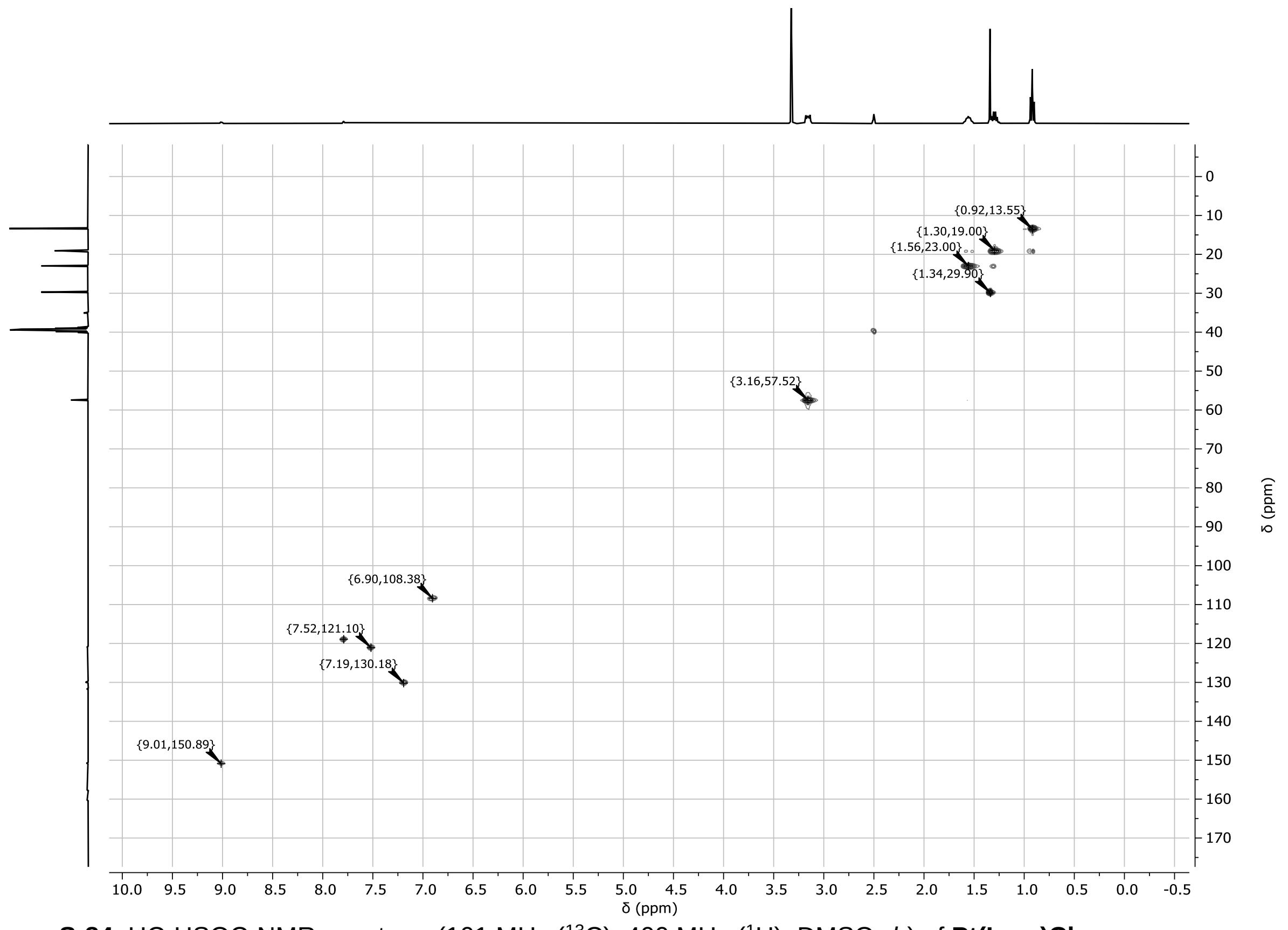

Figure S-84. HC-HSQC-NMR spectrum (101 MHz (^{13}C), 400 MHz (^{1}H), DMSO-d_6) of **Pt(L$_{NCO}$)Cl**.

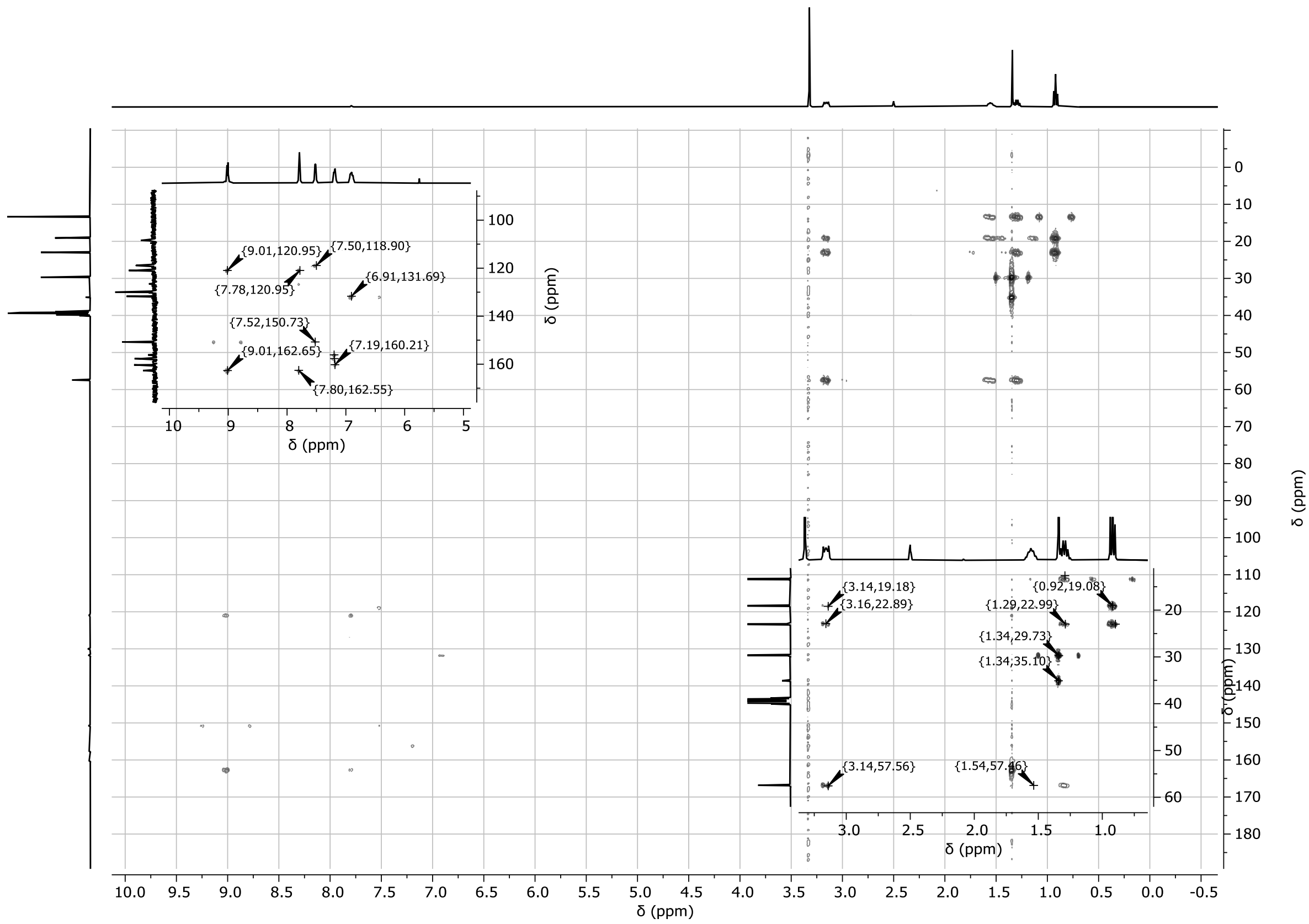

Figure S-85. HC-HMBC-NMR spectrum (101 MHz (^{13}C), 400 MHz (^{1}H), DMSO-d_6) of **Pt(L$_{NCO}$)Cl**.

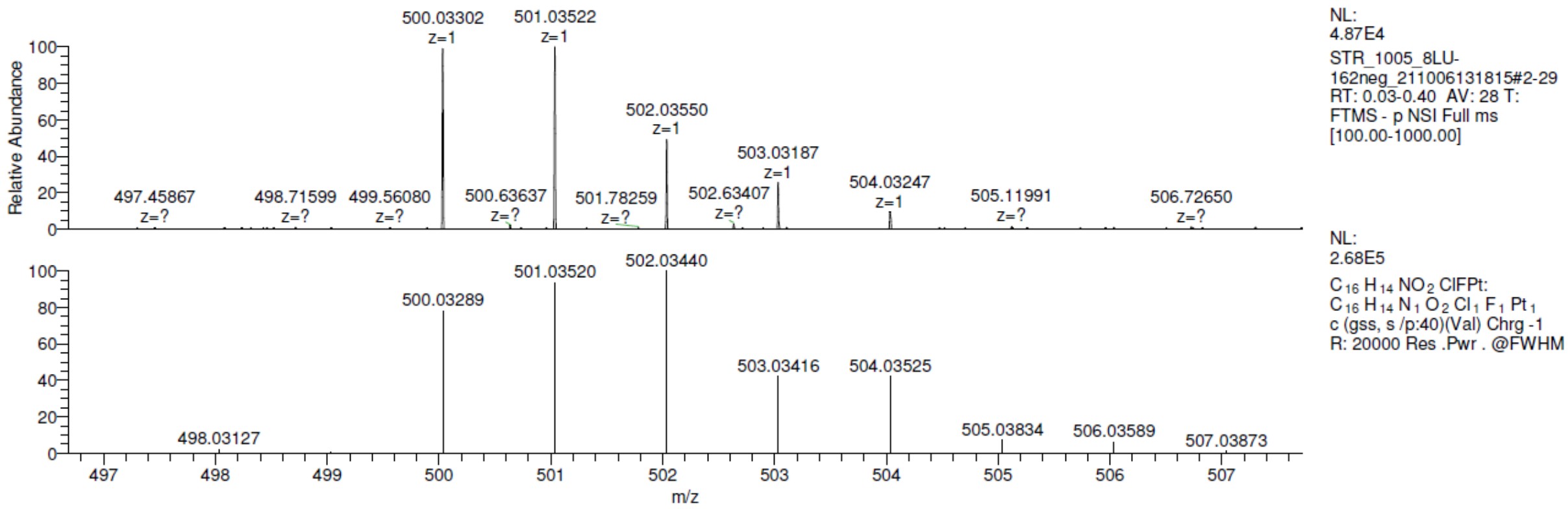

Figure S-86. Mass spectrum of **Pt(L$_{NCO}$)Cl** (MeOH). Additional simulation of the [**Pt(L$_{NCO}$)Cl**]$^-$ adduct.

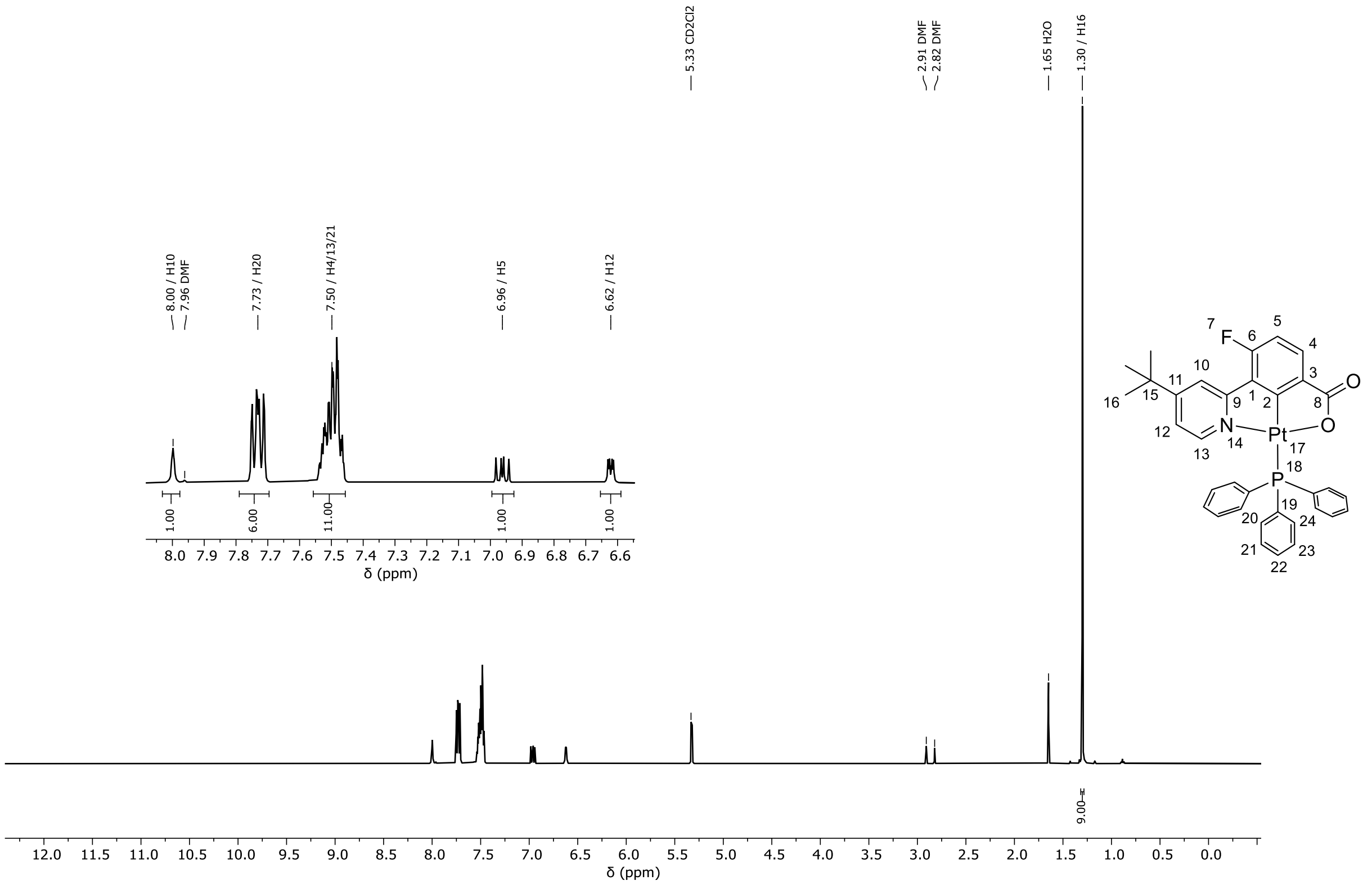

Figure S-87. ^{1}H-NMR spectrum (400 MHz, DCM-d_2) of **Pt(L$_{NCO}$)PPh$_3$**.

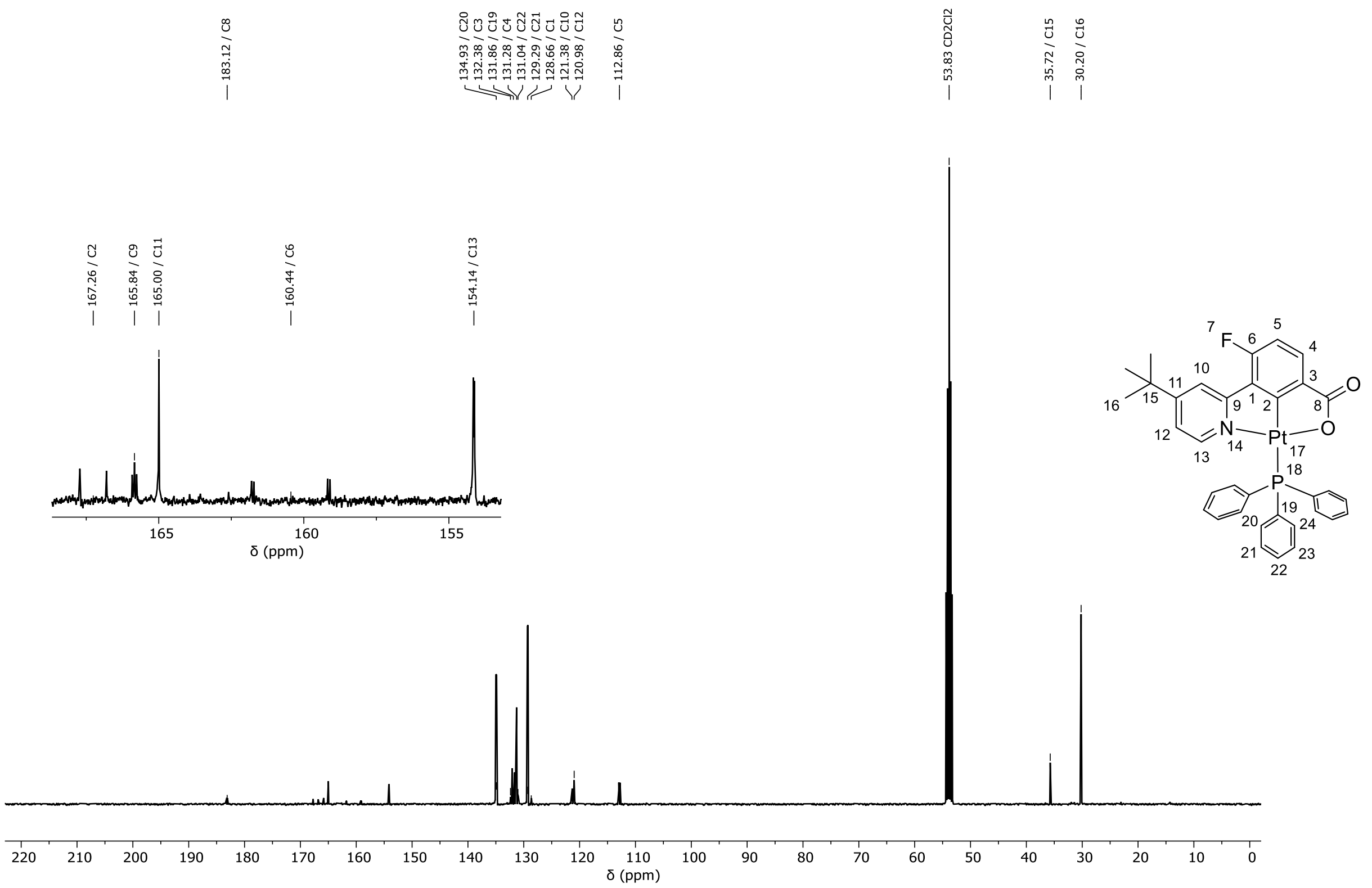

Figure S-88. ^{13}C{^{1}H}-NMR spectrum (101 MHz, DCM-d_2) of **Pt(L$_{NCO}$)PPh$_3$**.

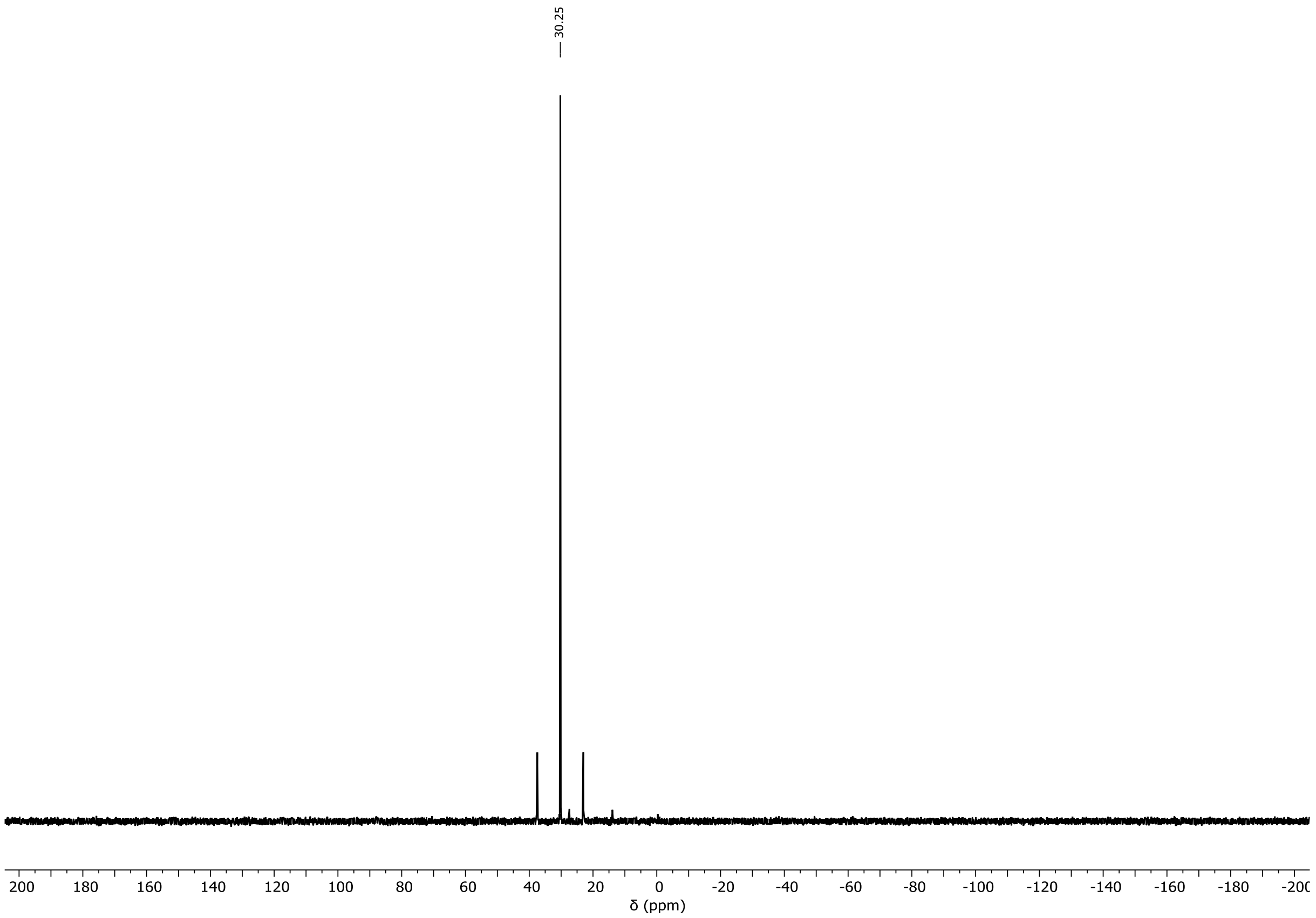

Figure S-89. $^{31}P\{^1H\}$-NMR spectrum (162 MHz, DCM-d_2) of **Pt(L$_{NCO}$)PPh$_3$**.

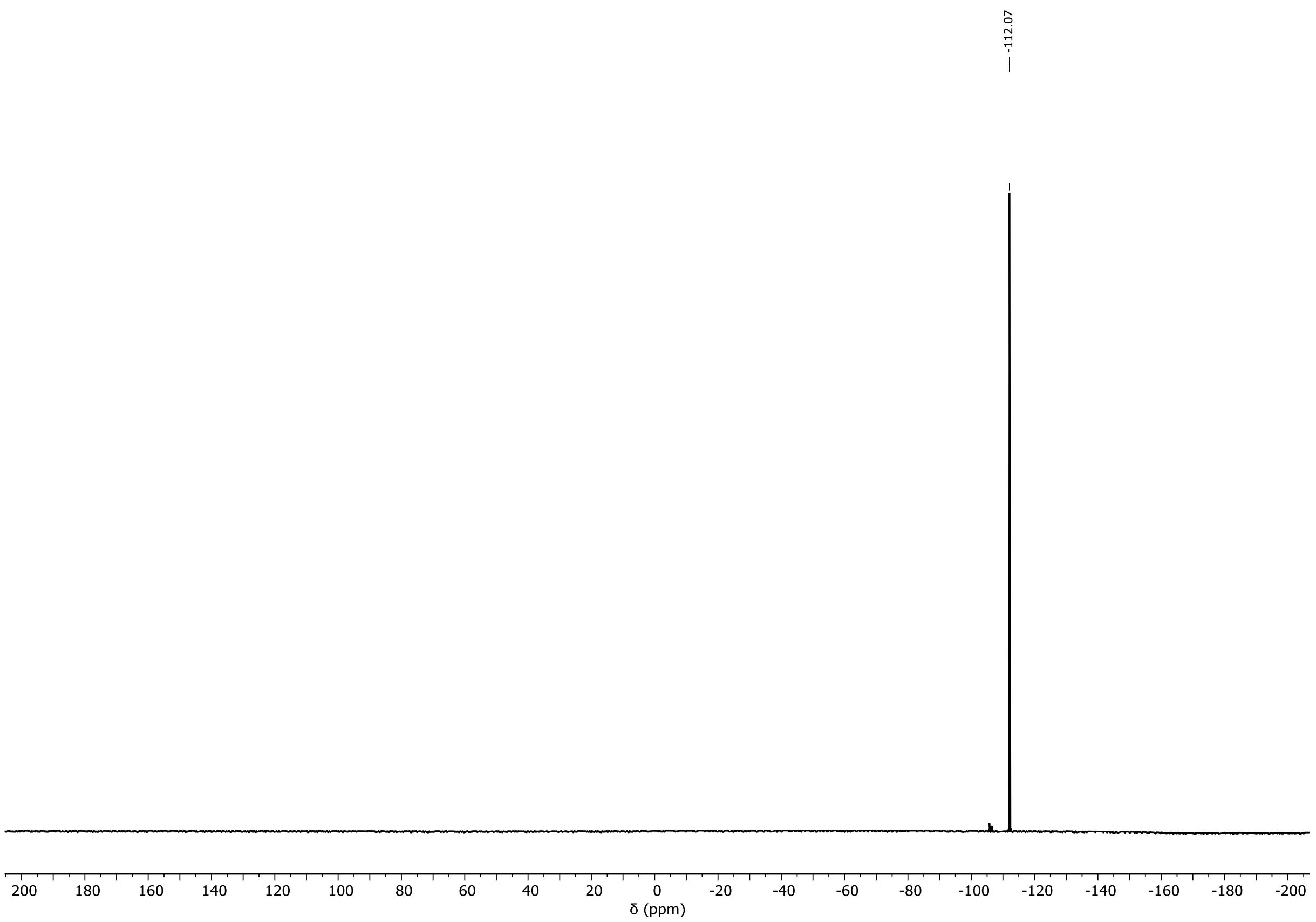

Figure S-90. $^{19}F\{^1H\}$-NMR spectrum (376 MHz, DCM-d_2) of **Pt(L$_{NCO}$)PPh$_3$**.

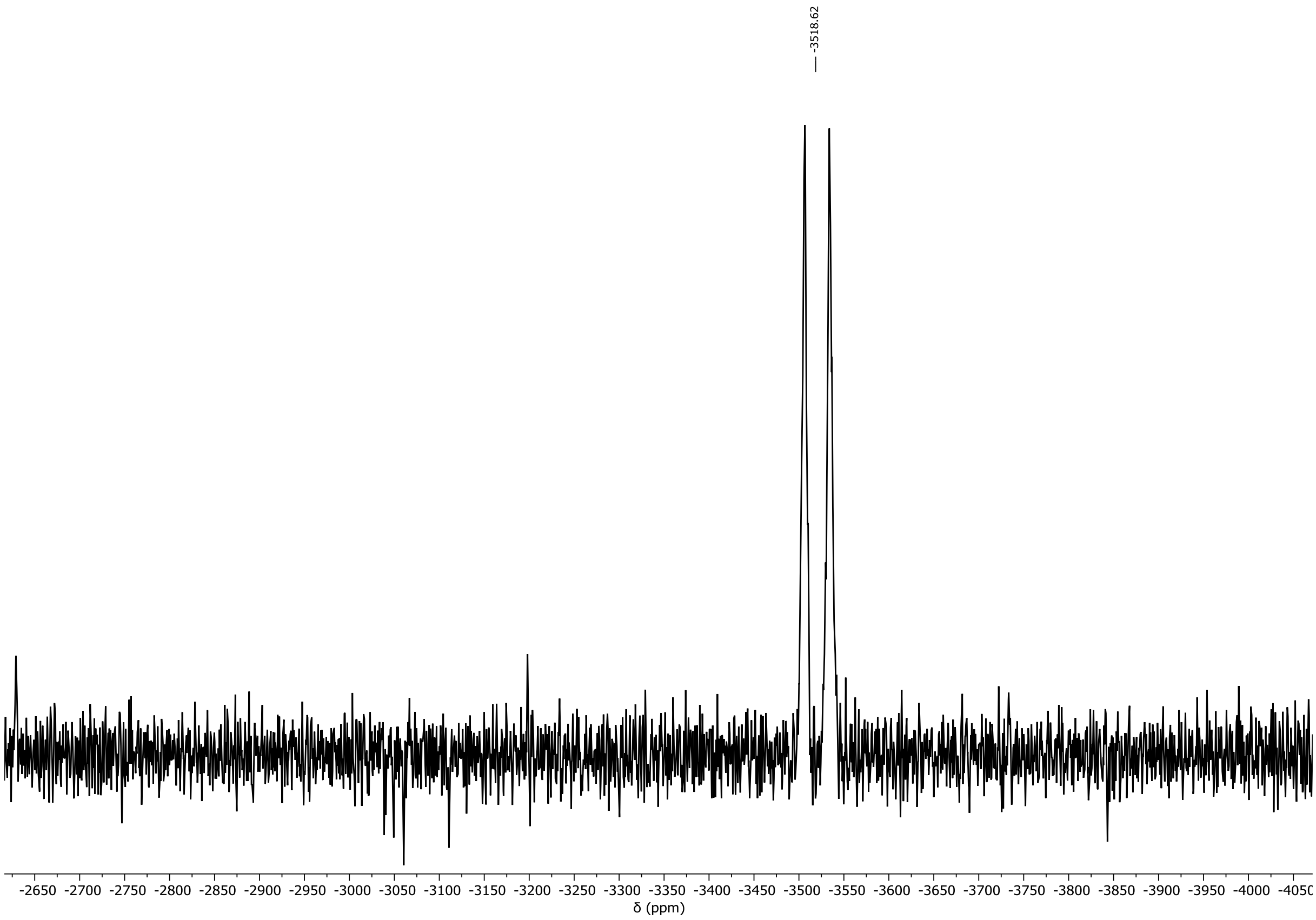

Figure S-91. ^{195}Pt-NMR spectrum (86 MHz, DCM-d_2) of **Pt(L$_{NCO}$)PPh$_3$**.

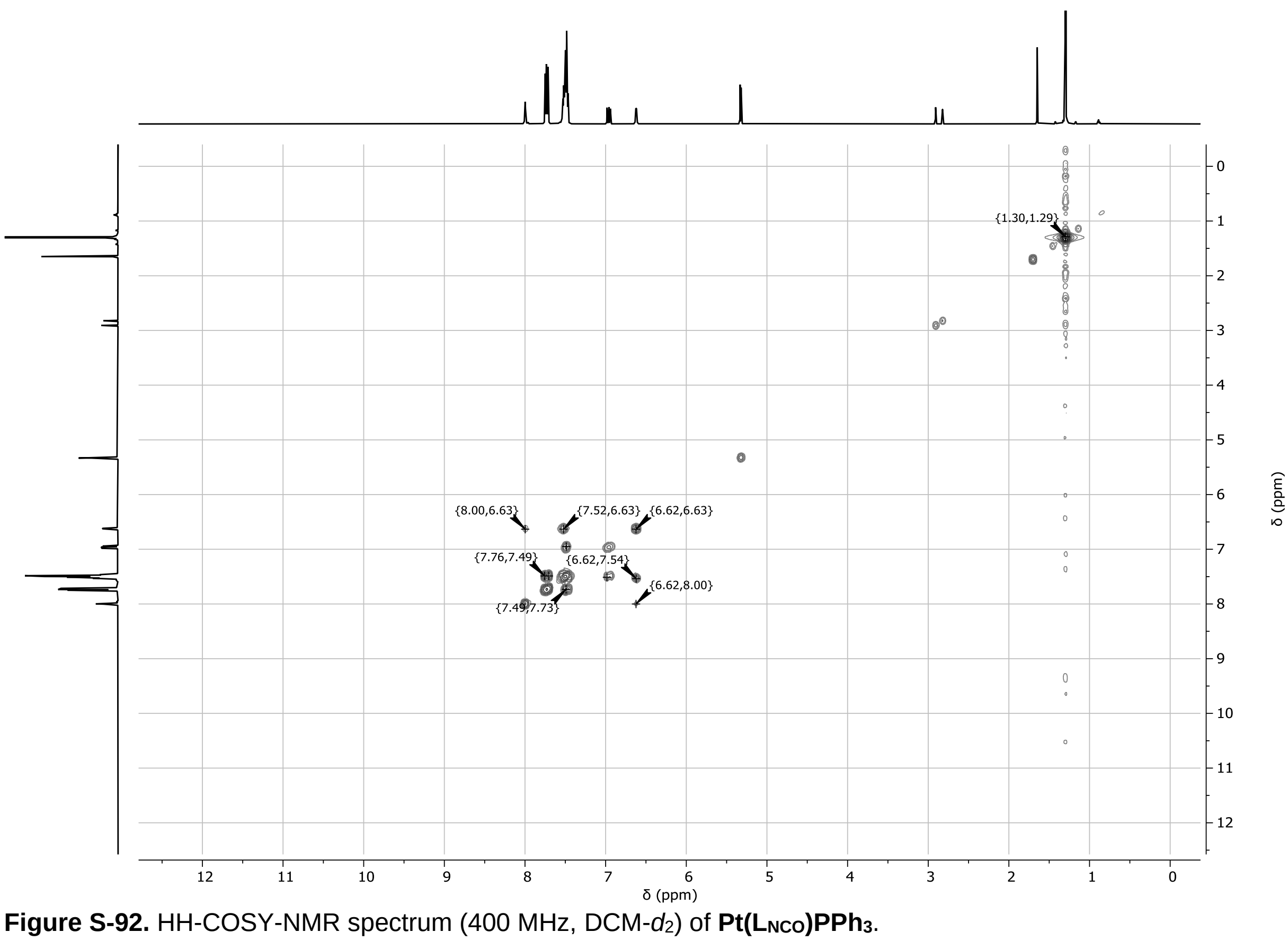

Figure S-92. HH-COSY-NMR spectrum (400 MHz, DCM-d_2) of **Pt(L$_{NCO}$)PPh$_3$**.

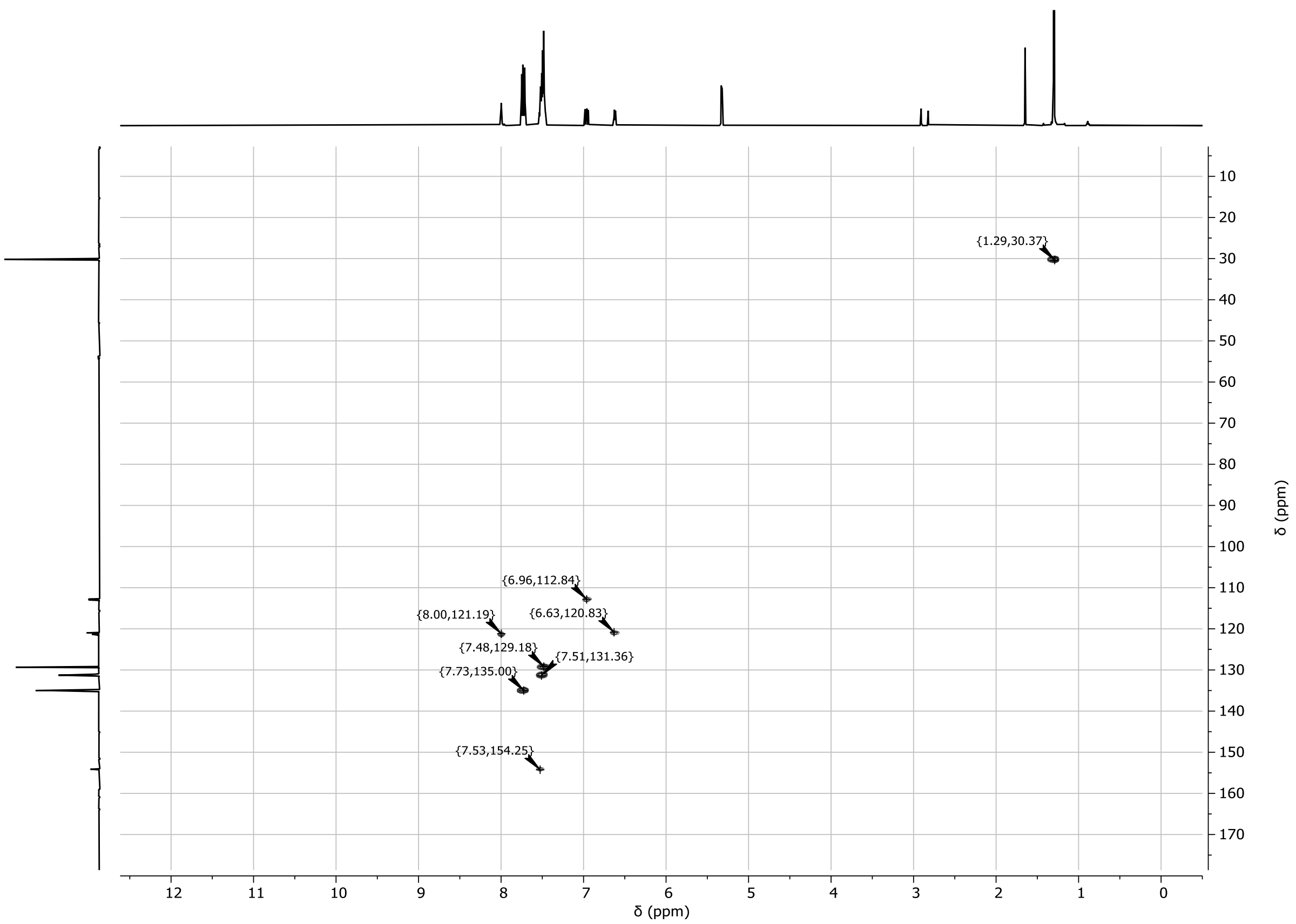

Figure S-93. HC-HSQC-NMR spectrum (101 MHz (^{13}C), 400 MHz (^{1}H), DCM-d_2) of **Pt(L$_{NCO}$)PPh$_3$**.

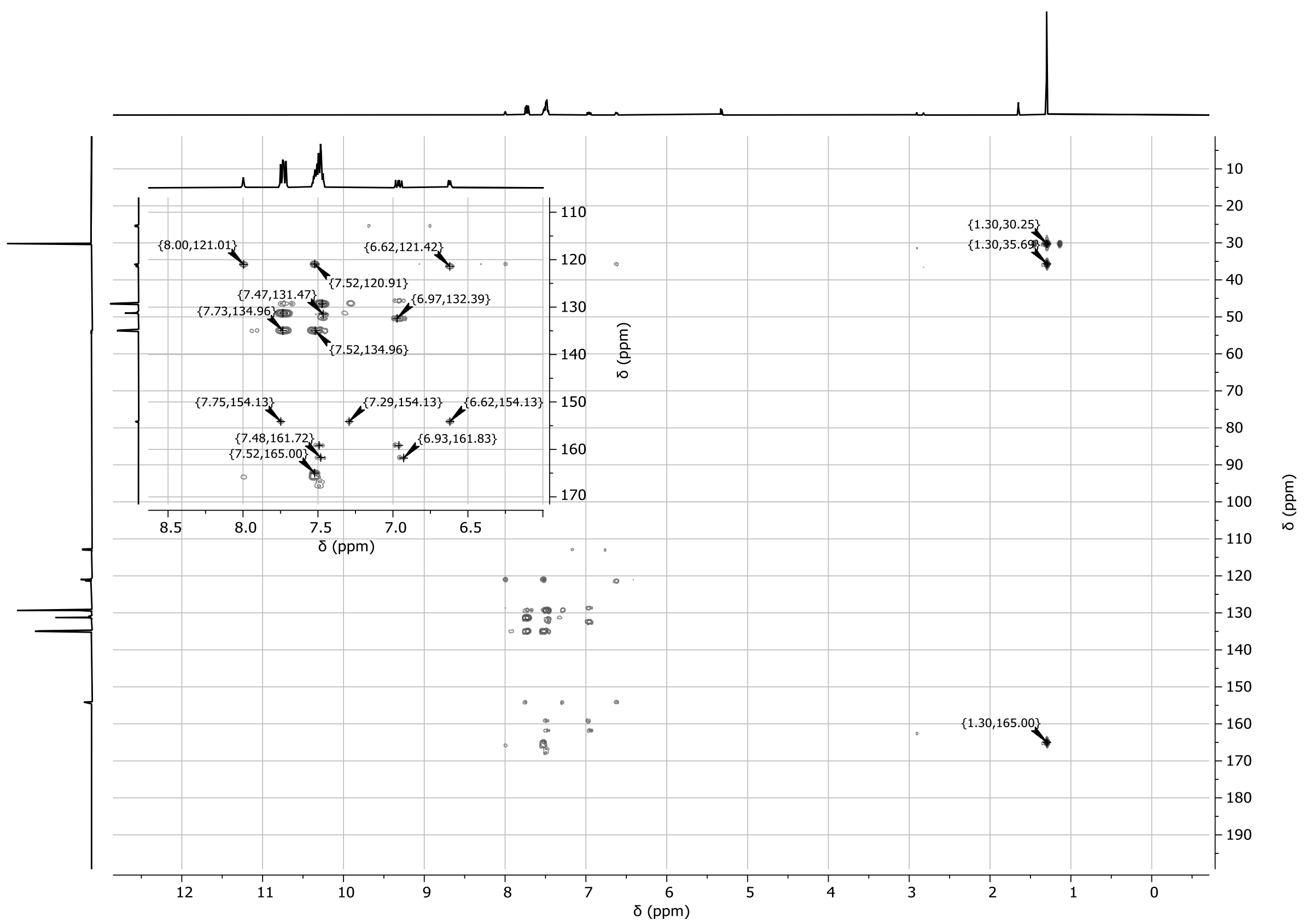

Figure S-94. HC-HMBC-NMR spectrum (101 MHz (^{13}C), 400 MHz (^{1}H), DCM-d_2) of **Pt(L$_{NCO}$)PPh$_3$**.

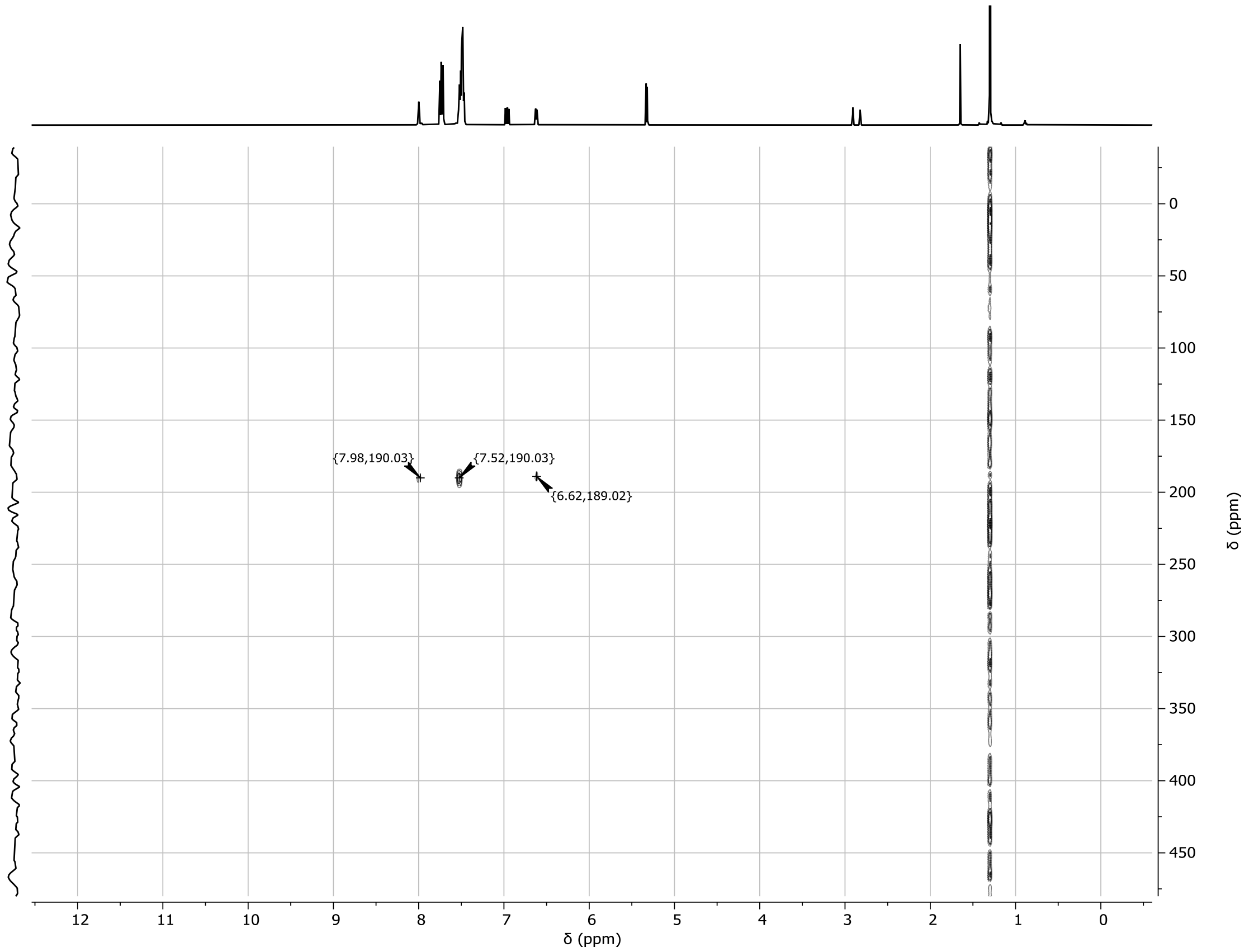

Figure S-95. HN-HMBC-NMR spectrum (41 MHz (^{15}N), 400 MHz (^{1}H), DCM-d_2) of **Pt(L_{NCO})PPh_3**.

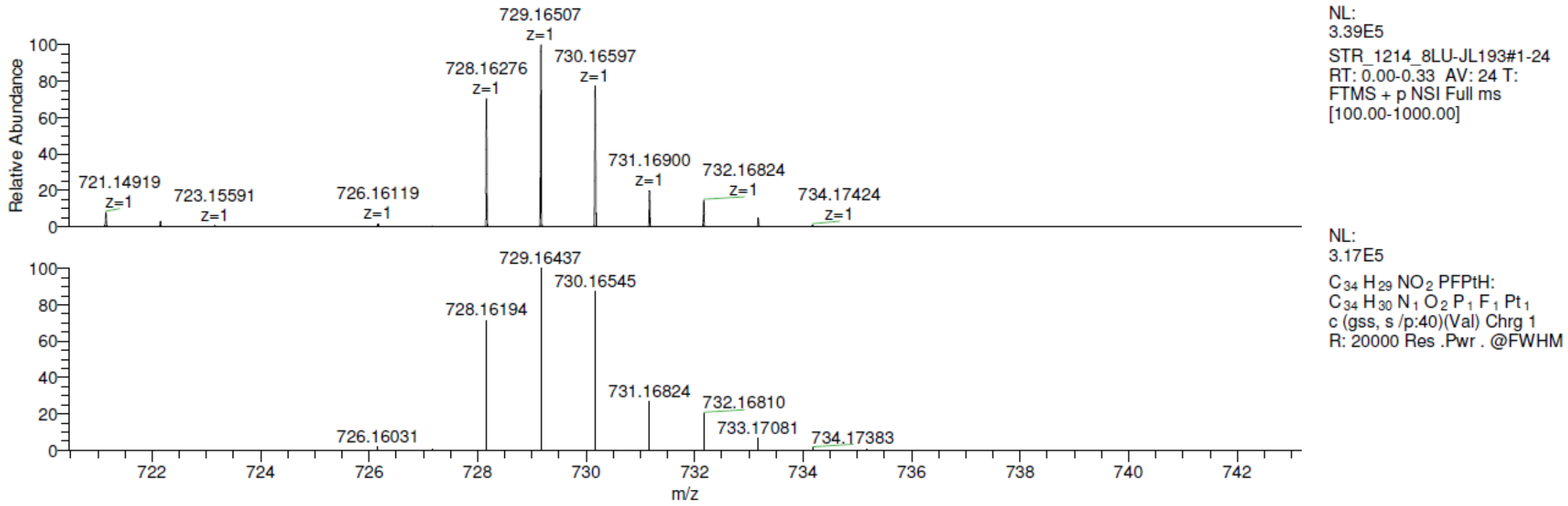

Figure S-96. Mass spectrum of **Pt(L_{NCO})PPh_3** (MeOH). Additional simulation of the [**Pt(L_{NCO})PPh_3**+H]$^+$ adduct.

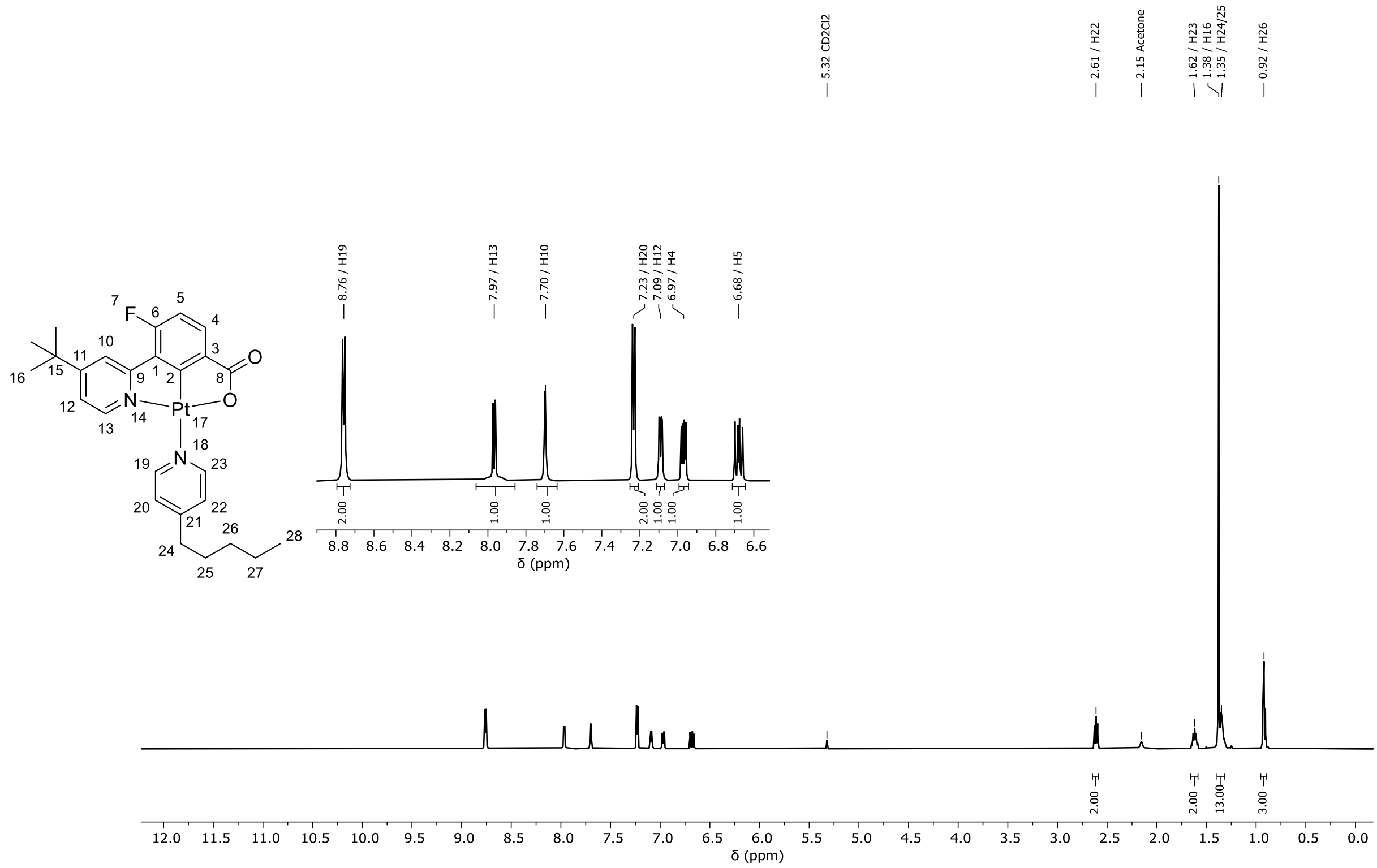

Figure S-97. ^{1}H-NMR spectrum (500 MHz, DCM-d_2) of **Pt(L$_{NCO}$)AmPy**.

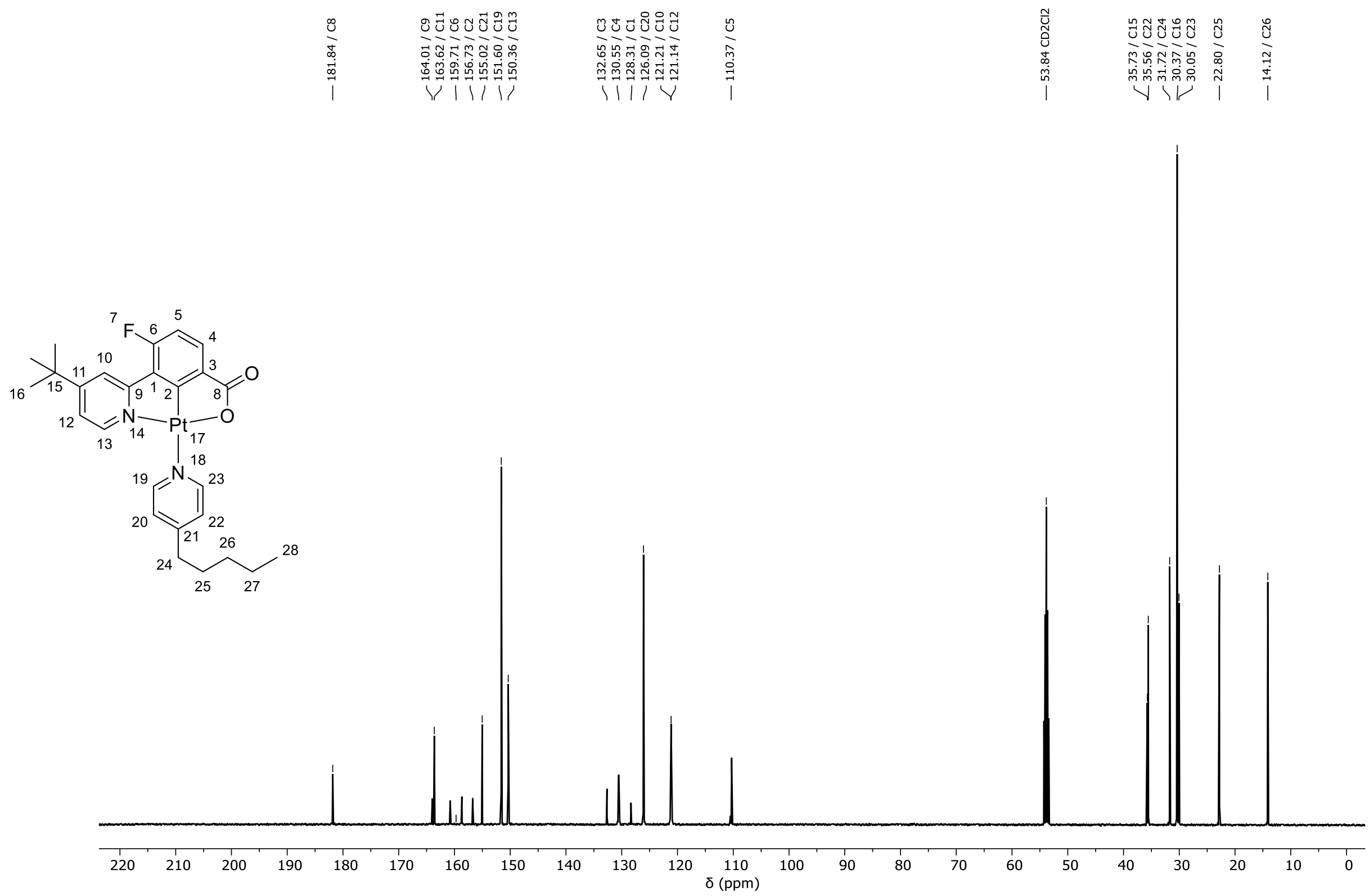

Figure S-98. ^{13}C{^{1}H}-NMR spectrum (126 MHz, DCM-d_2) of **Pt(L$_{NCO}$)AmPy**.

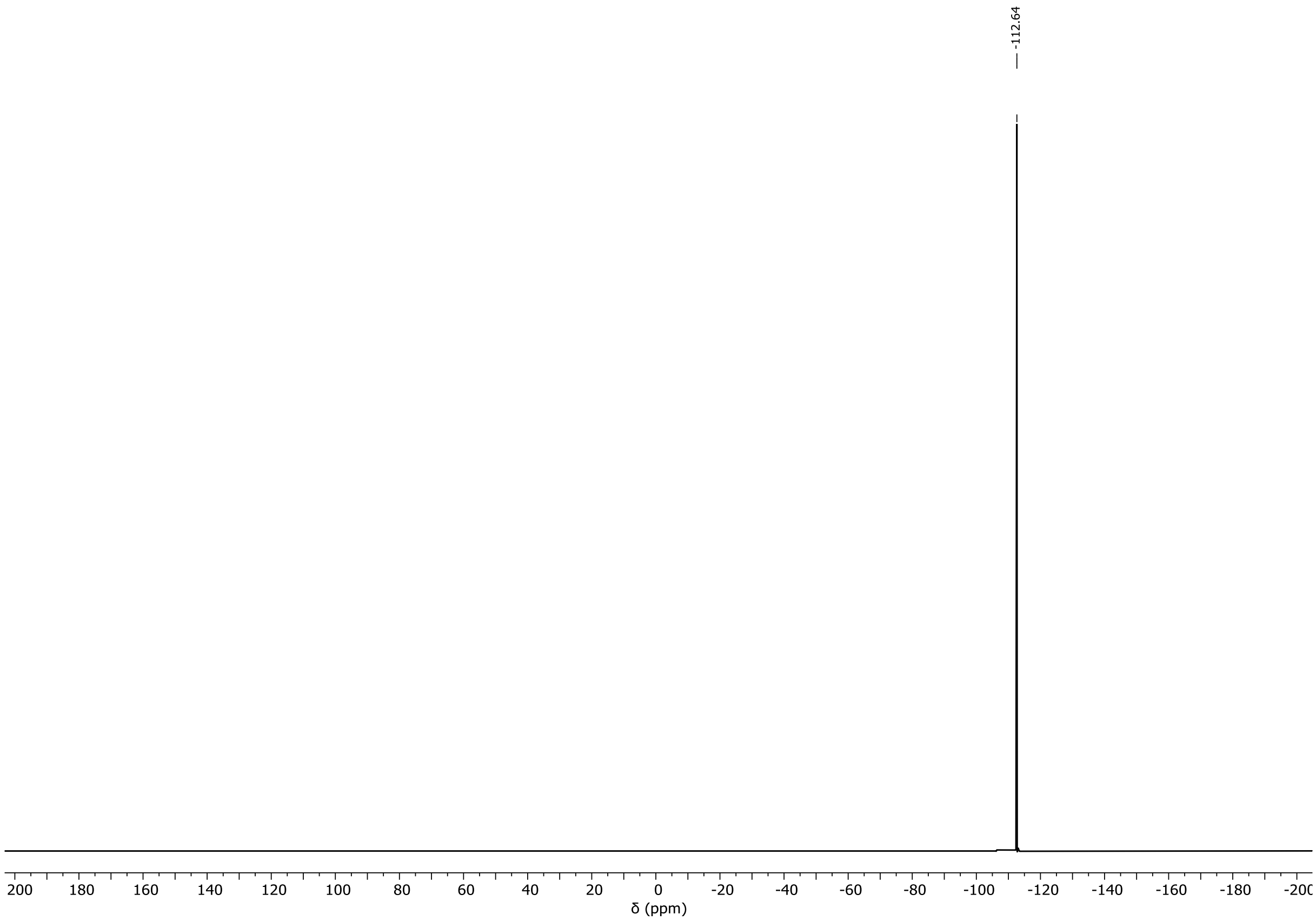

Figure S-99. $^{19}F\{^{1}H\}$-NMR spectrum (471 MHz, DCM-d_2) of **Pt(L_{NCO})PPh_3**.

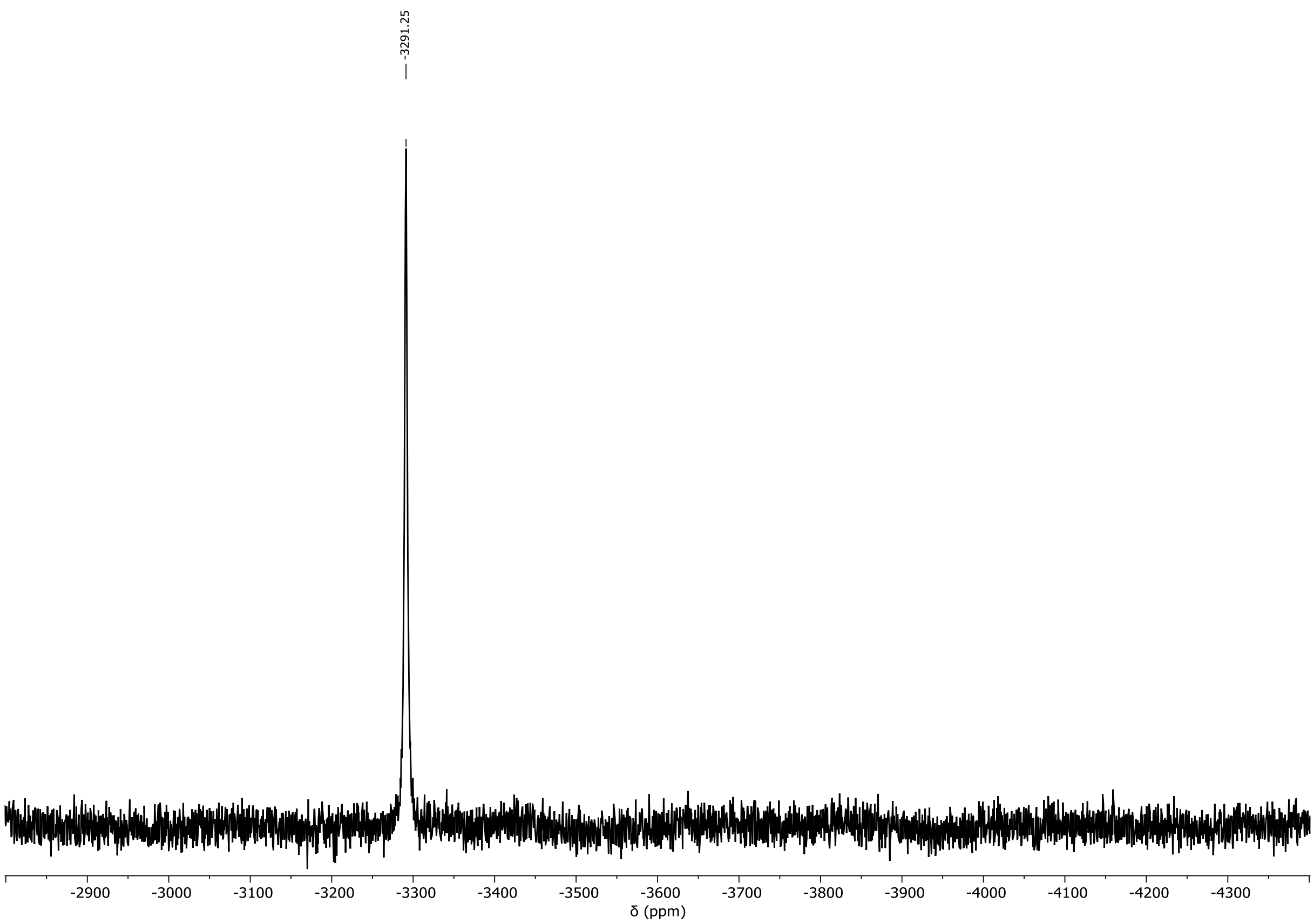

Figure S-100. ^{195}Pt-NMR spectrum (107 MHz, DCM-d_2) of **Pt(L_{NCO})AmPy**.

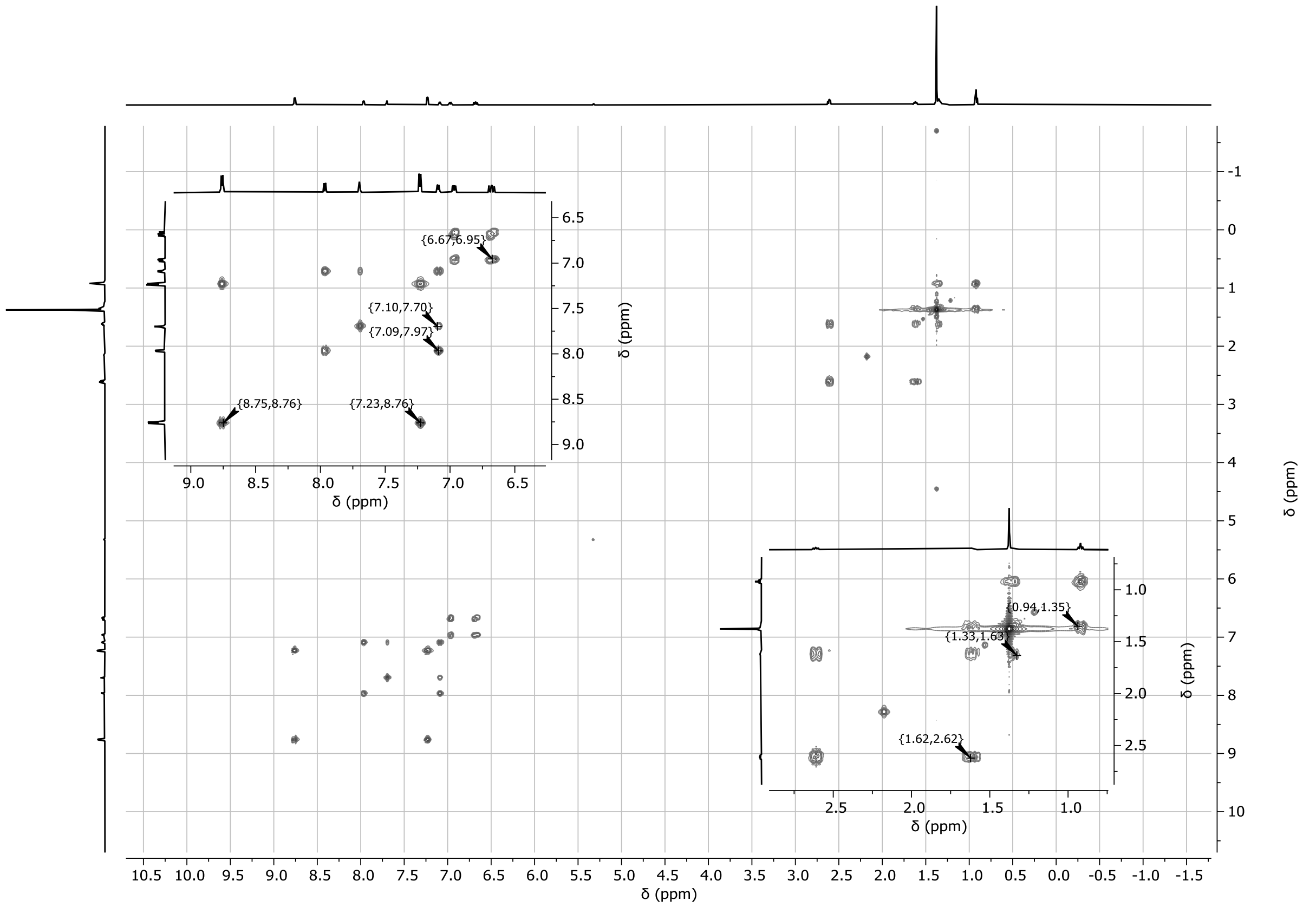

Figure S-101. HC-COSY-NMR spectrum (500 MHz, DCM-d_2) of **Pt(L$_{NCO}$)AmPy**.

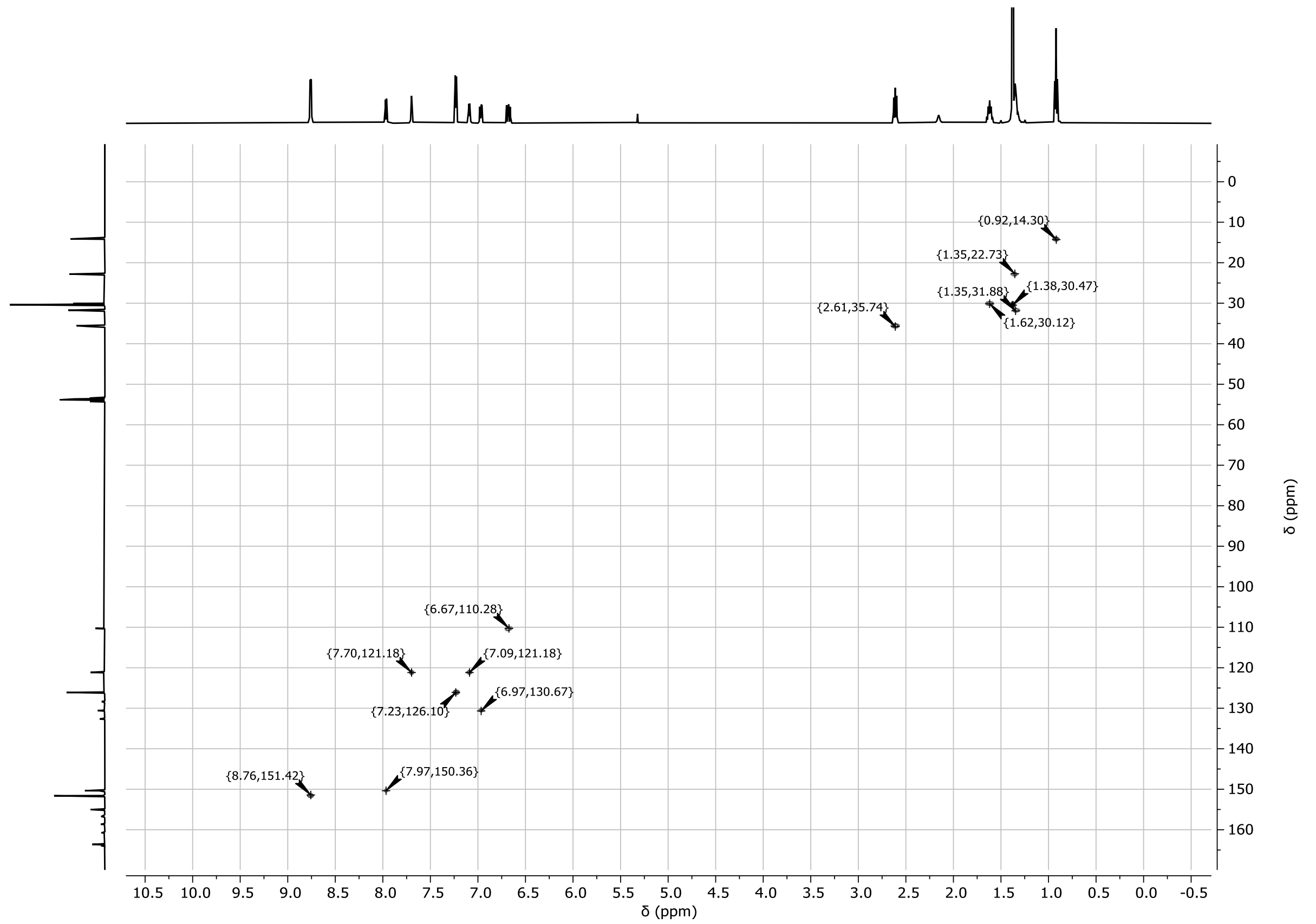

Figure S-102. HC-HSQC-NMR spectrum (126 MHz (^{13}C), 500 MHz (^{1}H), DCM-d_2) of **Pt(L$_{NCO}$)AmPy**.

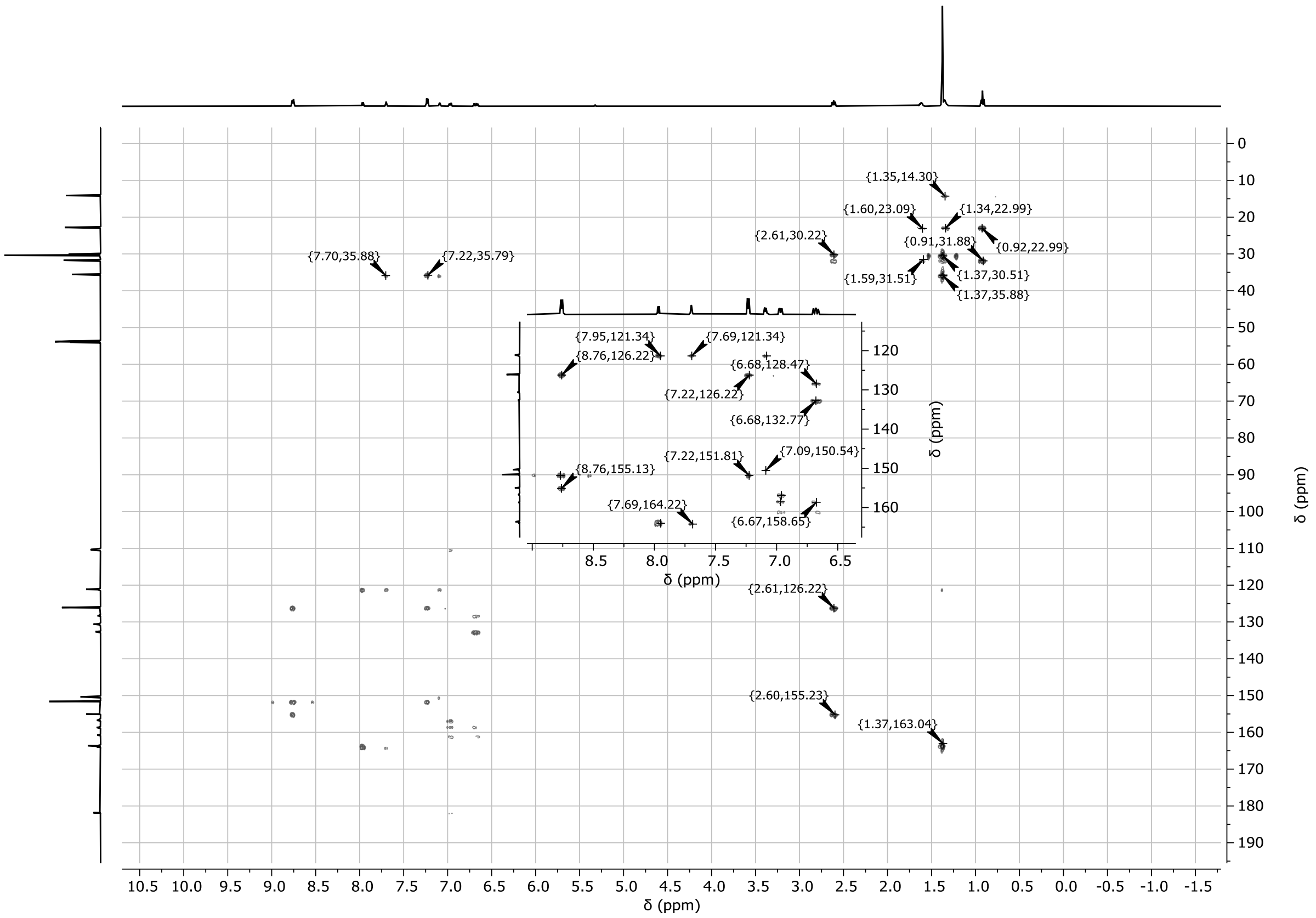

Figure S-103. HC-HMBC-NMR spectrum (126 MHz (^{13}C), 500 MHz (^{1}H), DCM-d_2) of **Pt(L_{NCO})AmPy**.

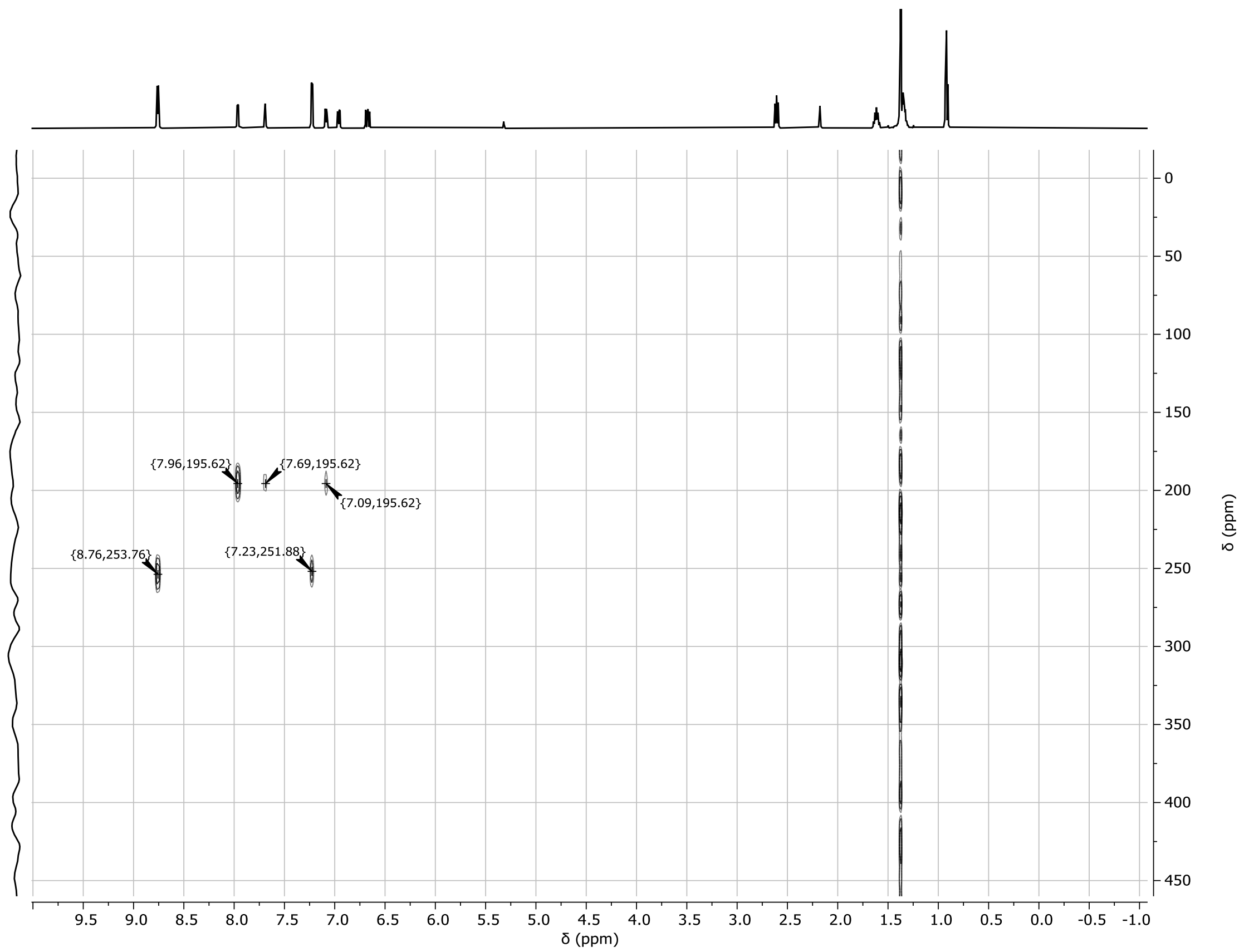

Figure S-104. HN-HMBC-NMR spectrum (51 MHz (^{15}N), 500 MHz (^{1}H), DCM-d_2) of **Pt(L_{NCO})AmPy**.

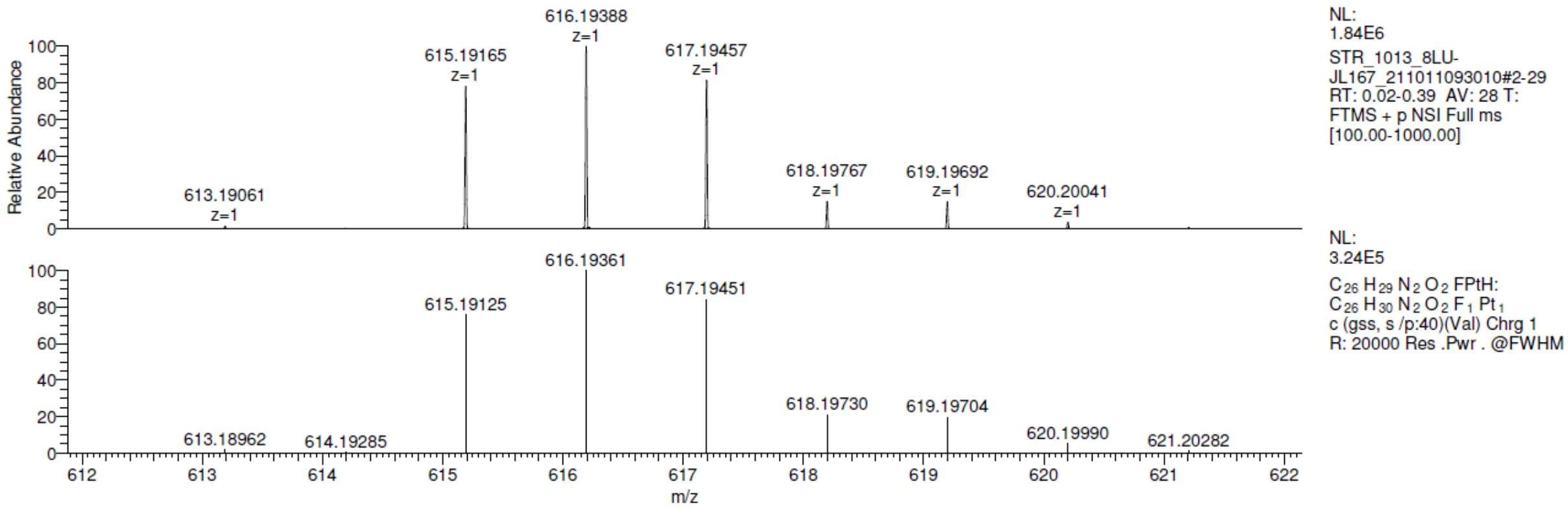

Figure S-105. Mass spectrum of **Pt(L$_{NCO}$)AmPy** (MeOH). Additional simulation of the [**Pt(L$_{NCO}$)AmPy**+H]$^+$ adduct.

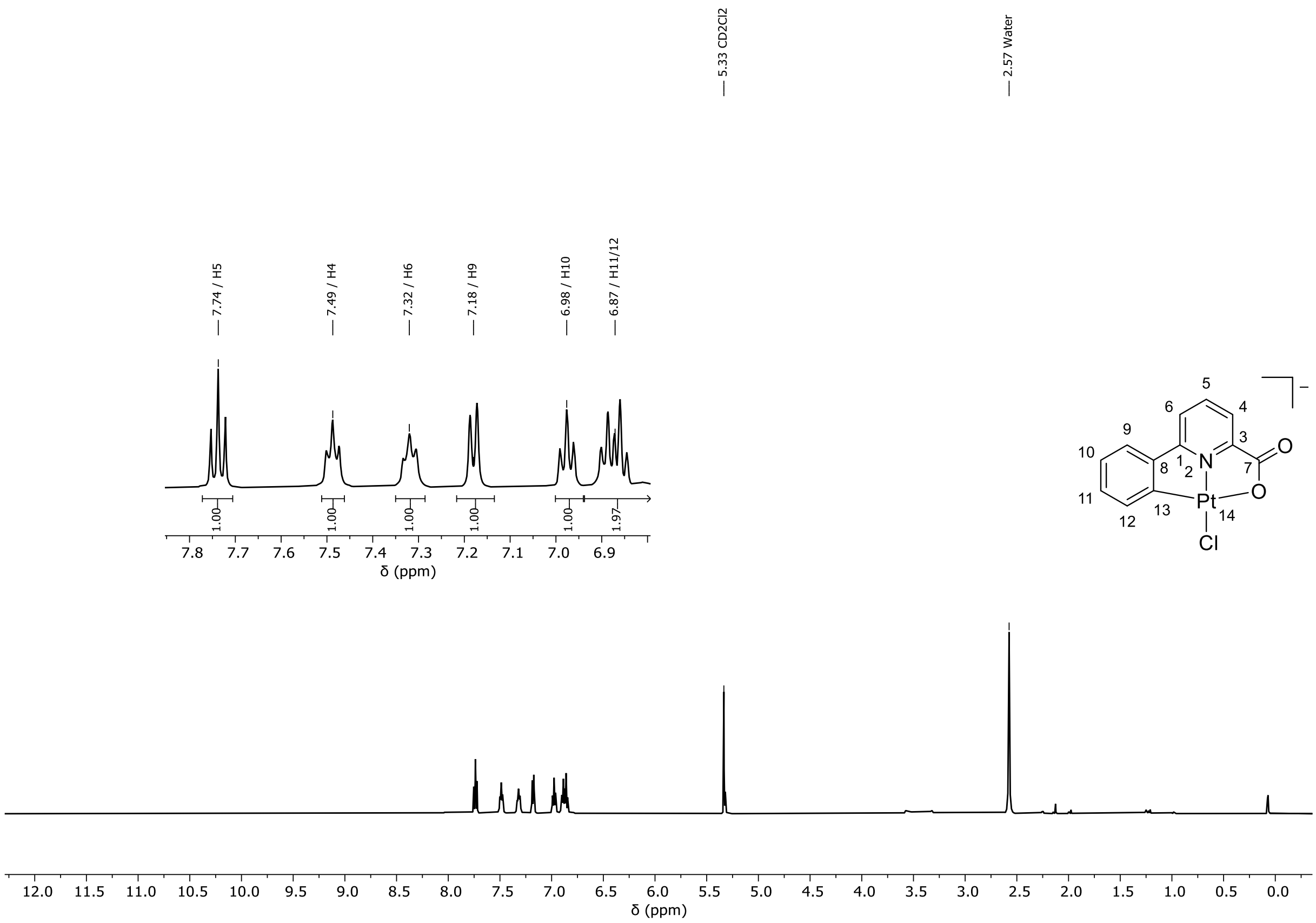

Figure S-106. ^{1}H-NMR spectrum (500 MHz, DCM-d_2) of **Pt(L$_H$)Cl**.

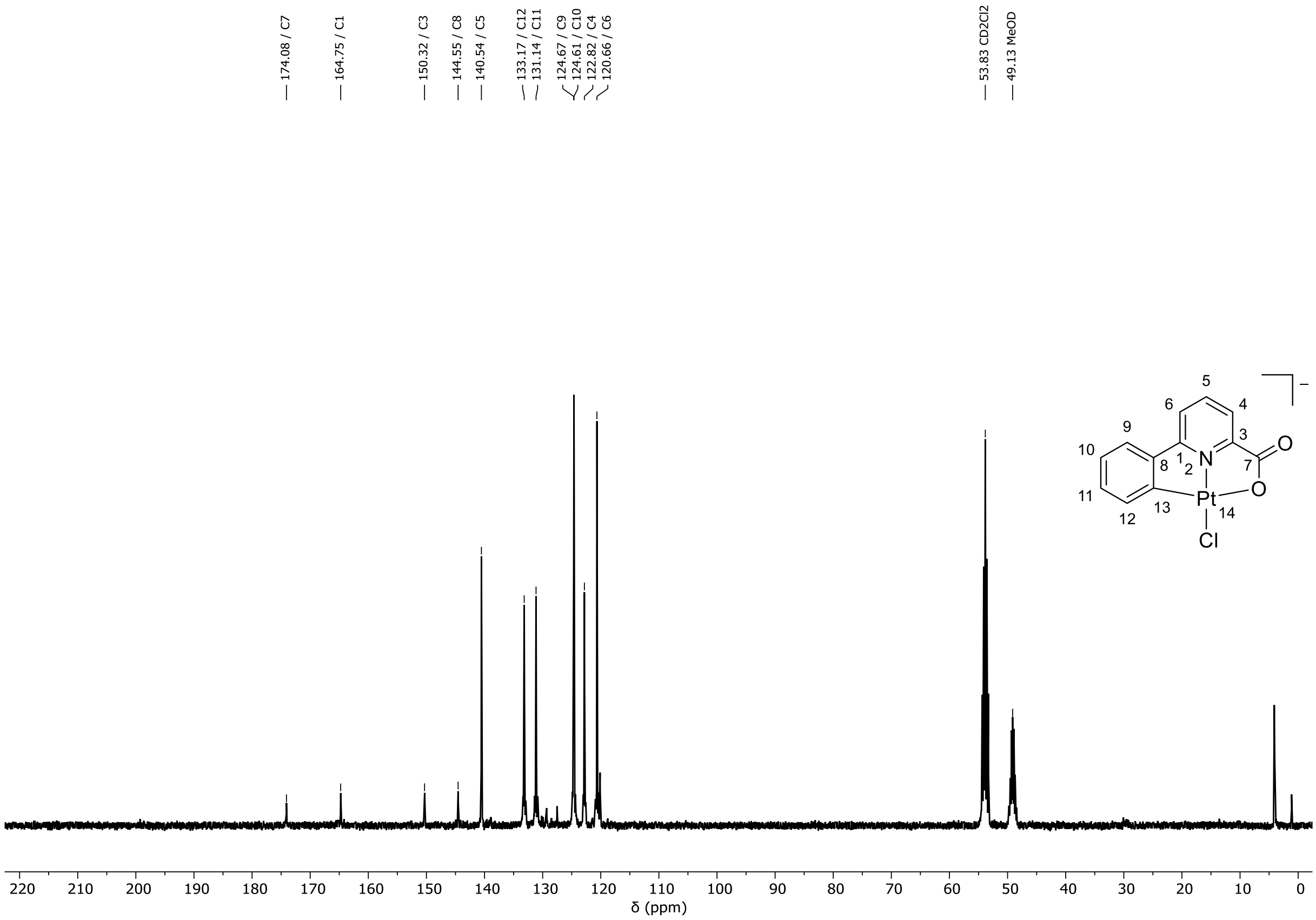

Figure S-107. $^{13}C\{^{1}H\}$-NMR spectrum (126 MHz, DCM-d_2) of **Pt(L$_H$)Cl**.

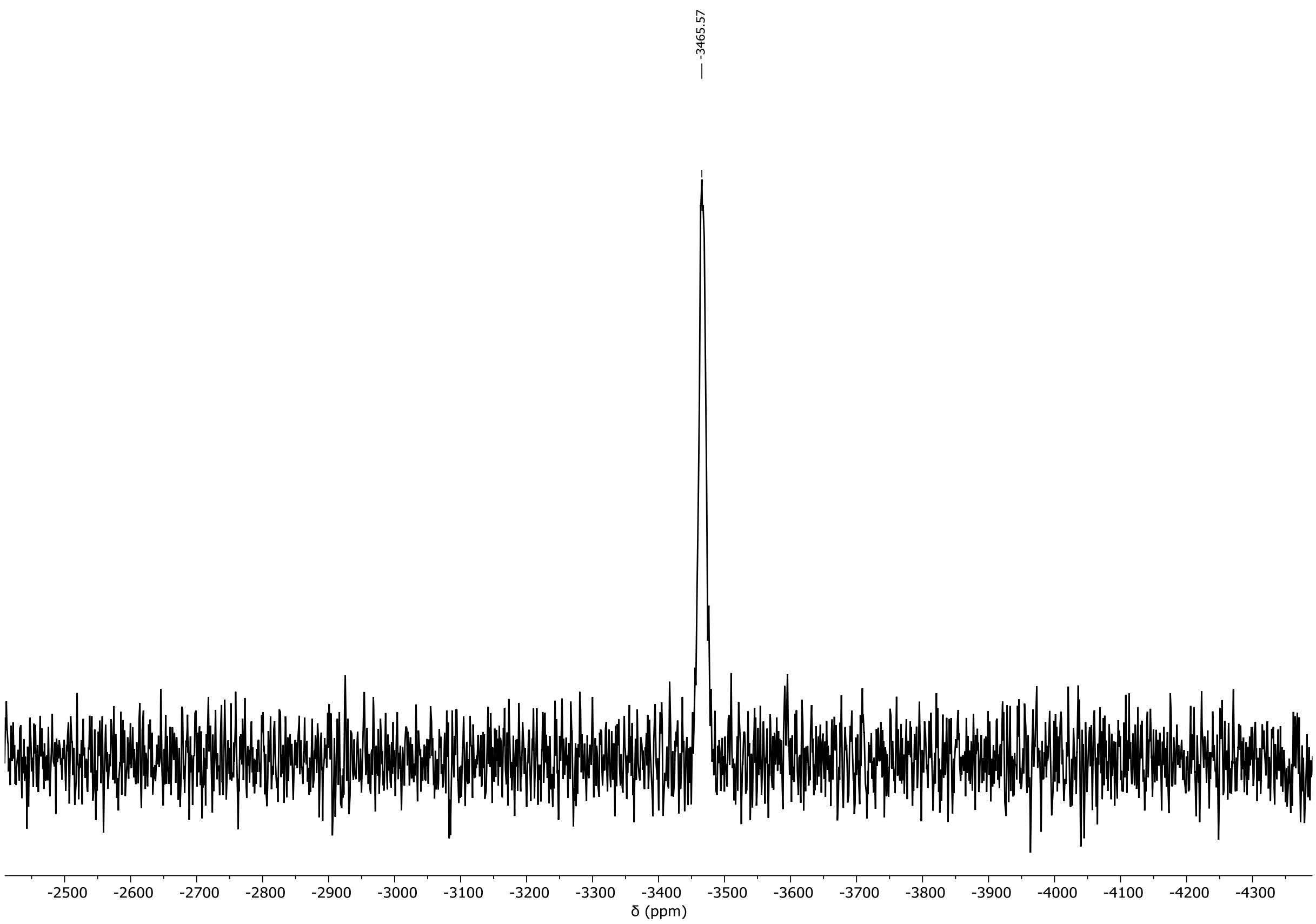

Figure S-108. ^{195}Pt-NMR spectrum (107 MHz, DCM-d_2) of **Pt(L$_H$)Cl**.

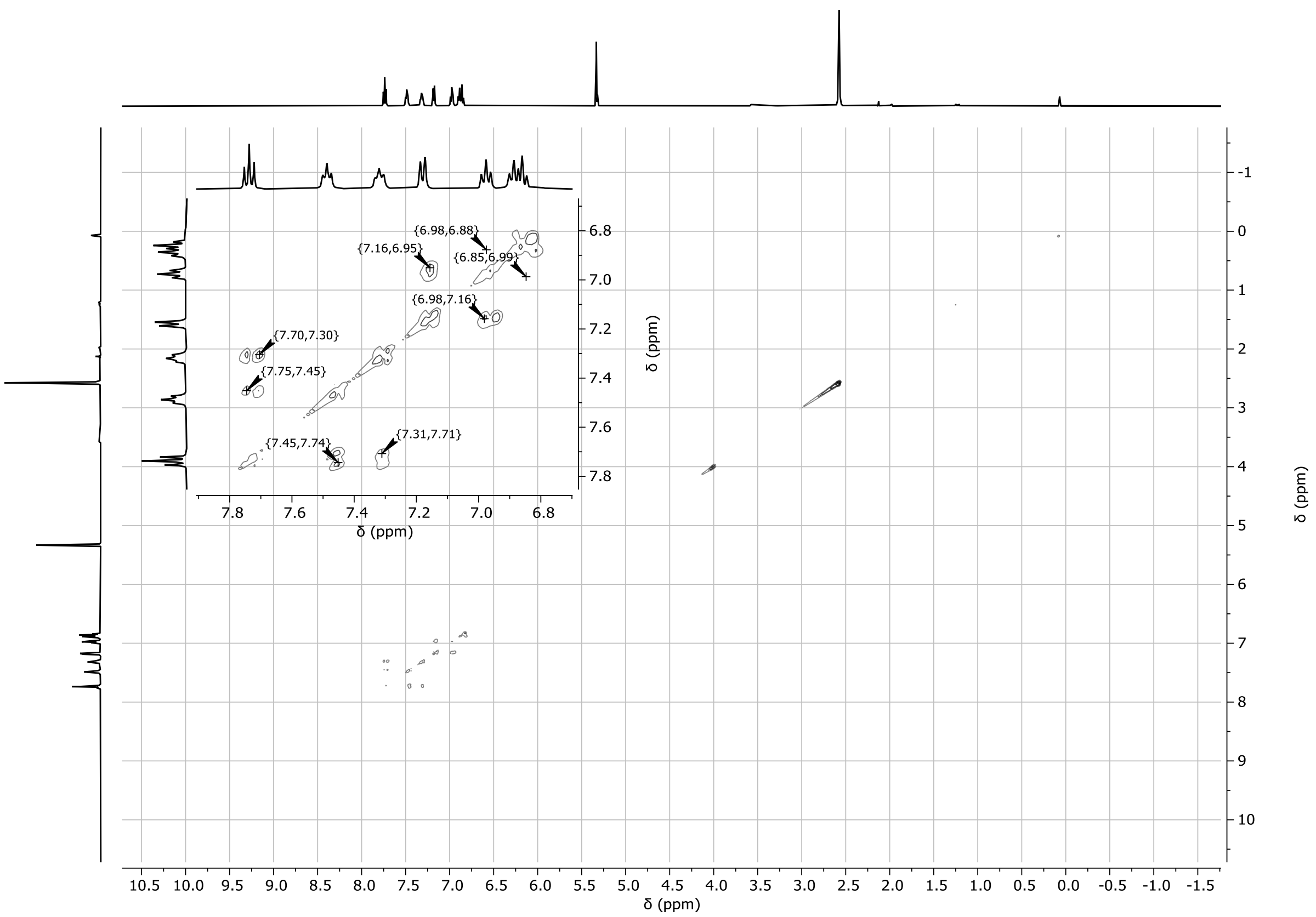

Figure S-109. HC-COSY-NMR spectrum (500 MHz, DCM-d_2) of **Pt(L$_H$)Cl**.

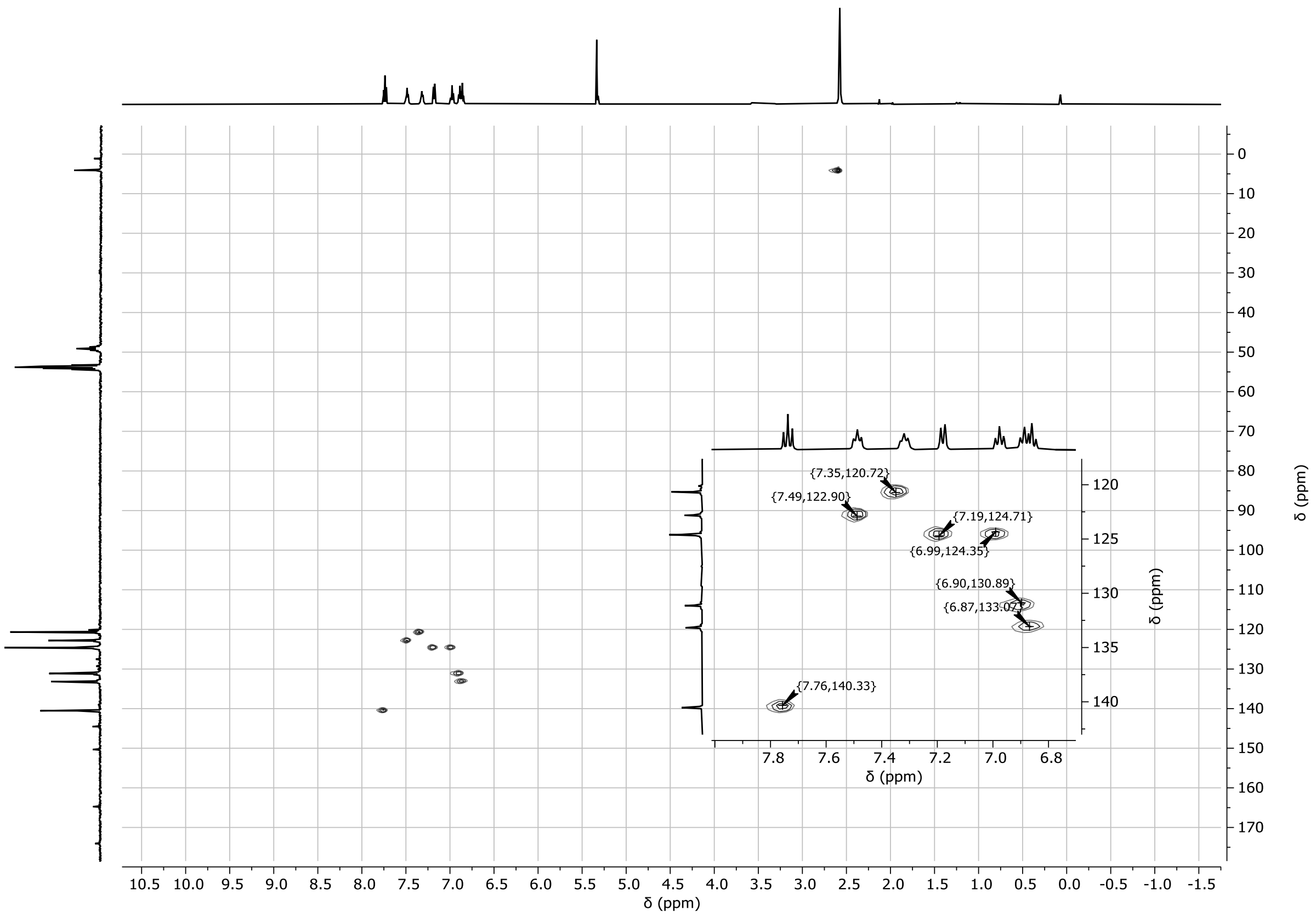

Figure S-110. HC-HSQC-NMR spectrum (126 MHz (^{13}C), 500 MHz (^{1}H), DCM-d_2) of **Pt(L$_H$)Cl**.

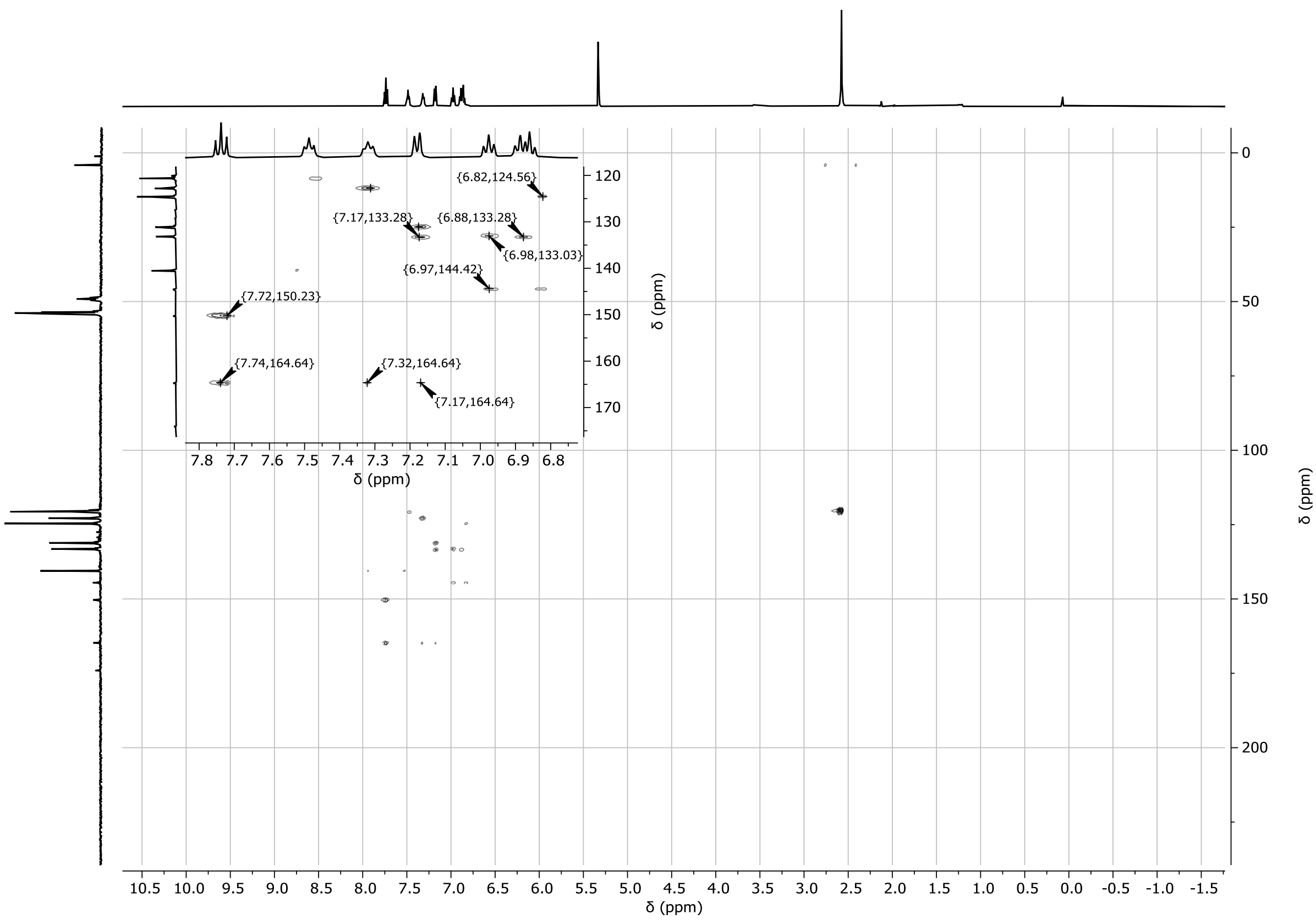

Figure S-111. HC-HMBC-NMR spectrum (126 MHz (^{13}C), 500 MHz (^{1}H), DCM-d_2) of **Pt(L$_H$)Cl**.

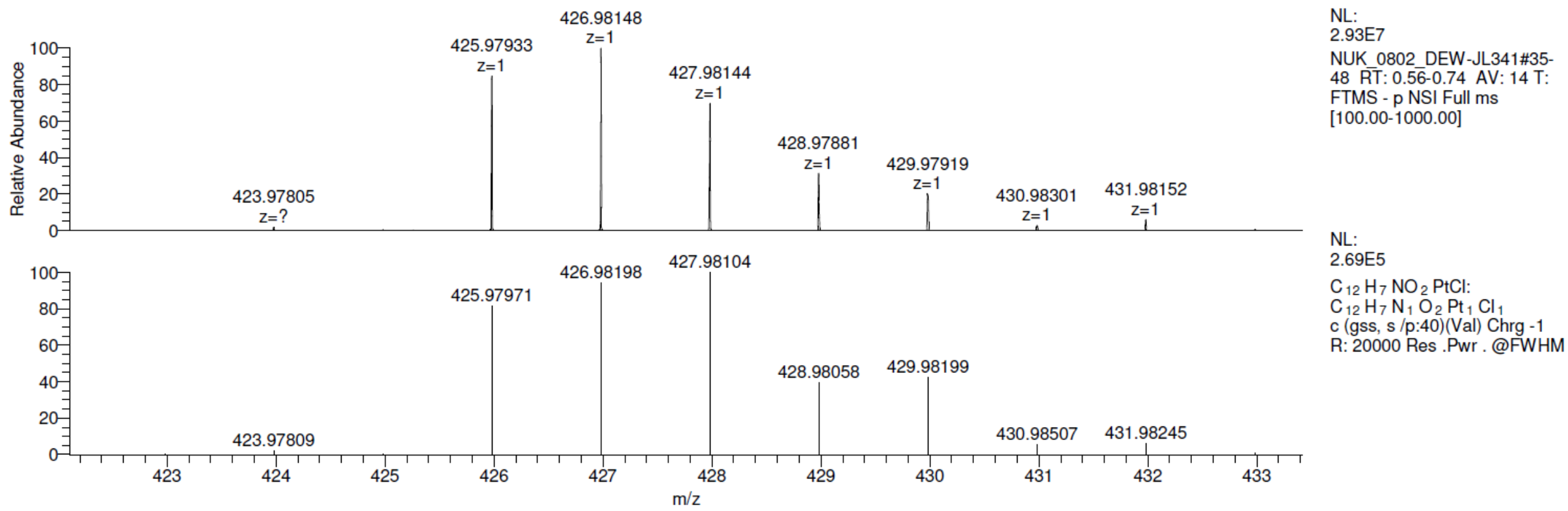

Figure S-112. Mass spectrum of **Pt(L$_H$)Cl** (MeOH). Additional simulation of the [**Pt(L$_H$)Cl**]$^-$ adduct.

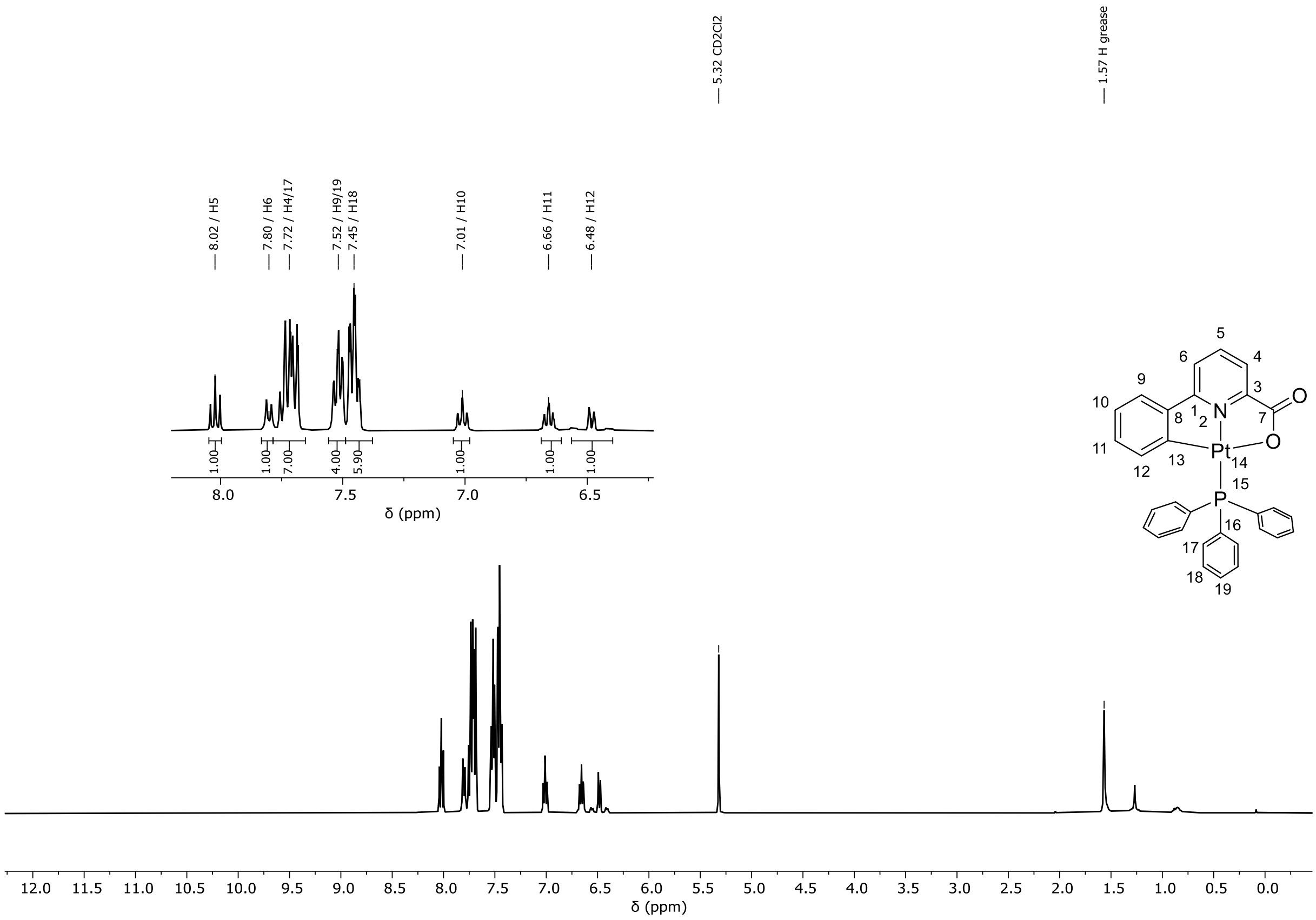

Figure S-113. ^{1}H-NMR spectrum (500 MHz, DCM-d_2) of **Pt(L$_H$)PPh$_3$**.

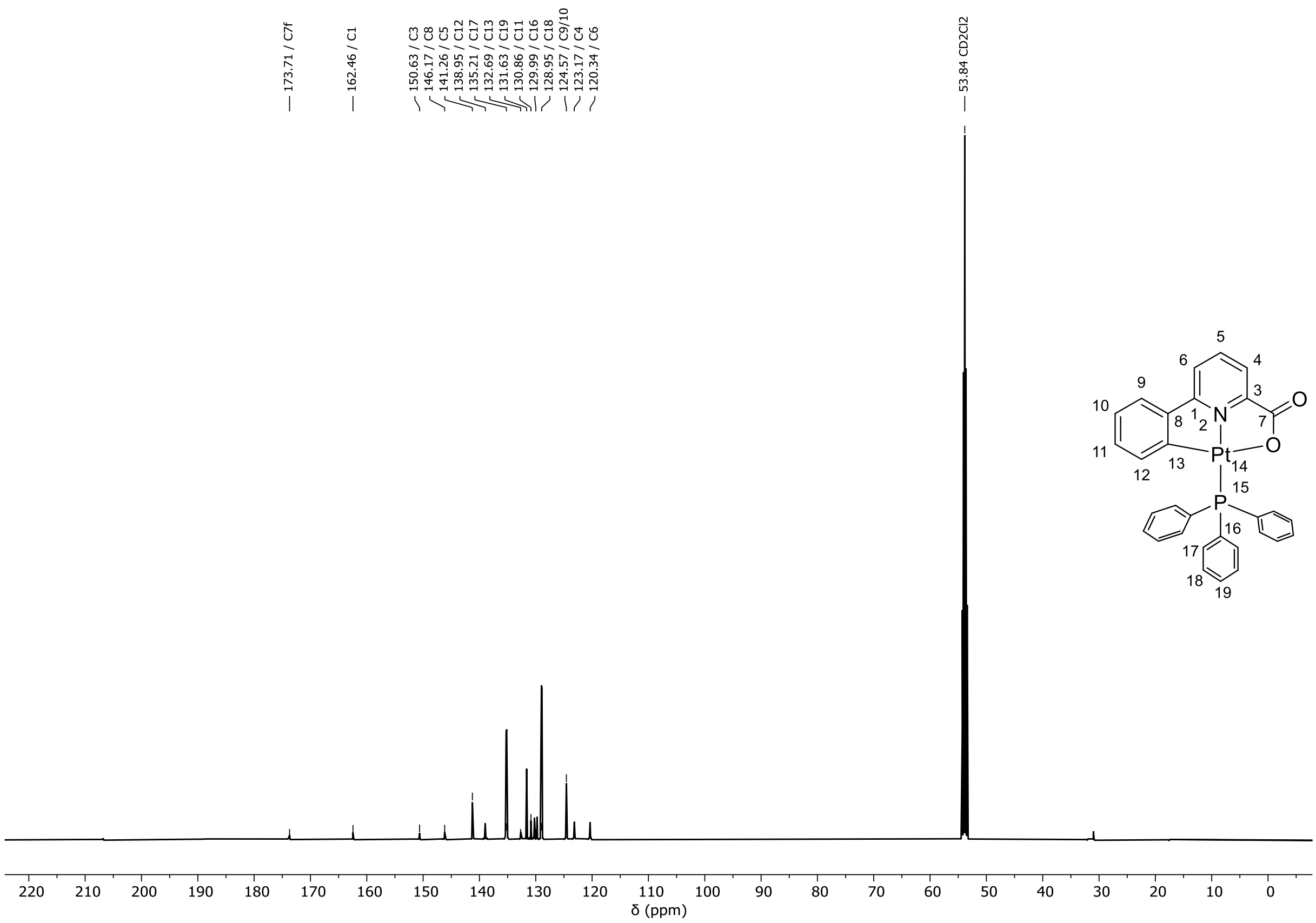

Figure S-114. ^{13}C{^{1}H}-NMR spectrum (126 MHz, DCM-d_2) of **Pt(L$_H$)PPh$_3$**.

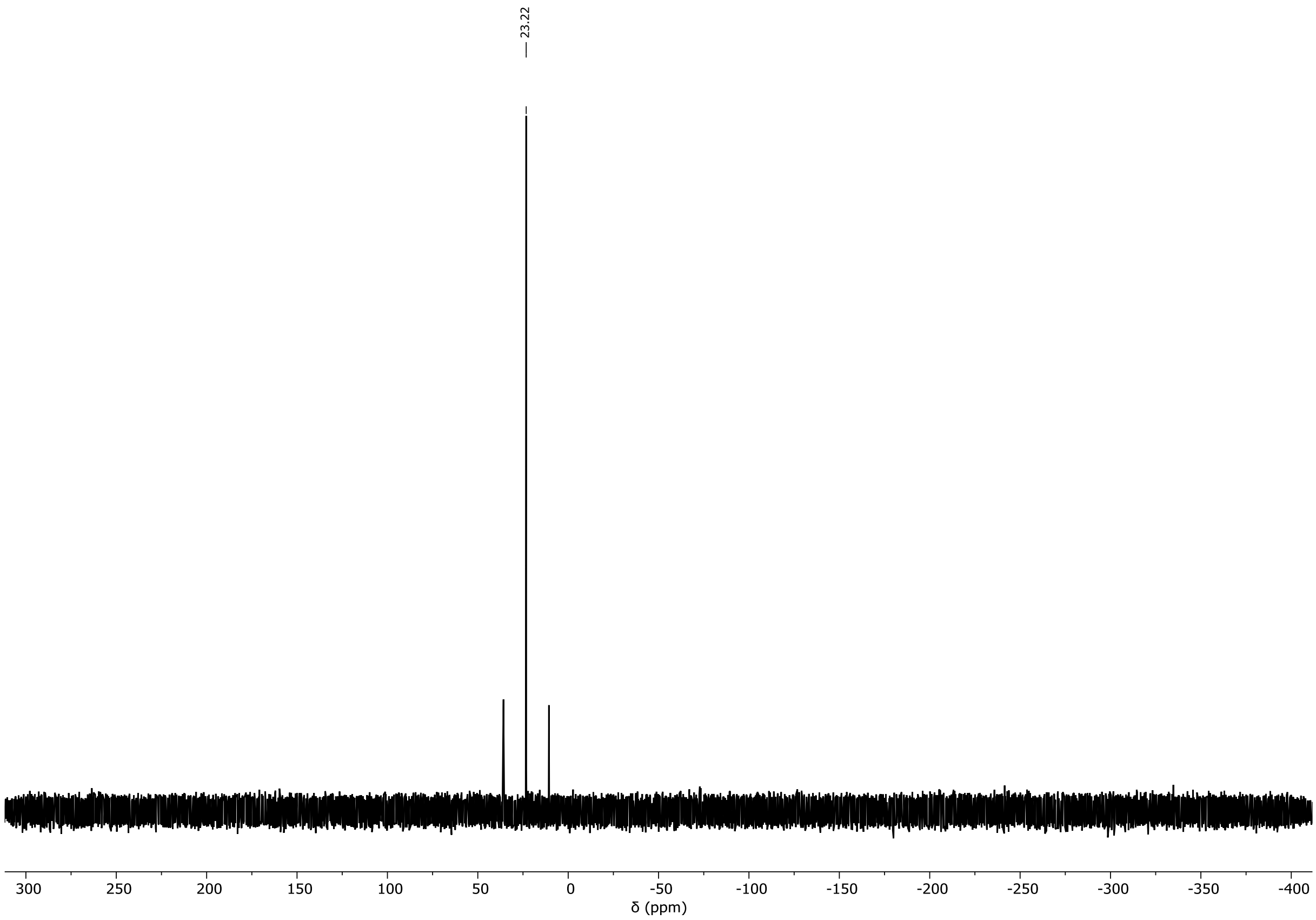

Figure S-115. $^{31}P\{^1H\}$-NMR spectrum (162 MHz, DCM-d_2) of **Pt(L$_H$)PPh$_3$**.

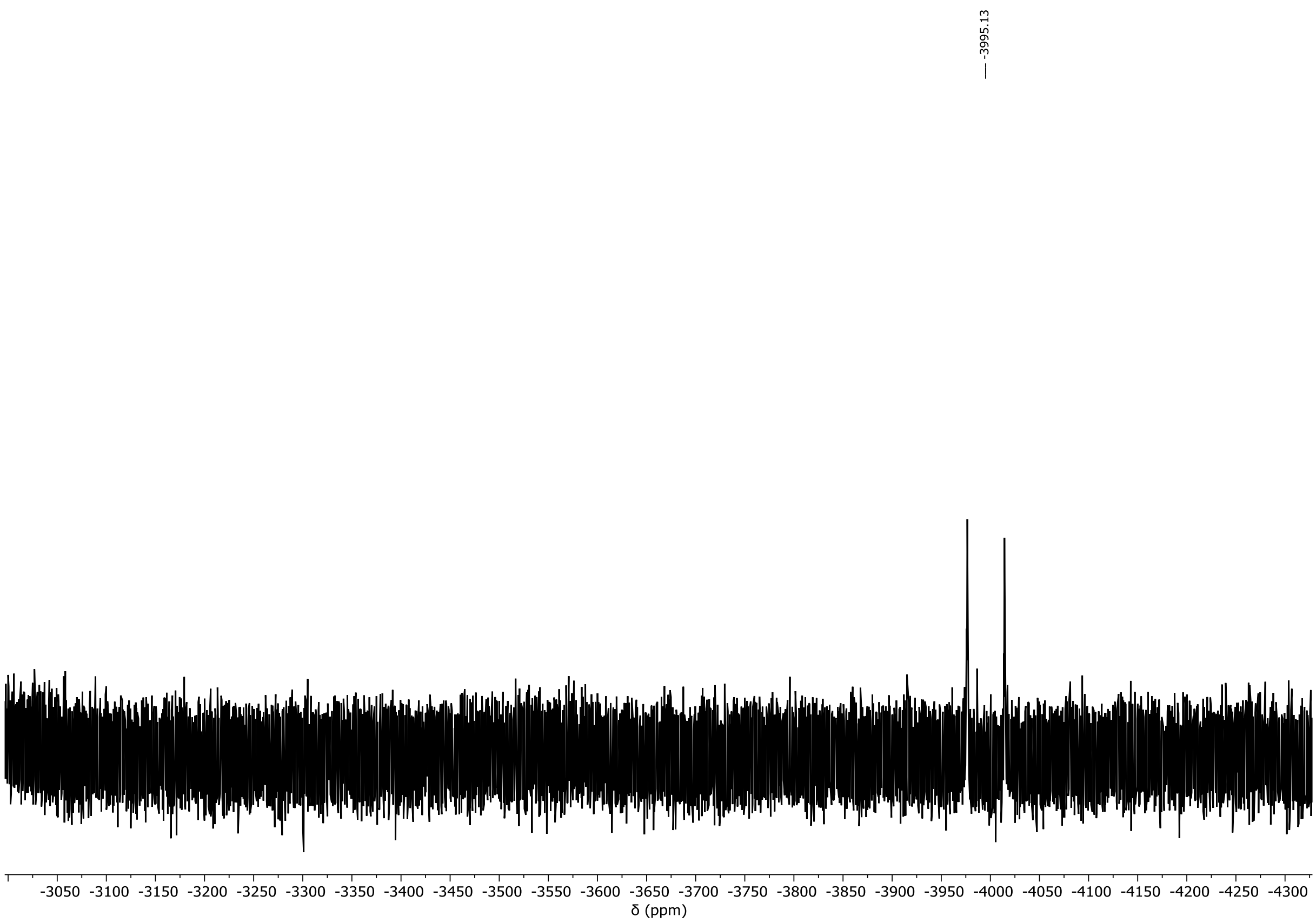

Figure S-116. ^{195}Pt-NMR spectrum (107 MHz, DCM-d_2) of **Pt(L$_H$)PPh$_3$**.

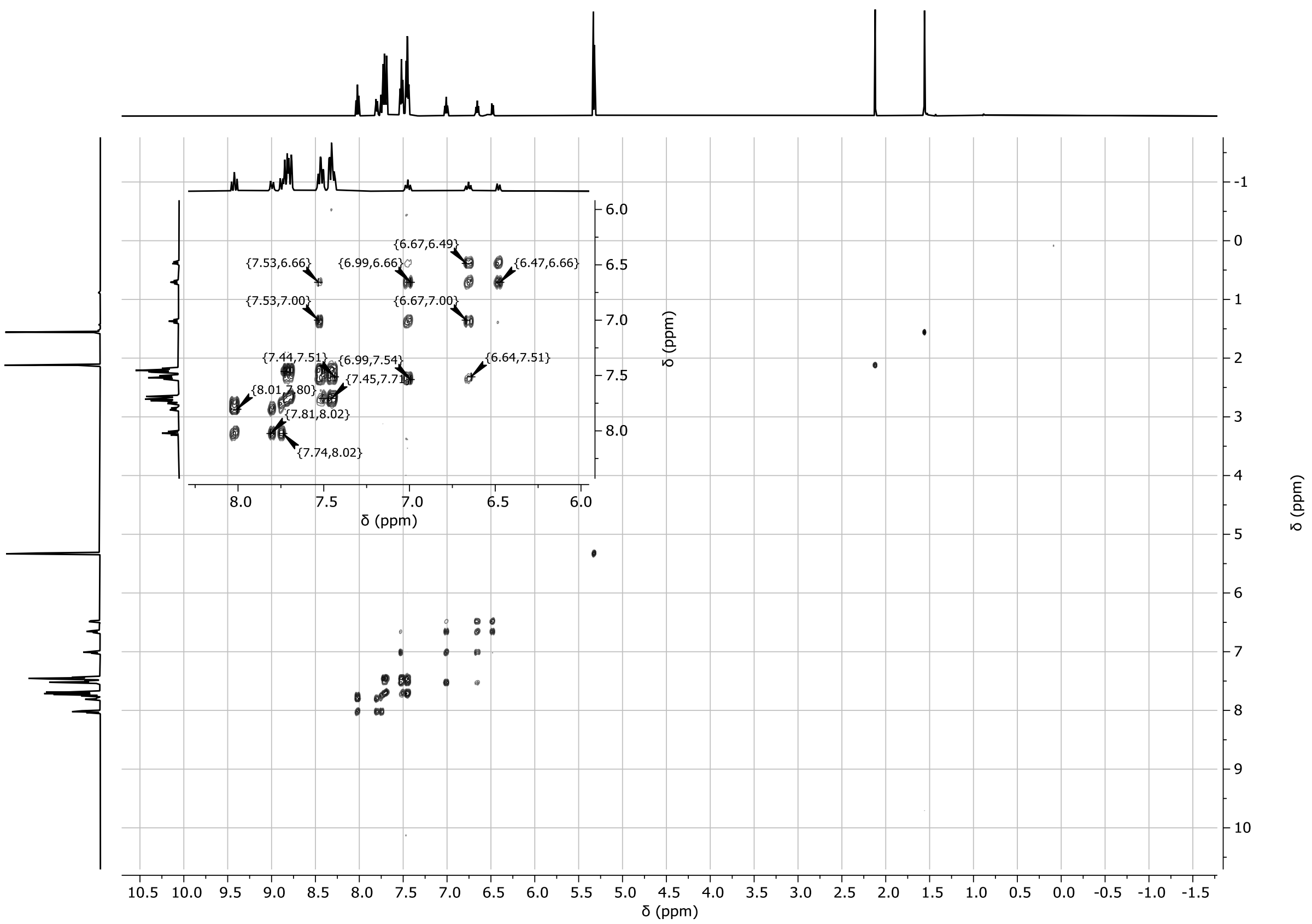

Figure S-117. HH-COSY-NMR spectrum (500 MHz, DCM-d_2) of **Pt(L$_H$)PPh$_3$**.

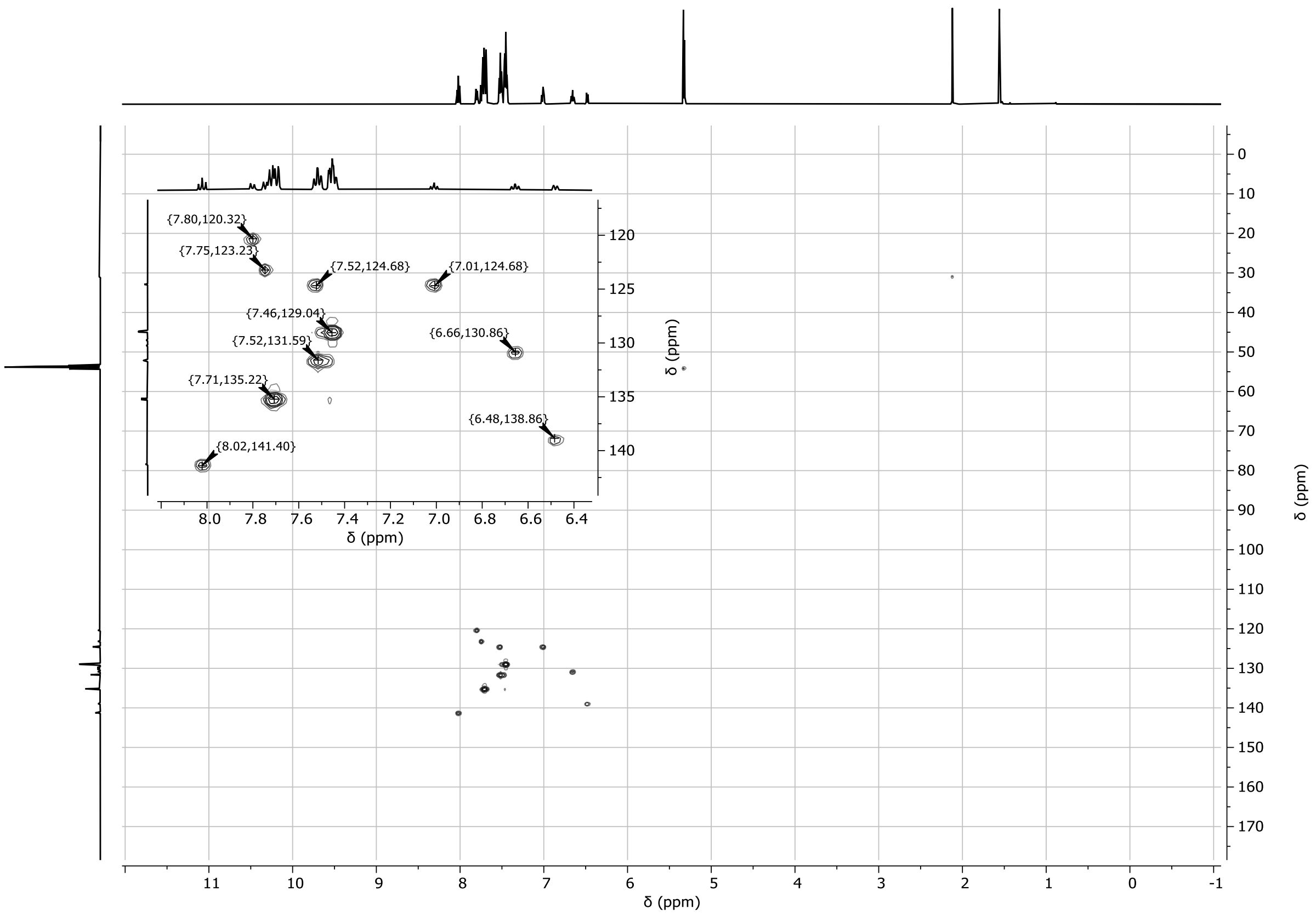

Figure S-118. HC-HSQC-NMR spectrum (126 MHz (^{13}C), 500 MHz (^{1}H), DCM-d_2) of **Pt(L$_H$)PPh$_3$**.

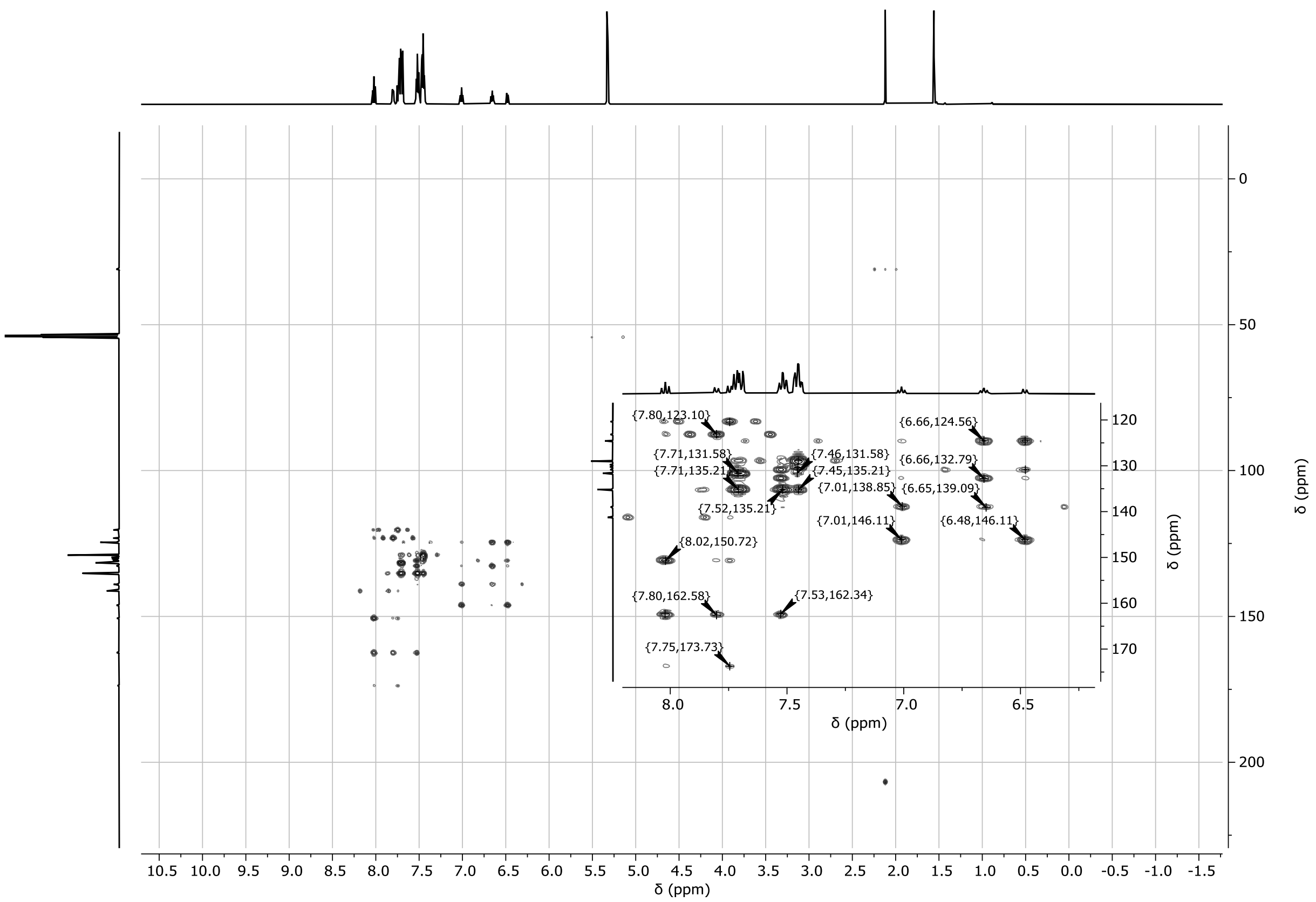

Figure S-119. HC-HMBC-NMR spectrum (126 MHz (^{13}C), 500 MHz (^{1}H), DCM-d_2) of **Pt(L$_H$)PPh$_3$**.

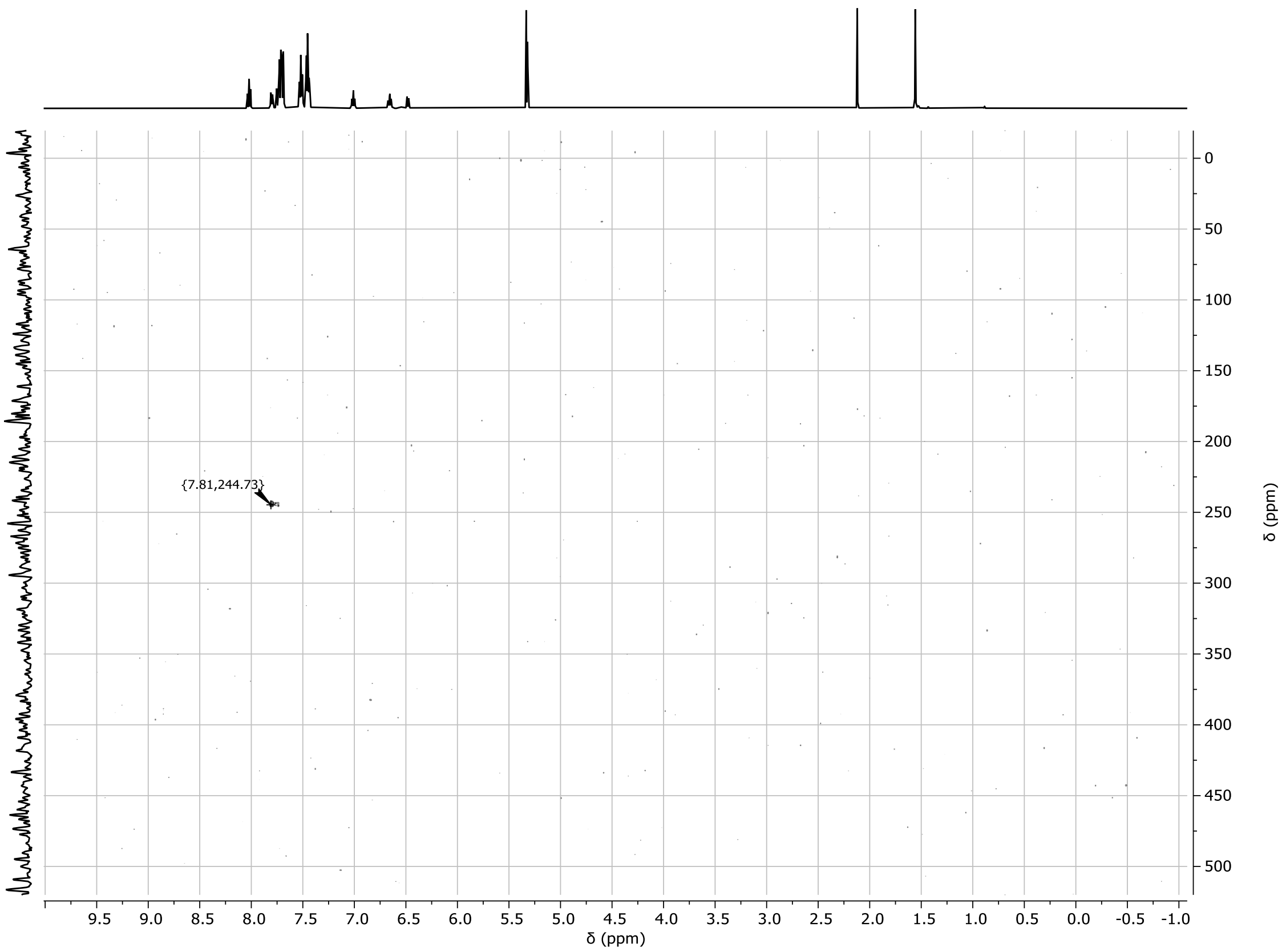

Figure S-120. HN-HMBC-NMR spectrum (51 MHz (^{15}N), 500 MHz (^{1}H), DCM-d_2) of **Pt(L$_H$)PPh$_3$**.

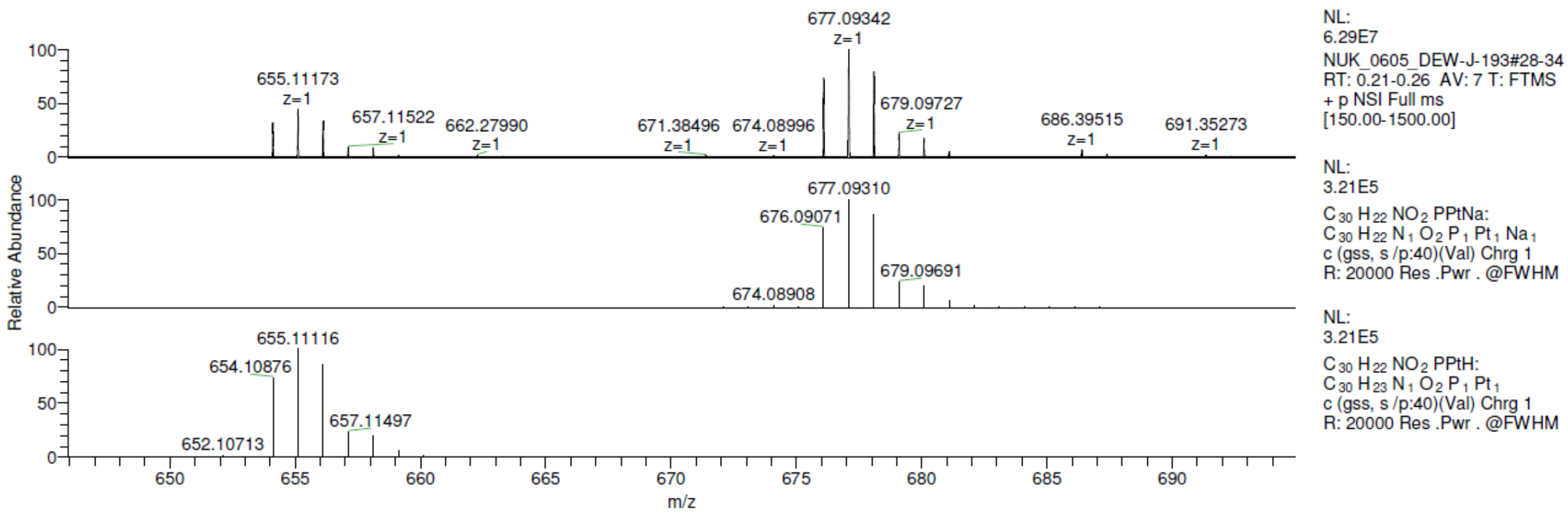

Figure S-121. Mass spectrum of **Pt(L$_H$)PPh$_3$** (MeOH). Additional simulation of the [**Pt(L$_H$)PPh$_3$**+H]$^+$ and [**Pt(L$_H$)PPh$_3$**+Na]$^+$ adduct.

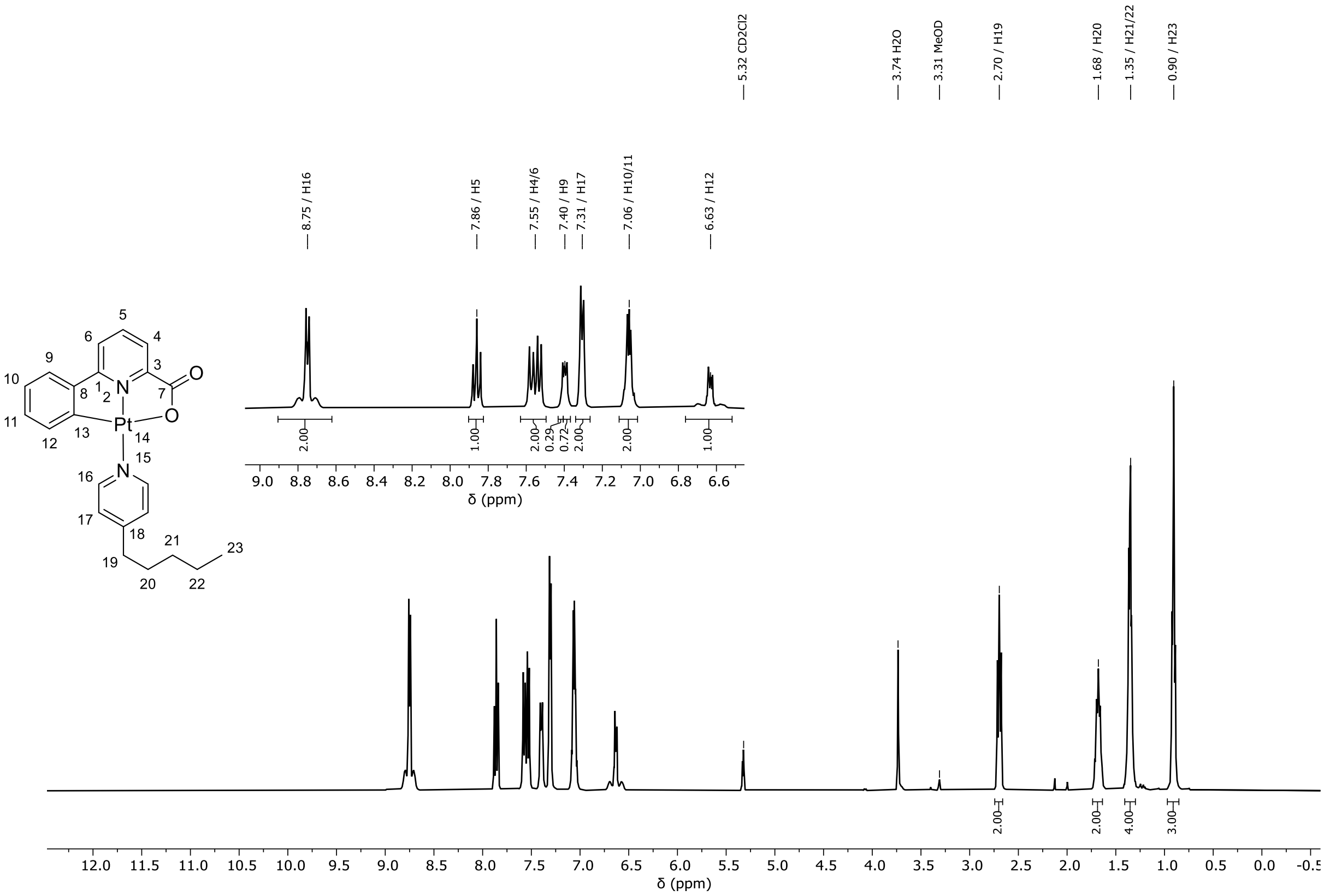

Figure S-122. ^{1}H-NMR spectrum (500 MHz, DCM-d_2) of **Pt(L$_H$)AmPy**.

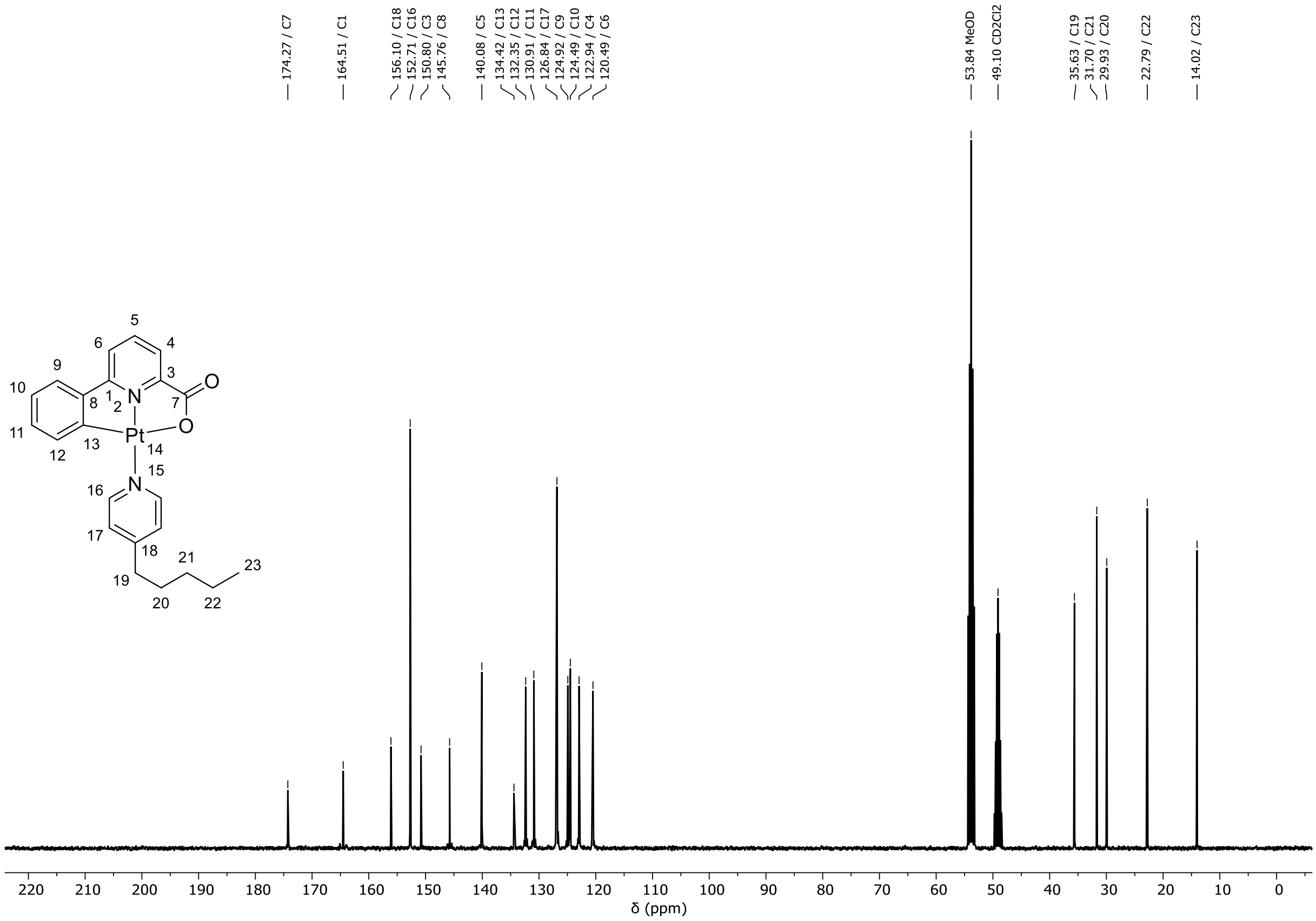

Figure S-123. $^{13}C\{^{1}H\}$-NMR spectrum (126 MHz, DCM-d_2) of **Pt(L$_H$)AmPy**.

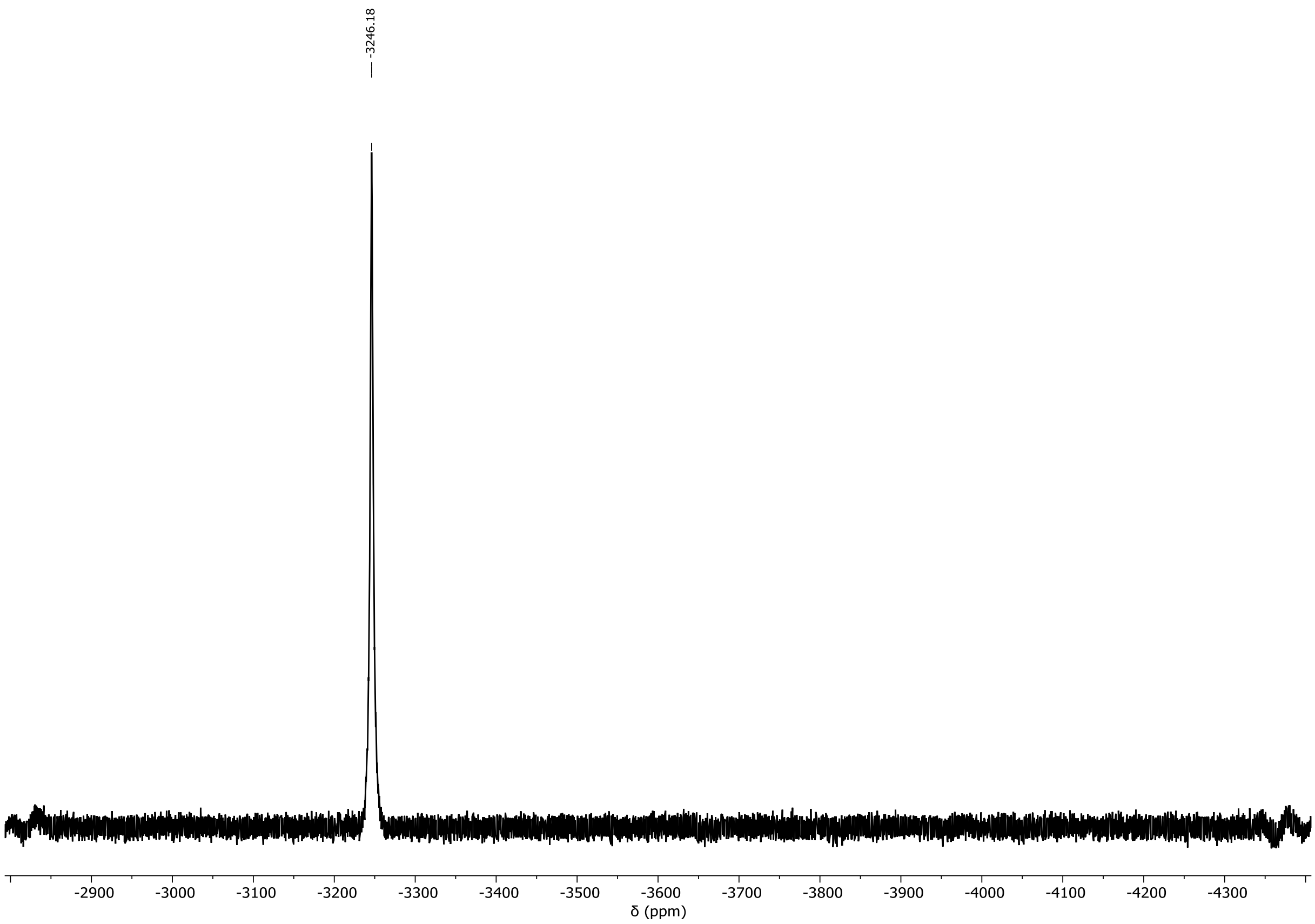

Figure S-124. ^{195}Pt-NMR spectrum (107 MHz, DCM-d_2) of **Pt(L$_H$)AmPy**.

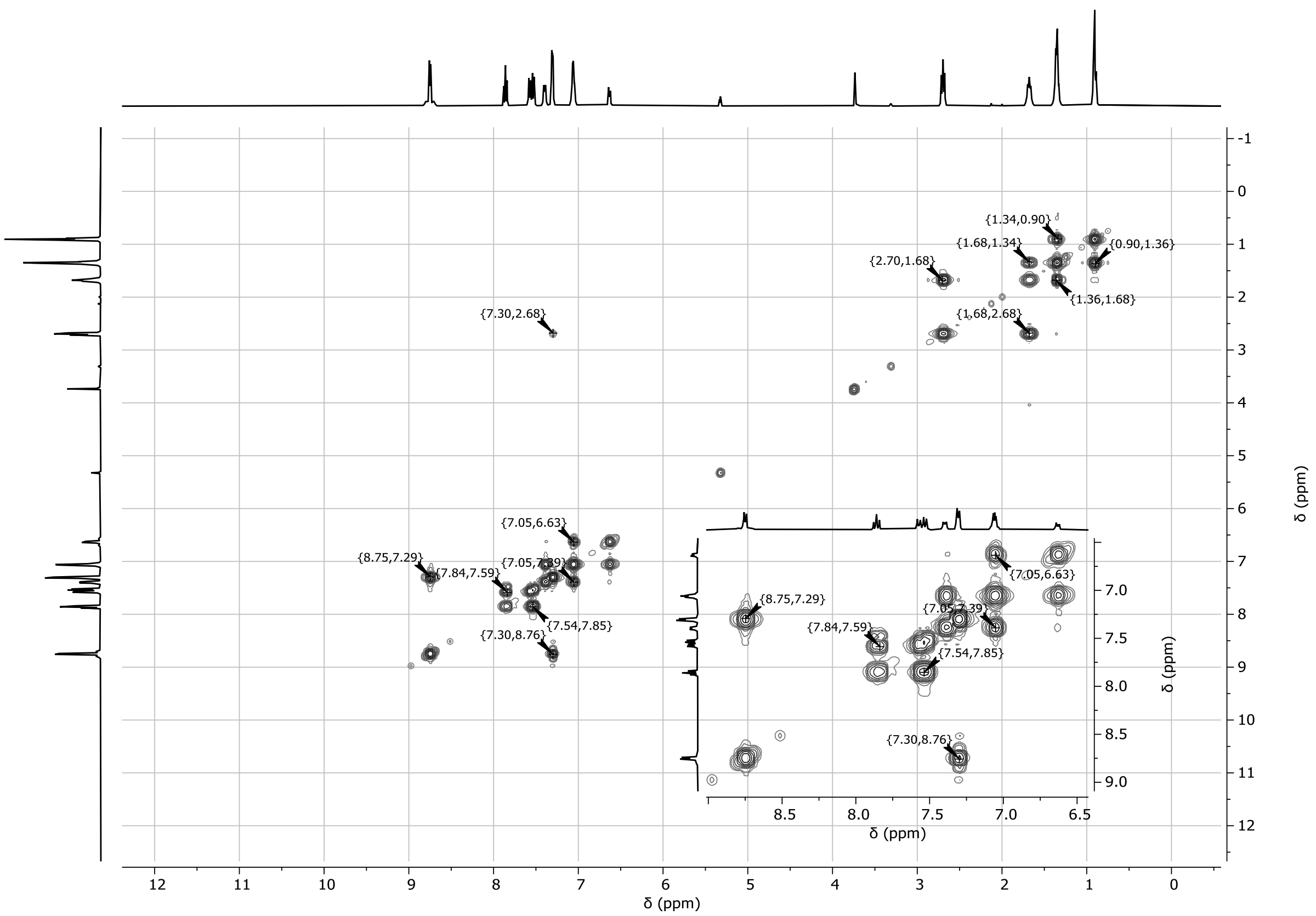

Figure S-125. HH-COSY-NMR spectrum (500 MHz, DCM-d_2) of **Pt(L$_H$)AmPy**.

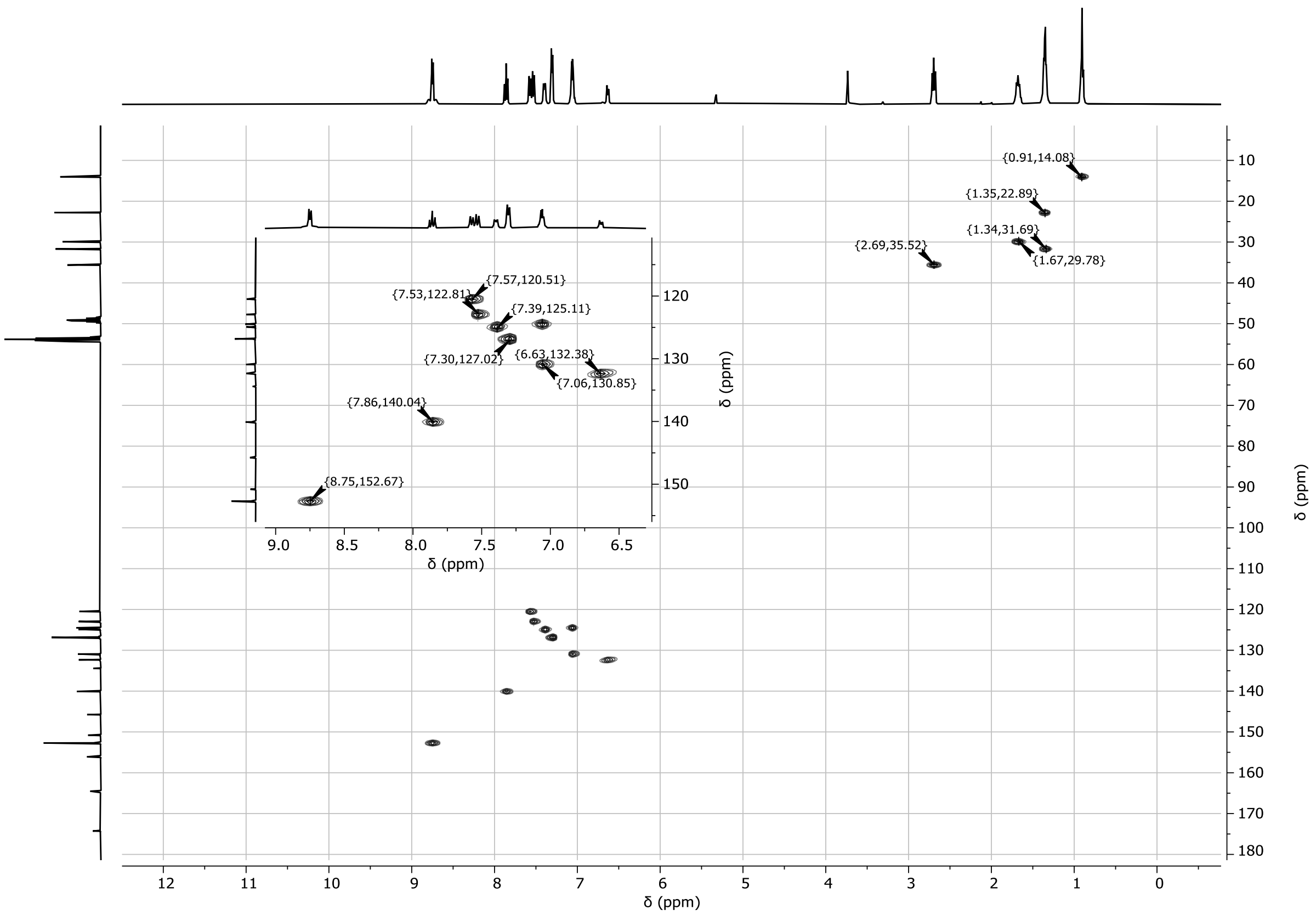

Figure S-126. HC-HSQC-NMR spectrum (126 MHz (^{13}C), 500 MHz (^{1}H), DCM-d_2) of **Pt(L$_H$)AmPy**.

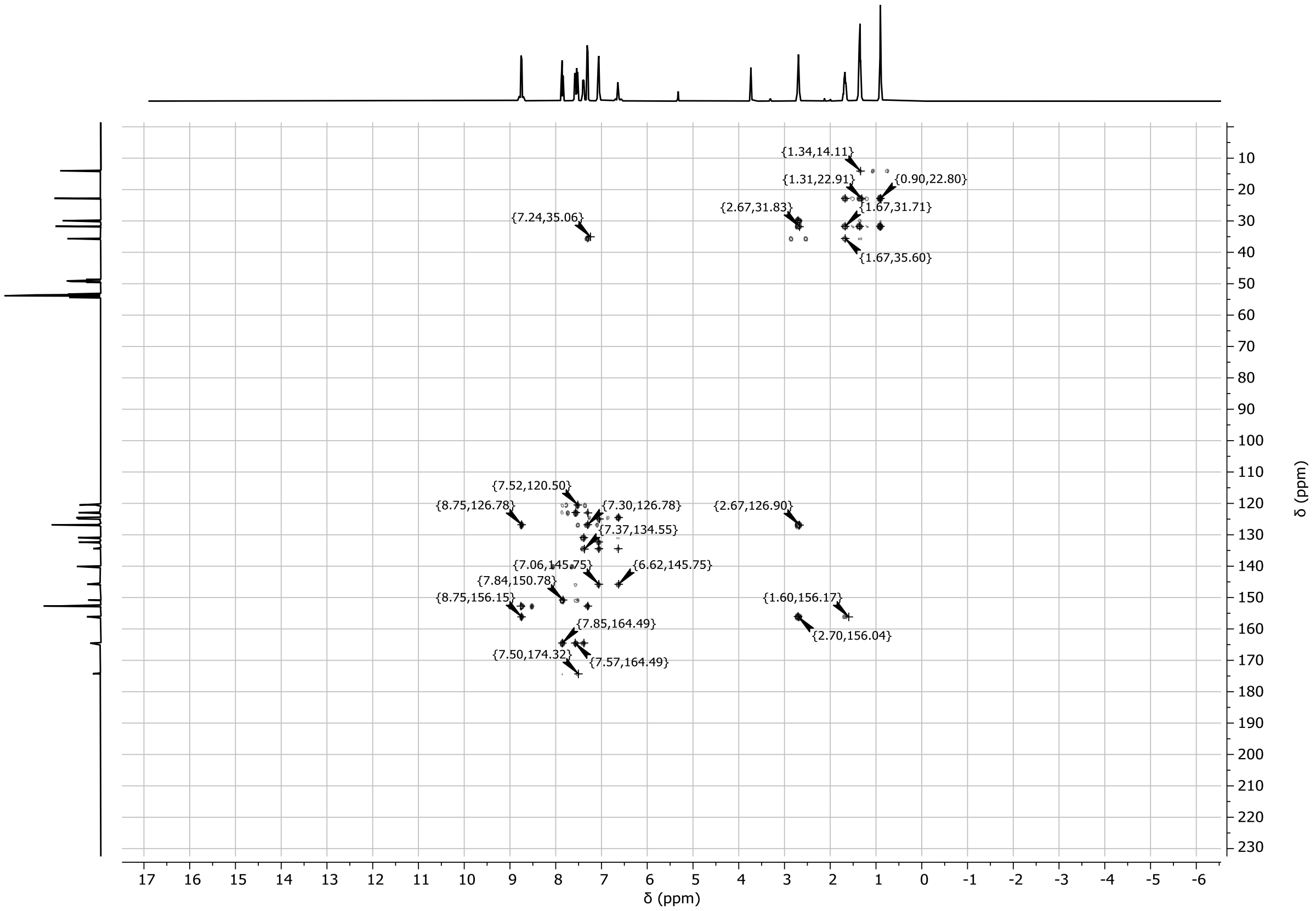

Figure S-127. HC-HMBC-NMR spectrum (126 MHz (^{13}C), 500 MHz (^{1}H), DCM-d_2) of **Pt(L$_H$)AmPy**.

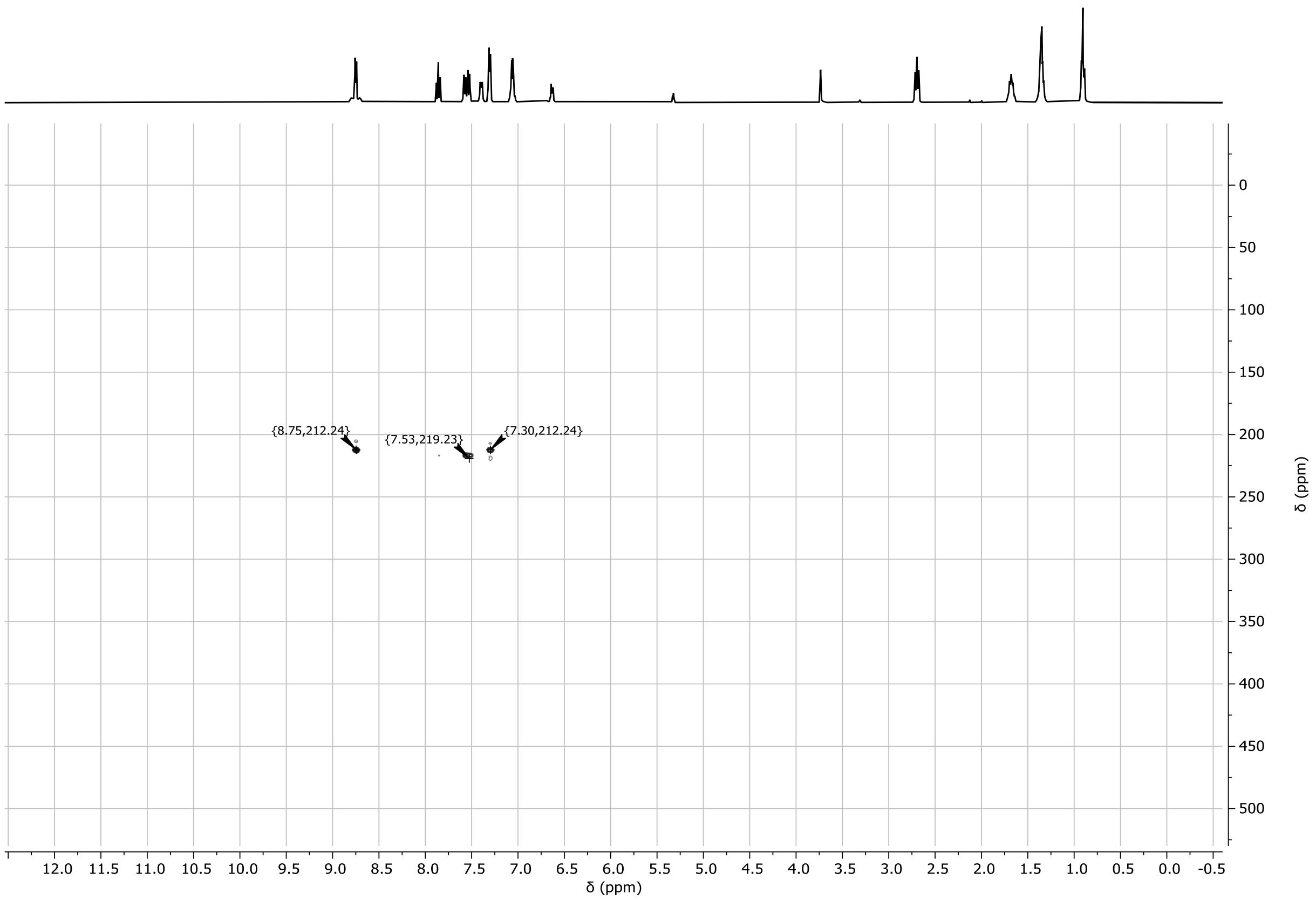

Figure S-128. HN-HMBC-NMR spectrum (51 MHz (^{15}N), 500 MHz (^{1}H), DCM-d_2) of **Pt(L$_H$)AmPy**.

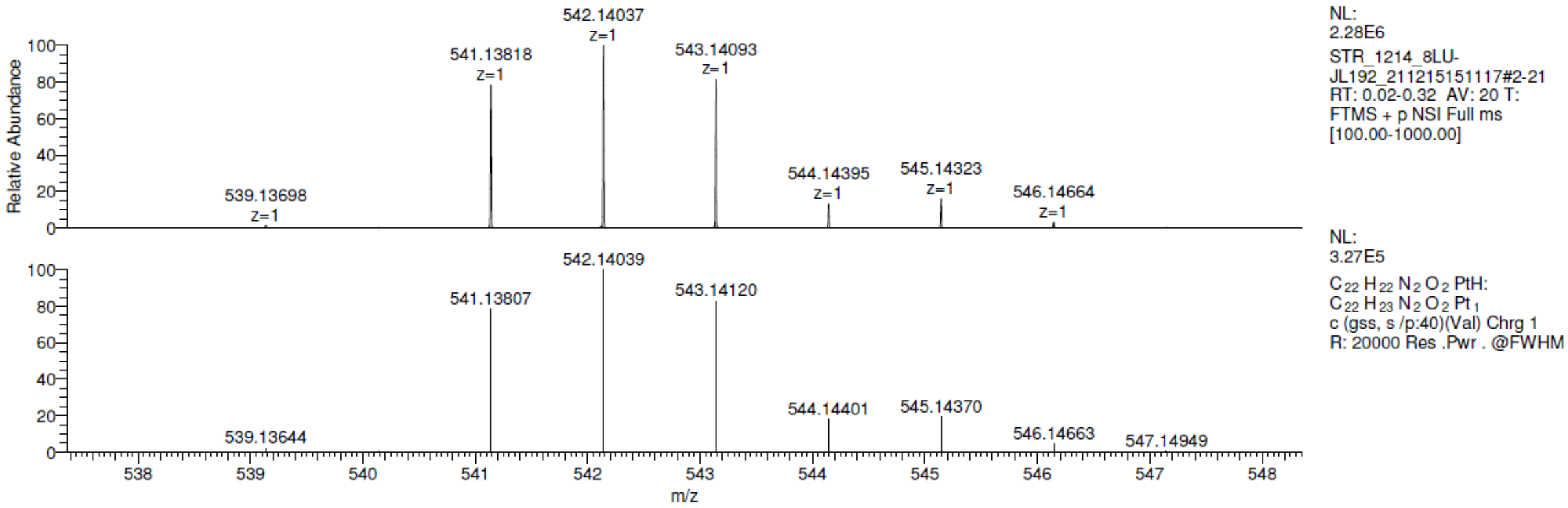

Figure S-129. Mass spectrum of **Pt(LH)AmPy** (MeOH). Additional simulation of the [**Pt(LH)AmPy**+H]+ adduct.

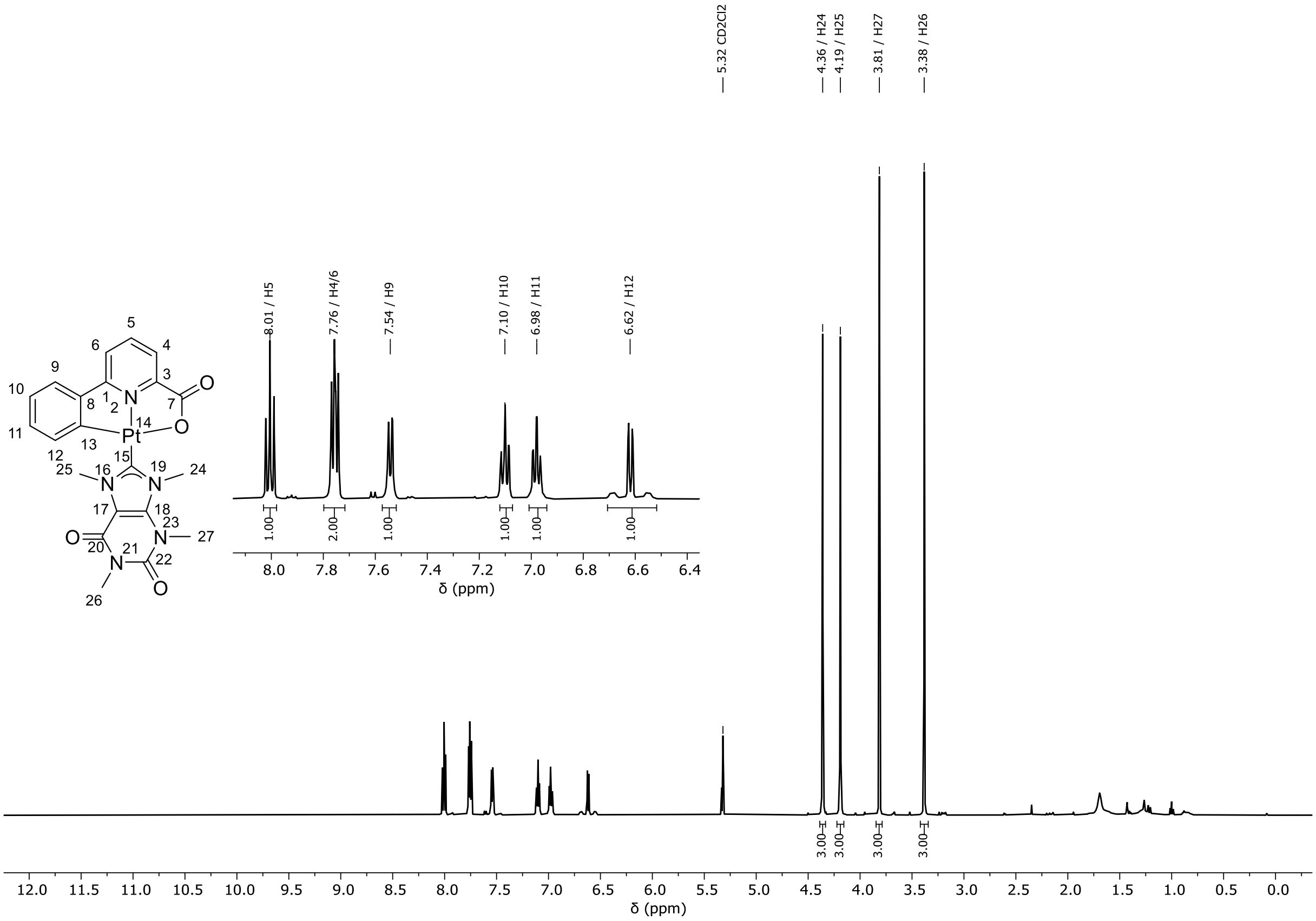

Figure S-130. ^{195}Pt-NMR spectrum (107 MHz, DCM-d_2) of **Pt(LH)NHC**.

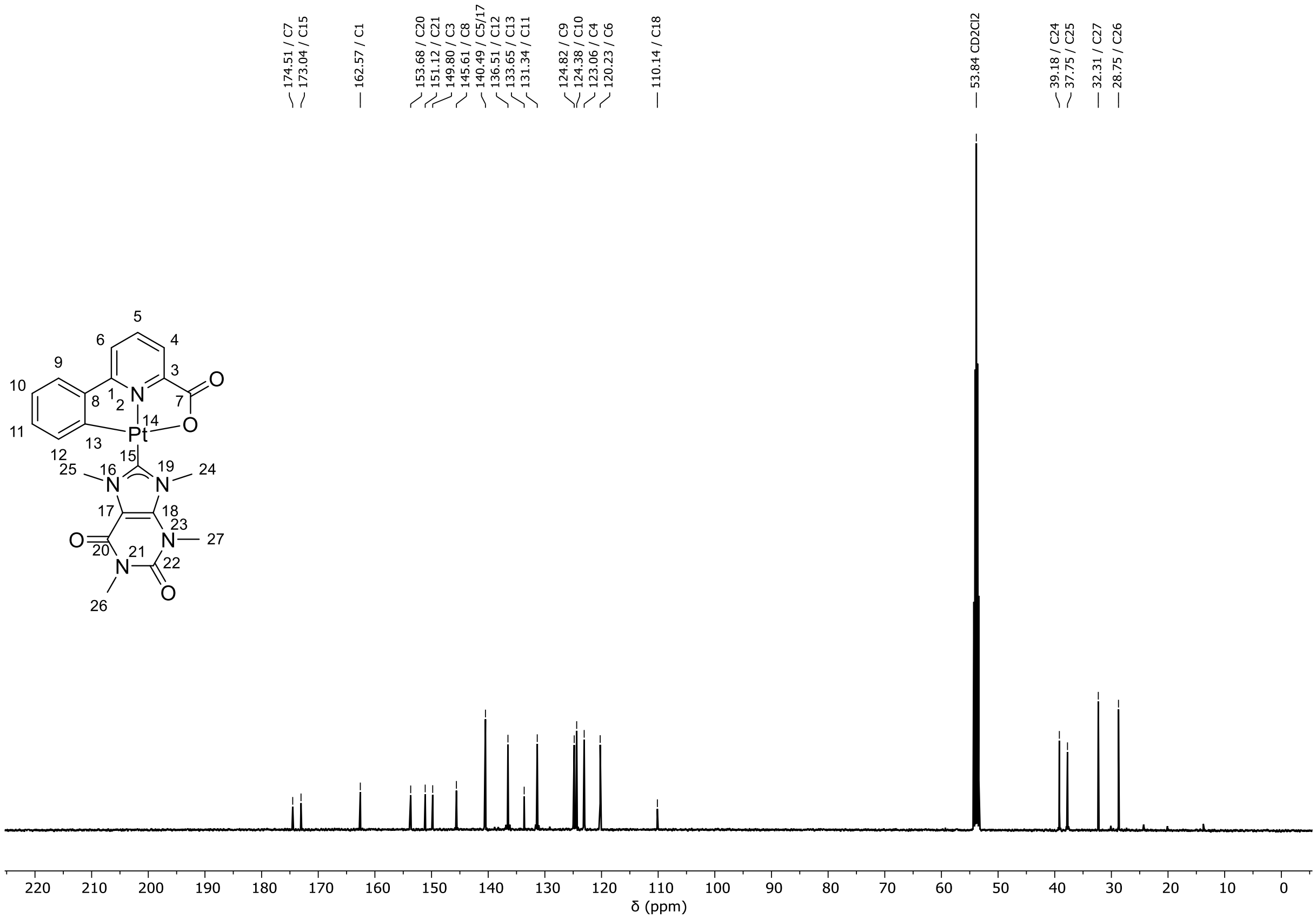

Figure S-131. $^{13}C\{^{1}H\}$-NMR spectrum (126 MHz, DCM-d_2) of **Pt(L$_H$)NHC**.

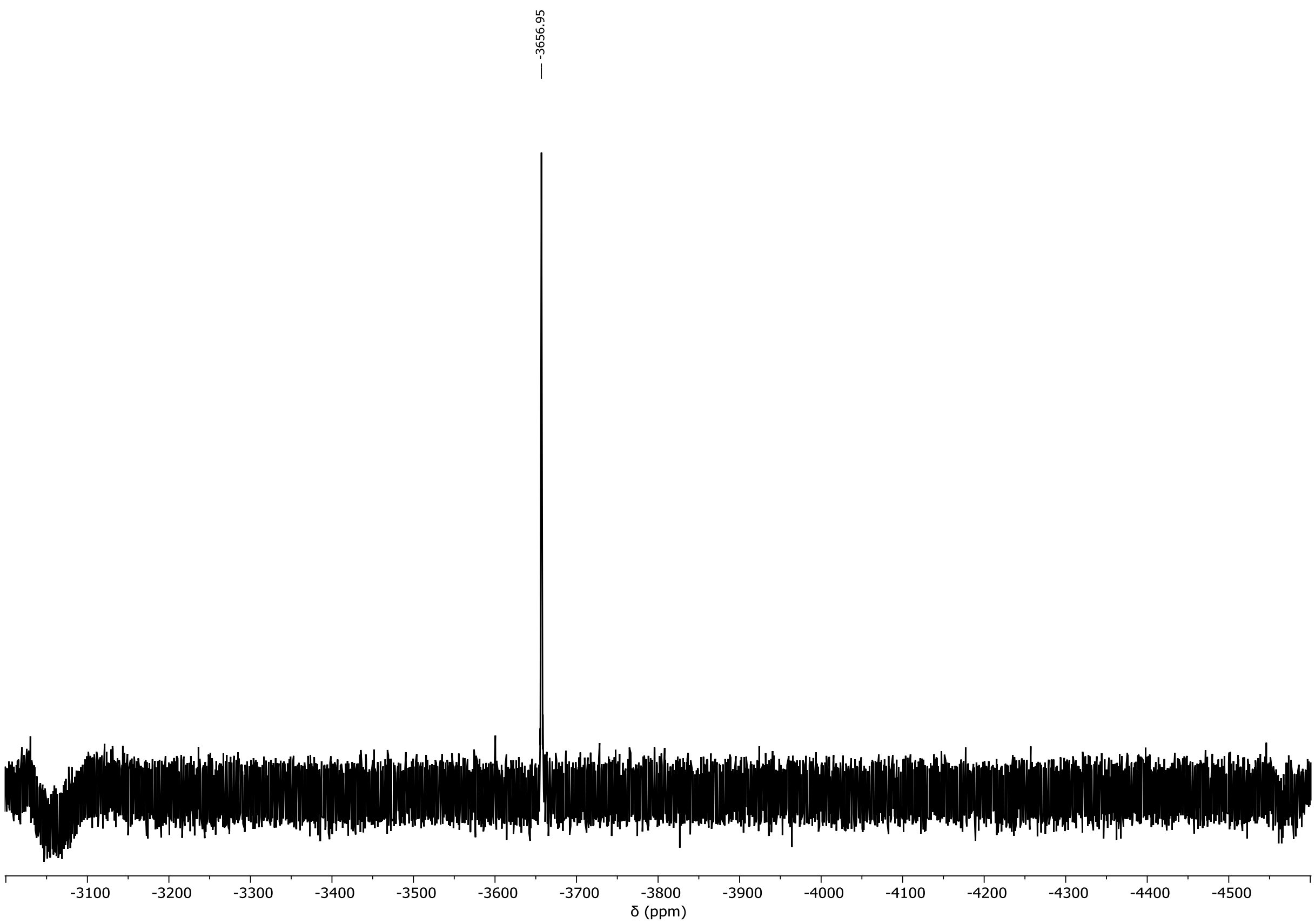

Figure S-132. ^{195}Pt-NMR spectrum (107 MHz, DCM-d_2) of **Pt(L$_H$)NHC**.

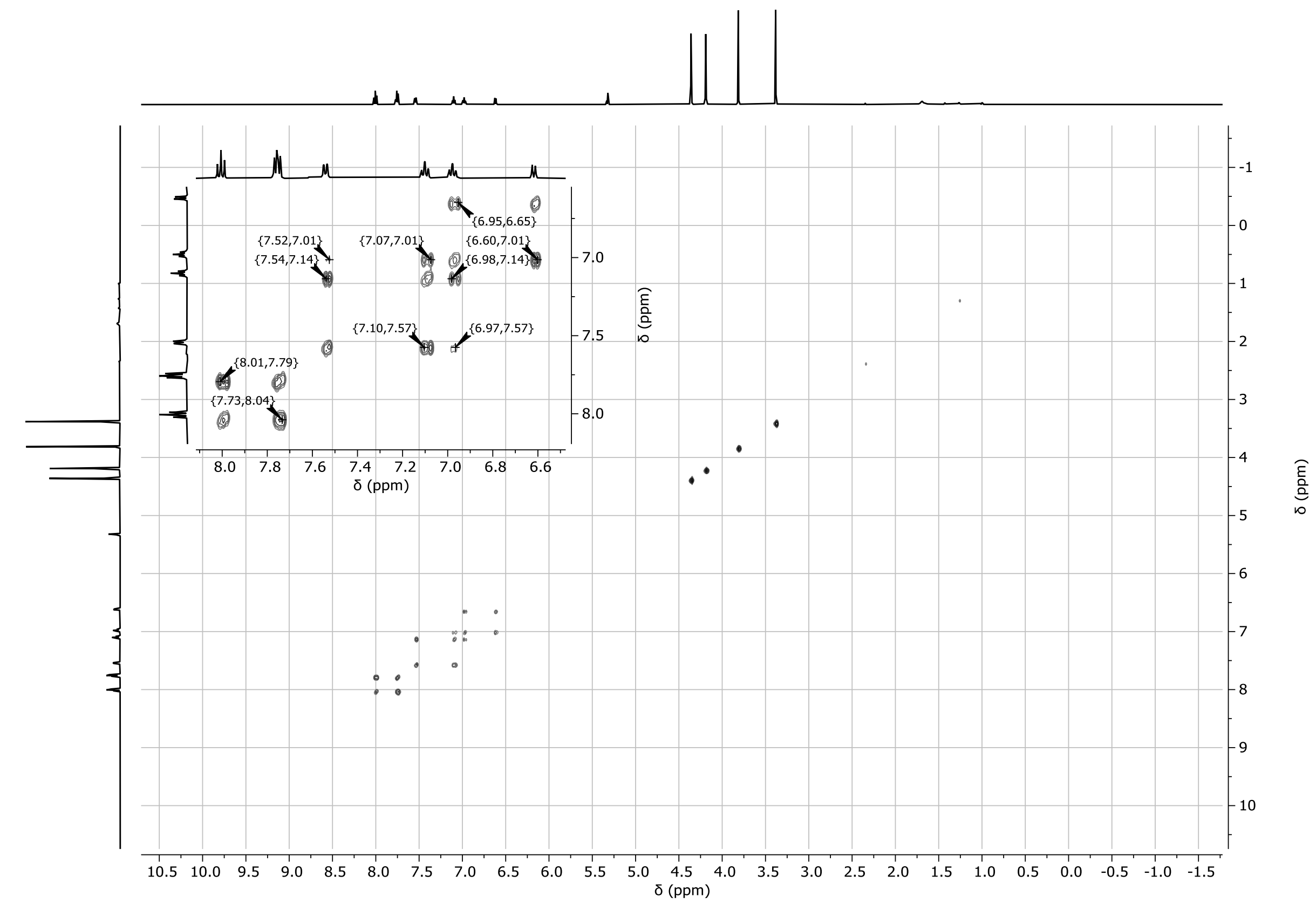

Figure S-133. HH-COSY-NMR spectrum (500 MHz, DCM-d_2) of **Pt(L$_H$)NHC**.

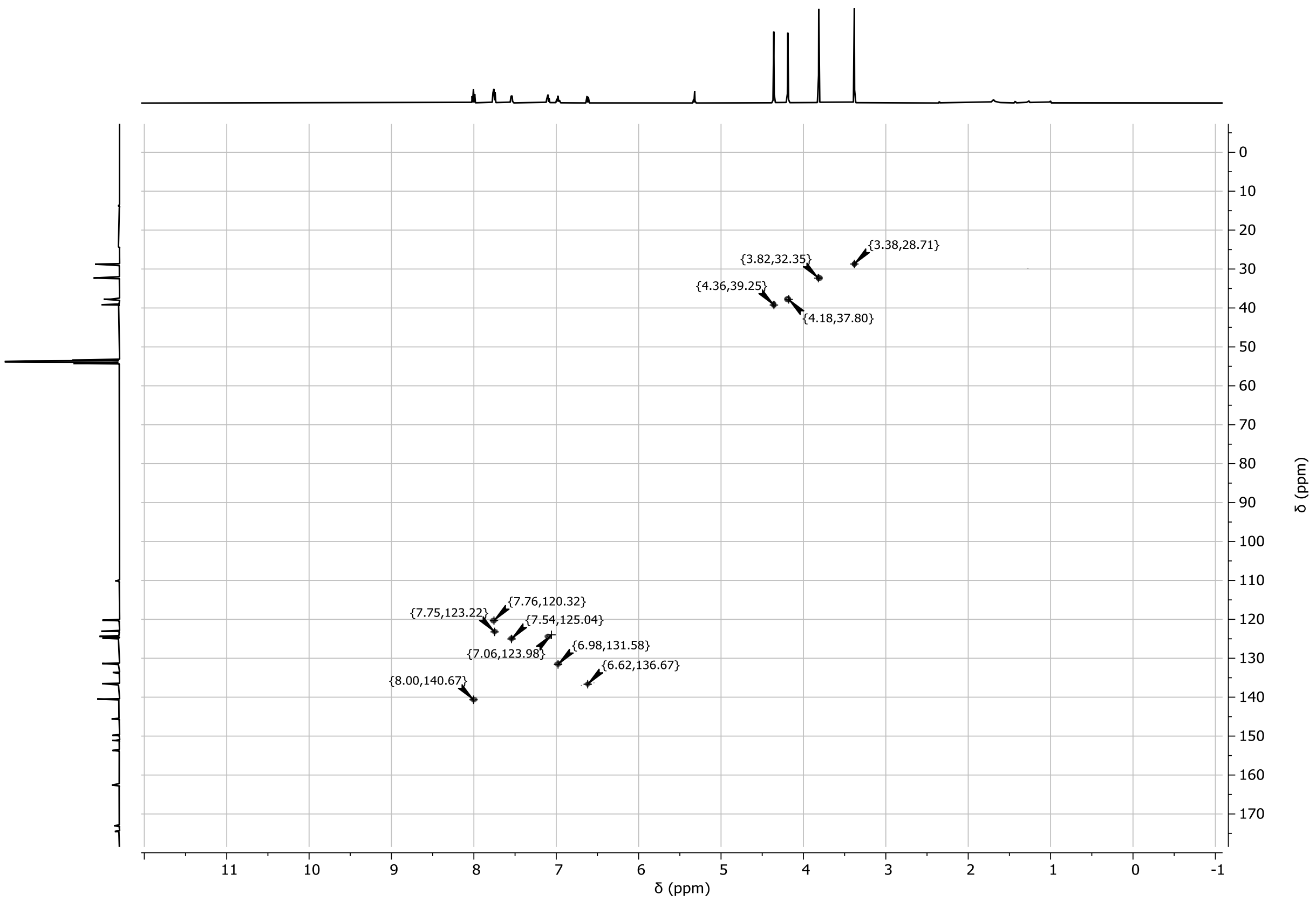

Figure S-134. HC-HSQC-NMR spectrum (126 MHz (^{13}C), 500 MHz (^{1}H), DCM-d_2) of **Pt(L$_H$)NHC**.

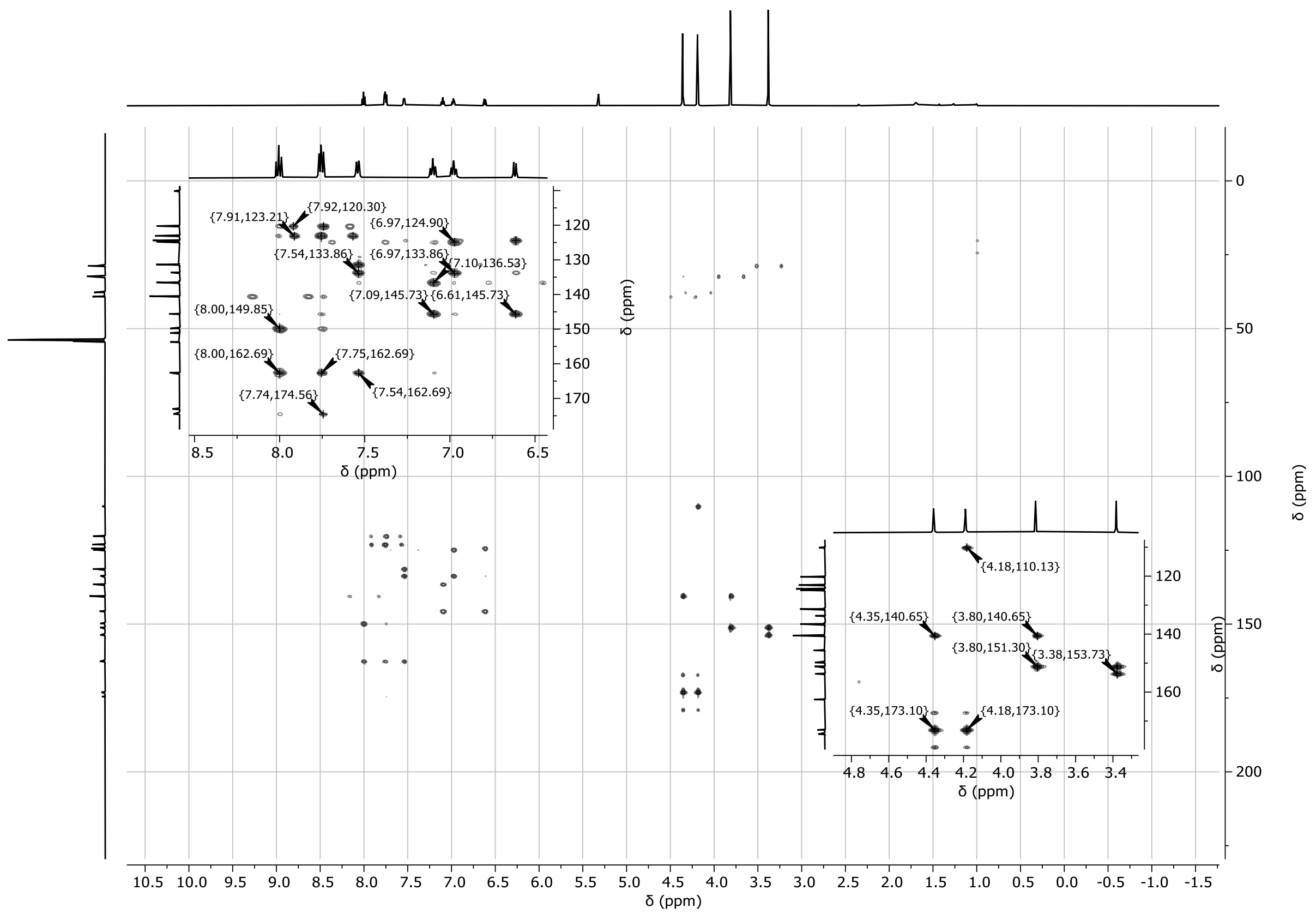

Figure S-135. HC-HMBC-NMR spectrum (126 MHz (^{13}C), 500 MHz (^{1}H), DCM-d_2) of **Pt(L$_H$)NHC**.

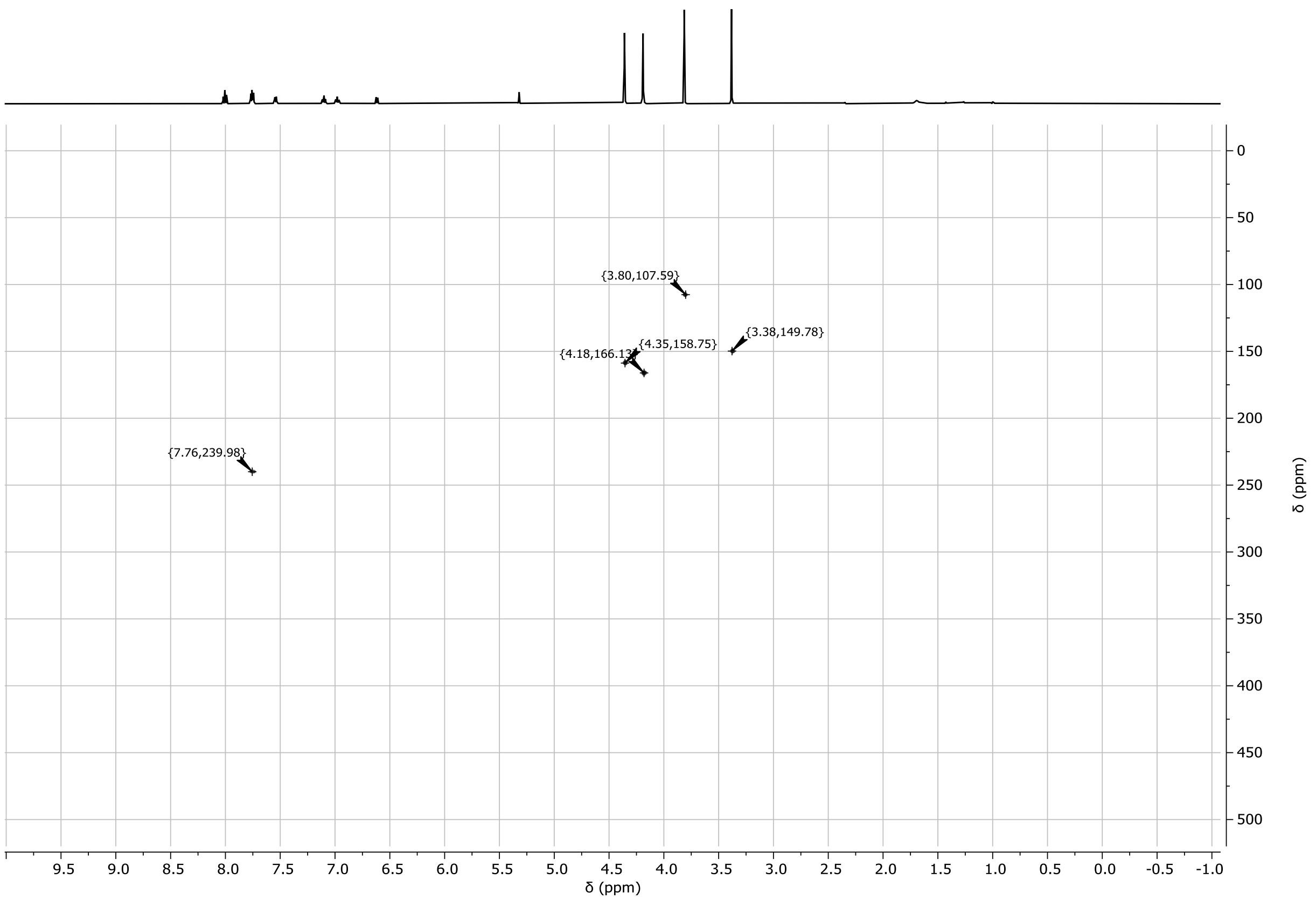

Figure S-136. HN-HMBC-NMR spectrum (51 MHz (^{15}N), 500 MHz (^{1}H), DCM-d_2) of **Pt(L$_H$)AmPy**.

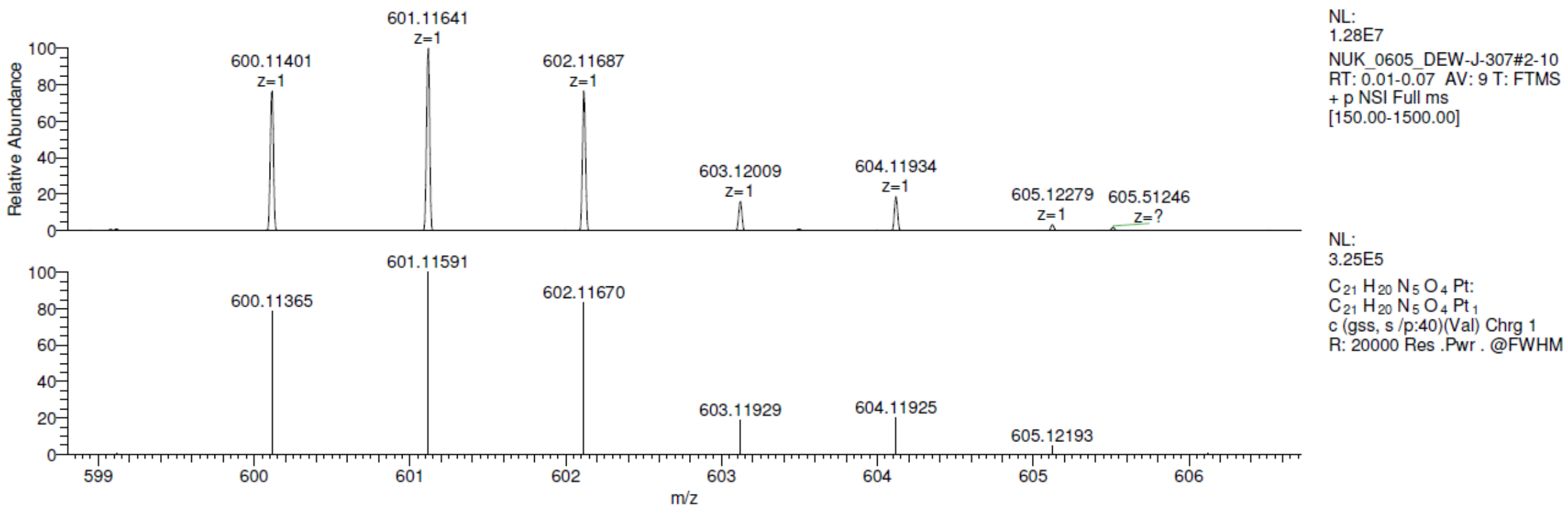

Figure S-137. Mass spectrum of **Pt(L$_H$)NHC** (MeOH). Additional simulation of the [**Pt(L$_H$)NHC**+H]$^+$ adduct.

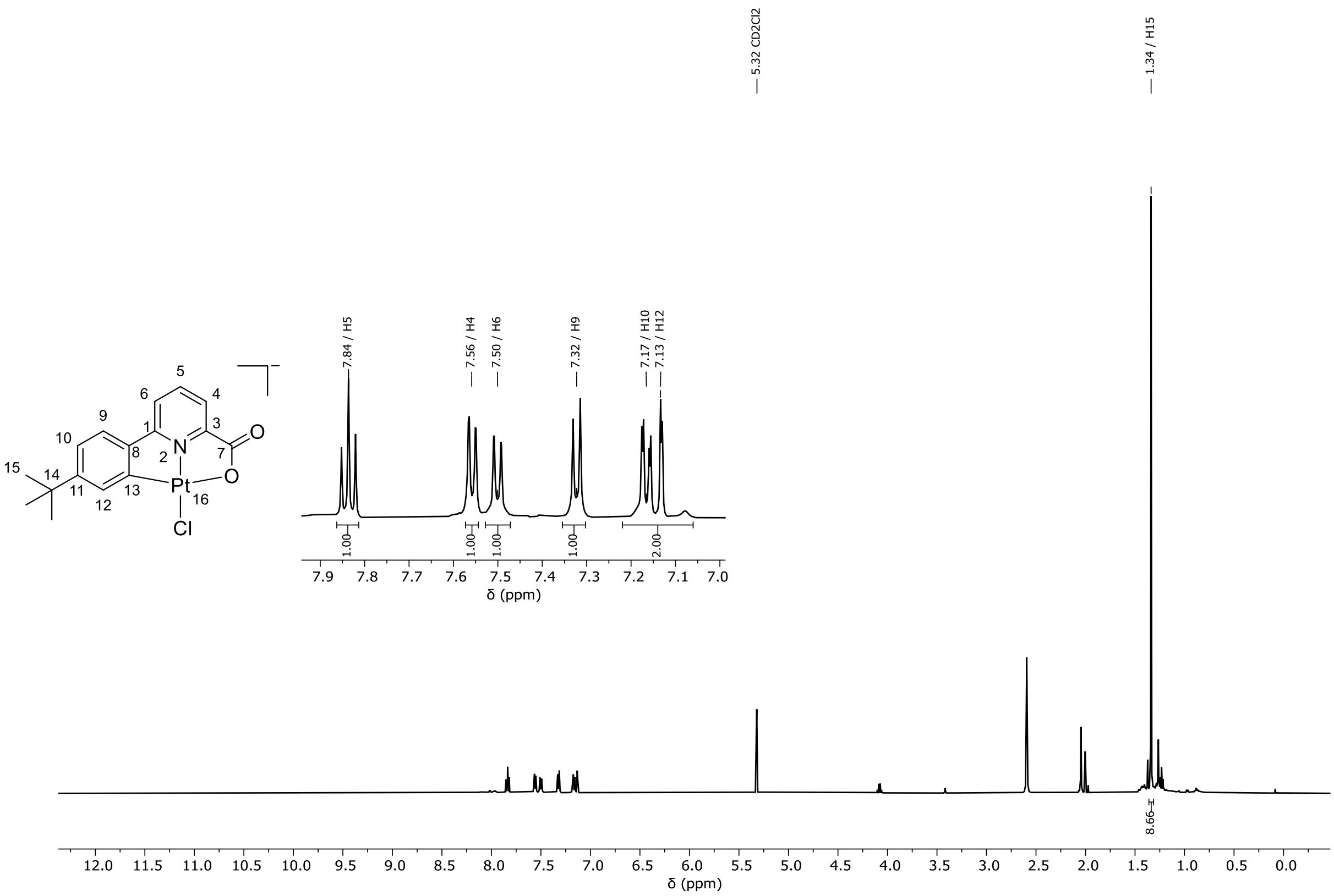

Figure S-138. ^{1}H-NMR spectrum (400 MHz, DCM-d_2) of **Pt(L$_{tBu}$)Cl**.

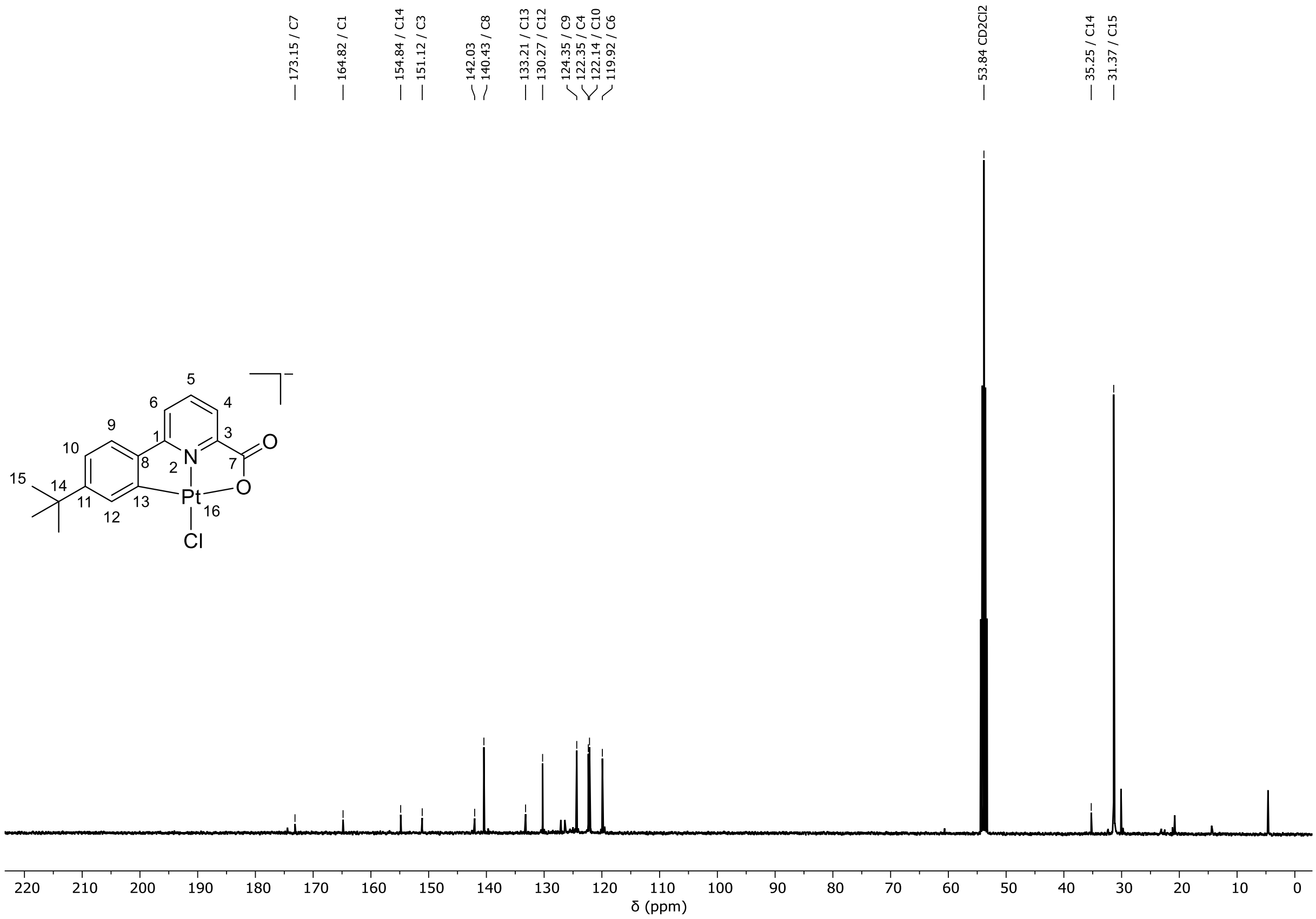

Figure S-139. $^{13}C\{^{1}H\}$-NMR spectrum (101 MHz, DCM-d_2) of **Pt(L$_{tBu}$)Cl**.

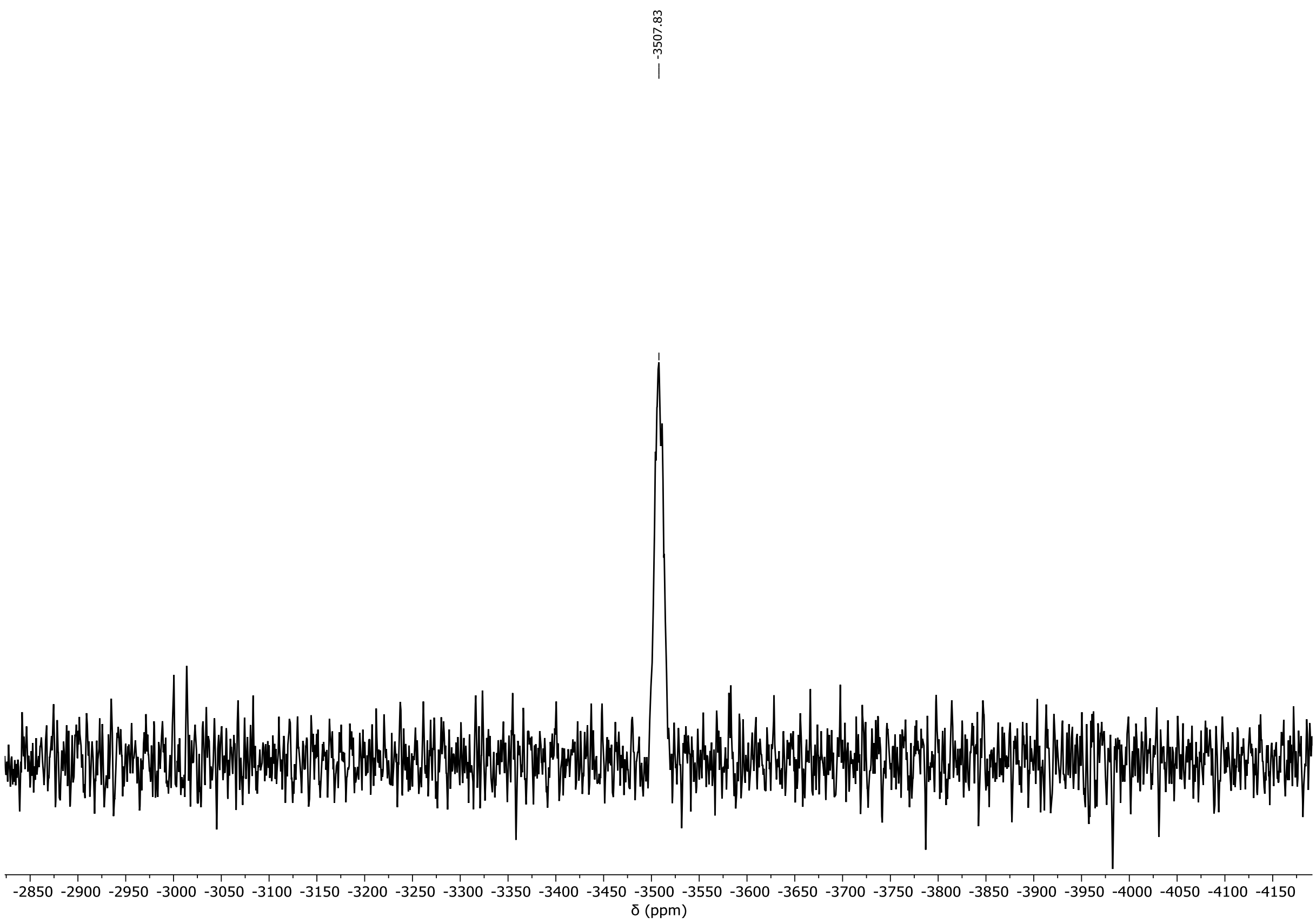

Figure S-140. ^{195}Pt-NMR spectrum (86 MHz, DCM-d_2) of **Pt(L$_{tBu}$)Cl**.

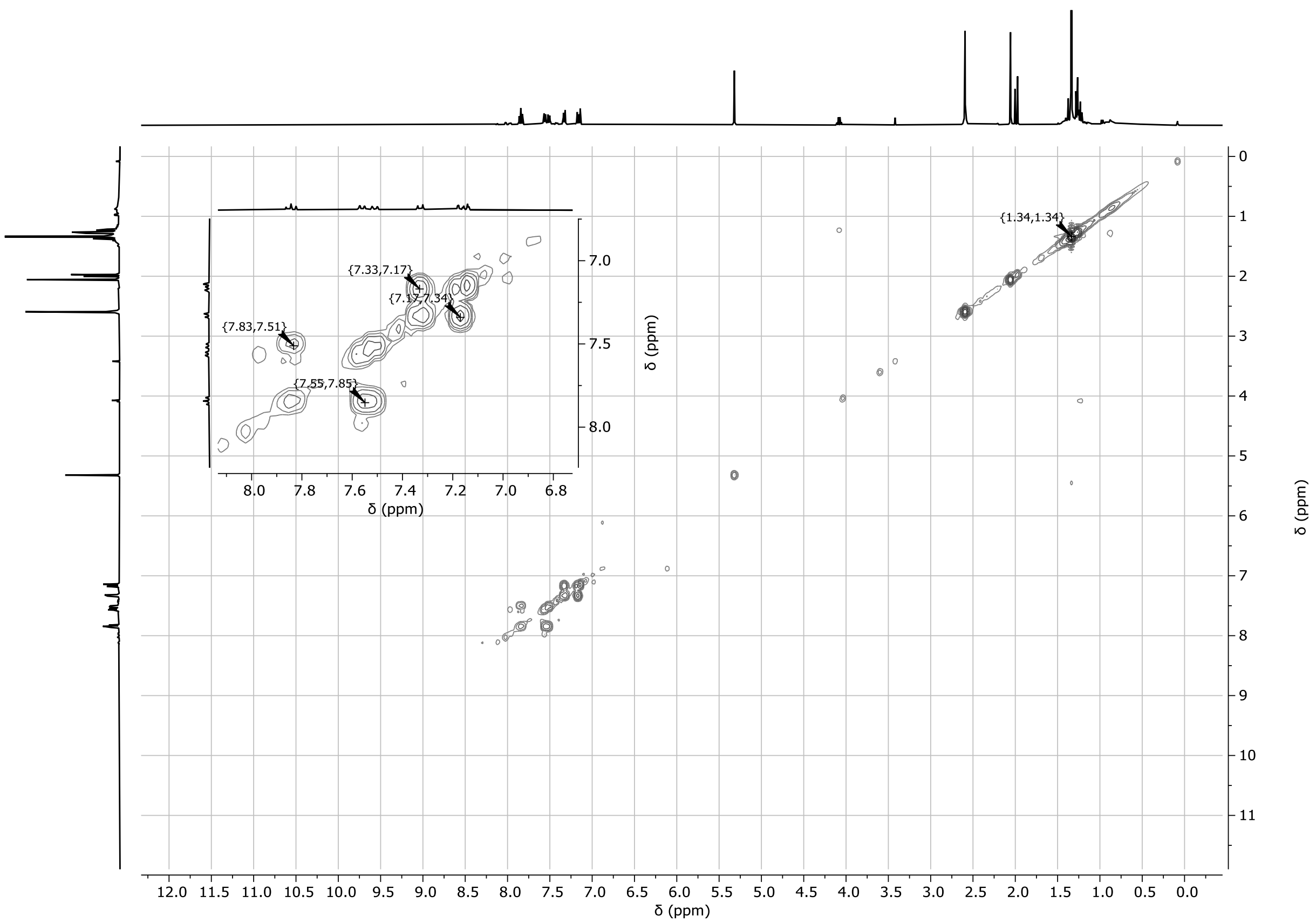

Figure S-141. HH-COSY-NMR spectrum (400 MHz, DCM-d_2) of **Pt(L_{tBu})Cl**.

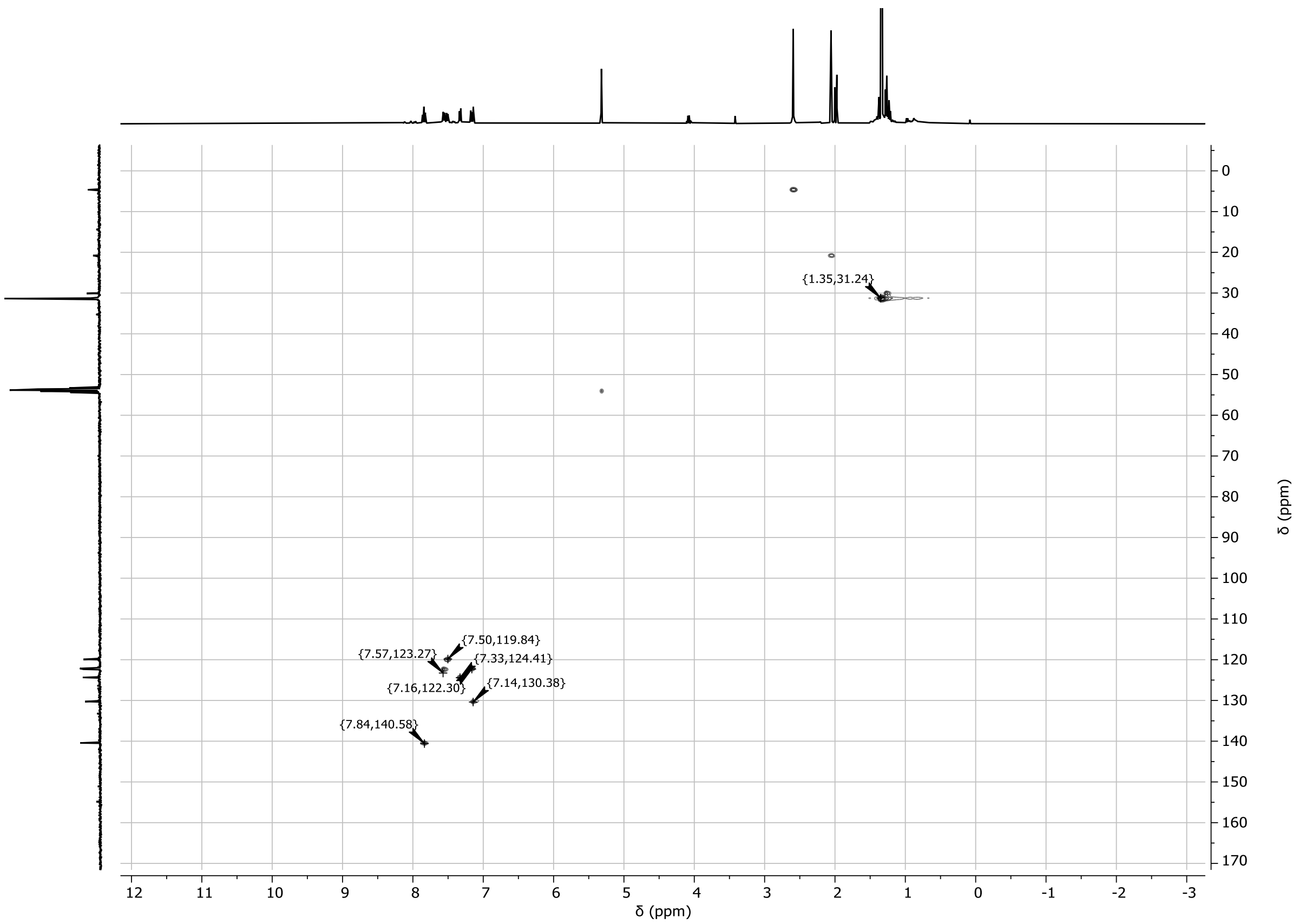

Figure S-142. HC-HSQC-NMR spectrum (101 MHz (^{13}C), 400 MHz (^{1}H), DCM-d_2) of **Pt(L_{tBu})Cl**.

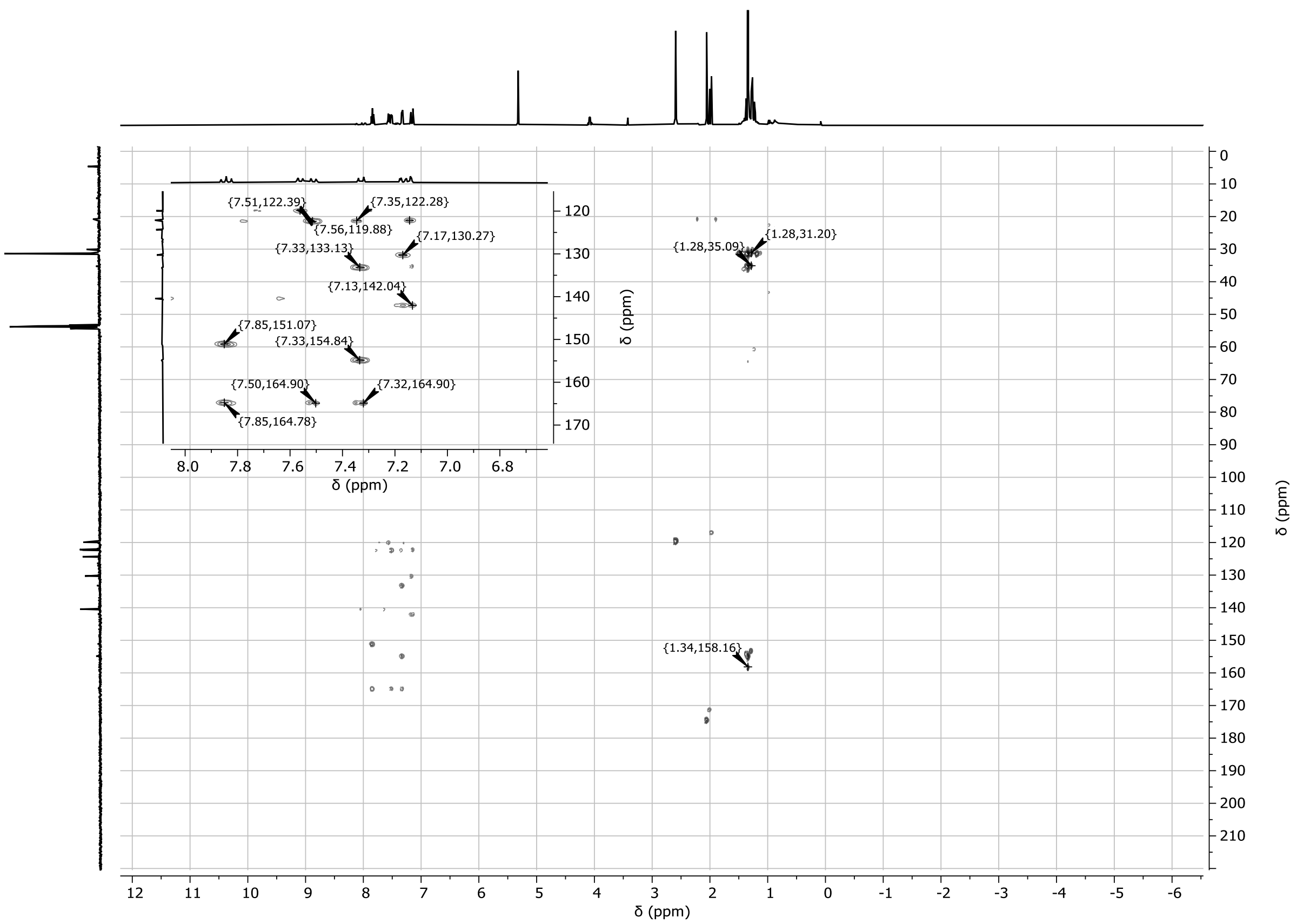

Figure S-143. HC-HMBC-NMR spectrum (101 MHz (^{13}C), 400 MHz (^{1}H), DCM-d_2) of **Pt(L$_{tBu}$)Cl**.

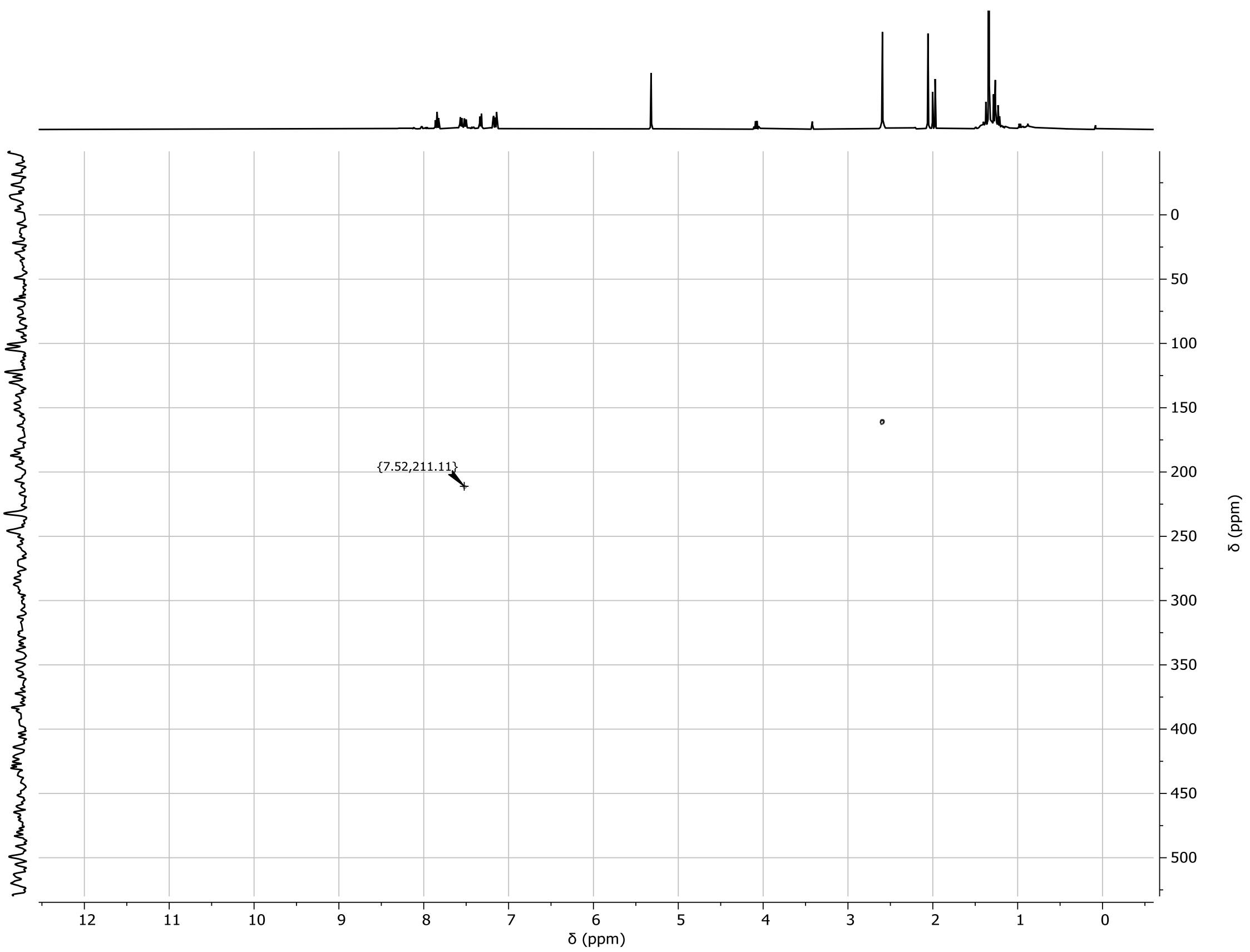

Figure S-144. HN-HMBC-NMR spectrum (101 MHz (^{15}N), 400 MHz (^{1}H), DCM-d_2) of **Pt(L$_{tBu}$)Cl**.

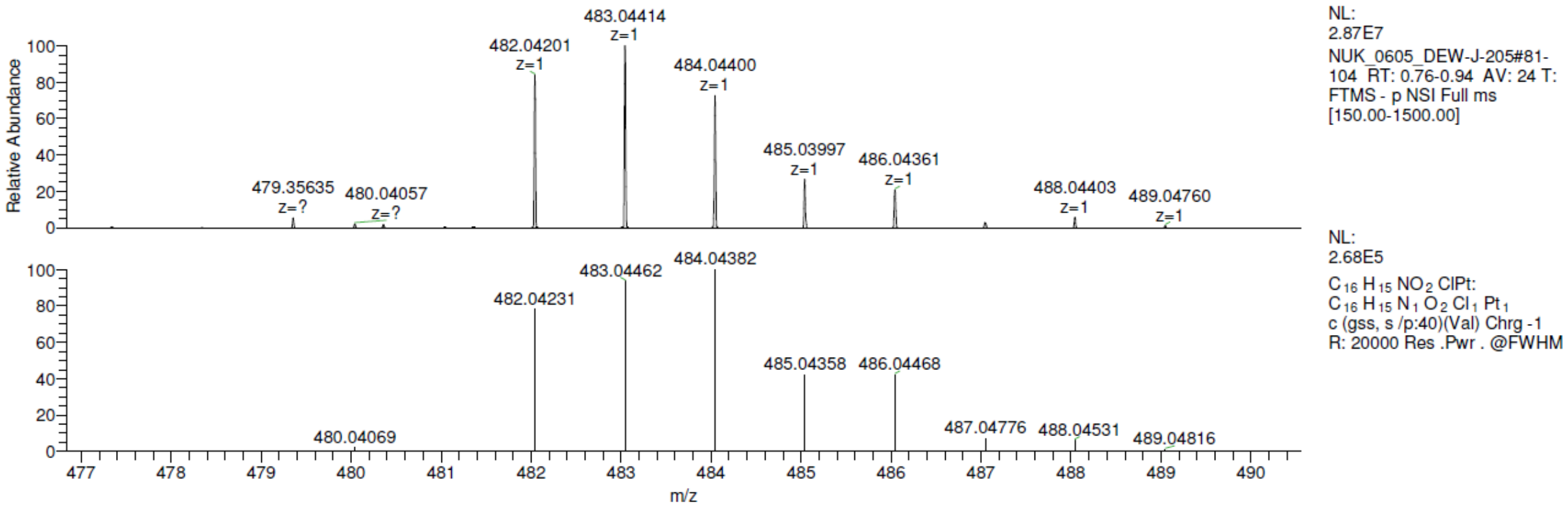

Figure S-145. Mass spectrum of **Pt(L$_{tBu}$)Cl** (MeOH). Additional simulation of the [**Pt(L$_{tBu}$)Cl**]$^-$ adduct.

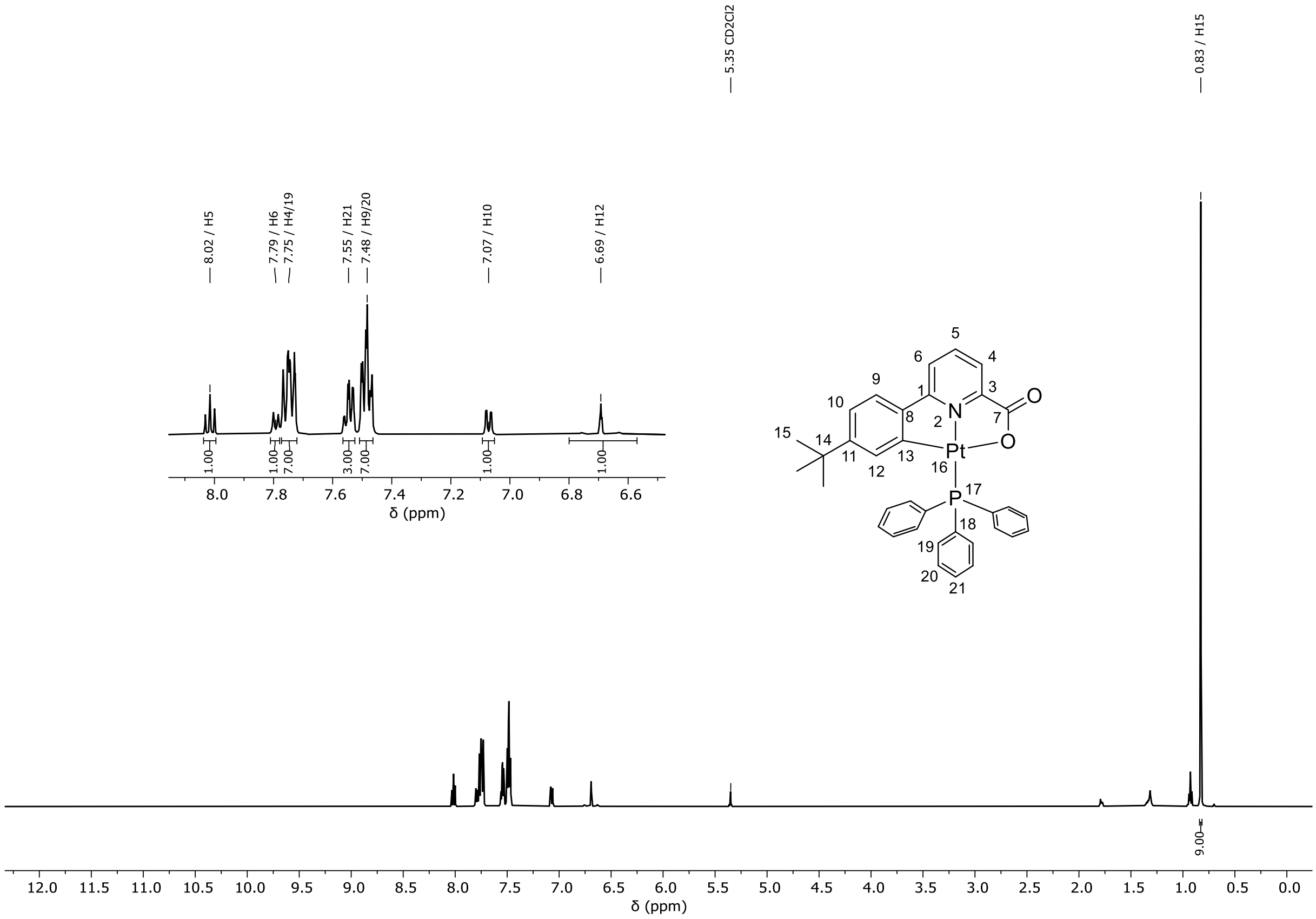

Figure S-146. ^{1}H-NMR spectrum (500 MHz, DCM-d_2) of **Pt(L$_{tBu}$)PPh$_3$**.

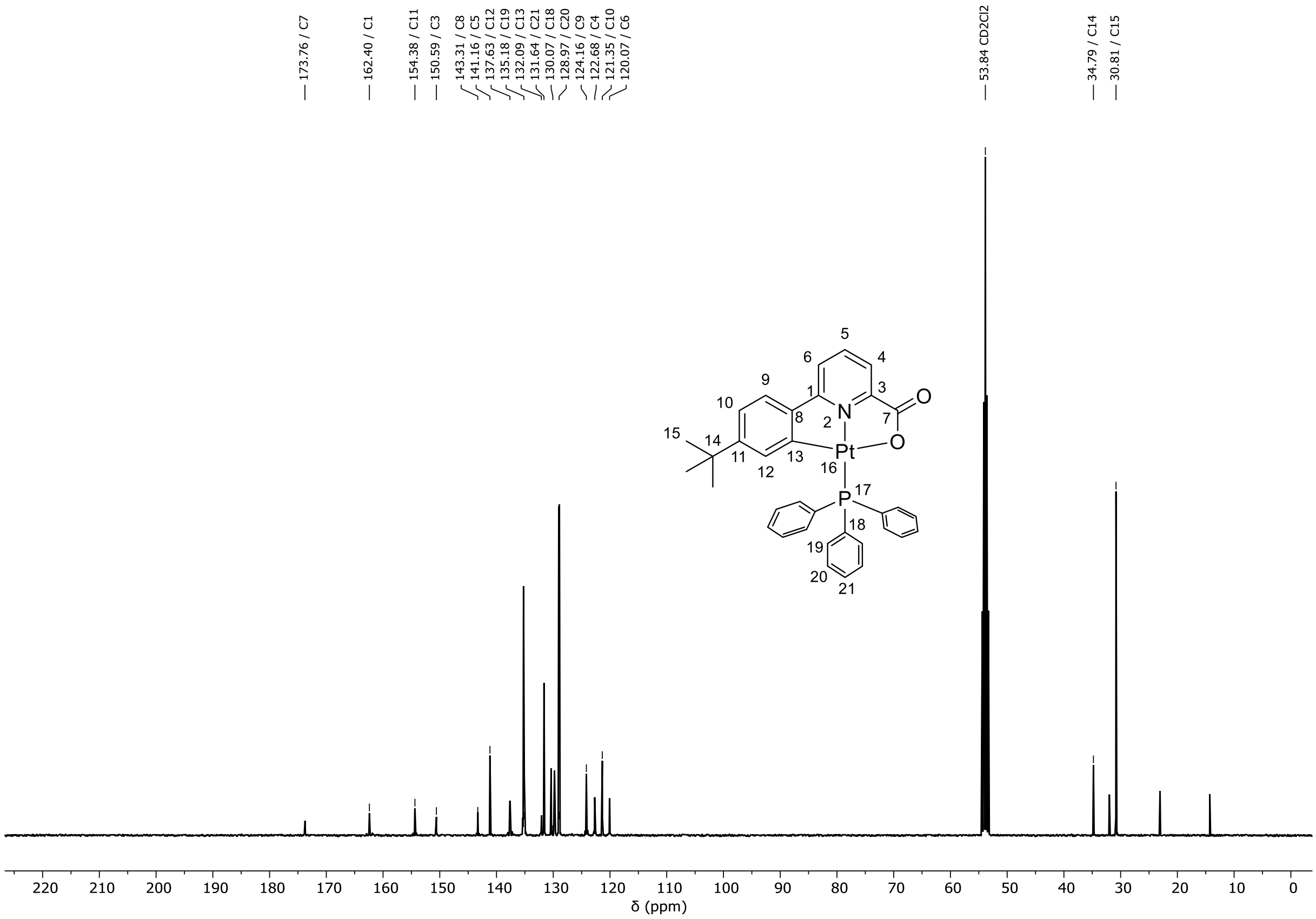

Figure S-147. $^{13}C\{^1H\}$-NMR spectrum (101 MHz, DCM-d_2) of **Pt(L$_{tBu}$)PPh$_3$**.

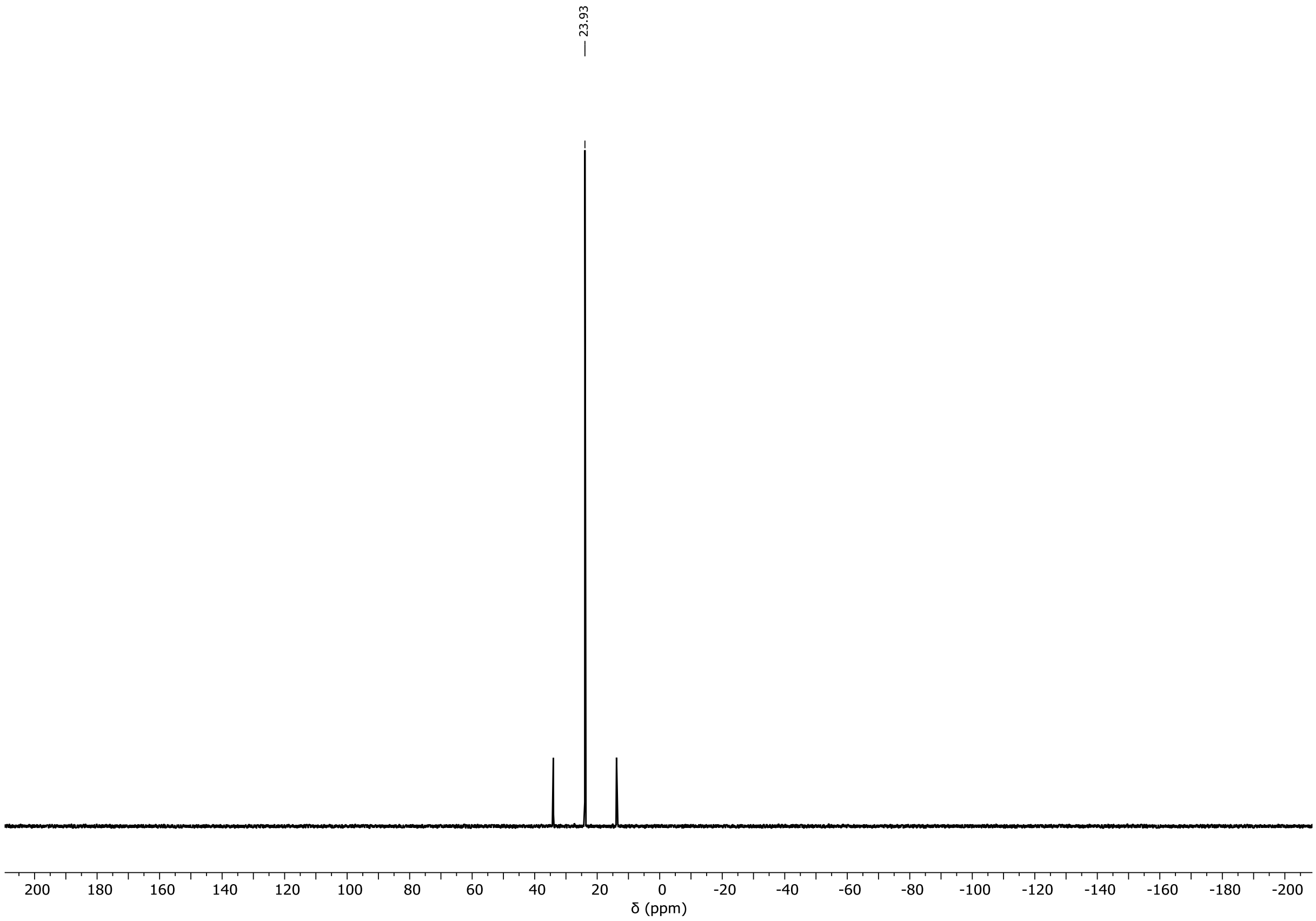

Figure S-148. $^{31}P\{^1H\}$-NMR spectrum (162 MHz, DCM-d_2) of **Pt(L$_{tBu}$)PPh$_3$**.

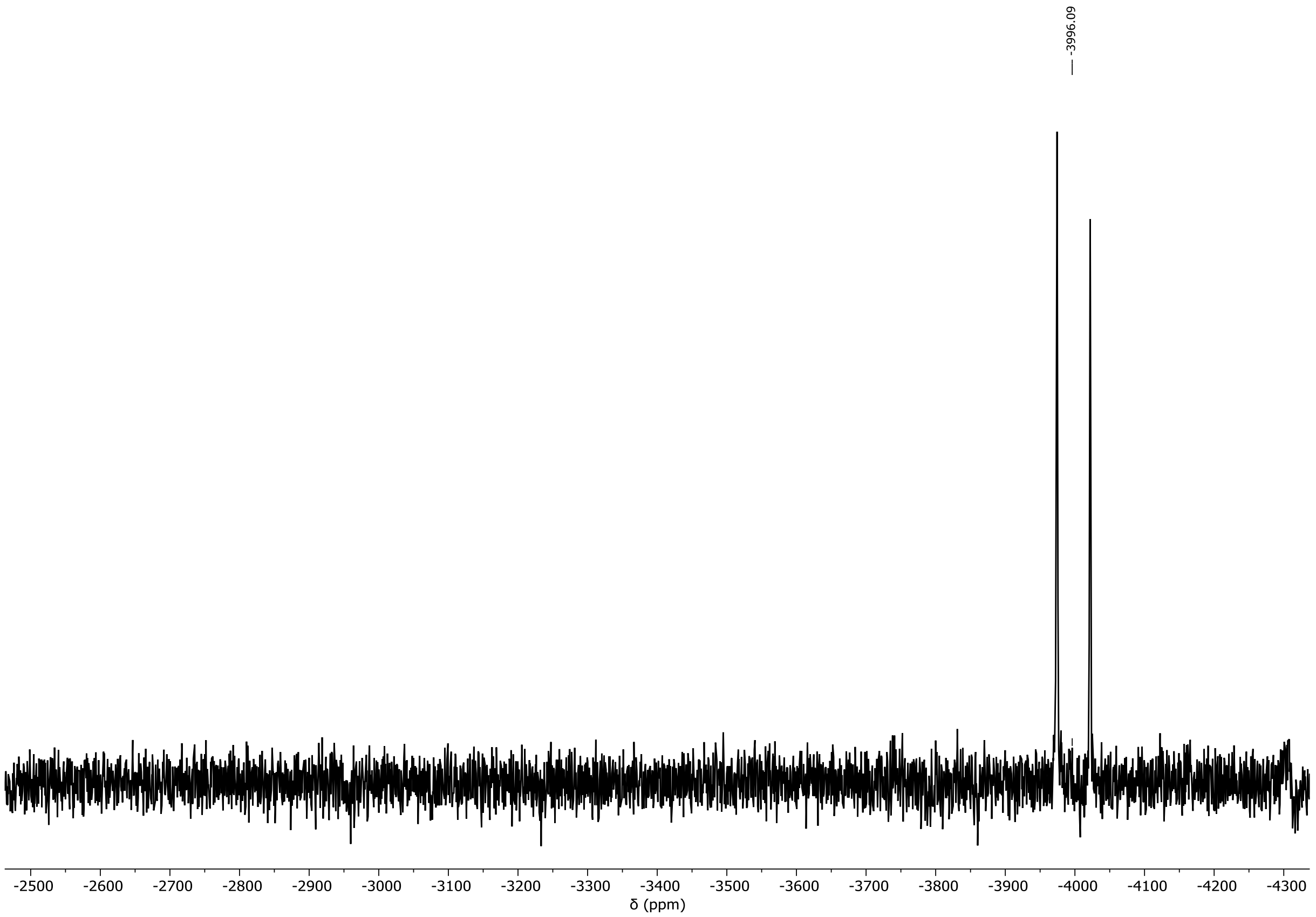

Figure S-149. ^{195}Pt-NMR spectrum (86 MHz, DCM-d_2) of $\mathbf{Pt(L_{tBu})PPh_3}$.

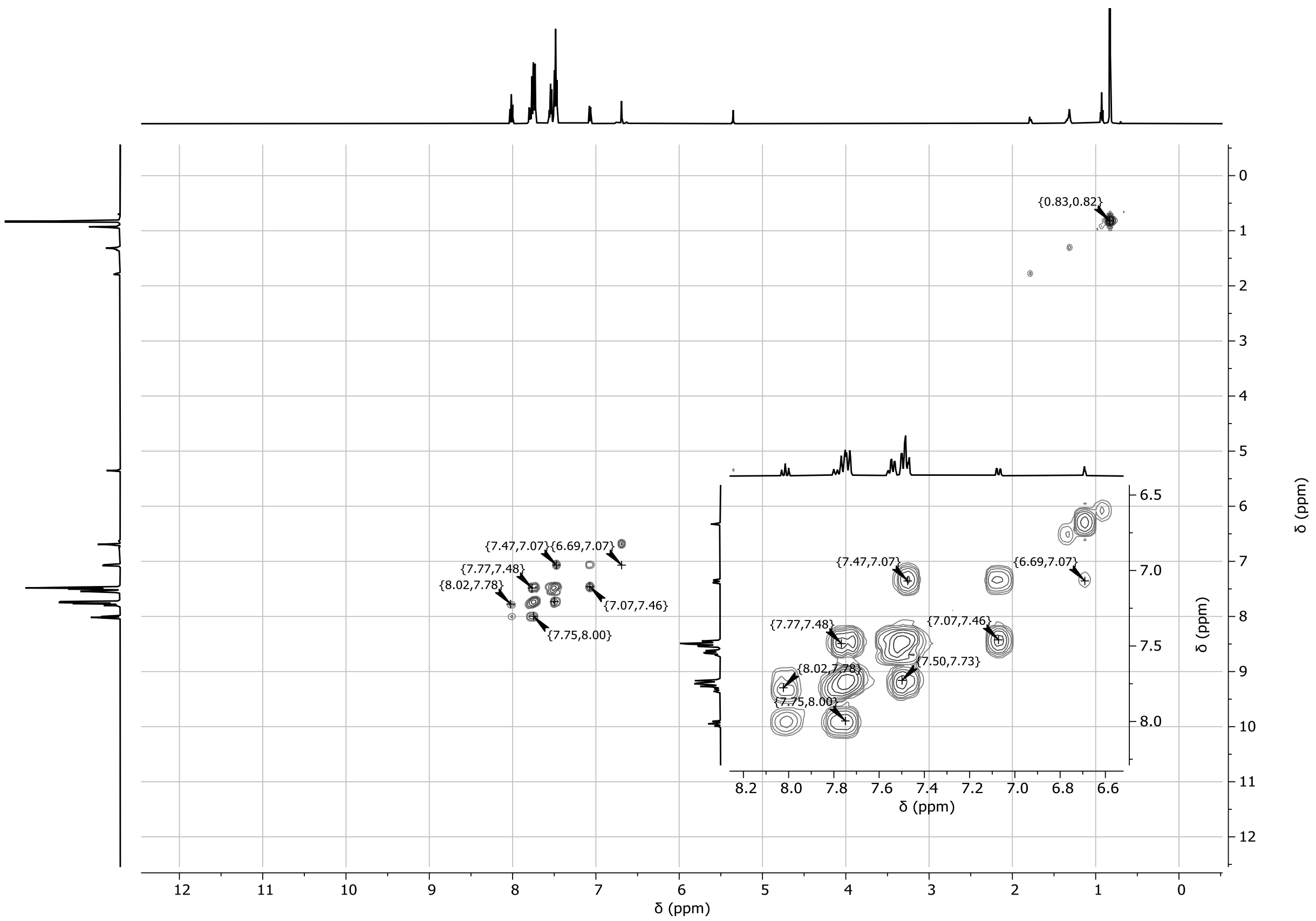

Figure S-150. HH-COSY-NMR spectrum (400 MHz, DCM-d_2) of $\mathbf{Pt(L_{tBu})PPh_3}$.

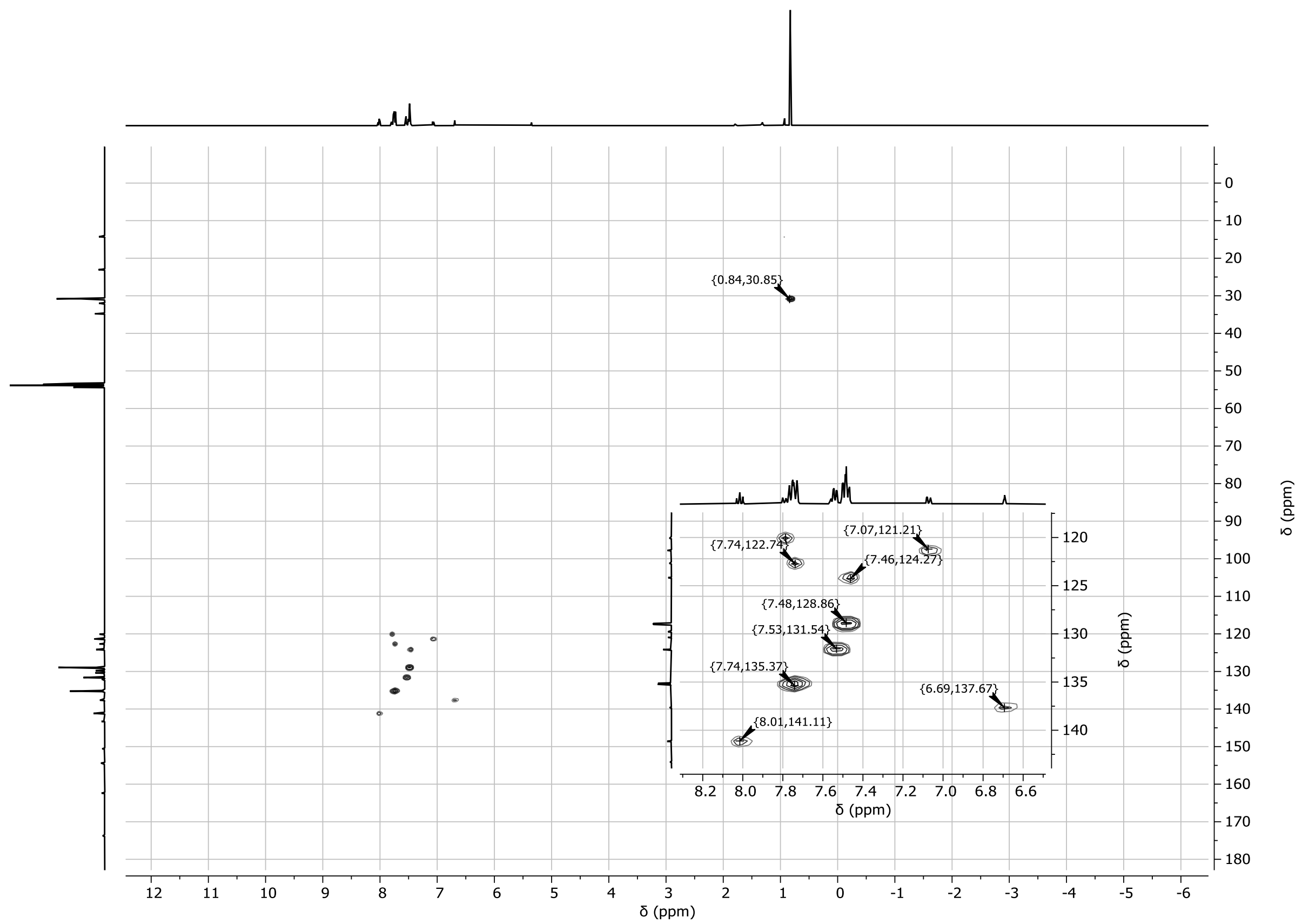

Figure S-151. HC-HSQC-NMR spectrum (101 MHz (^{13}C), 400 MHz (^{1}H), DCM-d_2) of **Pt(L$_{tBu}$)PPh$_3$**.

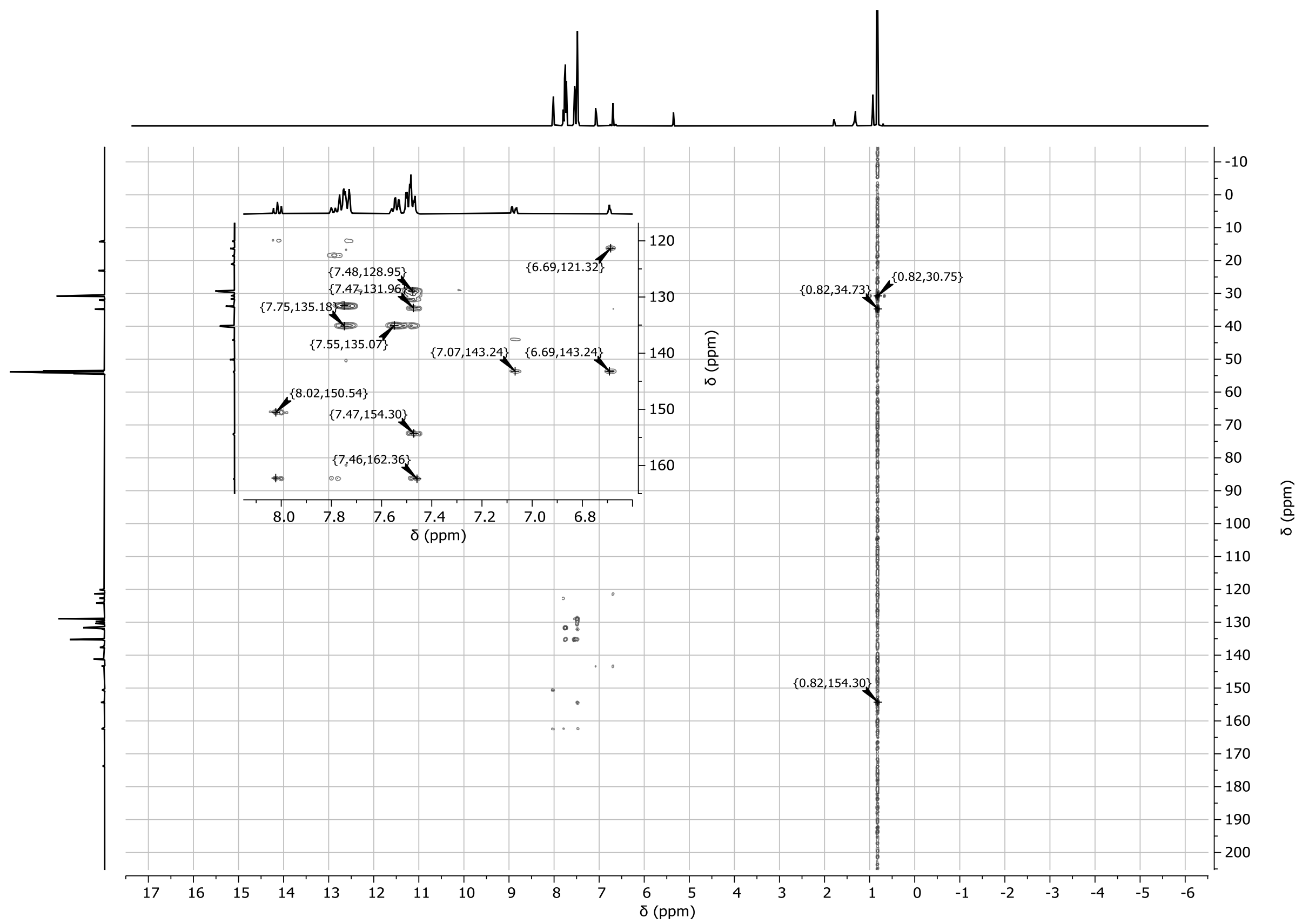

Figure S-152. HC-HMBC-NMR spectrum (101 MHz (^{13}C), 400 MHz (^{1}H), DCM-d_2) of **Pt(L$_{tBu}$)PPh$_3$**.

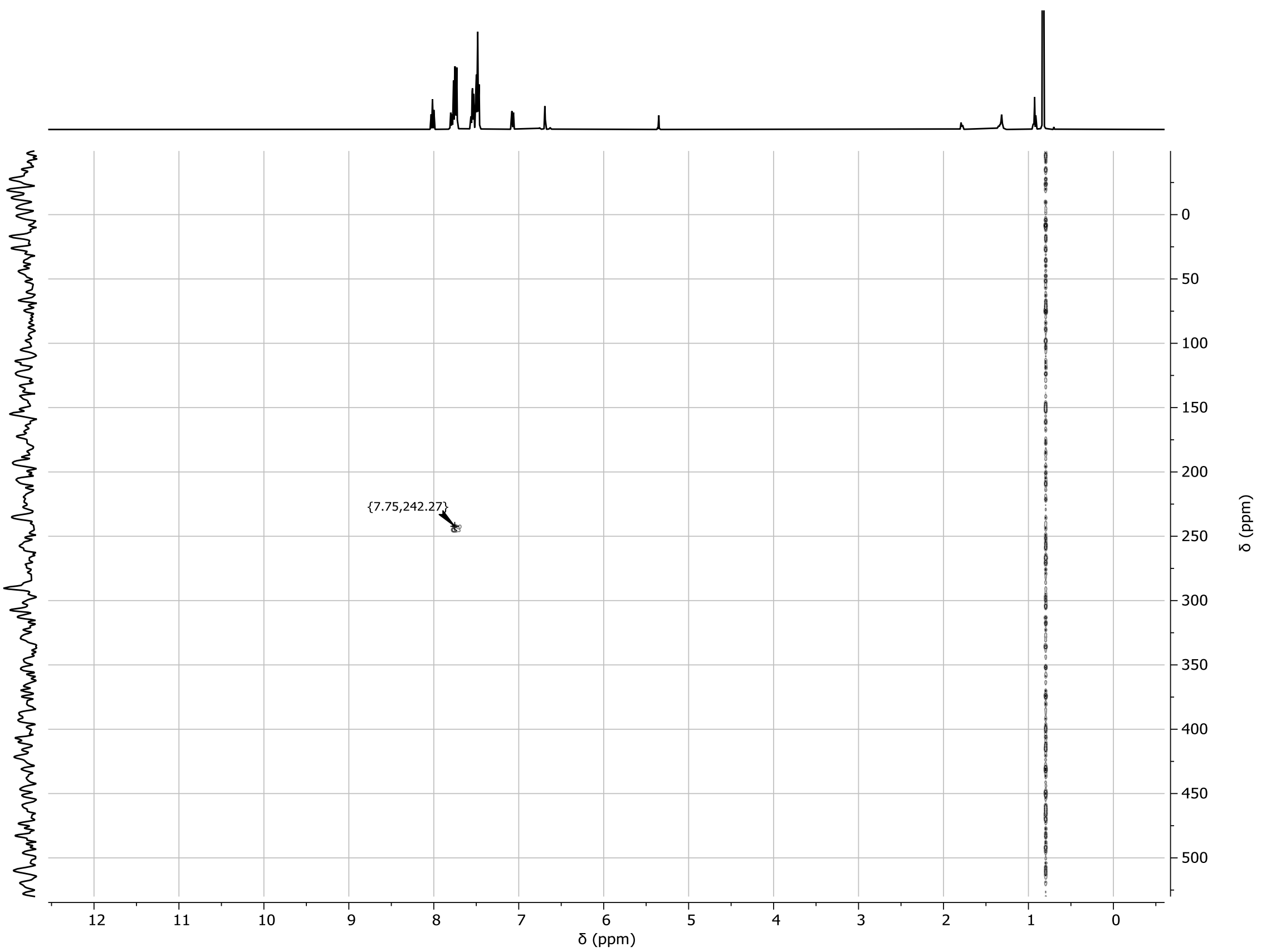

Figure S-153. HN-HMBC-NMR spectrum (41 MHz (^{15}N), 400 MHz (^{1}H), DCM-d_2) of **Pt(L$_{tBu}$)PPh$_3$**.

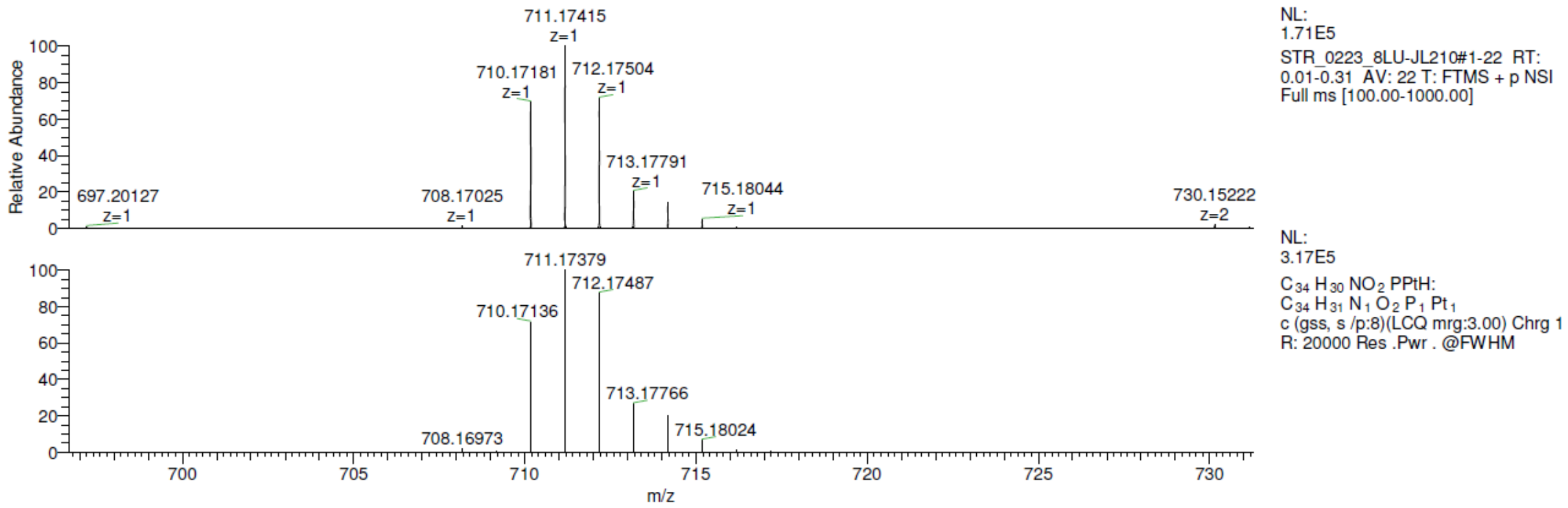

Figure S-154. Mass spectrum of **Pt(L$_{tBu}$)PPh$_3$** (MeOH). Additional simulation of the [**Pt(L$_{tBu}$)PPh$_3$**+H]$^+$ adduct.

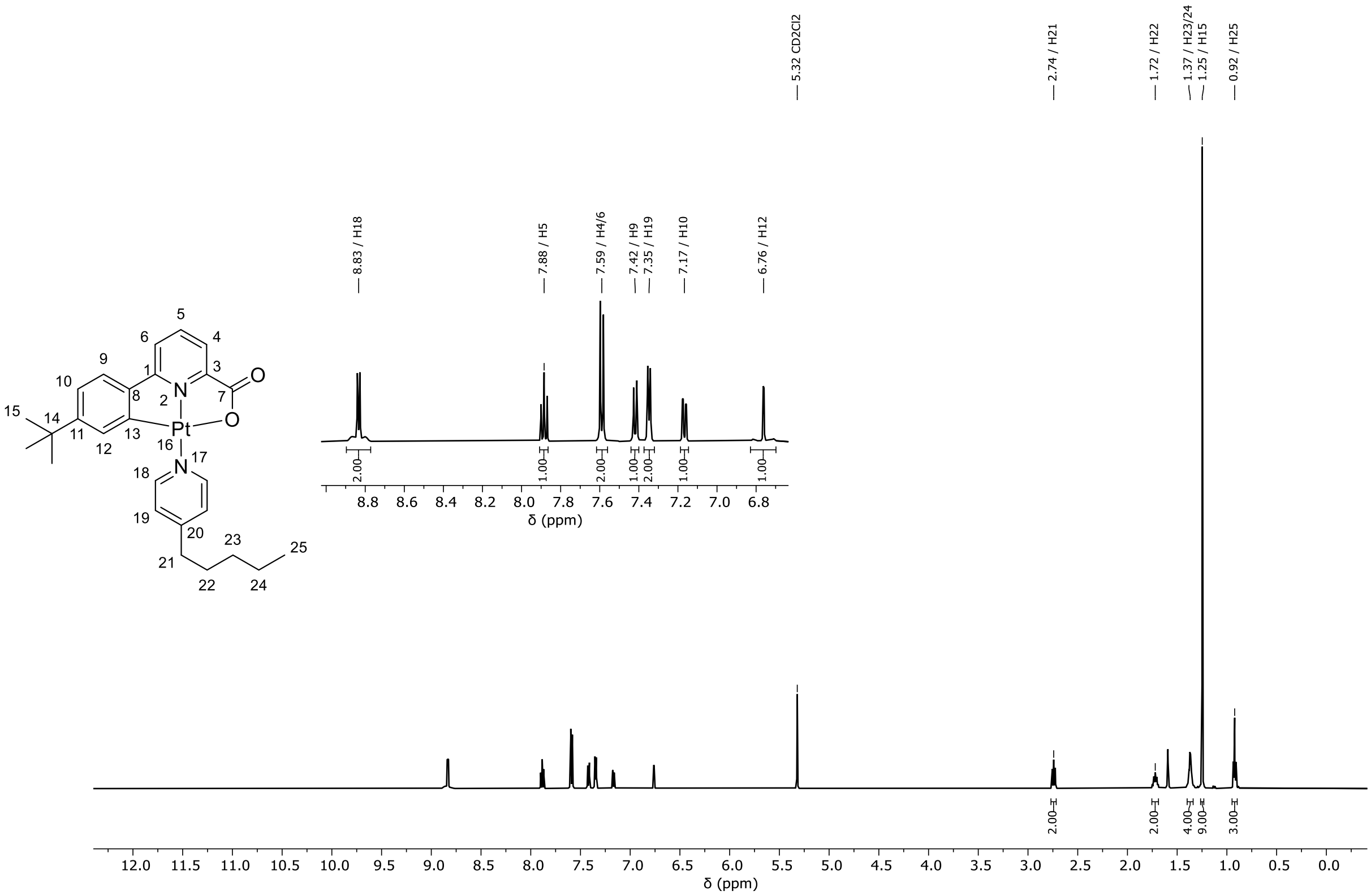

Figure S-155. ^{1}H-NMR spectrum (500 MHz, DCM-d_2) of **Pt(L$_{tBu}$)AmPy**.

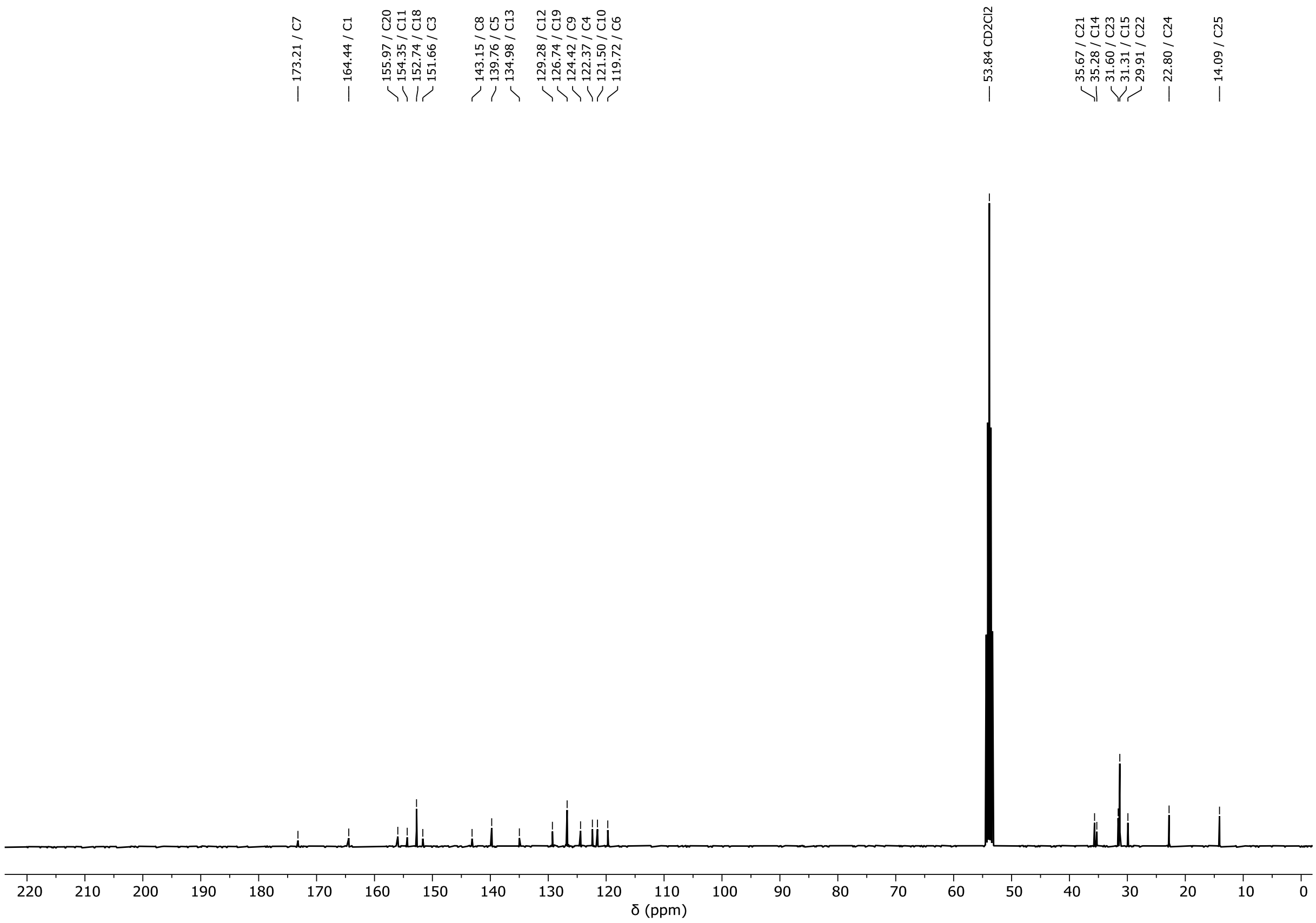

Figure S-156. ^{13}C{^{1}H}-NMR spectrum (101 MHz, DCM-d_2) of **Pt(L$_{tBu}$)AmPy**.

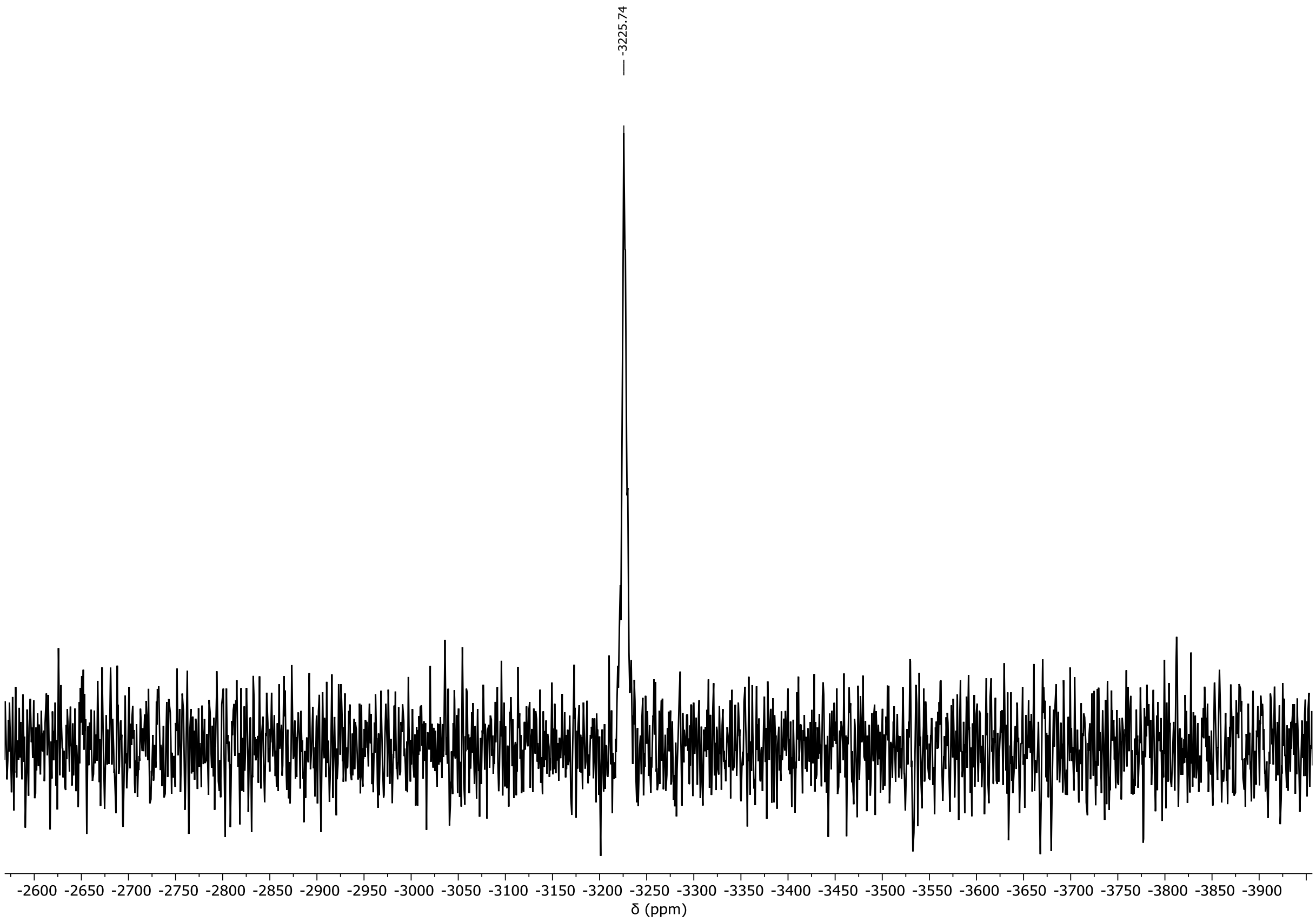

Figure S-157. ^{195}Pt-NMR spectrum (86 MHz, DCM-d_2) of **Pt(L$_{tBu}$)AmPy**.

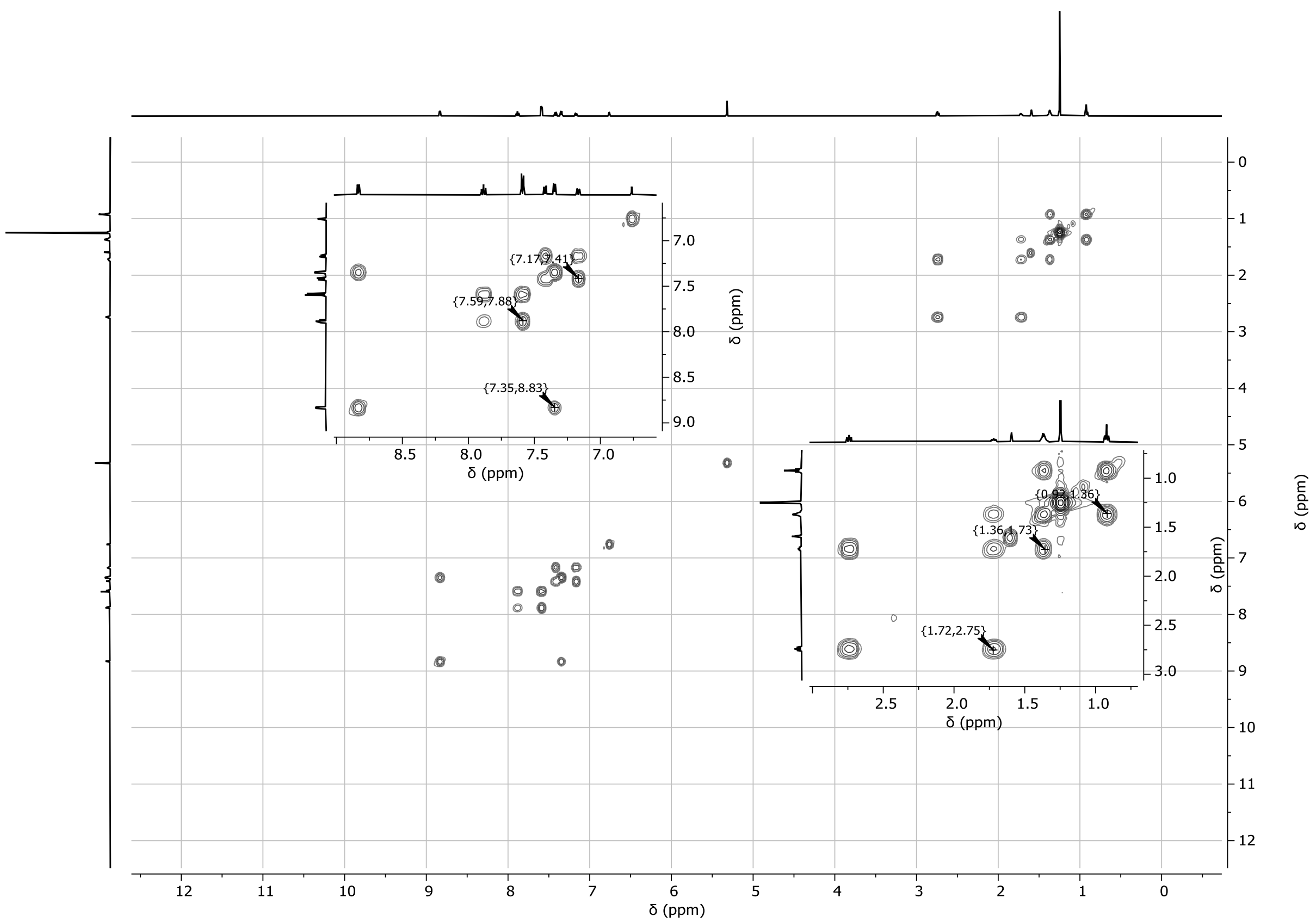

Figure S-158. HH-COSY-NMR spectrum (400 MHz, DCM-d_2) of **Pt(L$_{tBu}$)AmPy**.

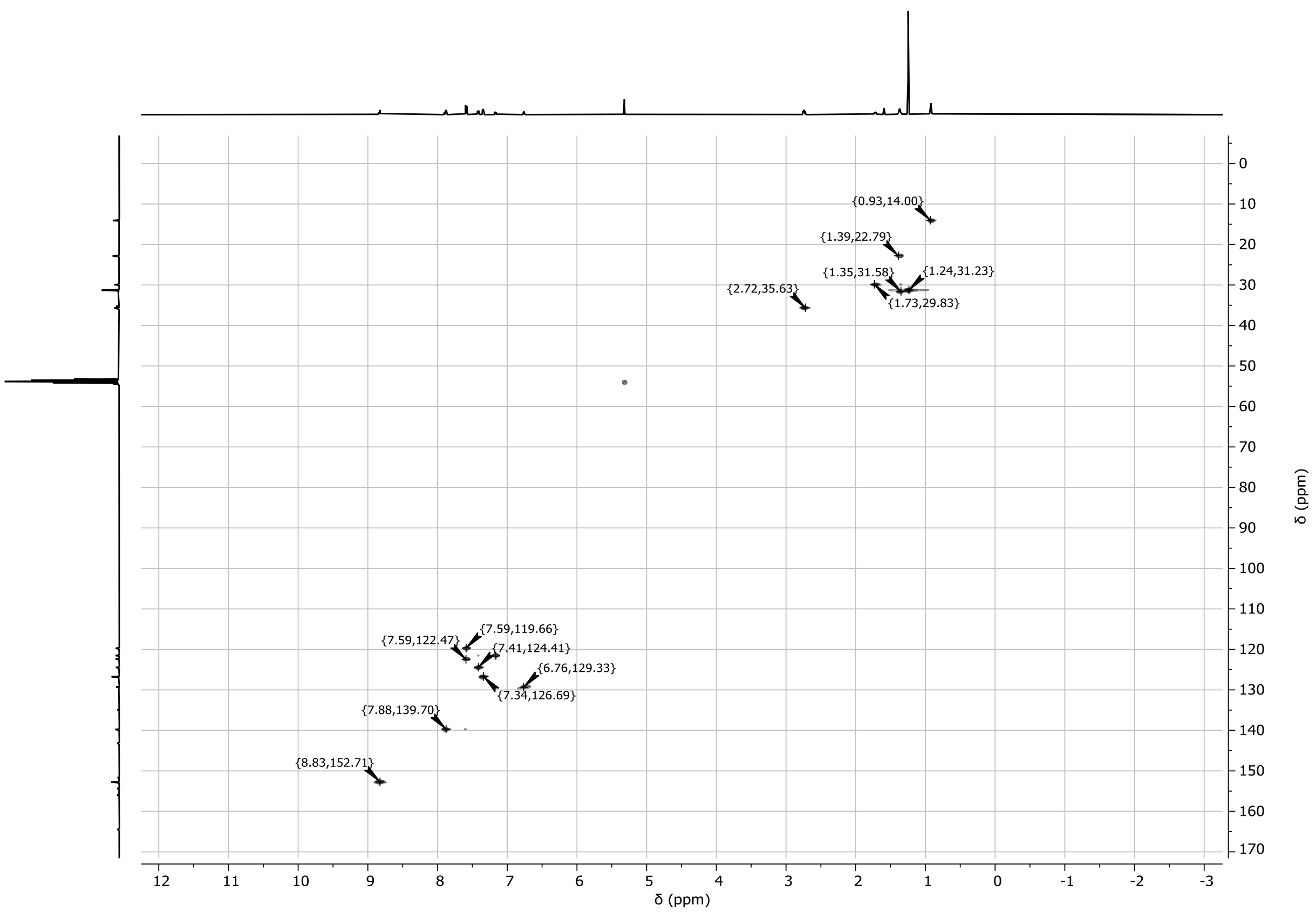

Figure S-159. HC-HSQC-NMR spectrum (101 MHz (^{13}C), 400 MHz (^{1}H), DCM-d_2) of **Pt(L$_{tBu}$)AmPy**.

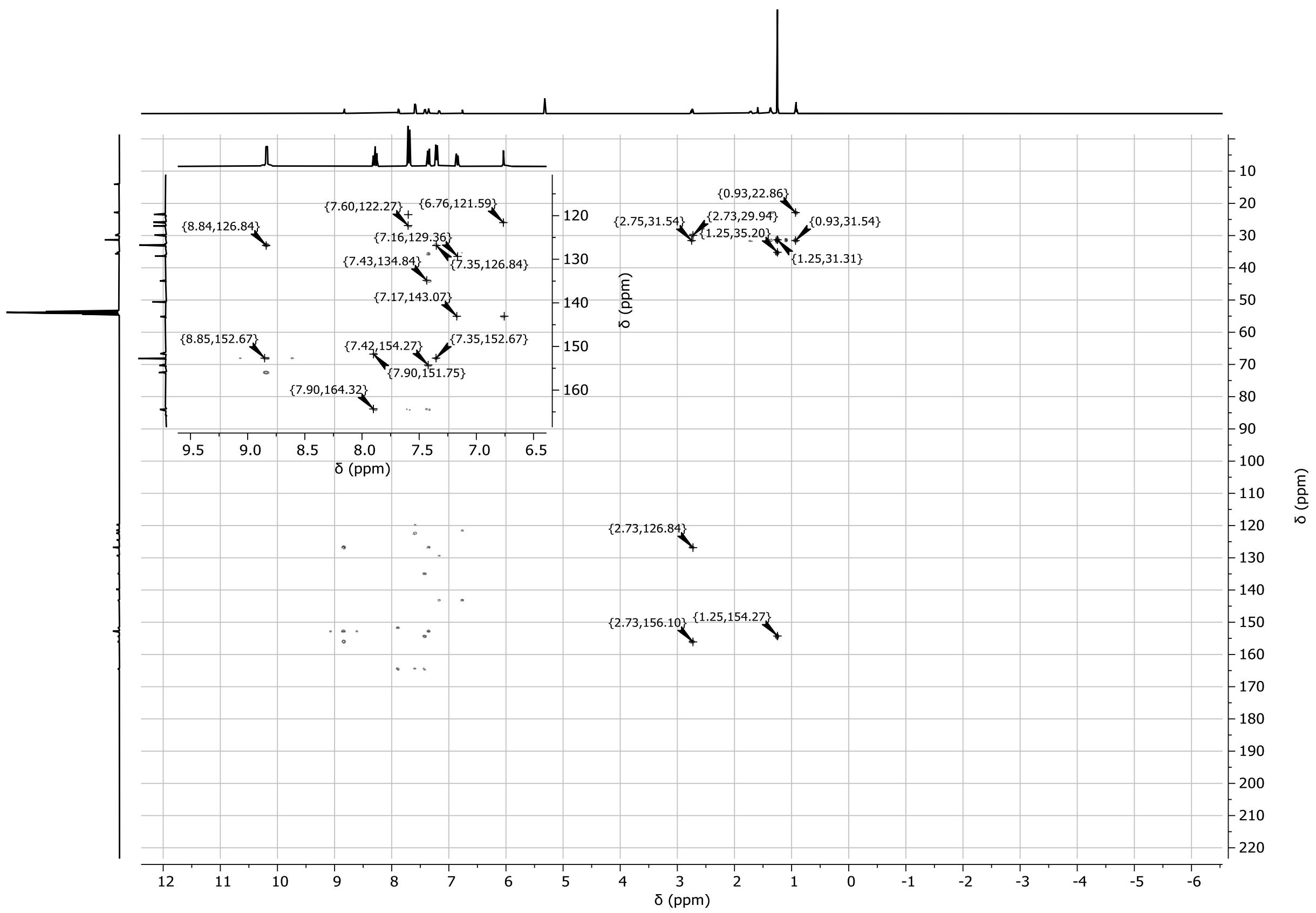

Figure S-160. HC-HMBC-NMR spectrum (101 MHz (^{13}C), 400 MHz (^{1}H), DCM-d_2) of **Pt(L$_{tBu}$)AmPy**.

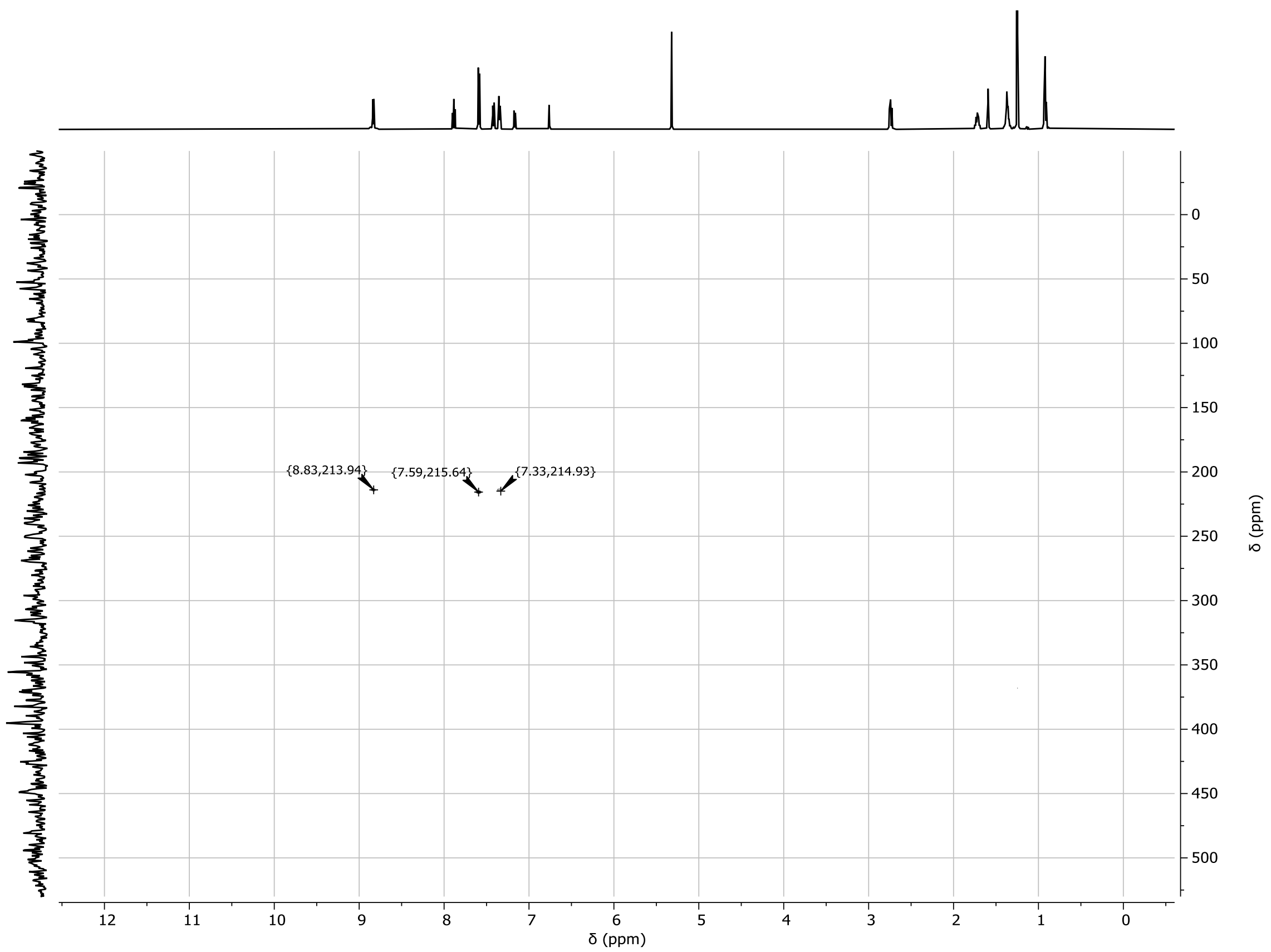

Figure S-161. HN-HMBC-NMR spectrum (41 MHz (^{15}N), 400 MHz (^{1}H), DCM-d_2) of **Pt(L$_{tBu}$)AmPy**.

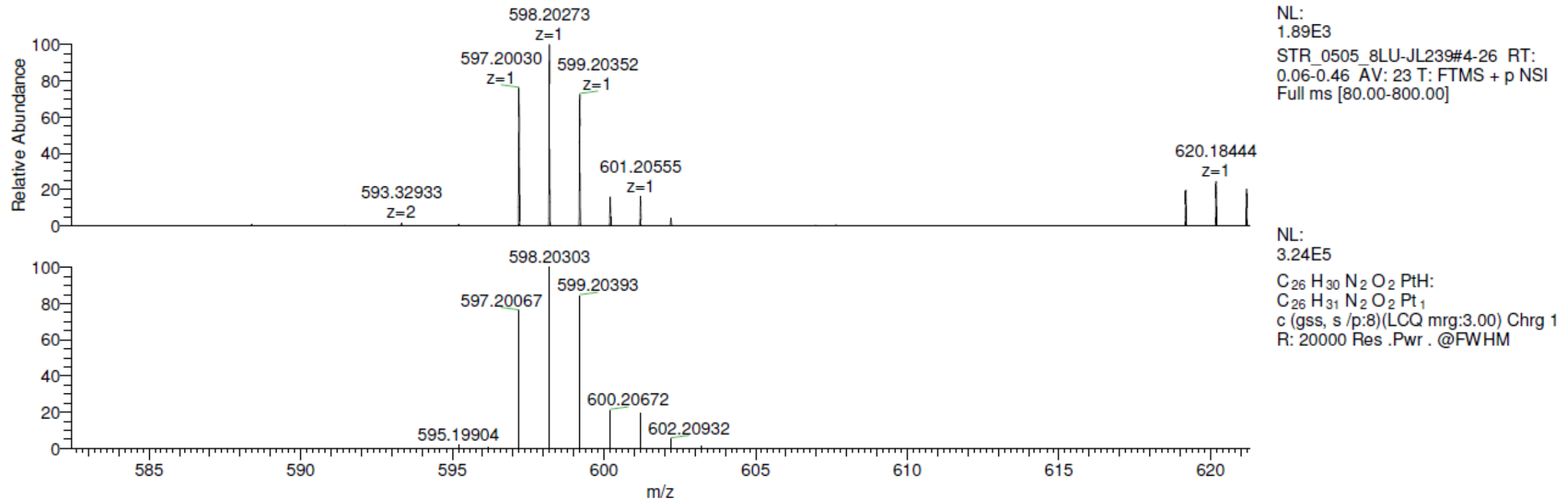

Figure S-162. Mass spectrum of **Pt(L$_{tBu}$)AmPy** (MeOH). Additional simulation of the [**Pt(L$_{tBu}$)AmPy**+H]$^+$ adduct.

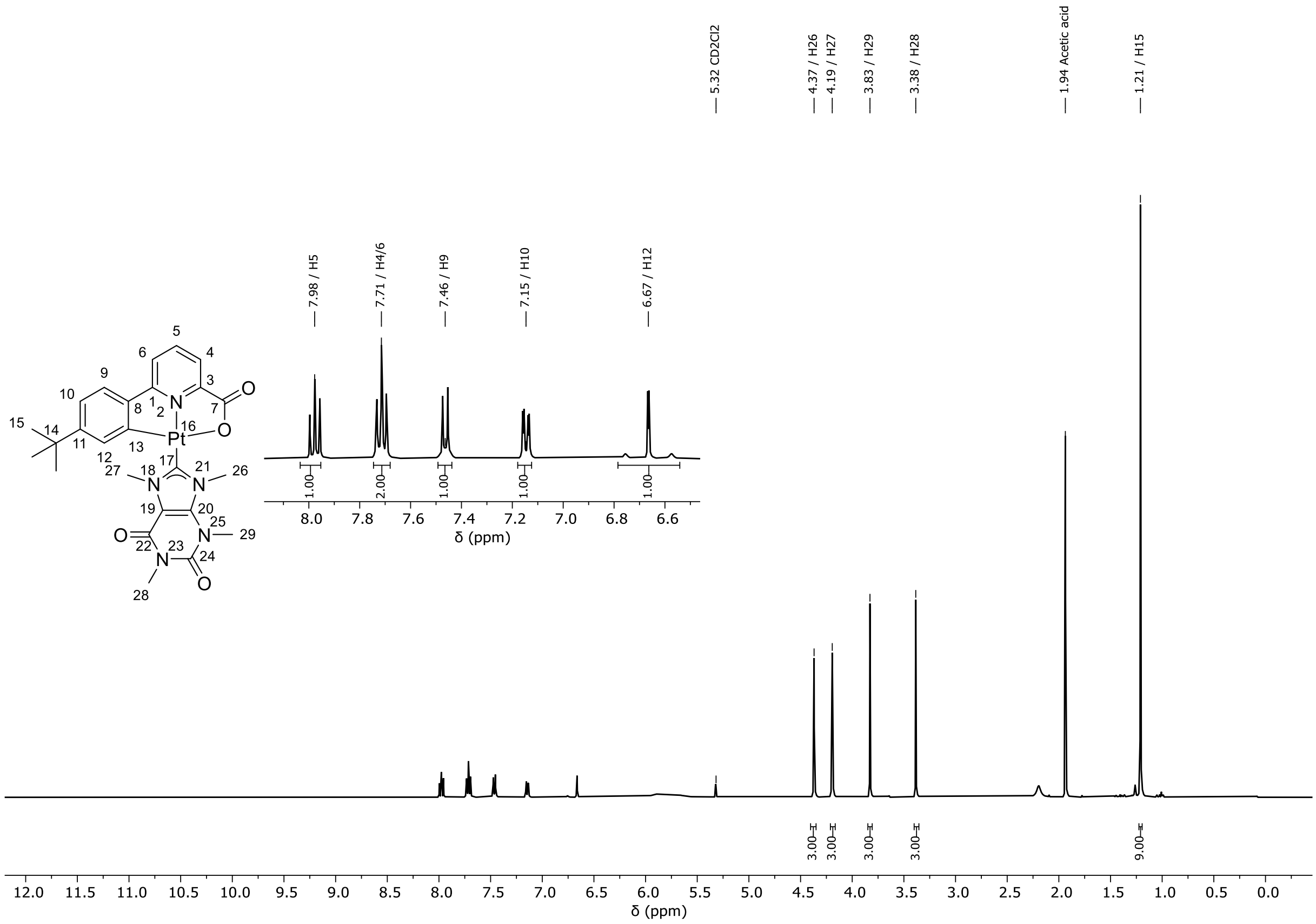

Figure S-163. ^{1}H-NMR spectrum (400 MHz, DCM-d_2) of **Pt(L$_{tBu}$)NHC**.

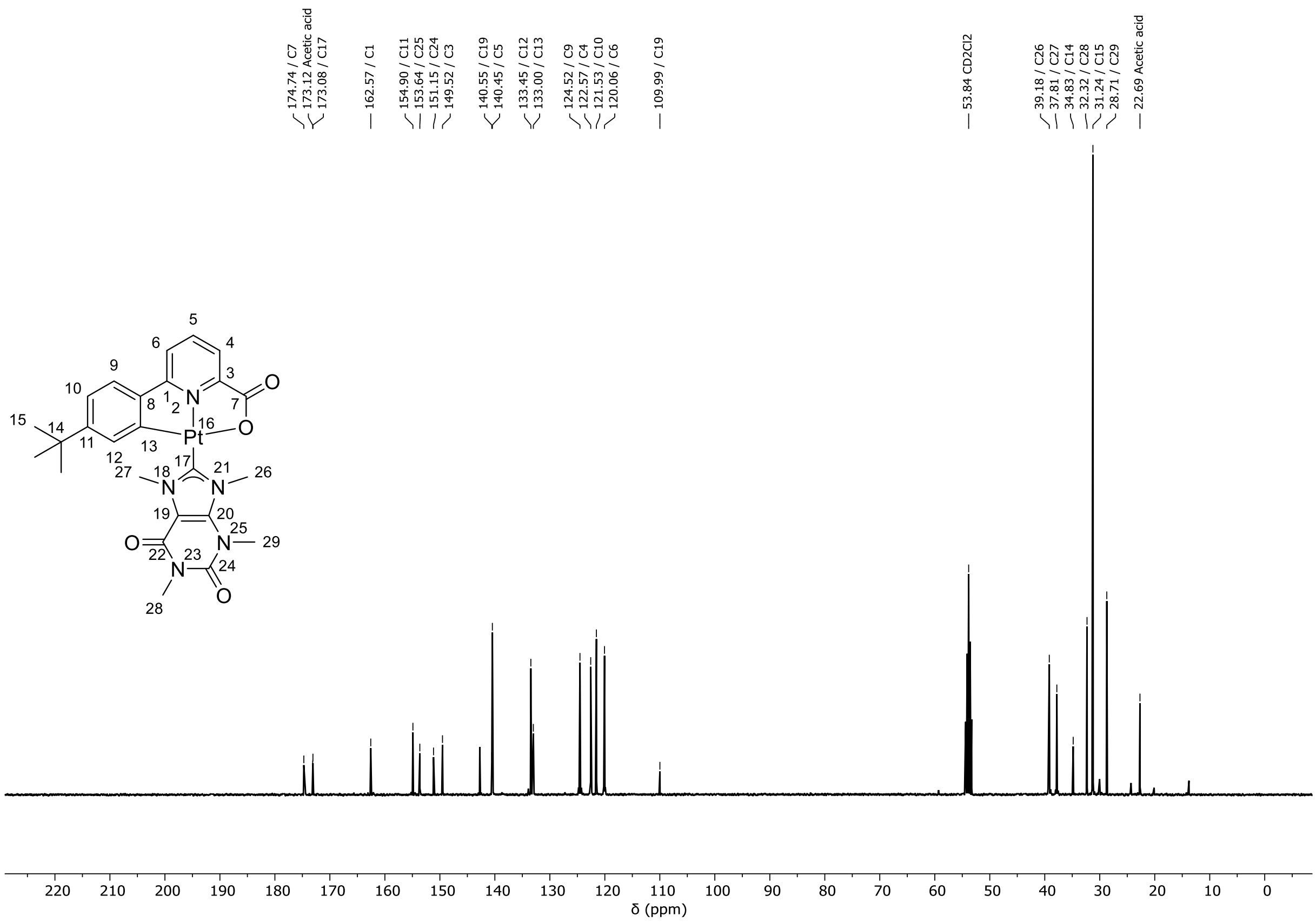

Figure S-164. ^{13}C{^{1}H}-NMR spectrum (101 MHz, DCM-d_2) of **Pt(L$_{tBu}$)NHC**.

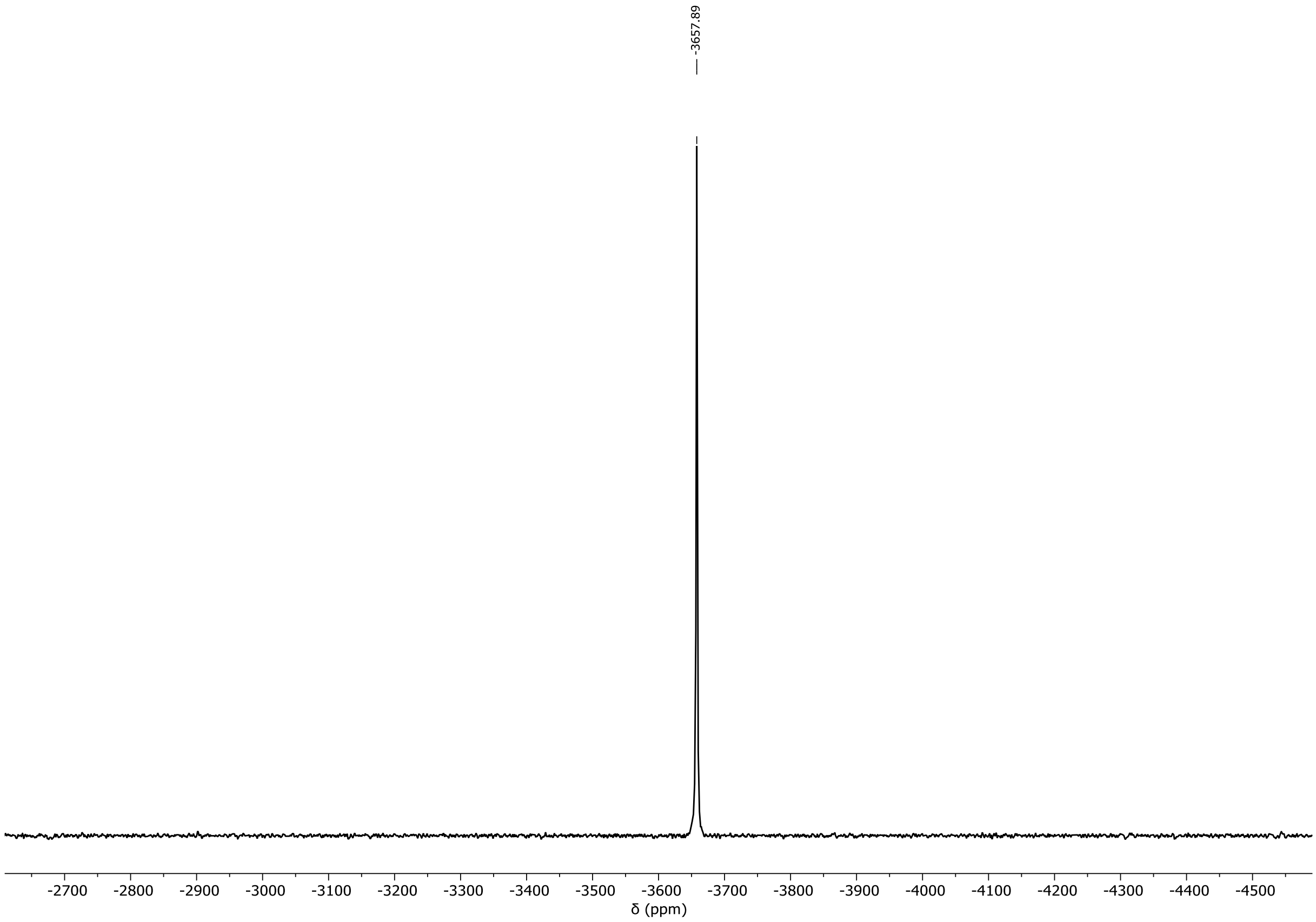

Figure S-165. ^{195}H-NMR spectrum (86 MHz, DCM-d_2) of **Pt(L$_{tBu}$)NHC**.

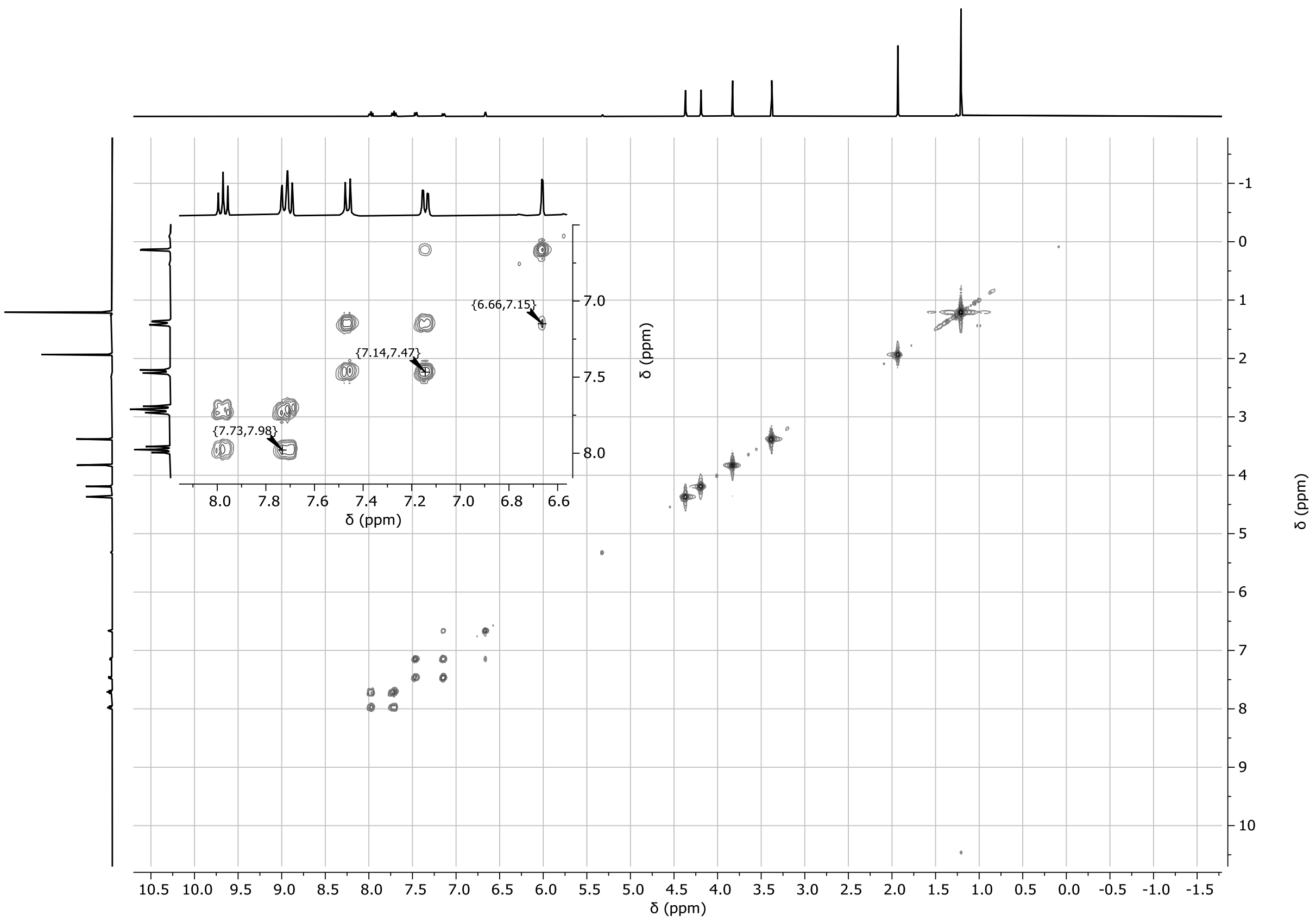

Figure S-166. HH-COSY-NMR spectrum (400 MHz, DCM-d_2) of **Pt(L$_{tBu}$)NHC**.

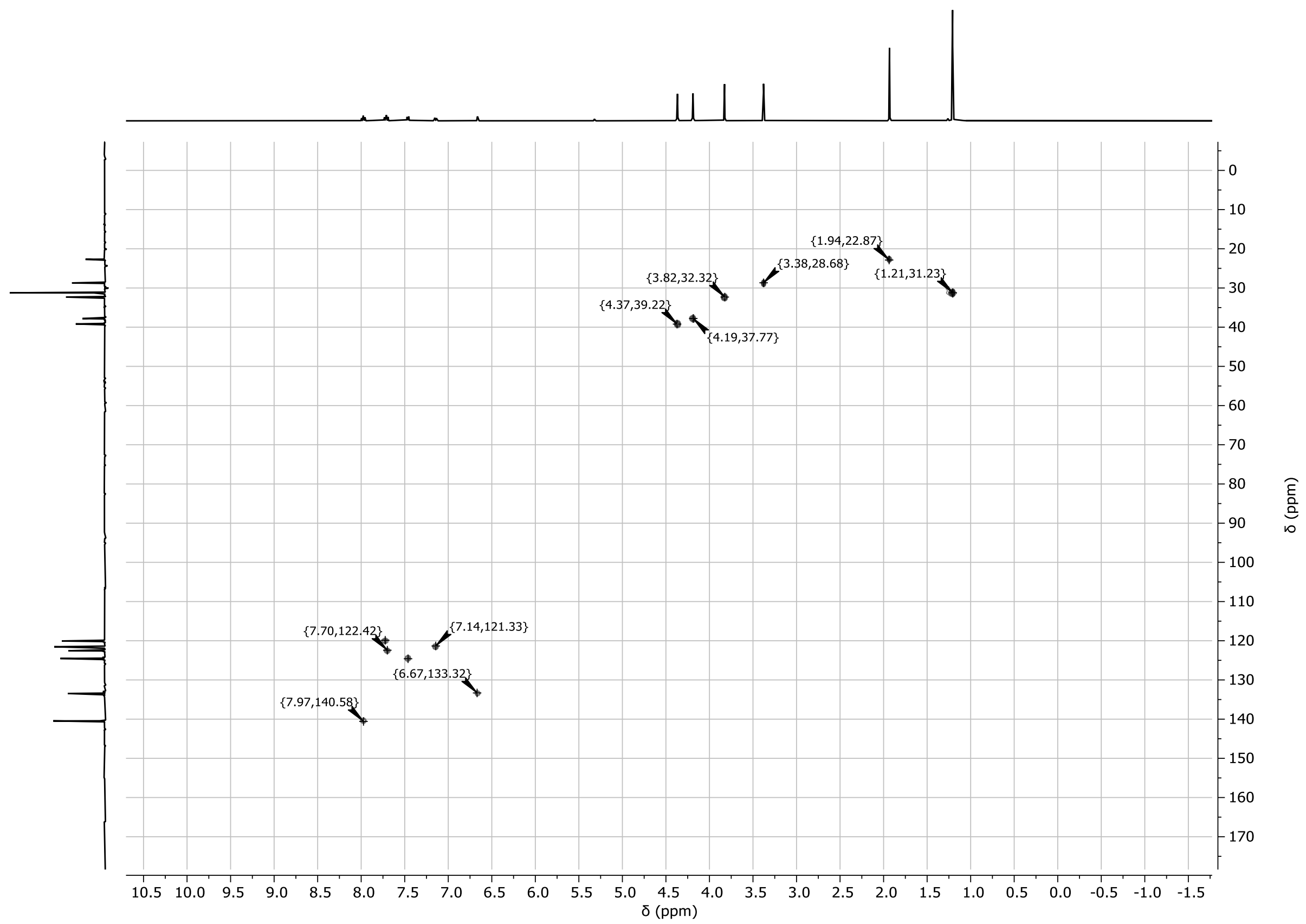

Figure S-167. HC-HSQC-NMR spectrum (101 MHz (^{13}C), 400 MHz (^{1}H), DCM-d_2) of **Pt(L_{tBu})NHC**.

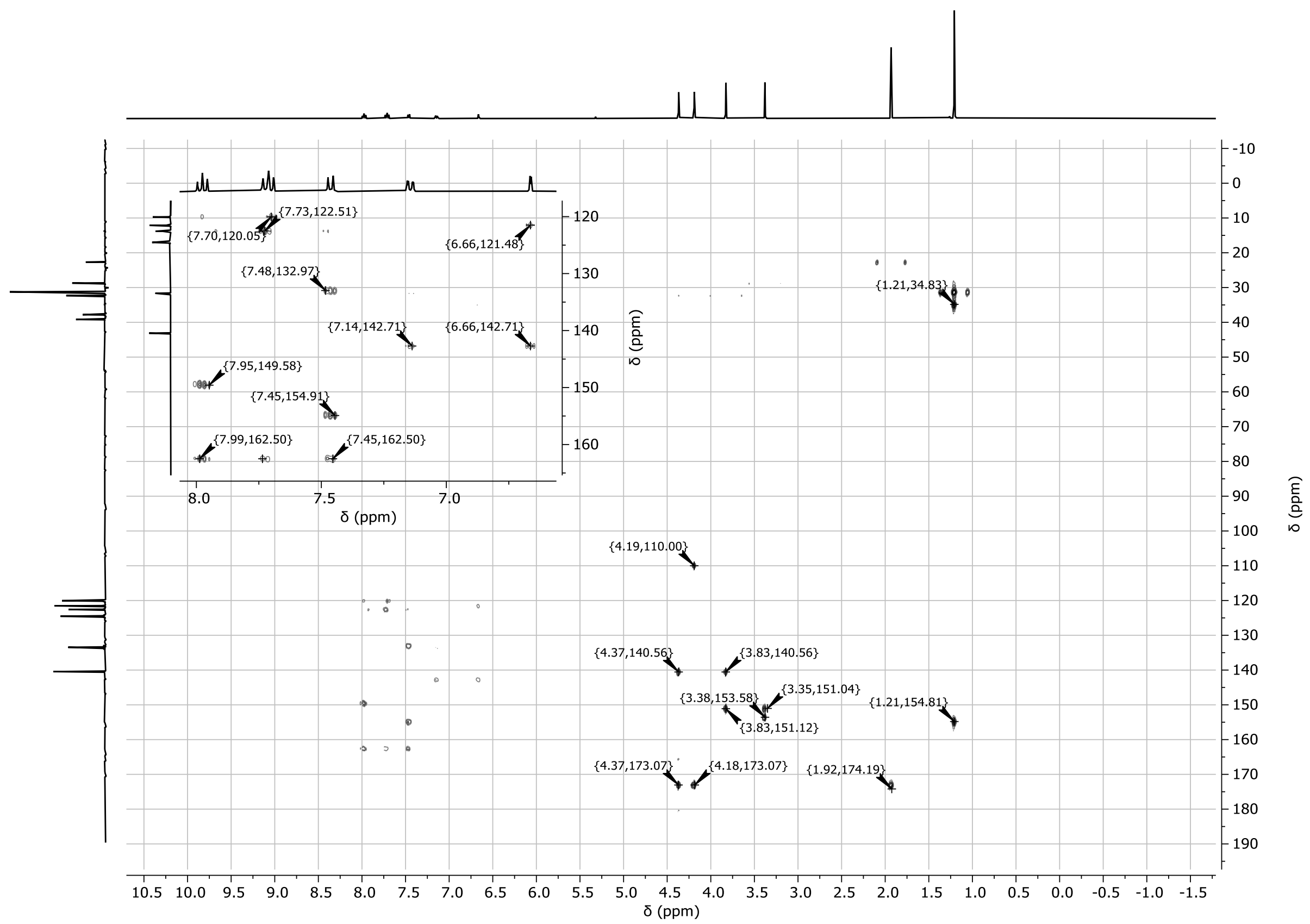

Figure S-168. HC-HMBC-NMR spectrum (101 MHz (^{13}C), 400 MHz (^{1}H), DCM-d_2) of **Pt(L_{tBu})NHC**.

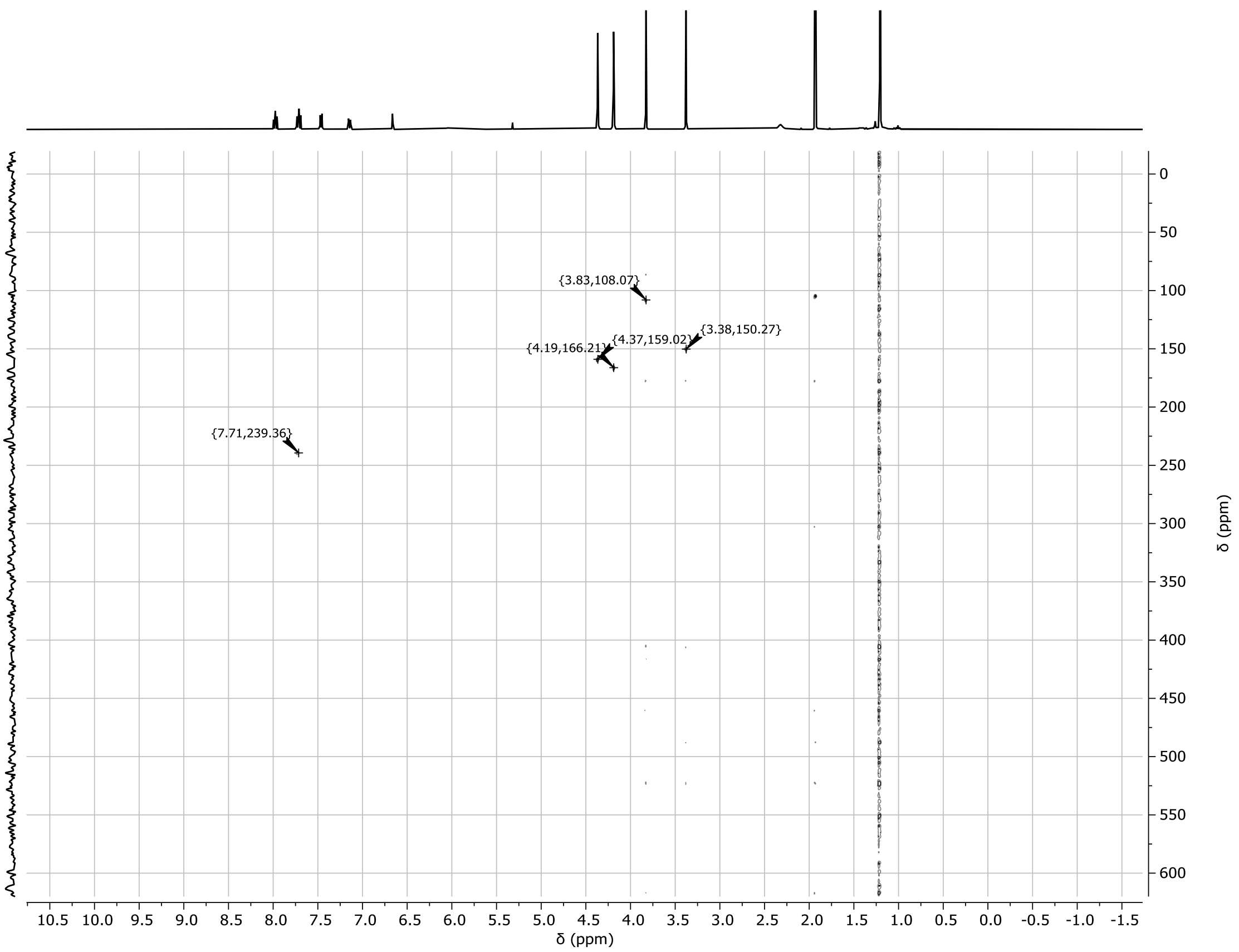

Figure S-169. HN-HMBC-NMR spectrum (101 MHz (^{15}N), 400 MHz (^{1}H), DCM-d_2) of **Pt(L$_{tBu}$)NHC**.

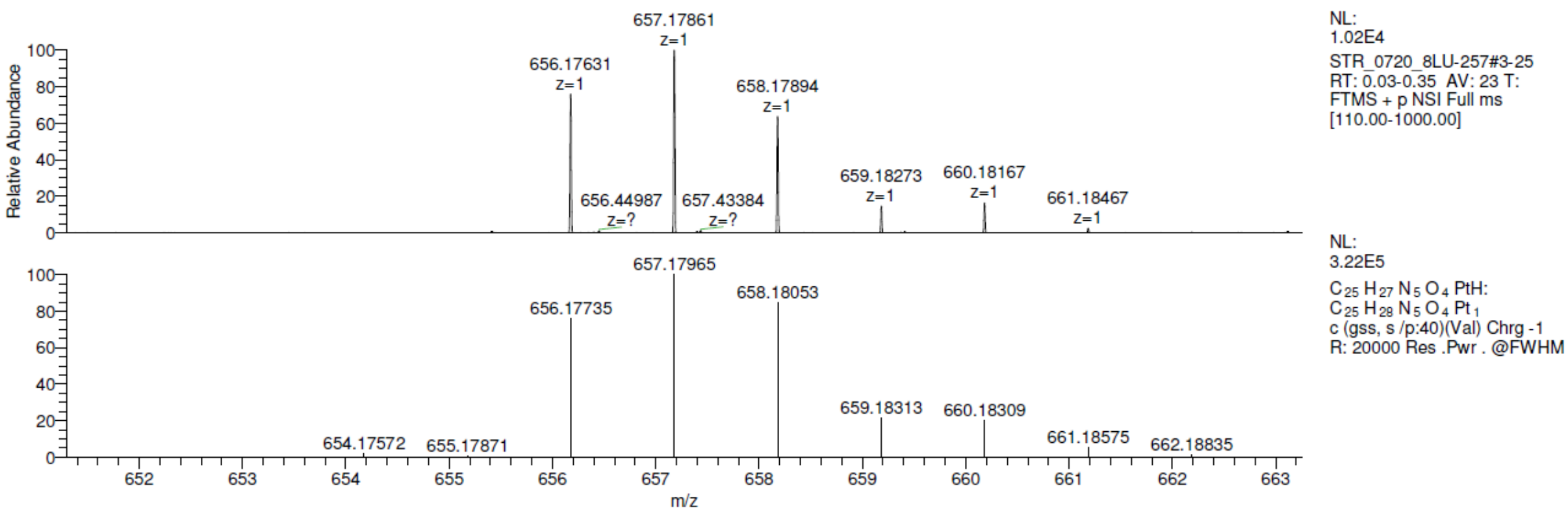

Figure S-170. Mass spectrum of **Pt(L$_{tBu}$)NHC** (MeOH). Additional simulation of the [**Pt(L$_{tBu}$)NHC**+H]$^+$ adduct.

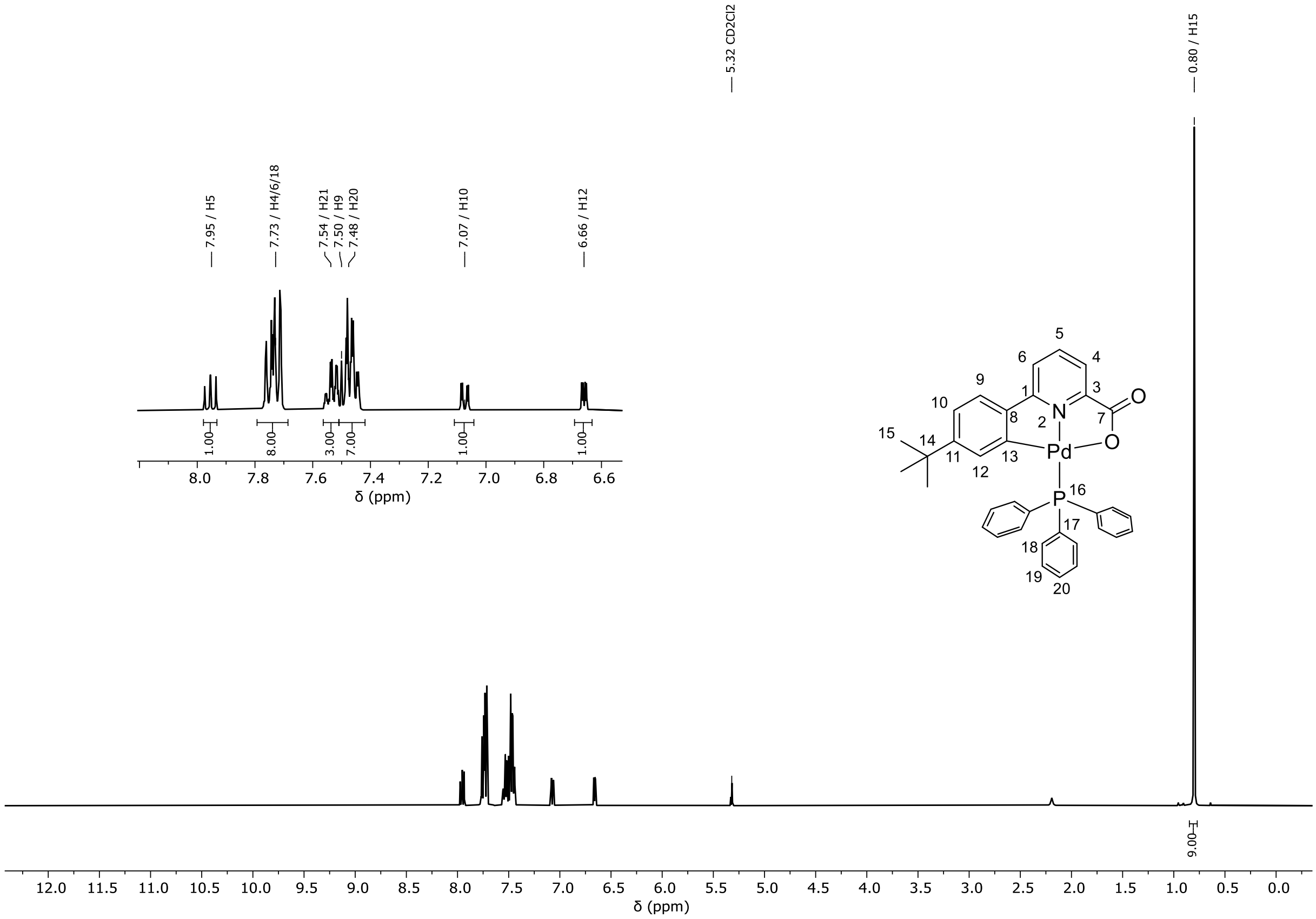

Figure S-171. ^{1}H-NMR spectrum (400 MHz, DCM-d_2) of **Pd(L$_{tBu}$)PPh$_3$**.

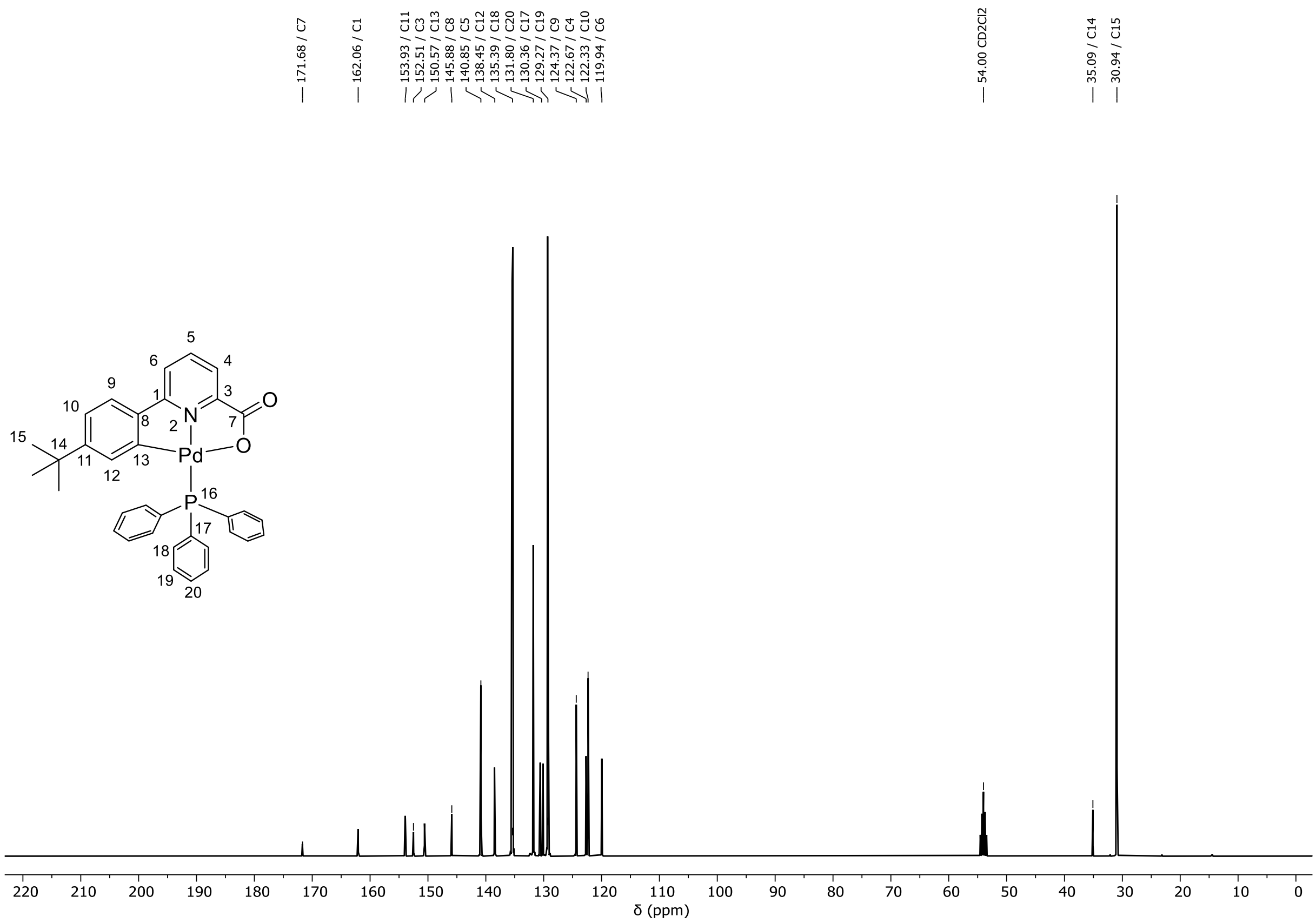

Figure S-172. ^{13}C{^{1}H}-NMR spectrum (101 MHz, DCM-d_2) of **Pd(L$_{tBu}$)PPh$_3$**.

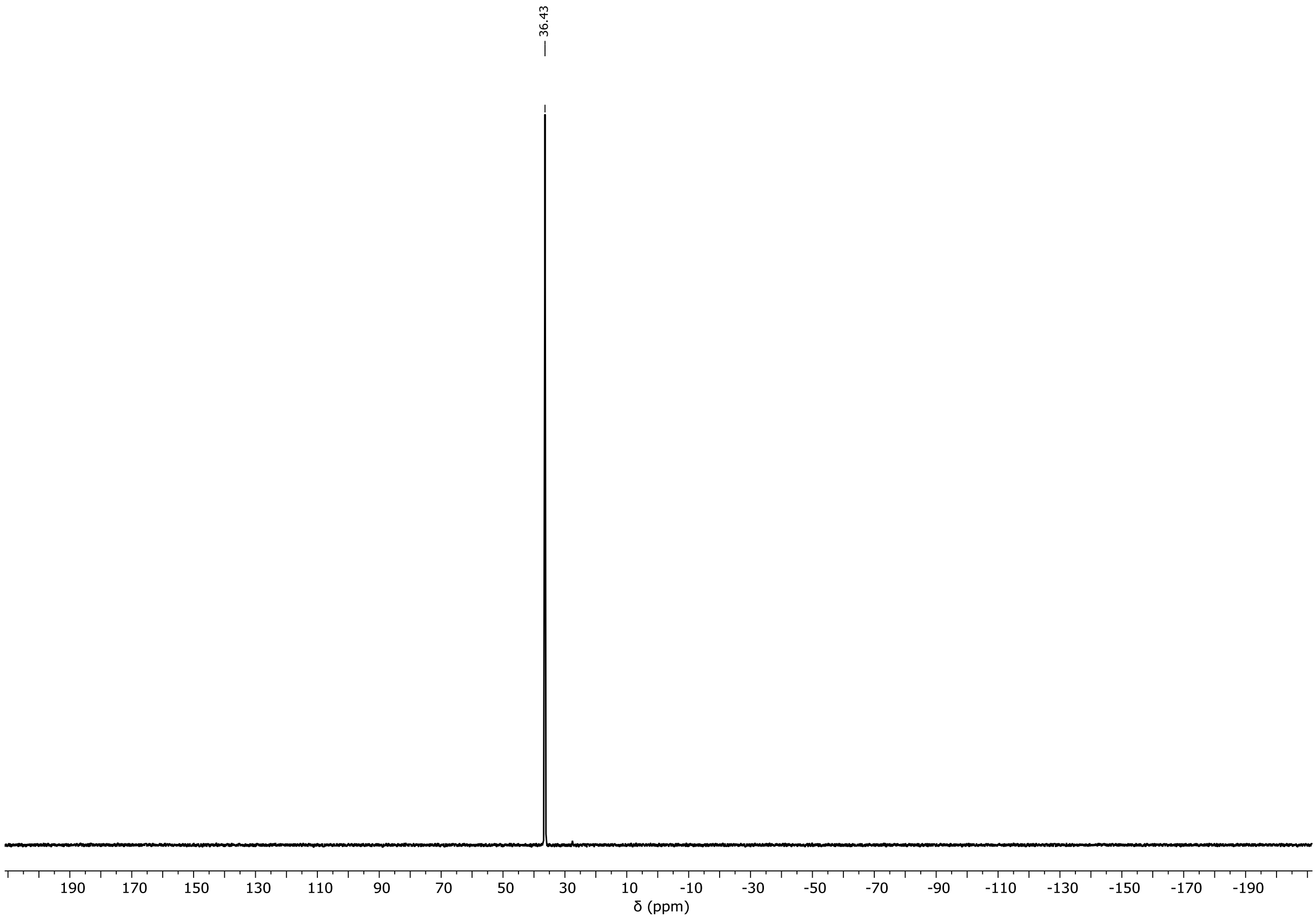

Figure S-173. $^{31}P\{^{1}H\}$-NMR spectrum (162 MHz, DCM-d_2) of **Pd(L$_{tBu}$)PPh$_3$**.

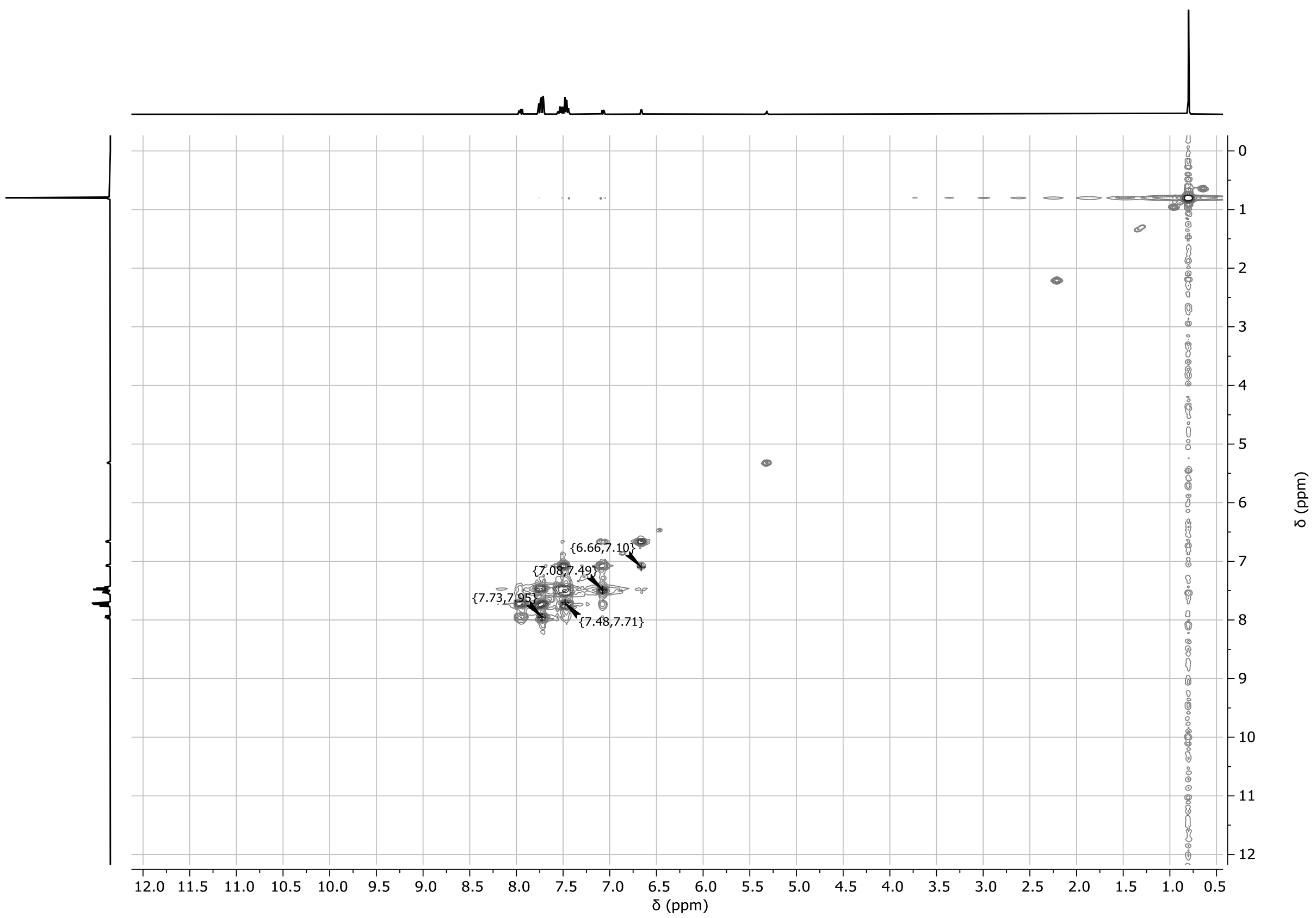

Figure S-174. HH-COSY-NMR spectrum (400 MHz, DCM-d_2) of **Pd(L$_{tBu}$)PPh$_3$**.

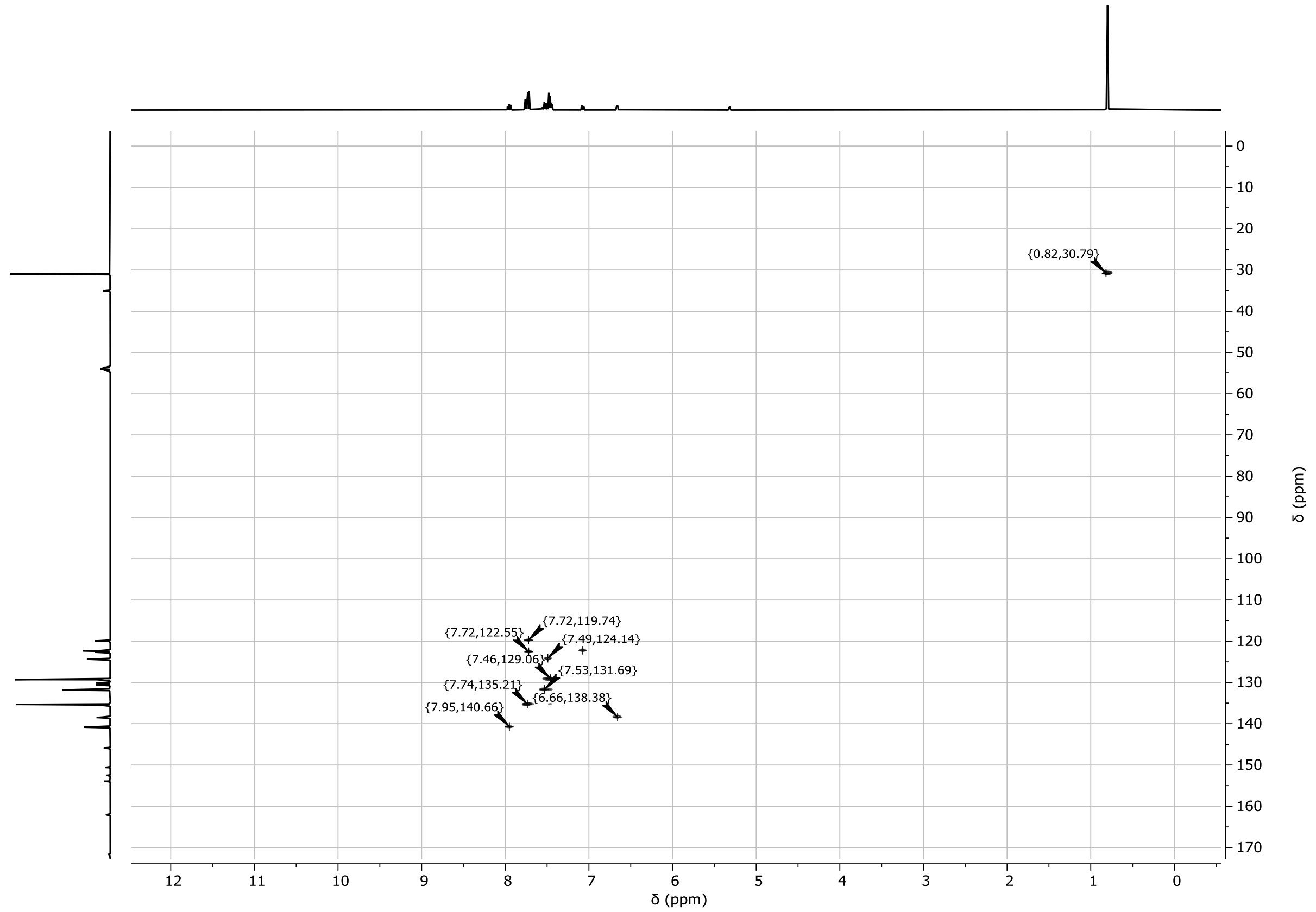

Figure S-175. HC-HSQC-NMR spectrum (101 MHz (^{13}C), 400 MHz (^{1}H), DCM-d_2) of **Pd(L$_{tBu}$)PPh$_3$**.

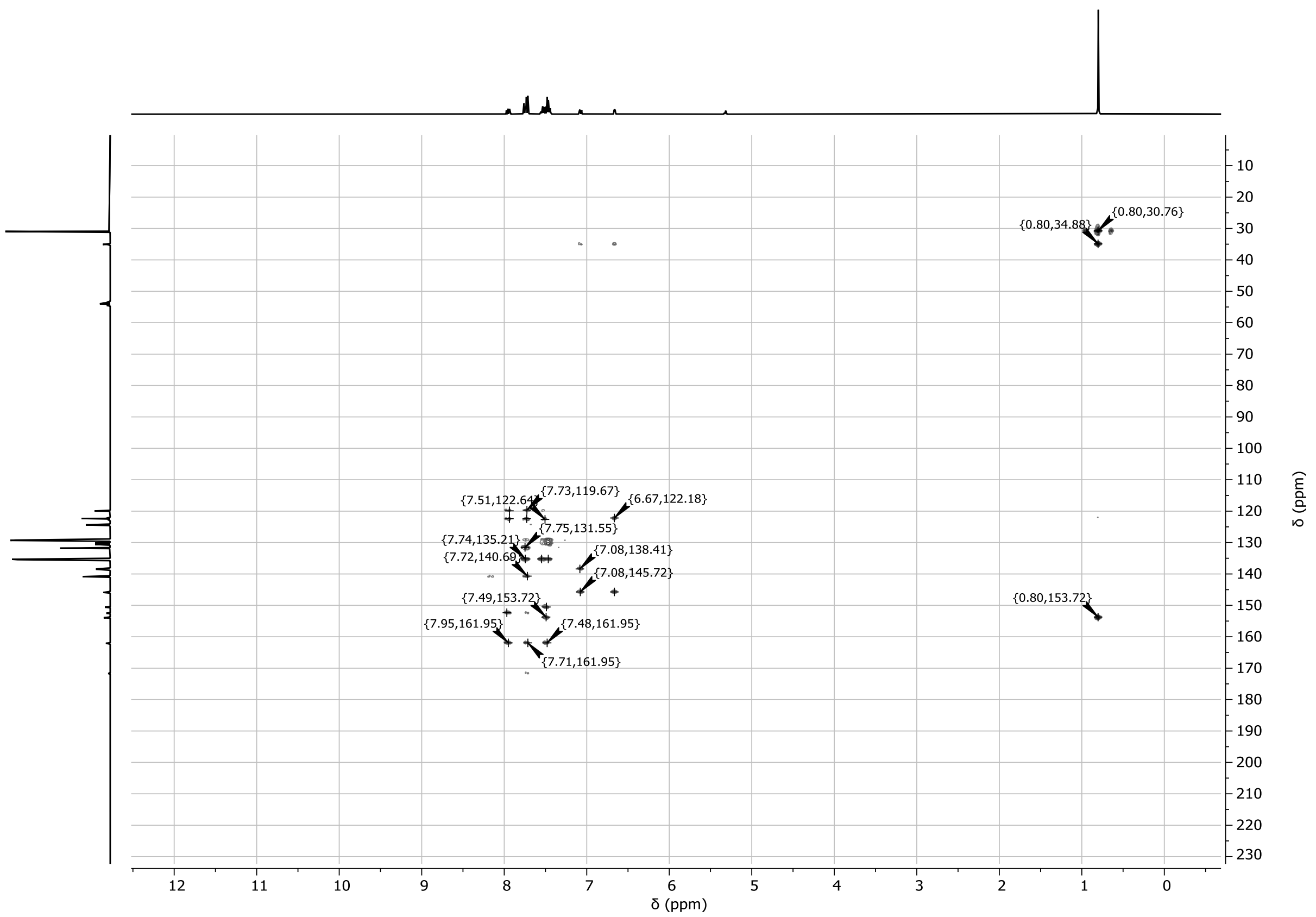

Figure S-176. HC-HMBC-NMR spectrum (101 MHz (^{13}C), 400 MHz (^{1}H), DCM-d_2) of **Pd(L$_{tBu}$)PPh$_3$**.

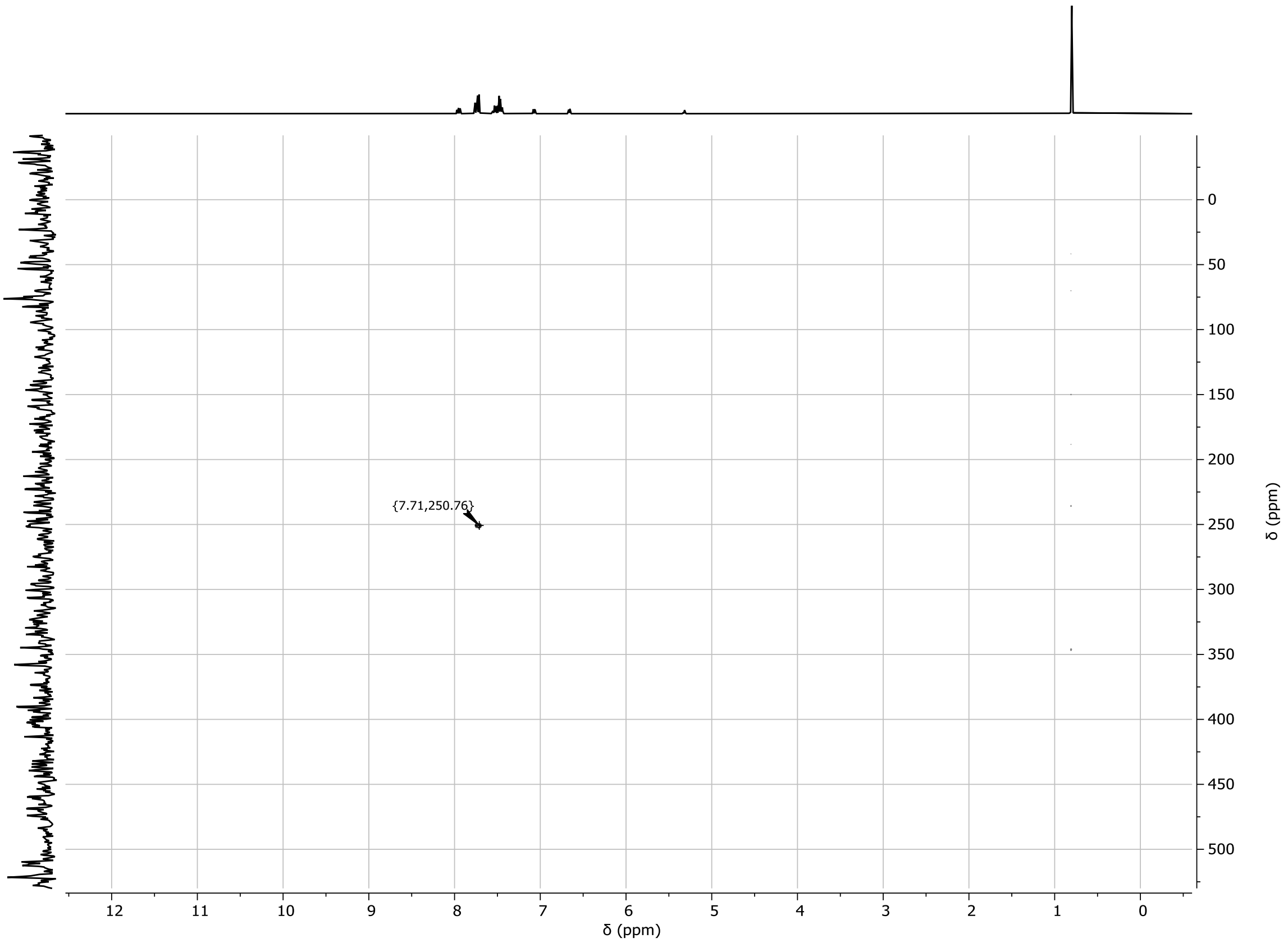

Figure S-177. HN-HMBC-NMR spectrum (41 MHz (^{15}C), 400 MHz (^{1}H), DCM-d_2) of **Pd(L$_{tBu}$)PPh$_3$**.

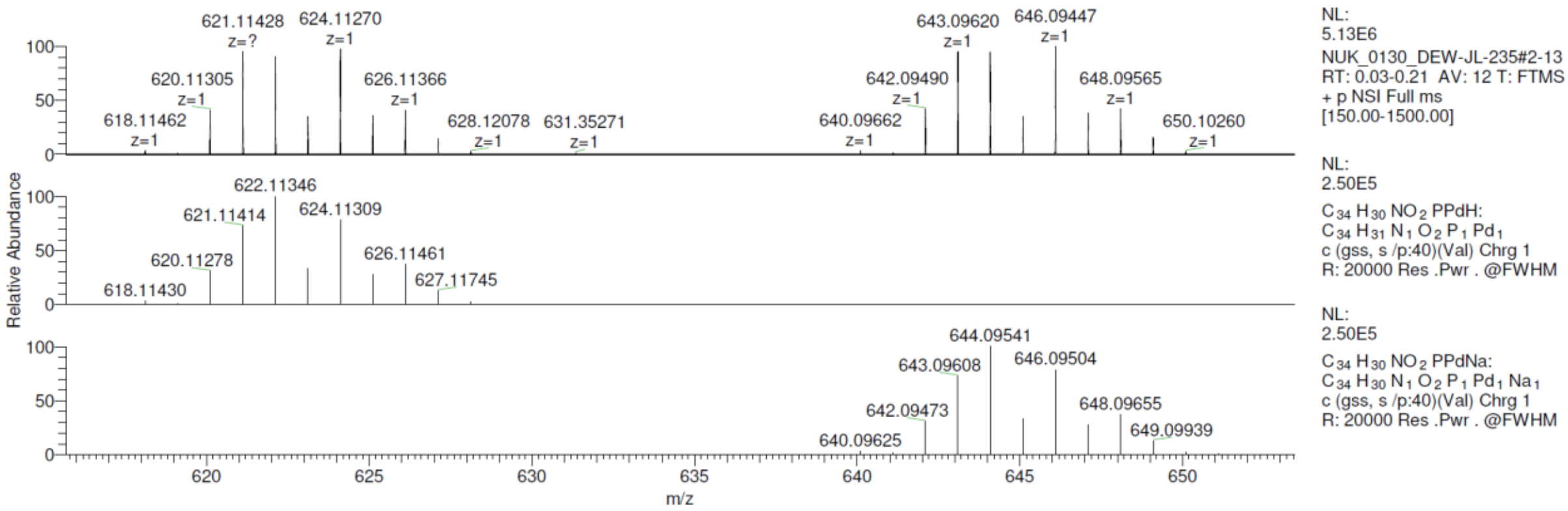

Figure S-178. Mass spectrum of **Pd(L$_{tBu}$)PPh$_3$** (MeOH). Additional simulation of the [**Pd(L$_{tBu}$)PPh$_3$**+H]$^+$ and [**Pd(L$_{tBu}$)PPh$_3$**+Na]$^+$ adduct.

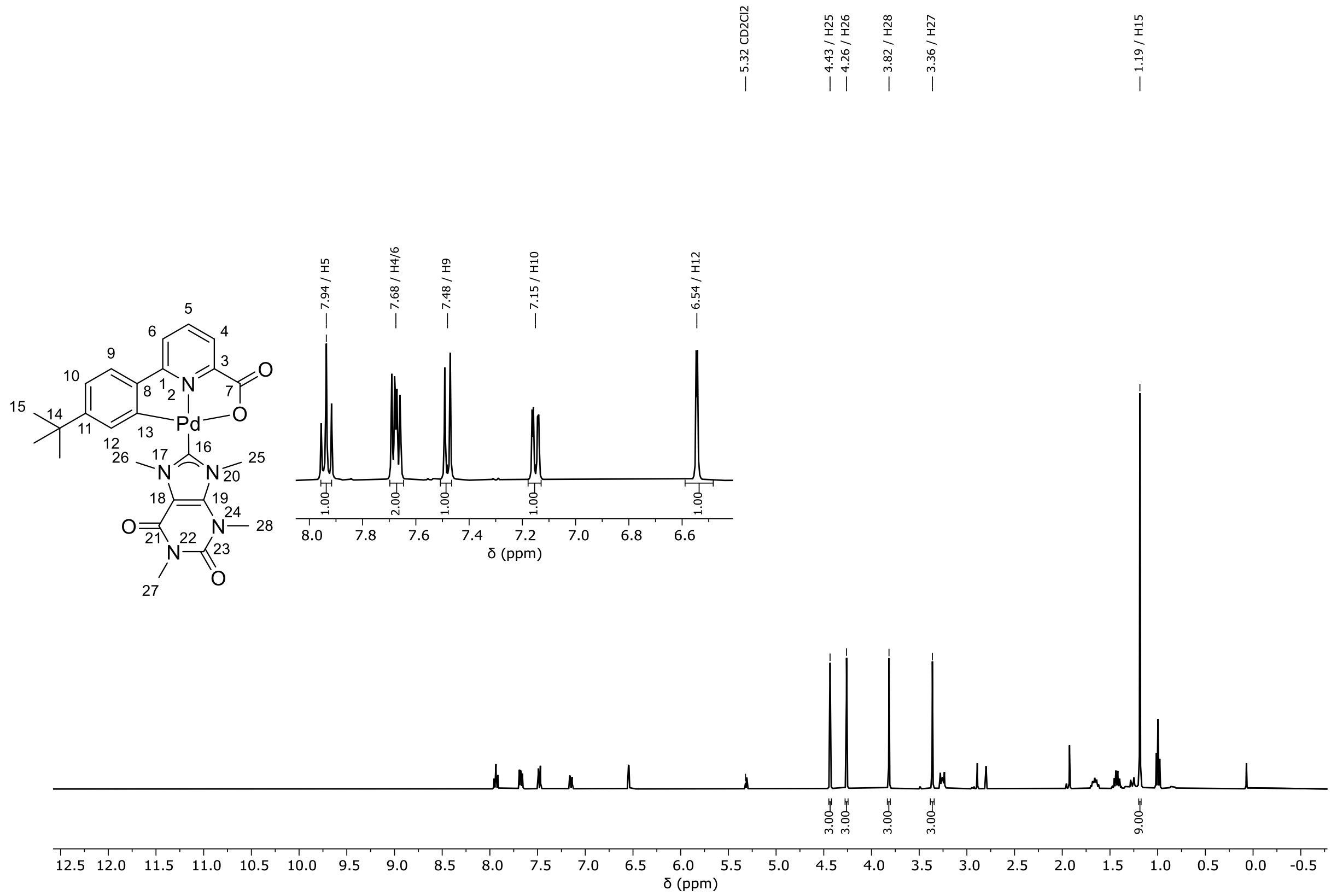

Figure S-179. ^{1}H-NMR spectrum (500 MHz, DCM-d_2) of **Pd(L_{tBu})NHC**.

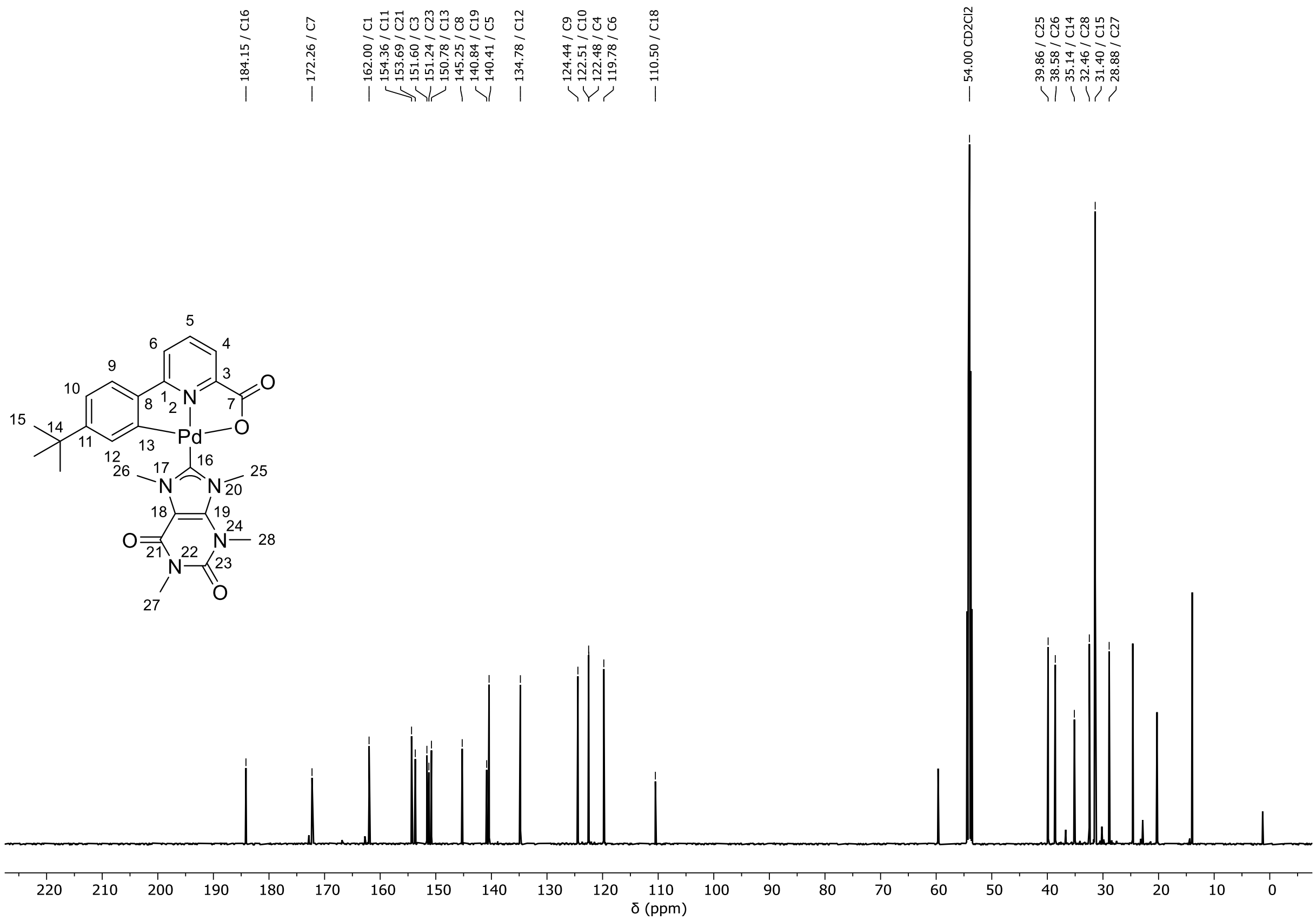

Figure S-180. $^{13}C\{^{1}H\}$-NMR spectrum (126 MHz, DCM-d_2) of **Pd(L_{tBu})NHC**.

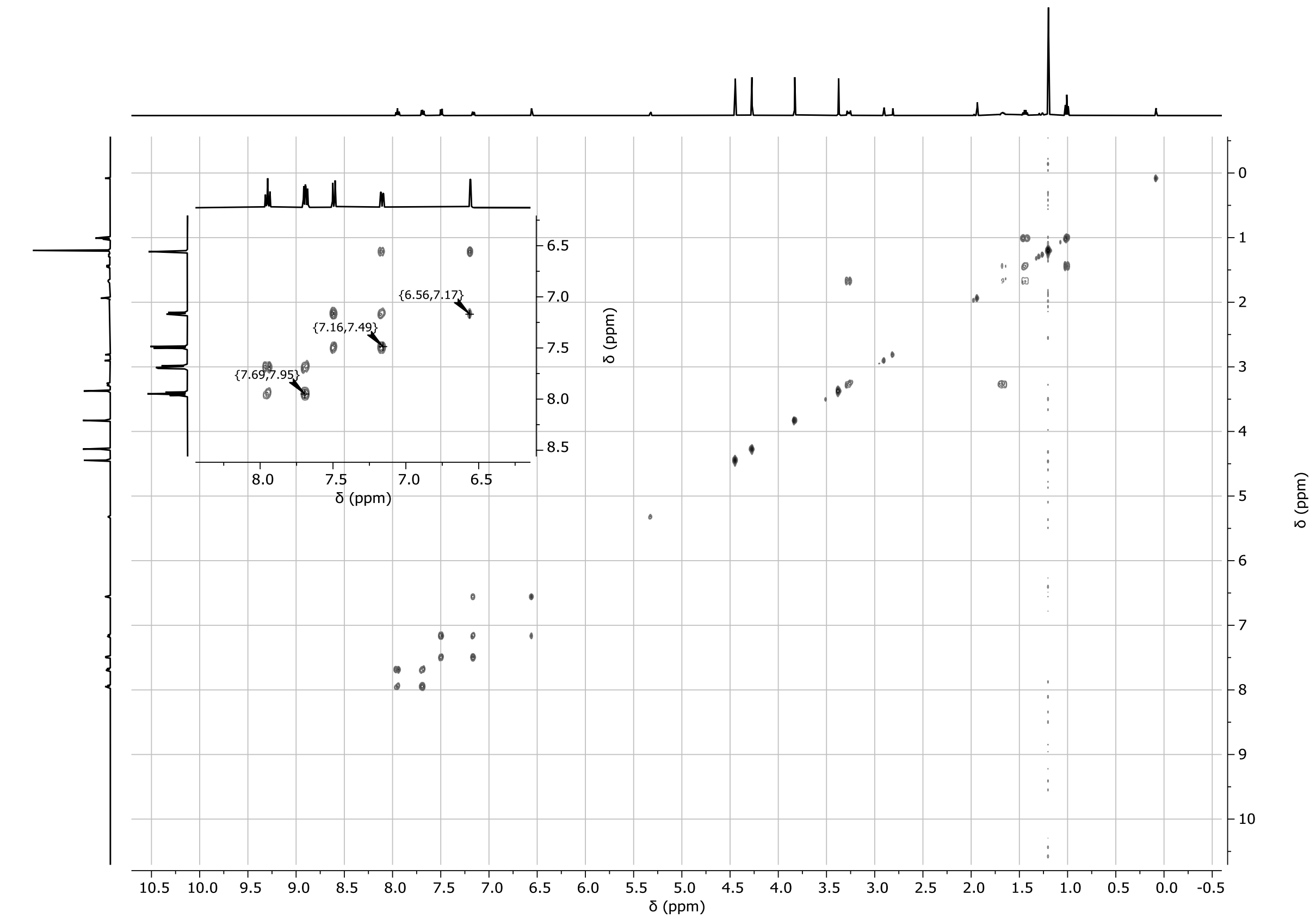

Figure S-181. HH-COSY-NMR spectrum (500 MHz, DCM-d_2) of **Pd(L_{tBu})NHC**.

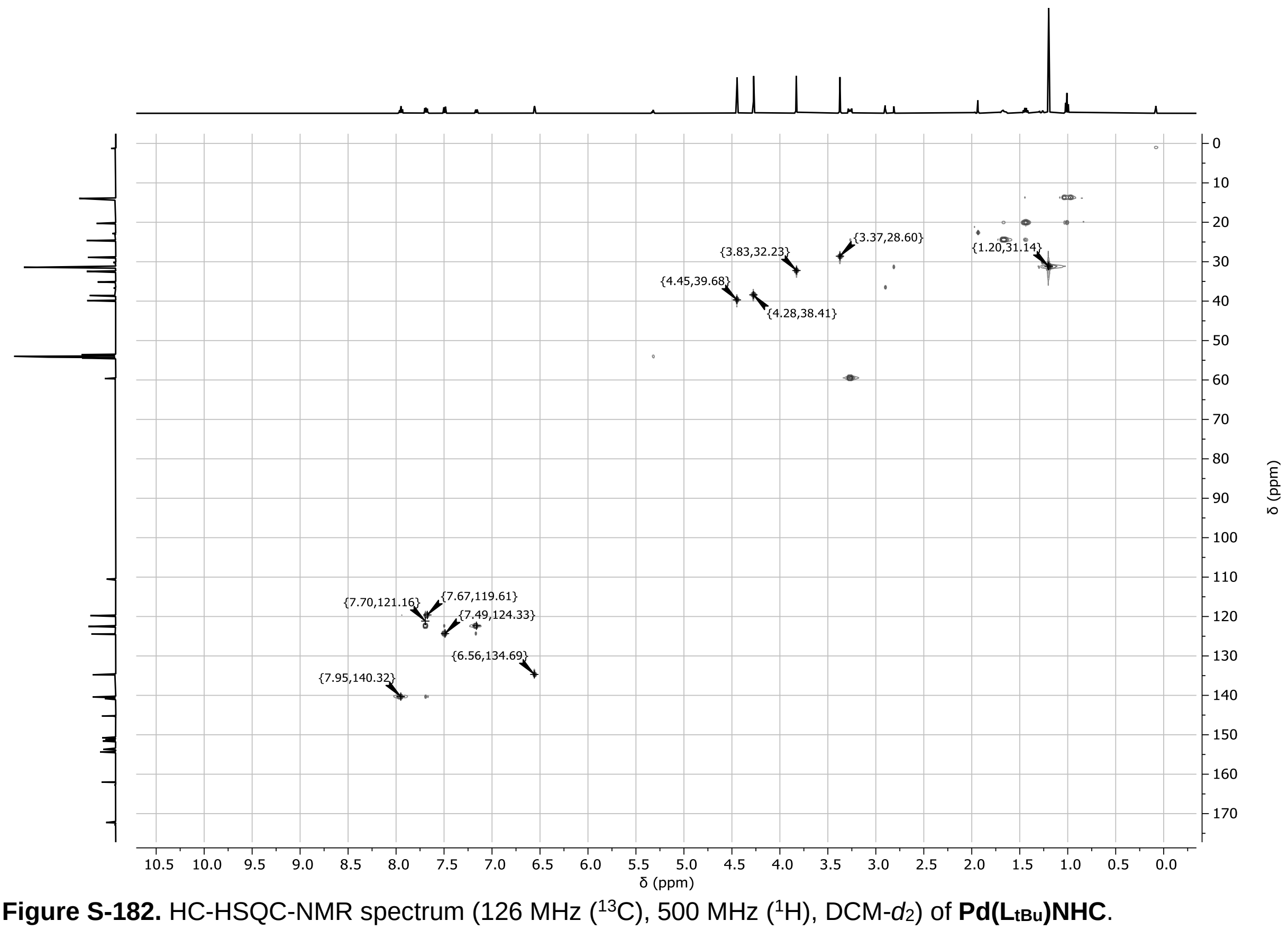

Figure S-182. HC-HSQC-NMR spectrum (126 MHz (^{13}C), 500 MHz (^{1}H), DCM-d_2) of **Pd(L_{tBu})NHC**.

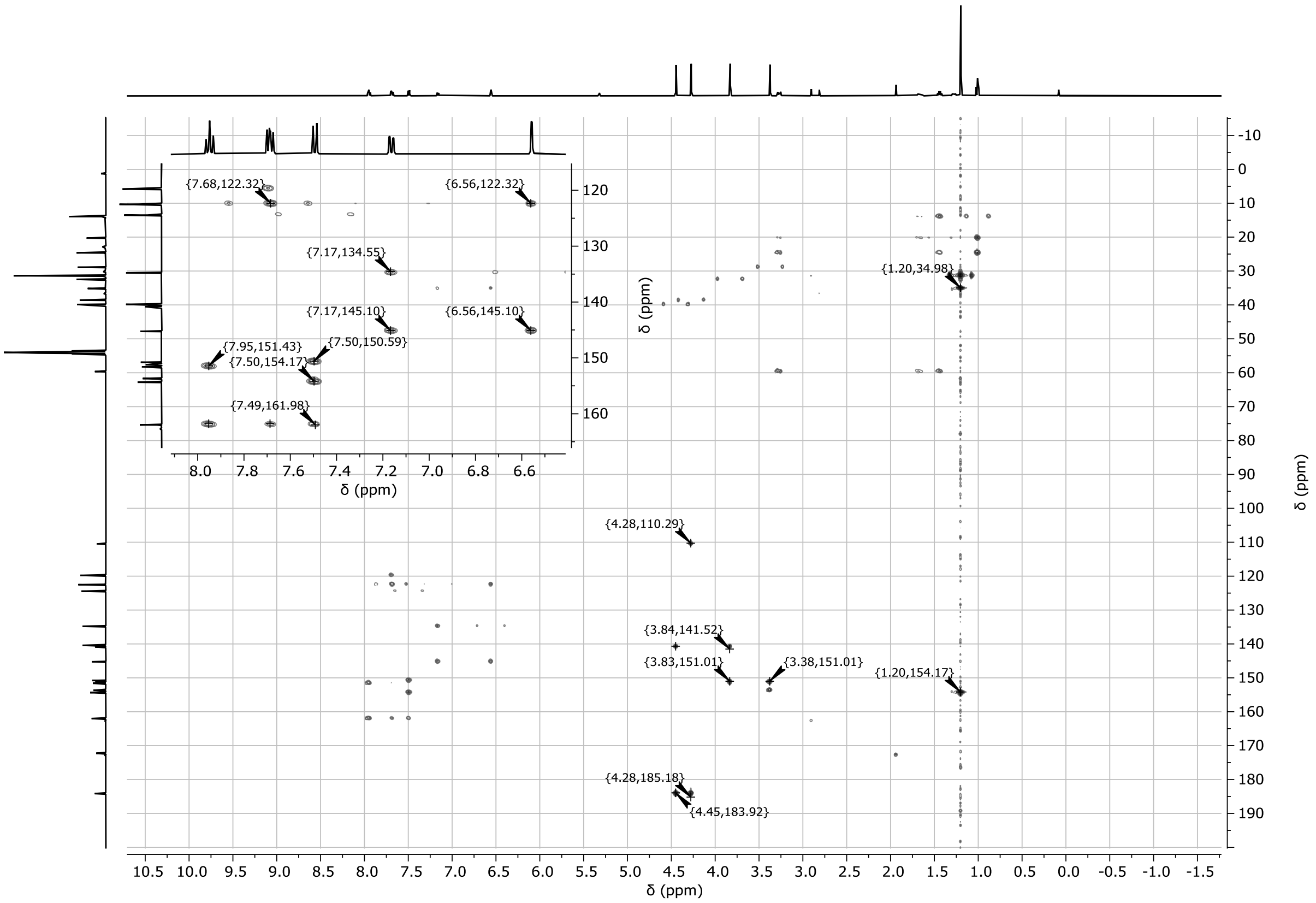

Figure S-183. HC-HMBC-NMR spectrum (126 MHz (^{13}C), 500 MHz (^{1}H), DCM-d_2) of **Pd(L_{tBu})NHC**.

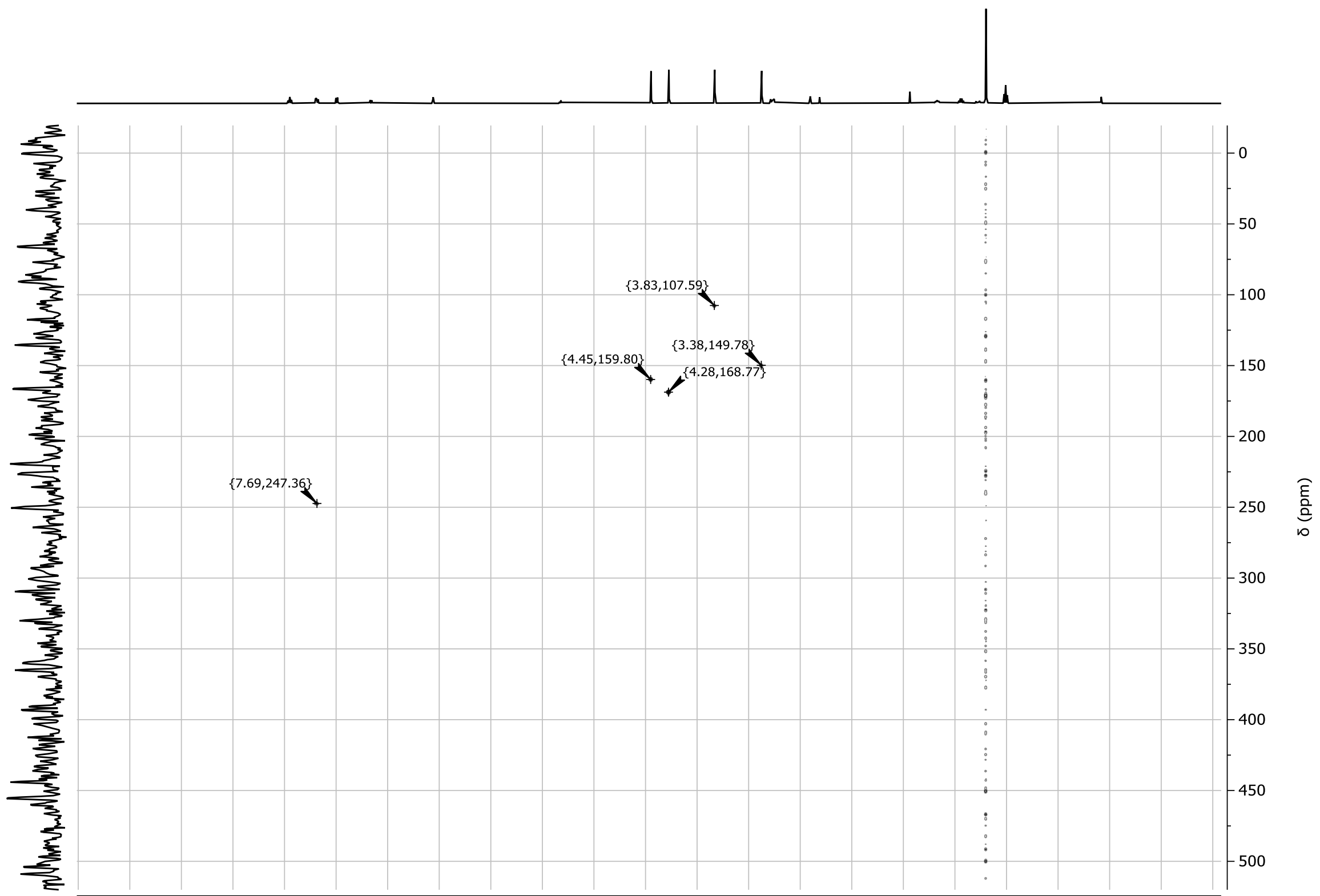

Figure S-184. HN-HMBC-NMR spectrum (51 MHz (^{15}N), 500 MHz (^{1}H), DCM-d_2) of **Pd(L_{tBu})NHC**.

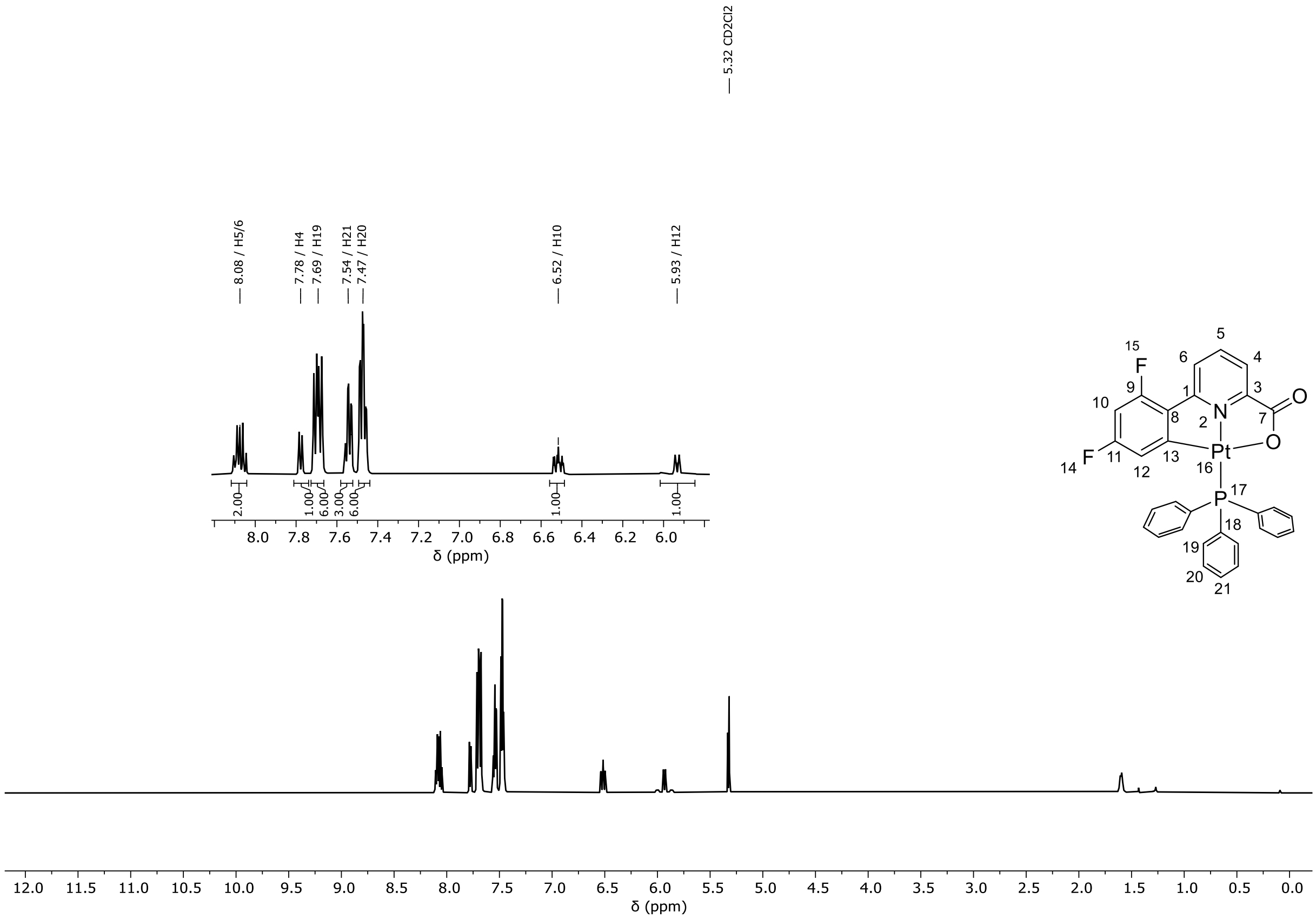

Figure S-185. ^{1}H-NMR spectrum (500 MHz, DCM-d_2) of **Pt(L$_{DiF}$)PPh$_3$**.

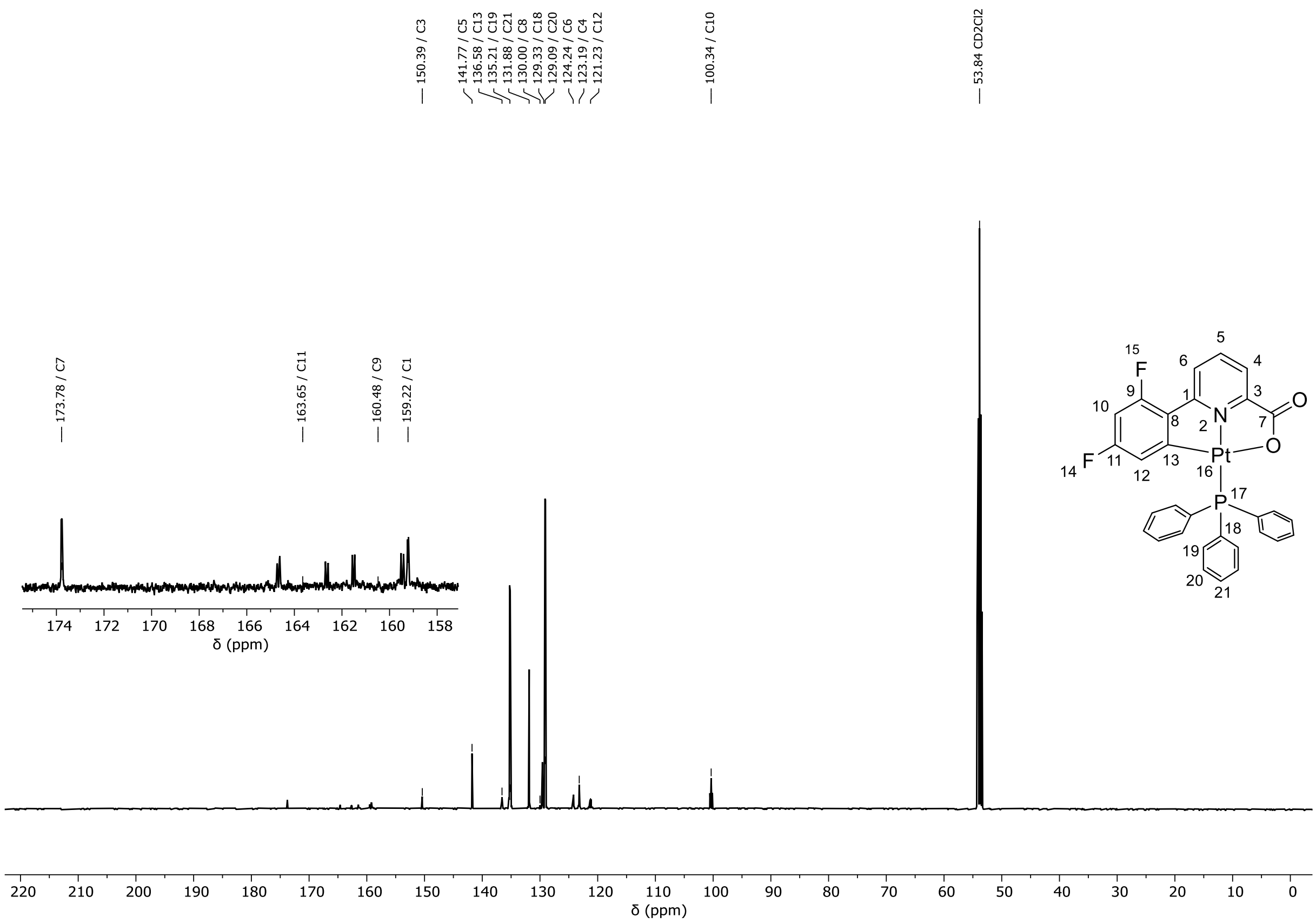

Figure S-186. ^{13}C{^{1}H}-NMR spectrum (126 MHz, DCM-d_2) of **Pt(L$_{DiF}$)PPh$_3$**.

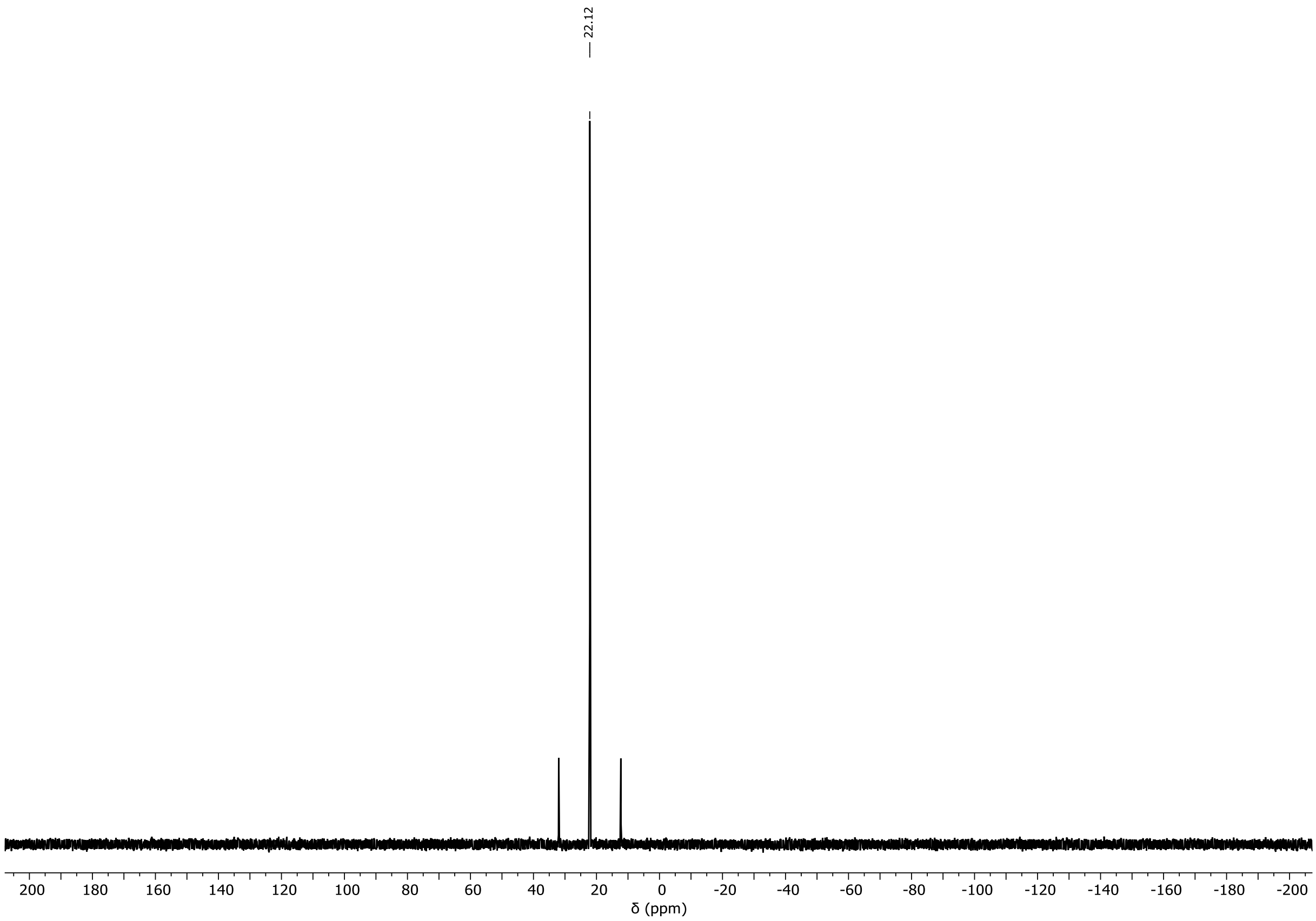

Figure S-187. $^{19}F\{^{1}H\}$-NMR spectrum (471 MHz, DCM-d_2) of **Pt(L$_{DiF}$)PPh$_3$**.

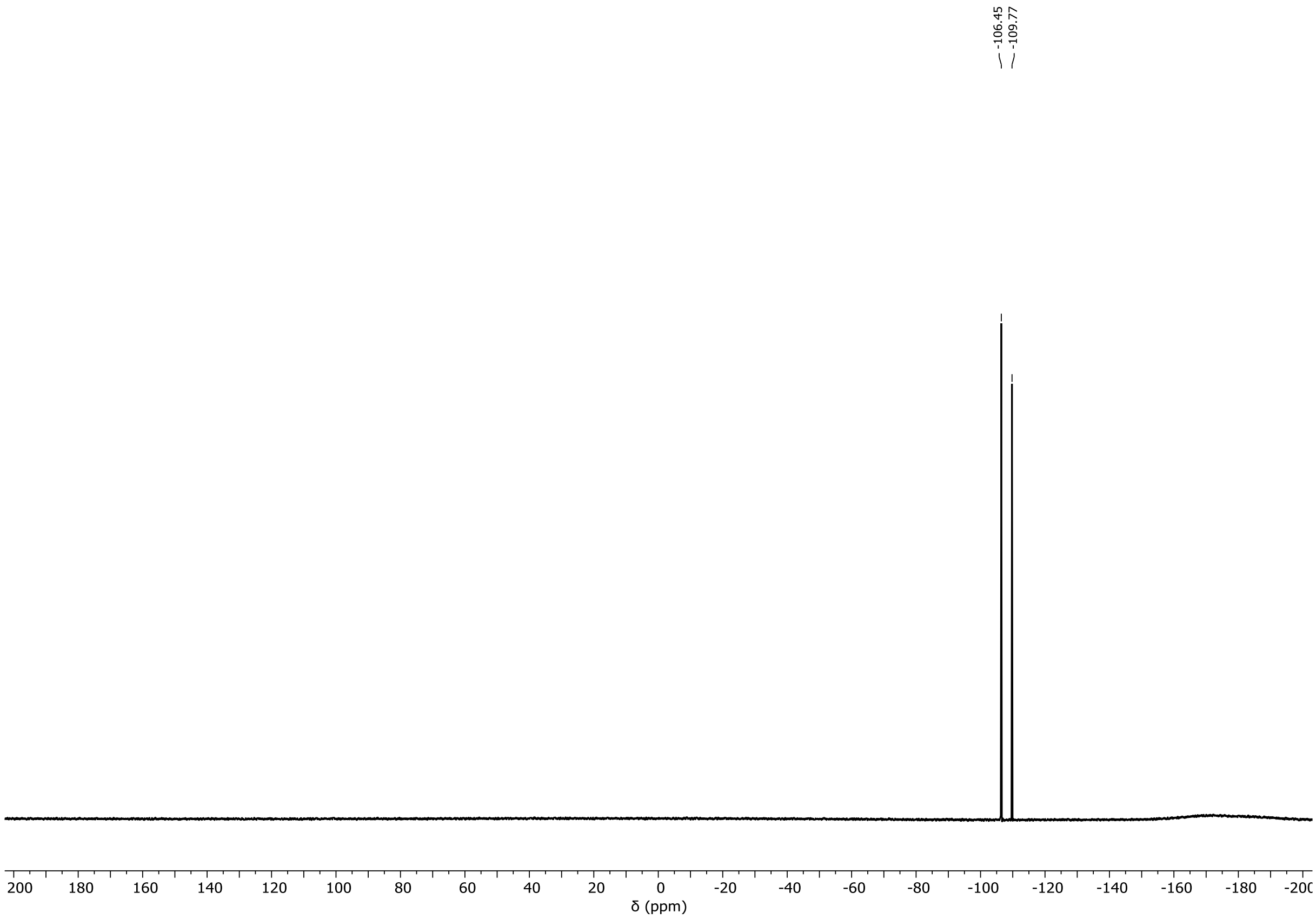

Figure S-188. $^{31}P\{^{1}H\}$-NMR spectrum (202 MHz, DCM-d_2) of **Pt(L$_{DiF}$)PPh$_3$**.

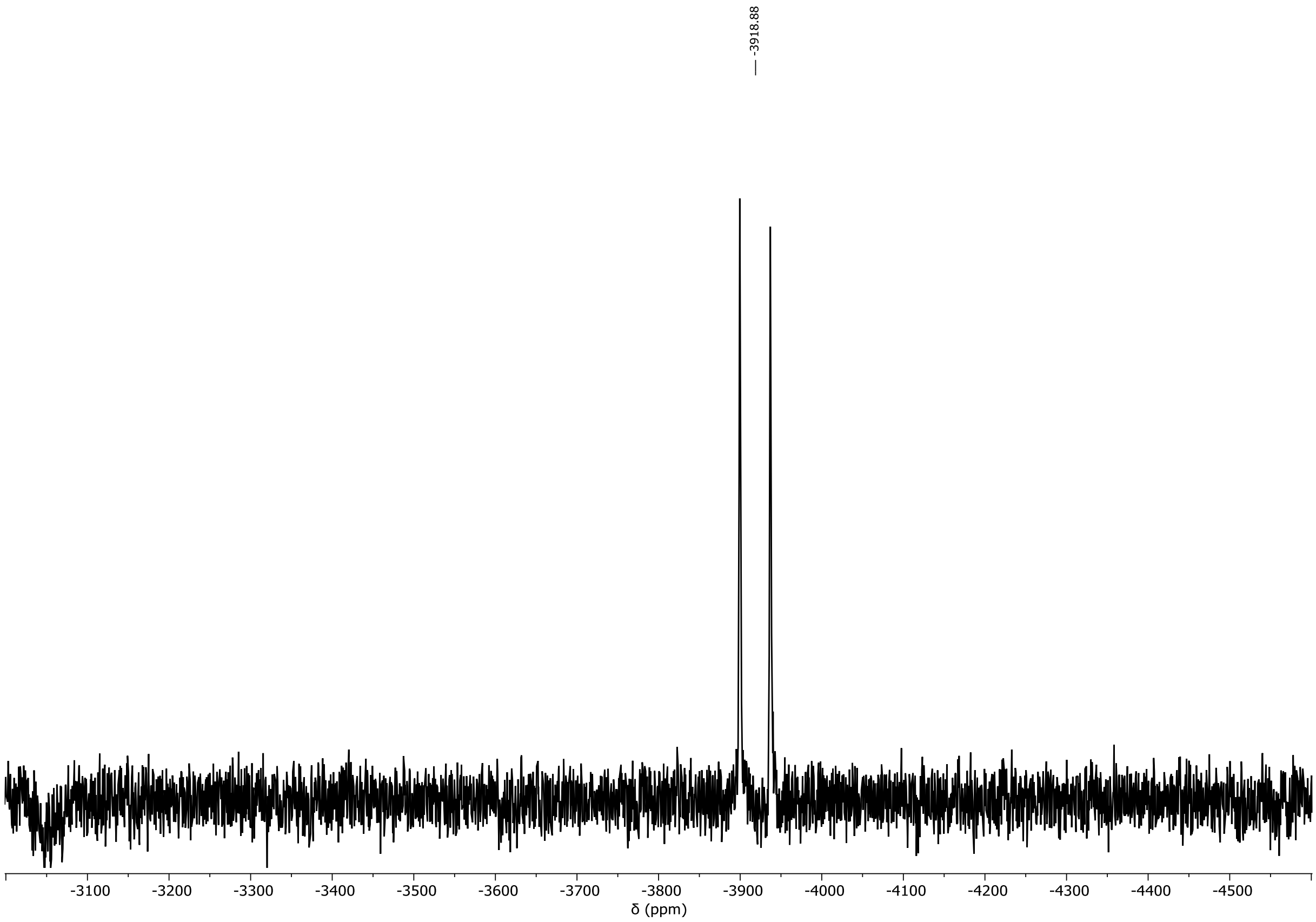

Figure S-189. ^{195}Pt-NMR spectrum (107 MHz, DCM-d_2) of **Pt(L$_{DiF}$)PPh$_3$**.

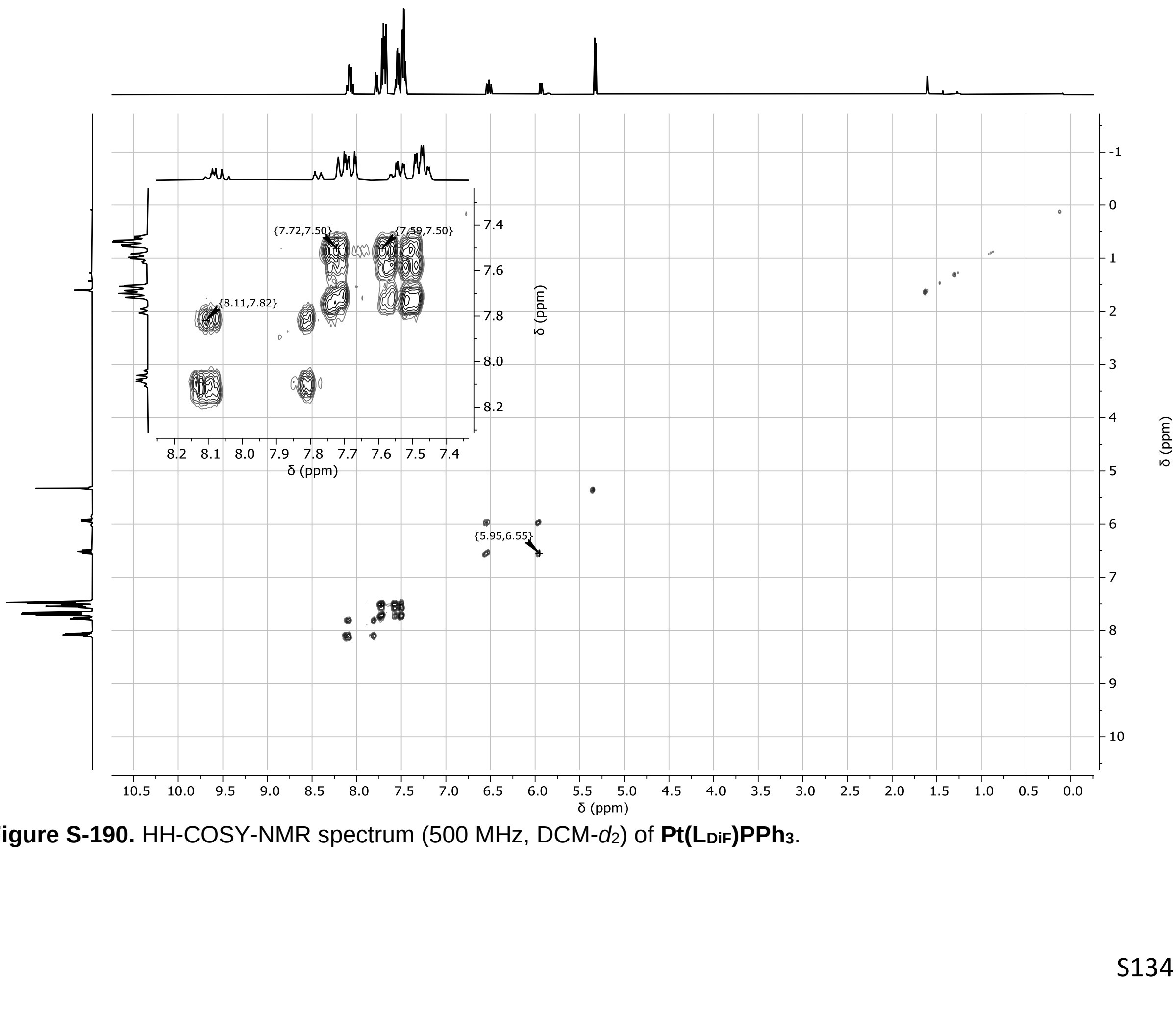

Figure S-190. HH-COSY-NMR spectrum (500 MHz, DCM-d_2) of **Pt(L$_{DiF}$)PPh$_3$**.

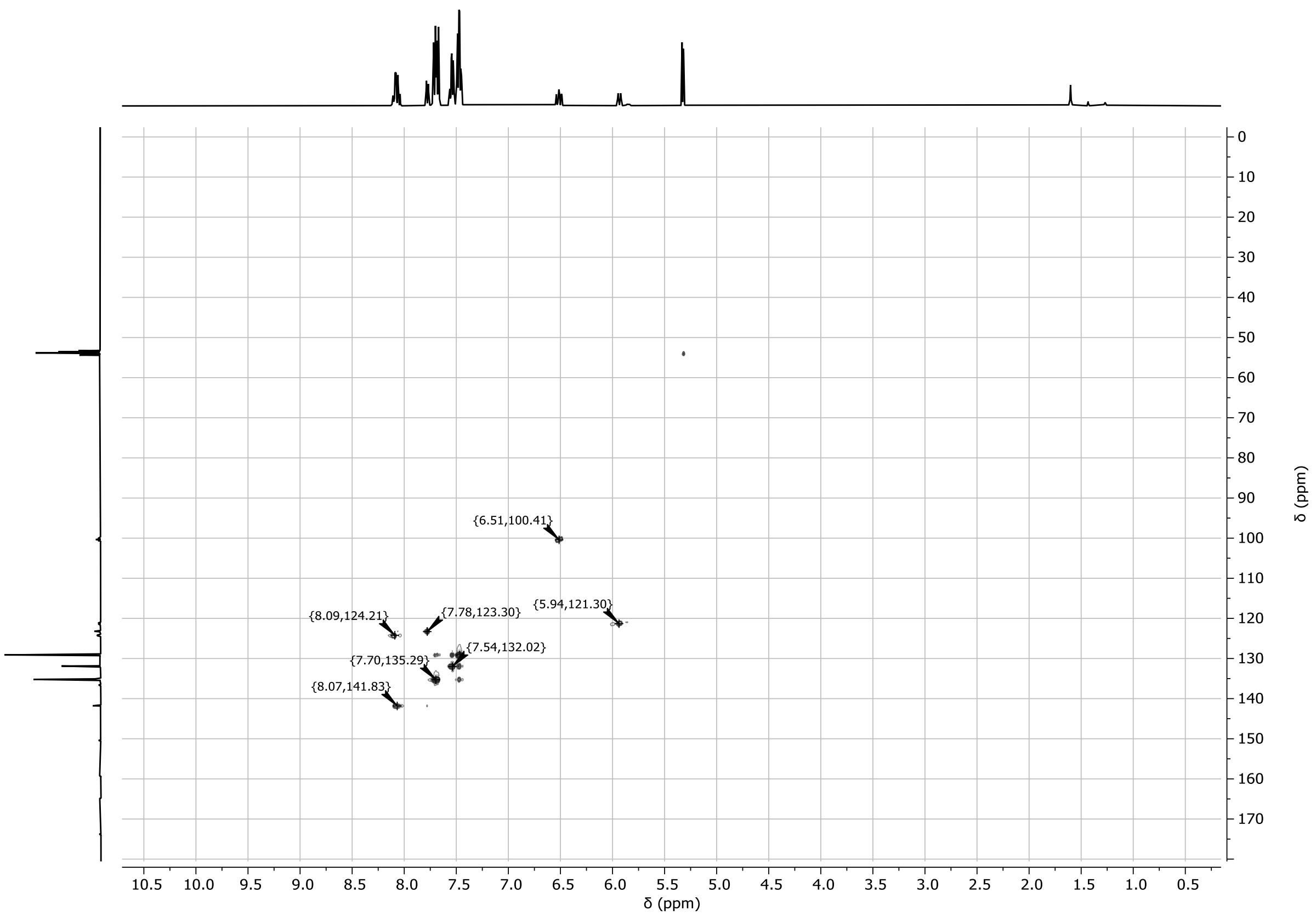

Figure S-191. HC-HSQC-NMR spectrum (126 MHz (^{13}C), 500 MHz (^{1}H), DCM-d_2) of **Pt(L$_{DiF}$)PPh$_3$**.

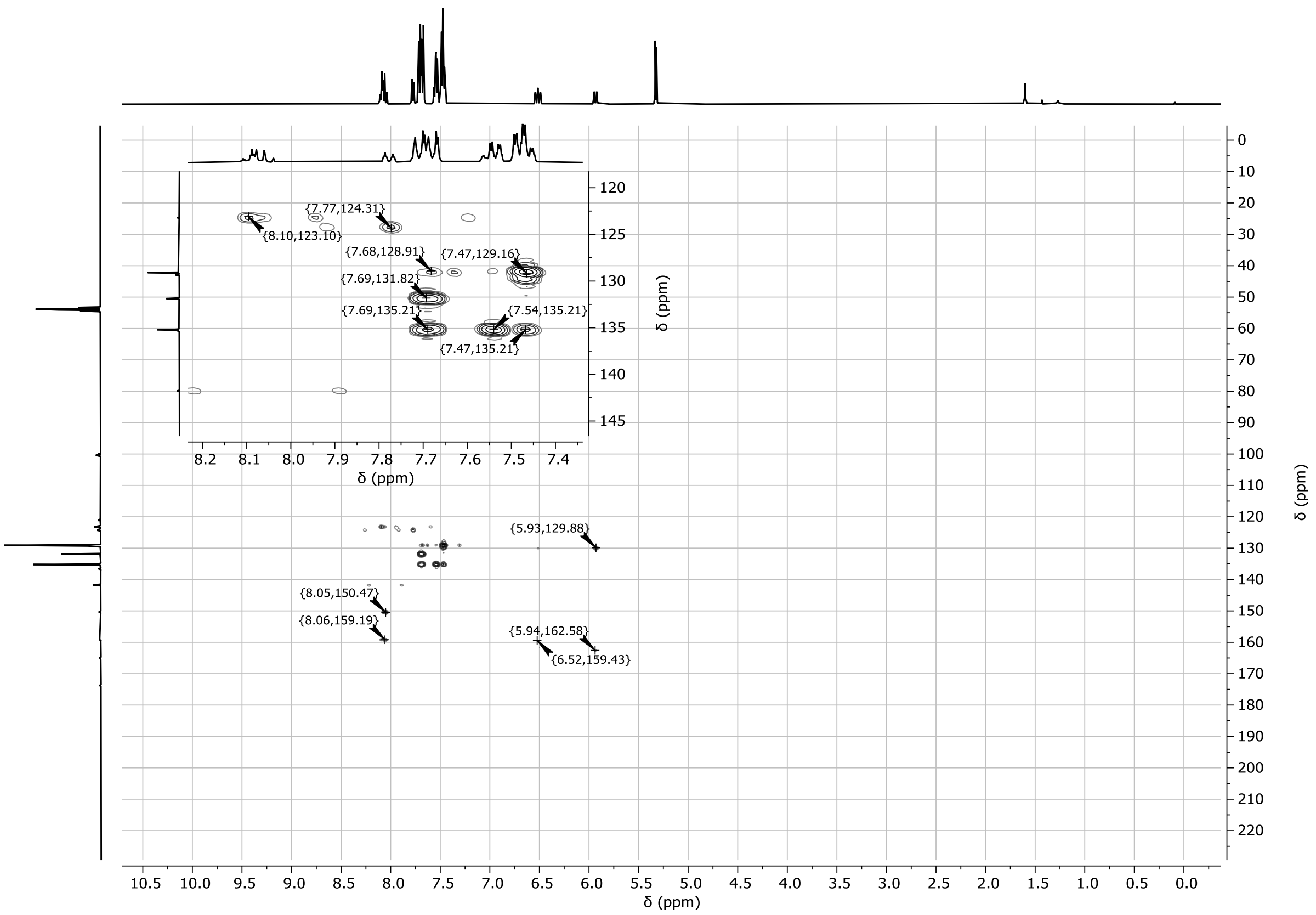

Figure S-192. HC-HMBC-NMR spectrum (126 MHz (^{13}C), 500 MHz (^{1}H), DCM-d_2) of **Pt(L$_{DiF}$)PPh$_3$**.

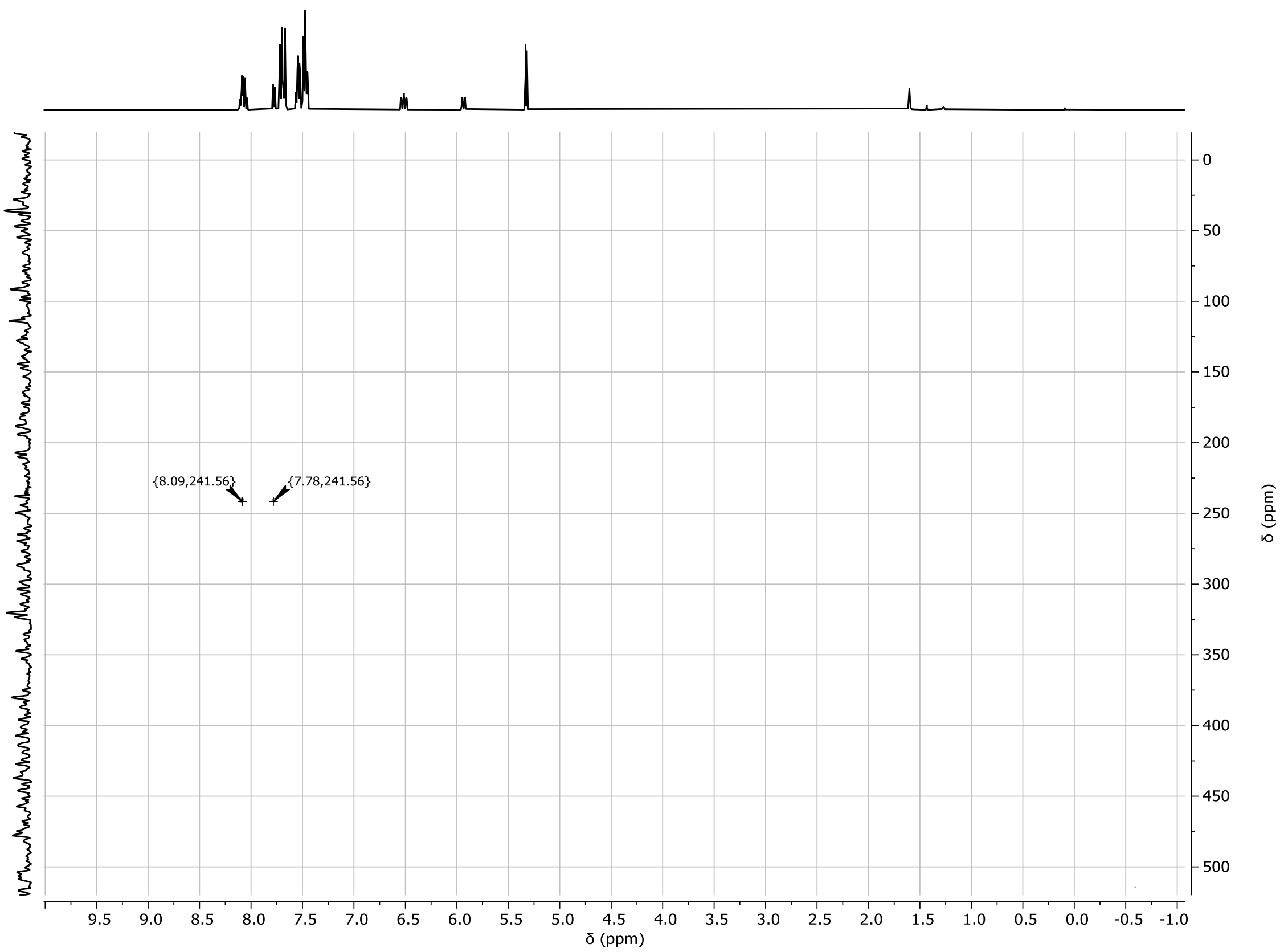

Figure S-193. HN-HMBC-NMR spectrum (51 MHz (^{15}N), 500 MHz (^{1}H), DCM-d_2) of **Pt(L$_{DiF}$)PPh$_3$**.

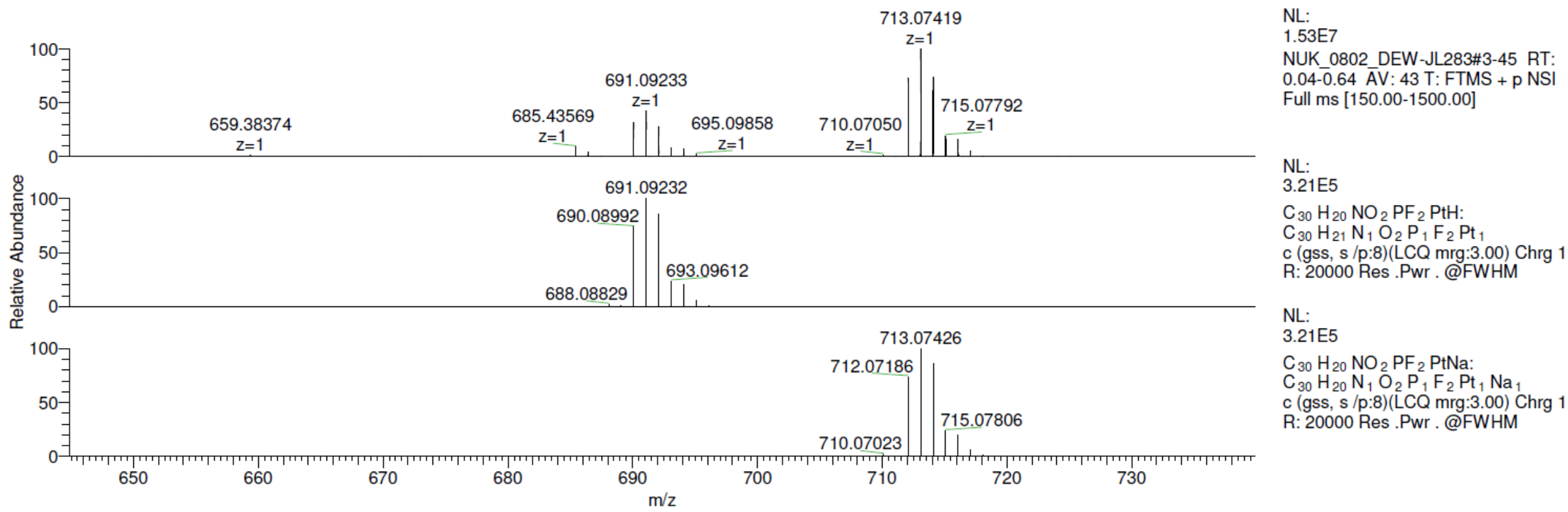

Figure S-194. Mass spectrum of **Pt(L$_{DiF}$)PPh$_3$** (MeOH). Additional simulation of the [**Pt(L$_{DiF}$)PPh$_3$**+H]$^+$ and [**Pt(L$_{DiF}$)PPh$_3$**+Na]$^+$ adduct.

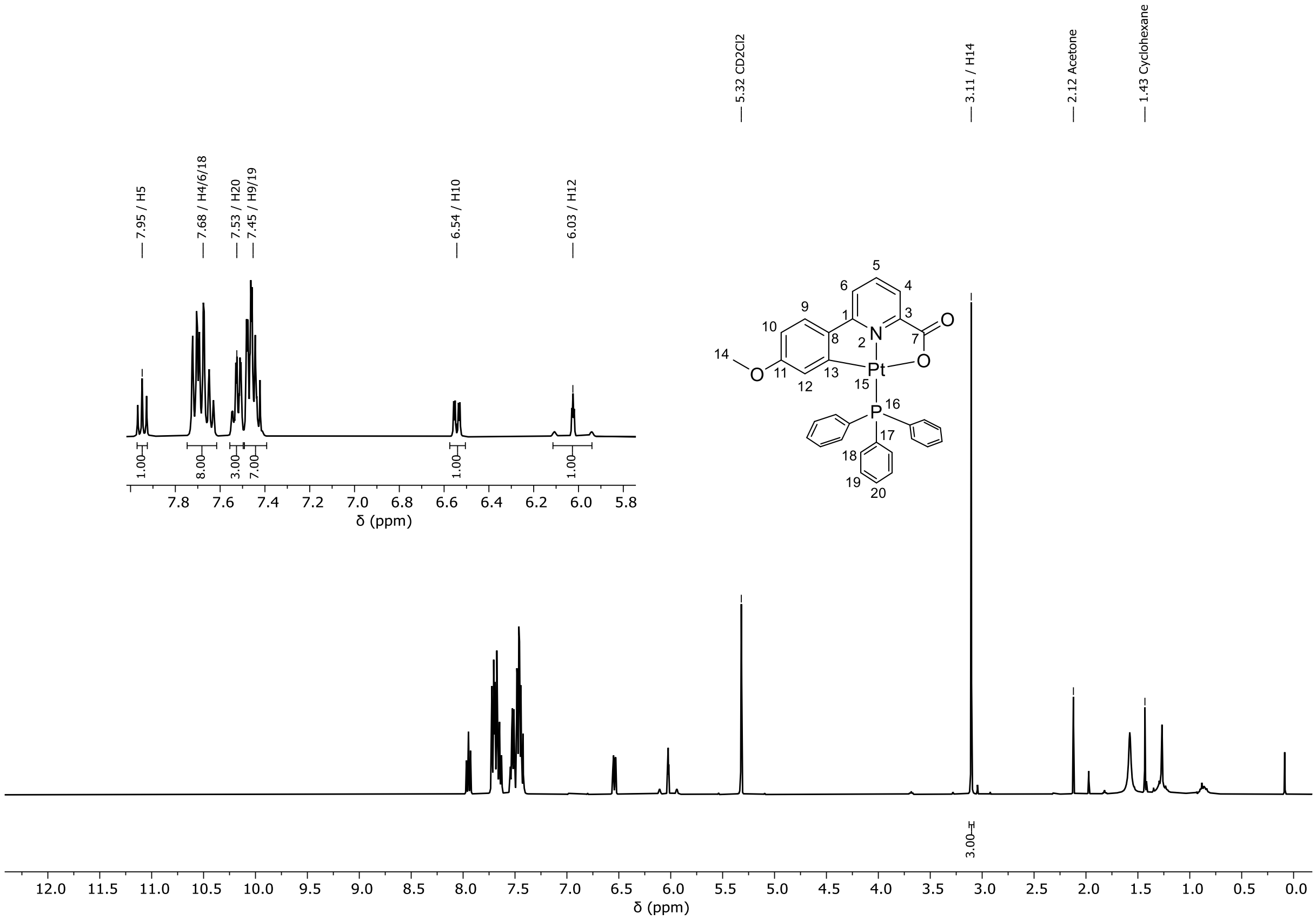

Figure S-195. ^{1}H-NMR spectrum (400 MHz, DCM-d_2) of **Pt(L$_{mOMe}$)PPh$_3$**.

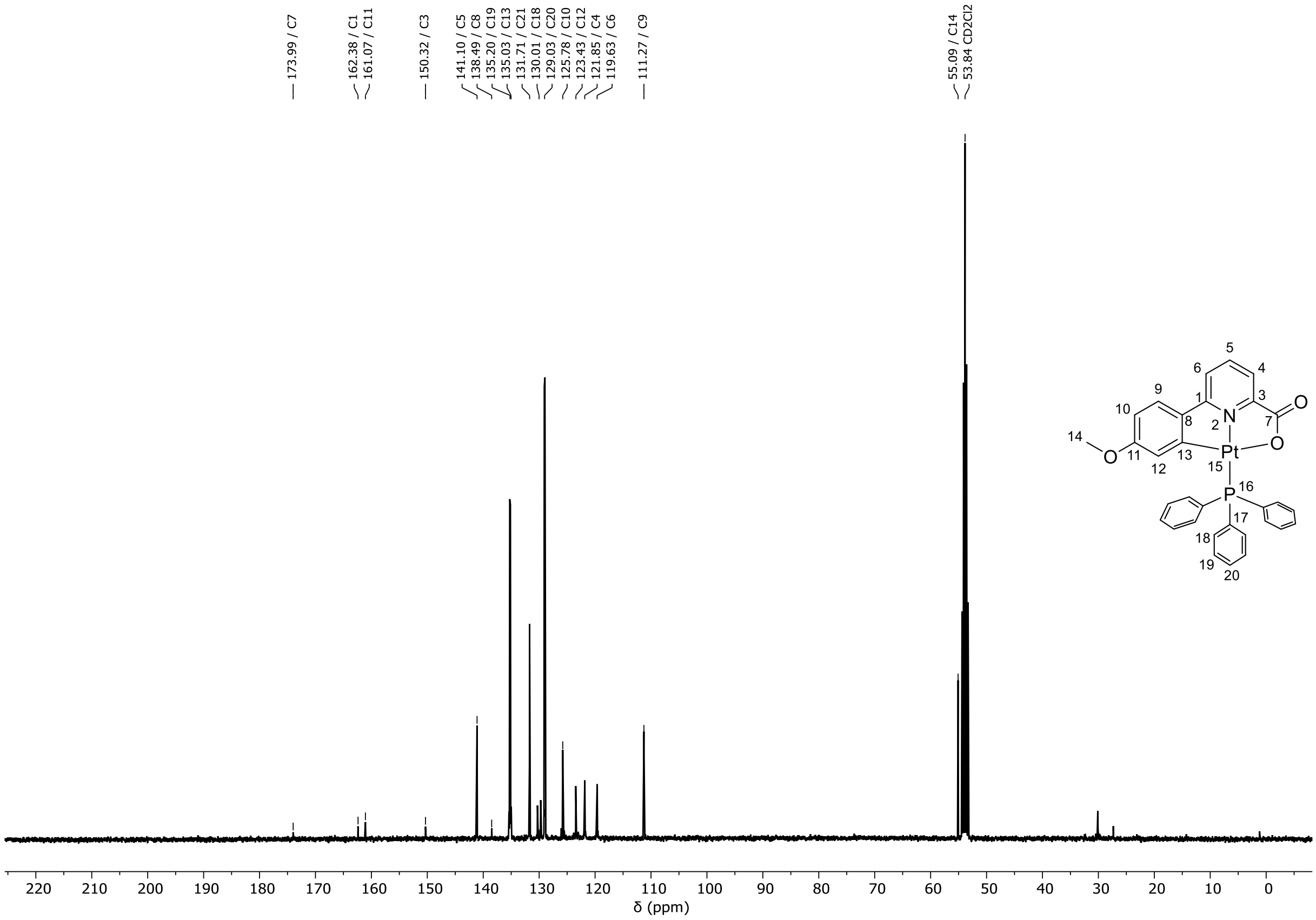

Figure S-196. ^{13}C{^{1}H}-NMR spectrum (101 MHz, DCM-d_2) of **Pt(L$_{mOMe}$)PPh$_3$**.

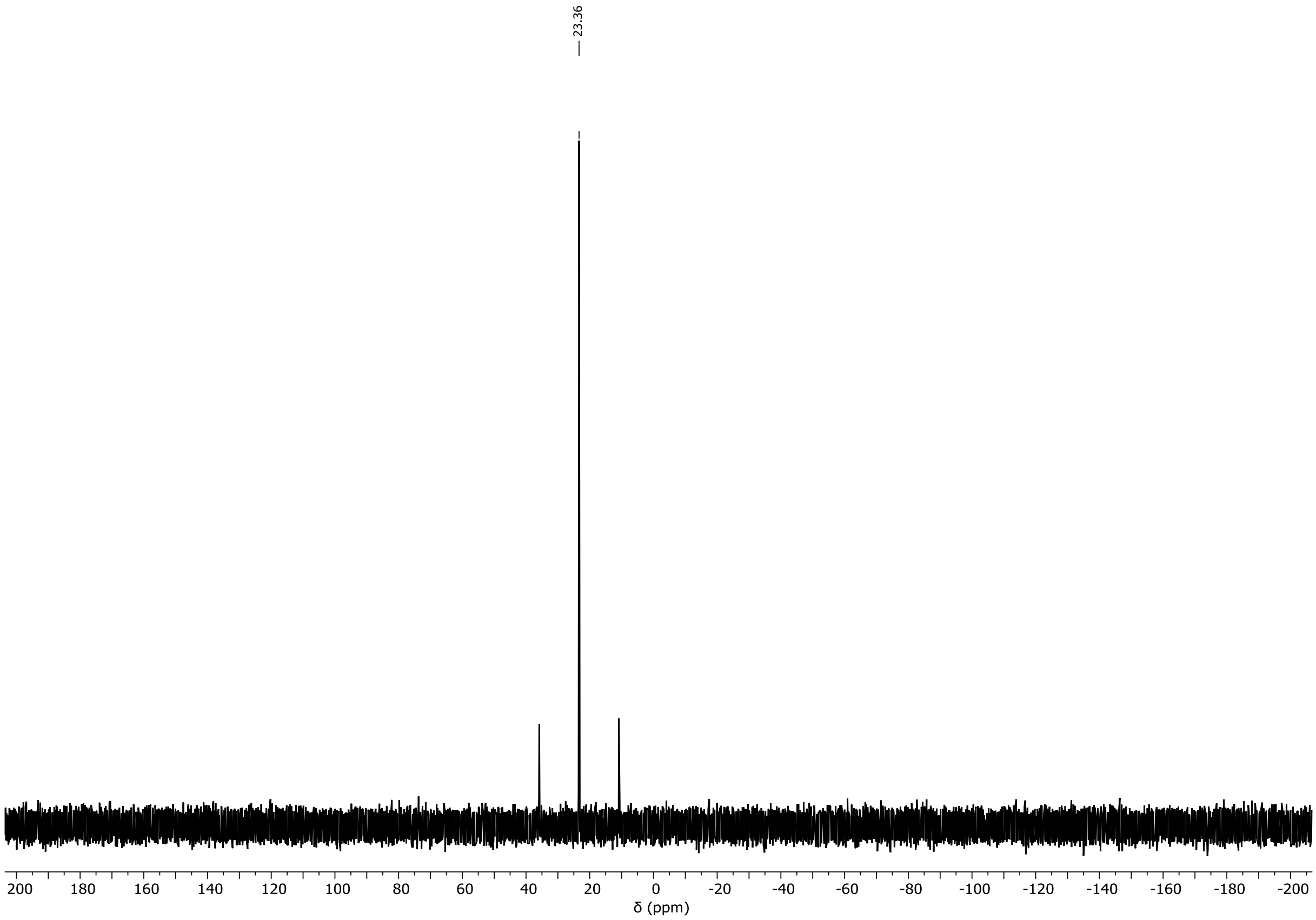

Figure S-197. $^{31}P\{^{1}H\}$-NMR spectrum (162 MHz, DCM-d_2) of **Pt(L$_{mOMe}$)PPh$_3$**.

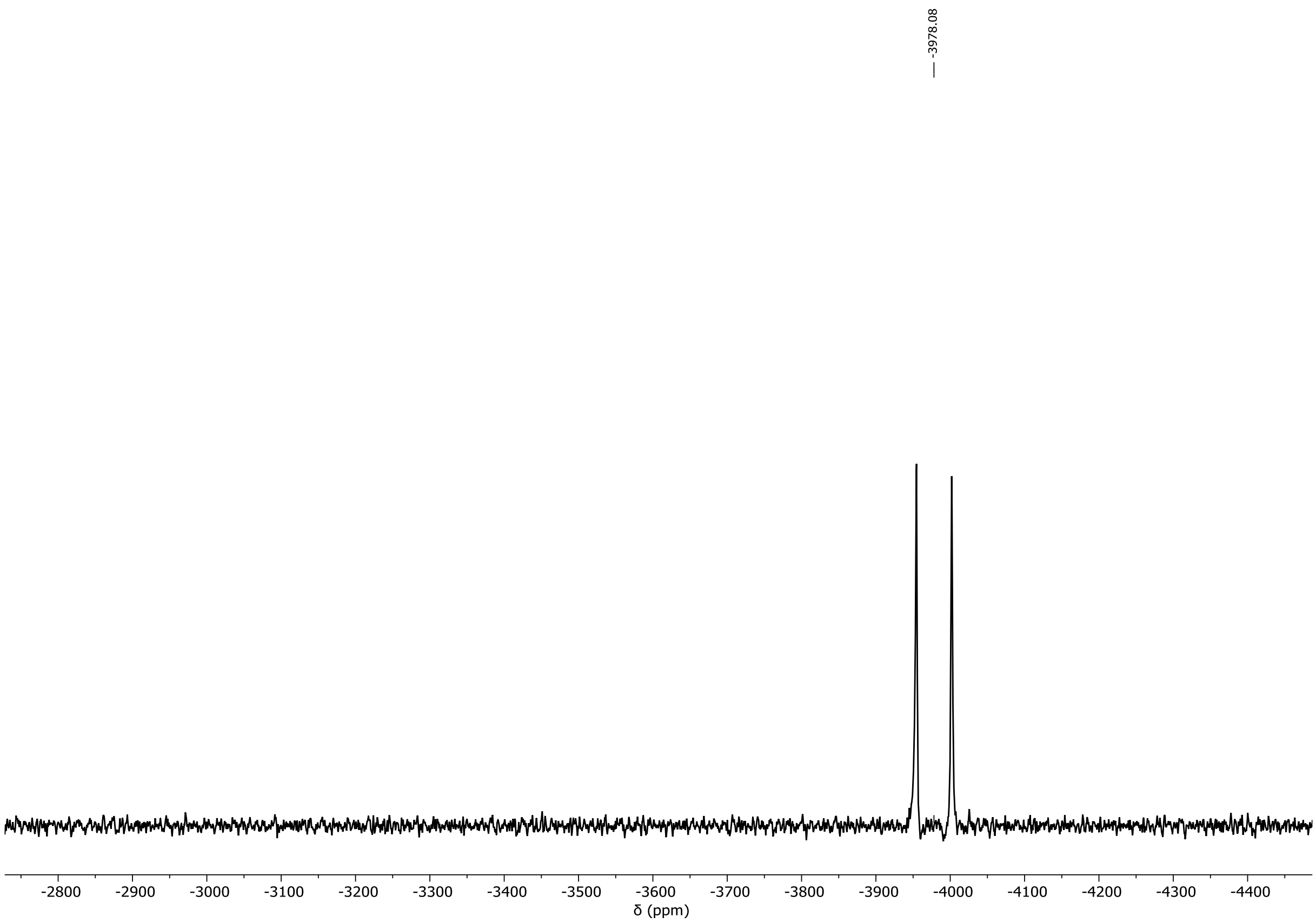

Figure S-198. ^{195}Pt-NMR spectrum (86 MHz, DCM-d_2) of **Pt(L$_{mOMe}$)PPh$_3$**.

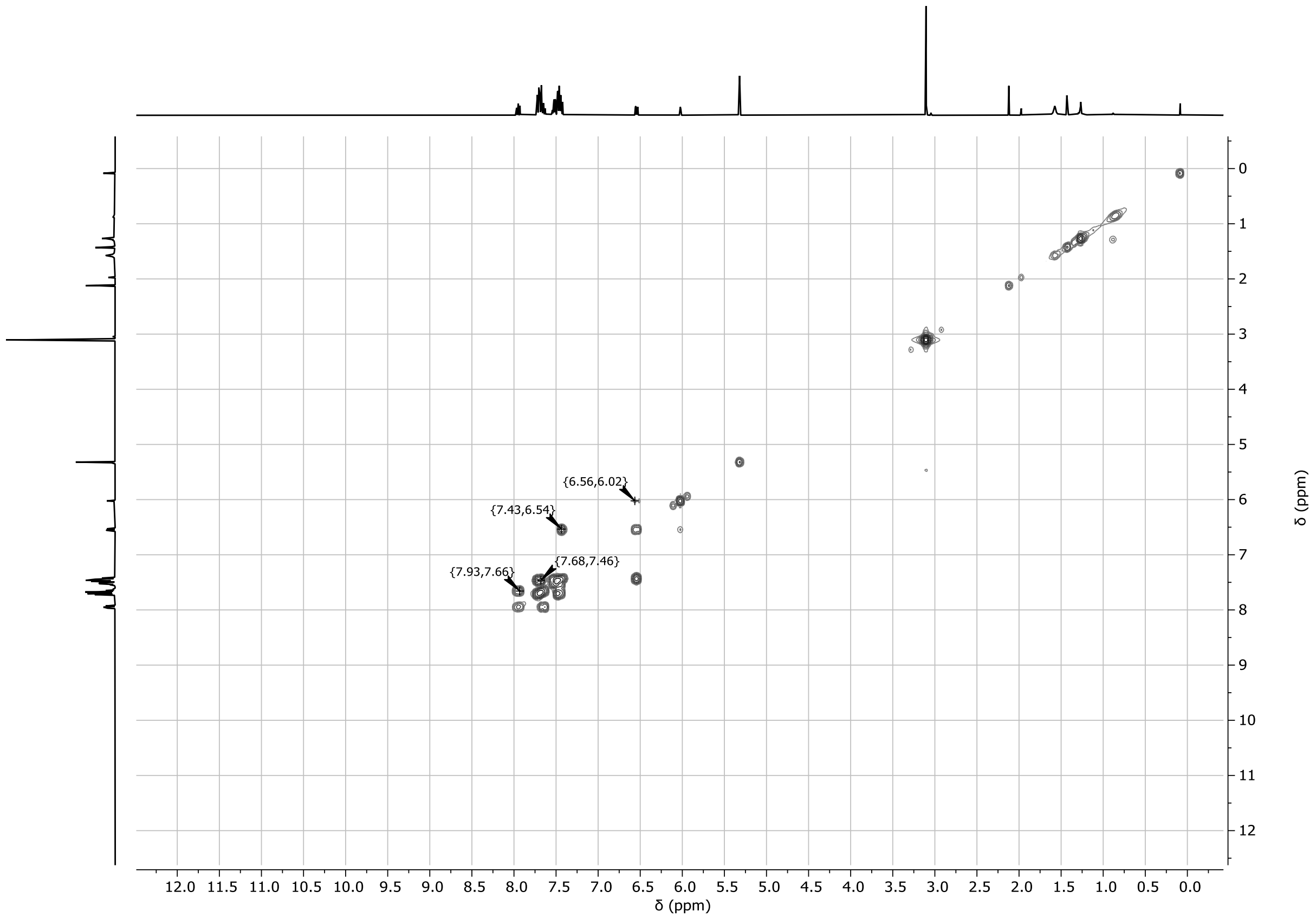

Figure S-199. HH-COSY-NMR spectrum (400 MHz, DCM-d_2) of **Pt(L$_{mOMe}$)PPh$_3$**.

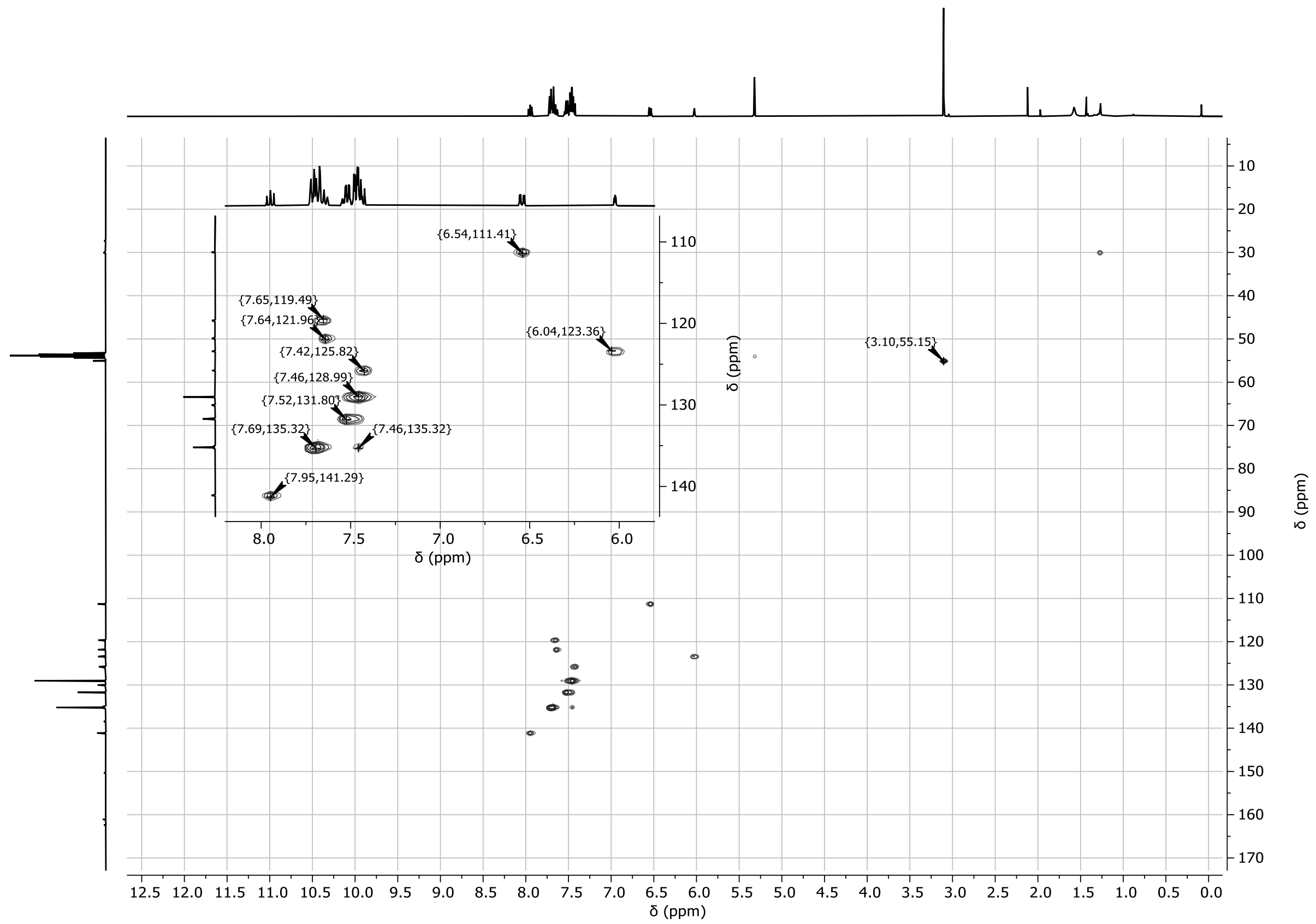

Figure S-200. HC-HSQC-NMR spectrum (101 MHz (^{13}C), 400 MHz (^{1}H), DCM-d_2) of **Pt(L$_{mOMe}$)PPh$_3$**.

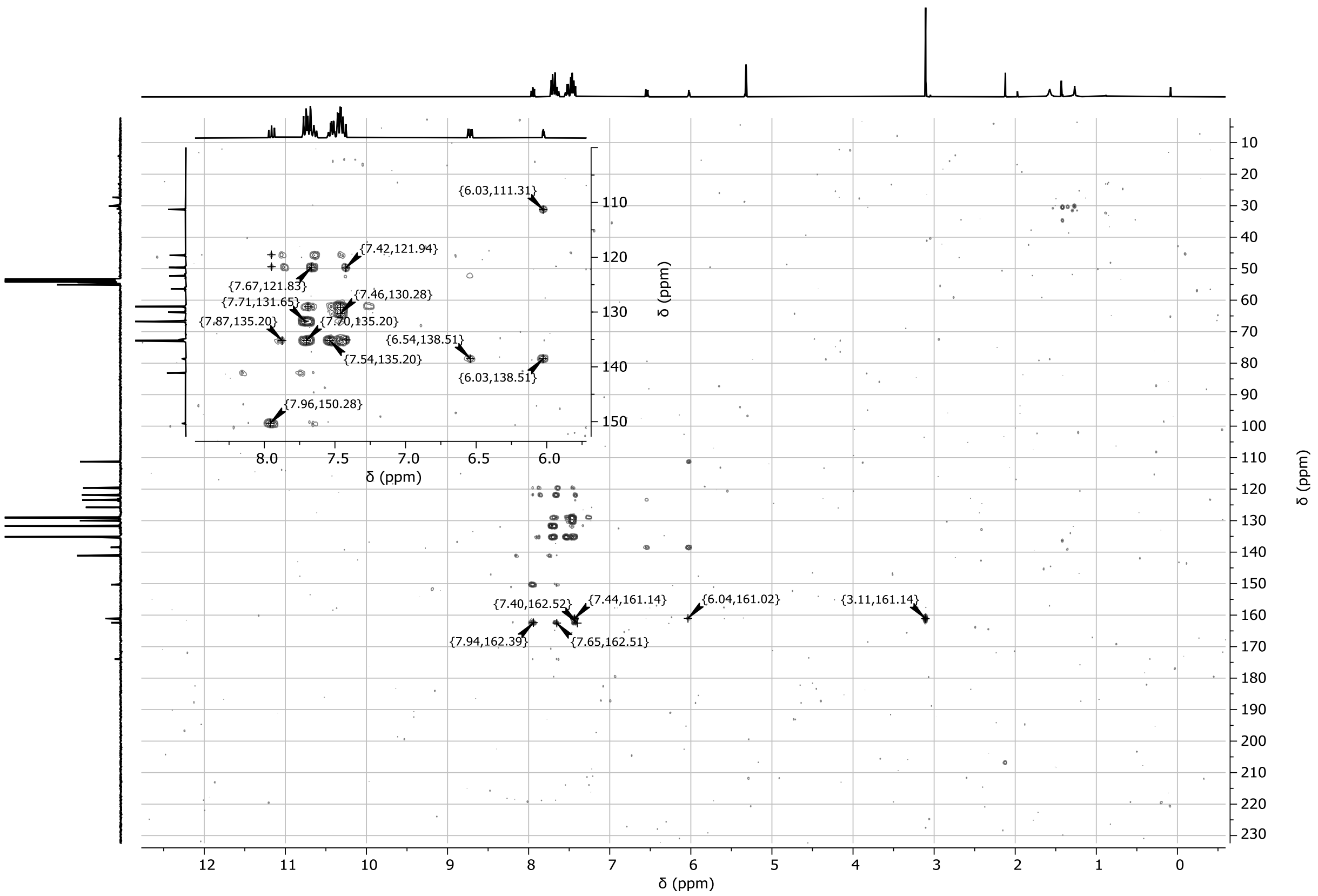

Figure S-201. HC-HMBC-NMR spectrum (101 MHz (^{13}C), 400 MHz (^{1}H), DCM-d_2) of **Pt(L$_{mOMe}$)PPh$_3$**.

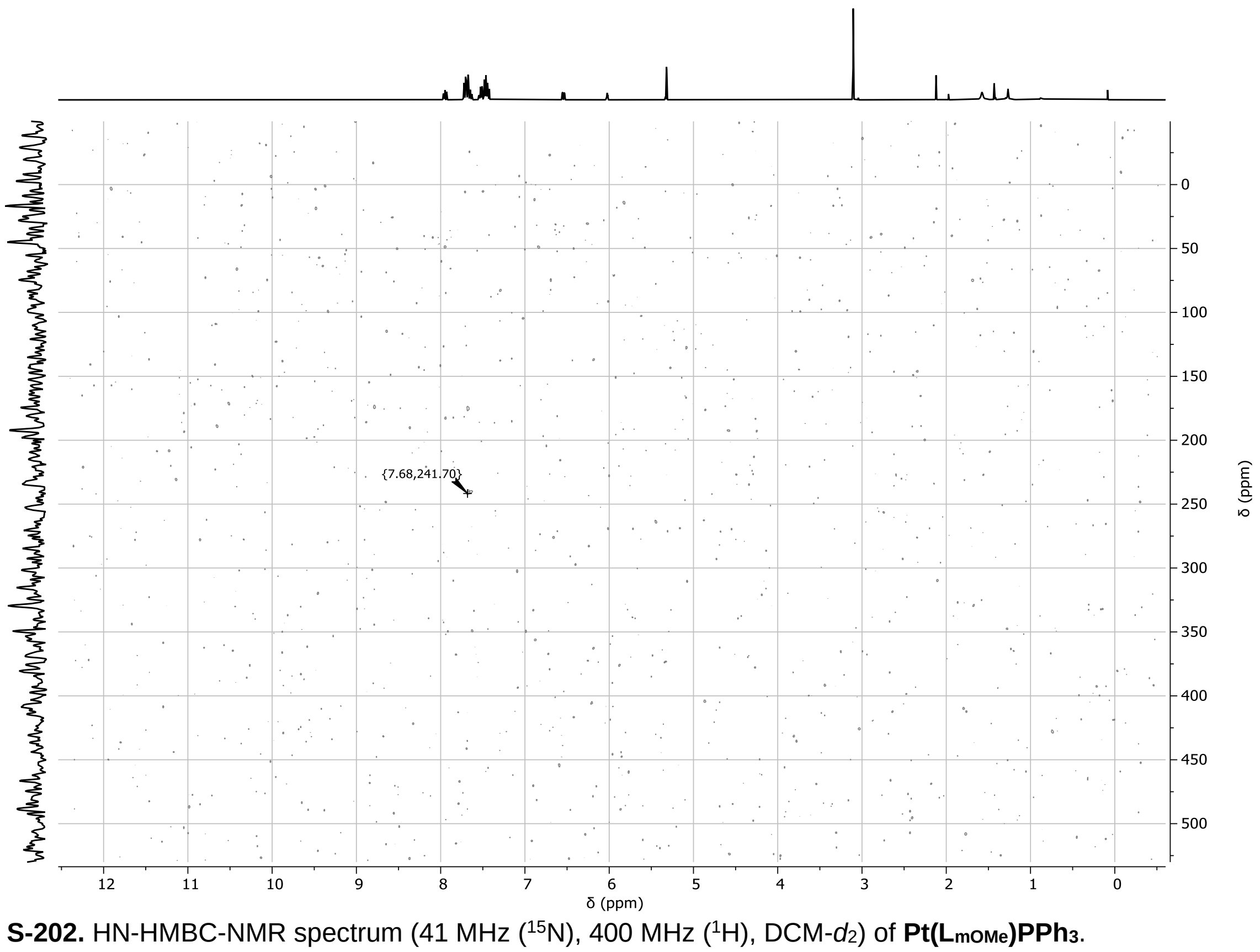

Figure S-202. HN-HMBC-NMR spectrum (41 MHz (^{15}N), 400 MHz (^{1}H), DCM-d_2) of **Pt(L$_{mOMe}$)PPh$_3$**.

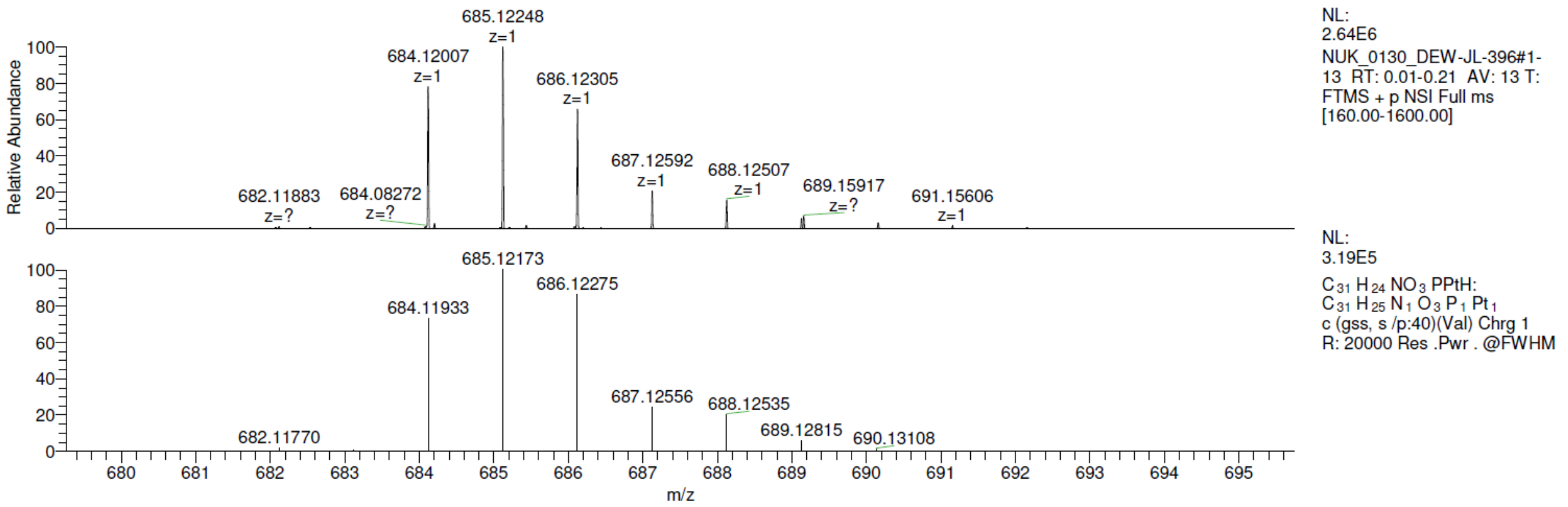

Figure S-203. Mass spectrum of **Pt(L$_{mOMe}$)PPh$_3$** (MeOH). Additional simulation of the [**Pt(L$_{mOMe}$)PPh$_3$**+H]$^+$ adduct.

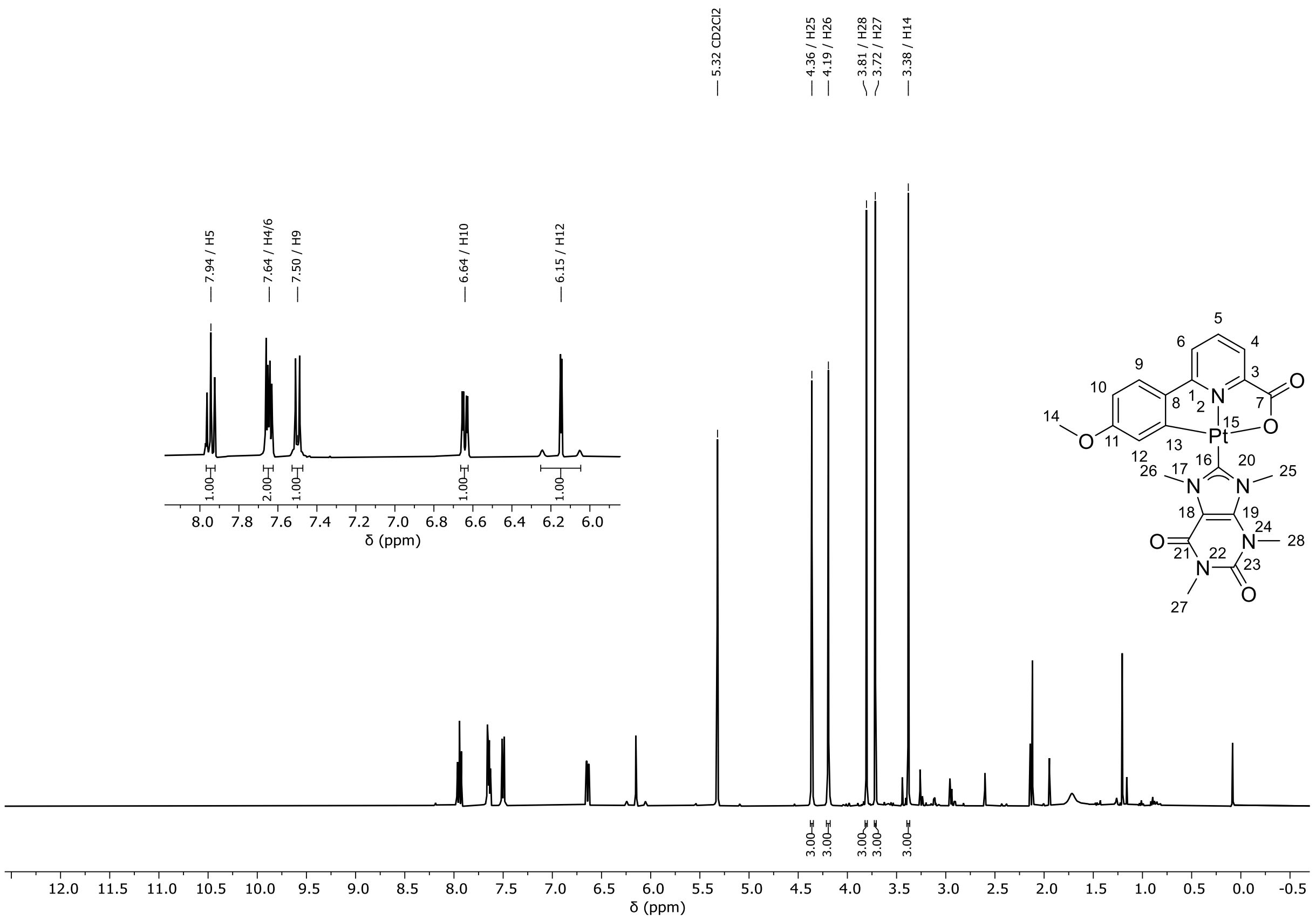

Figure S-204. ^{1}H-NMR spectrum (400 MHz, DCM-d_2) of **Pt(L$_{mOMe}$)NHC**.

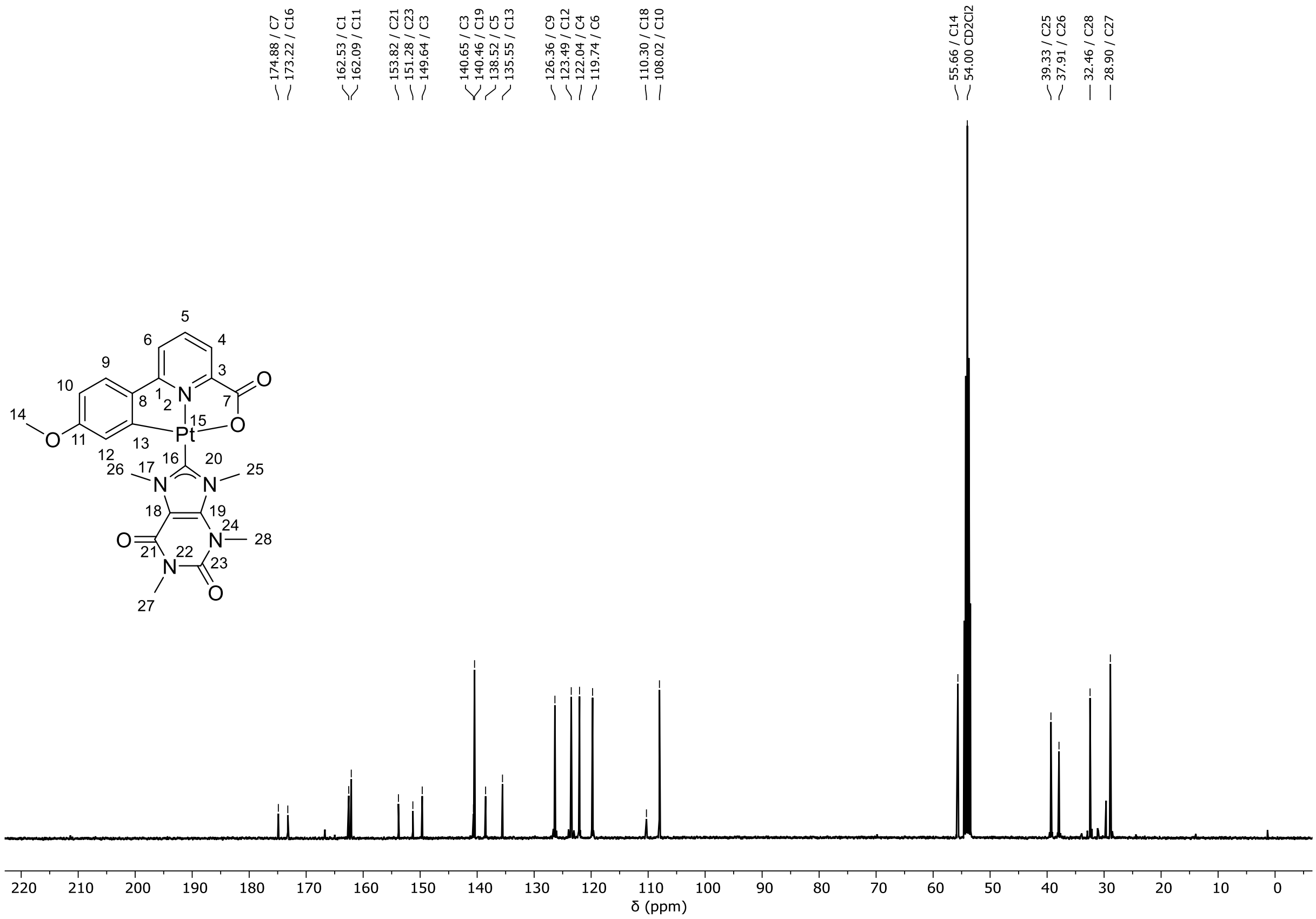

Figure S-205. $^{13}C\{^{1}H\}$-NMR spectrum (101 MHz, DCM-d_2) of **Pt(L$_{mOMe}$)NHC**.

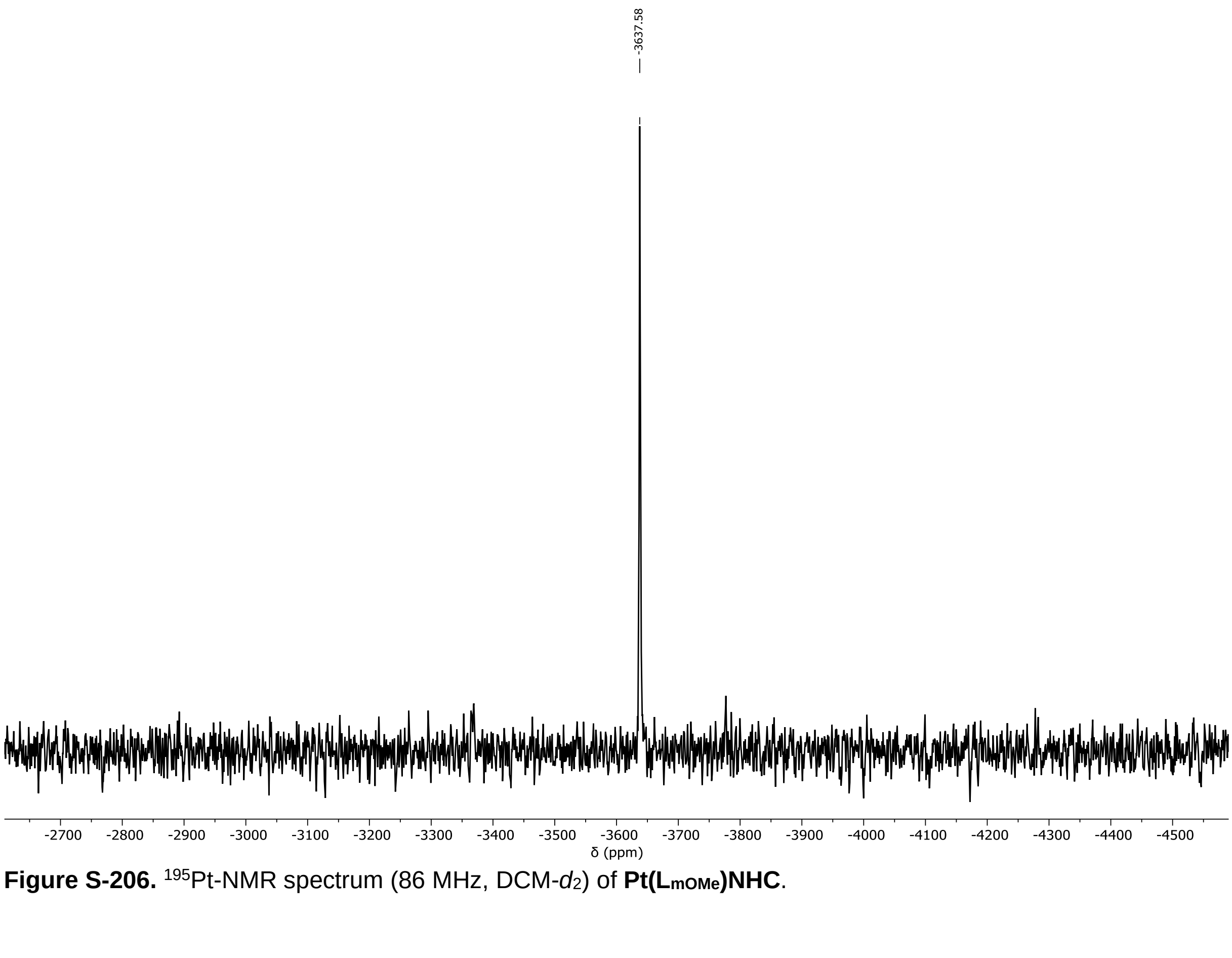

Figure S-206. ^{195}Pt-NMR spectrum (86 MHz, DCM-d_2) of **Pt(L$_{mOMe}$)NHC**.

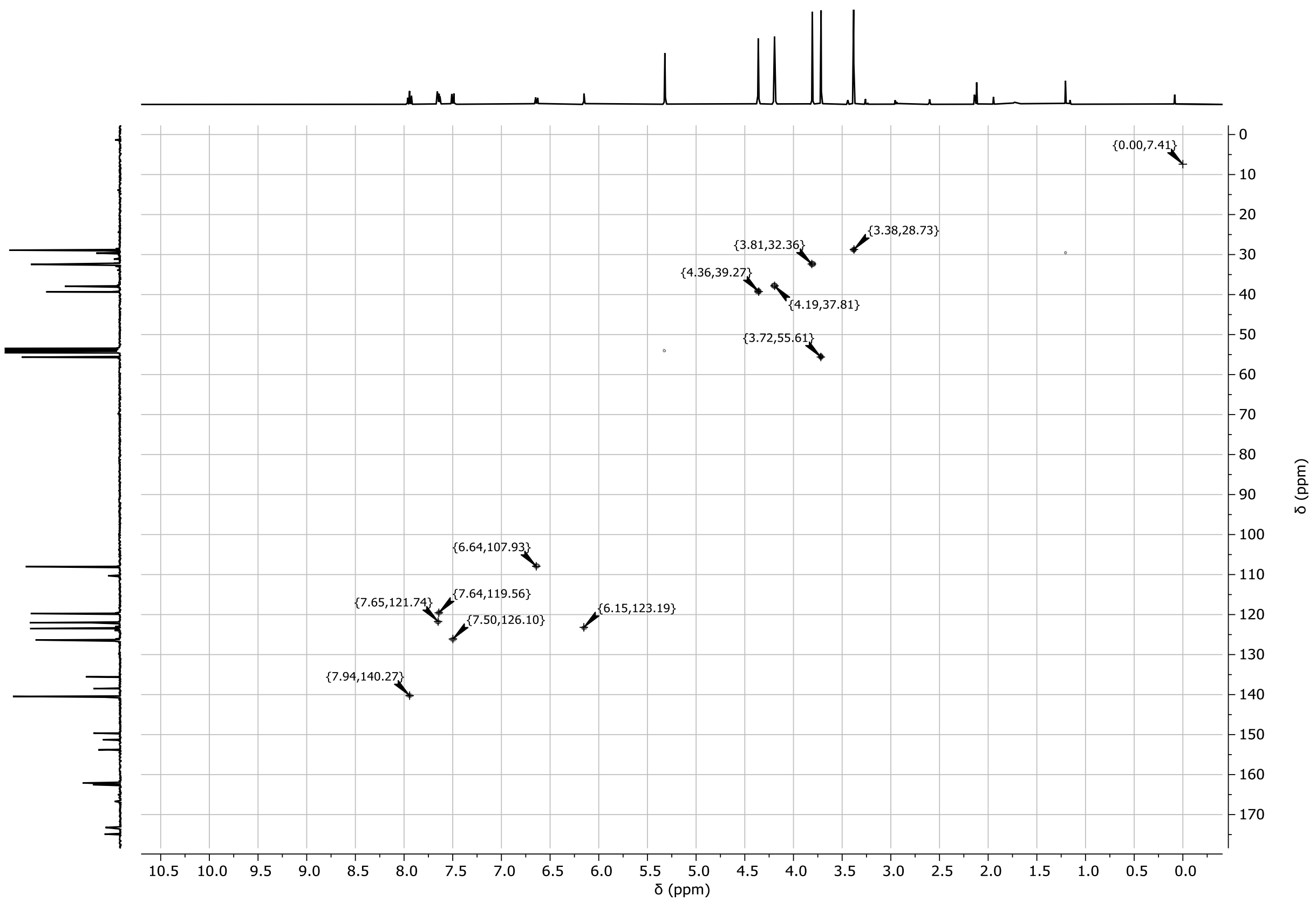

Figure S-207. HC-HSQC-NMR spectrum (101 MHz (^{13}C), 400 MHz (^{1}H), DCM-d_2) of **Pt(L$_{mOMe}$)NHC.**

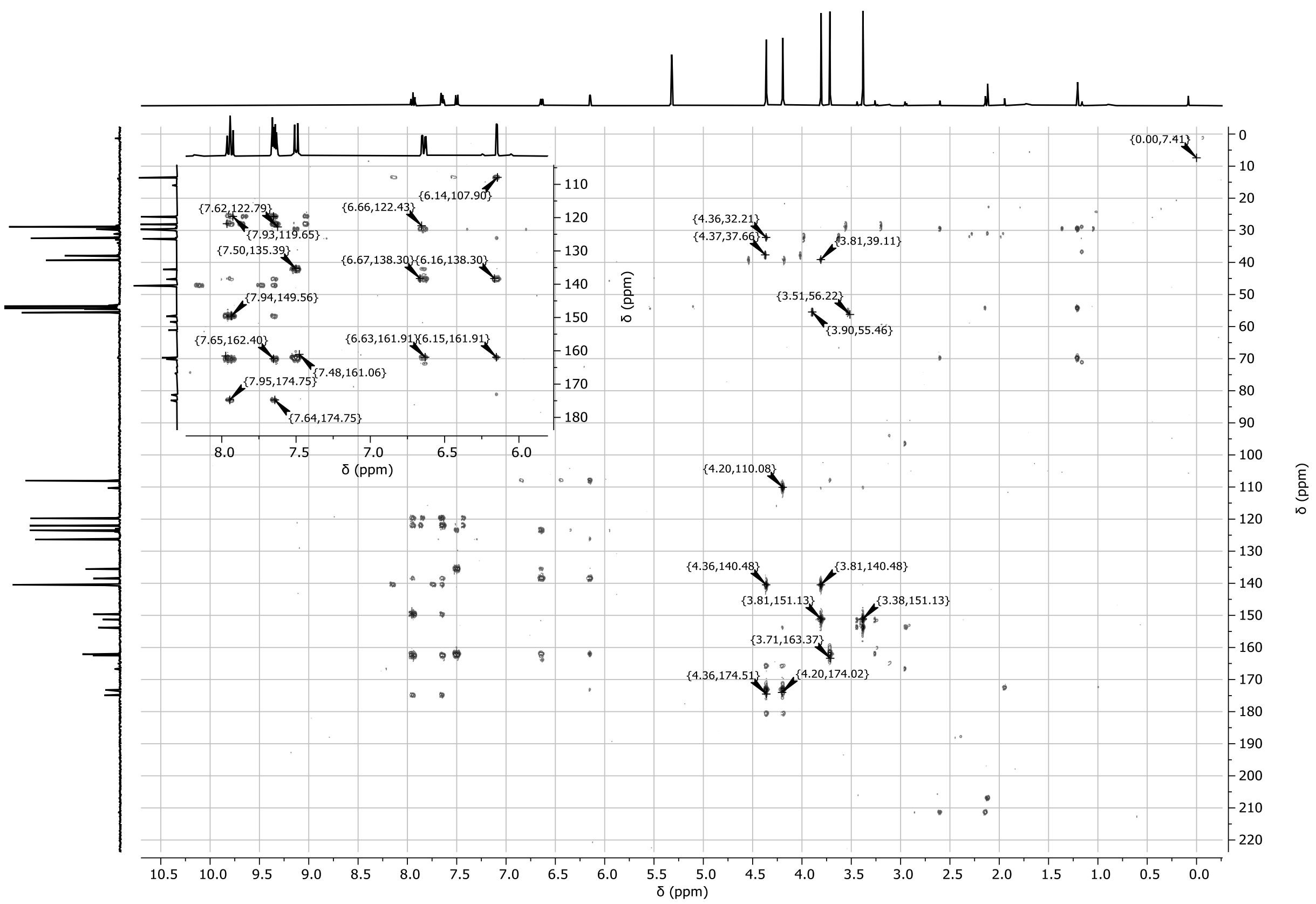

Figure S-208. HC-HMBC-NMR spectrum (101 MHz (^{13}C), 400 MHz (^{1}H), DCM-d_2) of **Pt(L$_{mOMe}$)NHC**.

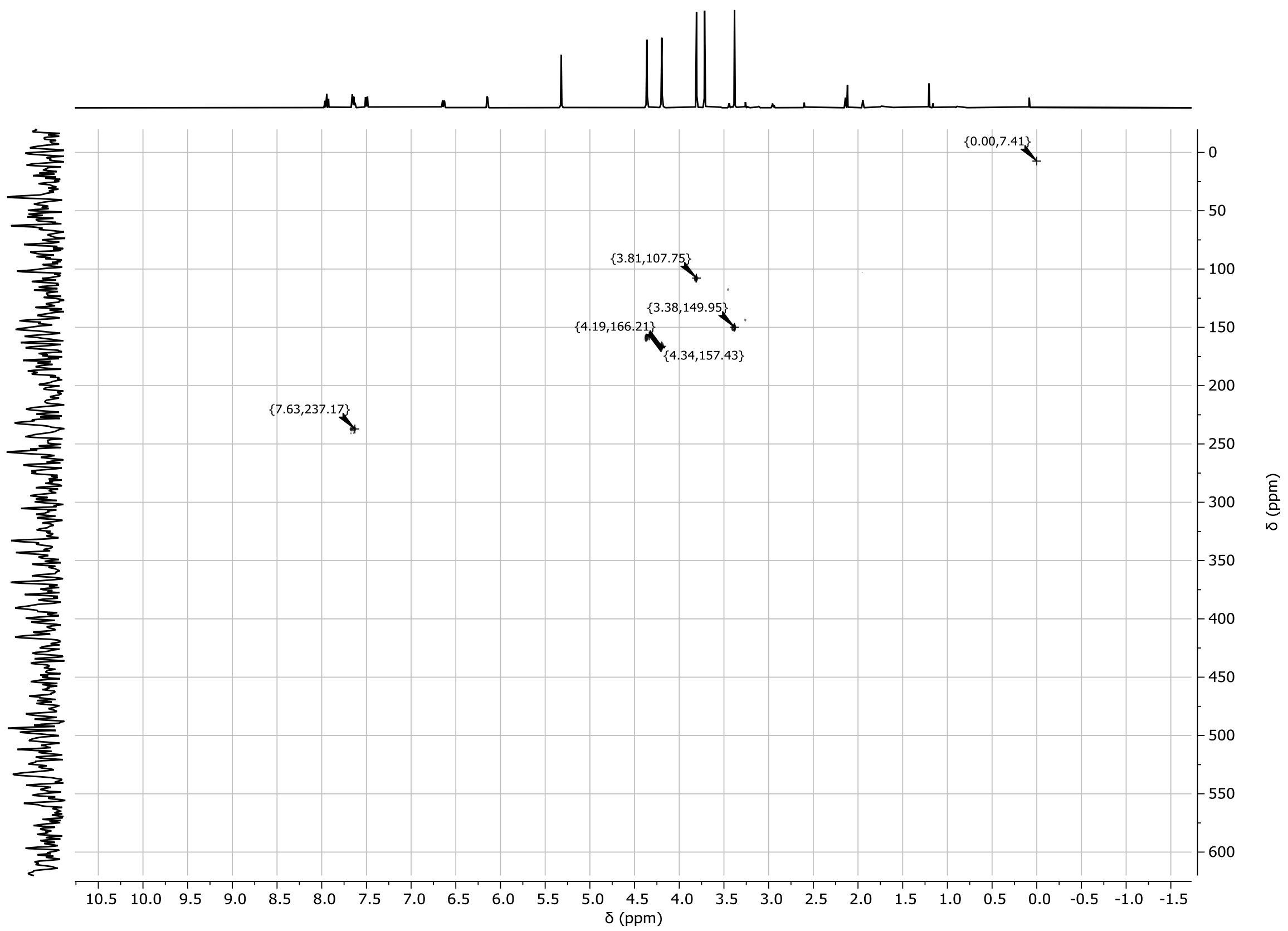

Figure S-209. HN-HMBC-NMR spectrum (41 MHz (^{15}N), 400 MHz (^{1}H), DCM-d_2) of **Pt(L$_{mOMe}$)NHC**.

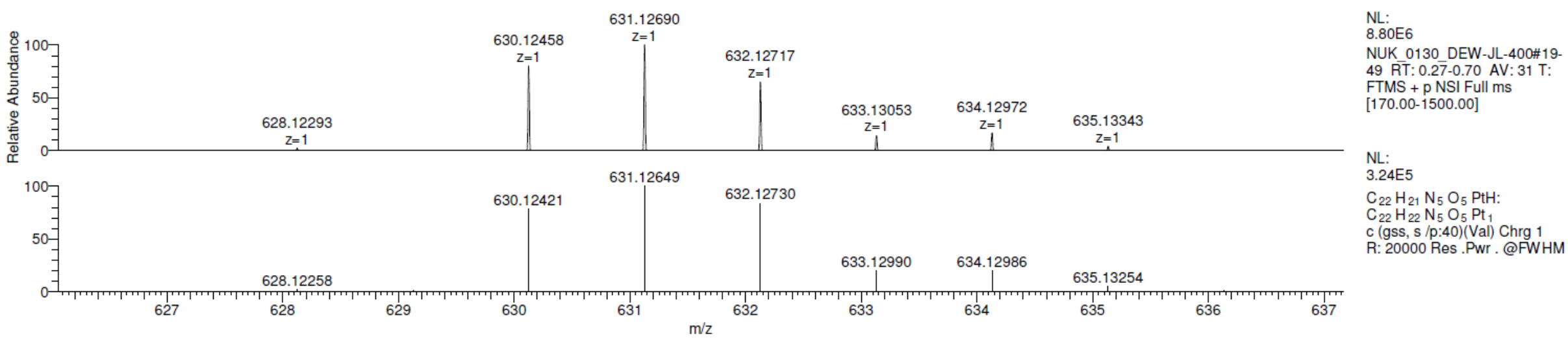

Figure S-210. Mass spectrum of **Pt(L$_{mOMe}$)NHC** (MeOH). Additional simulation of the [**Pt(L$_{mOMe}$)NHC**+H]$^+$ adduct.

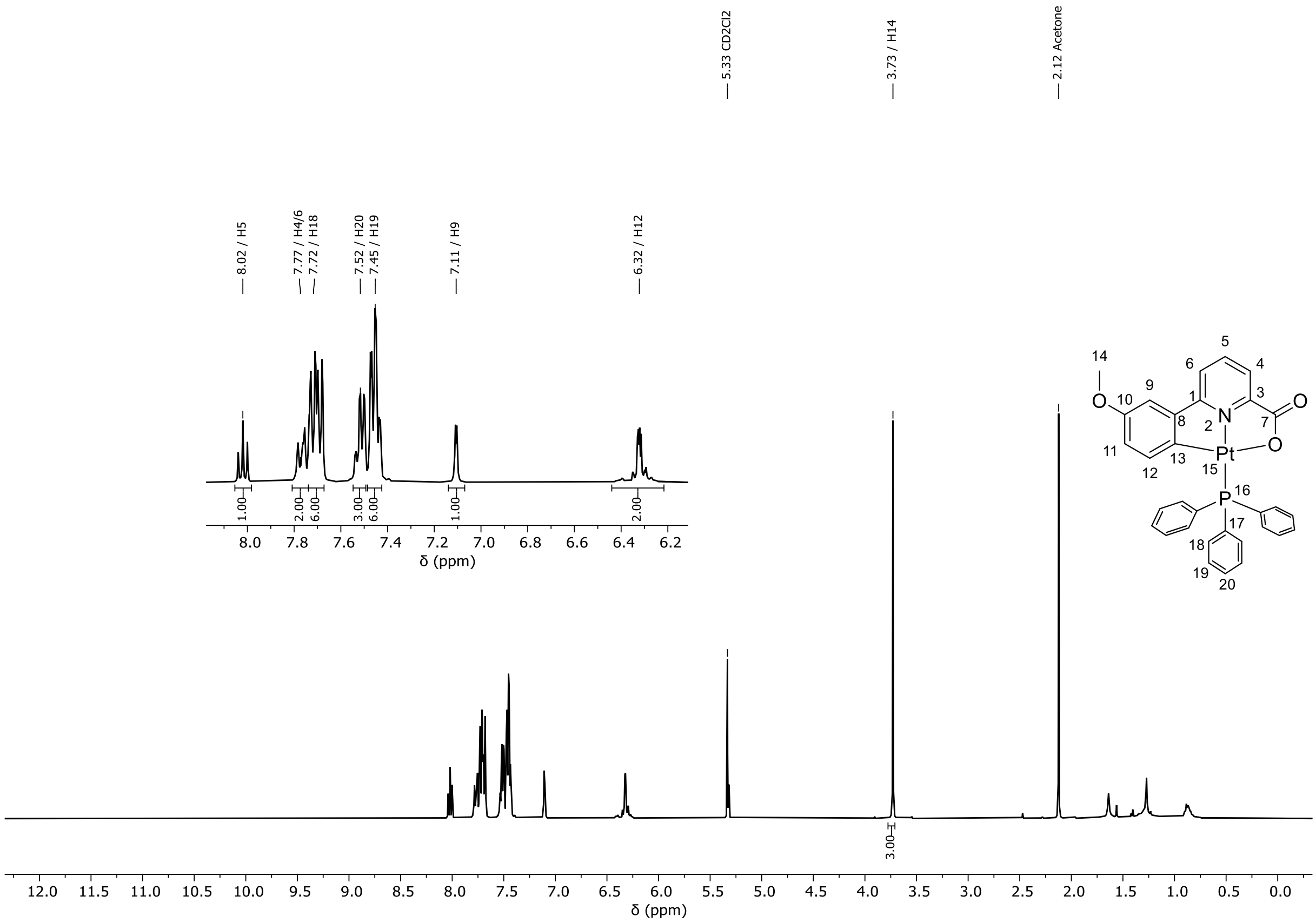

Figure S-211. ^{1}H-NMR spectrum (400 MHz, DCM-d_2) of **Pt(L$_{pOMe}$)PPh$_3$**.

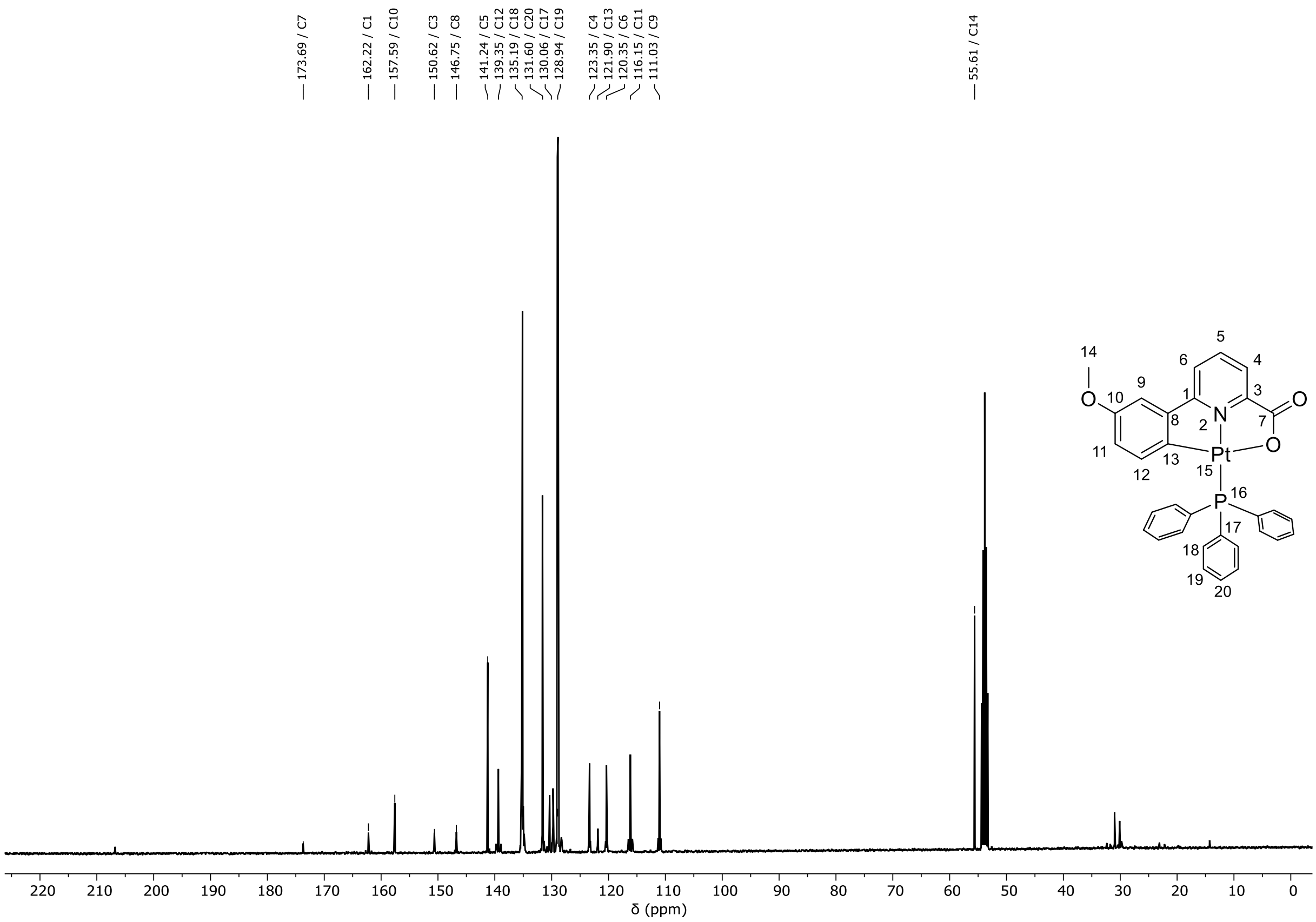

Figure S-212. ^{13}C{^{1}H}-NMR spectrum (101 MHz, DCM-d_2) of **Pt(L$_{pOMe}$)PPh$_3$**.

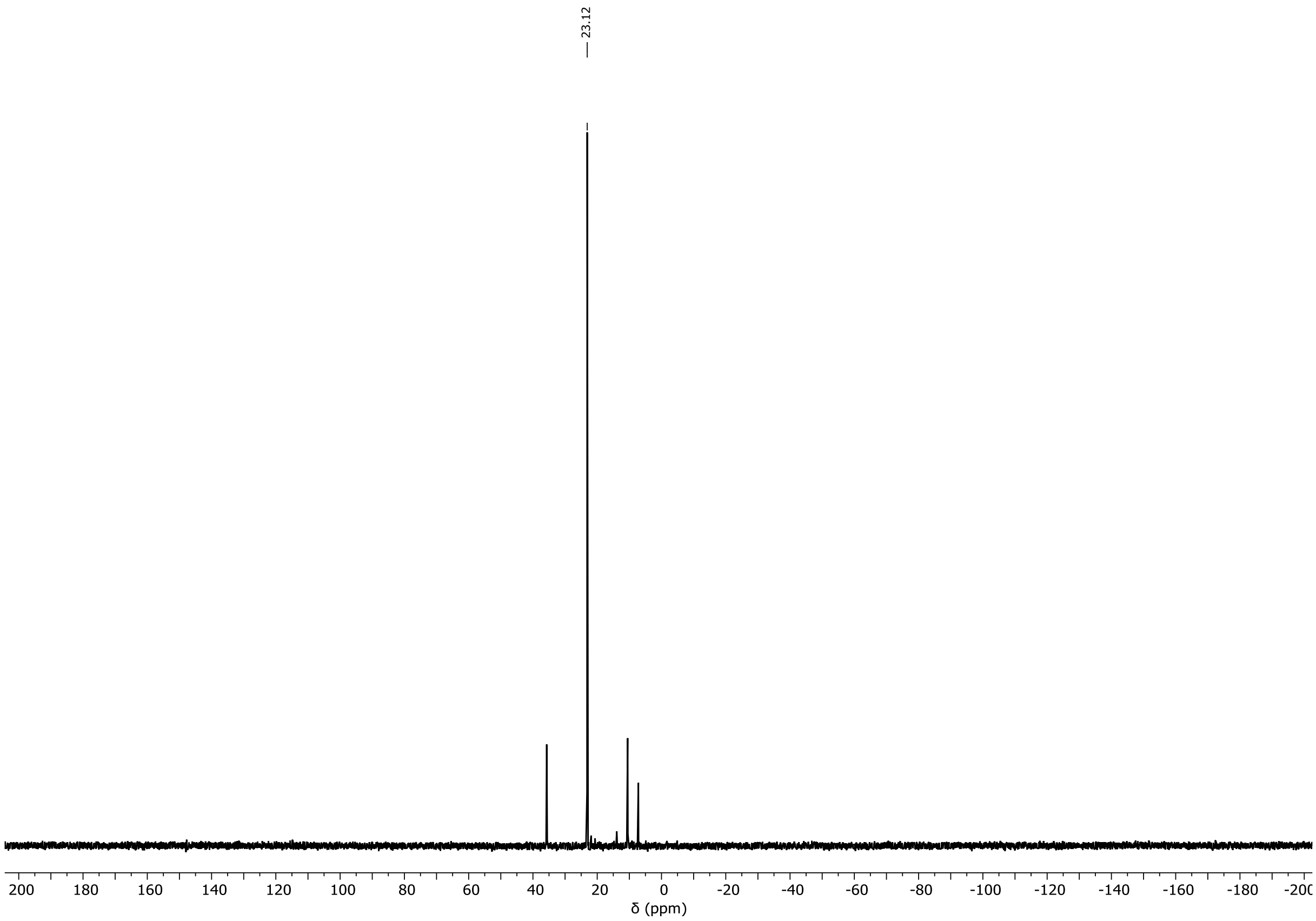

Figure S-213. $^{31}P\{^{1}H\}$-NMR spectrum (162 MHz, DCM-d_2) of **Pt(L$_{pOMe}$)PPh$_3$**.

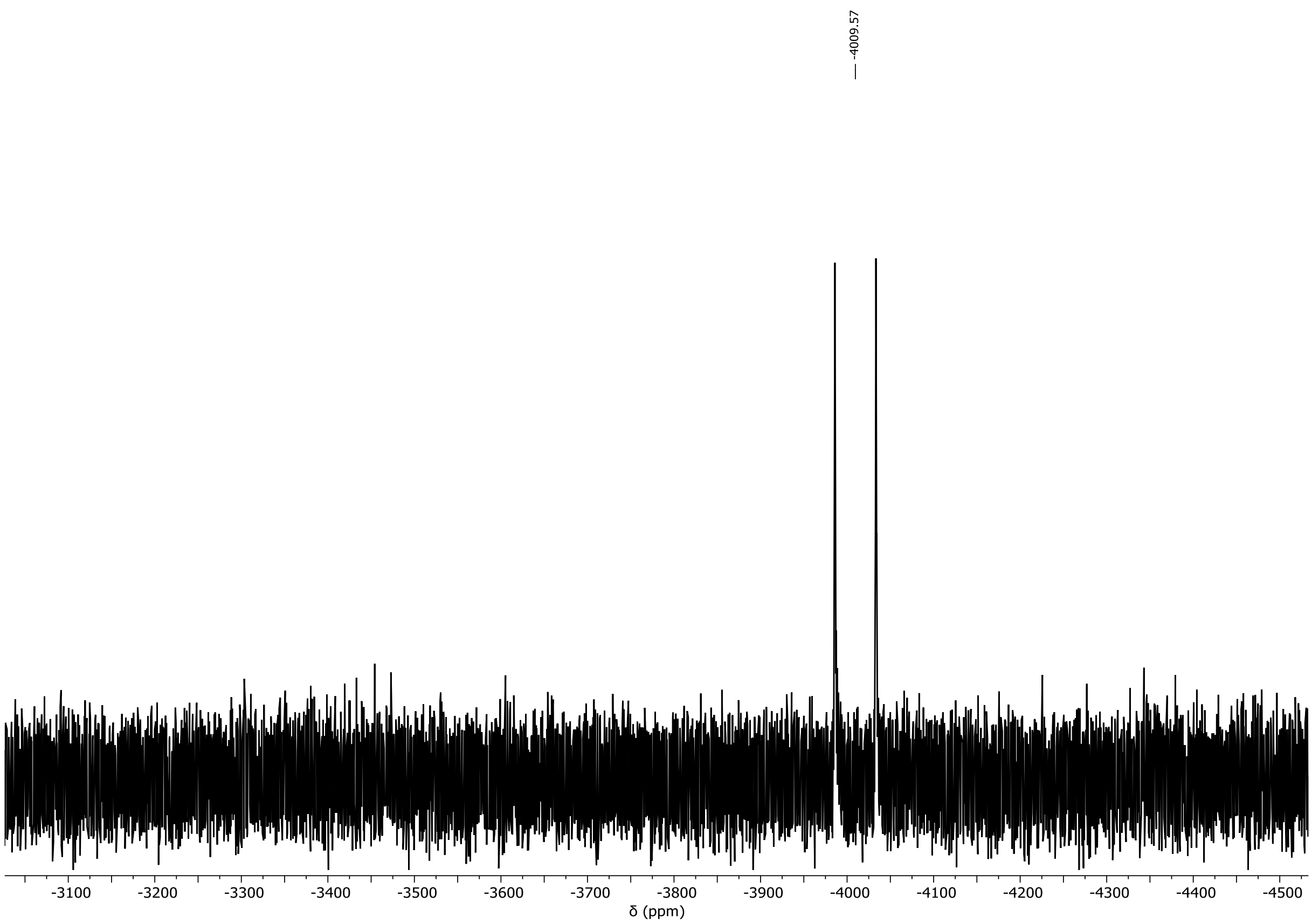

Figure S-214. ^{195}Pt-NMR spectrum (86 MHz, DCM-d_2) of **Pt(L$_{pOMe}$)PPh$_3$**.

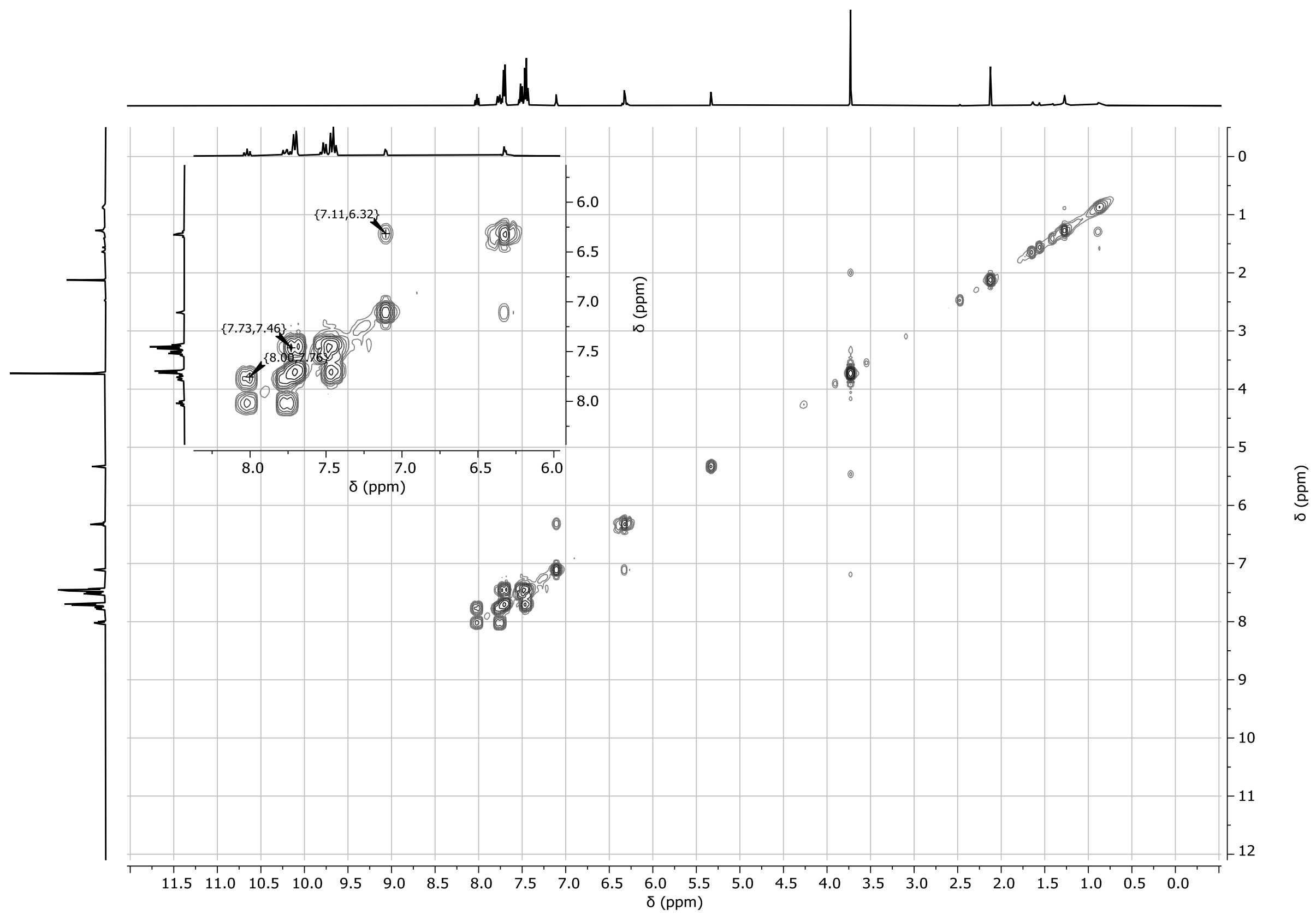

Figure S-215. HH-COSY-NMR spectrum (400 MHz, DCM-d_2) of **Pt(L$_{\mathbf{pOMe}}$)PPh$_3$**.

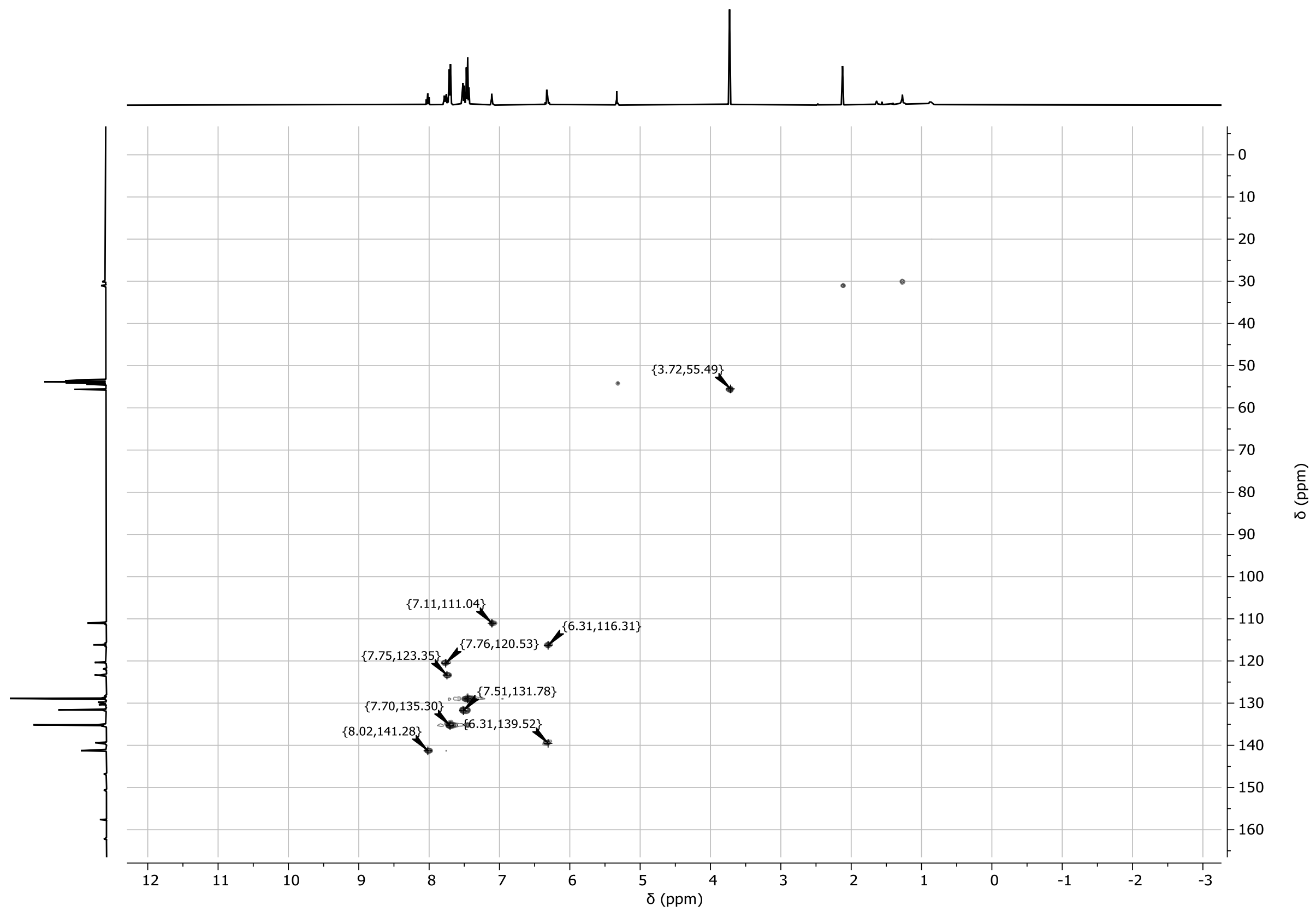

Figure S-216. HC-HSQC-NMR spectrum (101 MHz (^{13}C), 400 MHz (^{1}H), DCM-d_2) of **Pt(L$_{\mathbf{pOMe}}$)PPh$_3$**.

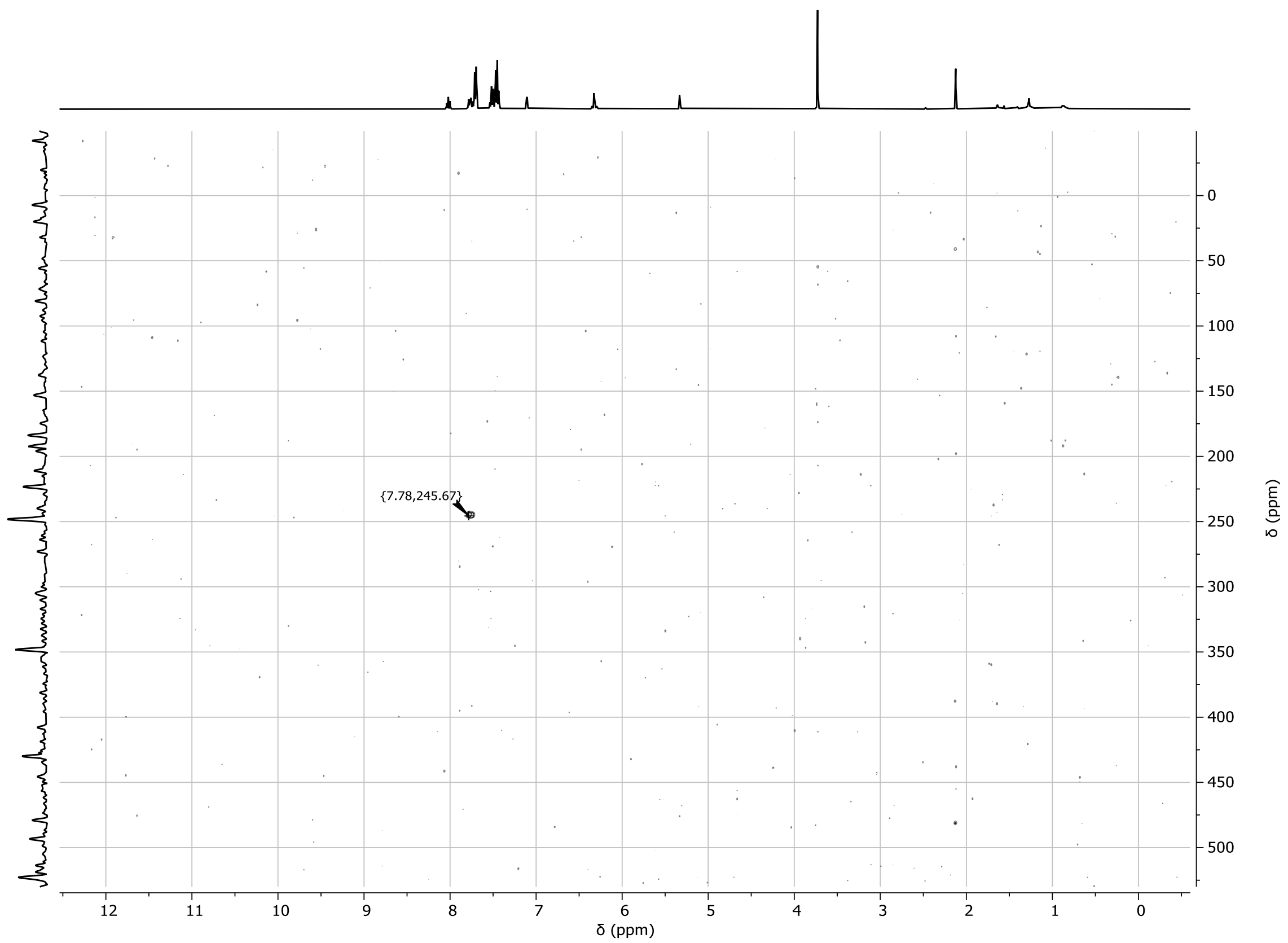

Figure S-217. HN-HMBC-NMR spectrum (41 MHz (^{15}N), 400 MHz (^{1}H), DCM-d_2) of **Pt(L$_{pOMe}$)PPh$_3$**.

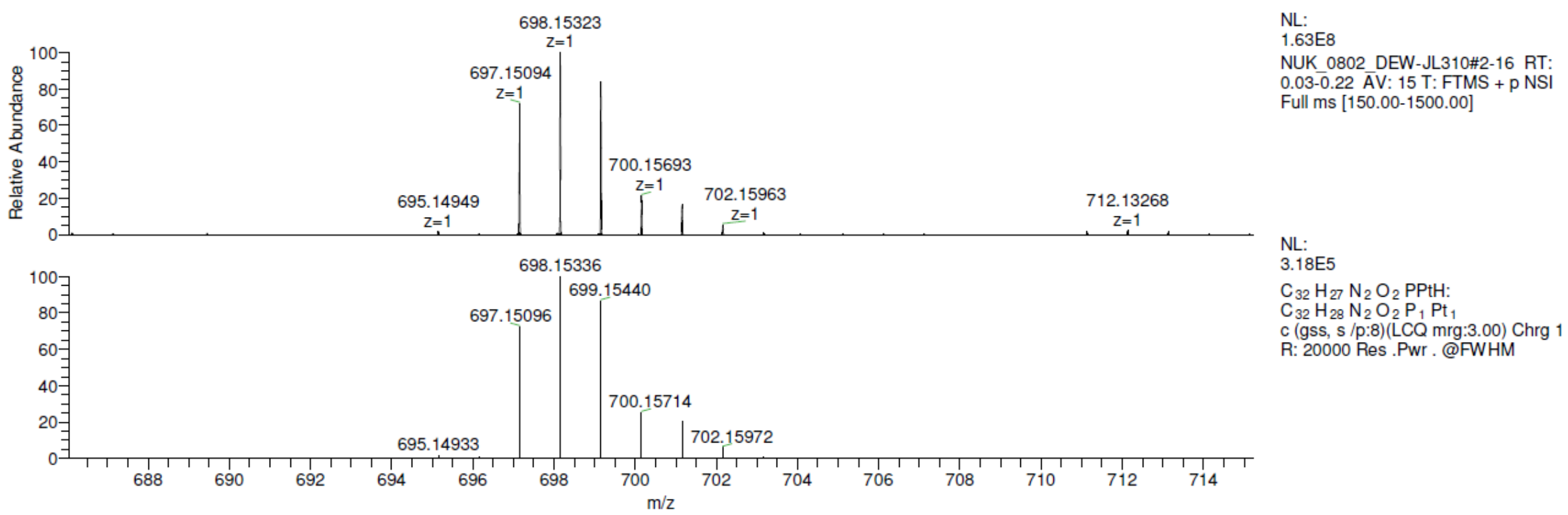

Figure S-218. Mass spectrum of **Pt(L$_{pOMe}$)PPh$_3$** (MeOH). Additional simulation of the [**Pt(L$_{pOMe}$)PPh$_3$**+H]$^+$ adduct.

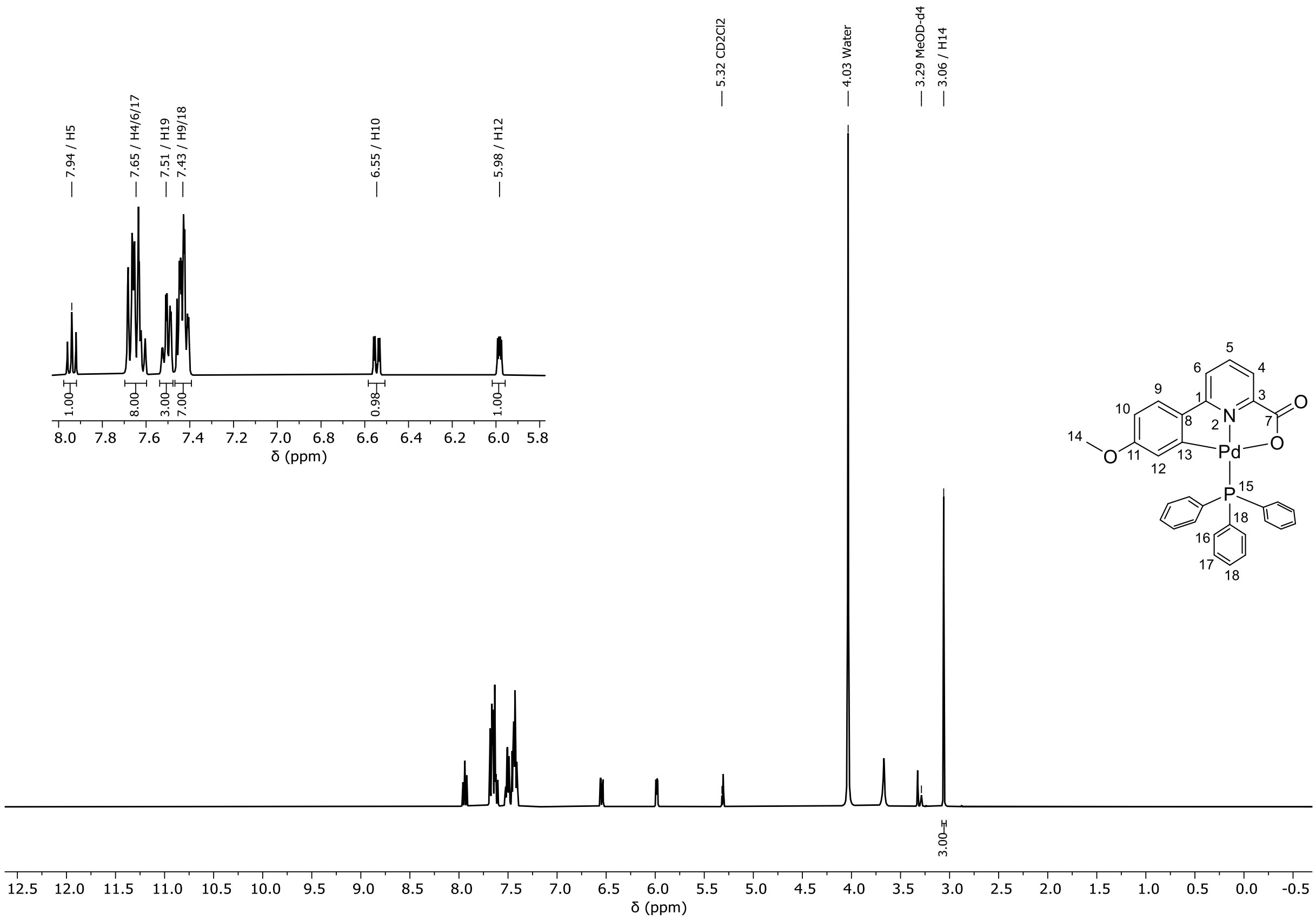

Figure S-219. ^{1}H-NMR spectrum (400 MHz, DCM-d_2) of **Pd(L$_{mOMe}$)PPh$_3$**.

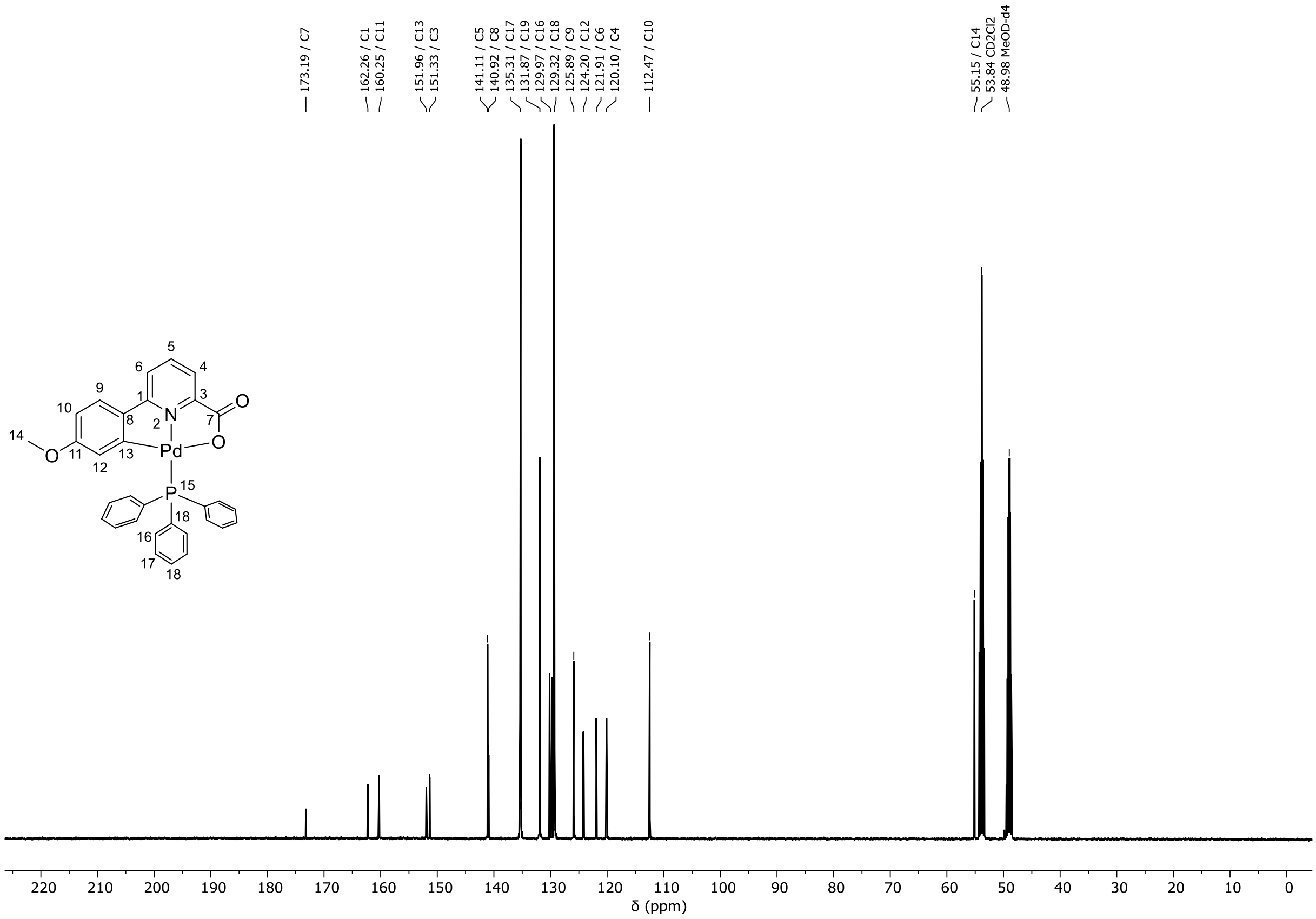

Figure S-220. ^{13}C{^{1}H}-NMR spectrum (126 MHz, DCM-d_2) of **Pd(L$_{mOMe}$)PPh$_3$**.

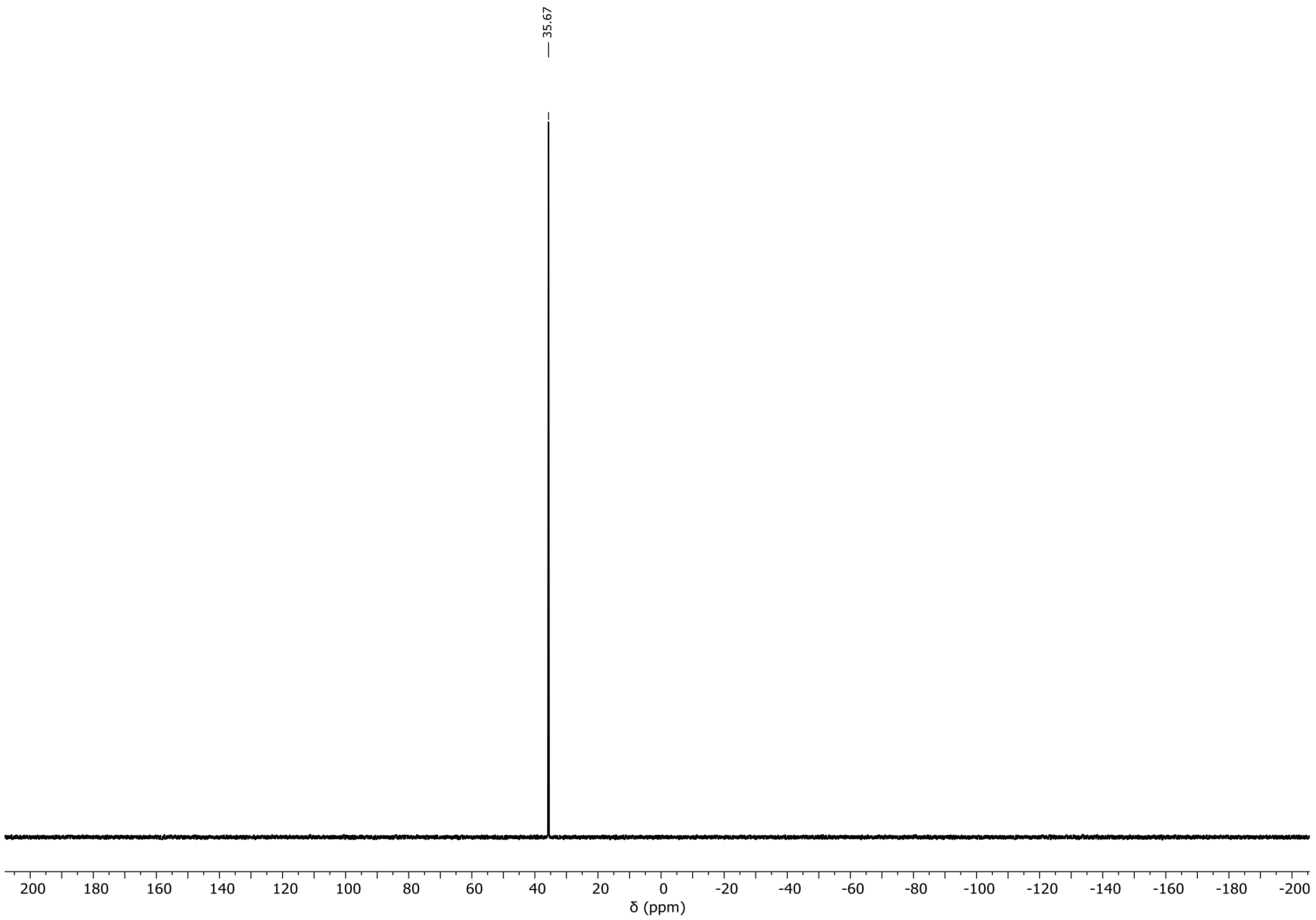

Figure S-221. $^{31}P\{^1H\}$-NMR spectrum (162 MHz, DCM-d_2) of **Pd(L$_{mOMe}$)PPh$_3$**.

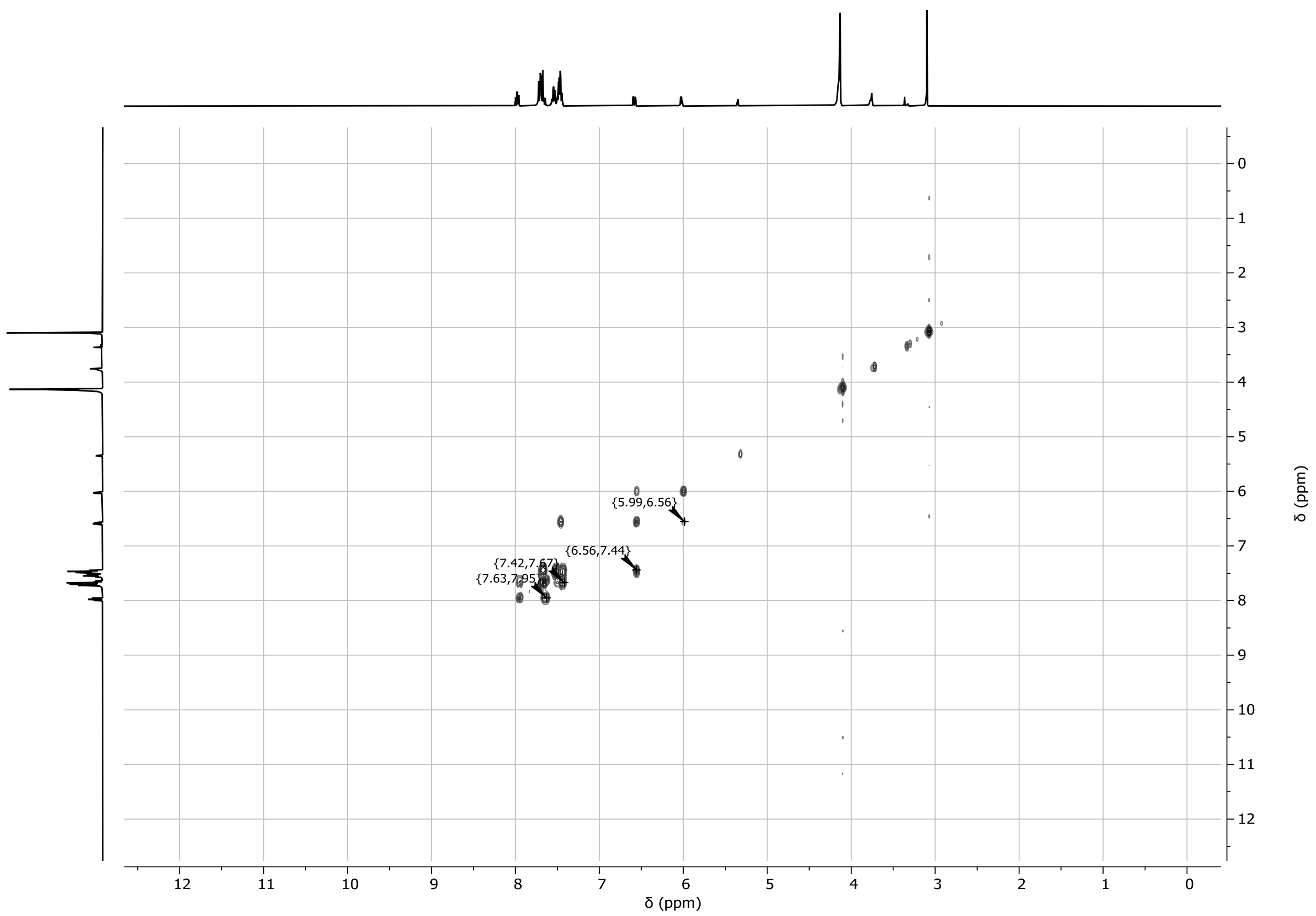

Figure S-222. HH-COSY-NMR spectrum (500 MHz, DCM-d_2) of **Pd(L$_{mOMe}$)PPh$_3$**.

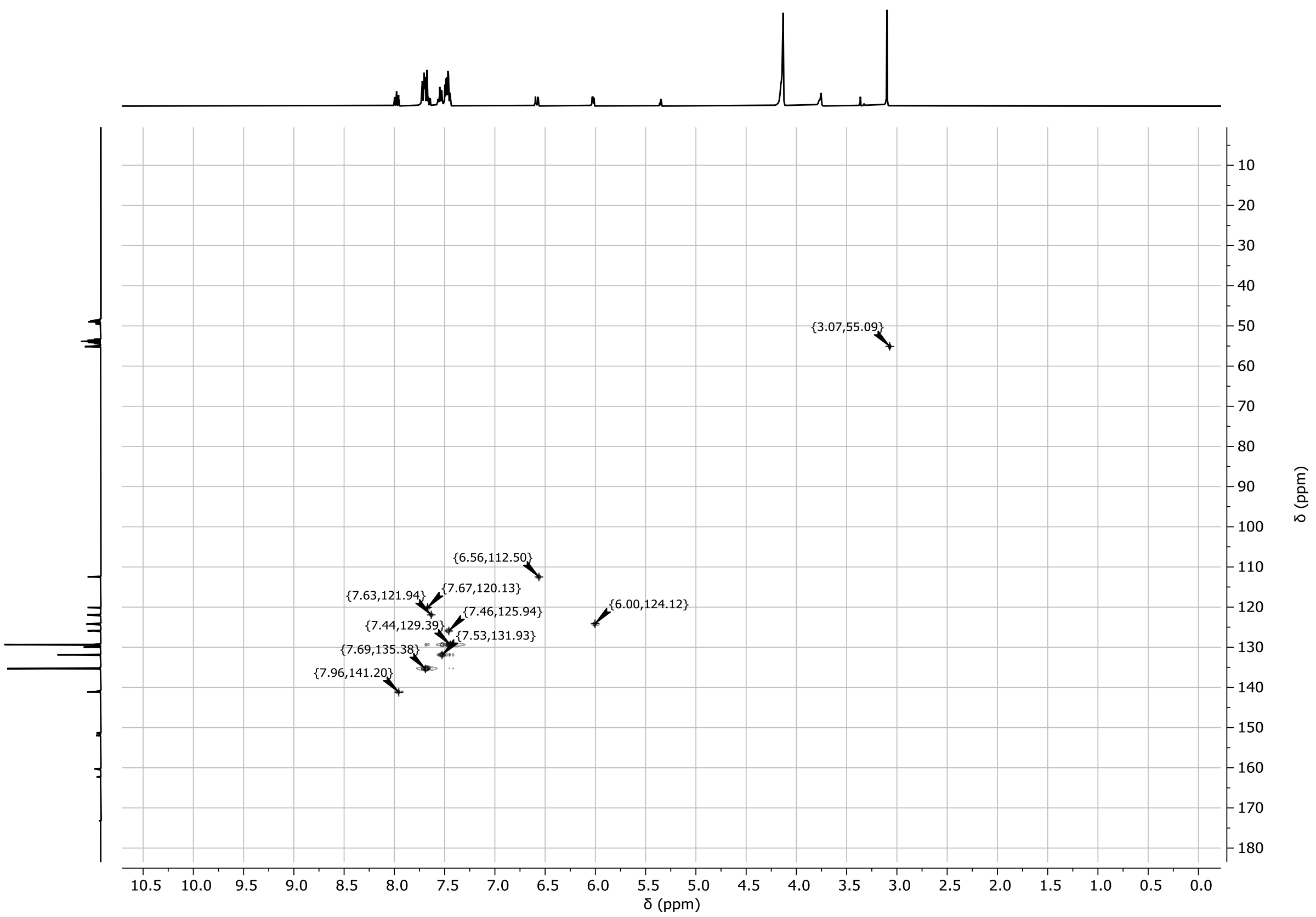

Figure S-223. HC-HSQC-NMR spectrum (126 MHz (^{13}C), 500 MHz (^{1}H), DCM-d_2) of **Pd(L$_{mOMe}$)PPh$_3$**.

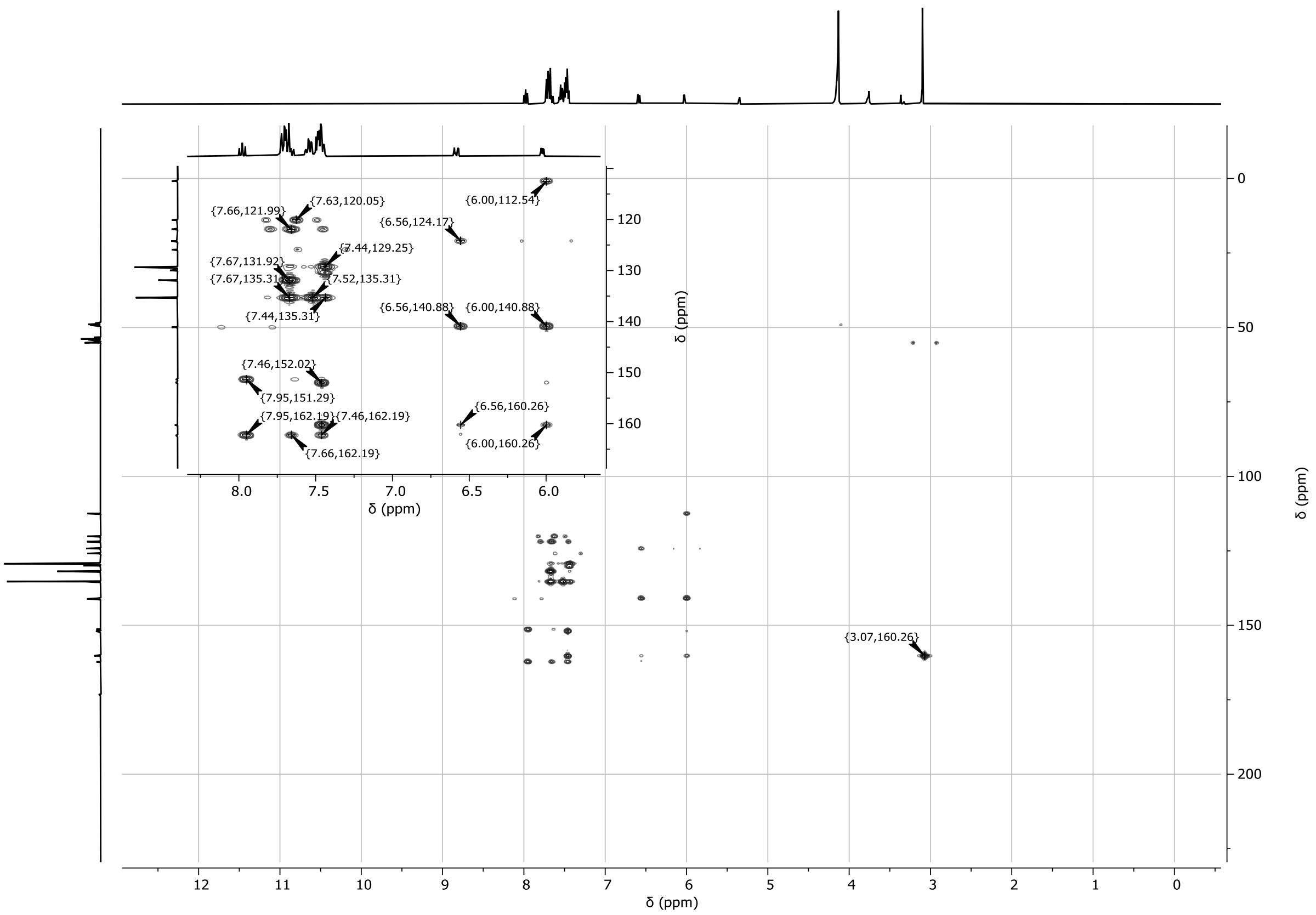

Figure S-224. HC-HMBC-NMR spectrum (126 MHz (^{13}C), 500 MHz (^{1}H), DCM-d_2) of **Pd(L$_{mOMe}$)PPh$_3$**.

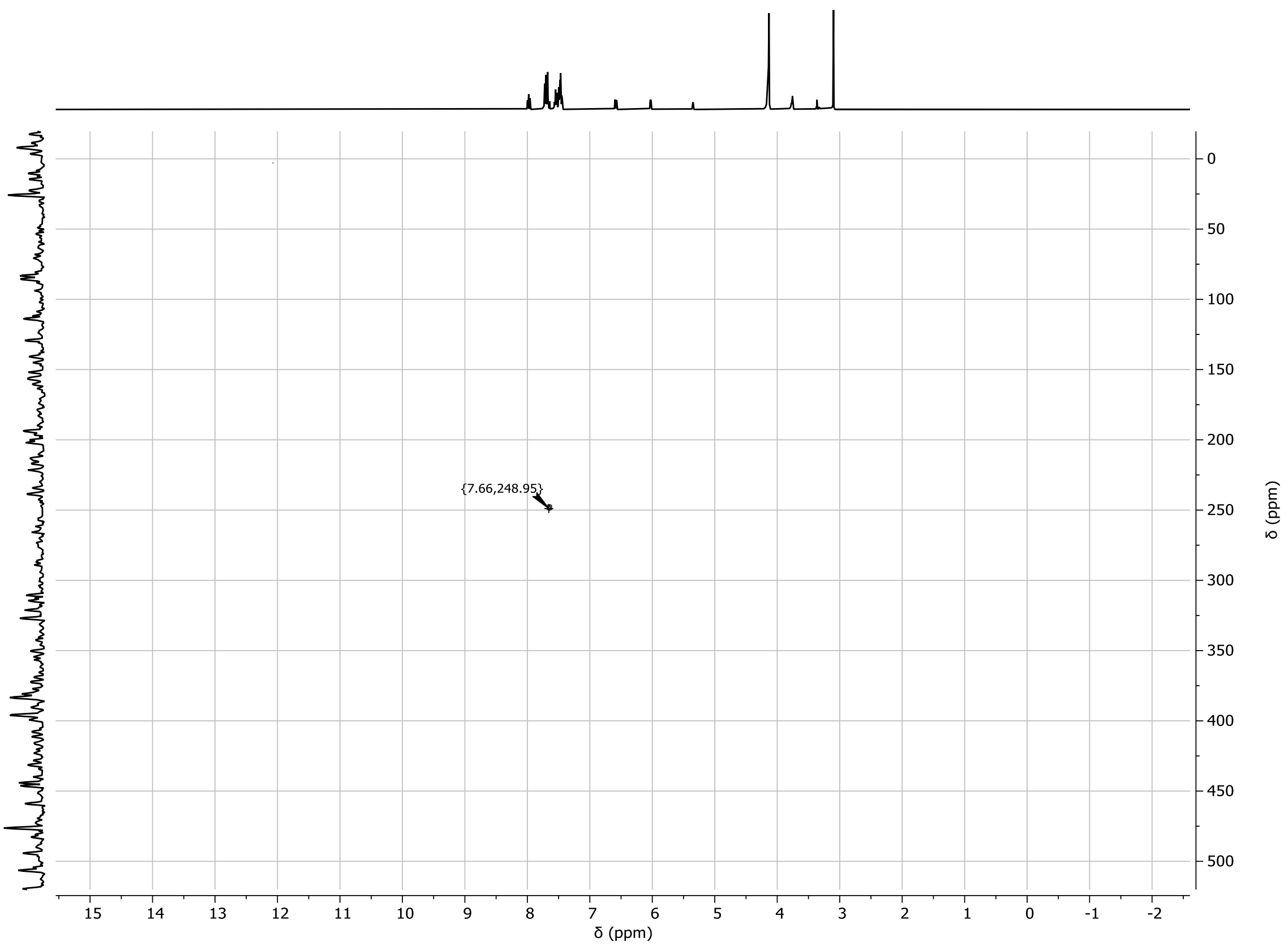

Figure S-225. HN-HMBC-NMR spectrum (51 MHz (^{15}N), 500 MHz (^{1}H), DCM-d_2) of **Pd(L$_{mOMe}$)PPh$_3$**.

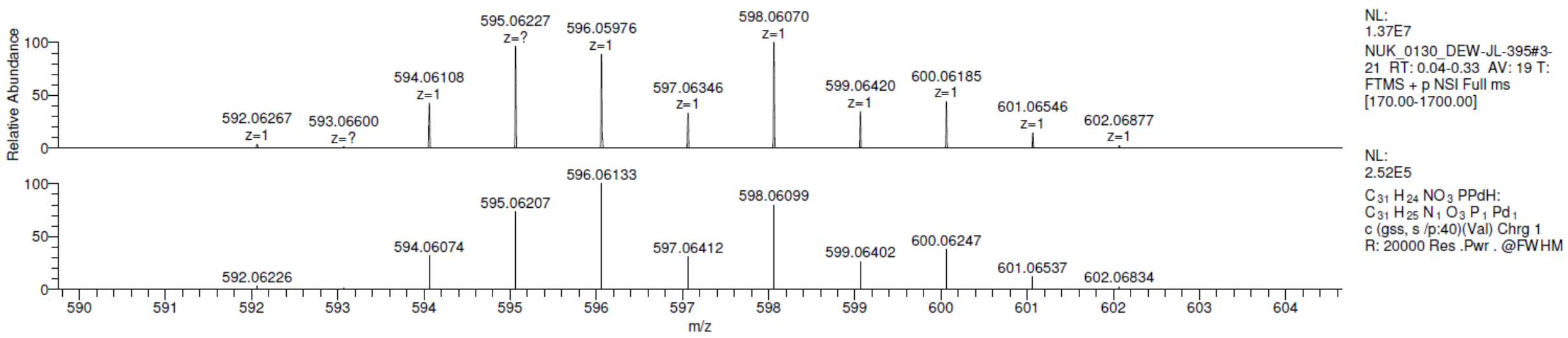

Figure S-226. Mass spectrum of **Pd(L$_{mOMe}$)PPh$_3$** (MeOH). Additional simulation of the [**Pd(L$_{mOMe}$)PPh$_3$**+H]$^+$ adduct.

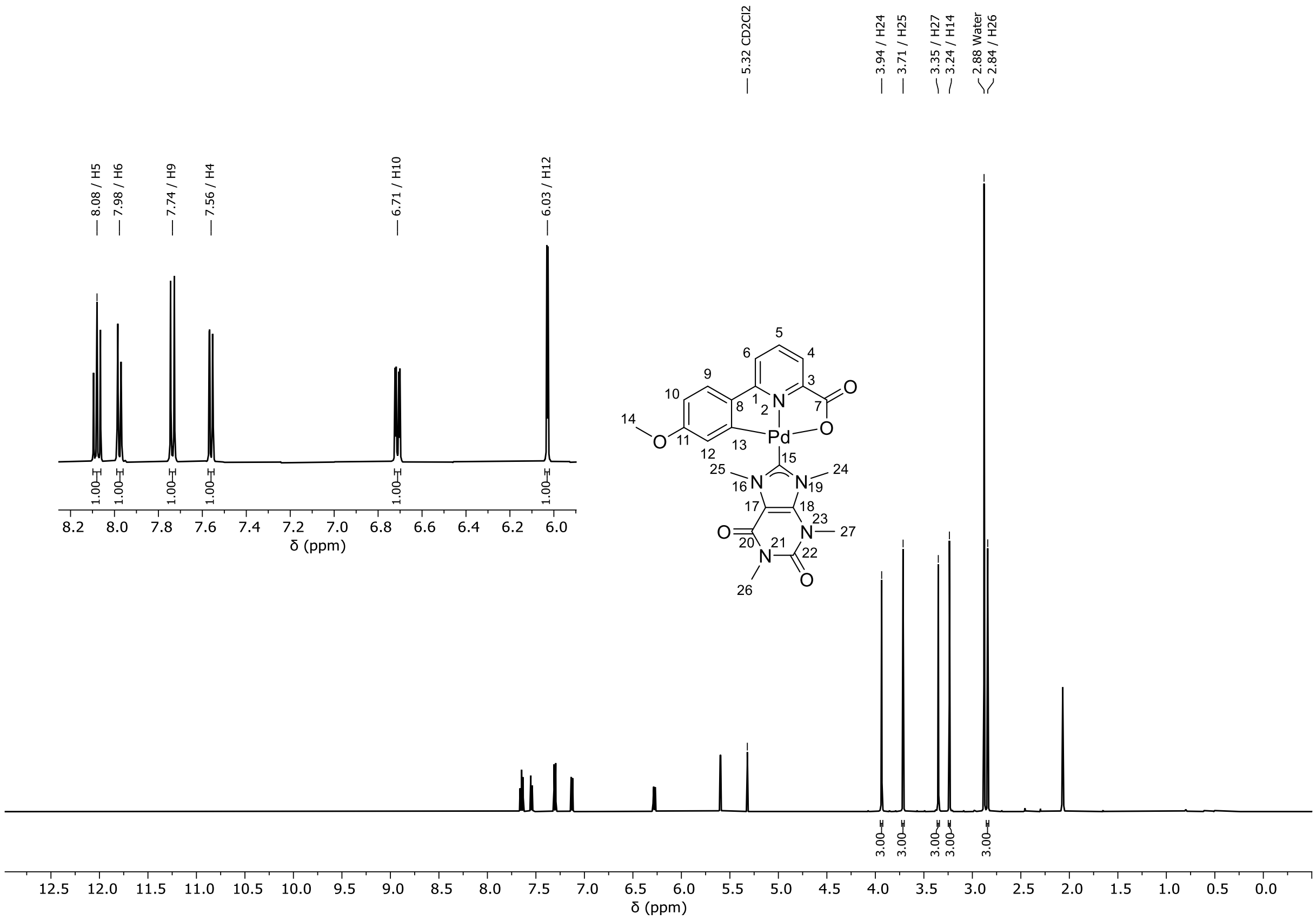

Figure S-227. ^{1}H-NMR spectrum (400 MHz, DCM-d_2) of **Pd(L$_{mOMe}$)NHC**.

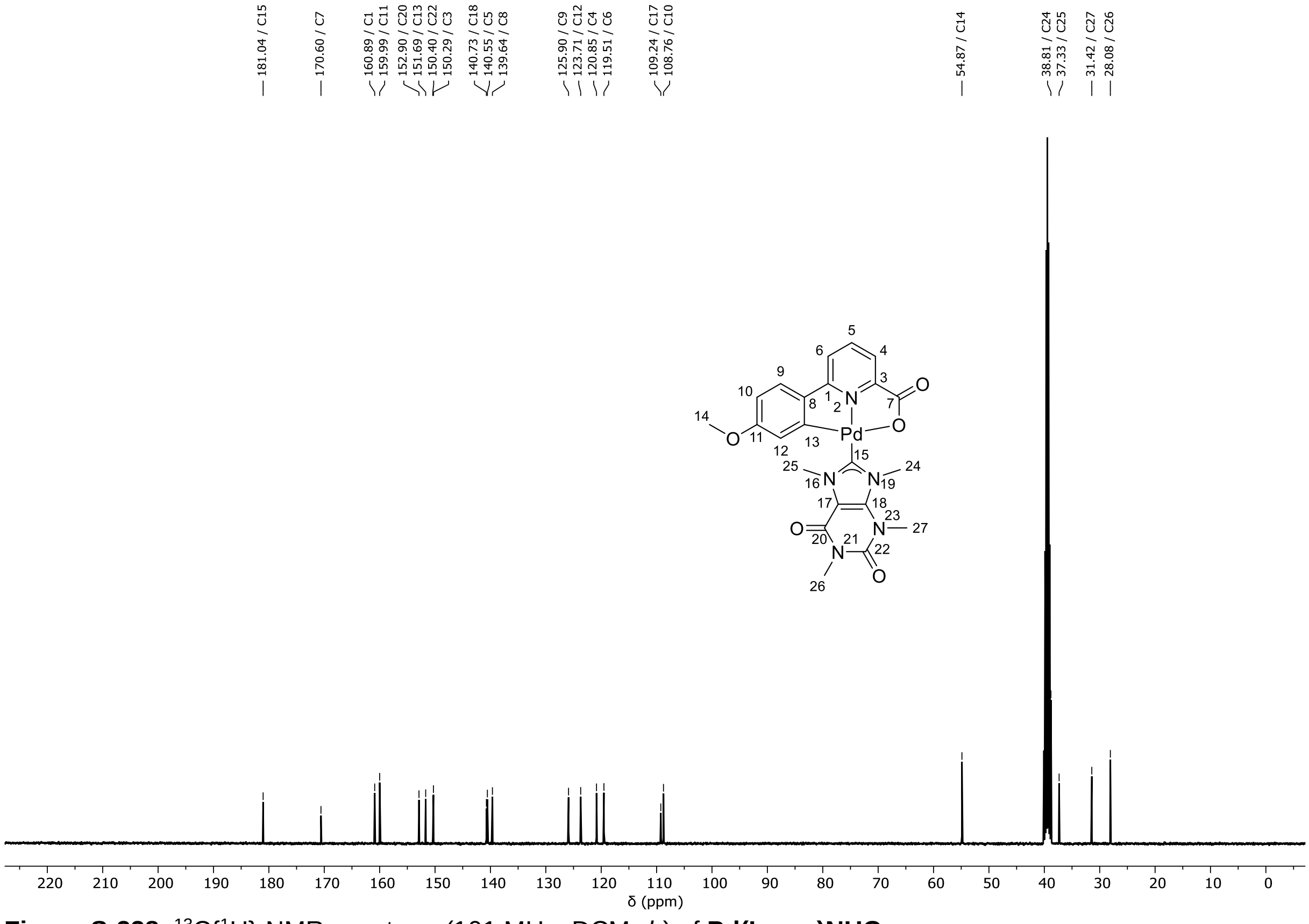

Figure S-228. ^{13}C{^{1}H}-NMR spectrum (101 MHz, DCM-d_2) of **Pd(L$_{mOMe}$)NHC**.

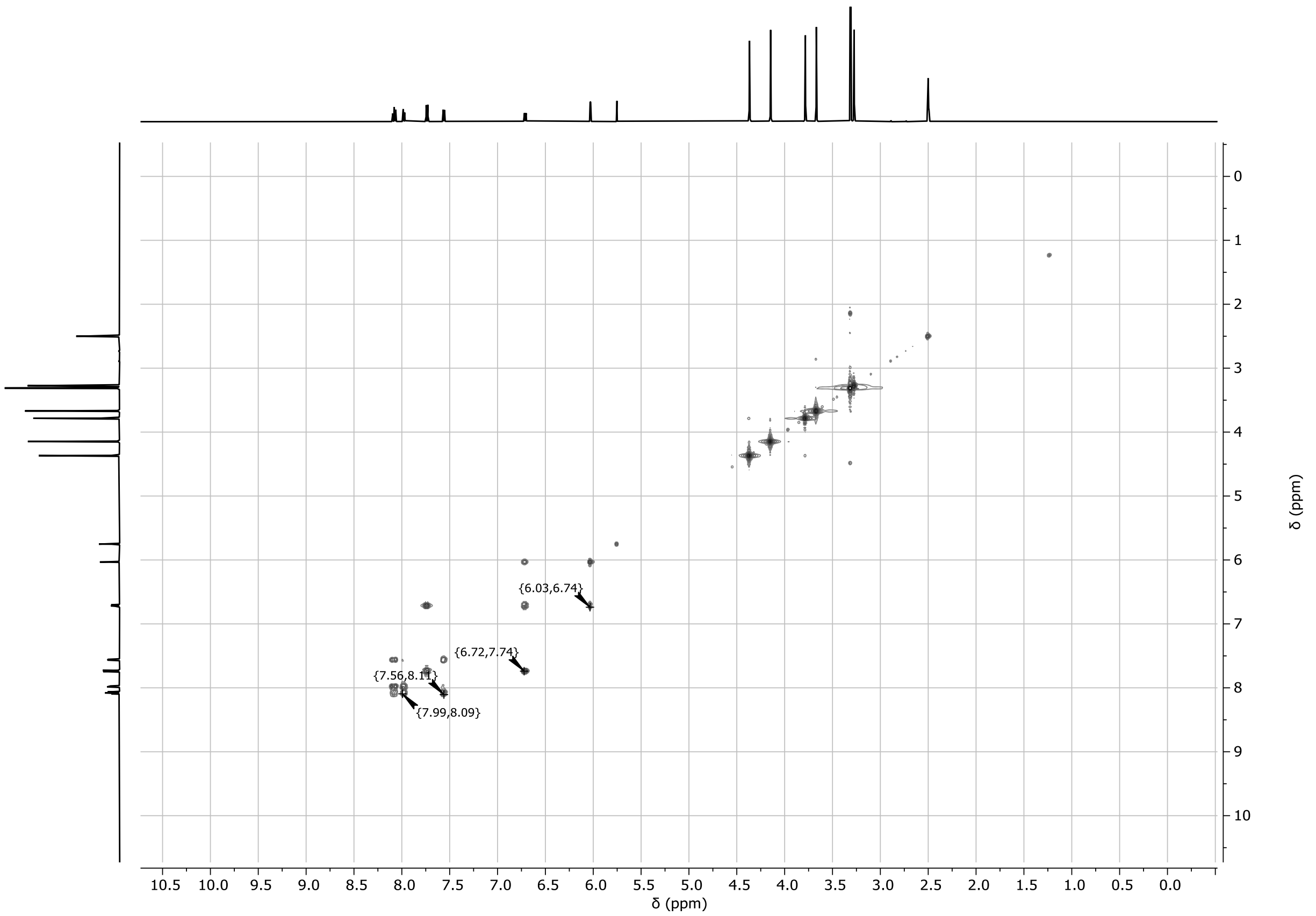

Figure S-229. HH-COSY-NMR spectrum (400 MHz (^{1}H), DCM-d_2) of **Pd(L$_{mOMe}$)NHC**.

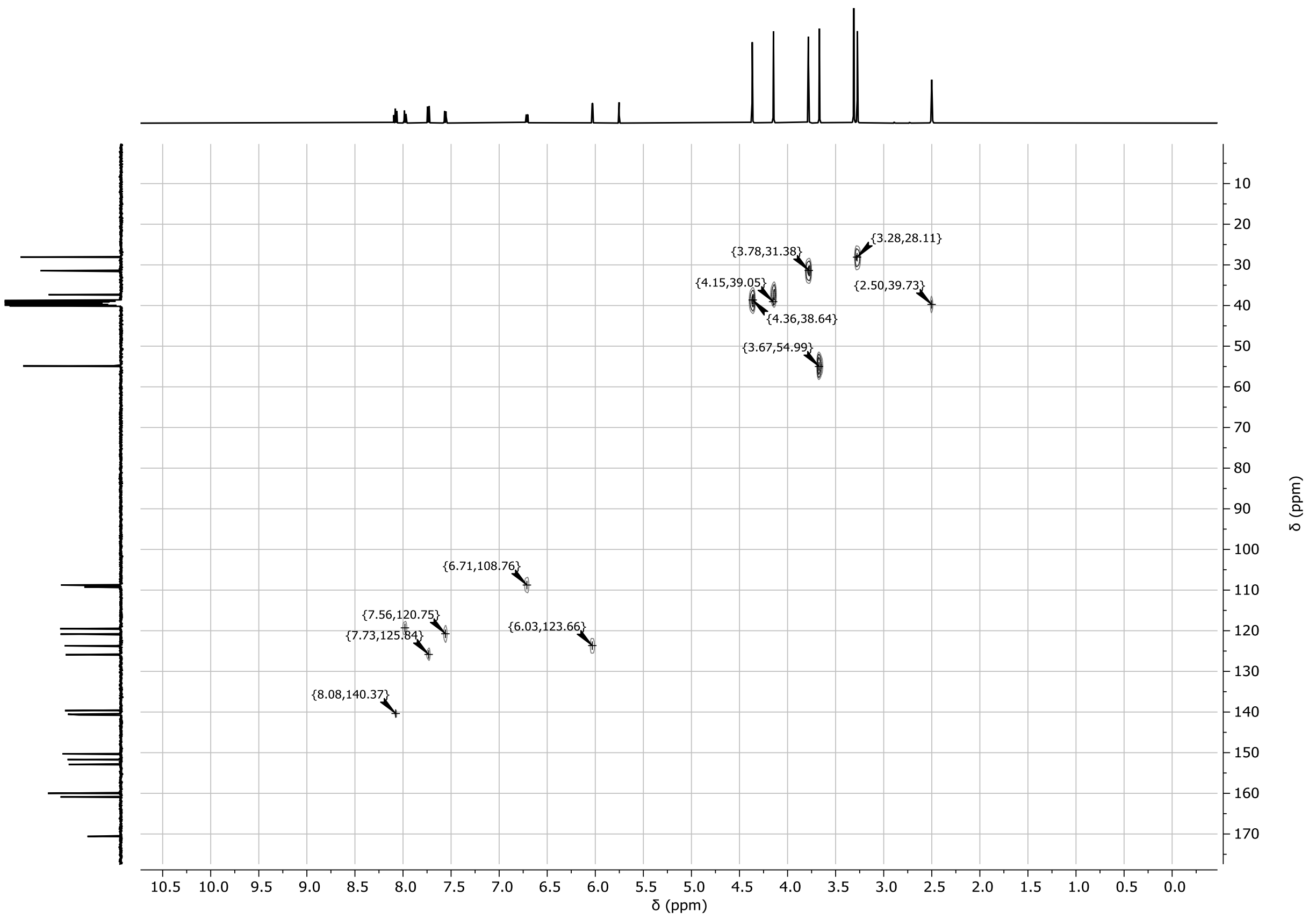

Figure S-230. HC-HSQC-NMR spectrum (101 MHz (^{13}C), 400 MHz (^{1}H), DCM-d_2) of **Pd(L$_{mOMe}$)NHC**.

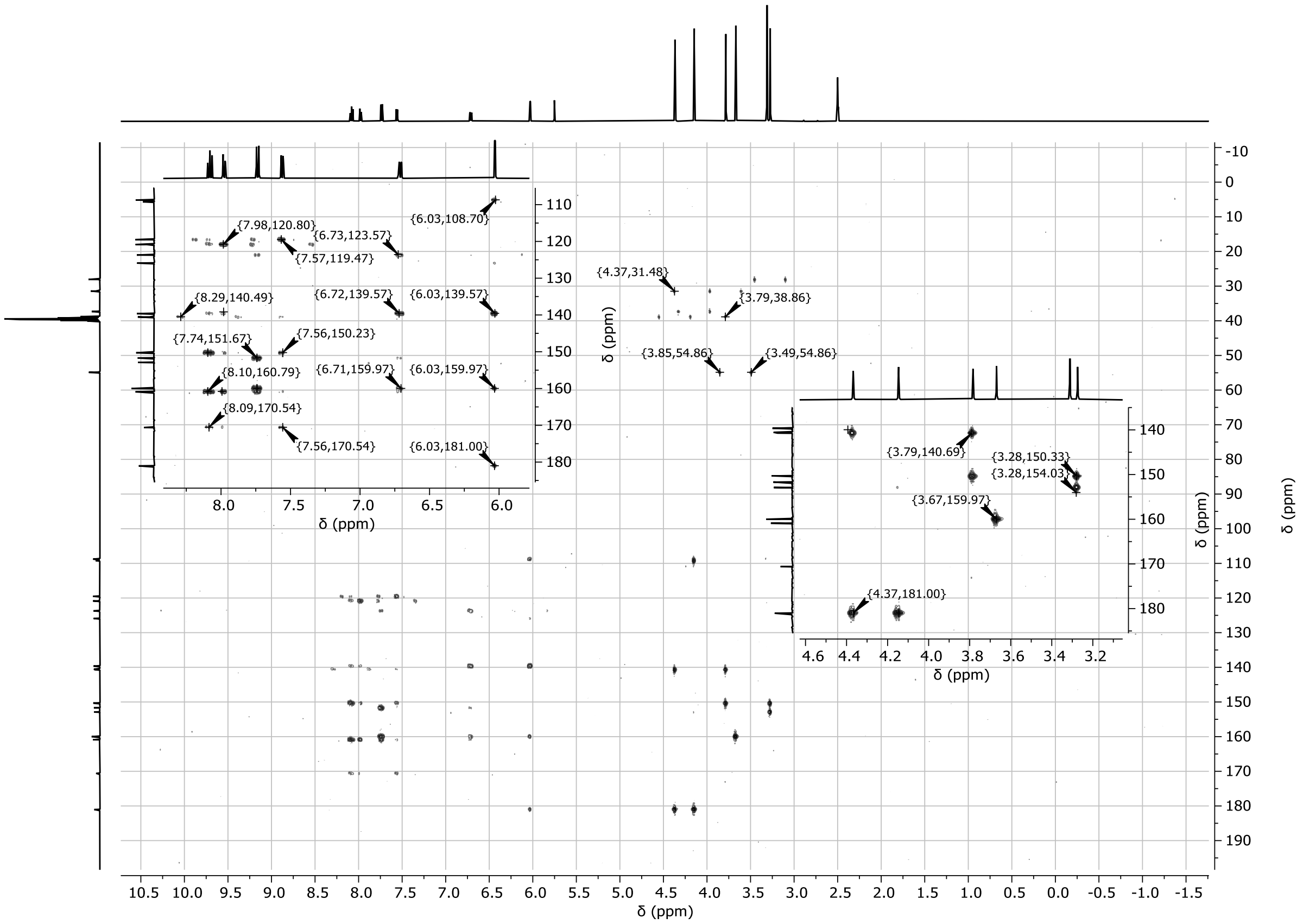

Figure S-231. HC-HMBC-NMR spectrum (101 MHz (^{13}C), 400 MHz (^{1}H), DCM-d_2) of **Pd(L_{mOMe})NHC**.

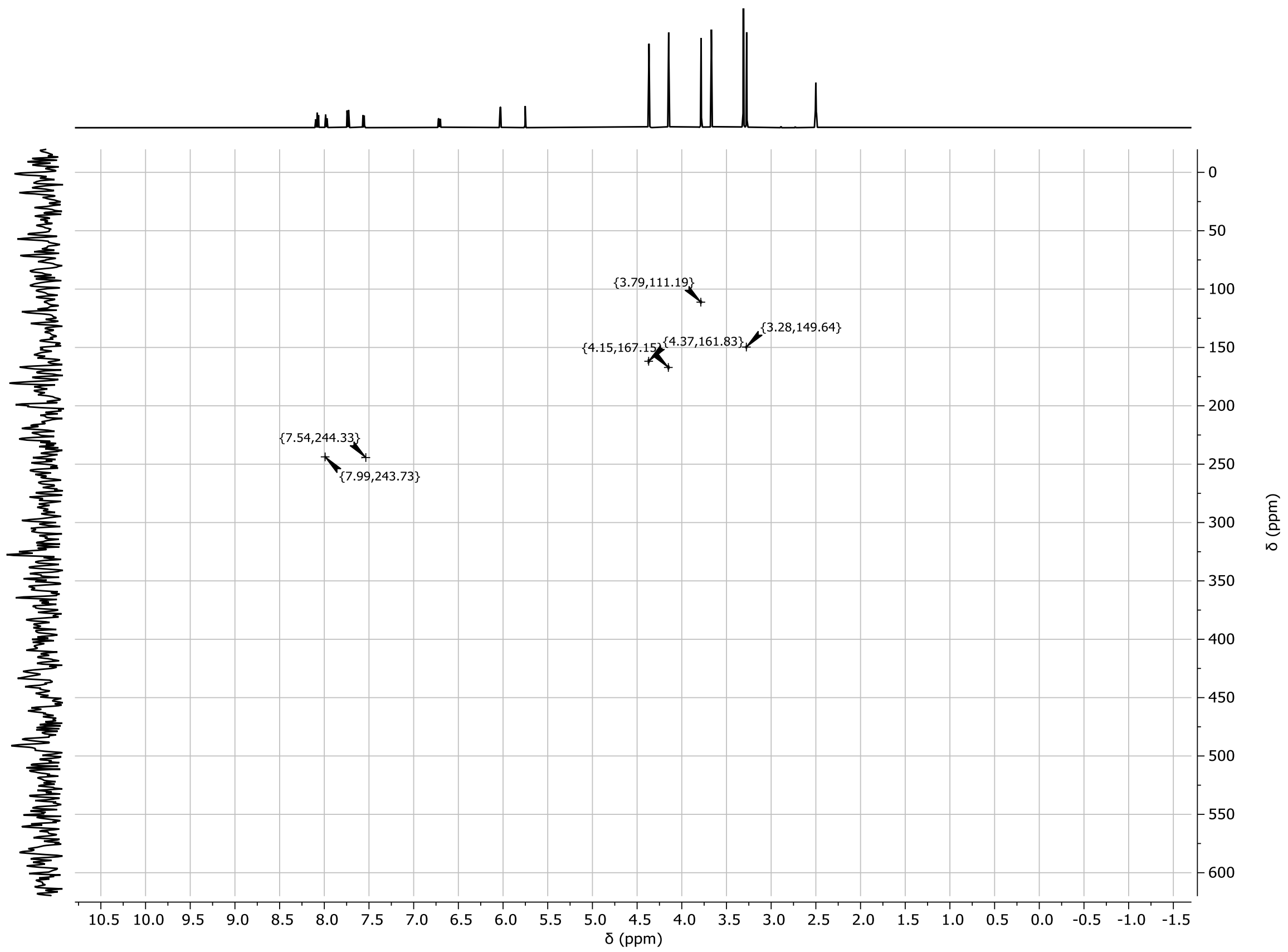

Figure S-232. HN-HMBC-NMR spectrum (41 MHz (^{13}C), 400 MHz (^{1}H), DCM-d_2) of **Pd(L_{mOMe})NHC**.

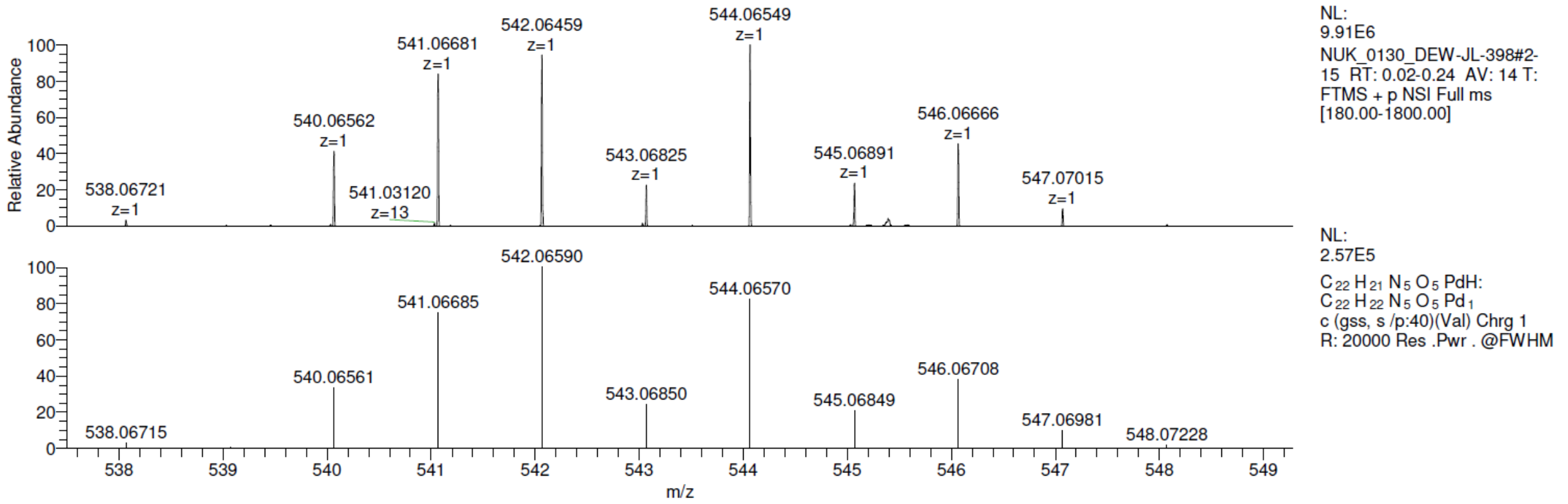

Figure S-233. Mass spectrum of **Pd(L$_{mOMe}$)NHC** (MeOH). Additional simulation of the [**Pd(L$_{mOMe}$)NHC**+H]$^+$ adduct.

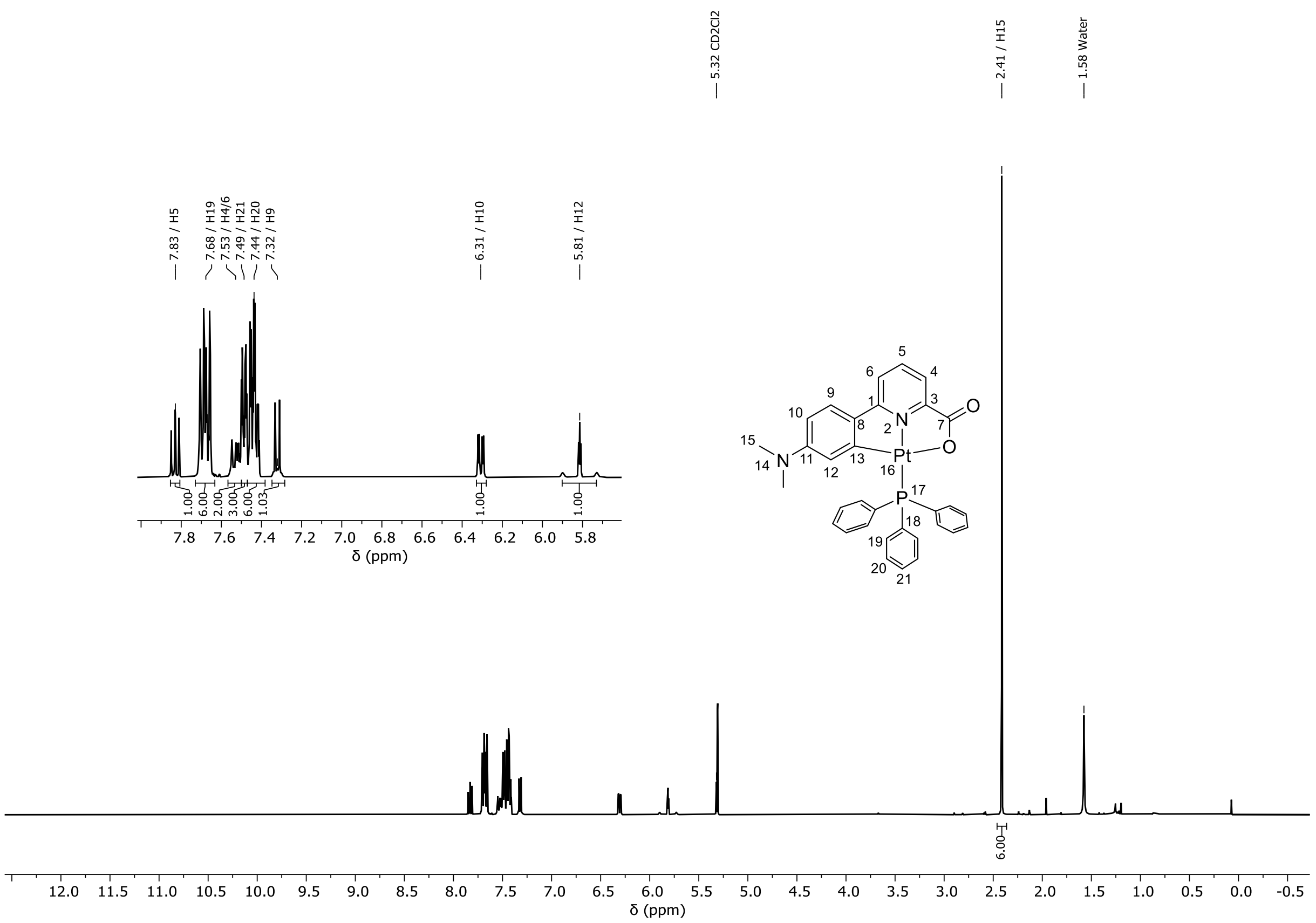

Figure S-234. ^{1}H-NMR spectrum (400 MHz, DCM-d_2) of **Pt(L$_{mDMA}$)PPh$_3$.**

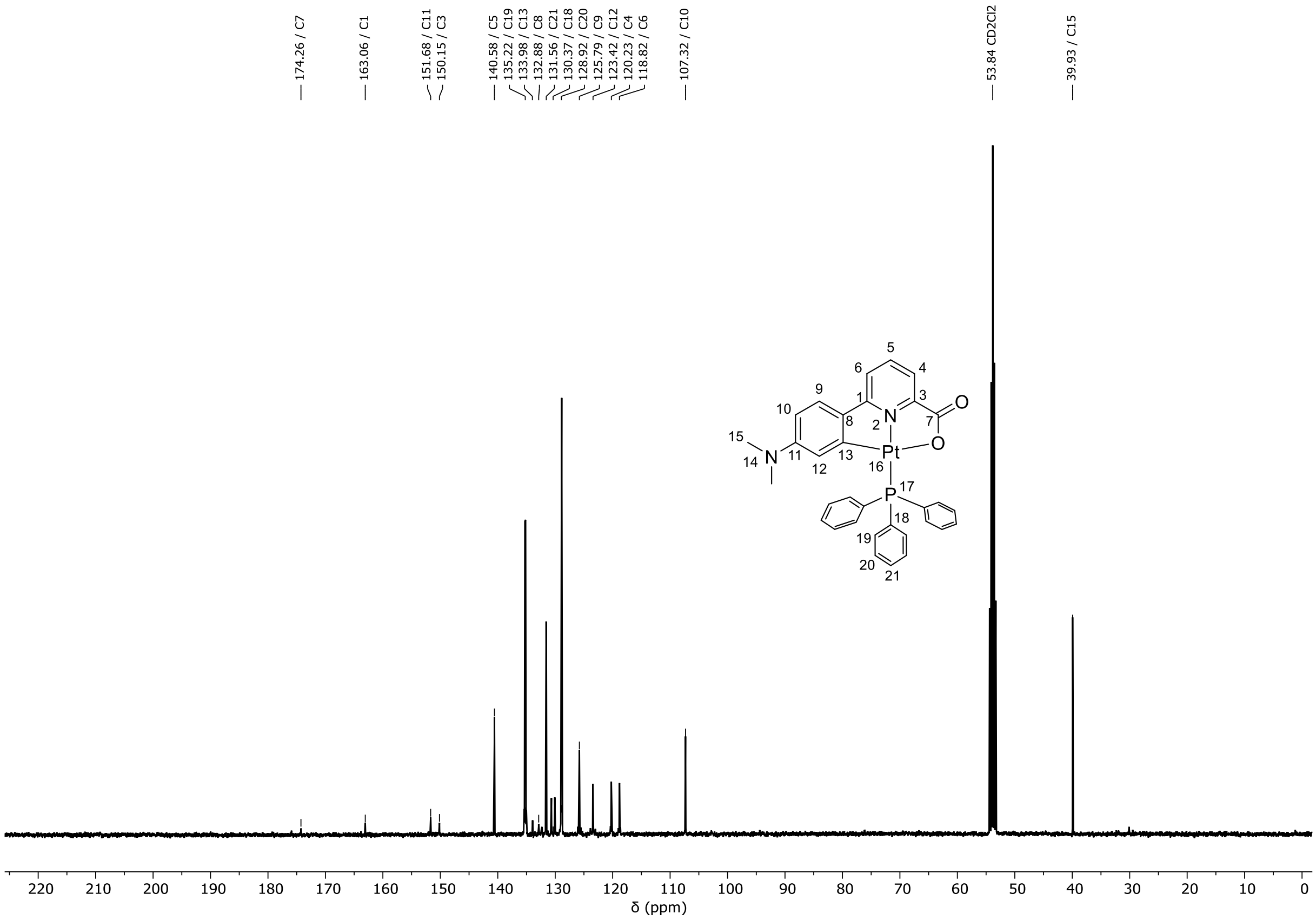

Figure S-235. $^{13}C\{^{1}H\}$-NMR spectrum (101 MHz, DCM-d_2) of **Pt(L$_{mDMA}$)PPh$_3$**.

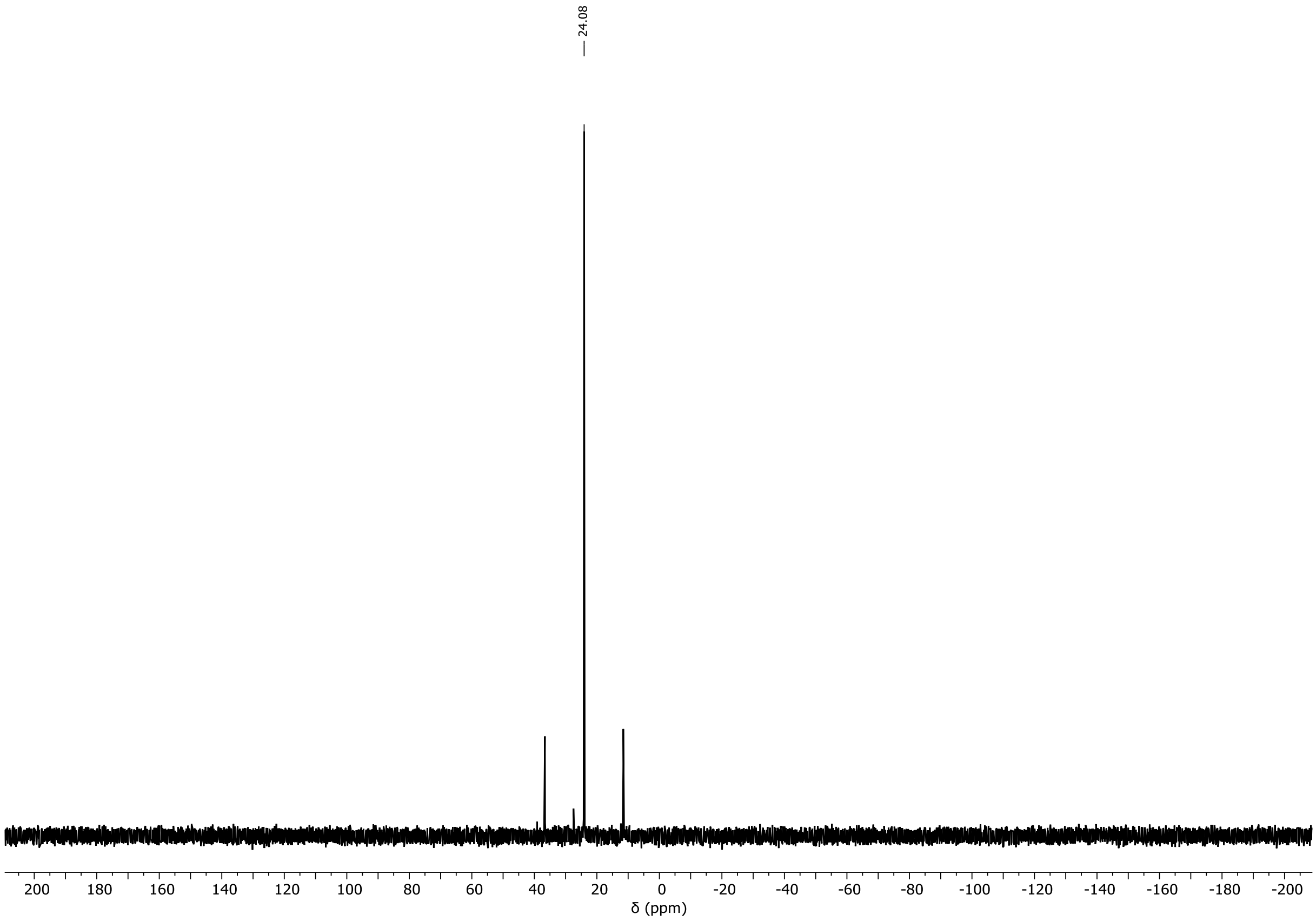

Figure S-236. $^{31}P\{^{1}H\}$-NMR spectrum (162 MHz, DCM-d_2) of **Pt(L$_{mDMA}$)PPh$_3$**.

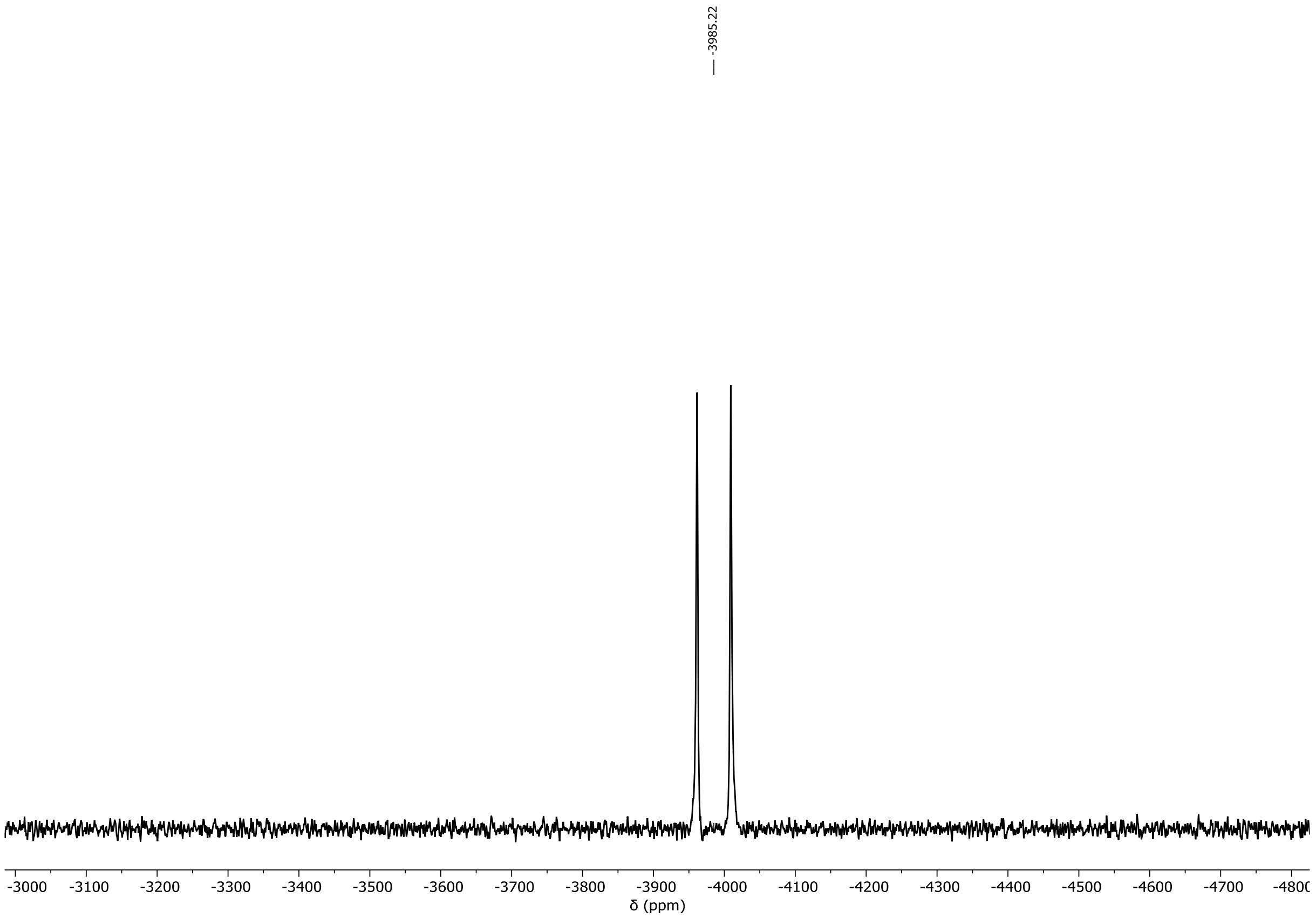

Figure S-237. ^{195}Pt-NMR spectrum (86 MHz, DCM-d_2) of **Pt(L$_{mDMA}$)PPh$_3$**.

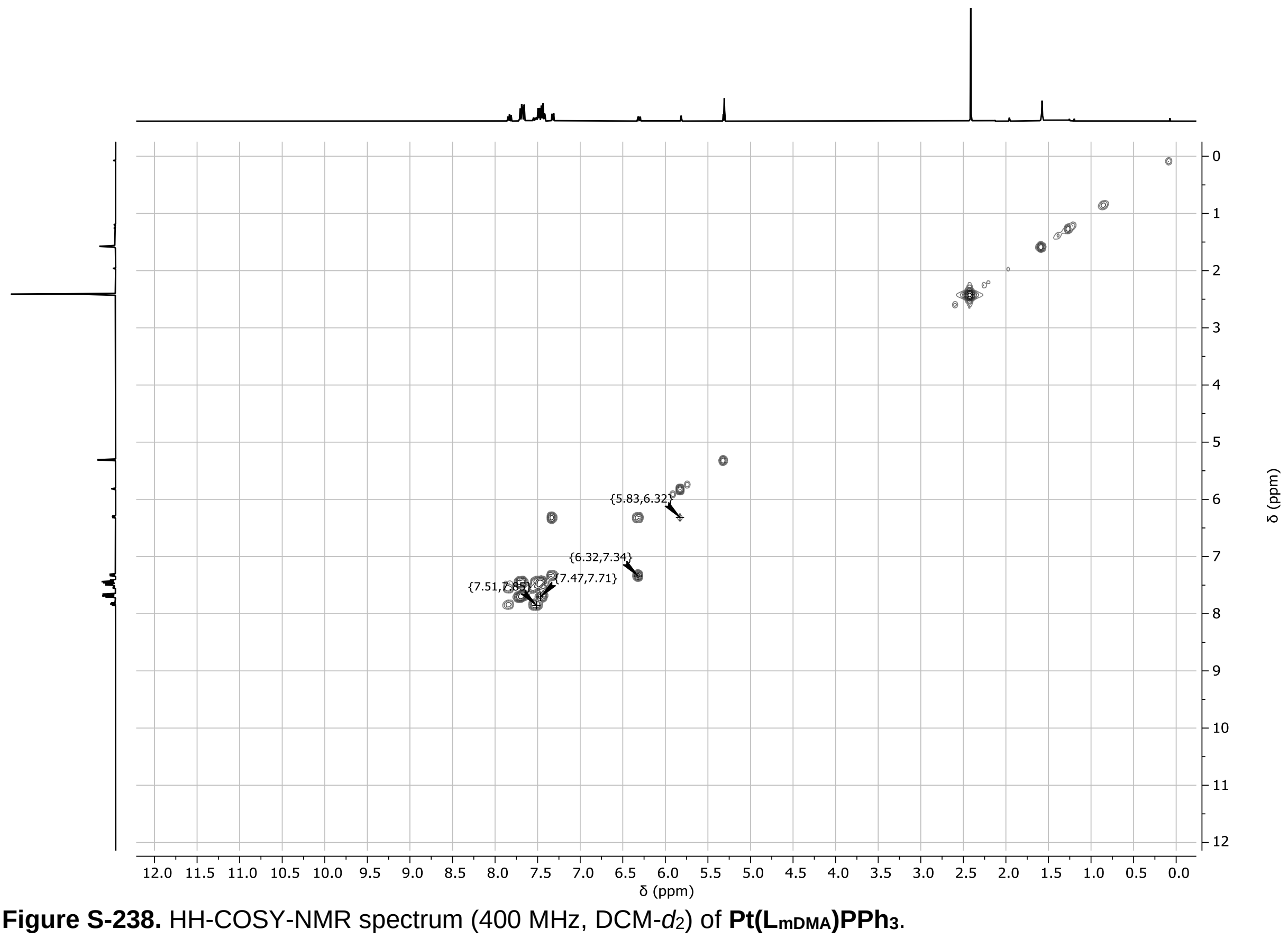

Figure S-238. HH-COSY-NMR spectrum (400 MHz, DCM-d_2) of **Pt(L$_{mDMA}$)PPh$_3$**.

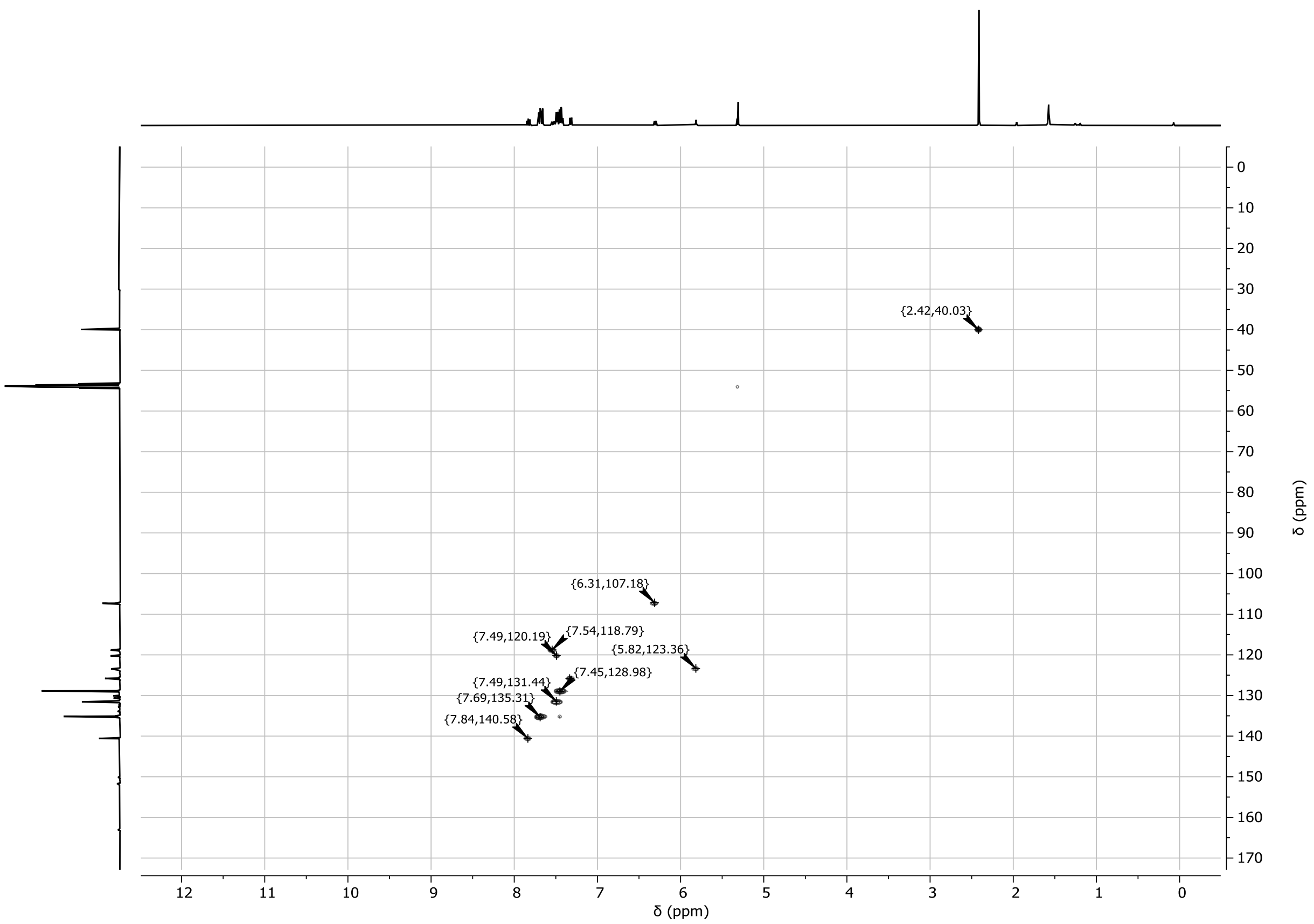

Figure S-239. HC-HSQC-NMR spectrum (101 MHz (^{13}C), 400 MHz (^{1}H), DCM-d_2) of **Pt(L_{mDMA})PPh_3**.

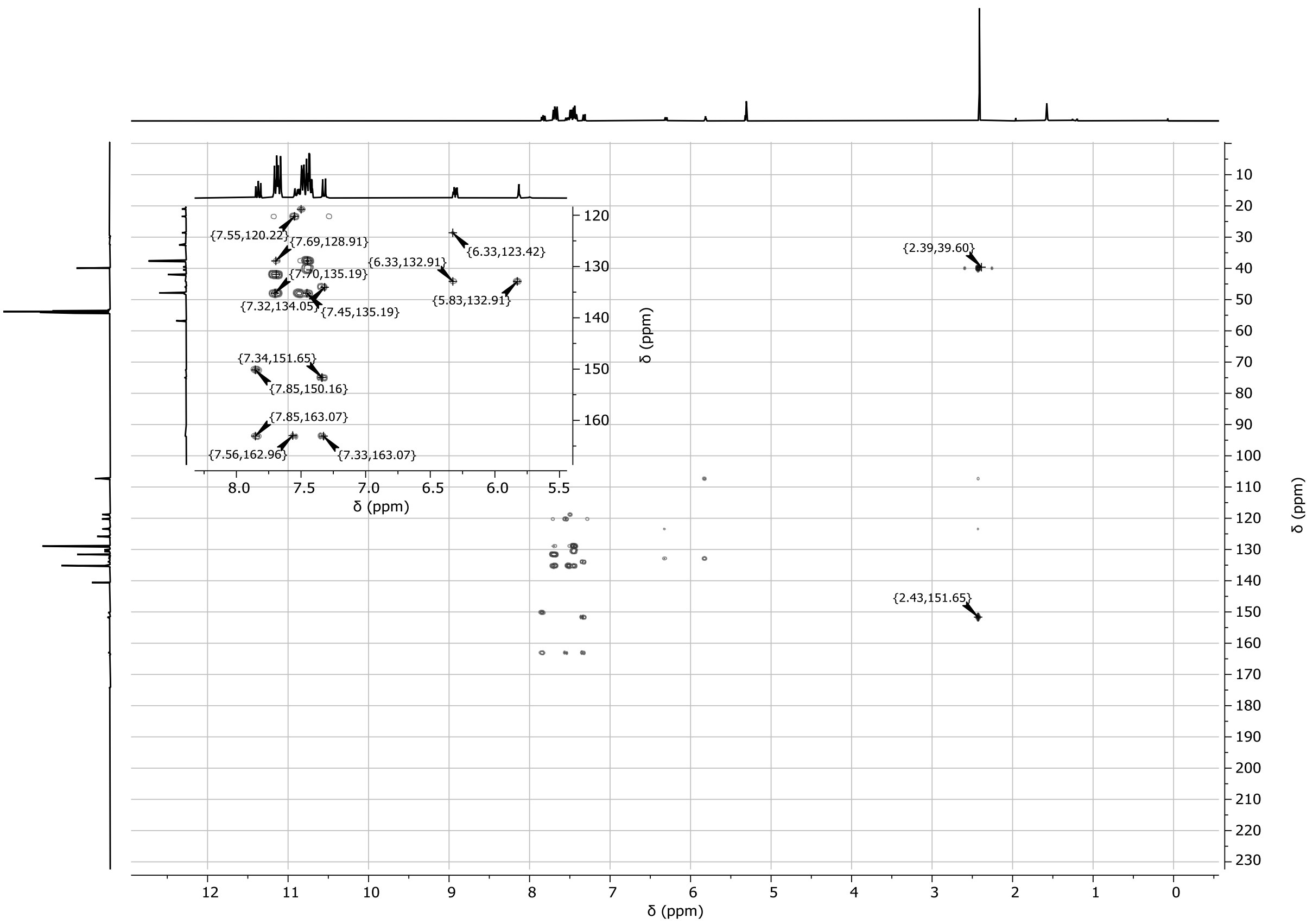

Figure S-240. HC-HMBC-NMR spectrum (101 MHz (^{13}C), 400 MHz (^{1}H), DCM-d_2) of **Pt(L_{mDMA})PPh_3**.

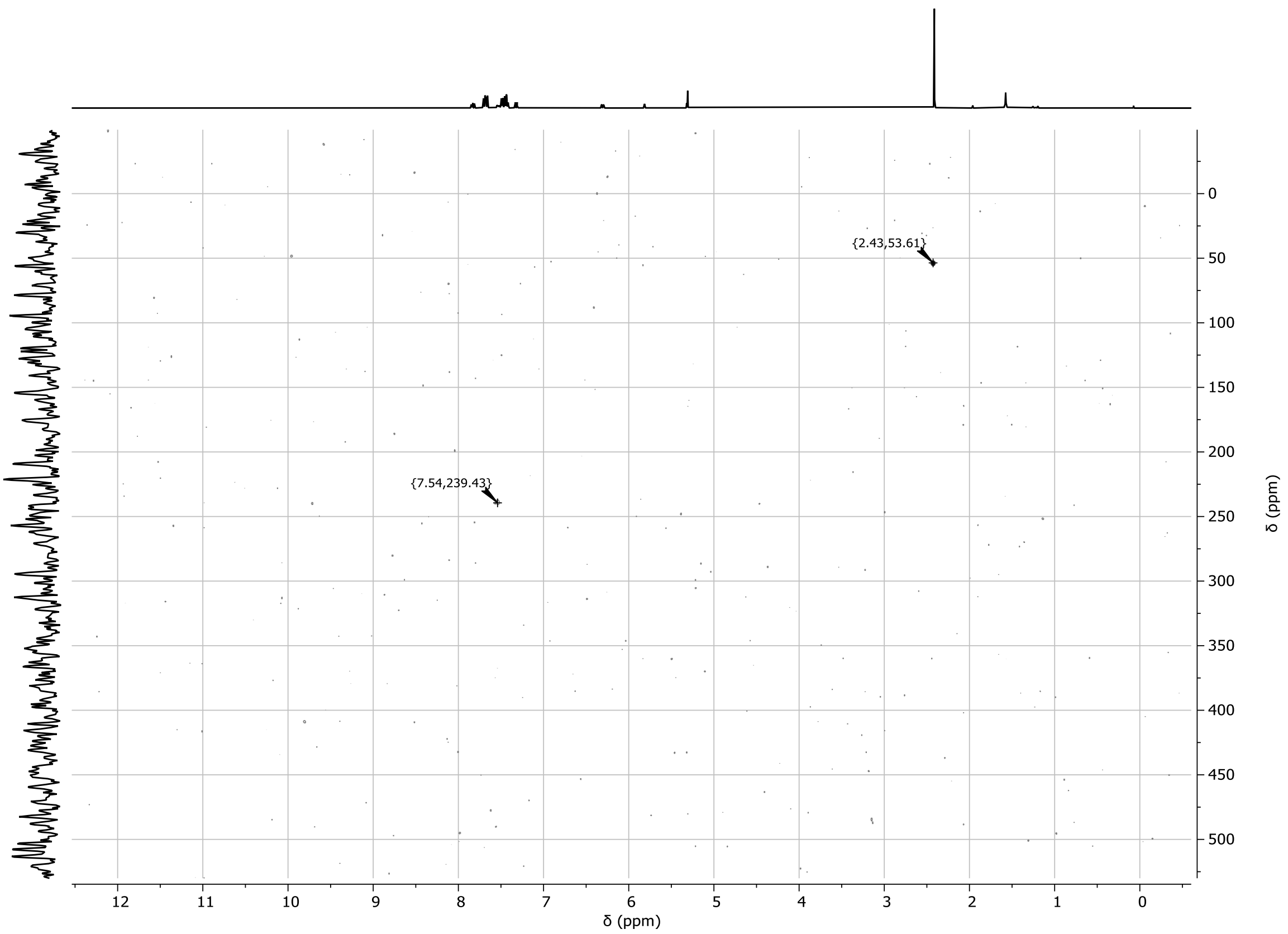

Figure S-241. HN-HMBC-NMR spectrum (41 MHz (^{15}N), 400 MHz (^{1}H), DCM-d_2) of **Pt(L$_{mDMA}$)PPh$_3$**.

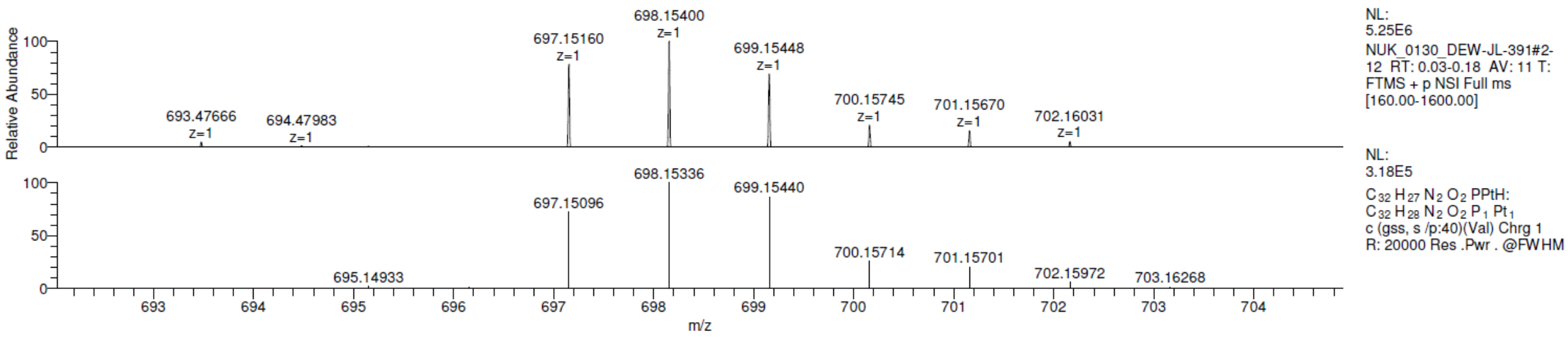

Figure S-242. Mass spectrum of **Pt(L$_{mDMA}$)PPh$_3$** (MeOH). Additional simulation of the [**Pt(L$_{mDMA}$)PPh$_3$**+H]$^+$ adduct.

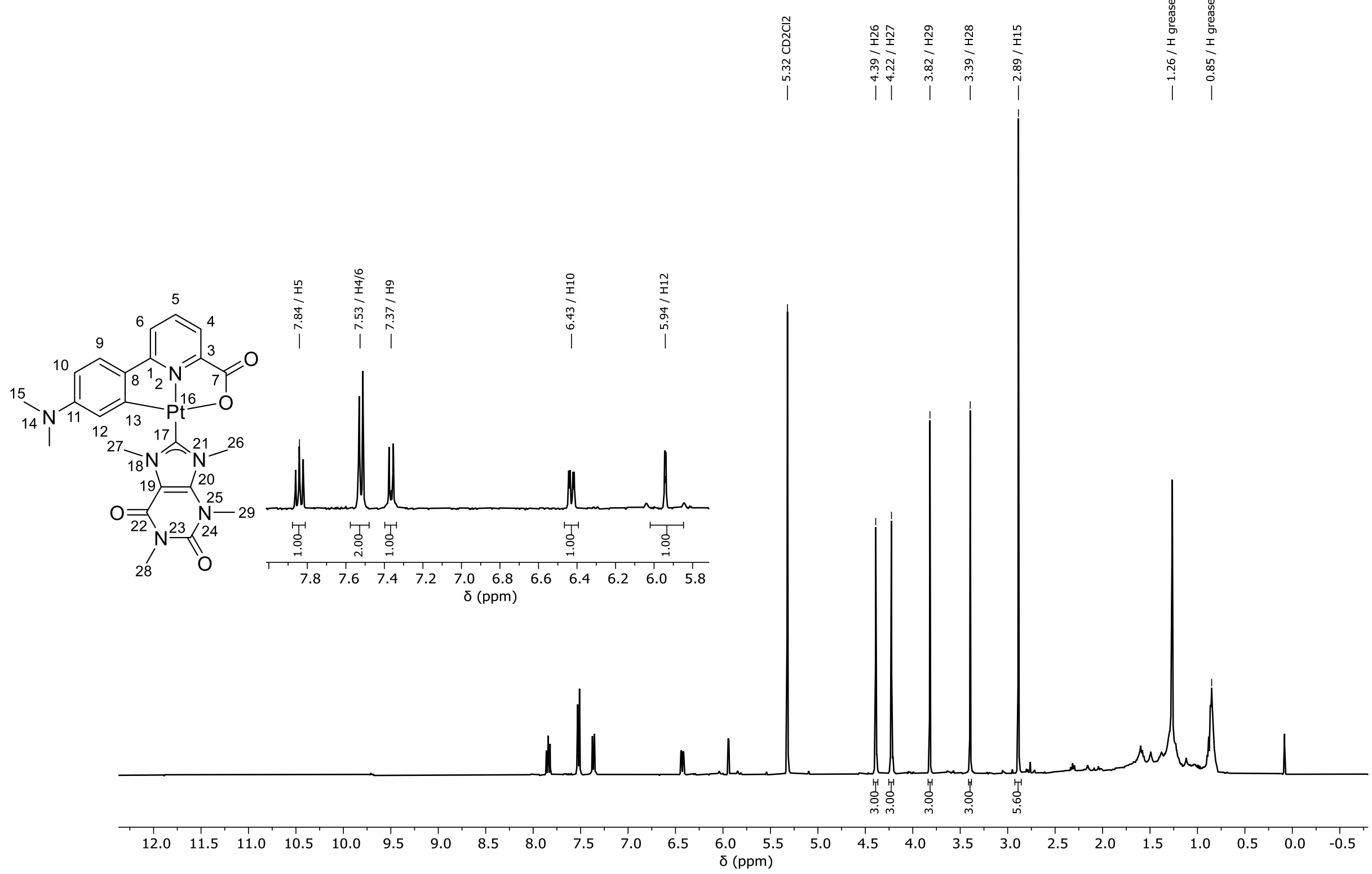

Figure S-243. ^{1}H-NMR spectrum (500 MHz, DCM-d_2) of **Pt(L$_{mDMA}$)NHC**.

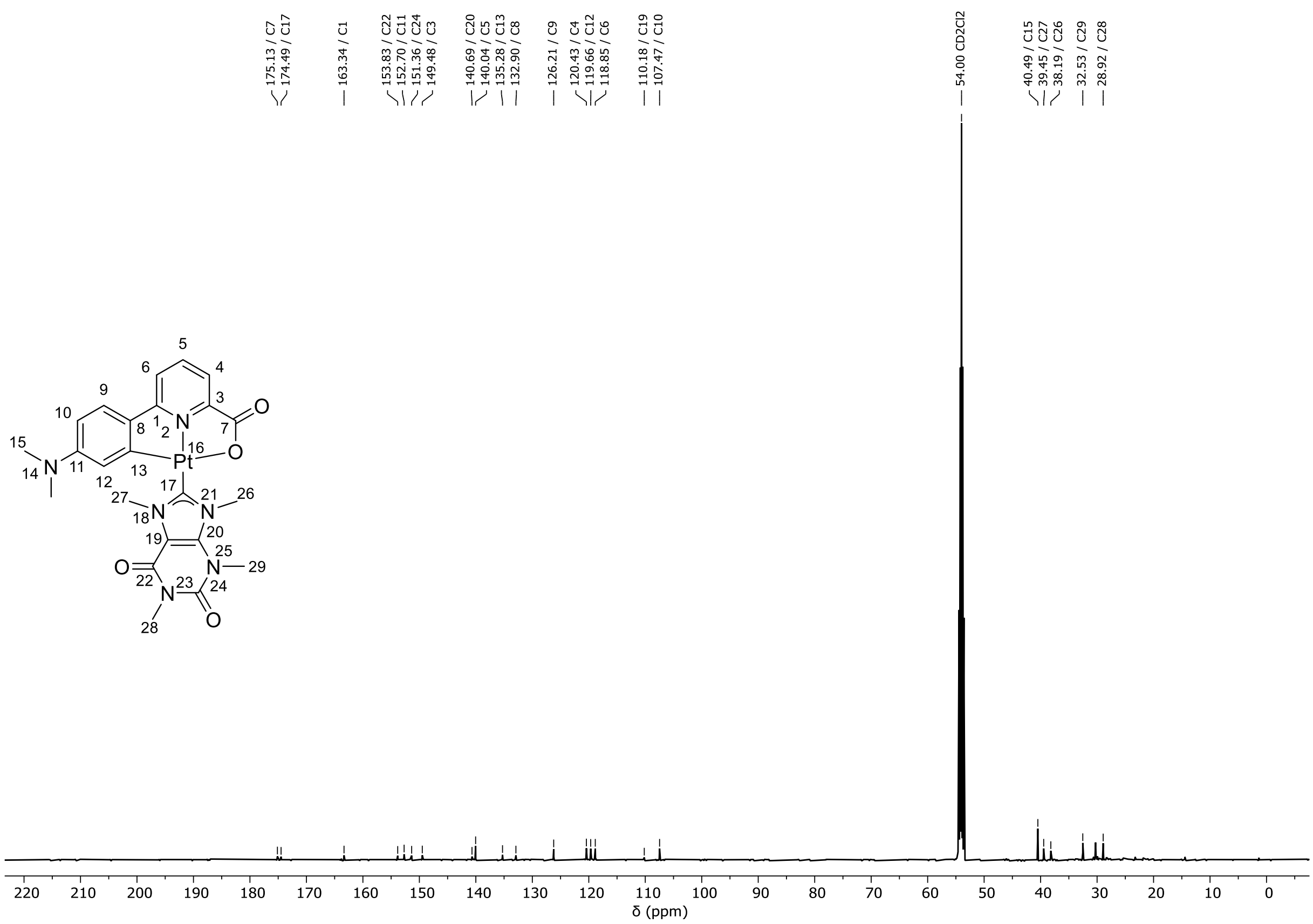

Figure S-244. ^{13}C{^{1}H}-NMR spectrum (126 MHz, DCM-d_2) of **Pt(L$_{mDMA}$)NHC**.

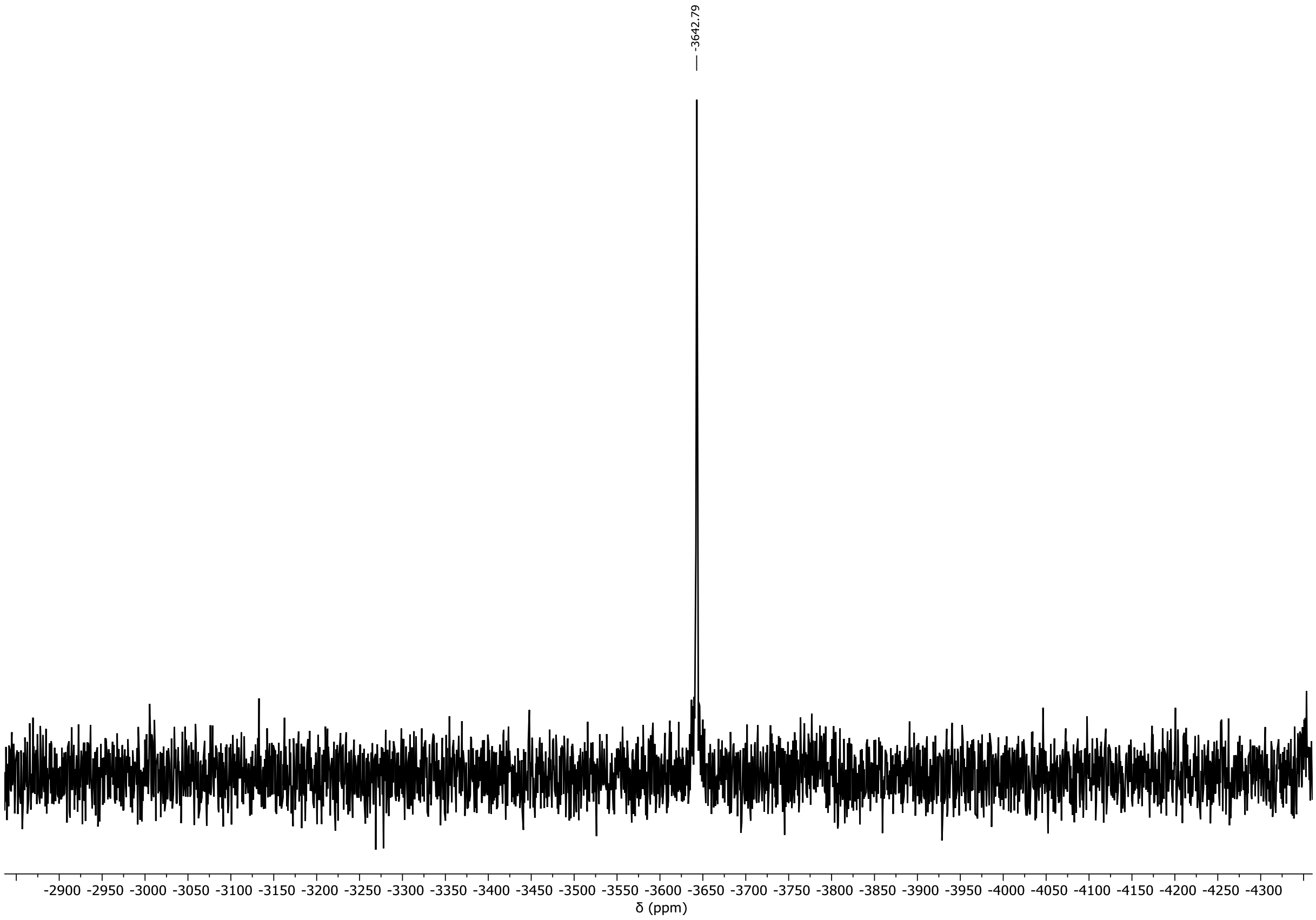

Figure S-245. ^{195}Pt-NMR spectrum (107 MHz, DCM-d_2) of **Pt(L$_{\mathbf{mDMA}}$)NHC**.

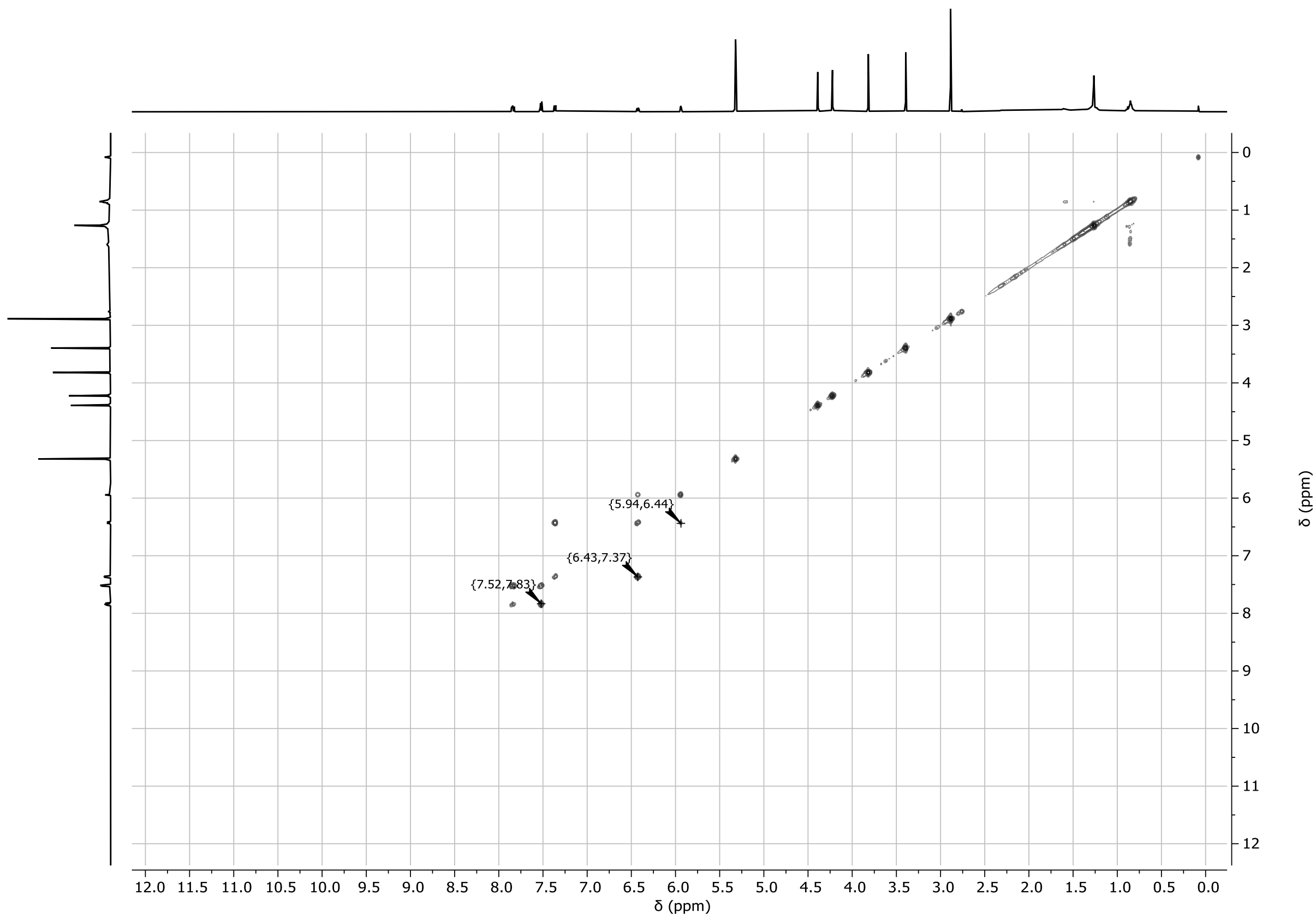

Figure S-246. HH-COSY-NMR spectrum (500 MHz, DCM-d_2) of **Pt(L$_{\mathbf{mDMA}}$)NHC**.

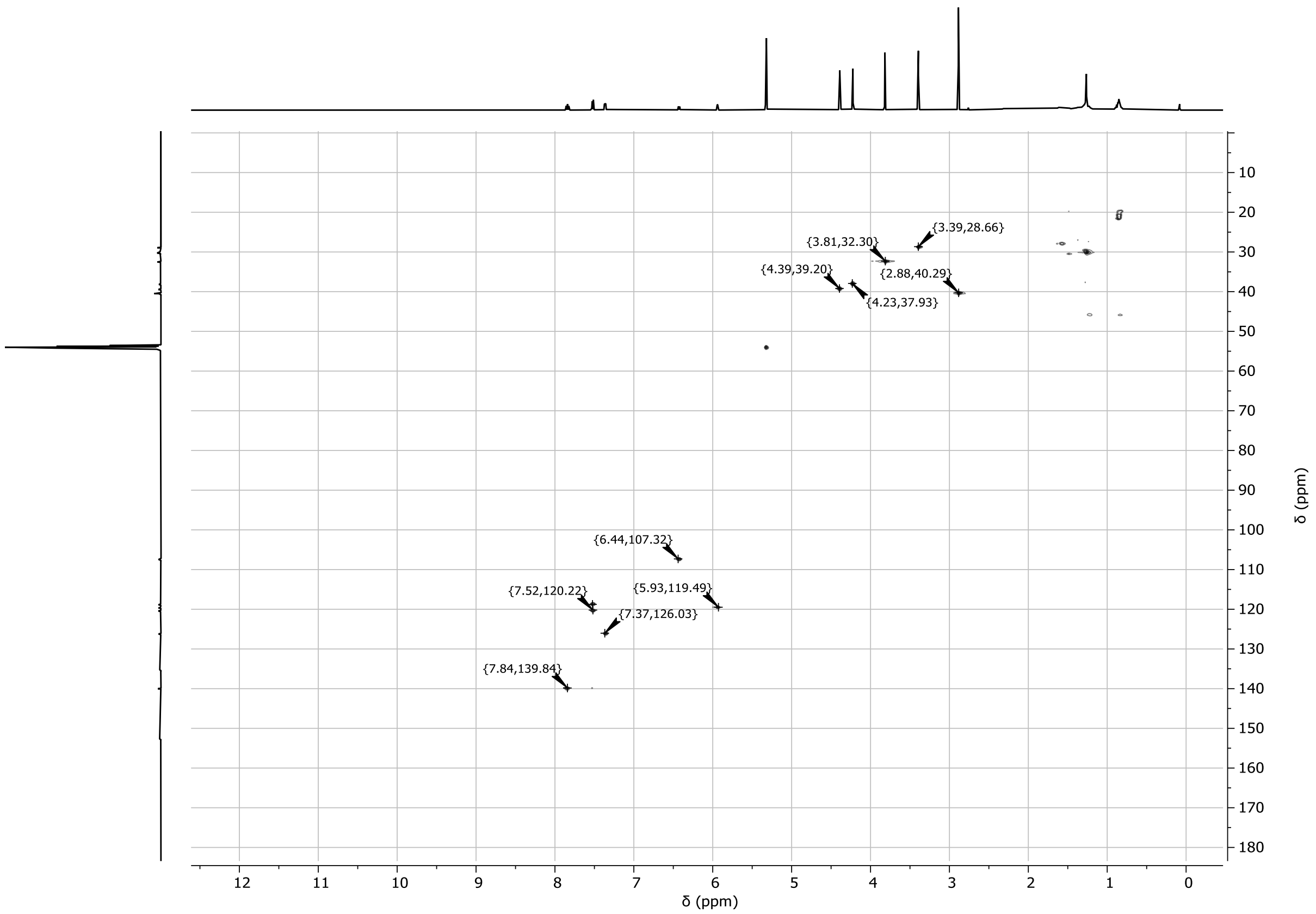

Figure S-247. HC-HSQC-NMR spectrum (126 MHz (^{13}C), 500 MHz (^{1}H), DCM-d_2) of **Pt(L$_{mDMA}$)NHC**.

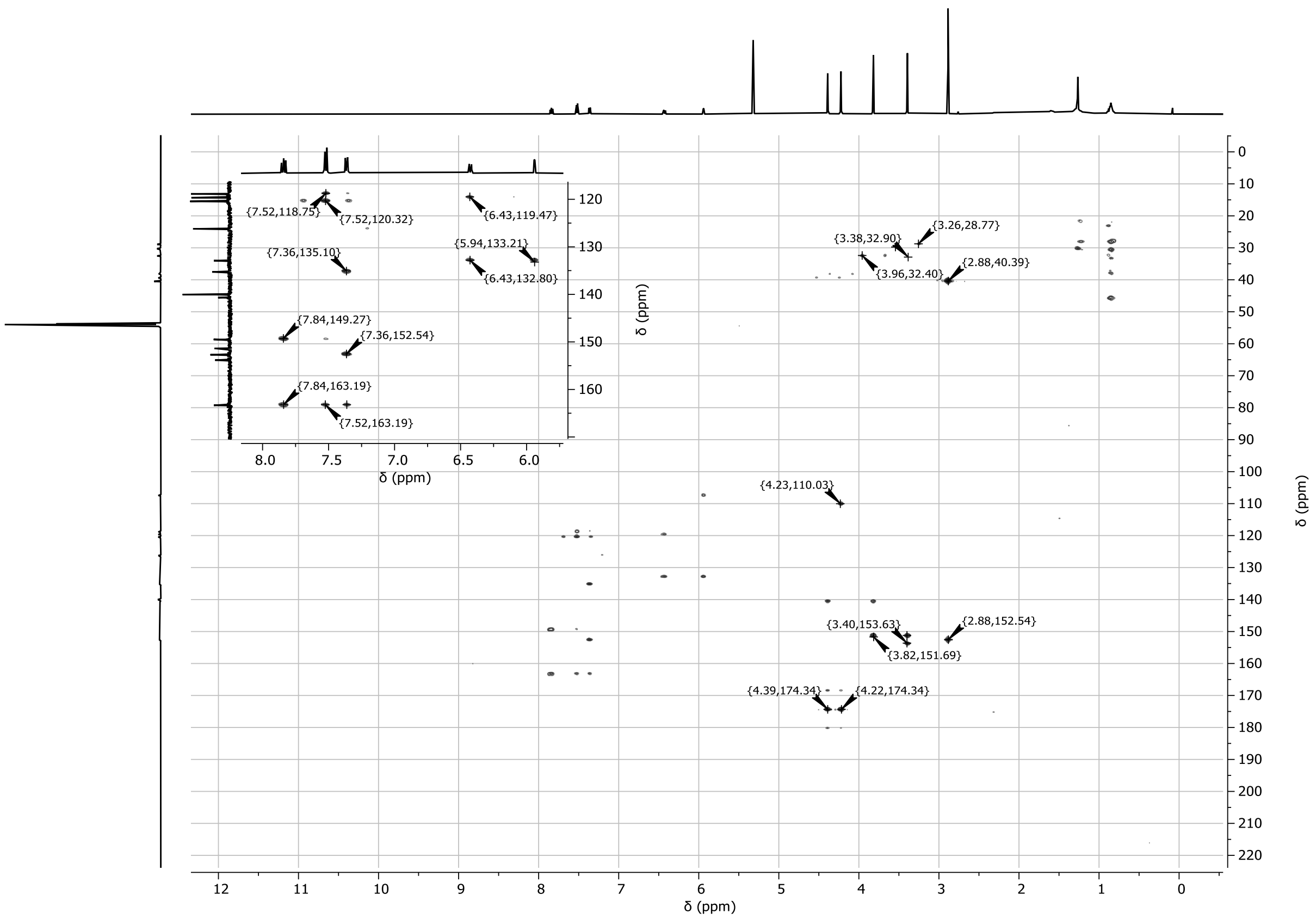

Figure S-248. HC-HMBC-NMR spectrum (126 MHz (^{13}C), 500 MHz (^{1}H), DCM-d_2) of **Pt(L$_{mDMA}$)NHC**.

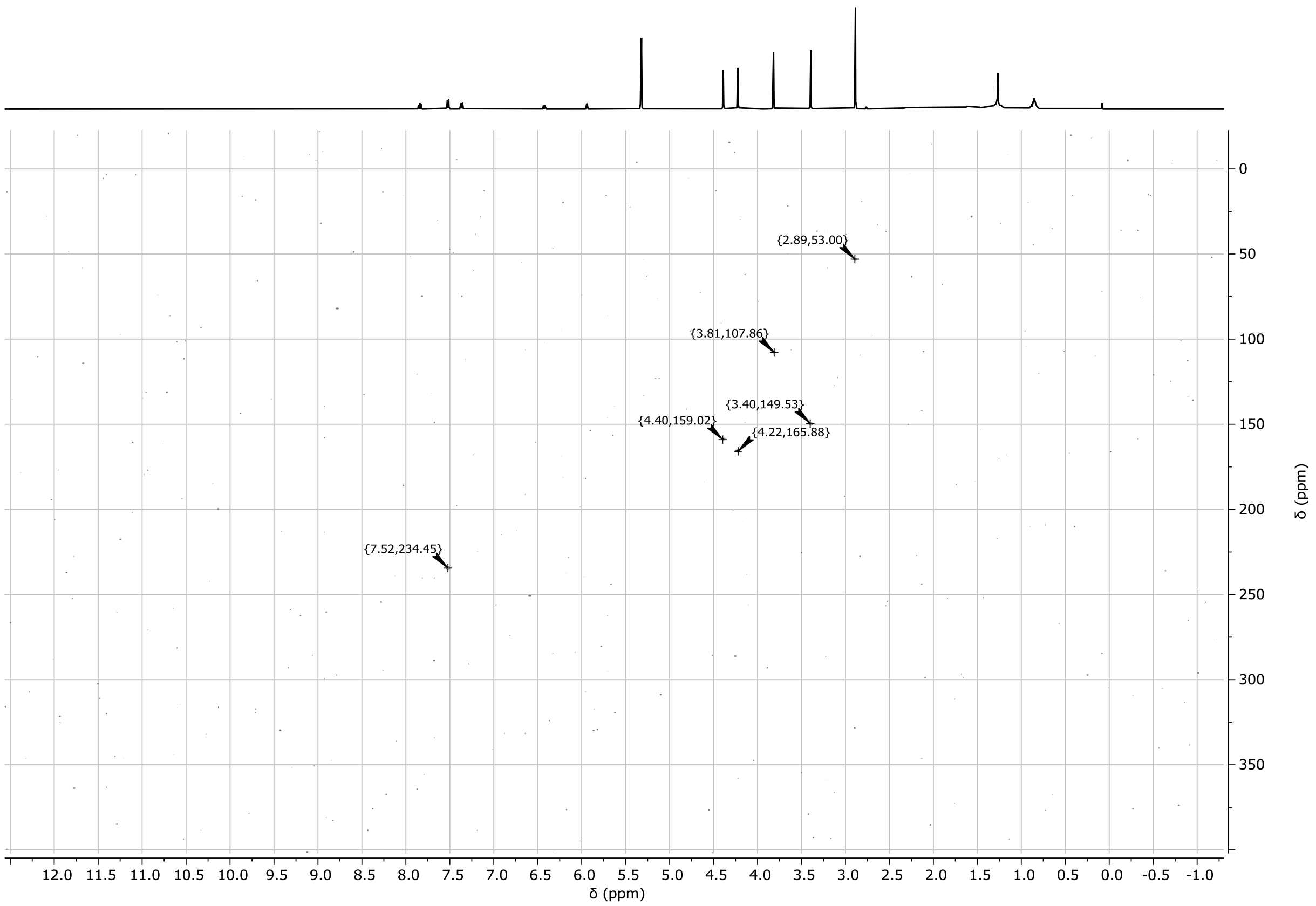

Figure S-249. HN-HMBC-NMR spectrum (51 MHz (^{15}C), 500 MHz (^{1}H), DCM-d_2) of **Pt(L$_{mDMA}$)NHC**.

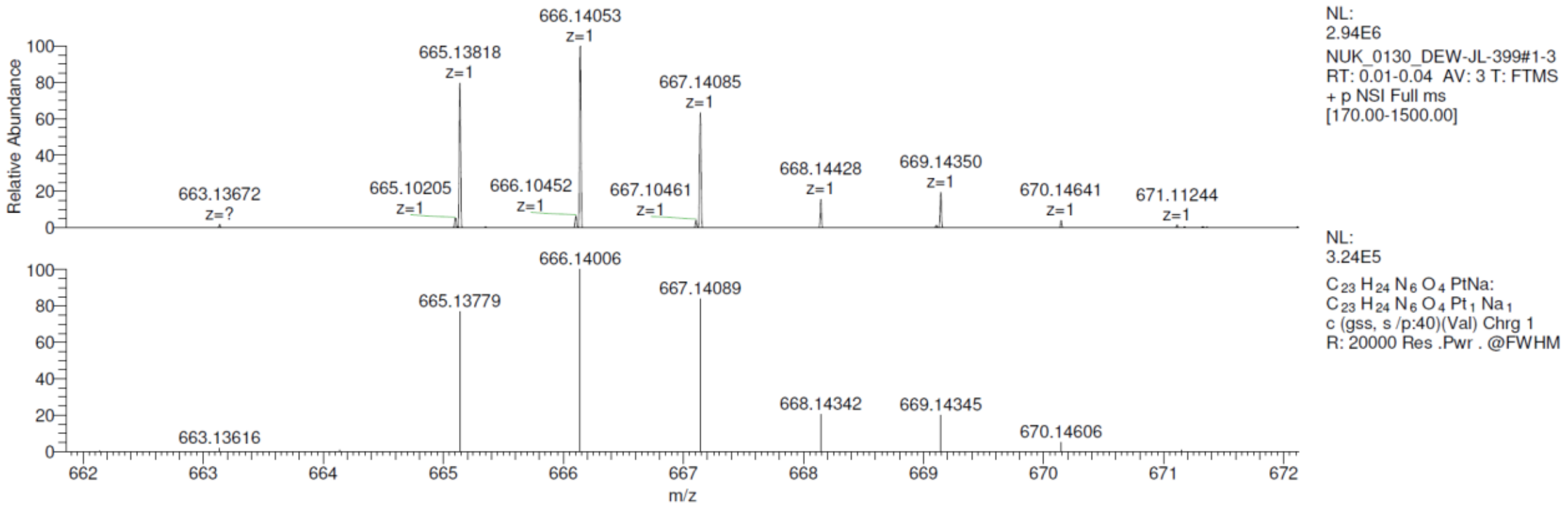

Figure S-250. Mass spectrum of **Pt(L$_{mDMA}$)NHC** (MeOH). Additional simulation of the [**Pt(L$_{mDMA}$)**NHC+Na]$^+$ adduct.

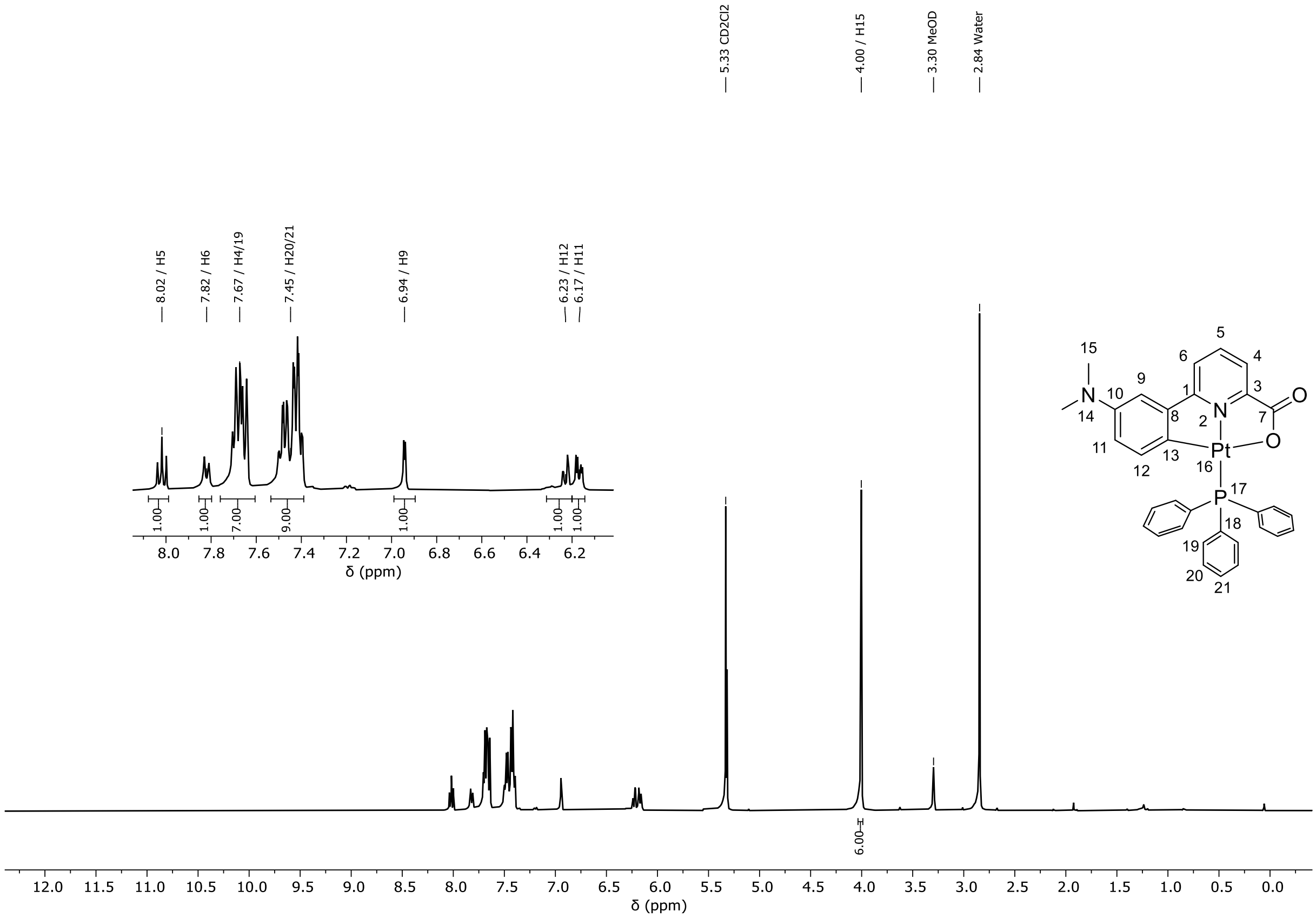

Figure S-251. ^{1}H-NMR spectrum (400 MHz, DCM-d_2) of **Pt(L$_{pDMA}$)PPh$_3$**.

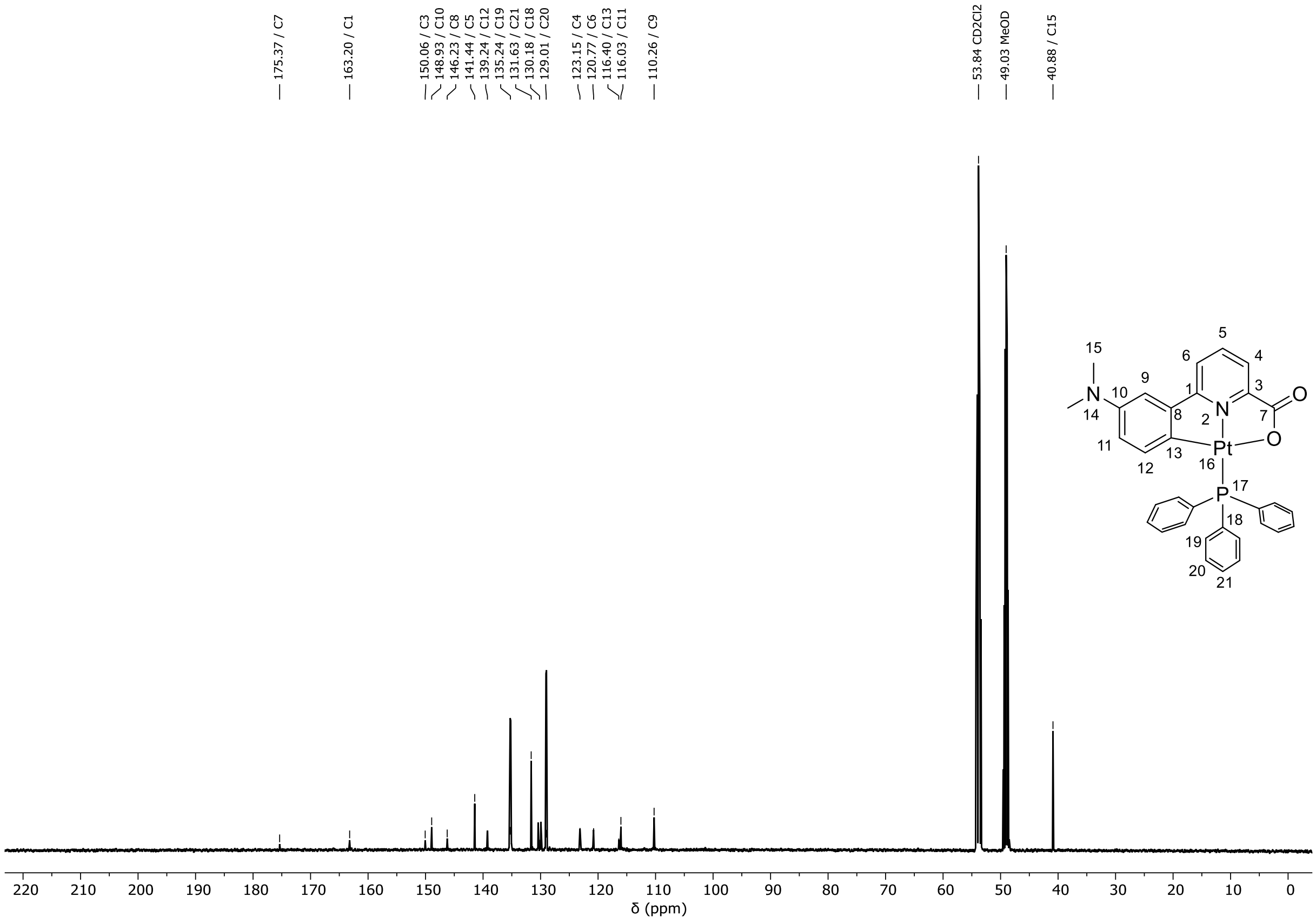

Figure S-252. ^{13}C{^{1}H}-NMR spectrum (101 MHz, DCM-d_2) of **Pt(L$_{pDMA}$)PPh$_3$**.

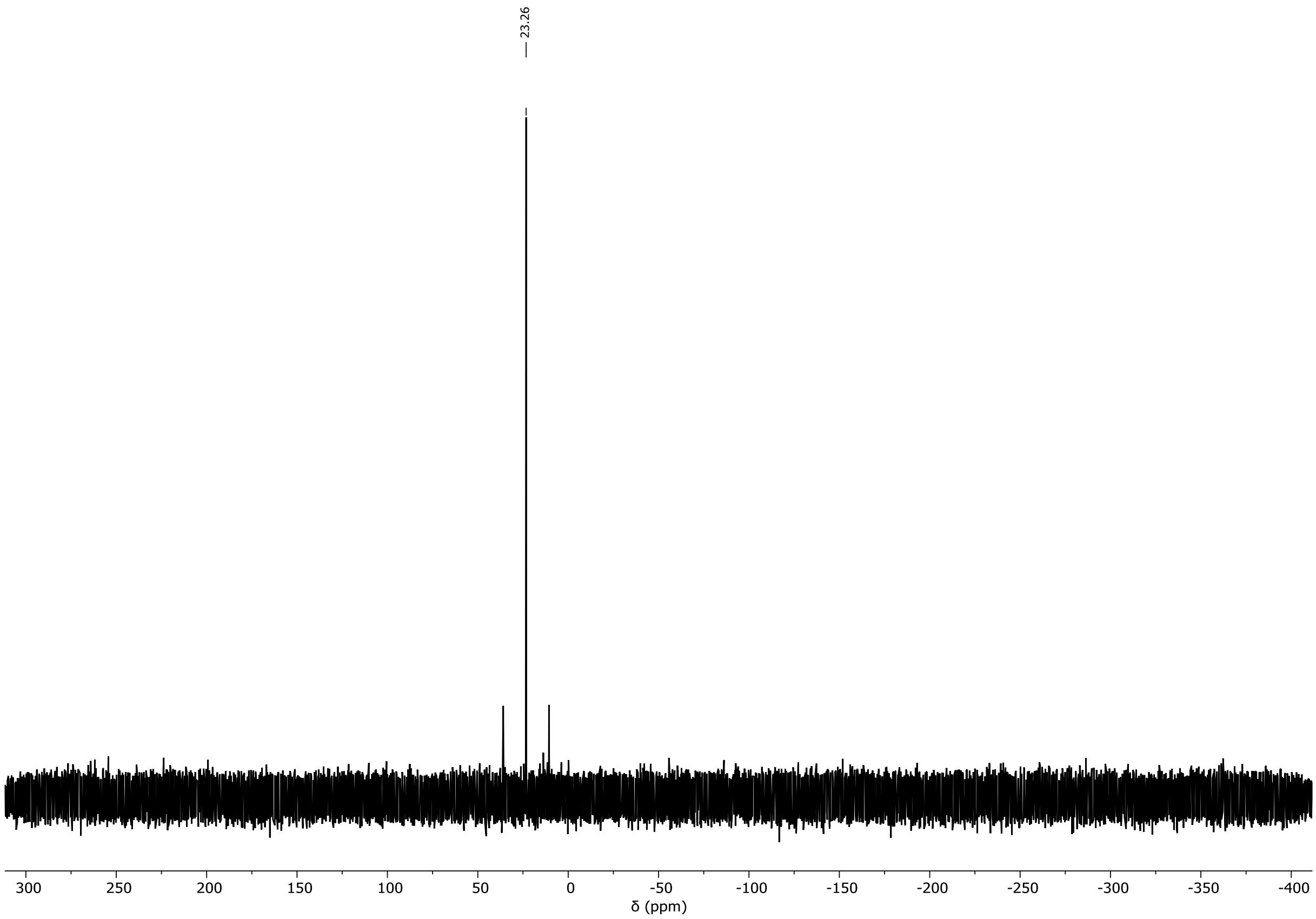

Figure S-253. $^{31}C\{^{1}H\}$-NMR spectrum (126 MHz, DCM-d_2) of **Pt(L$_{pDMA}$)PPh$_3$**.

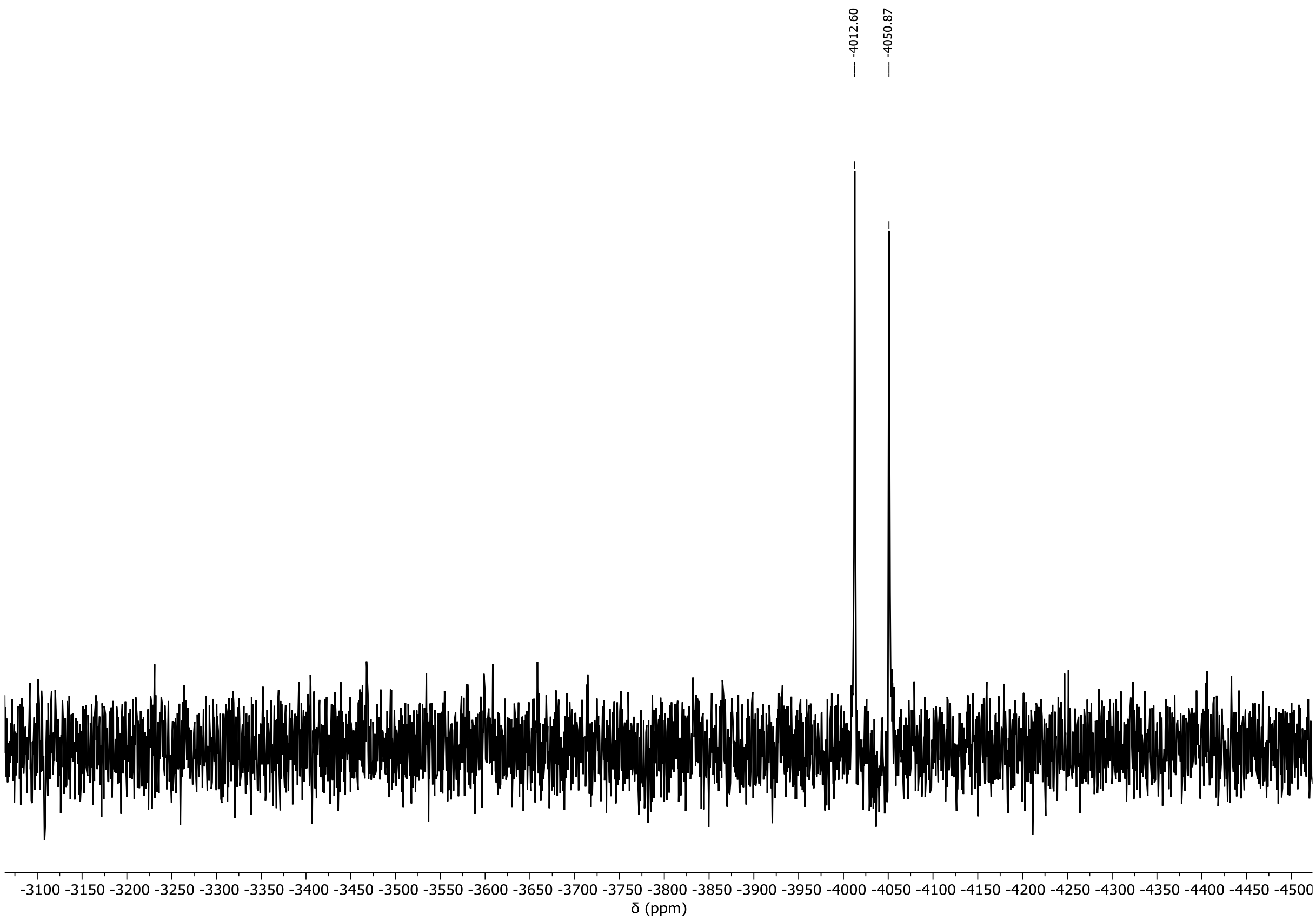

Figure S-254. ^{195}Pt-NMR spectrum (86 MHz, DCM-d_2) of **Pt(L$_{pDMA}$)PPh$_3$**.

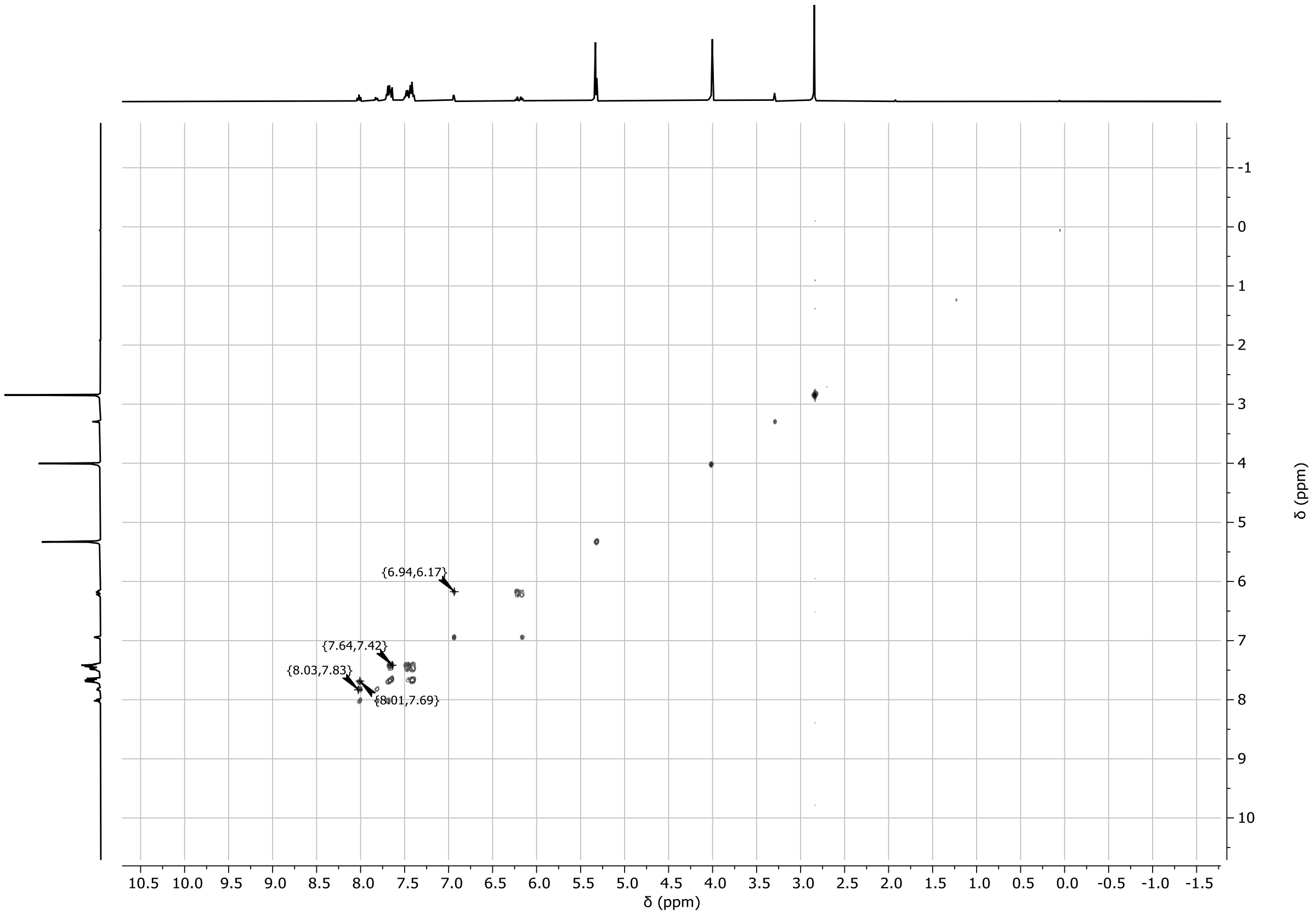

Figure S-255. HH-COSY-NMR spectrum (400 MHz, DCM-d_2) of **Pt(L$_{pDMA}$)PPh$_3$**.

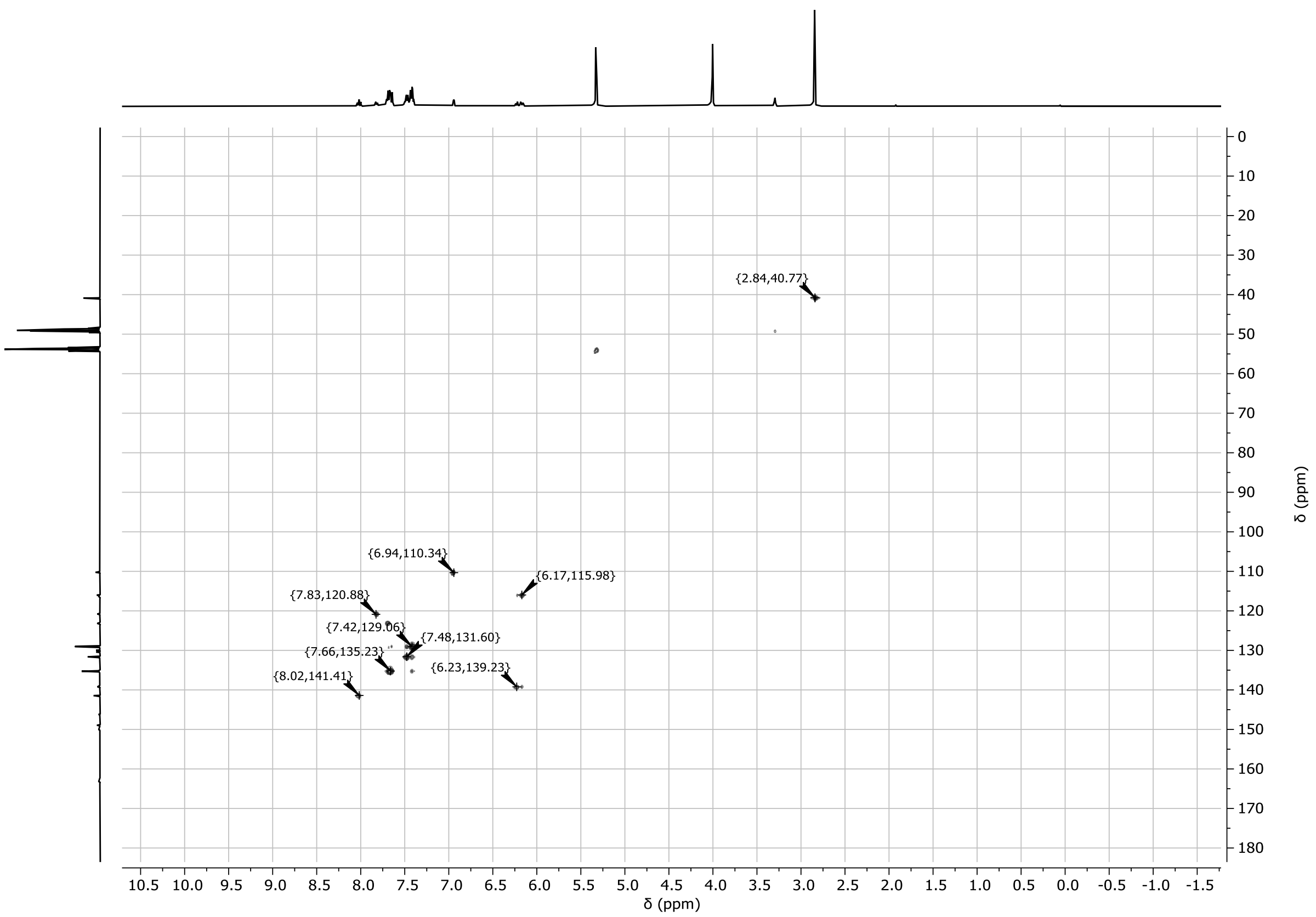

Figure S-256. HC-HSQC-NMR spectrum (101 MHz (^{13}C), 400 MHz (^{1}H), DCM-d_2) of **Pt(L$_{pDMA}$)PPh$_3$**.

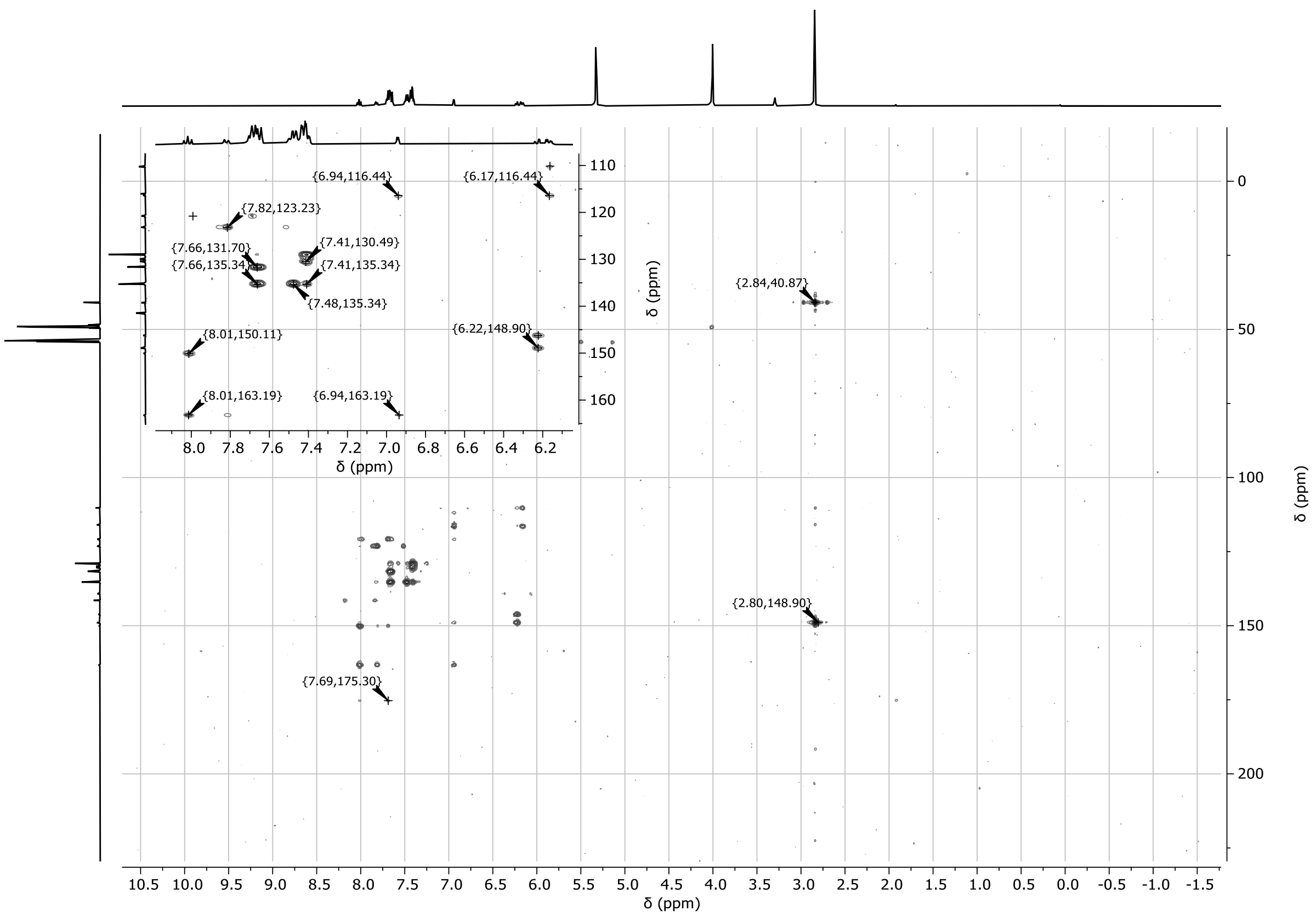

Figure S-257. HC-HMBC-NMR spectrum (101 MHz (^{13}C), 400 MHz (^{1}H), DCM-d_2) of **Pt(L_{pDMA})PPh_3**.

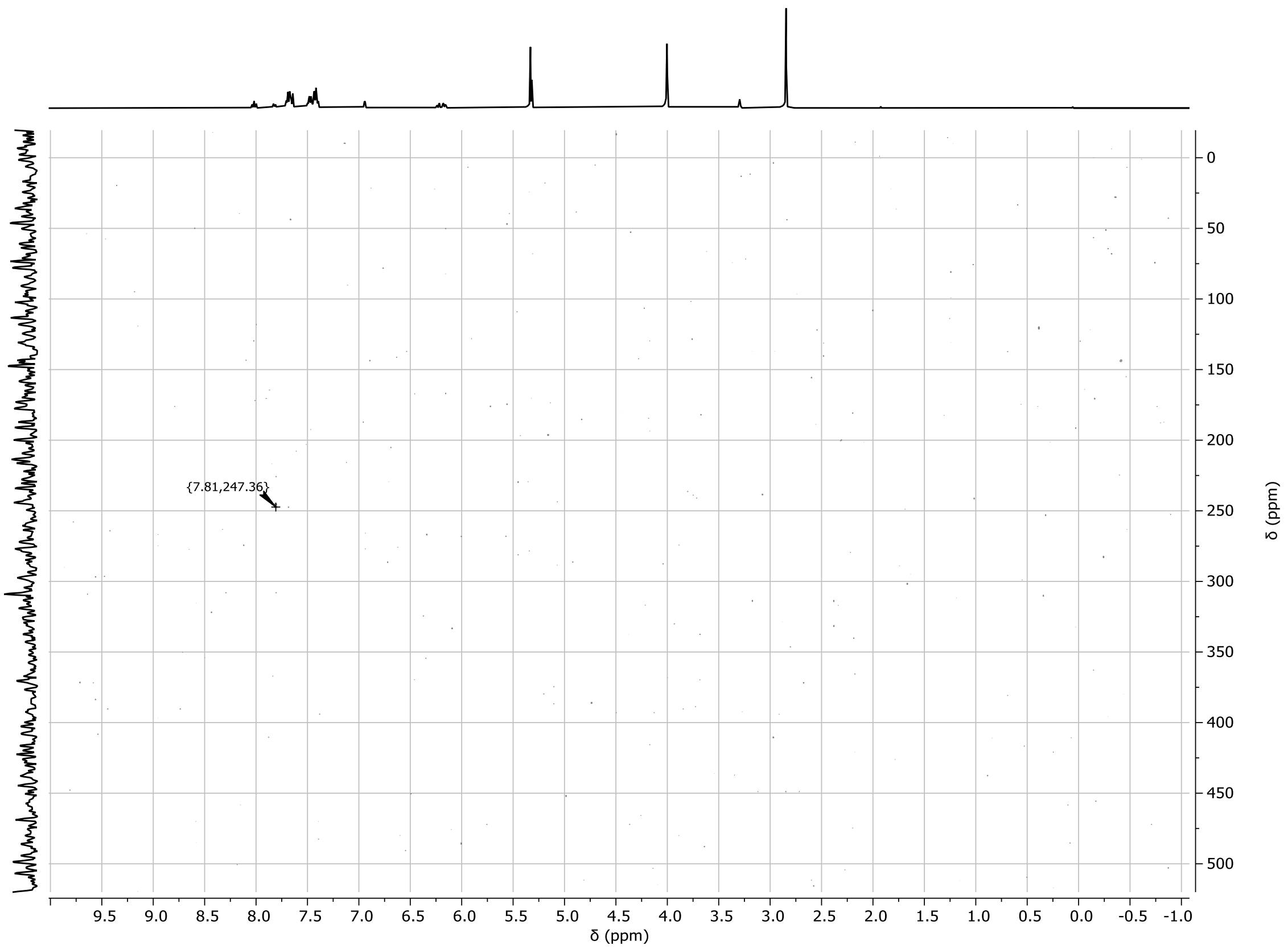

Figure S-258. HN-HMBC-NMR spectrum (41 MHz (^{15}N), 400 MHz (^{1}H), DCM-d_2) of **Pt(L_{pDMA})PPh_3**.

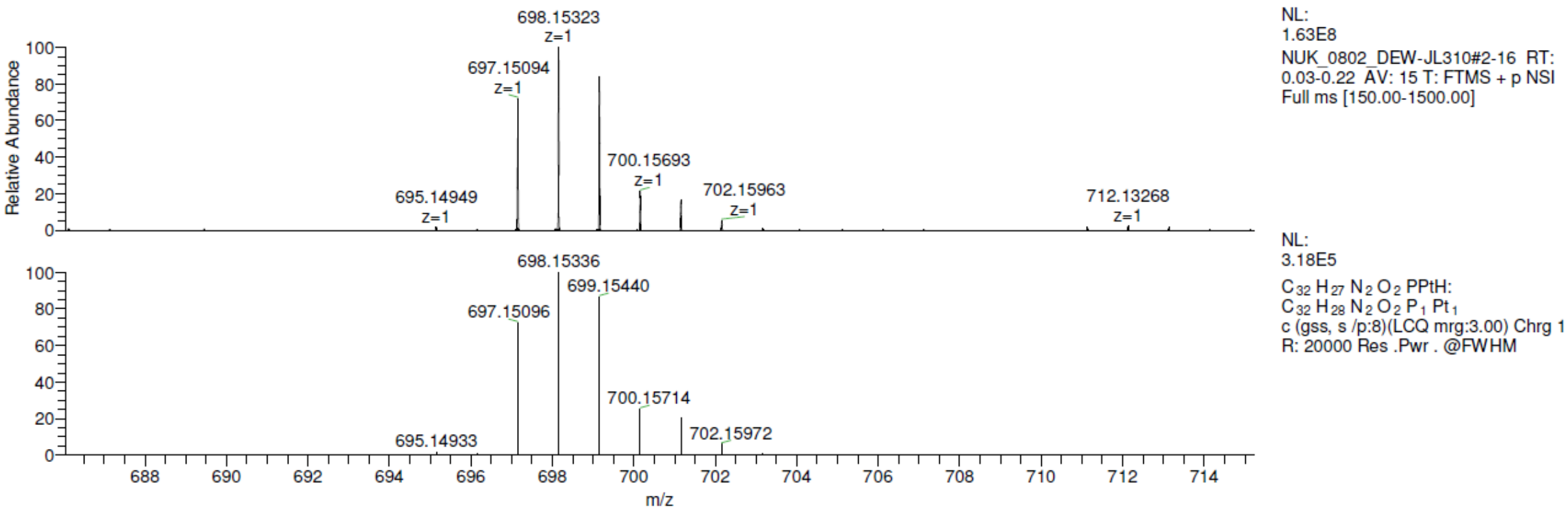

Figure S-259. Mass spectrum of **Pt(L$_{pDMA}$)PPh$_3$** (MeOH). Additional simulation of the [**Pt(L$_{pDMA}$)PPh$_3$**+H]$^+$ adduct.

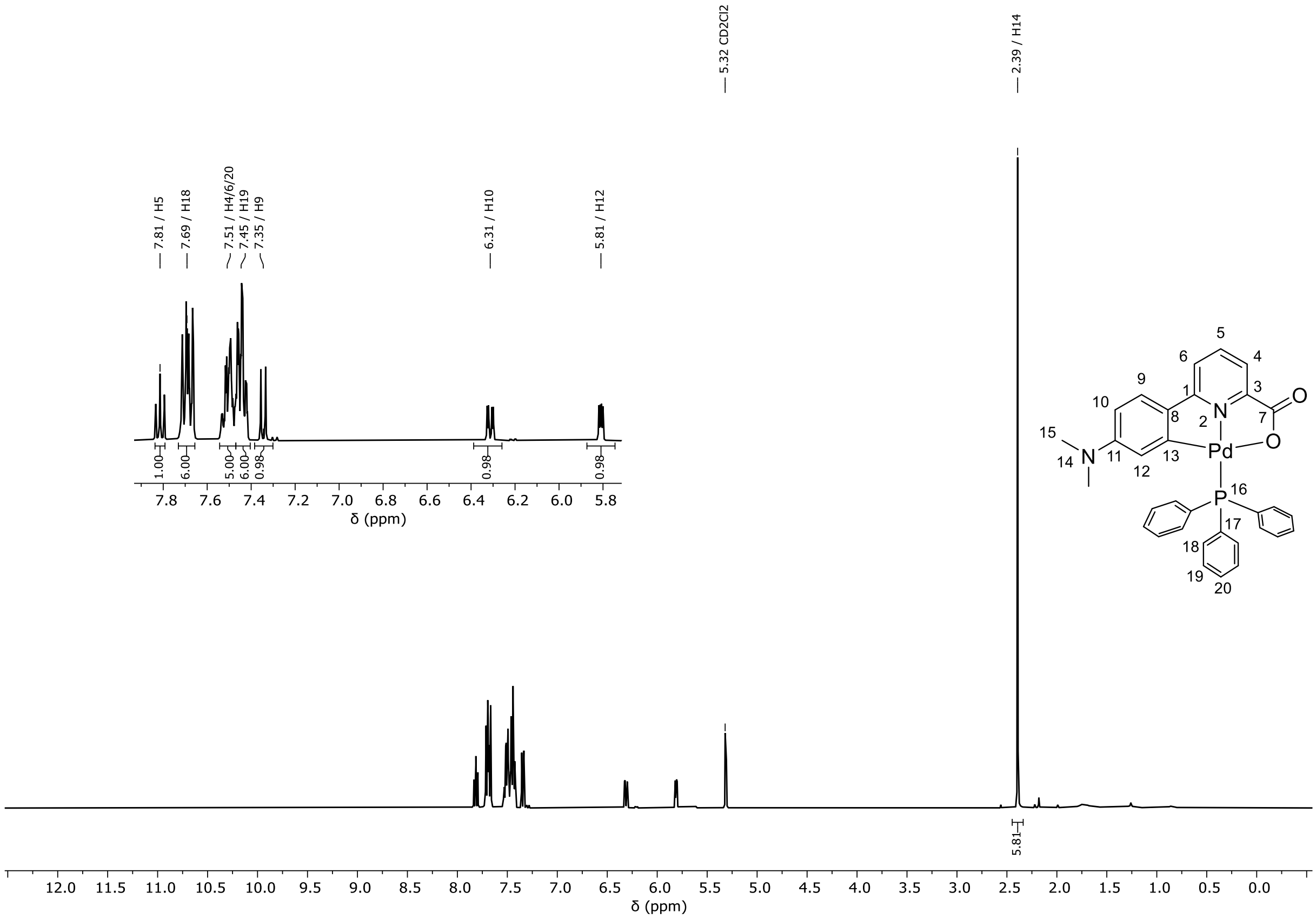

Figure S-260. ^{1}H-NMR spectrum (500 MHz, DCM-d_2) of **Pd(L$_{mDMA}$)PPh$_3$**.

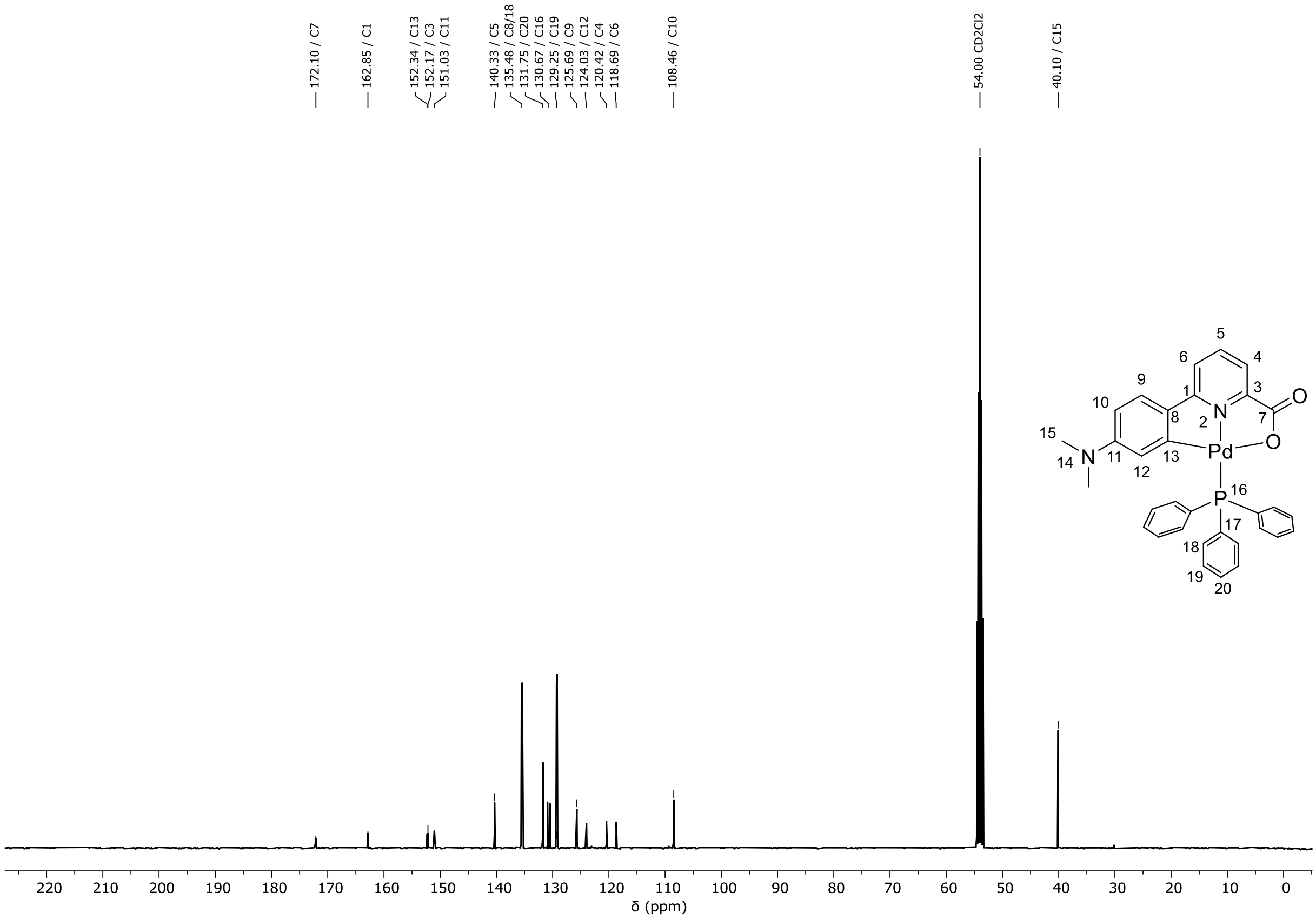

Figure S-261. $^{13}C\{^1H\}$-NMR spectrum (126 MHz, DCM-d_2) of **Pd(L$_{mDMA}$)PPh$_3$**.

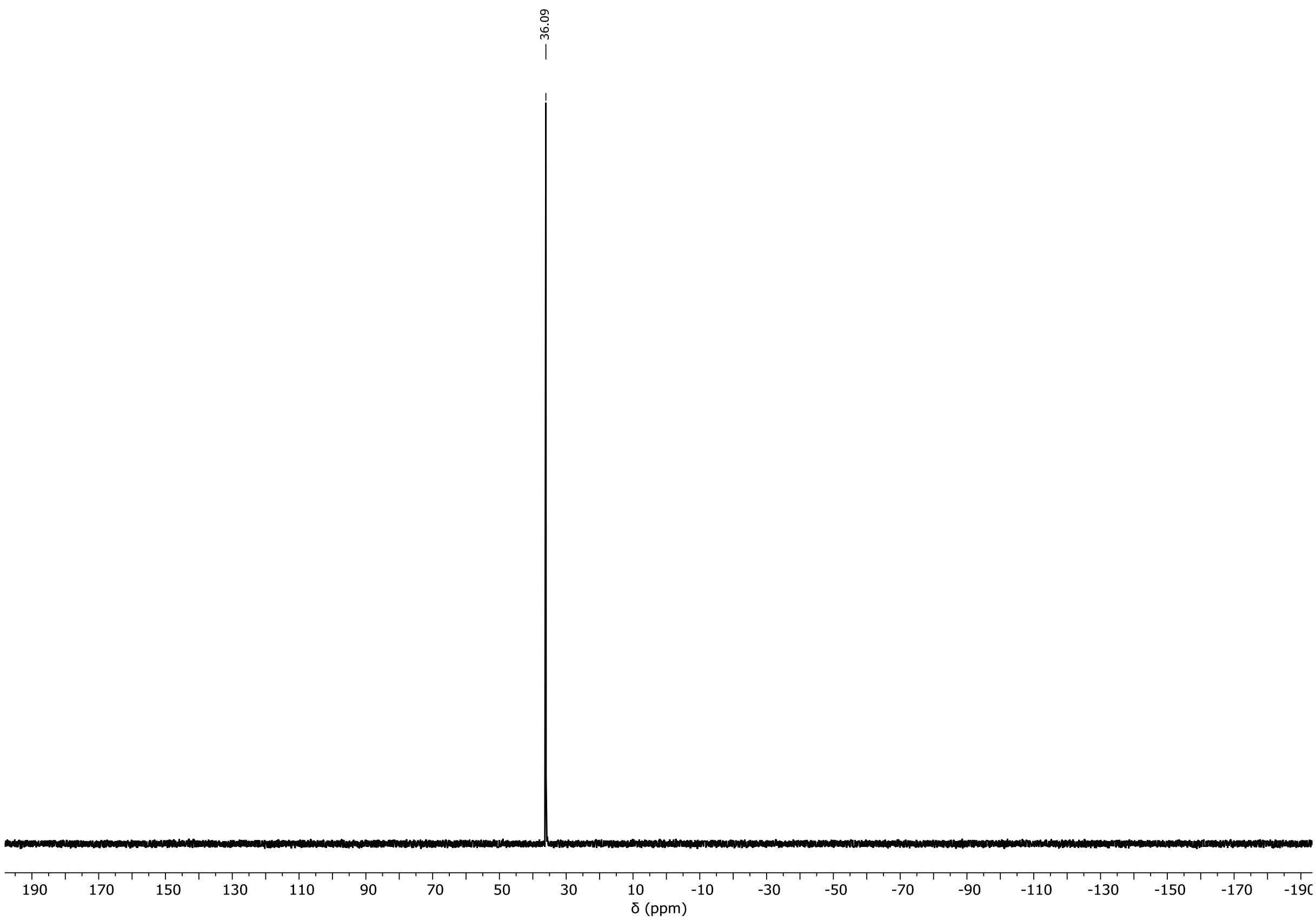

Figure S-262. $^{31}P\{^1H\}$-NMR spectrum (202 MHz, DCM-d_2) of **Pd(L$_{mDMA}$)PPh$_3$**.

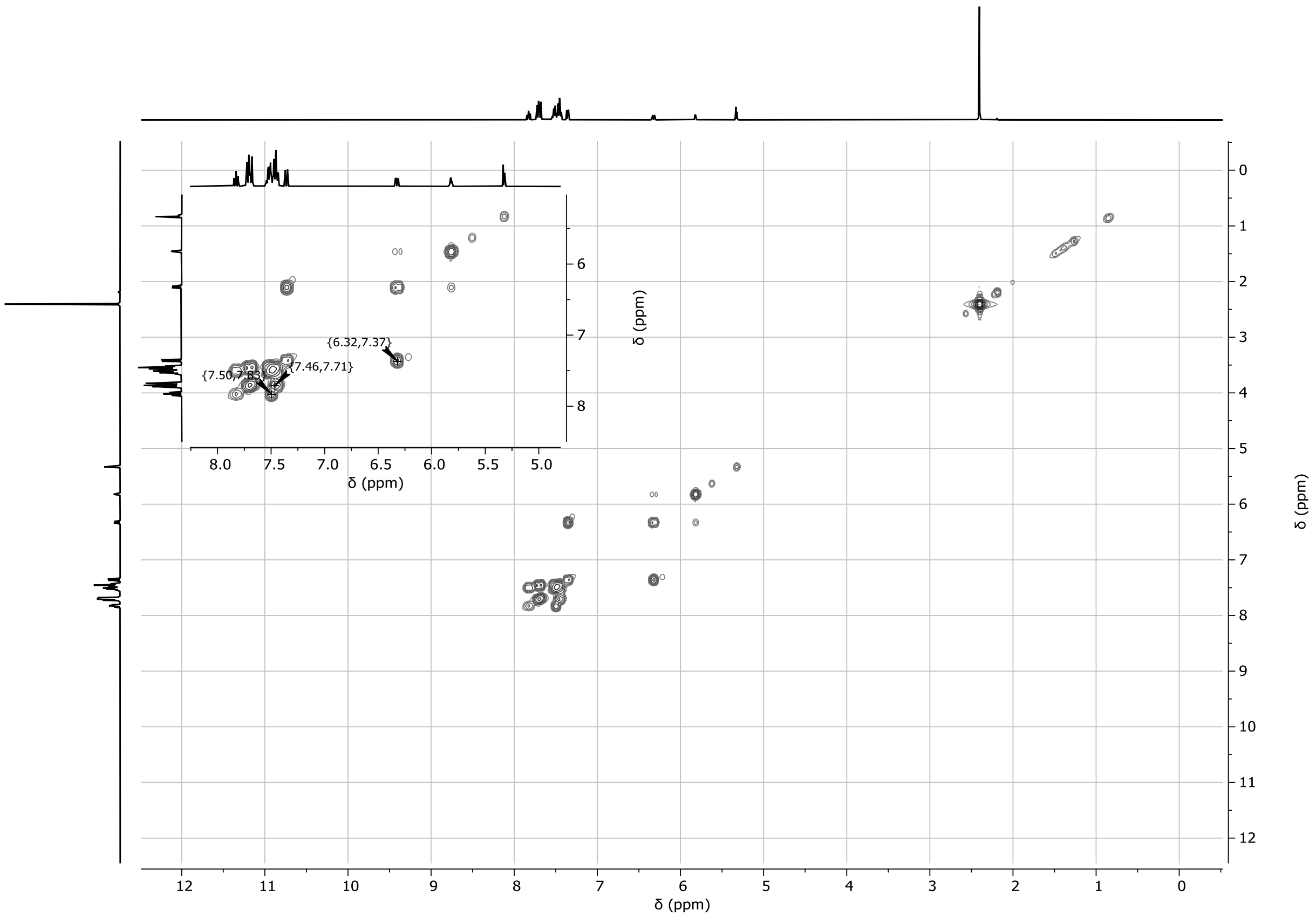

Figure S-263. HH-COSY-NMR spectrum (500 MHz, DCM-d_2) of **Pd(L$_{mDMA}$)PPh$_3$.**

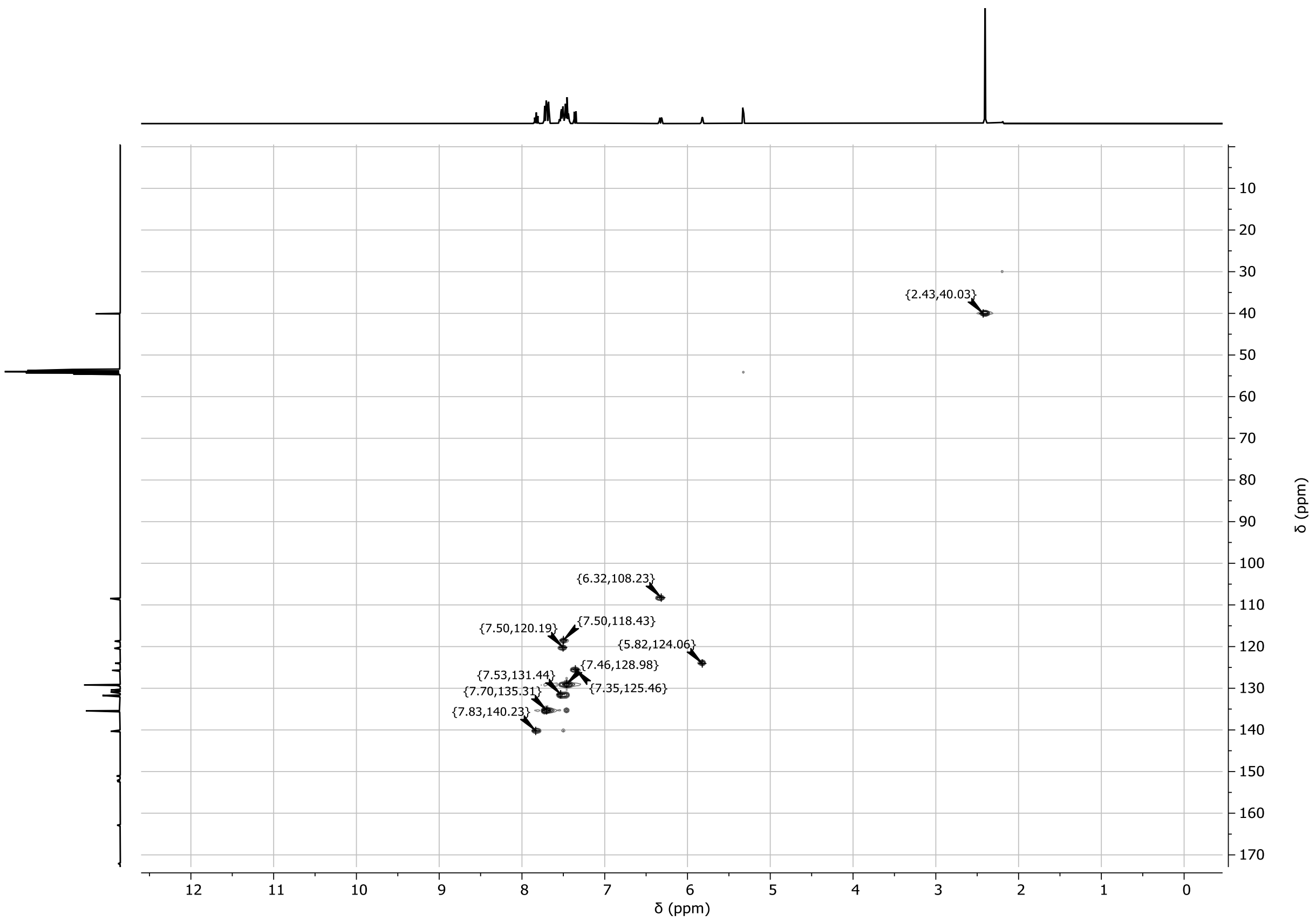

Figure S-264. HC-HSQC-NMR spectrum (126 MHz (^{13}C), 500 MHz (^{1}H), DCM-d_2) of **Pd(L$_{mDMA}$)PPh$_3$.**

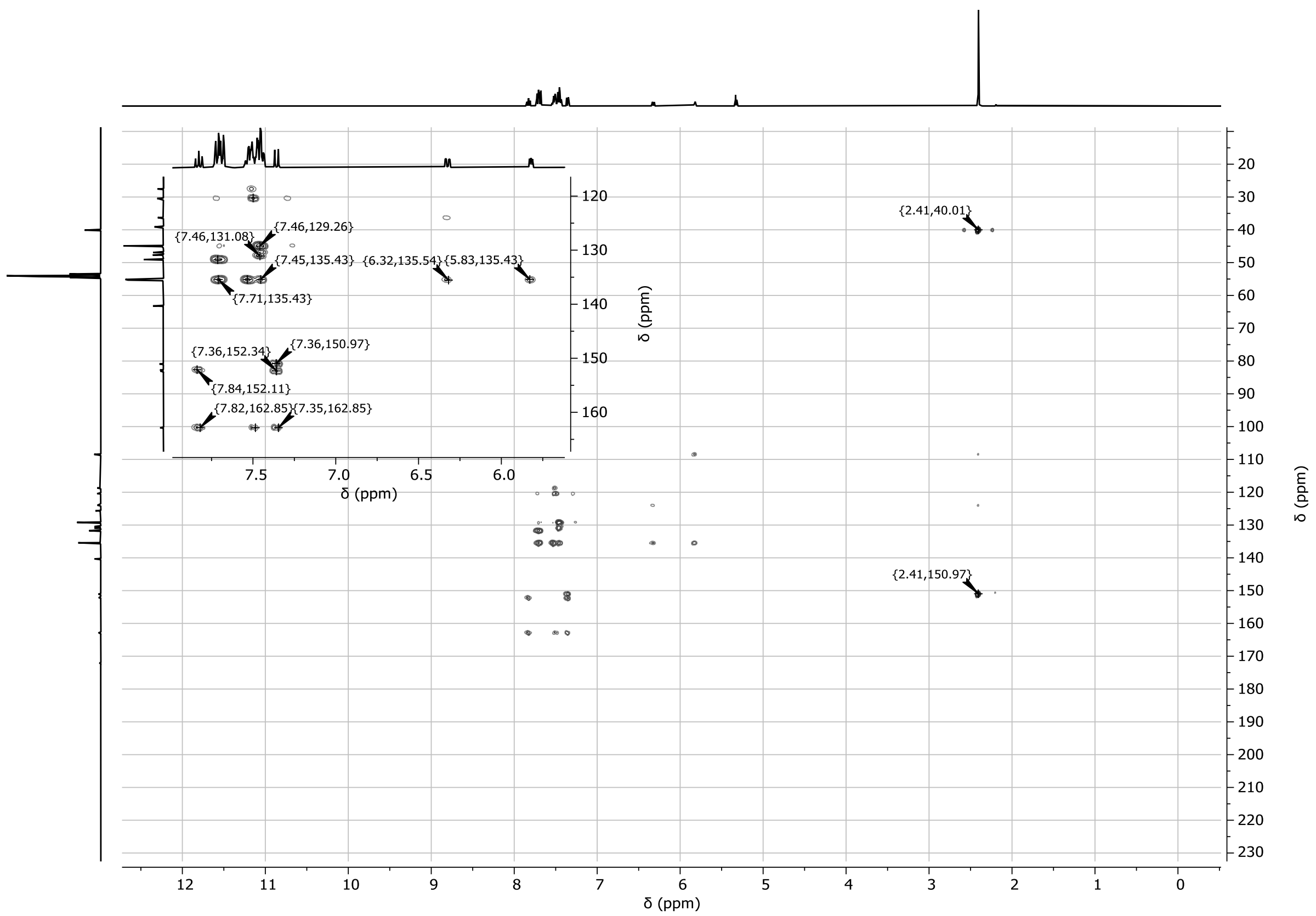

Figure S-265. HC-HMBC-NMR spectrum (126 MHz (^{13}C), 500 MHz (^{1}H), DCM-d_2) of **Pd(L$_{mDMA}$)PPh$_3$**.

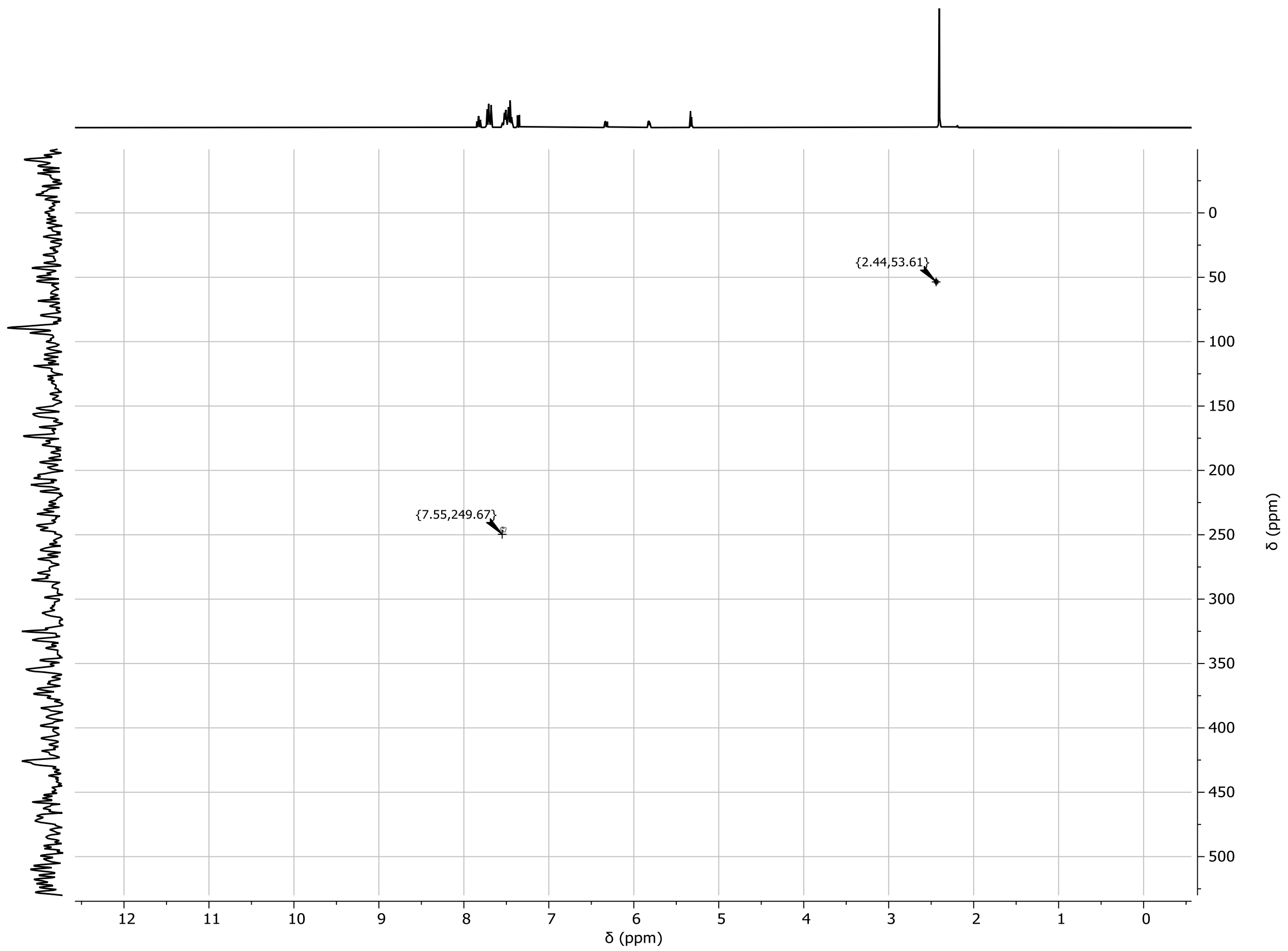

Figure S-266. HN-HMBC-NMR spectrum (51 MHz (^{15}N), 500 MHz (^{1}H), DCM-d_2) of **Pd(L$_{mDMA}$)PPh$_3$.**

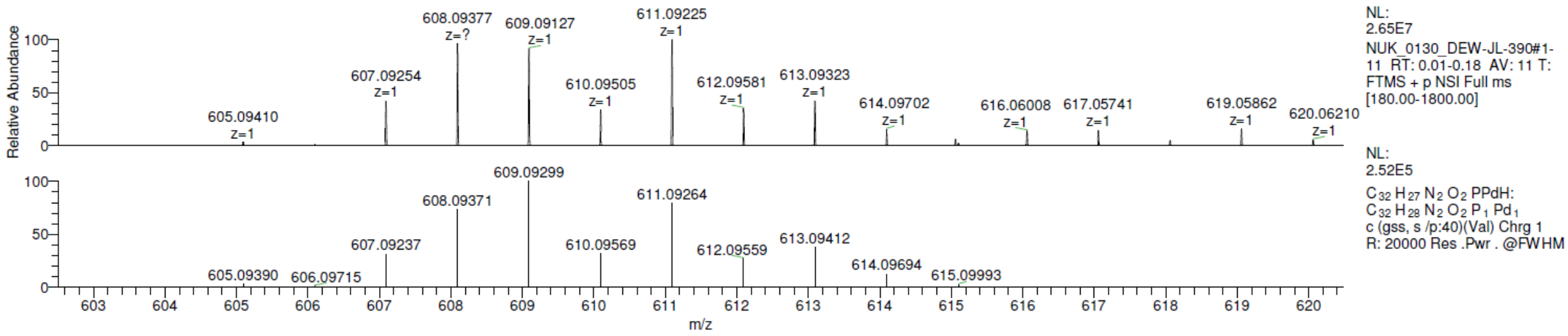

Figure S-267. Mass spectrum of **Pd(L$_{mDMA}$)PPh$_3$** (MeOH). Additional simulation of the [**Pd(L$_{mDMA}$)PPh$_3$**+H]$^+$ adduct.

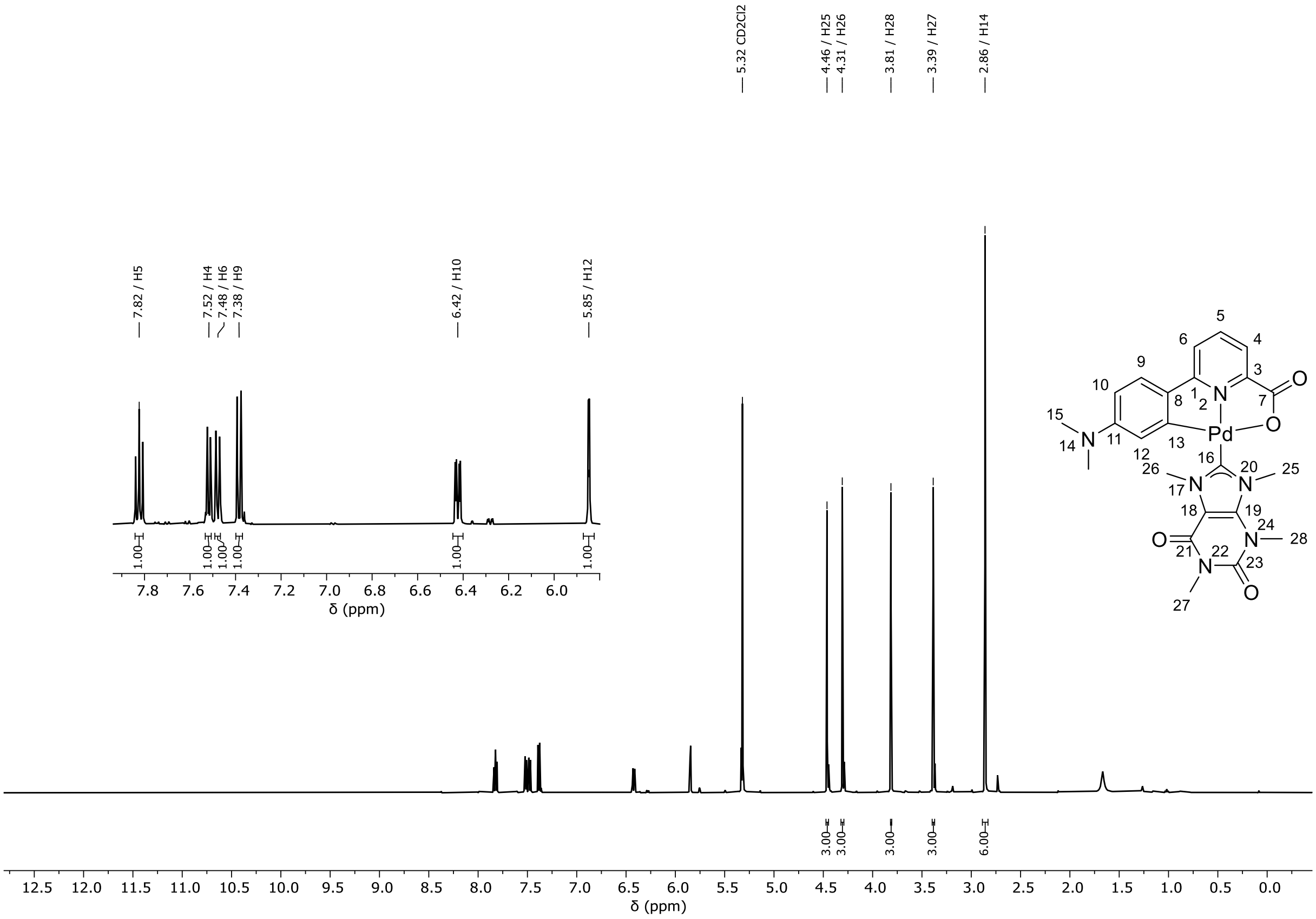

Figure S-268. ^{1}H-NMR spectrum (400 MHz, DCM-d_2) of **Pd(L$_{mDMA}$)PPh$_3$**.

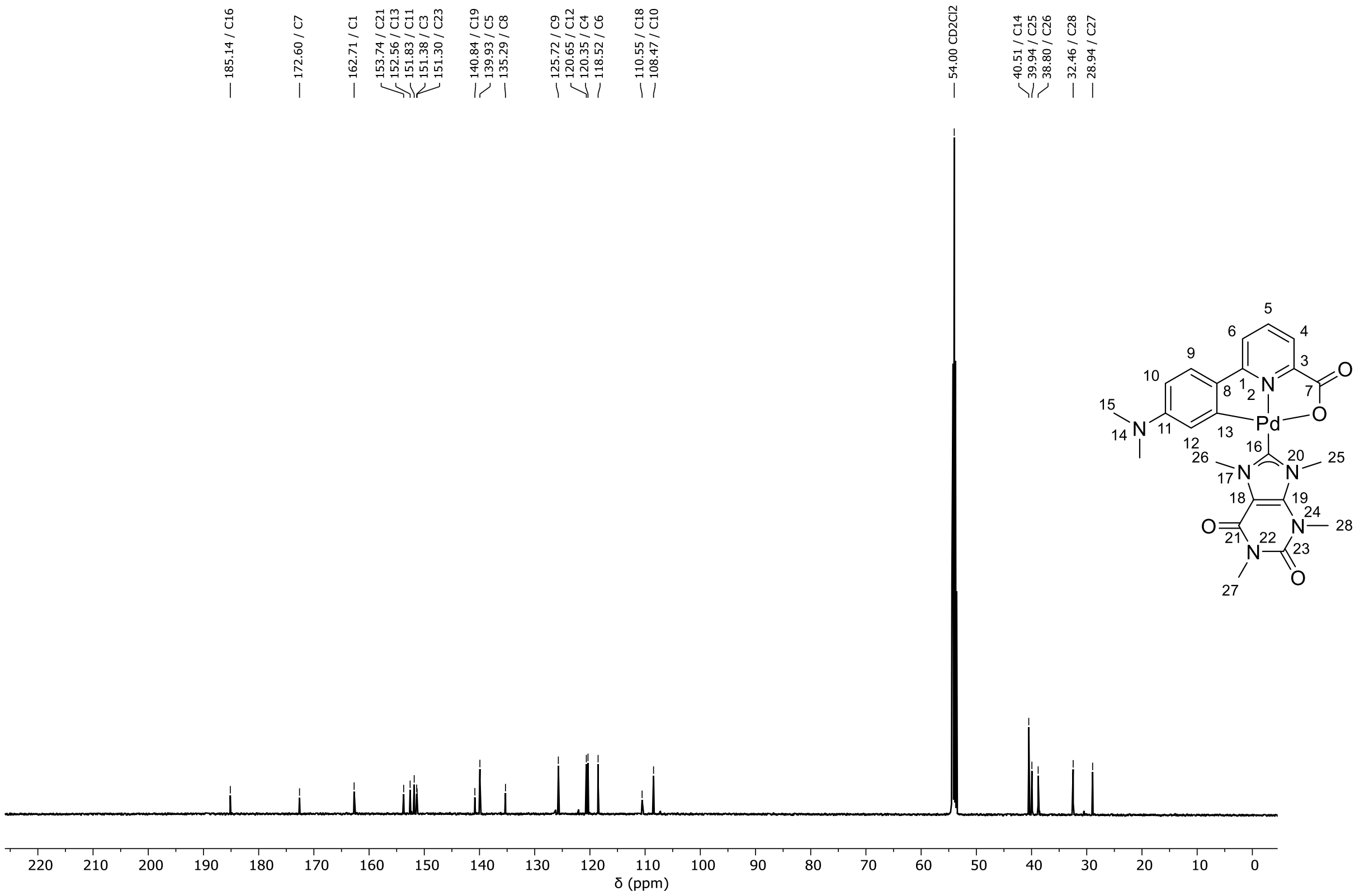

Figure S-269. $^{13}C\{^{1}H\}$-NMR spectrum (101 MHz, DCM-d_2) of **Pd(L$_{mDMA}$)NHC**.

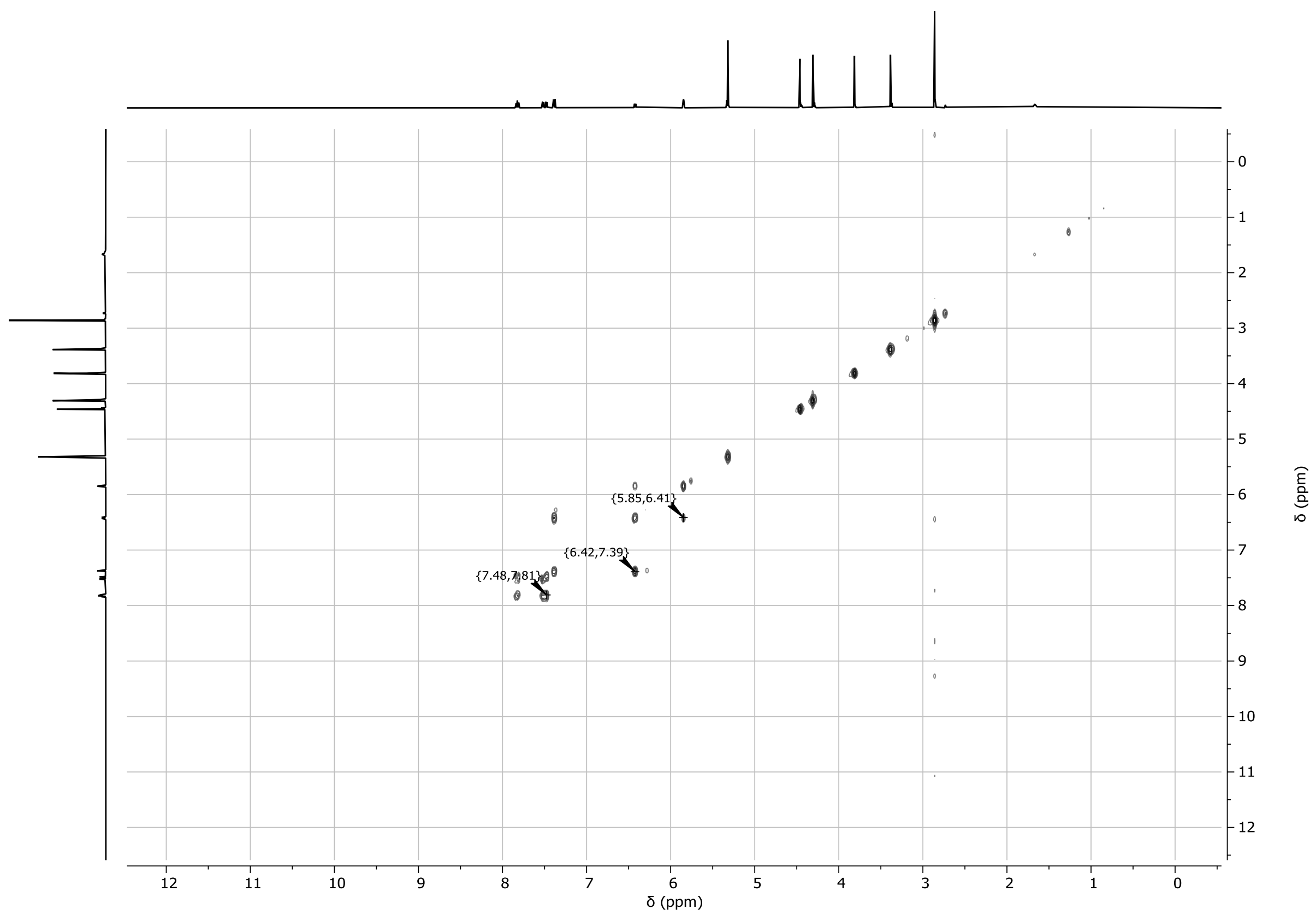

Figure S-270. HH-COSY-NMR spectrum (400 MHz, DCM-d_2) of **Pd(L$_{mDMA}$)NHC**.

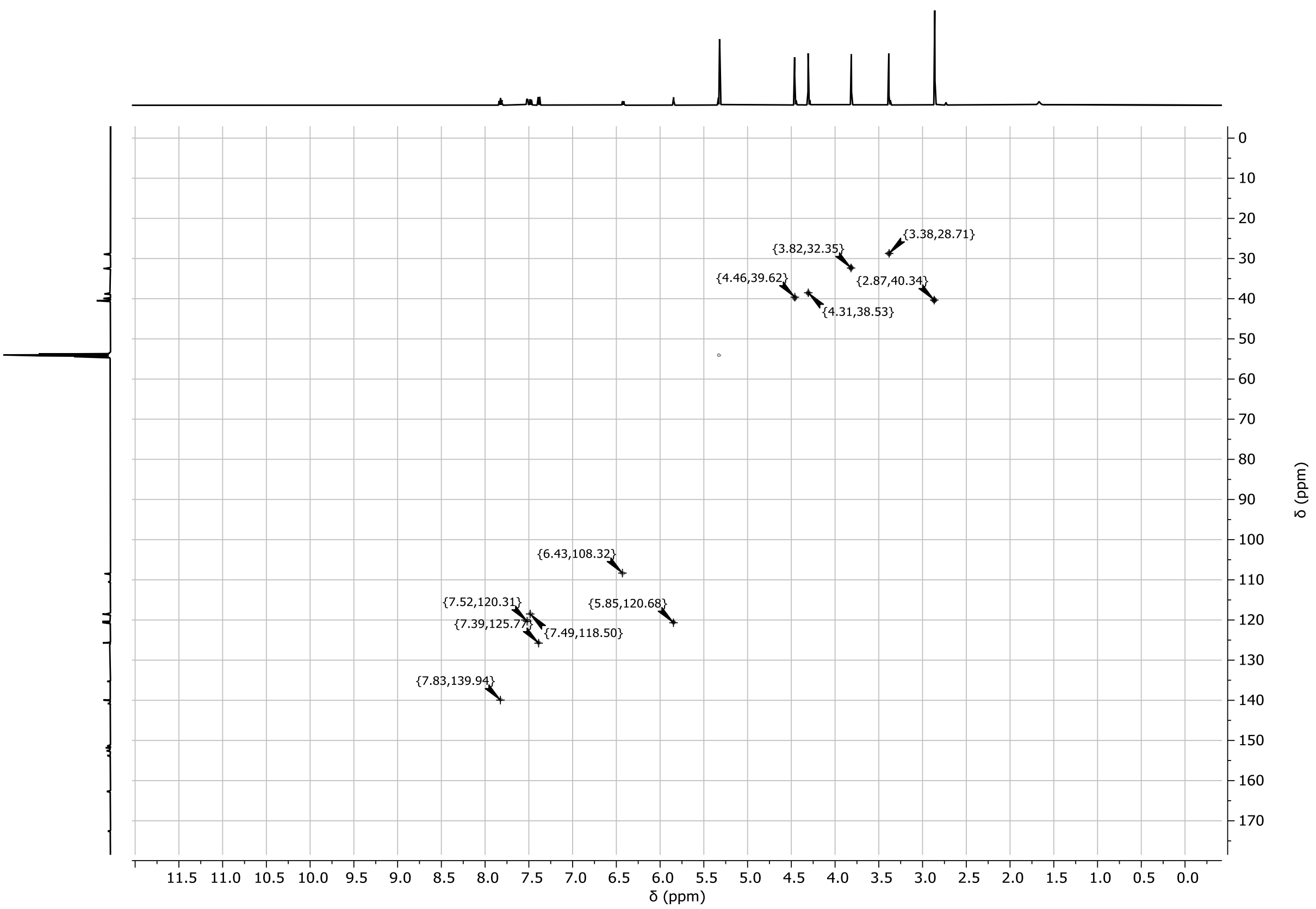

Figure S-271. HC-HSQC-NMR spectrum (101 MHz (^{13}C), 400 MHz (^{1}H), DCM-d_2) of **Pd(L$_{mDMA}$)NHC**.

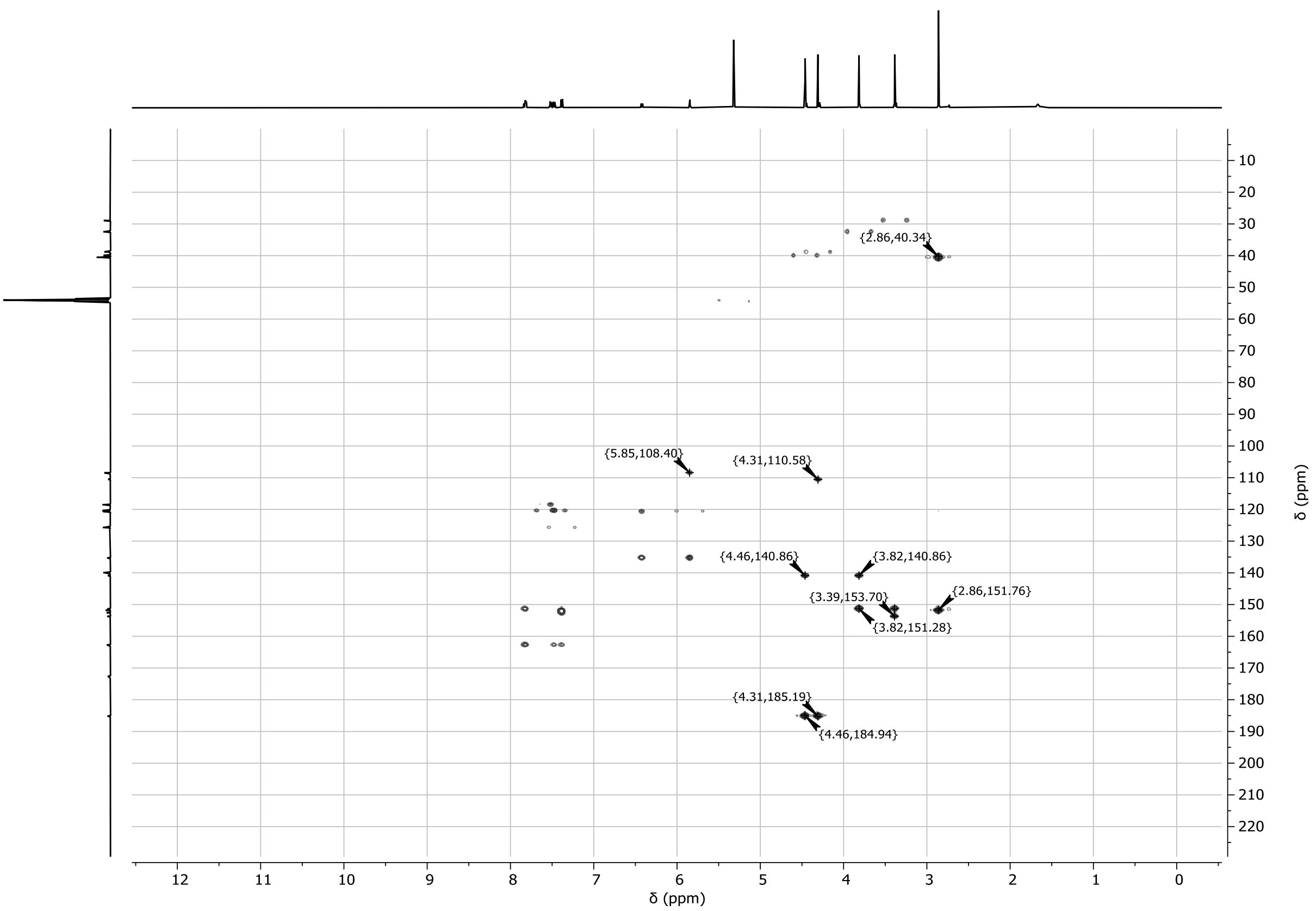

Figure S-272. HC-HMBC-NMR spectrum (101 MHz (^{13}C), 400 MHz (^{1}H), DCM-d_2) of **Pd(L$_{mDMA}$)NHC**.

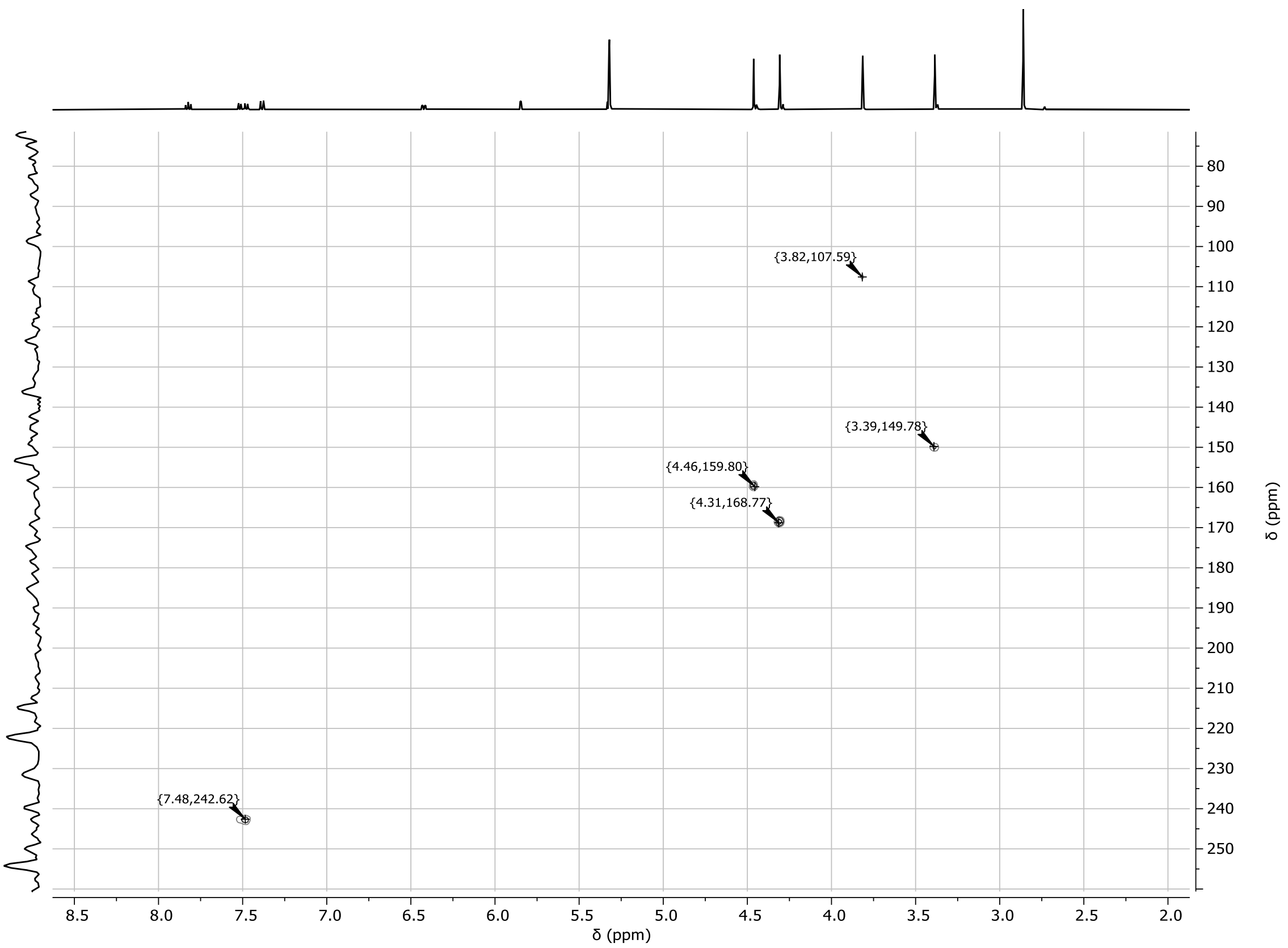

Figure S-273. HN-HMBC-NMR spectrum (41 MHz (^{15}N), 400 MHz (^{1}H), DCM-d_2) of **Pd(L$_{mDMA}$)NHC**.

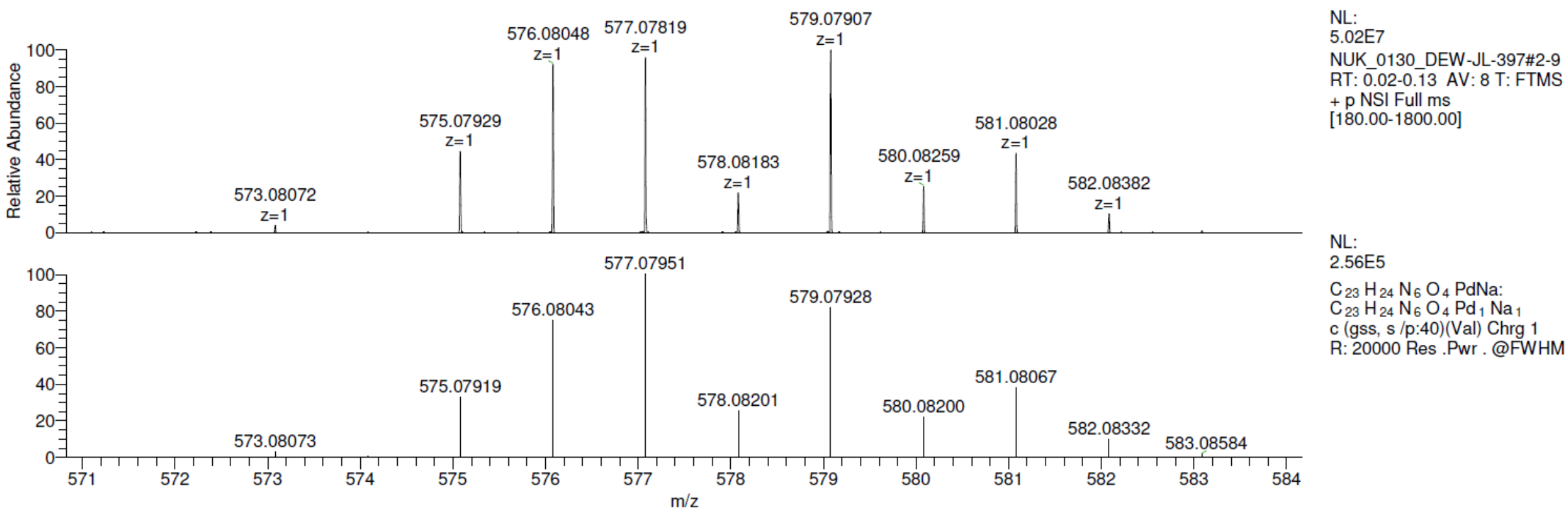

Figure S-274. Mass spectrum of **Pd(L$_{mDMA}$)NHC** (MeOH). Additional simulation of the [**Pd(L$_{mDMA}$)NHC**+Na]$^+$ adduct.

Section S4: X-ray diffractometry data

Table S-1. Overview of the crystallographic data for **Pt(L_{NCO})PPh_3**, **Pt(L_H)PPh_3** and **Pt(L_H)AmPy**.

Complex	Pt(L_{NCO})PPh_3	Pt(L_H)PPh_3	Pt(L_H)AmPy
**Formula**	$C_{34}H_{29}FNO_2PPt$	$C_{31}H_{24}NO_2PPt$	$C_{22}H_{22}N_2O_2Pt$
**MW**	728.67	654.57	541.51
**CCDC No.**	2422728	2422730	2422729
**Crystal color**	yellow	yellow	orange
**a / Å**	33.5646(11)	9.8884(4)	9.5347(3)
**b / Å**	9.6669(2)	18.2997(7)	10.4288(4)
**c / Å**	18.8684(6)	15.4132(6)	11.0290(4)
**V / Å³**	4721.41(30)	27575.50(19)	905.747(6)
**α / °**	90	90	108.6780(10)
**β / °**	102.694(3)	98.6320(10)	108.2760(10)
**γ / °**	90	90	104.8350(10)
**Space group**	*C*2/*c* (15)	$P2_1/c$ (14)	$P\bar{1}$ (2)
**Crystal system**	monoclinic	monoclinic	triclinic
**Z value**	8	4	2
**F_{000}**	2864.0	1440.0	524.0
**h, k, l_{max}**	$-47 \le h \le 47$, $-13 \le k \le 13$, $-26 \le l \le 26$	$-13 \le h \le 13$, $-25 \le k \le 25$, $-21 \le l \le 21$	$-14 \le h \le 14$, $-15 \le k \le 15$, $-16 \le l \le 16$
**N_{ref}**	8187	8038	6204
**T_{min}, T_{max}**	0.631, 0.746	0.437, 0.746	0.406, 0.746
**μ(MoKα) / mm^{-1}**	5.106	5.370	7.766
**Goodness-of-fit on F^2**	1.047	1.078	1.078
**Reflections collected**	45597	43677	16729
**Independent reflections**	7912 [R_{int} = 0.0176, R_{sigma} = 0.0254]	8038 [R_{int} = 0.0202, R_{sigma} = 0.0269]	6204 [R_{int} = 0.0370, R_{sigma} = 0.0334]
**Largest diff. peak/hole / eÅ^{-3}**	0.534/-0.540	0.960/-0.875	1.243/-2.439

Table S-2: Overview of the crystallographic data for **Pt(L_{tBu})AmPy**, **Pt(L_{DiF})PPh$_3$** and **Pt(L_{pOMe})PPh$_3$**.

Complex	Pt(L_{tBu})AmPy	Pt(L_{DiF})PPh$_3$	Pt(L_{pOMe})PPh$_3$
**Formula**	$C_{26}H_{30}N_2O_2Pt$	$C_{30}H_{20}NFO_2PPt$	$C_{31}H_{24}NO_3PPt$
**MW**	597.62	690.50	684.59
**CCDC No.**	2422727	2422724	2422726
**Crystal color**	orange	yellow	yellow
**a / Å**	10.5569(9)	13.7346(4)	9.7926(3)
**b / Å**	30.210(3)	17.4579(5)	18.8782(5)
**c / Å**	15.1751(13)	22.2418(6)	15.6827(4)
**V / Å³**	4721.41(70)	5098.51(30)	1451.85(8)
**α / °**	94.2720(10)	74.6540(10)	90
**β / °**	105.5020(10)	83.2770(10)	94.463(1)
**γ / °**	91.2700(10)	84.9530(10)	90
**Space group**	$P2_1/c$ (14)	$P\bar{1}$ (2)	$P2_1/c$ (14)
**Crystal system**	monoclinic	triclinic	monoclinic
**Z value**	8	8	4
**F_{000}**	2352.0	2756.0	1504.0
**h, k, l_{max}**	$-13\leq h\leq 13$, $-39\leq k\leq 39$, $-19\leq l\leq 19$	$-18\leq h\leq 18$, $-22\leq k\leq 22$, $-29\leq l\leq 29$	$-14\leq h\leq 14$, $-28\leq k\leq 28$, $-23\leq l\leq 23$
**N_{ref}**	11226	24311	10085
**T_{min}, T_{max}**	0.400, 0.613	0.399, 0.746	0.518, 0.746
**μ(MoKα) / mm^{-1}**	5.968	5.664	5.130
**Goodness-of-fit on F^2**	1.036	1.109	1.107
**Reflections collected**	55544	74830	56267
**Independent reflections**	8801 [R_{int} = 0.0540, R_{sigma} = 0.0662]	23490 [R_{int} = 0.0330, R_{sigma} = 0.0342]	9691 [R_{int} = 0.0211, R_{sigma} = 0.0310]
**Largest diff. peak/hole / eÅ^{-3}**	2.402/-1.215	1.211/-1.838	0.628/-0.974

Table S-3. Overview of the crystallographic data for **Pd(L$_{mOMe}$)PPh$_3$**, **Pt(L$_{mDMA}$)PPh$_3$** and **Pt(L$_{pDMA}$)PPh$_3$**.

Complex	Pd(L$_{mOMe}$)PPh$_3$	Pt(L$_{mDMA}$)PPh$_3$	Pt(L$_{pDMA}$)PPh$_3$
**Formula**	$C_{31}H_{24}NO_3PPd$	$C_{32}H_{27}N_2O_2PPt$	$C_{32}H_{27}N_2O_2PPt$
**MW**	595.93	697.64	697.64
**CCDC No.**	2422722	2422723	2422725
**Crystal color**	yellow	red	red
**a / Å**	8.2730(2)	9.0261(4)	9.2513(3)
**b / Å**	9.1217(2)	9.8602(4)	10.0935(3)
**c / Å**	17.5958(4)	15.3011(6)	15.9969(5)
**V / Å³**	1201.17(5)	1307.34(9)	1451.85(8)
**α / °**	92.588(1)	94.2720(10)	78.925(1)
**β / °**	101.196(1)	105.5020(10)	85.029(1)
**γ / °**	111.599(1)	91.2700(10)	82.966(1)
**Space group**	$P\bar{1}$ (2)	$P\bar{1}$ (2)	$P\bar{1}$ (2)
**Crystal system**	triclinic	triclinic	triclinic
**Z value**	2	2	2
**F_{000}**	604.0	684.0	768.0
**h, k, l_{max}**	$-12 \leq h \leq 12$, $-13 \leq k \leq 13$, $-26 \leq l \leq 26$	$-11 \leq h \leq 11$, $-12 \leq k \leq 12$, $-20 \leq l \leq 20$	$-13 \leq h \leq 13$, $-15 \leq k \leq 15$, $-23 \leq l \leq 23$
**N_{ref}**	8278	6141	9979
**T_{min}, T_{max}**	0.626, 0.746	0.533, 0.746	0.597, 0.746
**μ(MoKα) / mm^{-1}**	0.876	5.461	5.106
**Goodness-of-fit on F^2**	1.029	1.114	1.075
**Reflections collected**	23321	16538	28460
**Independent reflections**	8015 [R_{int} = 0.0221, R_{sigma} = 0.0192]	6079 [R_{int} = 0.0279, R_{sigma} = 0.0246]	9836 [R_{int} = 0.0275, R_{sigma} = 0.0247]
**Largest diff. peak/hole / eÅ^{-3}**	2.402/-1.215	0.952/-0.949	1.019/-1.126

Section S4.1: X-ray diffractometric results for single crystals of Pt(L_{NCO})PPh_3

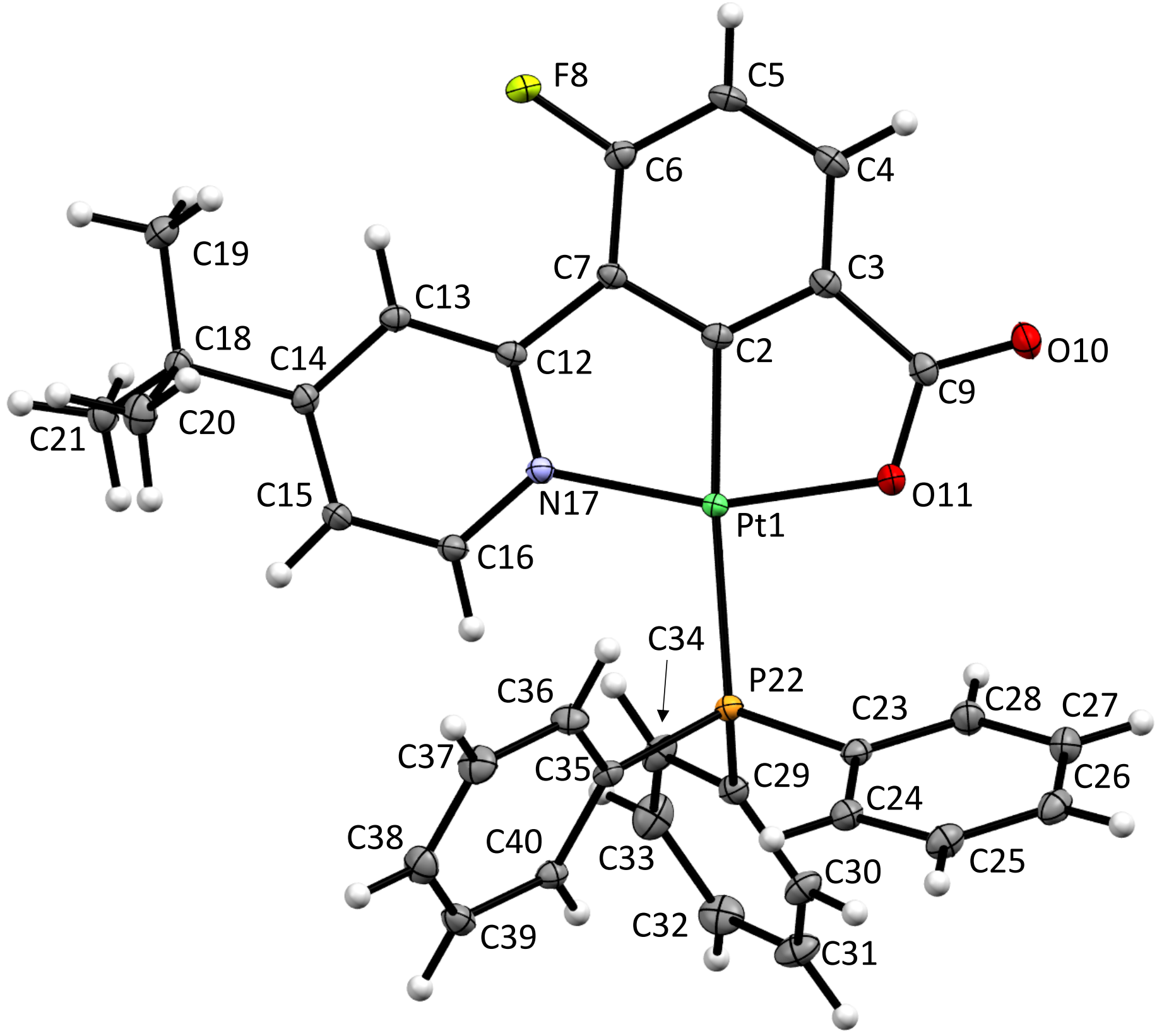

Figure S-275. Molecular structure of **Pt(L_{NCO})PPh_3** in the crystal with labelled atoms. Displacement ellipsoids are shown with 50% probability.

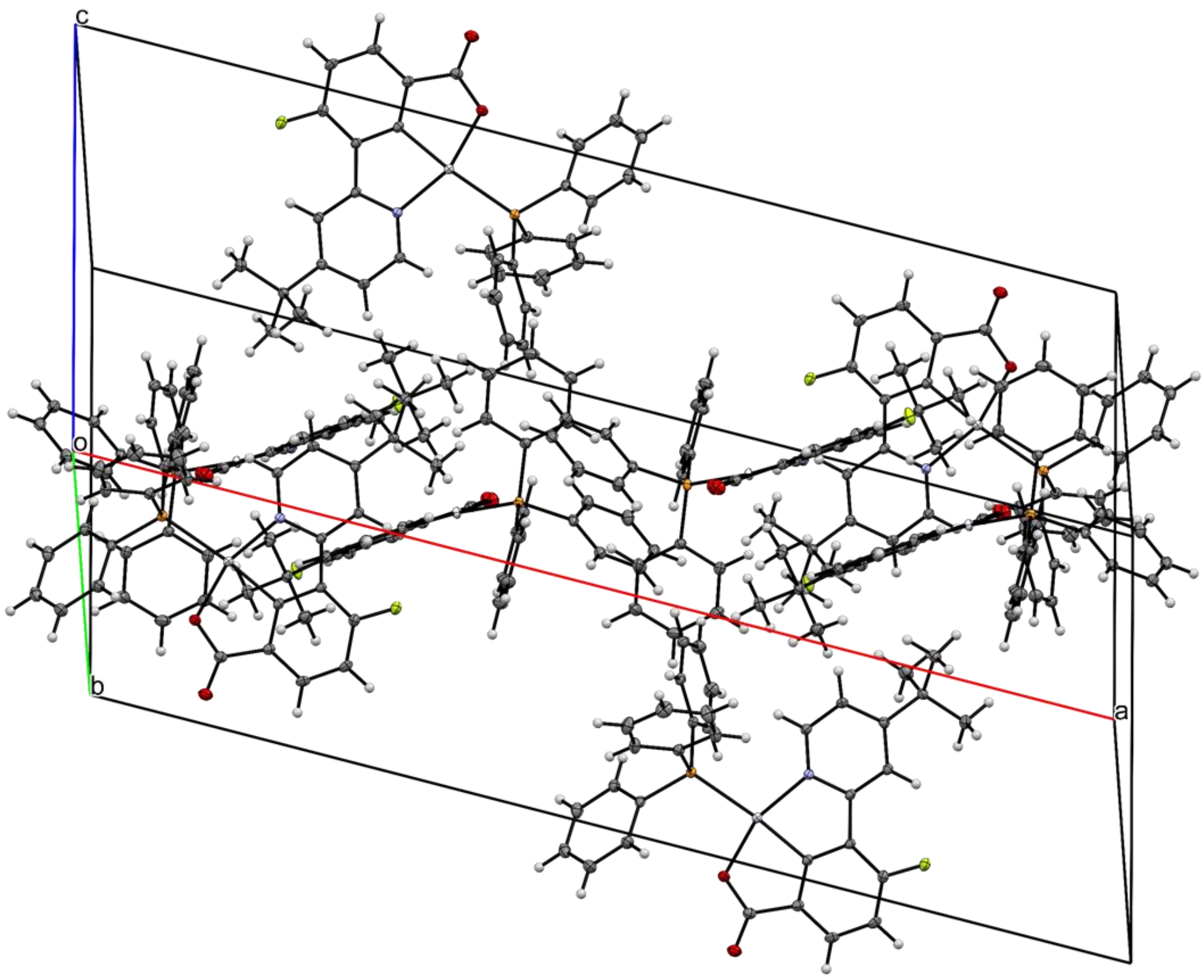

Figure S-276. Unit cell of **Pt(L_{NCO})PPh_3**. Displacement ellipsoids are shown with 50% probability.

Table S-4. Selected bond lengths and angles in **Pt(L_{NCO})PPh$_3$**.

**Bond length / angle**	
Pt1 – C2 / Å	1.943(2)
Pt1 – O11 / Å	2.042(1)
Pt1 – N17 / Å	2.042(1)
Pt1 – P22 / Å	2.3579(9)
C2 ⋯ Pt1 ⋯ O11 / °	80.74(5)
C2 ⋯ Pt1 ⋯ N17 / °	79.47(6)
C2 ⋯ Pt1 ⋯ P22 / °	173.82(5)
O11 ⋯ Pt1 ⋯ N17 / °	160.16(5)
011 ⋯ Pt1 ⋯ P22 / °	94.85(4)
N17 ⋯ Pt1 ⋯ P22 / °	104.98(4)
C2 ⋯ C3 ⋯ C8 / °	112.7(1)

Section S4.2: X-ray diffractometric results for single crystals of Pt(L_H)PPh_3

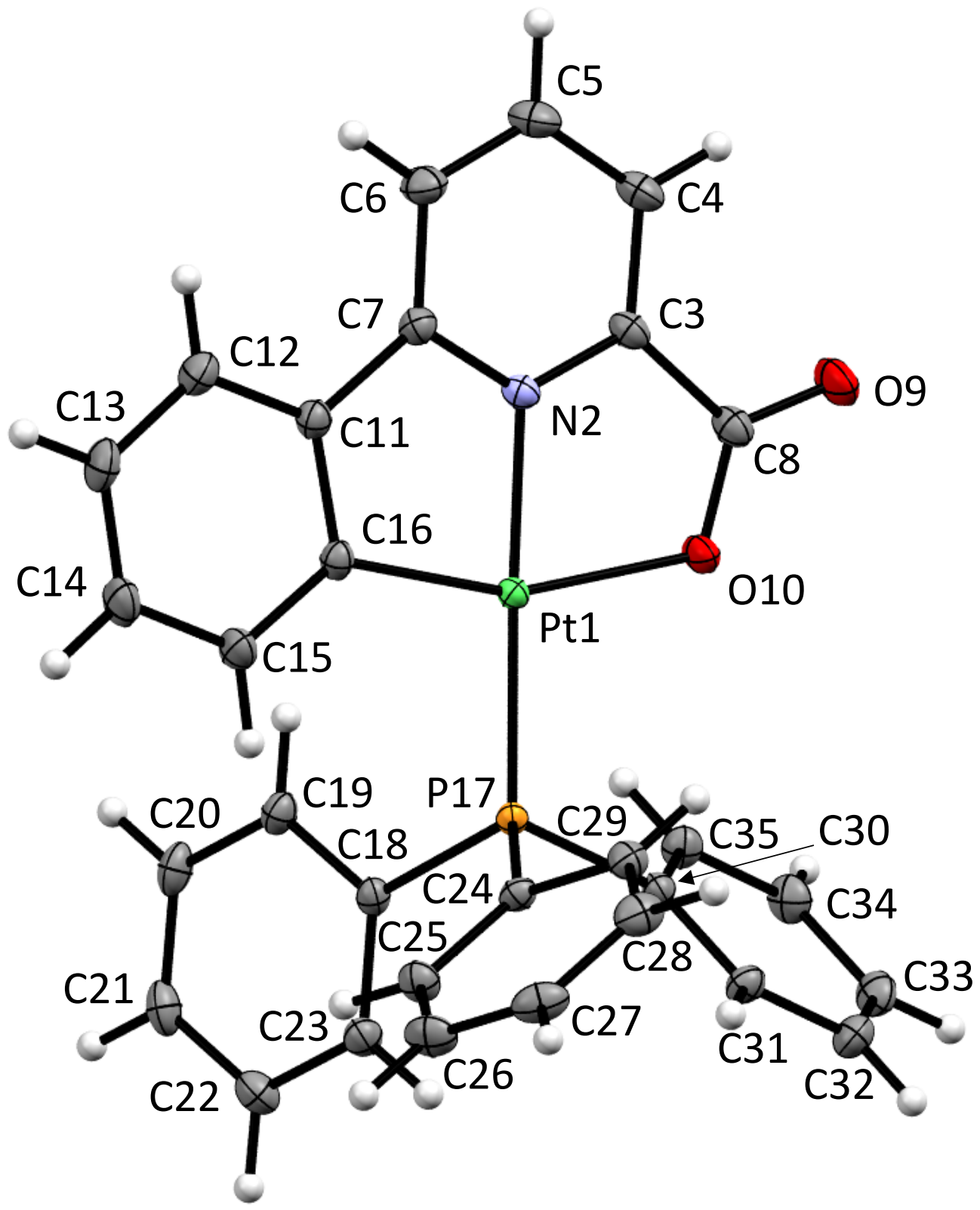

Figure S-277. Molecular structure of **Pt(L_H)PPh_3** in the crystal with labelled atoms. Displacement ellipsoids are shown with 50% probability. Solvent molecules were omitted for clarity.

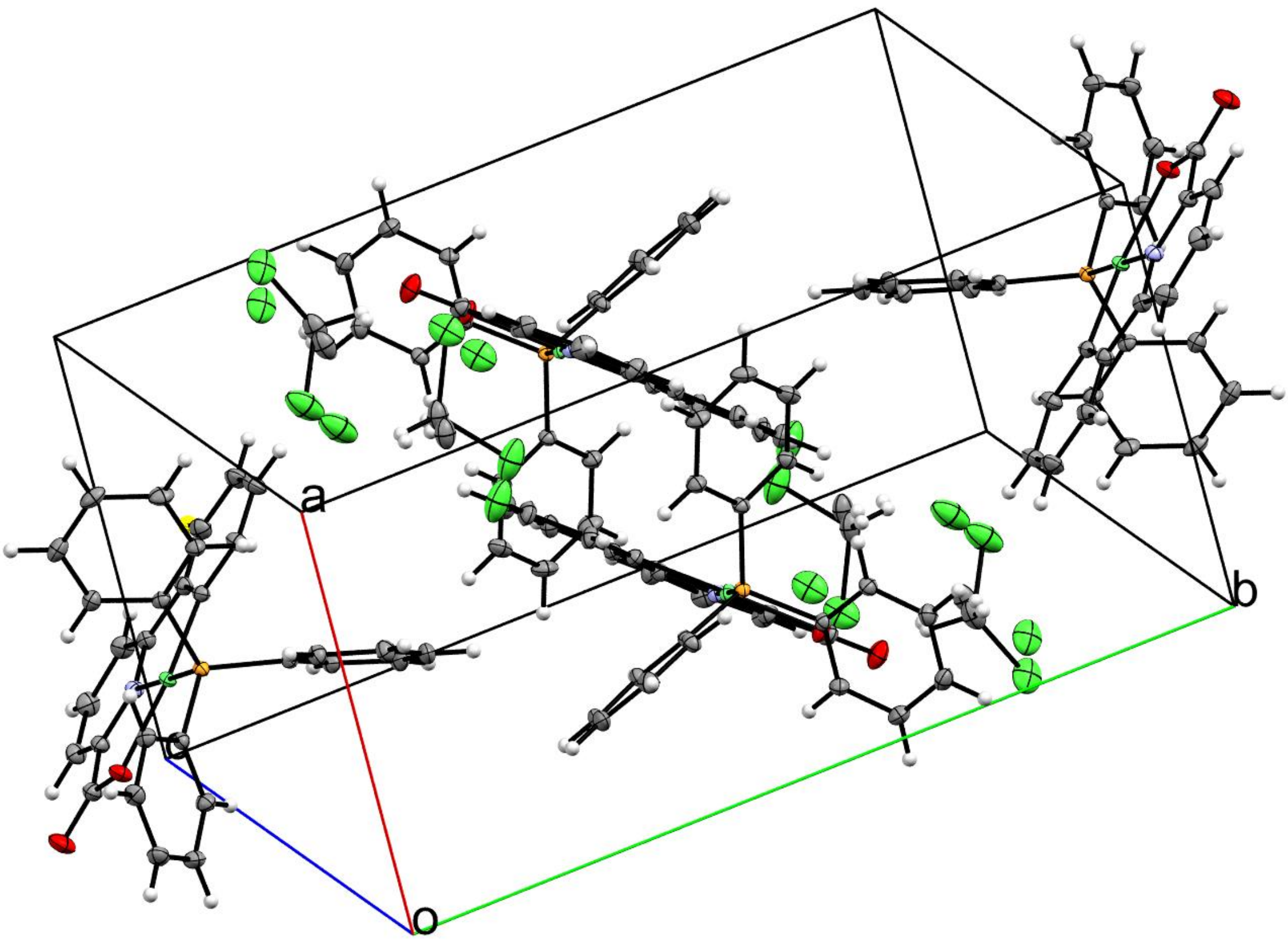

Figure S-278. Unit cell of **Pt(L_H)PPh_3**. Displacement ellipsoids are shown with 50% probability.

Table S-5. Selected bond lengths and angles in **Pt(L$_H$)PPh$_3$**.

**Bond length / angle**	
Pt1 – N2 / Å	1.9956(13)
Pt1 – C16 / Å	2.0013(14)
Pt1 – N17 / Å	2.1219(11)
Pt1 – O10 / Å	2.2304(4)
N2 ··· Pt1 ··· O10 / °	78.71(5)
N2 ··· Pt1 ··· C16 / °	81.25(6)
O10 ··· Pt1 ··· C16 / °	159.96(5)
N2 ··· Pt1 ··· P17 / °	177.82(3)
O10 ··· Pt1 ··· P17 / °	100.08(3)
C16 ··· Pt1 ··· P17 / °	99.95(5)
N2 ··· C3 ··· C8 / °	113.9(1)

Section S4.3: X-ray diffractometric results for single crystals of Pt(L_H)AmPy

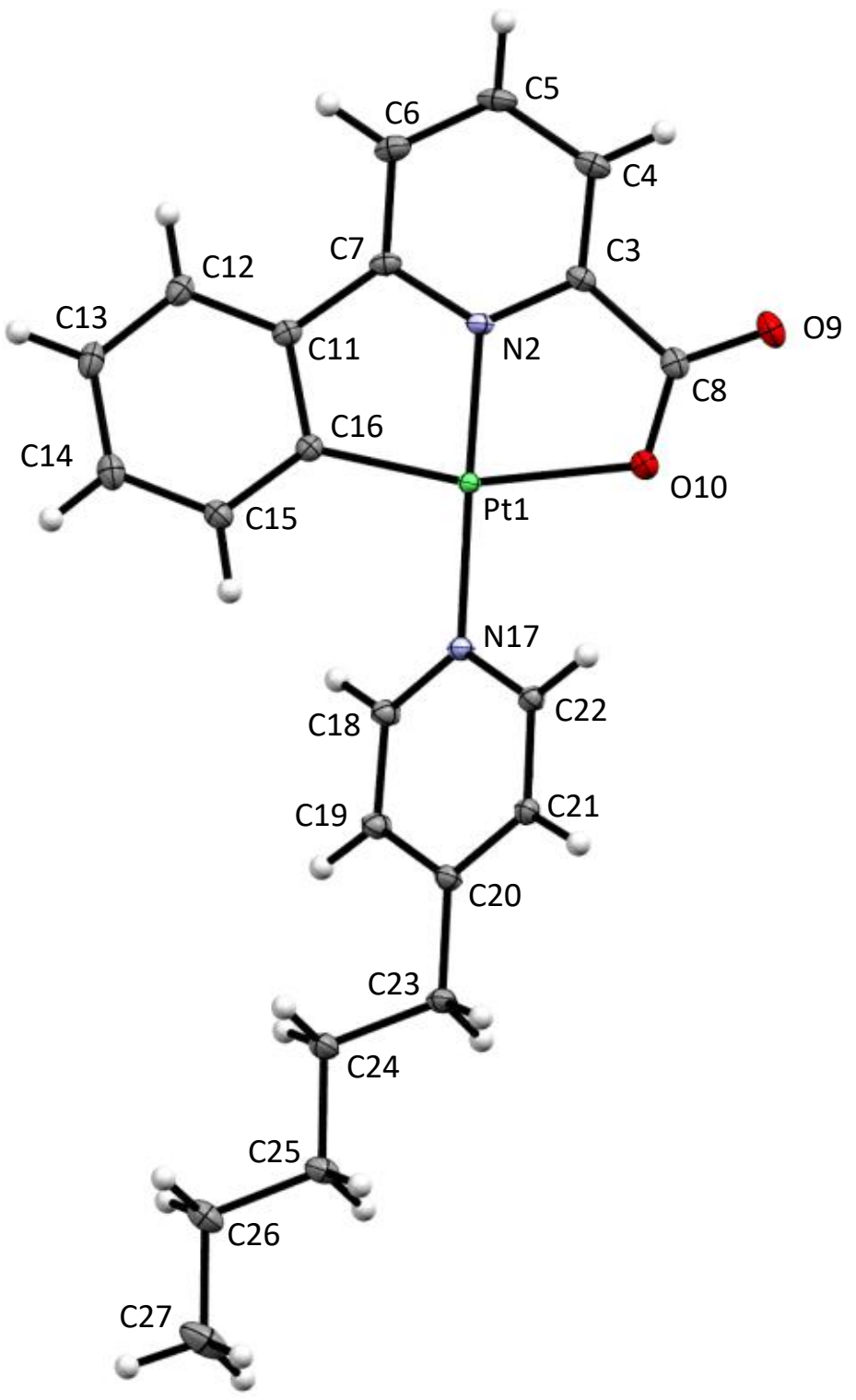

Figure S-279. Molecular structure of **Pt(L_H)AmPy** in the crystal with labelled atoms. Displacement ellipsoids are shown with 50% probability.

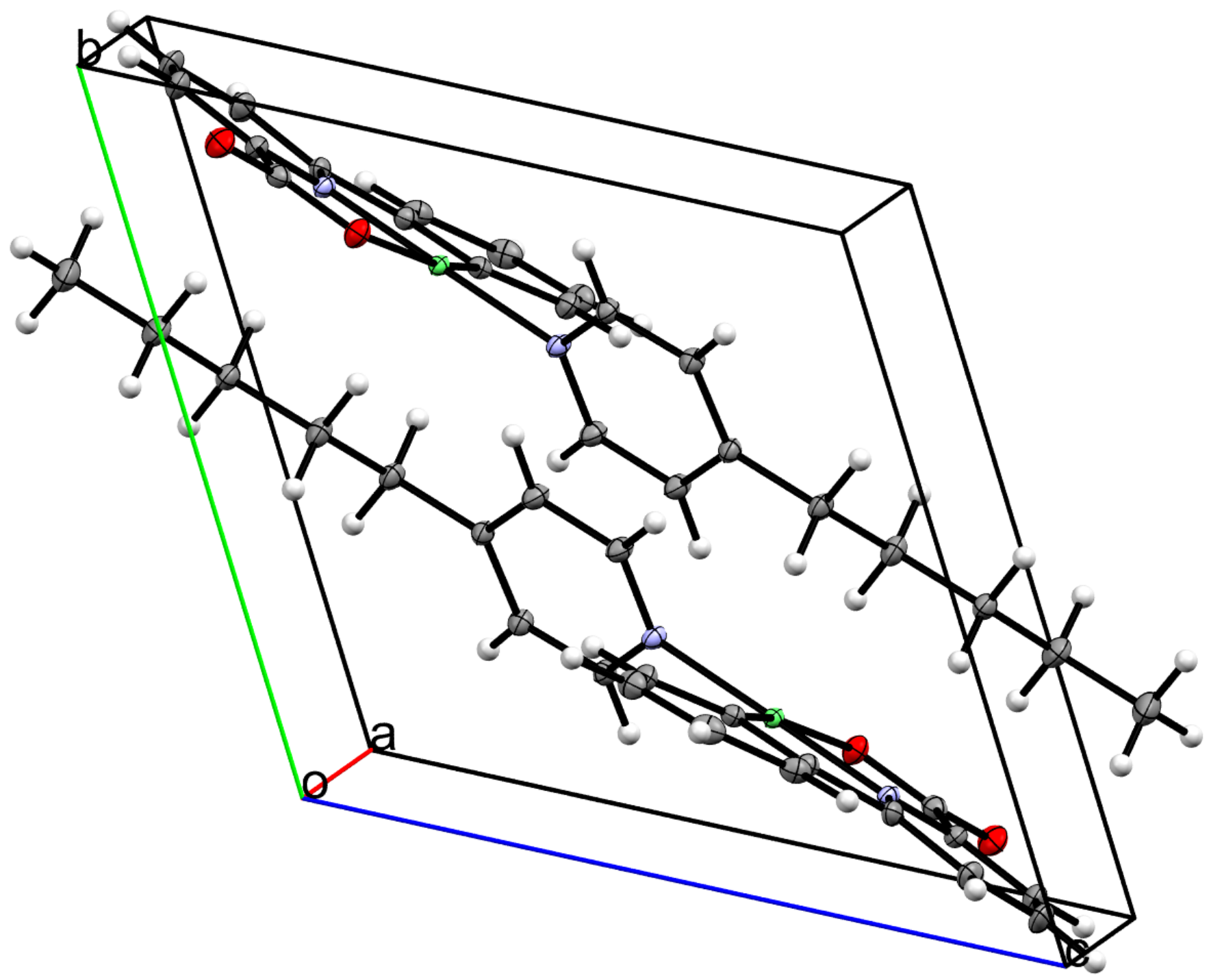

Figure S-280. Unit cell of **Pt(L_H)AmPy**. Displacement ellipsoids are shown with 50% probability.

Table S-6. Selected bond lengths and angles in **Pt(L_H)AmPy**.

**Bond length / angle**	
Pt1 – N2 / Å	1.9401(14)
Pt1 – C16 / Å	1.980(2)
Pt1 – N17 / Å	2.0258(18)
Pt1 – O10 / Å	2.1370(16)
N2 ⋯ Pt1 ⋯ C16 / °	82.18(8)
N17 ⋯ Pt1 ⋯ C16 / °	98.81(8)
O10 ⋯ Pt1 ⋯ C16 / °	161.78(8)
N2 ⋯ Pt1 ⋯ N17 / °	178.85(6)
N2 ⋯ Pt1 ⋯ O10 / °	79.63(7)
N17 ⋯ Pt1 ⋯ O10 / °	99.36(7)
N2 ⋯ C3 ⋯ C8 / °	115.4(2)

Section S4.4: X-ray diffractometric results for single crystals of Pt(L_{tBu})AmPy

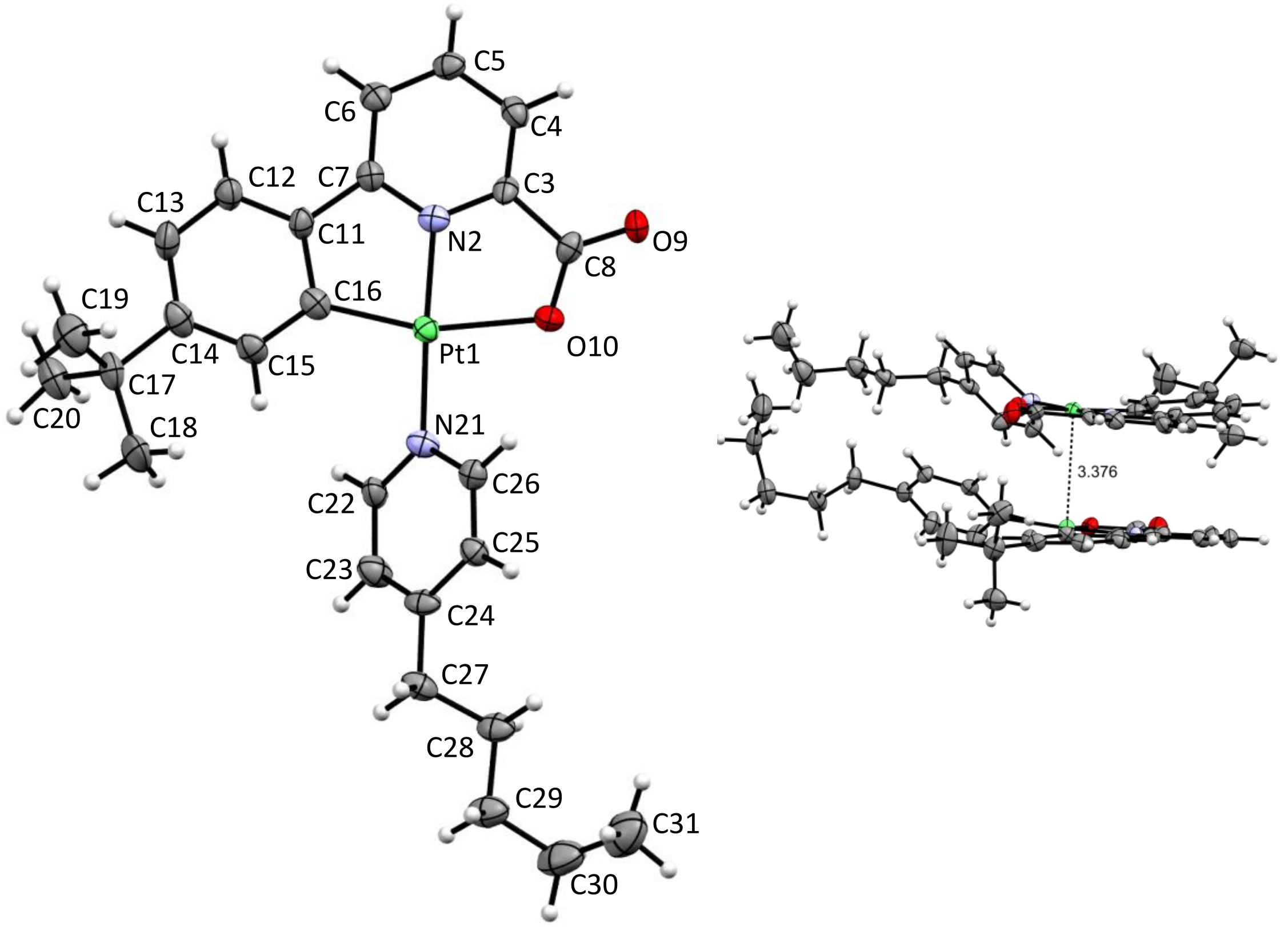

Figure S-281. Left: Molecular structure of **Pt(L_{tBu})AmPy** in the crystal with labelled atoms. Right: Metal-metal interactions within dimers (3.376 Å). Displacement ellipsoids are shown with 50% probability. Distance is given in angstrom (Å).

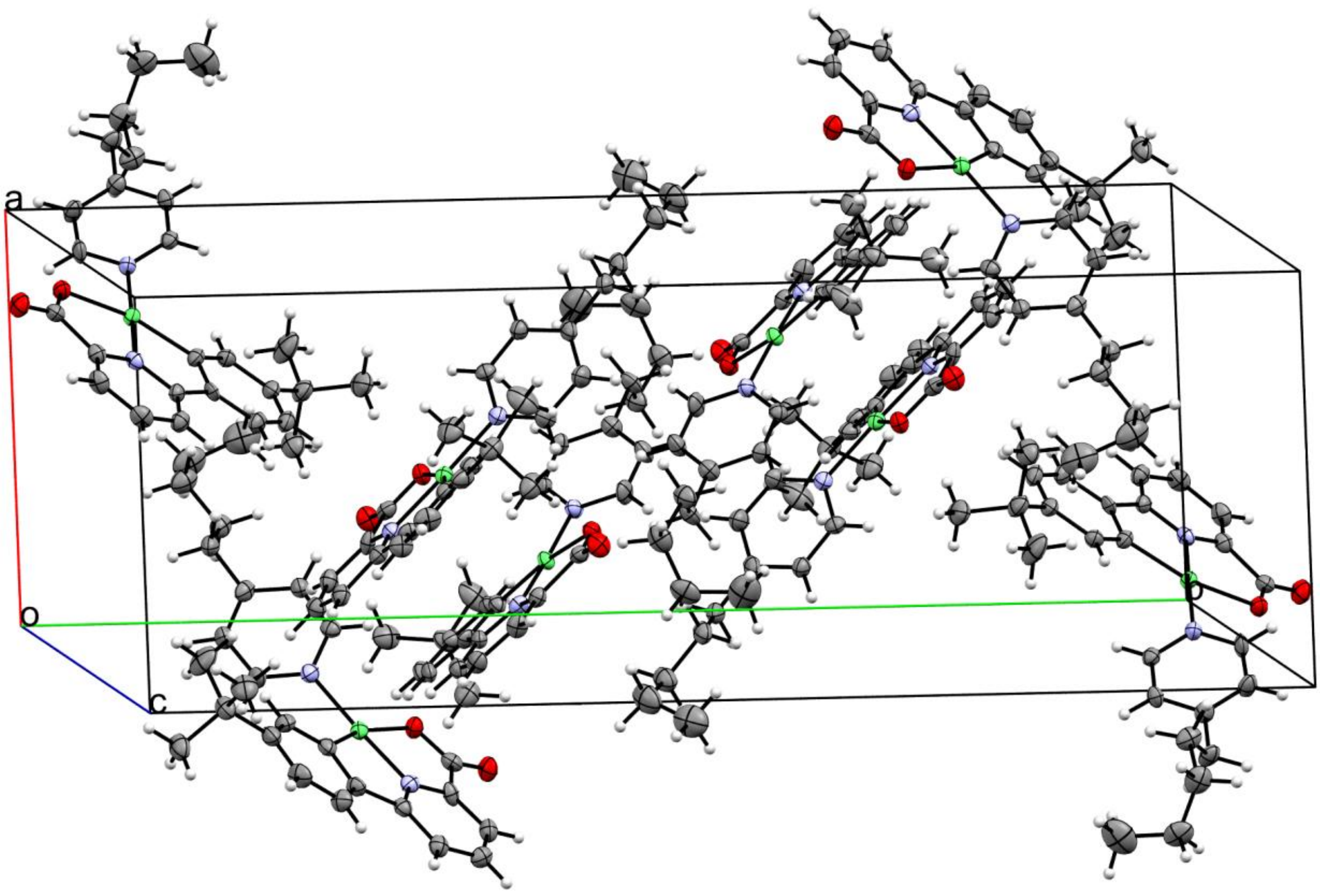

Figure S-282. Unit cell of **Pt(L_{tBu})AmPy**. Displacement ellipsoids are shown with 50% probability.

Table S-7. Selected bond lengths and angles of **Pt(L_{tBu})AmPy**.

**Bond length / angle**	
Pt1 – N2 / Å	1.953(4)
Pt1 – C16 / Å	1.977 (6)
Pt1 – O10 / Å	2.153(4)
Pt1 – N21 / Å	2.029(4)
N2 ⋯ Pt1 ⋯ 010 / °	80.5(2)
C16 ⋯ Pt1 ⋯ O10 / °	161.8(2)
C16 ⋯ Pt1 ⋯ N2 / °	81.7(5)
N2 ⋯ Pt1 ⋯ P20 / °	177.1(5)
C16 ⋯ Pt1 ⋯ P20 / °	100.7(2)
O10 ⋯ Pt1 ⋯ P20 / °	97.0(2)
N2 ⋯ C3 ⋯ C8 / °	115.3(5)

Section S4.5: X-ray diffractometric results for single crystals of Pt(L_{DiF})PPh_3

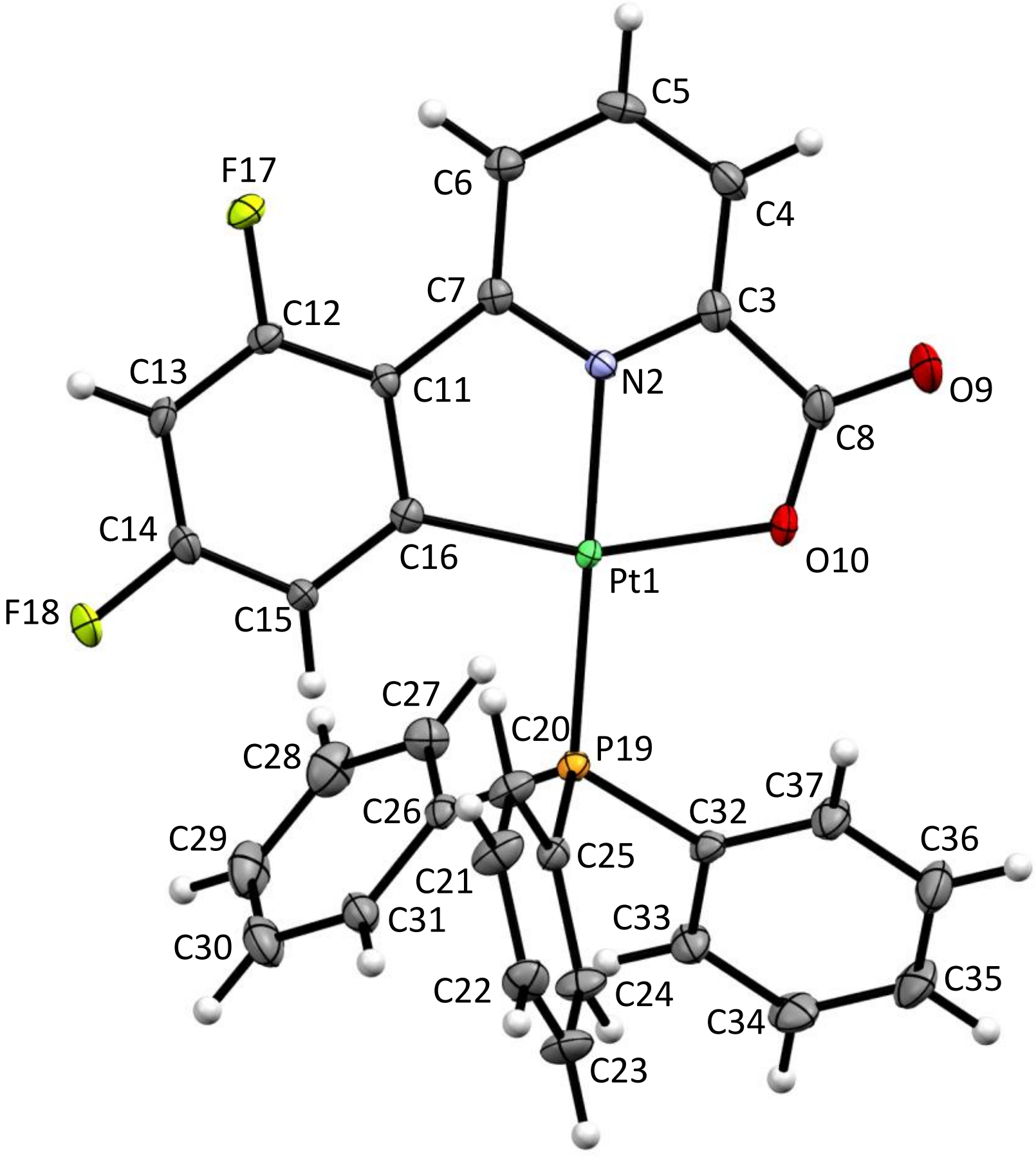

Figure S-283. Molecular structure of **Pt(L_{DiF})PPh_3** in the crystal with labelled atoms. Displacement ellipsoids are shown with 50% probability. Solvent molecules were omitted for clarity.

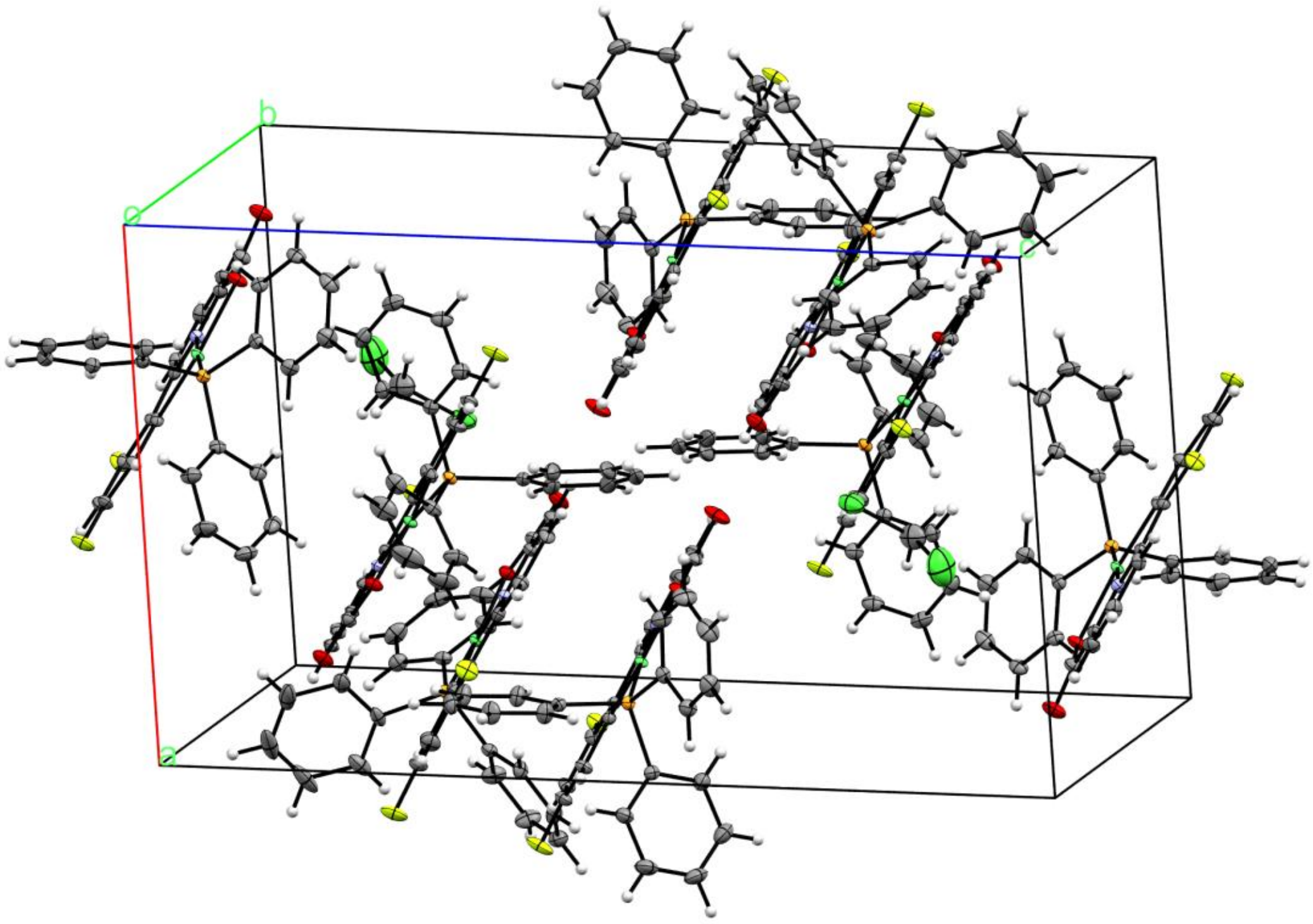

Figure S-284. Unit cell of **Pt(L_{DiF})PPh_3**. Displacement ellipsoids are shown with 50% probability.

Table S-8. Selected bond lengths and angles of **Pt(L_{DiF})PPh_3**.

**Bond length / angle**	
Pt1 – N2 / Å	1.996(2)
Pt1 – C16 / Å	1.999(2)
Pt1 – O10 / Å	2.109(2)
Pt1 – P20 / Å	2.2394(8)
N2 ··· Pt1 ··· 010 / °	78.96(8)
C16 ··· Pt1 ··· O10 / °	160.55(9)
C16 ··· Pt1 ··· N2 / °	81.68(9)
N2 ··· Pt1 ··· P20 / °	177.13(7)
C16 ··· Pt1 ··· P20 / °	99.32(7)
O10 ··· Pt1 ··· P20 / °	100.10(6)
N2 ··· C3 ··· C8 / °	113.4(2)

Section S4.6: X-ray diffractometric results for single crystals of Pt(L_{pOMe})PPh_3

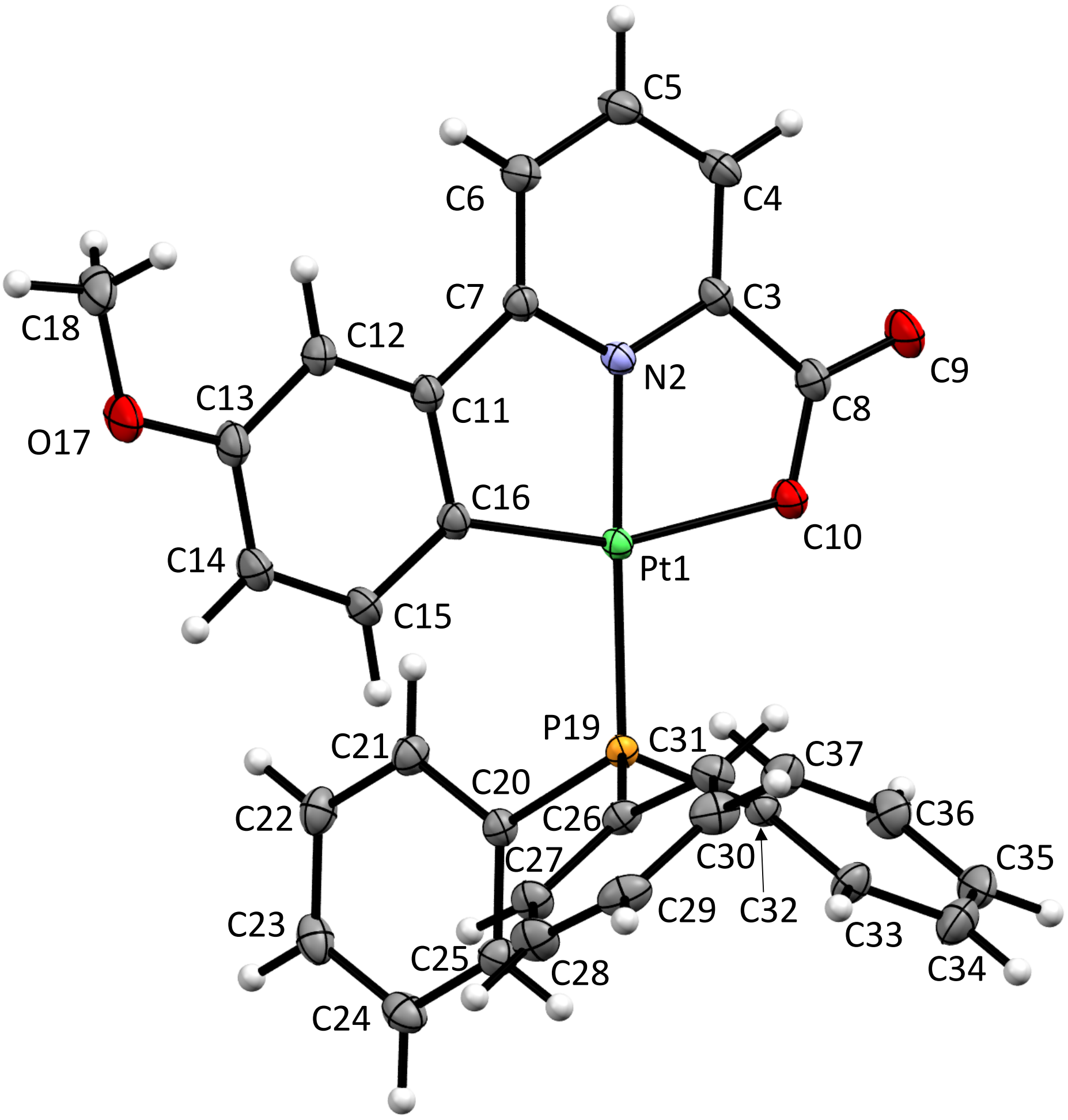

Figure S-285. Molecular structure of **Pt(L_{pOMe})PPh_3** in the crystal with labelled atoms. Displacement ellipsoids are shown with 50% probability. Solvent molecules were omitted for clarity.

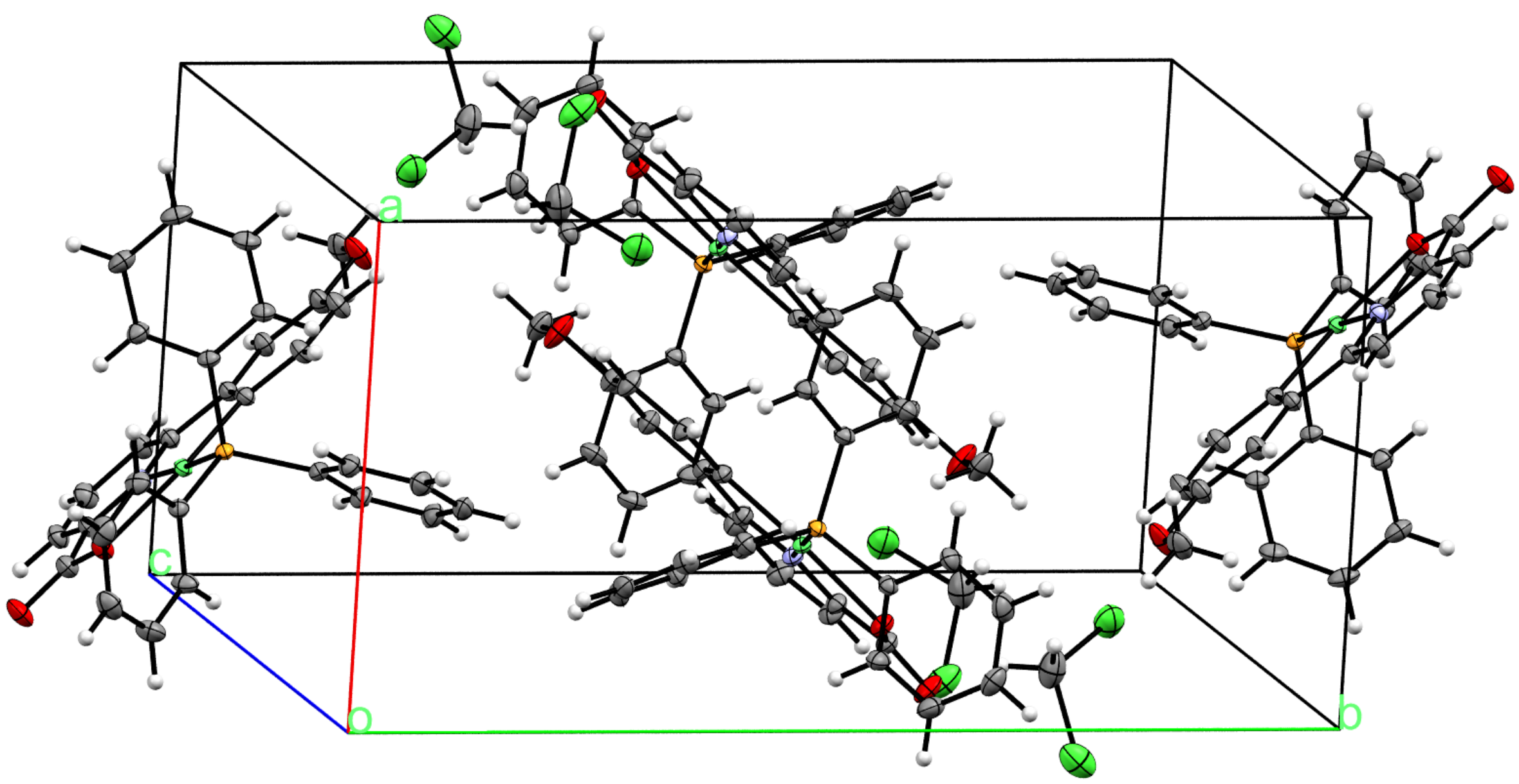

Figure S-286. Unit cell of **Pt(L_{pOMe})PPh_3**. Displacement ellipsoids are shown with 50% probability.

Table S-9. Selected bond lengths and angles of **Pt(L_{pOMe})PPh_3**.

**Bond length / angle**	
Pt1 – N2 / Å	1.999(1)
Pt1 – C16 / Å	2.000(2)
Pt1 – O10 / Å	2.132(2)
Pt1 – P20 / Å	2.2318(6)
N2 ··· Pt1 ··· 010 / °	78.45(5)
C16 ··· Pt1 ··· O10 / °	159.73(6)
C16 ··· Pt1 ··· N2 / °	81.28(6)
N2 ··· Pt1 ··· P20 / °	177.09(4)
C16 ··· Pt1 ··· P20 / °	99.08(5)
O10 ··· Pt1 ··· P20 / °	101.19(5)
N2 ··· C3 ··· C8 / °	113.5(1)

Section S4.7: X-ray diffractometric results for single crystals of Pd(L_{mOMe})PPh_3

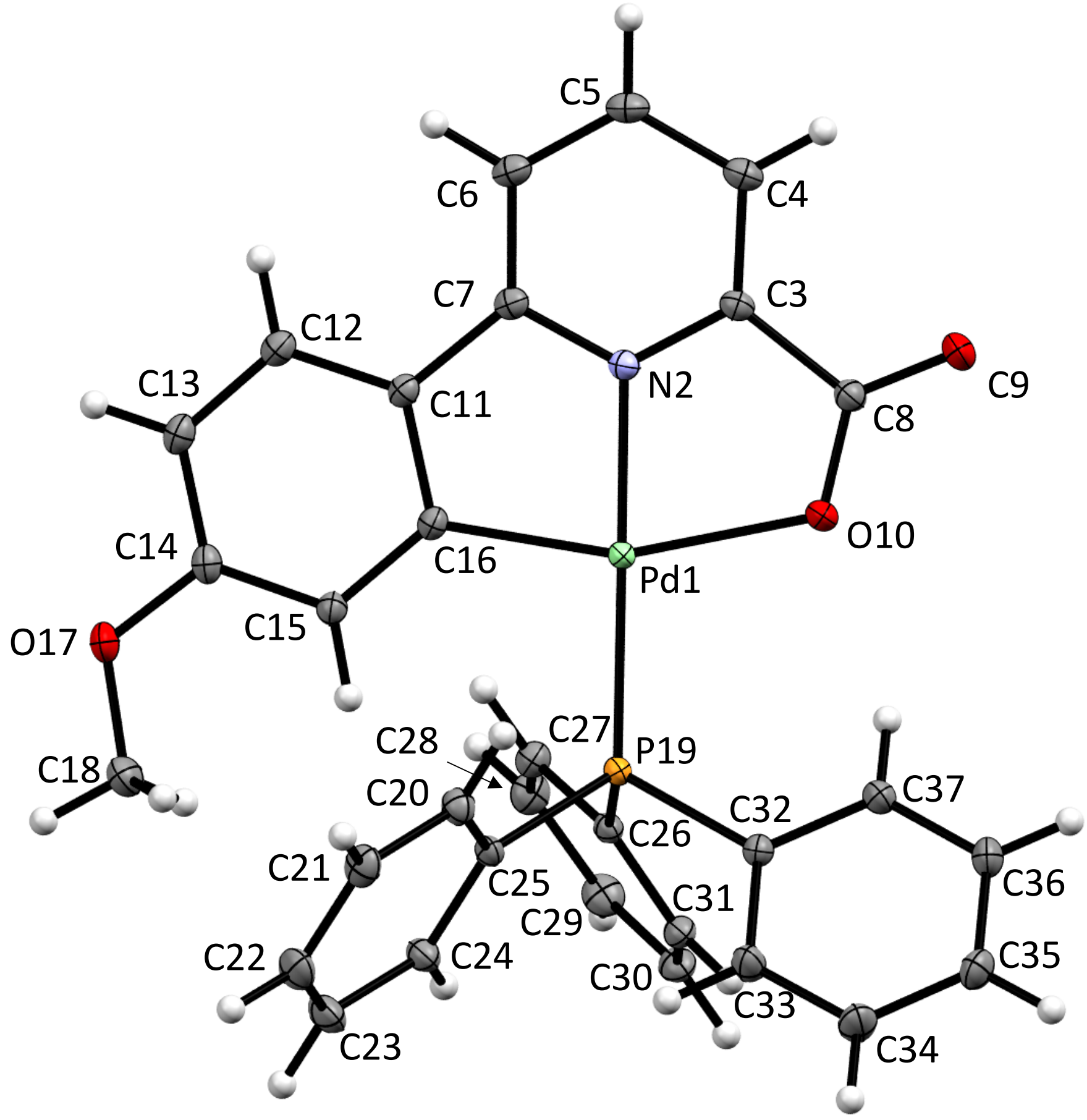

Figure S-287. Molecular structure of **Pd(L_{mOMe})PPh_3** in the crystal with labelled atoms. Displacement ellipsoids are shown with 50% probability.

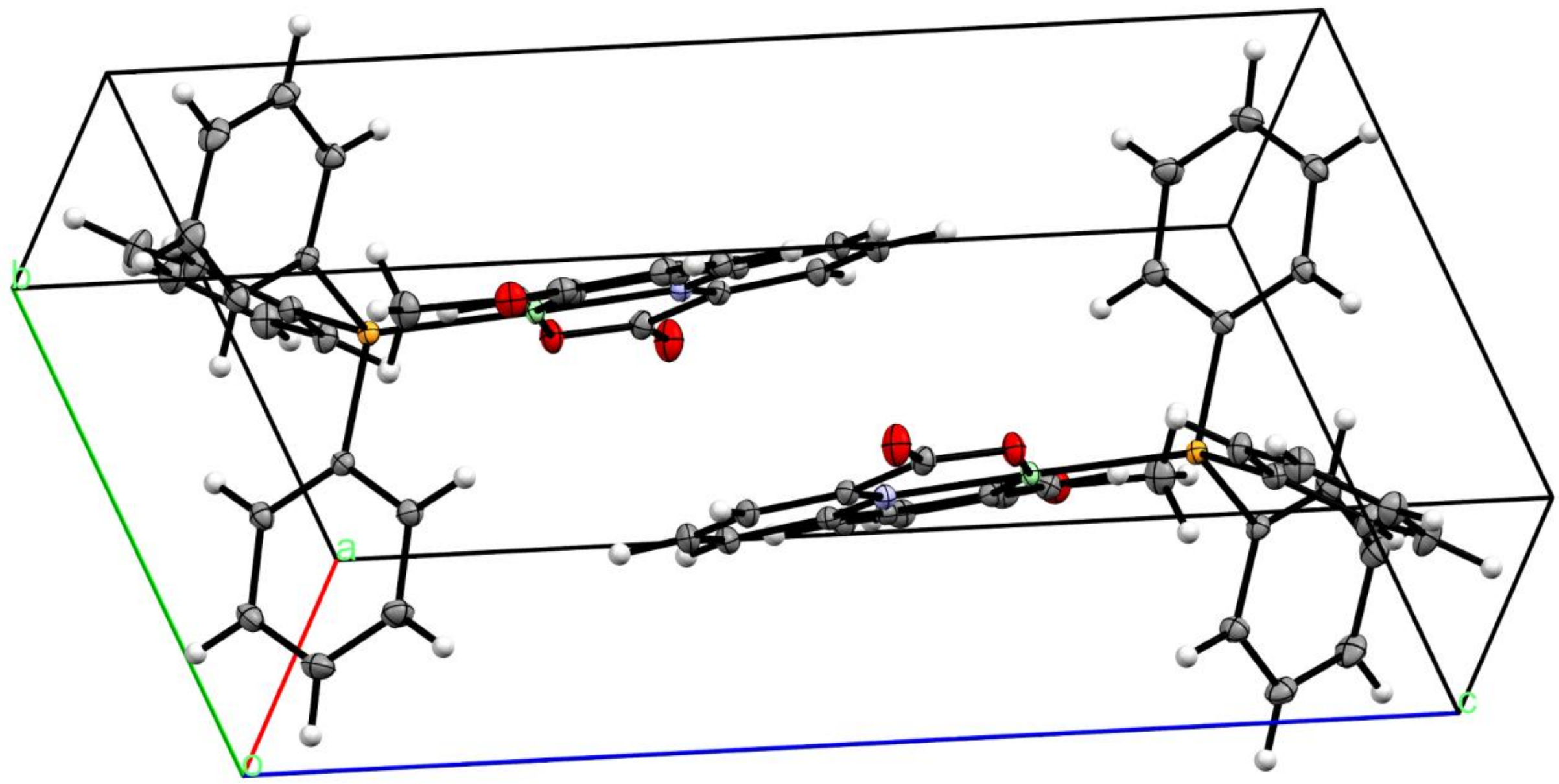

Figure S-288. Unit cell of **Pd(L_{mOMe})PPh_3**. Displacement ellipsoids are shown with 50% probability.

Table S-10. Selected bond lengths and angles of **Pd(L_{mOMe})PPh_3**.

Bond length / angle	
Pd1 – N2 / Å	1.9914(9)
Pd1 – C16 / Å	2.0106(11)
Pd1 – O10 / Å	2.1356(8)
Pd1 – P20 / Å	2.2630(3)
N2 ··· Pd1 ··· 010 / °	78.13(4)
C16 ··· Pd1 ··· O10 / °	158.91(4)
C16 ··· Pd1 ··· N2 / °	81.32(4)
N2 ··· Pd1 ··· P20 / °	178.83(3)
C16 ··· Pd1 ··· P20 / °	98.38(3)
O10 ··· Pd1 ··· P20 / °	102.25(2)
N2 ··· C3 ··· C8 / °	113.32(9)

Section S4.8: X-ray diffractometric results for single crystals of Pt(L_{mDMA})PPh_3

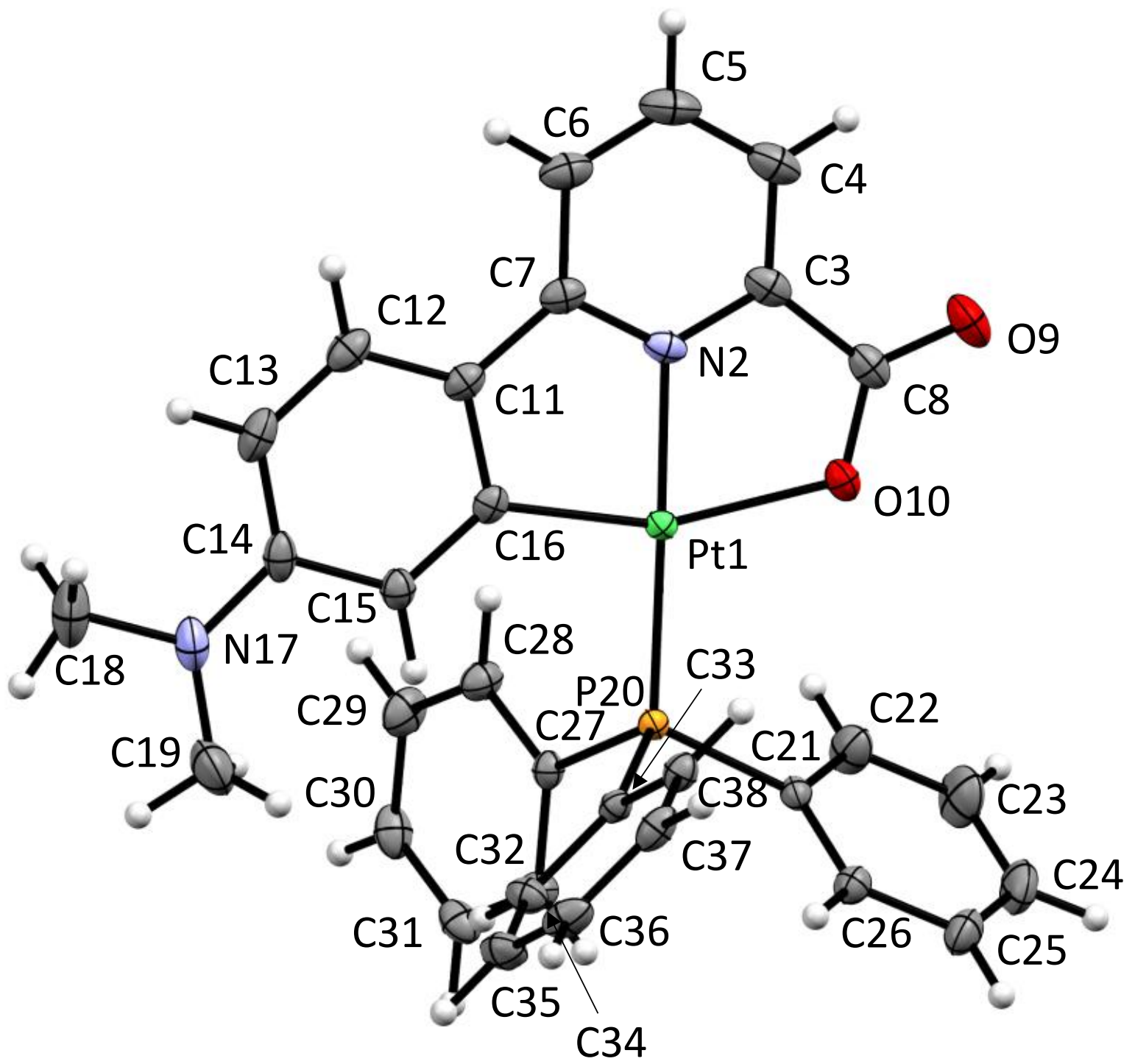

Figure S-289. Molecular structure of **Pt(L_{mDMA})PPh_3** in the crystal with labelled atoms. Displacement ellipsoids are shown with 50% probability.

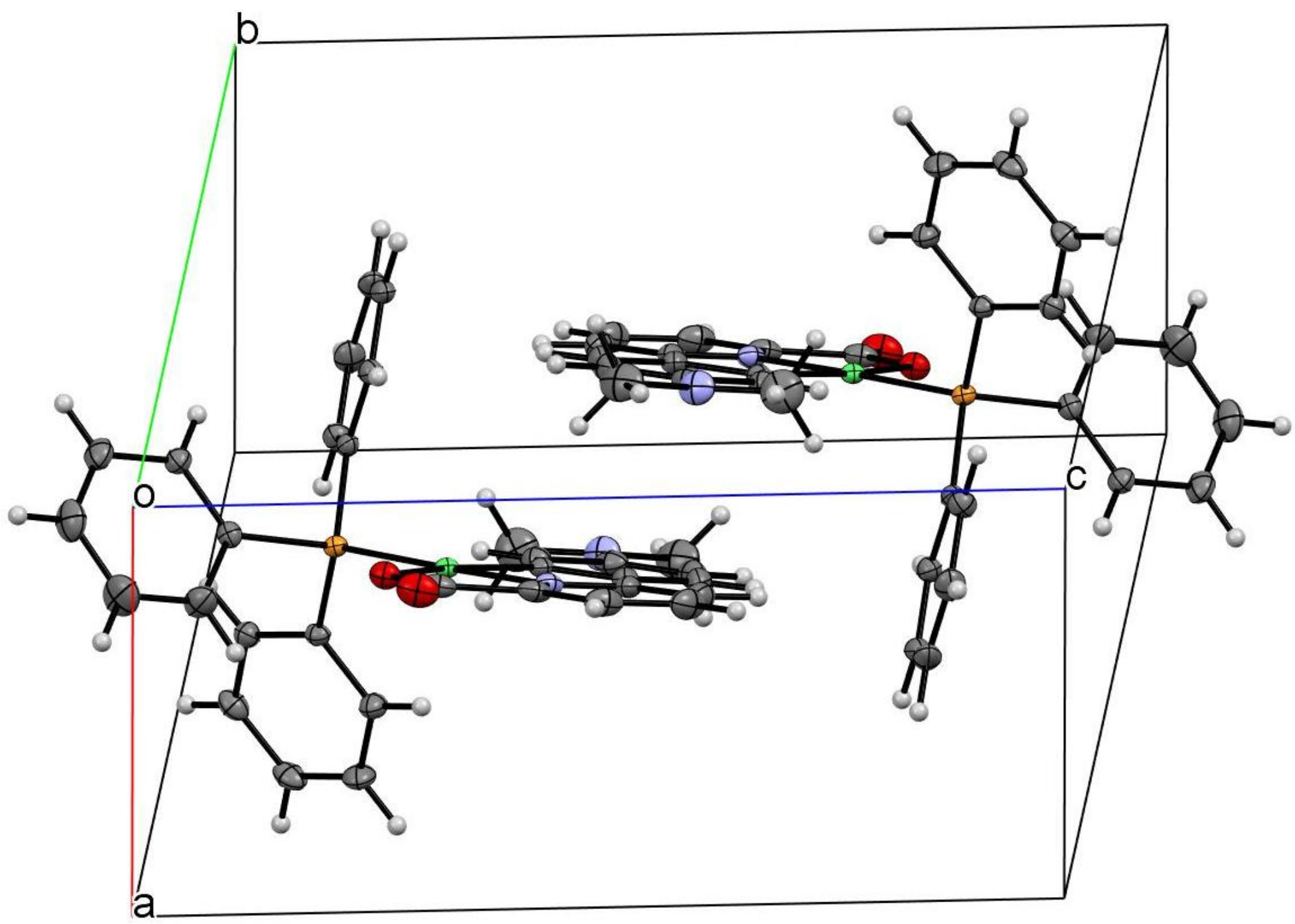

Figure S-290. Unit cell of **Pt(L_{mDMA})PPh_3**. Displacement ellipsoids are shown with 50% probability.

Table S-11. Selected bond lengths and angles of **Pt(L_{mDMA})PPh_3**.

**Bond length / angle**	
Pt1 – N2 / Å	1.9958(18)
Pt1 – C16 / Å	2.004(2)
Pt1 – O10 / Å	2.1317(6)
Pt1 – P20 / Å	2.2261(5)
N2 ··· Pt1 ··· 010 / °	78.39(7)
C16 ··· Pt1 ··· O10 / °	159.83(8)
C16 ··· Pt1 ··· N2 / °	82.51(5)
N2 ··· Pt1 ··· P20 / °	178.11(5)
C16 ··· Pt1 ··· P20 / °	97.11(6)
O10 ··· Pt1 ··· P20 / °	103.06(5)
N2 ··· C3 ··· C8 / °	113.49(19)

Section S4.9: X-ray diffractometric results for single crystals of Pt(L_{pDMA})PPh_3

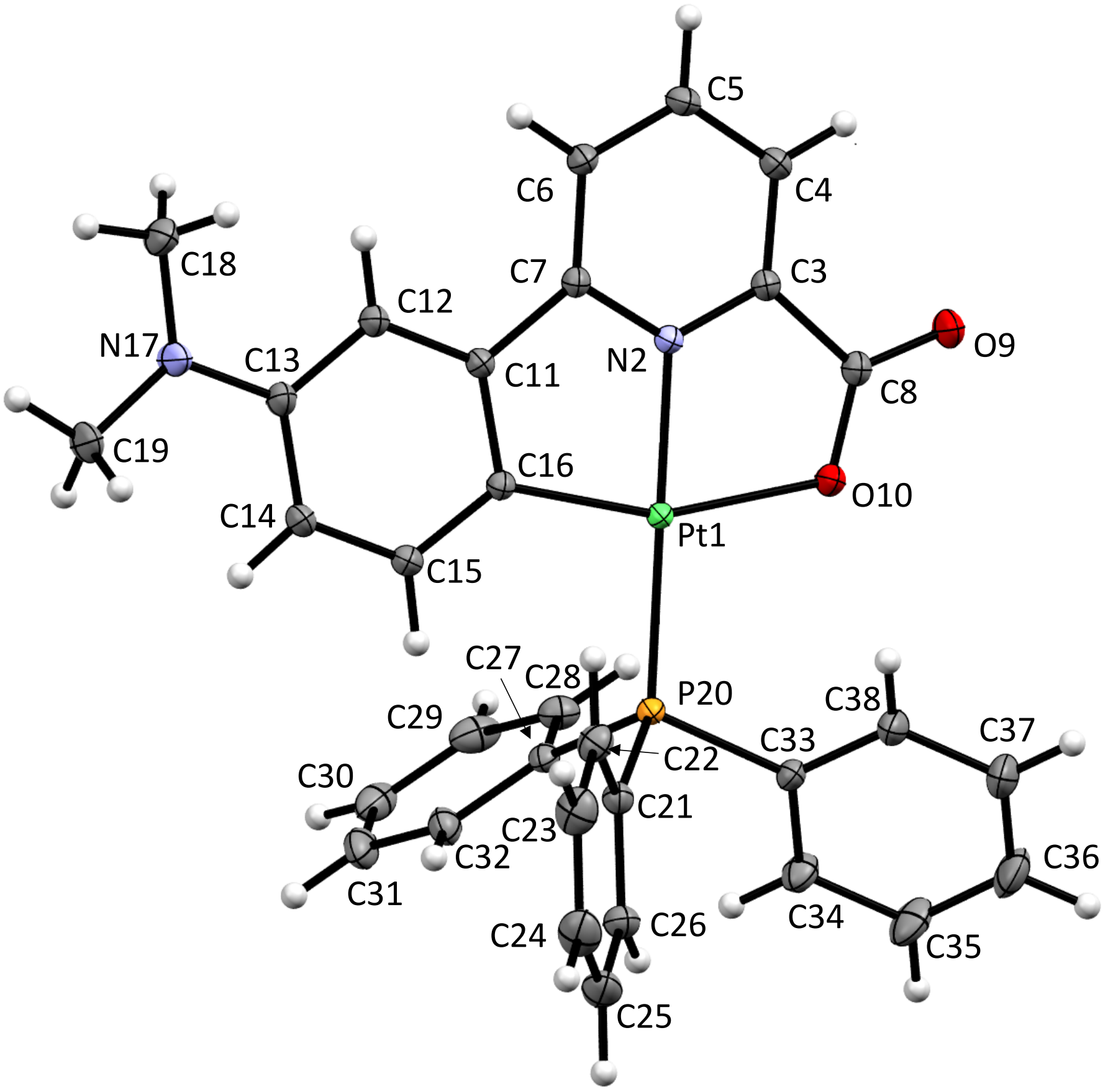

Figure S-291. Molecular structure of **Pt(L_{pDMA})PPh_3** in the crystal with labelled atoms. Displacement ellipsoids are shown with 50% probability. Solvent molecules were omitted for clarity.

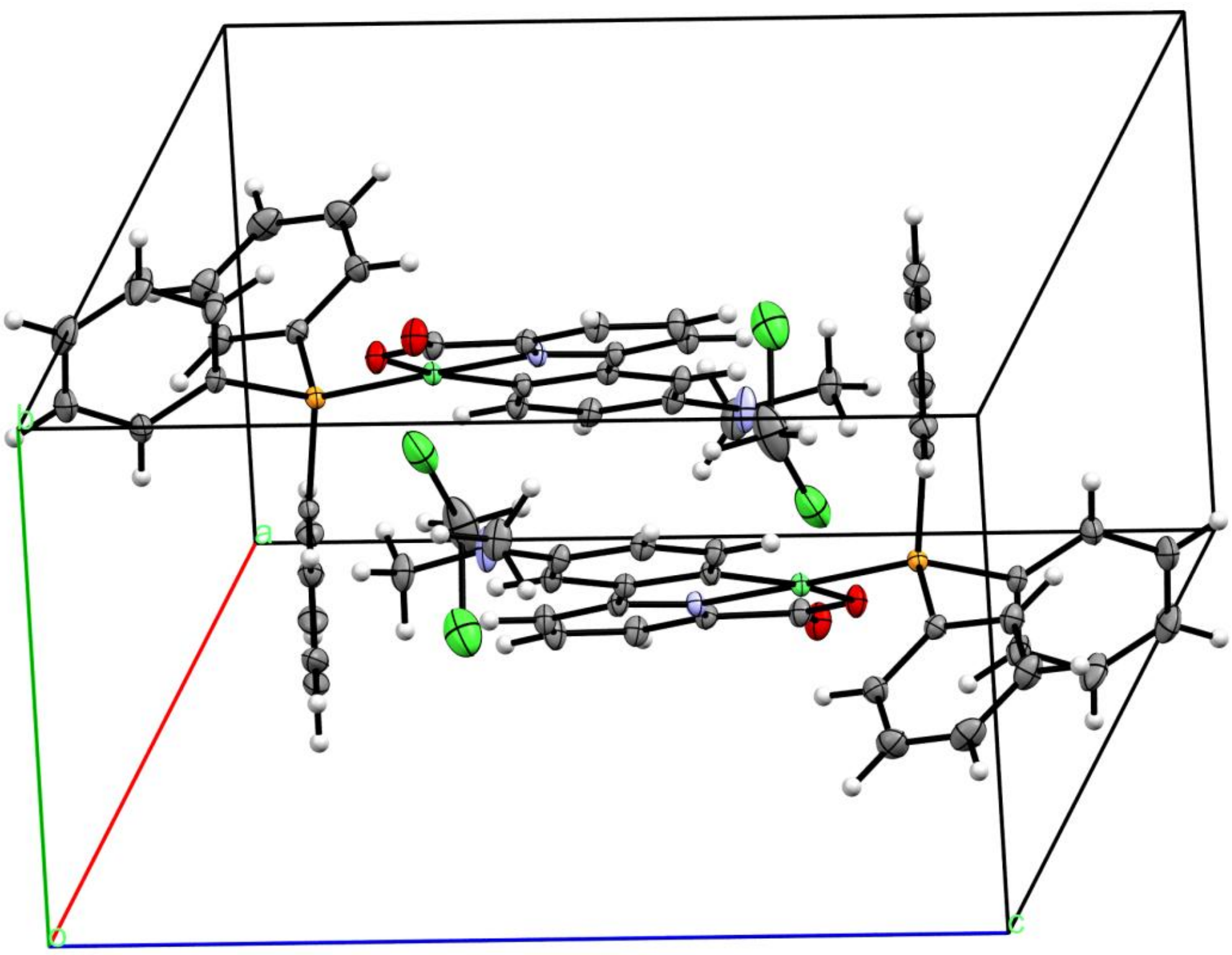

Figure S-292. Unit cell of **Pt(L_{pDMA})PPh_3**. Displacement ellipsoids are shown with 50% probability.

Table S-12. Selected bond lengths and angles of **Pt(L$_{pDMA}$)PPh$_3$**.

**Bond length / angle**	
Pt1 – N2 / Å	1.997(1)
Pt1 – C16 / Å	1.998(1)
Pt1 – O10 / Å	2.138(1)
Pt1 – P20 / Å	2.2249(5)
N2 ··· Pt1 ··· 010 / °	78.21(7)
C16 ··· Pt1 ··· O10 / °	159.43(5)
C16 ··· Pt1 ··· N2 / °	81.27(6)
N2 ··· Pt1 ··· P20 / °	178.80(4)
C16 ··· Pt1 ··· P20 / °	99.28(4)
O10 ··· Pt1 ··· P20 / °	101.21(5)
N2 ··· C3 ··· C8 / °	113.5(1)

Section S5: Photophysical characterization

Table S-13: Complete set of photophysical data for the Pt(II) complexes with a NCO ligand motif.

		λ_{abs} (nm) / ε (10^4 $M^{-1}cm^{-1}$)	λ_{exc} (nm)	λ_{em} (nm)	τ (µs)		Φ_L (%)		$k_{r(RT, Ar)}$ / 10^4 s^{-1}	$k_{nr(RT, Ar)}$ / 10^4 s^{-1}
					air	*Ar*	*air*	*Ar*		
**Pt(L$_{NCO}$)Cl**	liquid solution (298 K, DCM, 10^{-5} M)	267 / 2.26<br>344 / 0.44<br>379 / 0.53	292, 383	499, 530	0.278 ± 0.001	2.580 ± 0.001	2 ± 2	44 ± 4	17.1 ± 0.8	21.7 ± 0.8
	glassy matrix (DCM/MeOH, 77 K, 10^{-7} M)		291, 354	470, 503, 537, 582	9.08 ± 0.03<br>[10.9 ± 1.3 (49 %)<br>7 ± 1 (51 %)]		n.d.		n.d.	n.d.
**Pt(L$_{NCO}$)PPh$_3$**	liquid solution (298 K, DCM, 10^{-5} M)	265 / 2.20<br>311 / 1.14	n.d.	n.d.	n.d.	n.d.	n.d.	n.d.	n.d.	n.d.
	glassy matrix (DCM/MeOH, 77 K, 10^{-7} M)		299, 350	465, 493, 531, 579	14.84 ± 0.03		n.d.		n.d.	n.d.
**Pt(L$_{NCO}$)AmPy**	liquid solution (298 K, DCM, 10^{-5} M)	265 / 2,16<br>285 / 1.79<br>364 / 0.63	290, 368	483, 517, 551 (shoulder)	0.831 ± 0.001	5.849 ± 0.001	7 ± 2	76 ± 4	13 ± 0.5	4.1 ± 0.3
	glassy matrix (DCM/MeOH, 77 K, 10^{-7} M)		340	466, 499, 537	9.65 ± 0.02		n.d.		n.d.	n.d.

Table S-14: Complete set of photophysical data for the Pt(II) complexes with a CNO ligand motif.

		λ_{abs} (nm) / ε (10^4 $M^{-1}cm^{-1}$)	λ_{exc} (nm)	λ_{em} (nm)	τ (µs)		Φ_L (%)		$k_{r(RT, Ar)}$ / 10^4 s^{-1}	$k_{nr(RT, Ar)}$ / 10^4 s^{-1}
					air	*Ar*	*air*	*Ar*		
**Pt(L$_H$)Cl**	liquid solution (298 K, DCM, 10^{-5} M)	246 / 1.81<br>262 / 1.85<br>294 / 0.90	391	515, 532	0.089 ± 0.001<br>[0.145 ± 0.001 (28 %)<br>0.067 ± 0.001 (72 %)]	8.958 ± 0.009<br>[9.68 ± 0.01 (87 %)<br>3.9 ± 0.1 (13 %)]	< 2	56 ± 3	6.2 ± 0.2	4.9 ± 0.2
	glassy matrix (DCM/MeOH, 77 K, 10^{-7} M)		375	499, 535, 573, 615	9.92 ± 0.02<br>[4.9 ± 0.5 (91 %)<br>10.42 ± 0.04 (9 %)]		n.d.		n.d.	n.d.
**Pt(L$_H$)PPh$_3$**	liquid solution (298 K, DCM, 10^{-5} M)	259/ 2.32<br>300 / 0.97<br>340 / 0.59	333, 391	497, 524, 563 (shoulder)	0.477 ± 0.001	6.960 ± 0.006	3 ± 2	42 ± 4	6.0 ± 0.3	8.3 ± 0.3
	glassy matrix (DCM/MeOH, 77 K, 10^{-7} M)		322, 345, 381	486, 520, 554, 600	16.43 ± 0.02		n.d.		n.d.	n.d.
**Pt(L$_H$)AmPy**	liquid solution (298 K, DCM, 10^{-5} M)	247 / 2.32<br>266 / 2.62<br>316 / 0.73		499	0.359 ± 0.001	3.02 ± 0.02<br>[4.263 ± 0.005 (68 %)<br>0.352 ± 0.008 (32 %)]	4 ± 2	45 ± 4	6.5 ± 0.5	17.6 ± 0.5
	glassy matrix (DCM/MeOH, 77 K, 10^{-7} M)		307, 348	487, 521, 560, 601	10.71 ± 0.01		n.d.		n.d.	n.d.
**Pt(L$_H$)NHC**	liquid solution (298 K, DCM, 10^{-5} M)	268 / 2.44<br>322 / 0.83<br>352 / 0.54		500	0.350 ± 0.001	9.19 ± 0.02	3 ± 2	70 ± 4	7.6 ± 0.2	3.3 ± 0.2
	glassy matrix (DCM/MeOH, 77 K, 10^{-7} M)		293, 348	484, 513, 554, 594	11.001 ± 0.009		n.d.		n.d.	n.d.
**Pt(L$_{tBu}$)Cl**	liquid solution (298 K, DCM, 10^{-5} M)	253 / 1.11<br>295 / 0.91	352	503, 536, 577 (shoulder)	0.192 ± 0.002<br>[0.317 ± 0.004 (47 %)<br>0.082 ± 0.004 (53 %)]	4.409 ± 0.006<br>[5.2 ± 0.1 (54 %)<br>2.8 ± 0.1 (46 %)]	< 2	60 ± 3	15.2 ± 0.6	10.1 ± 0.1
	glassy matrix (DCM/MeOH, 77 K, 10^{-7} M)		318, 360	498, 534, 568	10.77 ± 0.02		n.d.		n.d.	n.d.
**Pt(L$_{tBu}$)PPh$_3$**	liquid solution (298 K, DCM, 10^{-5} M)	265 / 2.20<br>311 / 1.14	326, 395	500, 528, 571	0.530 ± 0.001	12.785 ± 0.005	< 2	60 ± 3	4.8 ± 0.2	3.2 ± 0.2
	glassy matrix (DCM/MeOH, 77 K, 10^{-7} M)		324, 347, 389	485, 518, 554	18.84 ± 0.01		n.d.		n.d.	n.d.

		λ_{abs} (nm) / ε (10^4 $M^{-1}cm^{-1}$)	λ_{exc} (nm)	λ_{em} (nm)	τ (µs)		Φ_L (%)		$k_{r(RT, Ar)}$ / 10^4 s^{-1}	$k_{nr(RT, Ar)}$ / 10^4 s^{-1}
					air	*Ar*	*air*	*Ar*		
**Pt(L$_{tBu}$)AmPy**	liquid solution (298 K, DCM, 10^{-5} M)	247 / 2.12<br>268 / 2.56<br>299 / 1.27	354	504, 537, 575 (shoulder)	0.345 ± 0.001	5.442 ± 0.005	3 ± 2	44 ± 4	9.6 ± 0.4	12.2 ± 0.4
	glassy matrix (DCM/MeOH, 77 K, 10^{-7} M)		332	492, 526, 558, 608	11.999 ± 0.009		n.d.		n.d.	n.d.
**Pt(L$_{tBu}$)NHC**	liquid solution (298 K, DCM, 10^{-5} M)	249 / 2.21<br>263 / 2.26<br>283 / 1.98	297, 349	492, 525, 558 (shoulder)	0.316 ± 0.001	9.336 ± 0.006	3 ± 2	75 ± 4	9.7 ± 0.2	1.0 ± 0.2
	glassy matrix (DCM/MeOH, 77 K, 10^{-7} M)		292, 349	484, 516, 552, 604	10.98 ± 0.01		n.d.		n.d.	n.d.
**Pt(L$_{DiF}$)PPh$_3$**	liquid solution (298 K, DCM, 10^{-5} M)	257 / 2.63<br>306 / 0.96<br>347 / 0.48	333, 390	475, 504, 542 (shoulder)	0.314 ± 0.001	0.551 ± 0.001	< 2	5 ± 2	9 ± 4	172 ± 4
	glassy matrix (DCM/MeOH, 77 K, 10^{-7} M)		333, 344, 375	468, 501, 534, 581	15.81 ± 0.02		n.d.		n.d.	n.d.
**Pt(L$_{mOMe}$)PPh$_3$**	liquid solution (298 K, DCM, 10^{-5} M)	268 / 2.19<br>324 / 1.49<br>386 / 0.14	326, 397	506, 538	0.385 ± 0.001	18.49 ± 0.03	< 2	54 ± 3	2.9 ± 0.1	2.5 ± 0.1
	glassy matrix (DCM/MeOH, 77 K, 10^{-7} M)		320, 407	500, 522, 533	25.37 ± 0.07		95 ± 5		3.7 ± 0.1	0.2 ± 0.1
**Pt(L$_{mOMe}$)NHC**	liquid solution (298 K, DCM, 10^{-5} M)	273 / 2.37<br>320 / 1.11<br>392 / 0.10	350, 395	505, 529	0.416 ± 0.001	14.43 ± 0.01	< 2	64 ± 3	4.4 ± 0.1	2.5 ± 0.1
	glassy matrix (DCM/MeOH, 77 K, 10^{-7} M)		320	480, 512	18.57 ± 0.03<br>[19.63 ± 0.6 (88 %)<br>10.9 ± 0.4 (12 %)]		95 ± 5		5.1 ± 0.1	0.3 ± 0.1
**Pt(L$_{pOMe}$)PPh$_3$**	liquid solution (298 K, DCM, 10^{-5} M),	257 / 2.52<br>308 / 0.96<br>360 / 0.38	326, 397	572	0.305 ± 0.001	13.339 ± 0.009	< 2	46 ± 5	2.8 ± 0.1	3.2 ± 0.1
	glassy matrix (DCM/MeOH, 77 K, 10^{-7} M)		320, 357	535, 558	17.84 ± 0.01		n.d.		n.d.	n.d.

		λ_{abs} (nm) / ε (10^4 $M^{-1}cm^{-1}$)	λ_{exc} (nm)	λ_{em} (nm)	τ (µs)		Φ_L (%)		$k_{r(RT, Ar)}$ / 10^4 s^{-1}	$k_{nr(RT, Ar)}$ / 10^4 s^{-1}
					air	*Ar*	*air*	*Ar*		
**Pt(L_{mDMA})PPh_3**	liquid solution (298 K, DCM, 10^{-5} M)	266 / 1.92<br>356 / 1.98<br>442 / 0.44	362, 460	570	0.216 ± 0.001	4.566 ± 0.009<br>[10 ± 1 (2 %)<br>4.43 ± 0.03<br>(98 %)]	< 2	4 ± 2	0.7 ± 0.3	16.7 ± 0.4
	glassy matrix (DCM/MeOH, 77 K, 10^{-7} M)		360, 450	548, 575, 589	33.8 ± 0.2<br>[57.8 ± 0.5 (59 %)<br>21.2 ± 0.4 (41 %)]		15 ± 2		0.52 ± 0.08	2.93 ± 0.09
**Pt(L_{mDMA})NHC**	liquid solution (298 K, DCM, 10^{-5} M)	280 / 2.62<br>333 / 1.80<br>416 / 0.28	350, 421	550	0.423 ± 0.001	8.59 ± 0.04	< 2	6 ± 2	0.7 ± 0.2	10.9 ± 0.3
	glassy matrix (DCM/MeOH, 77 K, 10^{-7} M)		353, 420	531, 541	253 ± 3<br>[391 ± 4 (31 %)<br>52.9 ± 0.6 (69 %)]		21 ± 2		0.083 ± 0.009	0.312 ± 0.009
**Pt(L_{pDMA})PPh_3**	liquid solution (298 K, DCM, 10^{-5} M)	260 / 2.38<br>318 / 0.86<br>400 / 0.21	355, 420, 460	693	0.130 ± 0.001	0.542 ± 0.002	< 2	< 2	< 3.7	180.8 - 184.5
	glassy matrix (DCM/MeOH, 77 K, 10^{-7} M)		n.d.	n.d	3.82 ± 0.06<br>[5.7 ± 0.3 (49 %)<br>2.1 ± 0.2 (51 %)]		n.d.		n.d.	n.d.

Table S-15. Complete set of photophysical data for the Pd(II) complexes with a CNO ligand motif.

		λ_{abs} (nm) / ε (10^4 $M^{-1}cm^{-1}$)	λ_{exc} (nm)	λ_{em} (nm)	τ (µs)		Φ_L (%)		$k_{r(RT, Ar)}$ / 10^4 s^{-1}	$k_{nr(RT, Ar)}$ / 10^4 s^{-1}
					air	*Ar*	*air*	*Ar*		
**Pd(L$_{tBu}$)PPh$_3$**	liquid solution (298 K, DCM, 10^{-5} M)	247 / 3.50<br>276 / 1.93<br>328 / 0.63	n.d.	n.d.	n.d.	n.d.	n.d.	n.d	n.d.	n.d.
	glassy matrix (DCM/MeOH, 77 K, 10^{-7} M)		364	461, 494, 526, 563	197 ± 1<br>[359 ± 6 (37 %)<br>146 ± 9 (39 %)<br>35 ± 3 (24 %)]		n.d.		n.d.	n.d.
**Pd(L$_{tBu}$)NHC**	liquid solution (298 K, DCM, 10^{-5} M)	277 / 2.52<br>326 / 0.47<br>358 / 0.16	n.d.	n.d.	n.d.	n.d.	n.d.	n.d.	n.d.	n.d.
	glassy matrix (DCM/MeOH, 77 K, 10^{-7} M)		367	464, 494, 528, 570	302.3 ± 0.4<br>[318.6 ± 0.8 (90 %)<br>164 ± 8 (10 %)]		n.d.		n.d.	n.d.
**Pd(L$_{mOMe}$)PPh$_3$**	liquid solution (298 K, DCM, 10^{-5} M)	277 / 2.43<br>327 / 0.77<br>364 / 0.33	320, 407	500, 522, 533	n.d.	n.d.	n.d.	n.d.	n.d.	n.d.
	glassy matrix (DCM/MeOH, 77 K, 10^{-7} M)		310, 385	475, 504, 535	445.6 ± 0.9<br>[556 ± 2 (61 %)<br>252 ± 4 (39 %)]		95 ± 5		0.214 ± 0.006	0.012 ± 0.005
**Pd(L$_{mOMe}$)NHC**	liquid solution (298 K, DCM, 10^{-5} M)	276 / 2.32<br>327 / 0.55<br>366 / 0.25	350	504, 527	n.d.	10.9 ± 0.2<br>[13.5 ± 0.5 (69 %)<br>5.3 ± 0.9 (31 %)]	n.d.	< 2	< 0.2	9.0 - 9.2
	glassy matrix (DCM/MeOH, 77 K, 10^{-7} M)		293, 365	310, 385	592.6 ± 0.6		95 ± 5		0.161 ± 0.004	0.008 ± 0.003
**Pd(L$_{mDMA}$)PPh$_3$**	liquid solution (298 K, DCM, 10^{-5} M)	250 / 3.11<br>355 / 1.13<br>413 / 0.51	355, 420, 460	540	n.d.	3.28 ± 0.04<br>[6.7 ± 0.2 (29 %)<br>1.90 ± 0.08 (71 %)]	n.d.	< 2	< 0.6	31.0 - 31.6
	glassy matrix (DCM/MeOH, 77 K, 10^{-7} M)		362, 433	527	834 ± 4<br>[1150 ± 10 (51 %)<br>500 ± 20 (49 %)]		45 ± 5		0.054 ± 0.003	0.066 ± 0.002
**Pd(L$_{mDMA}$)NHC**	liquid solution (298 K, DCM, 10^{-5} M)	278 / 2.38<br>340 / 0.68<br>413 / 0.21	330, 395	503, 530	n.d.	11.26 ± 0.02	< 2	< 2	< 0.1	8.7 - 8.8
	glassy matrix (DCM/MeOH, 77 K, 10^{-7} M)		367, 234	518, 539	793 ± 3<br>[1026 ± 7 (65 %)<br>36 ± 2 (35 %)]		21 ± 2		0.026 ± 0.003	0.100 ± 0.003

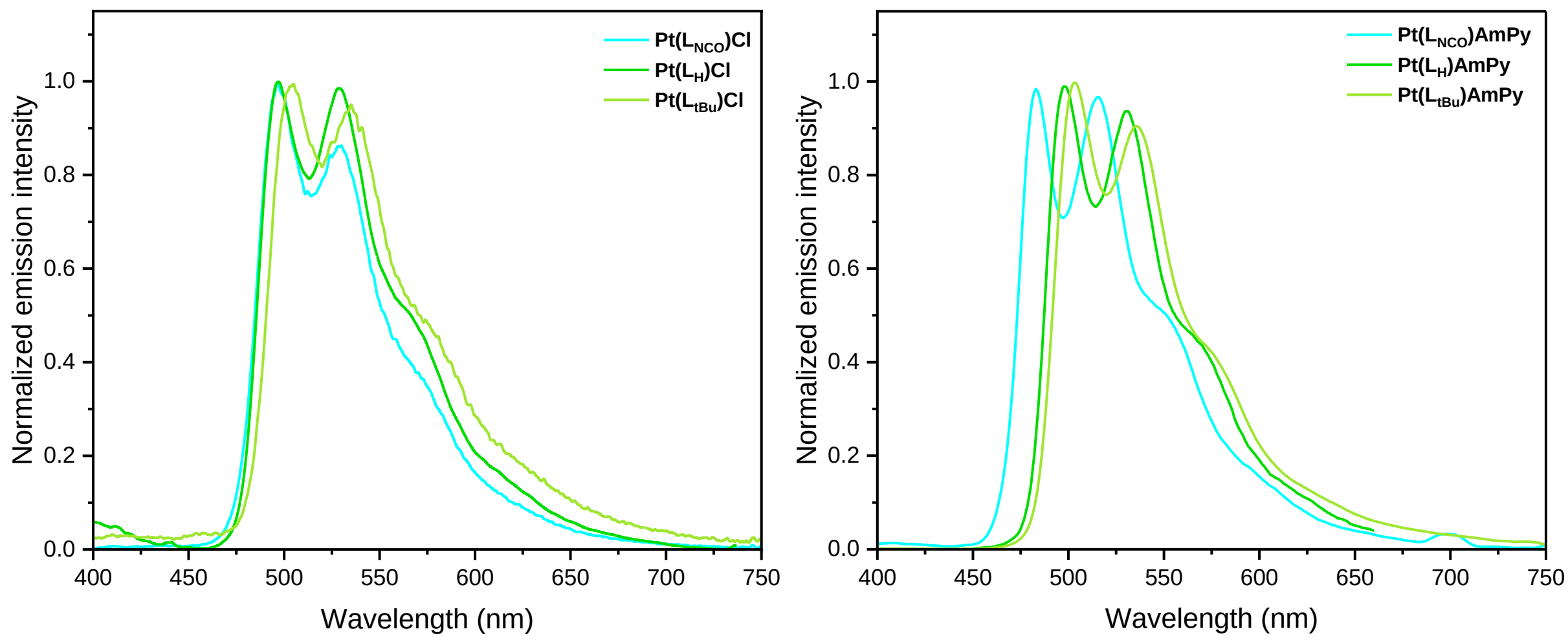

Figure S-293. Photoluminescence emission spectra (λ_{exc} = 350 nm) of **Pt(L$_{NCO}$)Cl**, **Pt(L$_{H}$)Cl** and **Pt(L$_{tBu}$)Cl** (left) and **Pt(L$_{NCO}$)AmPy**, **Pt(L$_{H}$)AmPy** and **Pt(L$_{tBu}$)AmPy** (right) at 298 K in liquid DCM (c = 10^{-5} M). Both spectra sets normalized to the highest intensity.

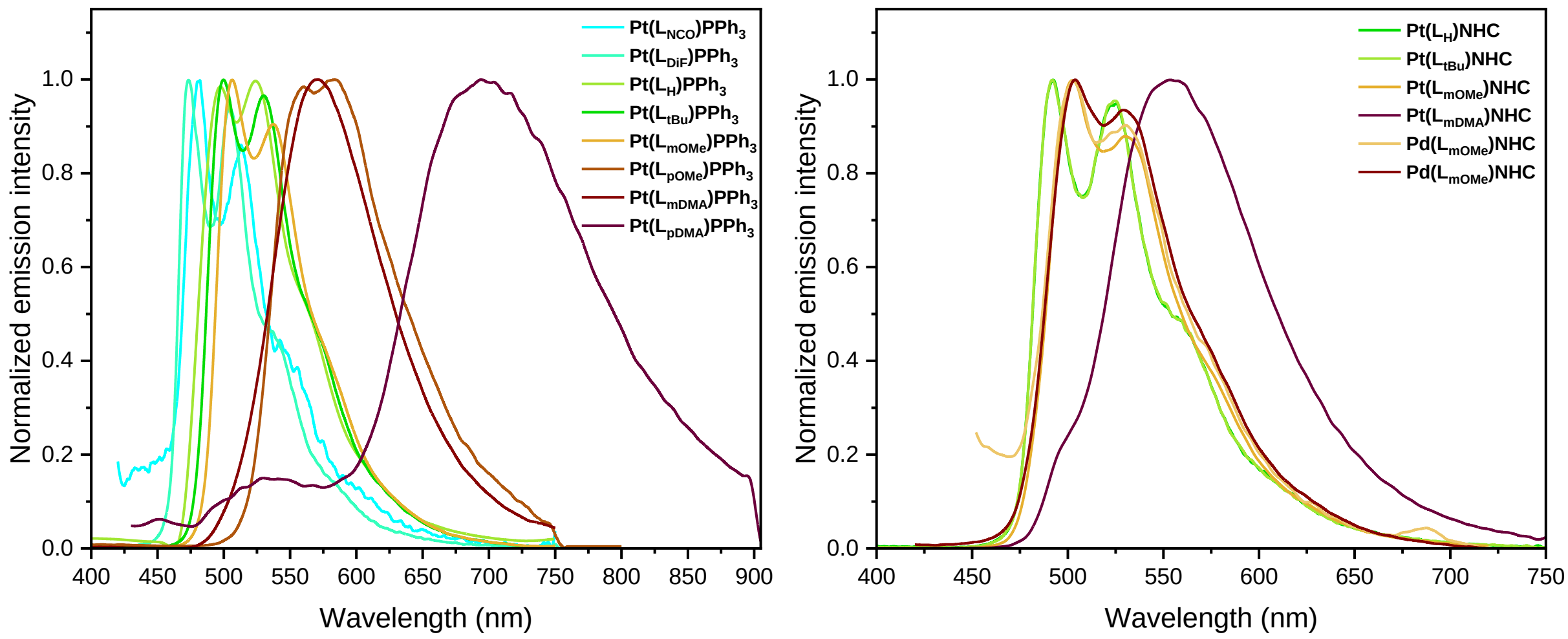

Figure S-294. Photoluminescence emission spectra (λ_{exc} = 350 nm) of **Pt(L$_{NCO}$)PPh$_3$**, **Pt(L$_{H}$)PPh$_3$**, **Pt(L$_{tBu}$)PPh$_3$**, **Pt(L$_{DiF}$)PPh$_3$**, **Pt(L$_{mOMe}$)PPh$_3$**, **Pt(L$_{pOMe}$)PPh$_3$**, **Pt(L$_{mDMA}$)PPh$_3$** and **Pt(L$_{pDMA}$)PPh$_3$** (left) and **Pt(L$_{H}$)NHC**, **Pt(L$_{tBu}$)NHC**, **Pt(L$_{mOMe}$)NHC**, **Pt(L$_{mDMA}$)NHC**, **Pd(L$_{mOMe}$)NHC** and **Pd(L$_{mDMA}$)NHC** (right) at 298 K in liquid DCM (c = 10^{-5} M). Both spectra sets normalized to the highest intensity.

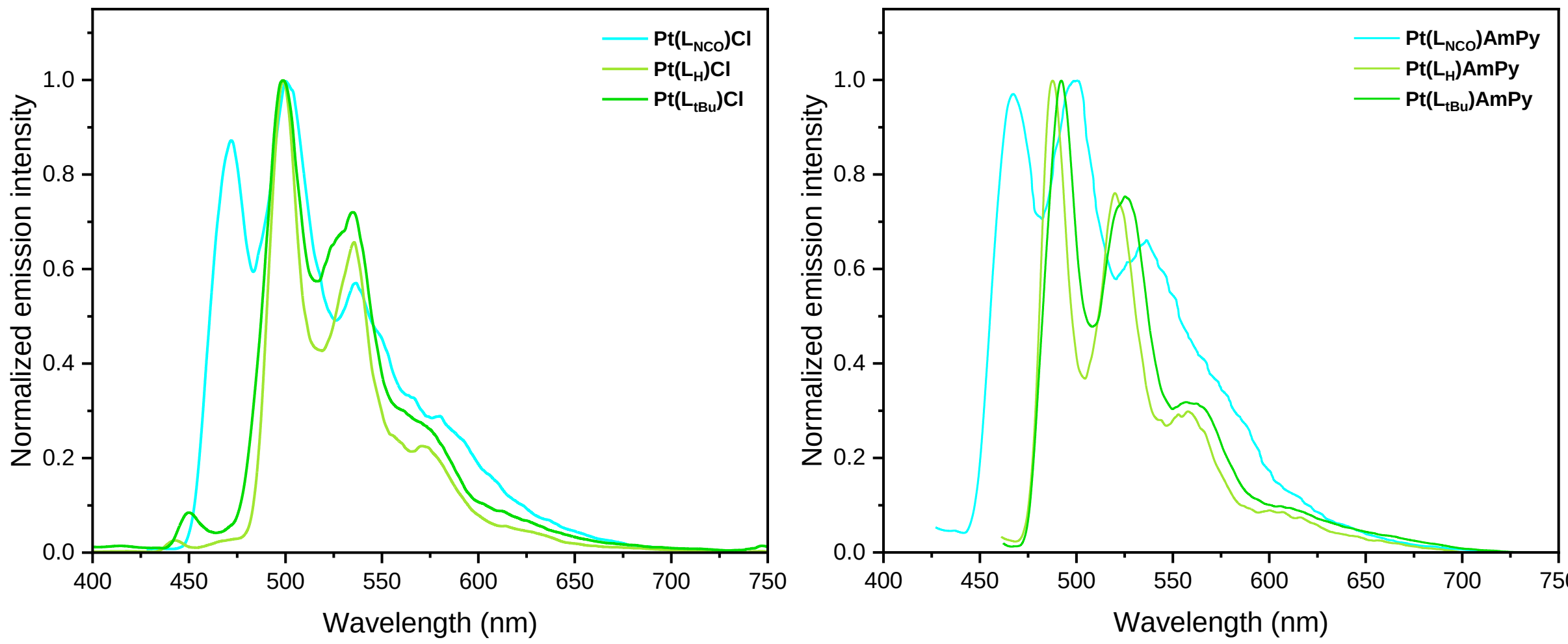

Figure S-295. Photoluminescence emission spectra (λ_{exc} = 350 nm) of **Pt(L$_{NCO}$)Cl**, **Pt(L$_{H}$)Cl** and **Pt(L$_{tBu}$)Cl** (left) and **Pt(L$_{NCO}$)AmPy**, **Pt(L$_{H}$)AmPy** and **Pt(L$_{tBu}$)AmPy** (right) at 77 K a frozen glassy matrix of DCM/MeOH (v/v = 1:1). Both spectra sets normalized to the highest intensity.

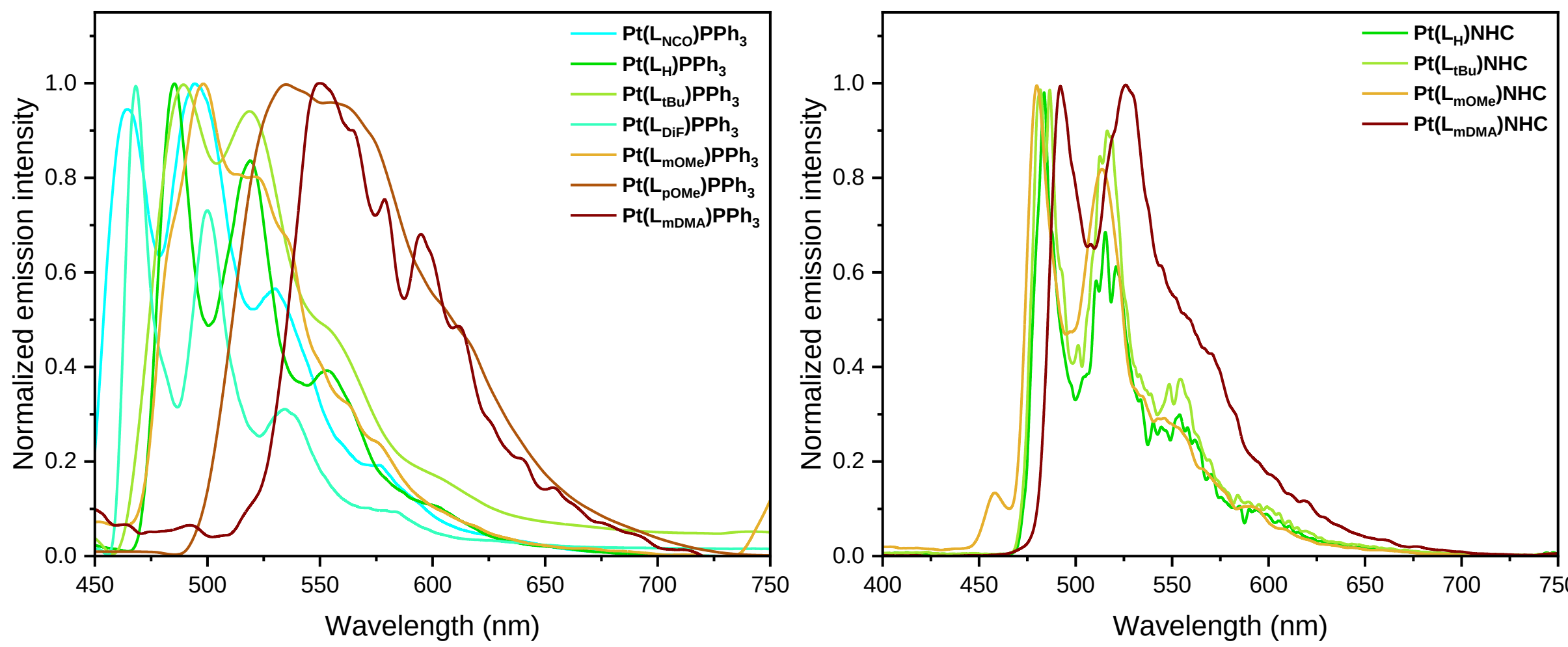

Figure S-296. Photoluminescence emission spectra (λ_{exc} = 350 nm) of **Pt(L$_{NCO}$)PPh$_3$**, **Pt(L$_{H}$)PPh$_3$**, **Pt(L$_{tBu}$)PPh$_3$**, **Pt(L$_{DiF}$)PPh$_3$**, **Pt(L$_{mOMe}$)PPh$_3$**, **Pt(L$_{pOMe}$)PPh$_3$** and **Pt(L$_{mDMA}$)PPh$_3$** (left) and **Pt(L$_{H}$)NHC**, **Pt(L$_{tBu}$)NHC**, **Pt(L$_{mOMe}$)NHC** and **Pt(L$_{mDMA}$)NHC** (right) at 77 K in a frozen glassy matrix of DCM/MeOH (v/v = 1:1). Both spectra sets normalized to the highest intensity.

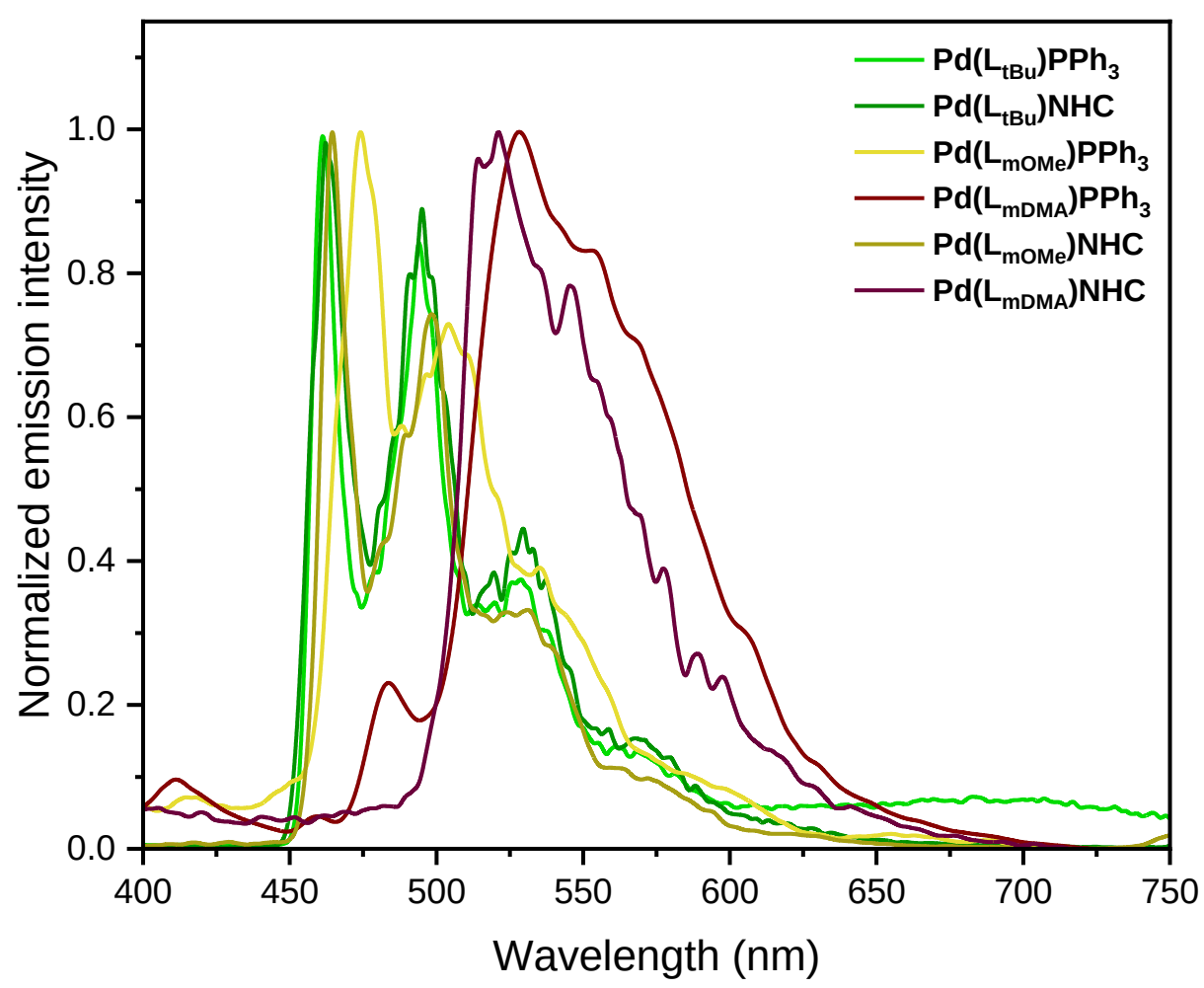

Figure S-297. Photoluminescence emission spectra (λ_{exc} = 350 nm) of **Pd(L$_{tBu}$)PPh$_3$**, **Pd(L$_{tBu}$)NHC**, **Pd(L$_{mOMe}$)PPh$_3$**, **Pd(L$_{mOMe}$)NHC**, **Pd(L$_{mDMA}$)PPh$_3$**, **Pd(L$_{mDMA}$)NHC** at 77 K in a frozen glassy matrix of DCM/MeOH (v/v = 1:1). Both spectra sets normalized to the highest intensity.

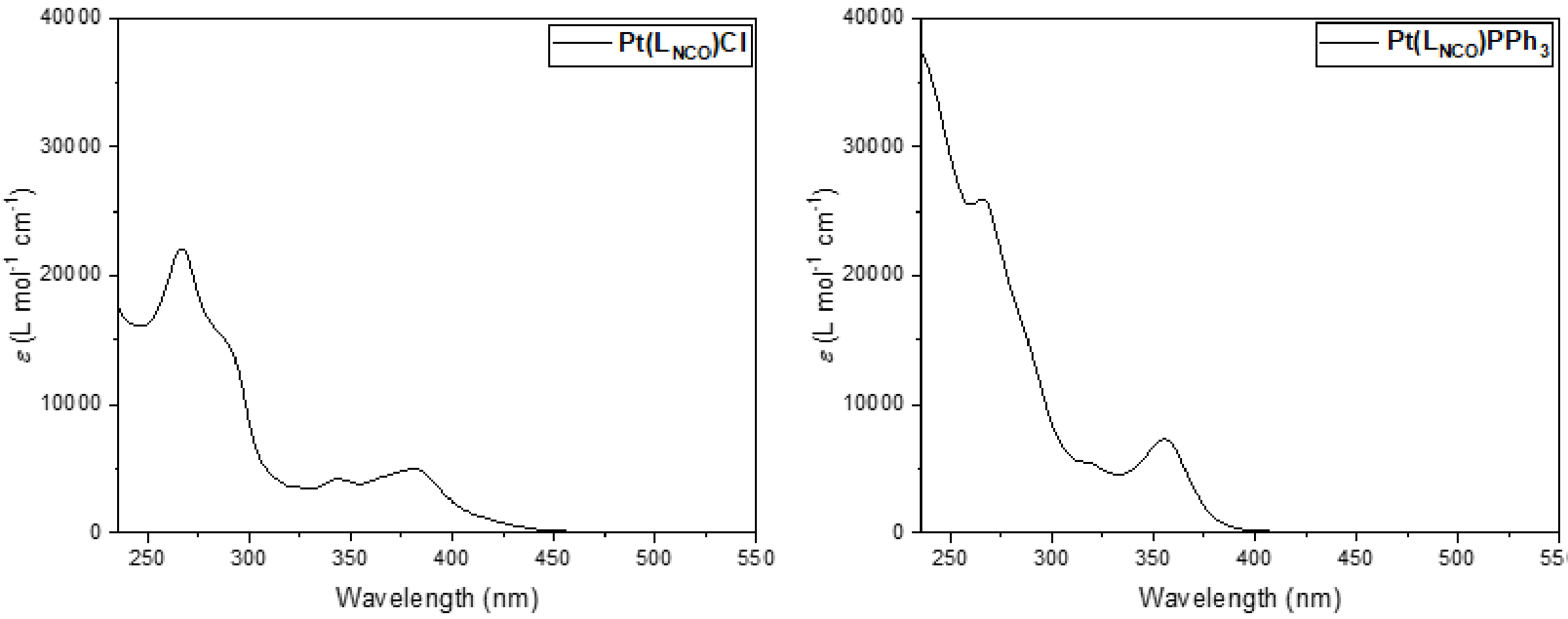

Figure S 298. UV/vis absorption spectra (molar absorption coefficients as a function of wavelength) for **Pt(L$_{NCO}$)Cl** (left) and **Pt(L$_{NCO}$)PPh$_3$** (right) in DCM at 298 K.

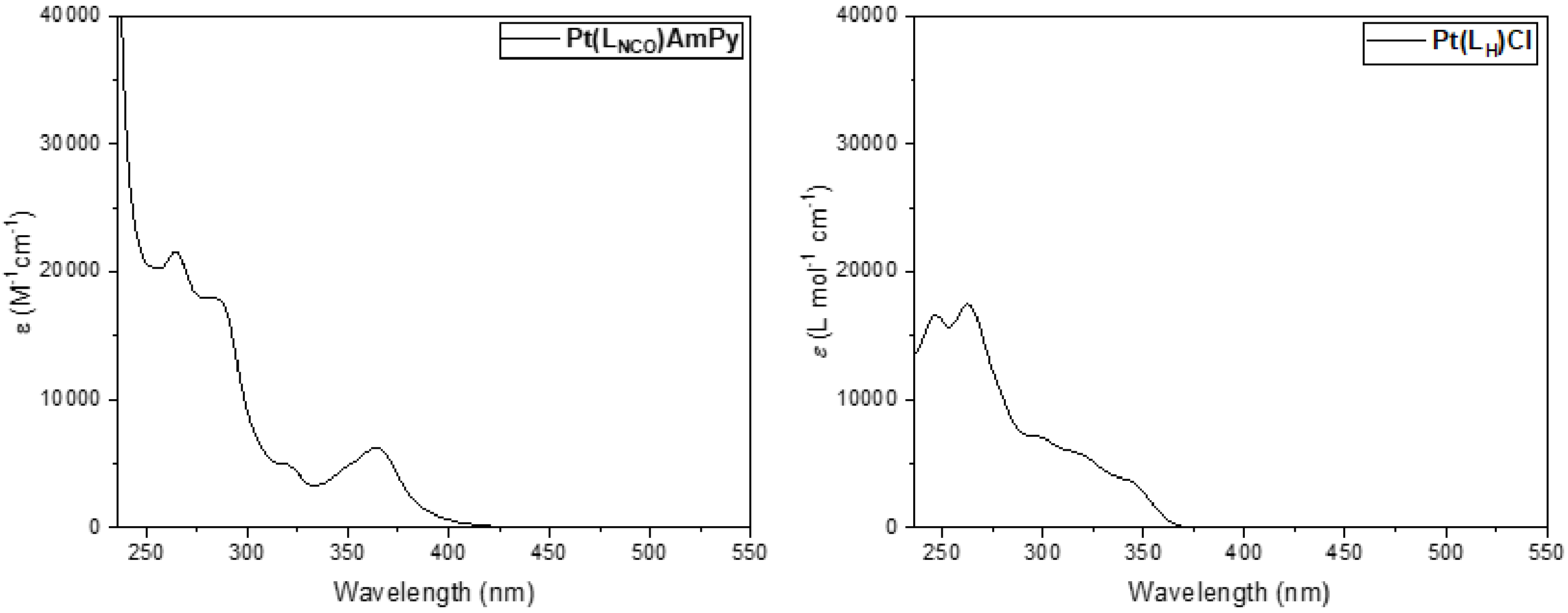

Figure S-299. UV/vis absorption spectra (molar absorption coefficients as a function of wavelength) for **Pt(L$_{NCO}$)AmPy** (left) and **Pt(L$_H$)Cl** (right) in DCM at 298 K.

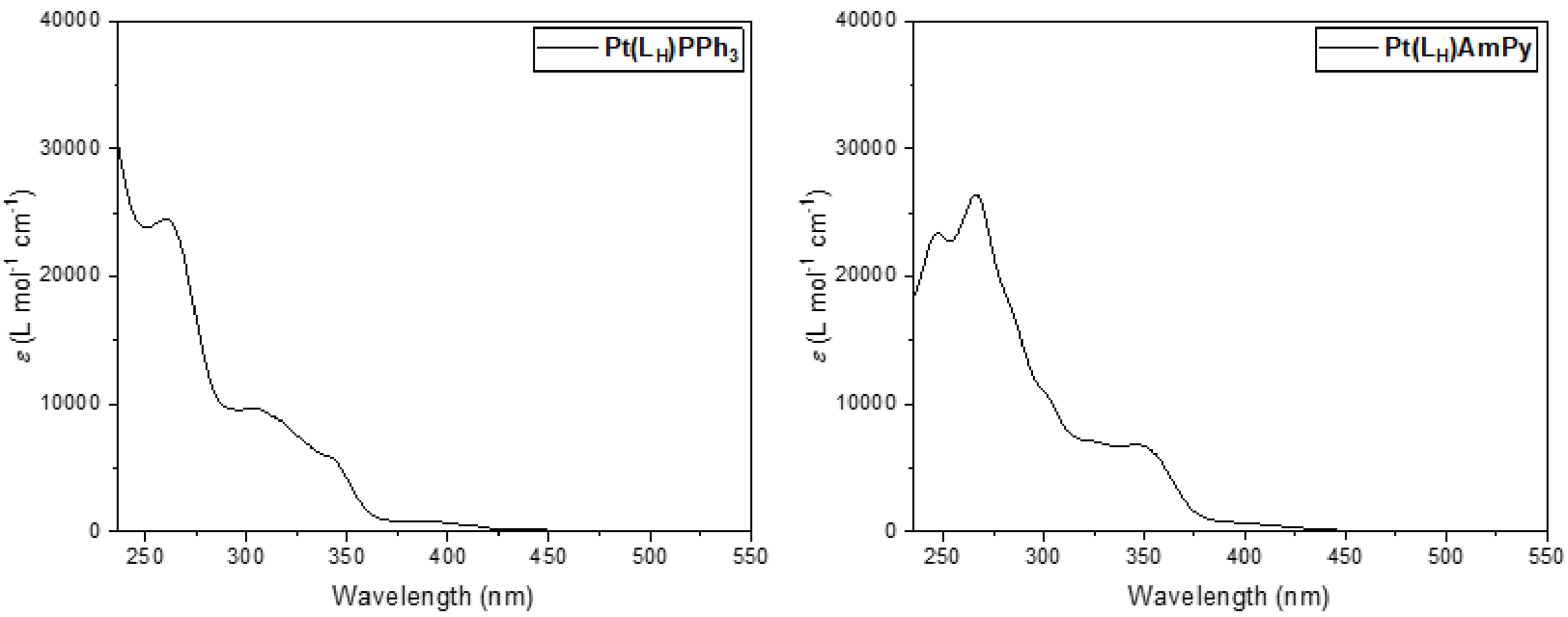

Figure S-300. UV/vis absorption spectra (molar absorption coefficients as a function of wavelength) for **Pt(L$_H$)PPh$_3$** (left) and **Pt(L$_H$)AmPy** (right) in DCM at 298 K.

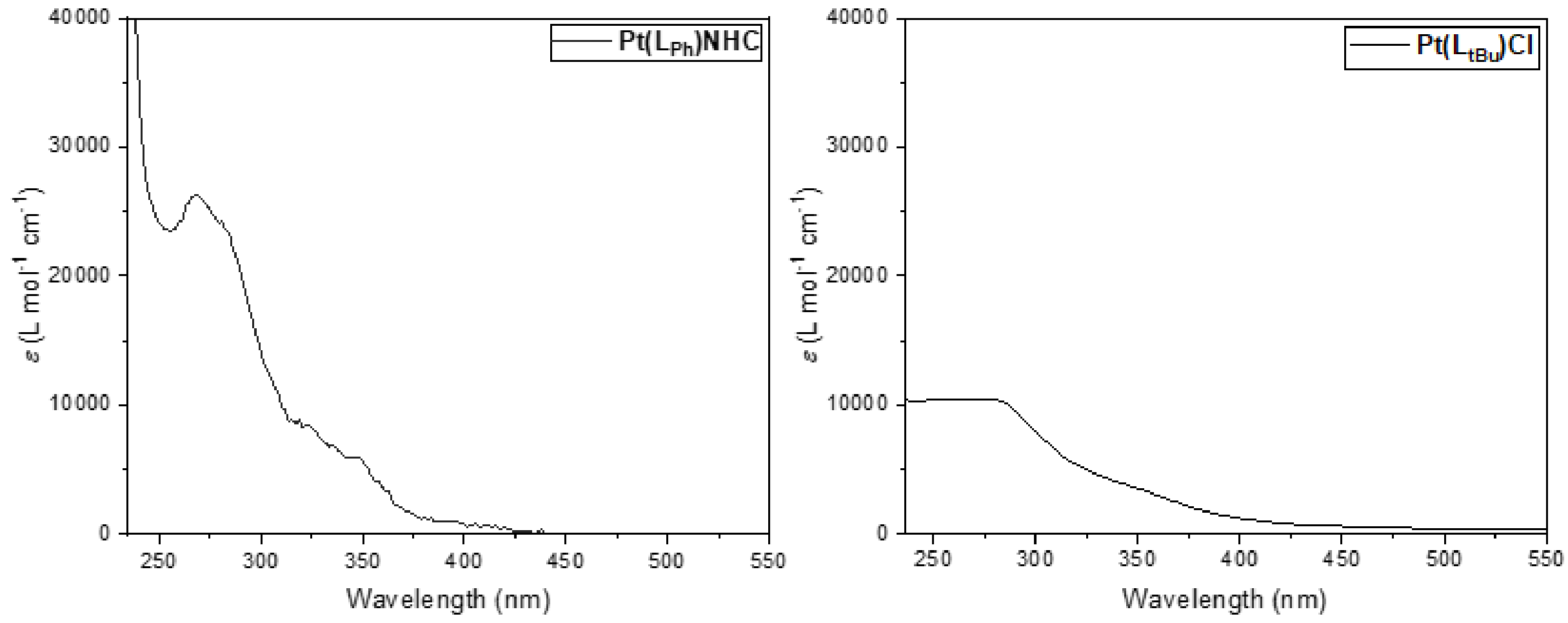

Figure S-301. UV/vis absorption spectra (molar absorption coefficients as a function of wavelength) for **Pt(L_H)NHC** (left) and **Pt(L_{tBu})Cl** (right) in DCM at 298 K.

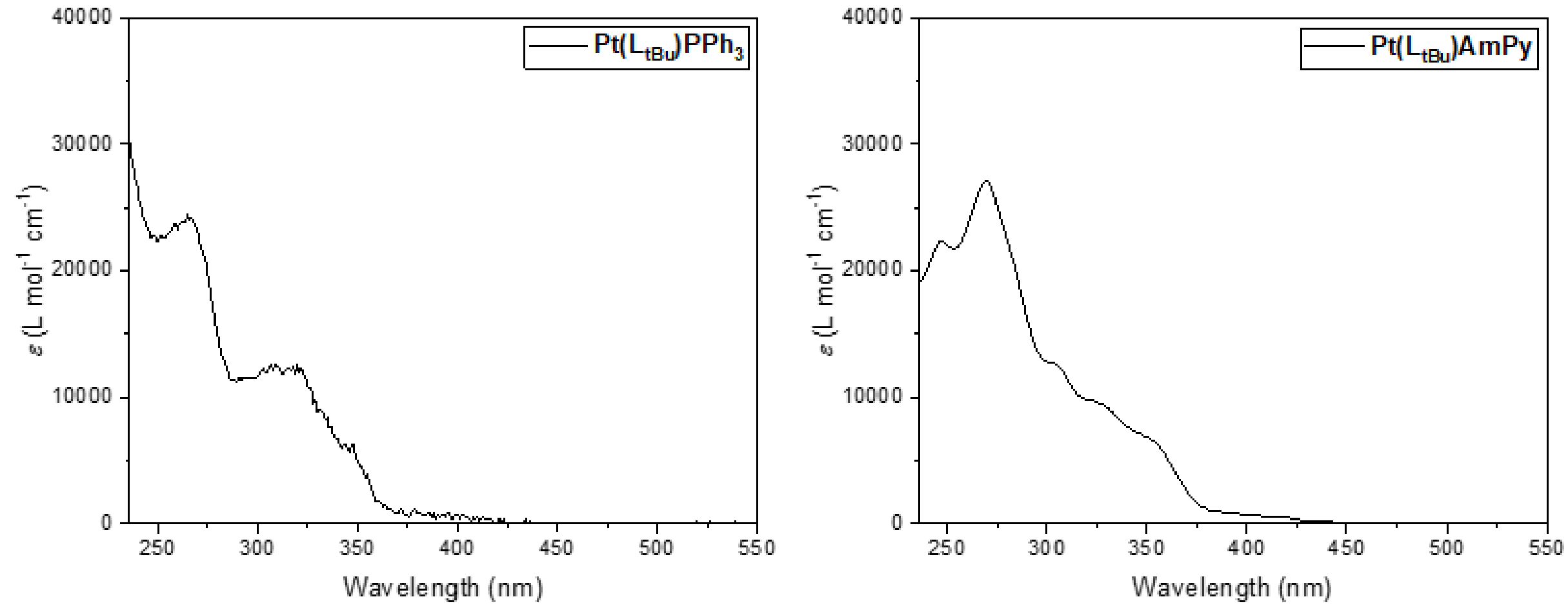

Figure S-302. UV/vis absorption spectra (molar absorption coefficients as a function of wavelength) for **Pt(L_{tBu})PPh_3** (left) and **Pt(L_{tBu})AmPy** (right) in DCM at 298 K.

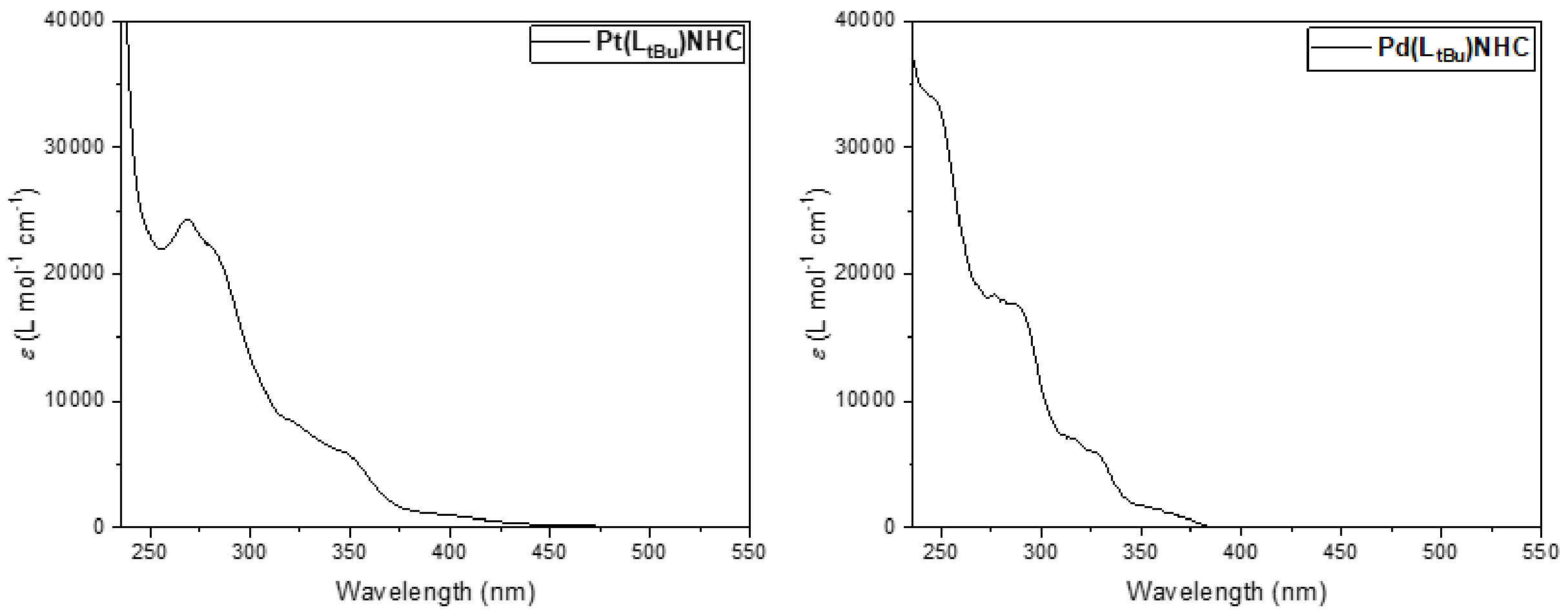

Figure S-303. UV/vis absorption spectra (molar absorption coefficients as a function of wavelength) for **Pt(L_{tBu})NHC** (left) and **Pd(L_{tBu})PPh_3** (right) in DCM at 298 K.

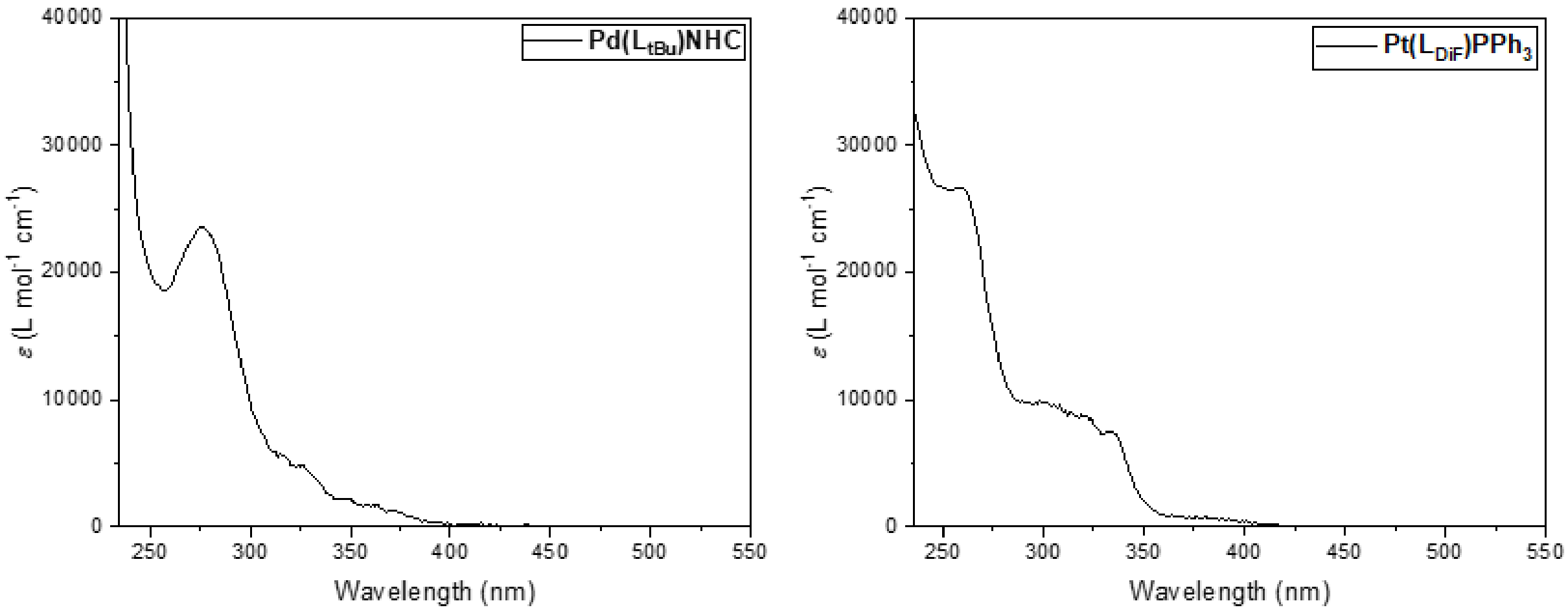

Figure S-304. UV/vis absorption spectra (molar absorption coefficients as a function of wavelength) for **Pd(L_{tBu})NHC** (left) and **Pd(L_{DiF})PPh_3** (right) in DCM at 298 K.

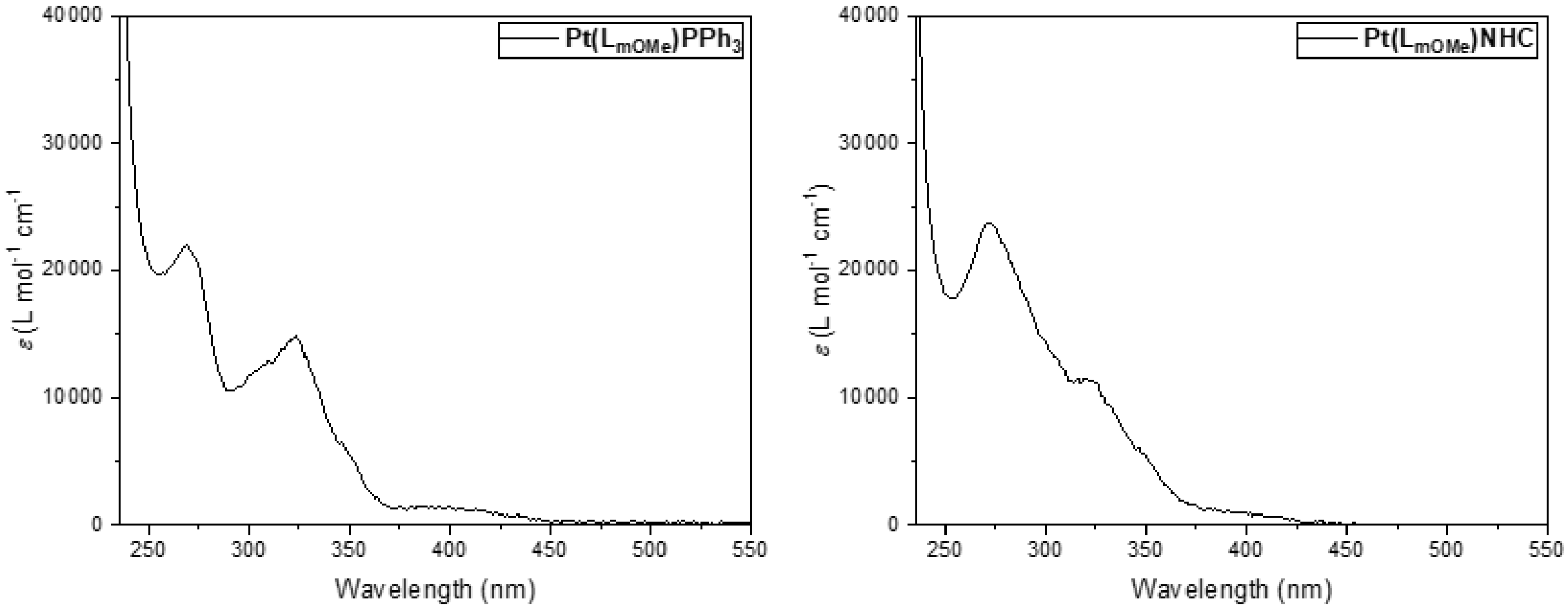

Figure S-305. UV/vis absorption spectra (molar absorption coefficients as a function of wavelength) for **Pt(L_{mOMe})PPh_3** (left) and **Pt(L_{mOMe})NHC** (right) in DCM at 298 K.

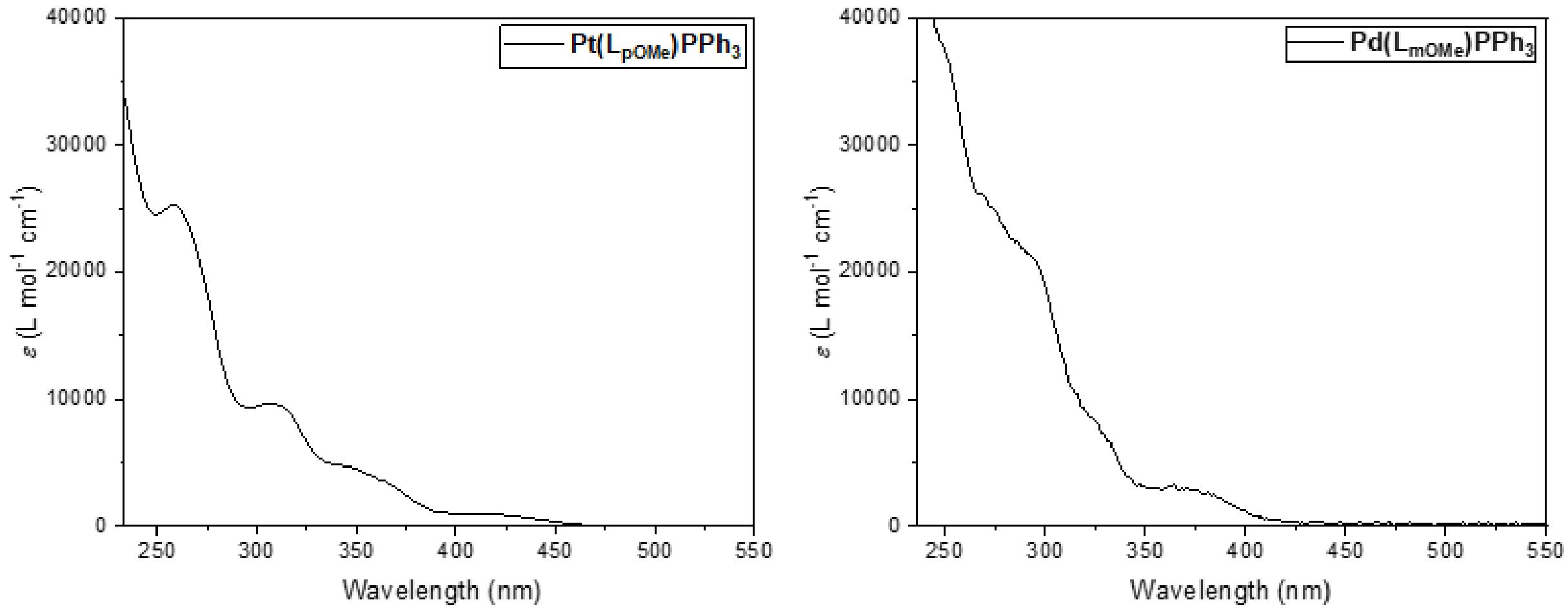

Figure S-306. UV/vis absorption spectra (molar absorption coefficients as a function of wavelength) for **Pt(L_{pOMe})PPh_3** (left) and **Pd(L_{mOMe})PPh_3** (right) in DCM at 298 K.

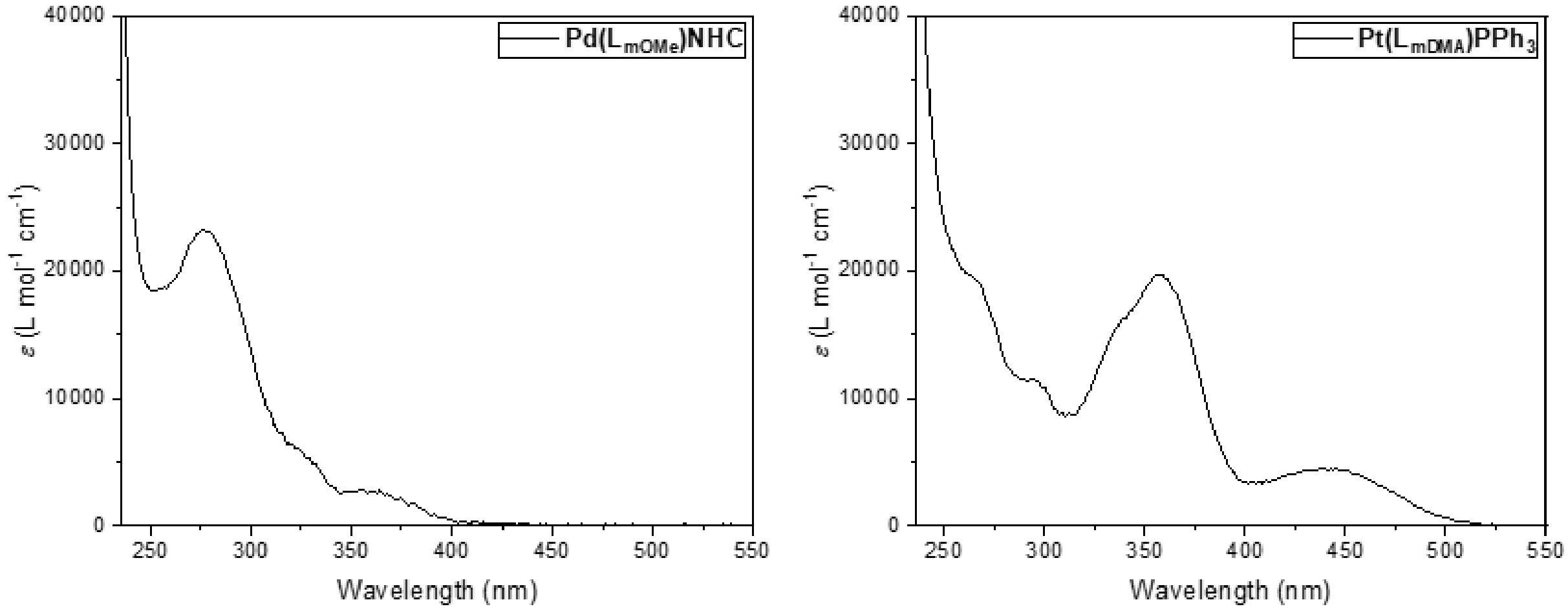

Figure S-307. UV/vis absorption spectra (molar absorption coefficients as a function of wavelength) for **Pd(L$_{mOMe}$)NHC** (left) and **Pt(L$_{mDMA}$)PPh$_3$** (right) in DCM at 298 K.

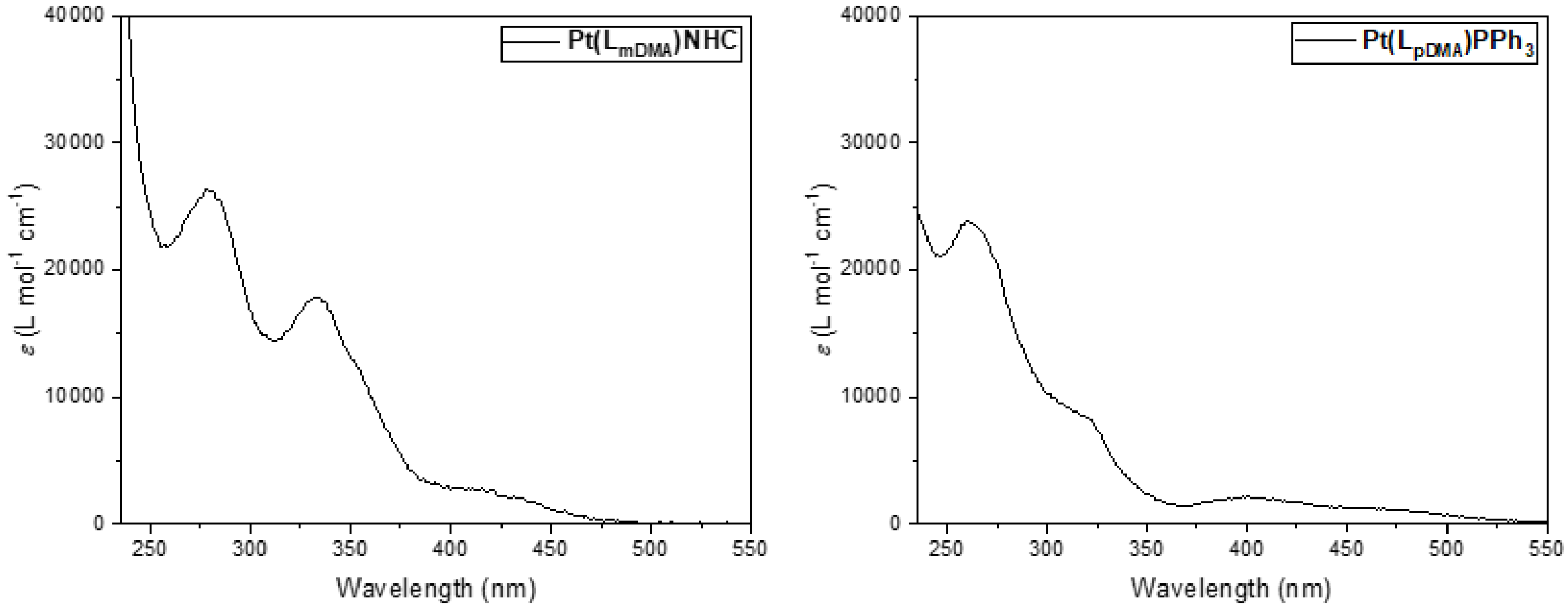

Figure S-308. UV/vis absorption spectra (molar absorption coefficients as a function of wavelength) for **Pt(L$_{mDMA}$)NHC** (left) and **Pt(L$_{pDMA}$)PPh$_3$** (right) in DCM at 298 K.

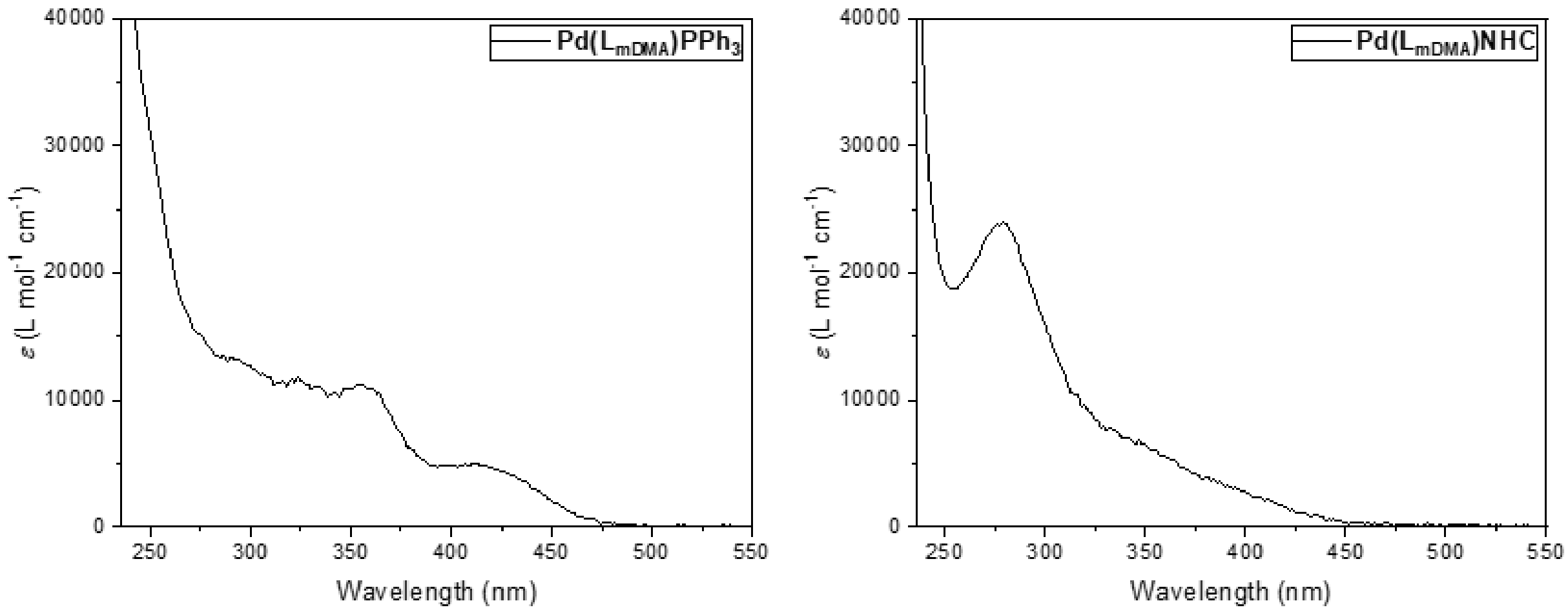

Figure S-309. UV/vis absorption spectra (molar absorption coefficients as a function of wavelength) for **Pd(L$_{mDMA}$)PPh$_3$** (left) and **Pd(L$_{mDMA}$)NHC** (right) in DCM at 298 K.

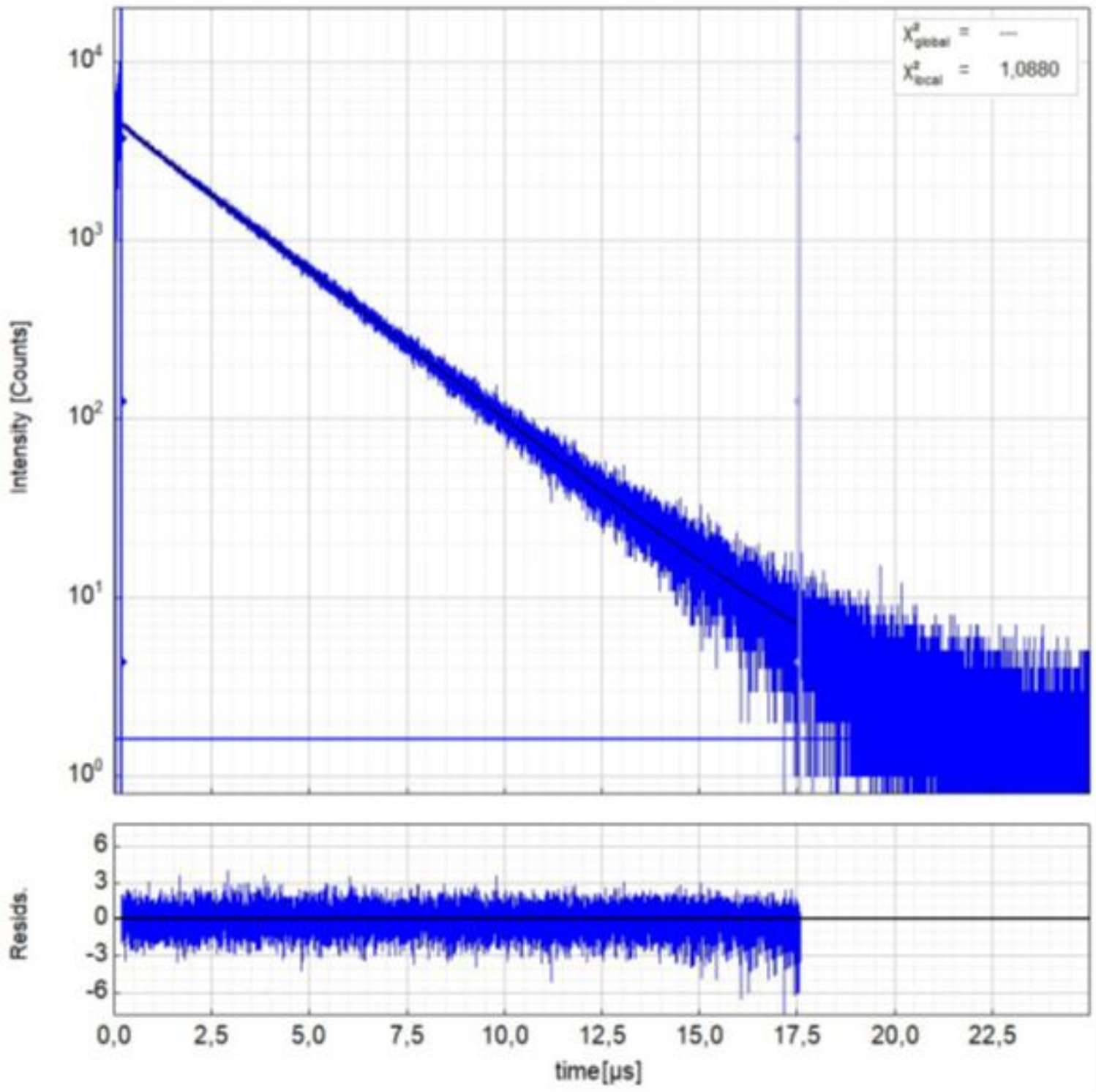

Parameter	Value	Δ	δ
A_1[kCnts/Chnl]	4,32	±0,13	3,0%
τ_1[ns]	2 598	±27	1,0%
I_1[kCnts]	11 220	±230	2,0%
A_{Rel1}[%]	96,6	±2,5	2,5%
I_{Rel1}[%]	99,1	±2,1	2,1%
A_2[kCnts/Chnl]	0,16	±0,11	69%
τ_2[ns]	710	±630	89%
I_2[kCnts]	110	±240	220%
A_{Rel2}[%]	3,5	±2,5	70%
I_{Rel2}[%]	1,0	±2,1	220%
$Bkgr_{Dec}$[kCnts]	0,0016	±0,0004	22%
τ_{AvInt}[ns]	2 579,6	±5,0	0,2%
τ_{AvAmp}[ns]	2 533	±16	0,6%

Figure S-310. Left: Raw (experimental) time-resolved photoluminescence decay of **Pt(L$_{NCO}$)Cl** in liquid DCM at 298 K (Ar-purged), including the residuals (λ_{exc} = 376.7 nm, λ_{em} = 496 nm). Right: Fitting parameters including pre-exponential factors and confidence limits.

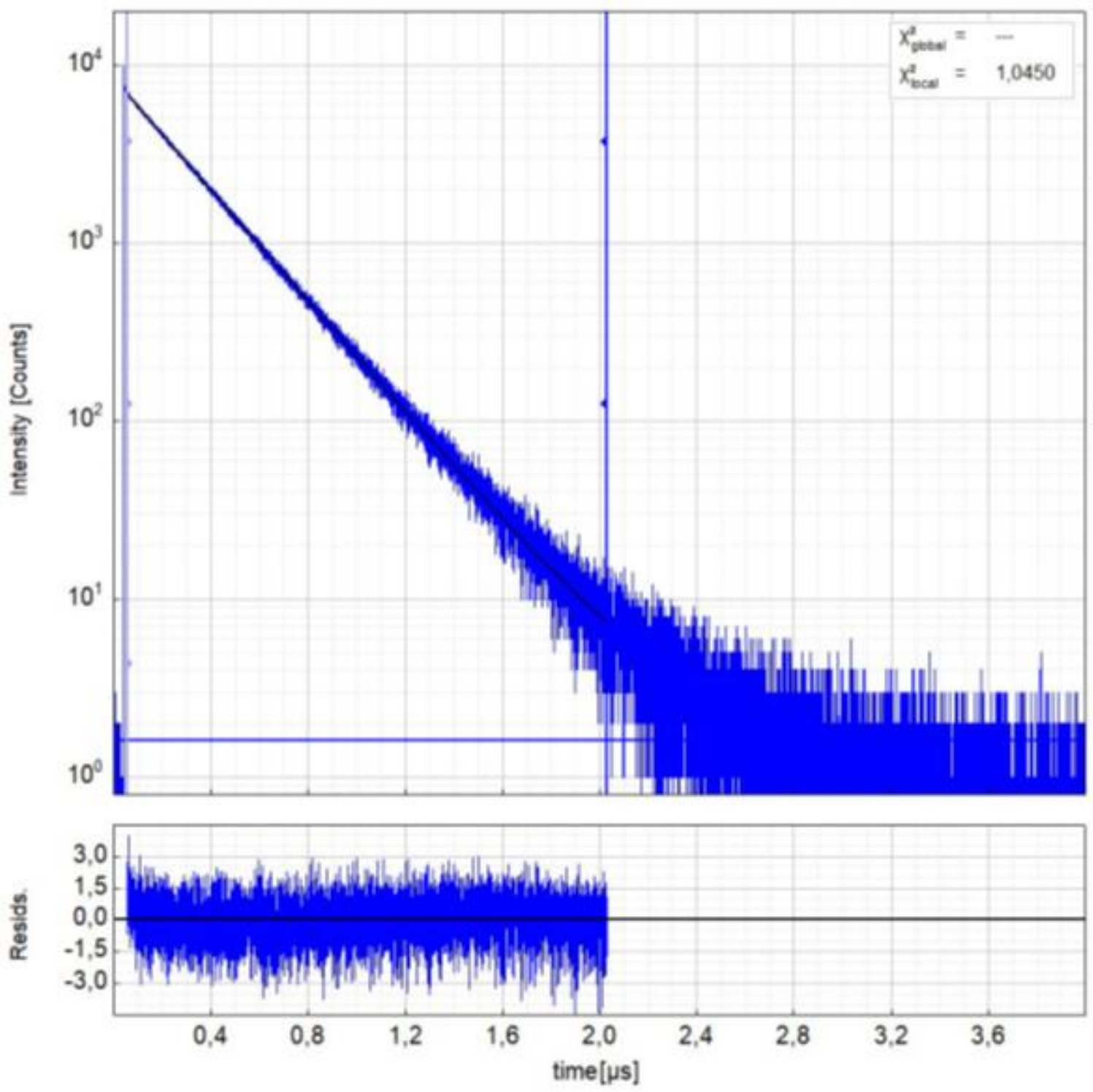

Parameter	Value	Δ	δ
A_1[kCnts/Chnl]	6,8029	±0,0091	0,1%
τ_1[ns]	278,90	±0,11	0,0%
I_1[kCnts]	7 589,3	±8,7	0,1%
A_{Rel1}[%]	100,0	---	---
I_{Rel1}[%]	100,0	---	---
$Bkgr_{Dec}$[kCnts]	0,0016	---	---
τ_{AvInt}[ns]	278,90	±0,11	0,0%
τ_{AvAmp}[ns]	278,90	±0,11	0,0%

Figure S-311. Left: Raw (experimental) time-resolved photoluminescence decay of **Pt(L$_{NCO}$)Cl** in liquid DCM at 298 K (air-equilibrated), including the residuals (λ_{exc} = 376.7 nm, λ_{em} = 490 nm). Right: Fitting parameters including pre-exponential factors and confidence limits.

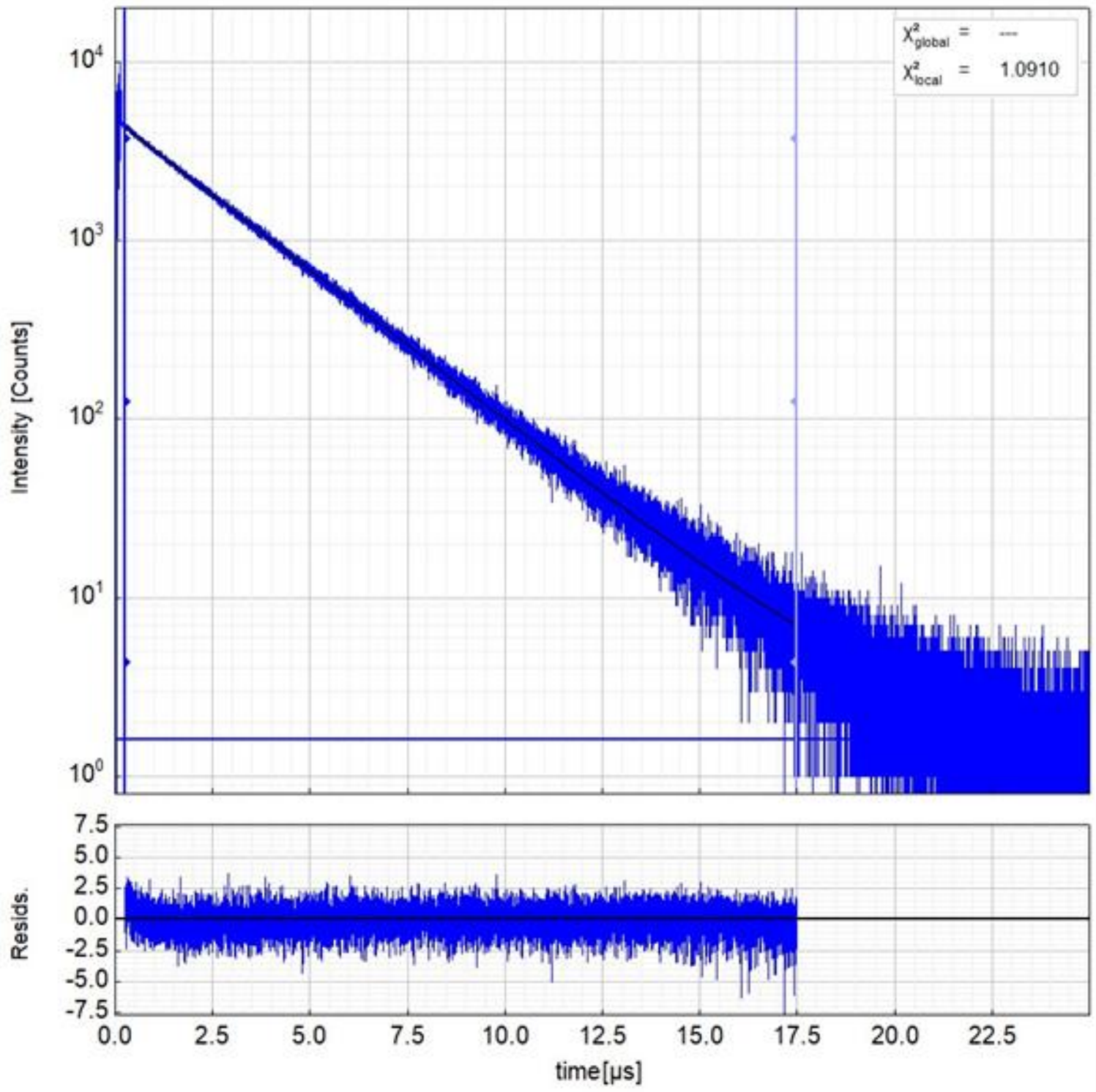

Parameter	Value	Δ	δ
A_1[kCnts/Chnl]	4.2906	±0.0026	0.1%
τ_1[ns]	2 579.69	±0.83	0.0%
I_1[kCnts]	11 068.3	±3.1	0.0%
A_{Rel1}[%]	100.0	---	---
I_{Rel1}[%]	100.0	---	---
$Bkgr_{Dec}$[kCnts]	0.0016	---	---
τ_{AvInt}[ns]	2 579.69	±0.83	0.0%
τ_{AvAmp}[ns]	2 579.69	±0.83	0.0%

Figure S-312. Left: Raw (experimental) time-resolved photoluminescence decay of Pt(L_{NCO})PPh_3 in a frozen glassy matrix of DCM/MeOH (v/v = 1:1) at 77 K, including the residuals (λ_{exc} = 376.7 nm, λ_{em} = 471 nm). Right: Fitting parameters including pre-exponential factors and confidence limits.

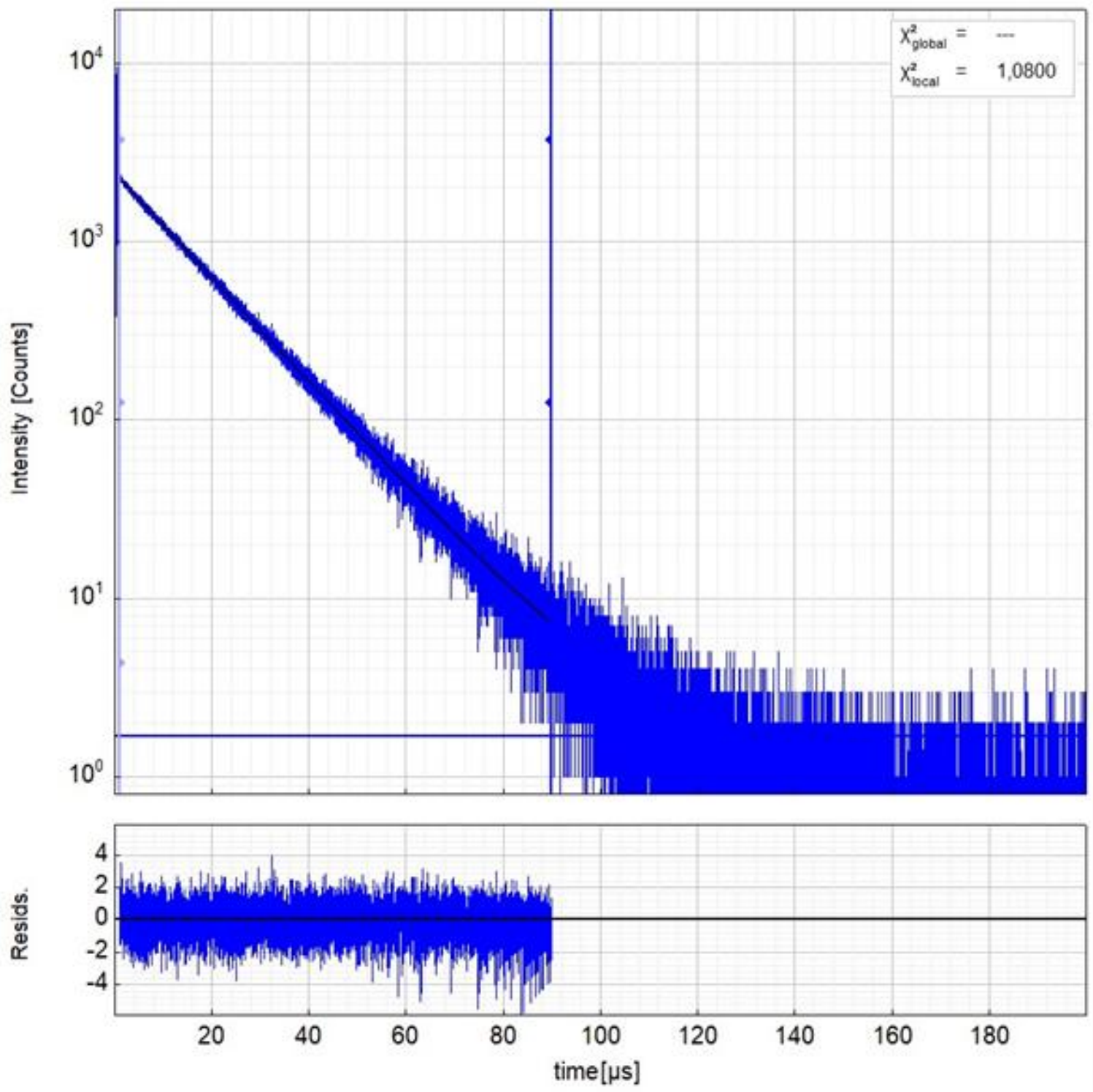

Parameter	Value	Δ	δ
A_1[kCnts/Chnl]	2,2372	±0,0044	0,2%
τ_1[ns]	14 843	±28	0,2%
I_1[kCnts]	4 150,5	±6,2	0,1%
A_{Rel1}[%]	100,0	---	---
I_{Rel1}[%]	100,0	---	---
$Bkgr_{Dec}$[kCnts]	0,0017	±0,0003	14%
τ_{AvInt}[ns]	14 843	±28	0,2%
τ_{AvAmp}[ns]	14 843	±28	0,2%

Figure S-313. Left: Raw (experimental) time-resolved photoluminescence decay of **Pt(L_{NCO})PPh_3** in a frozen glassy matrix of DCM/MeOH (v/v = 1:1) at 77 K, including the residuals (λ_{exc} = 376.7 nm, λ_{em} = 485 nm). Right: Fitting parameters including pre-exponential factors and confidence limits.

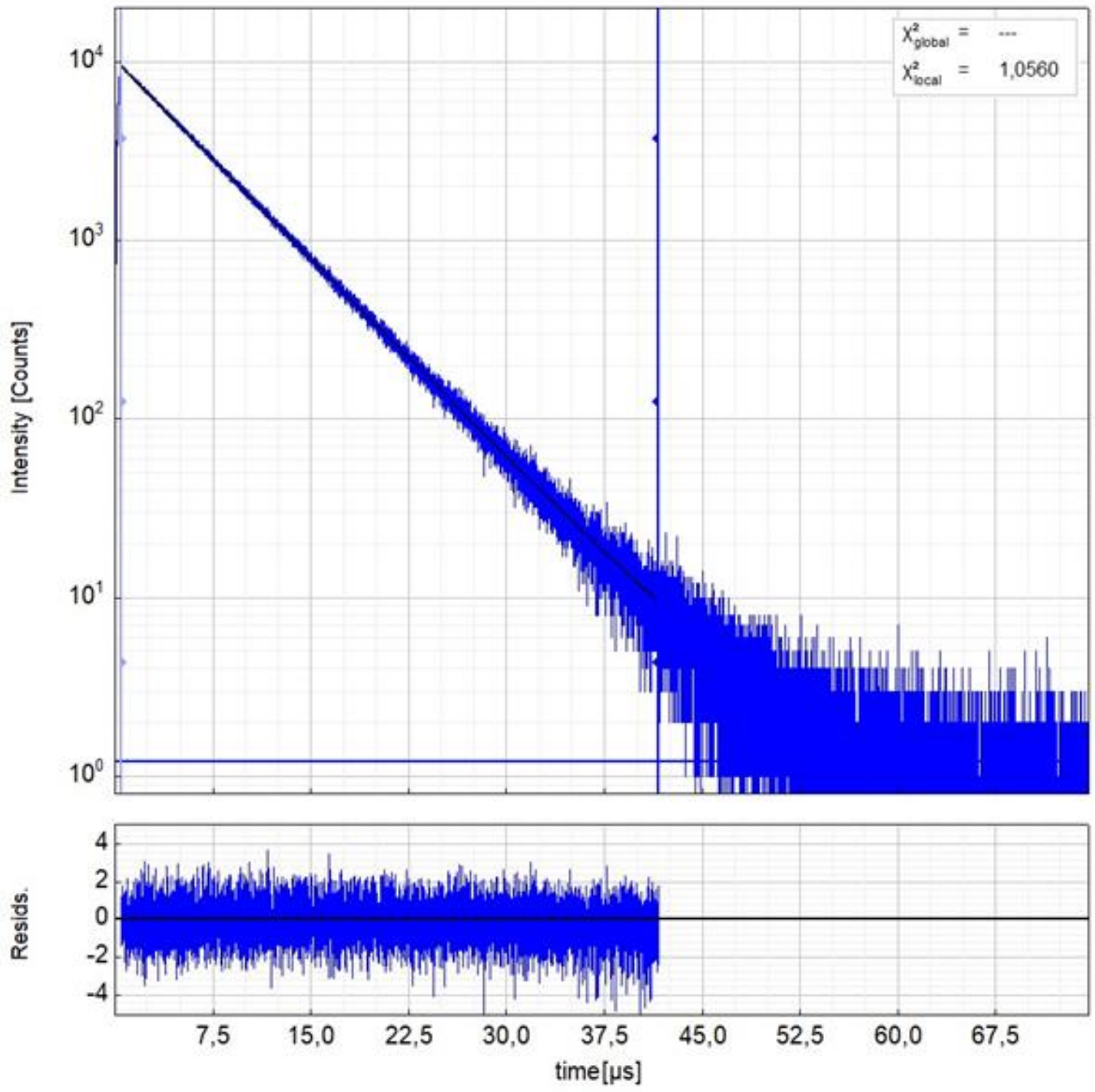

Parameter	Value	Δ	δ
A_1[kCnts/Chnl]	9,4540	±0,0023	0,0%
τ_1[ns]	5 849,33	±0,86	0,0%
I_1[kCnts]	13 824,8	±3,4	0,0%
A_{Rel1}[%]	100,0	---	---
I_{Rel1}[%]	100,0	---	---
$Bkgr_{Dec}$[kCnts]	0,0012	---	---
τ_{AvInt}[ns]	5 849,33	±0,86	0,0%
τ_{AvAmp}[ns]	5 849,33	±0,86	0,0%

Figure S-314. Left: Raw (experimental) time-resolved photoluminescence decay of **Pt(L_{NCO})AmPy** in liquid DCM at 298 K (Ar-purged), including the residuals (λ_{exc} = 376.7 nm, λ_{em} = 485 nm). Right: Fitting parameters including pre-exponential factors and confidence limits.

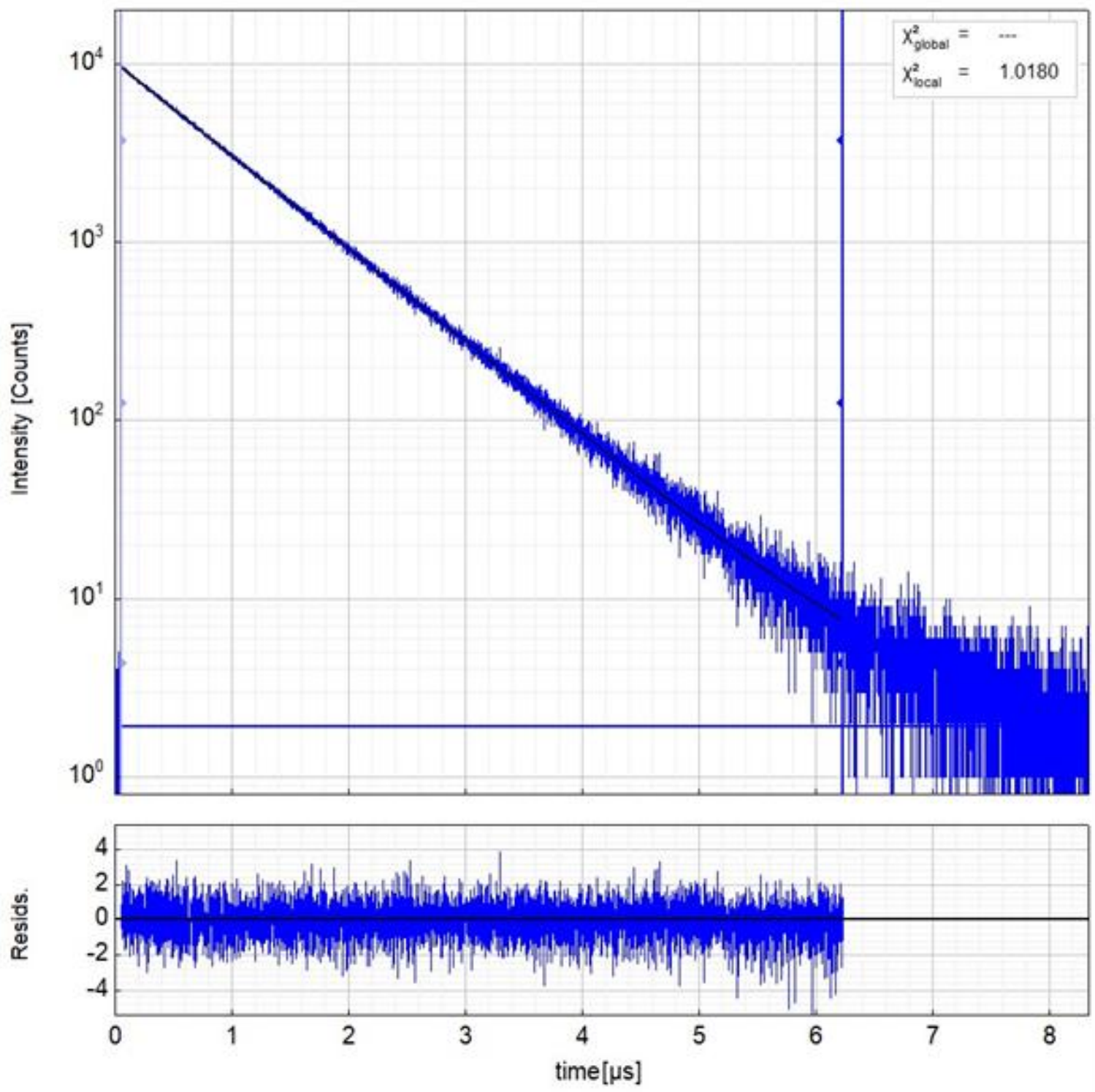

Parameter	Value	Δ	δ
A_1[kCnts/Chnl]	9.567	±0.011	0.1%
τ_1[ns]	831.30	±0.71	0.1%
I_1[kCnts]	7 952.6	±5.7	0.1%
A_{Rel1}[%]	100.0	---	---
I_{Rel1}[%]	100.0	---	---
$Bkgr_{Dec}$[kCnts]	0.0020	±0.0003	11%
τ_{AvInt}[ns]	831.30	±0.71	0.1%
τ_{AvAmp}[ns]	831.30	±0.71	0.1%

Figure S-315. Left: Raw (experimental) time-resolved photoluminescence decay of **Pt(L_{NCO})AmPy** in liquid DCM at 298 K (air-equilibrated), including the residuals (λ_{exc} = 376.7 nm, λ_{em} = 485 nm). Right: Fitting parameters including pre-exponential factors and confidence limits.

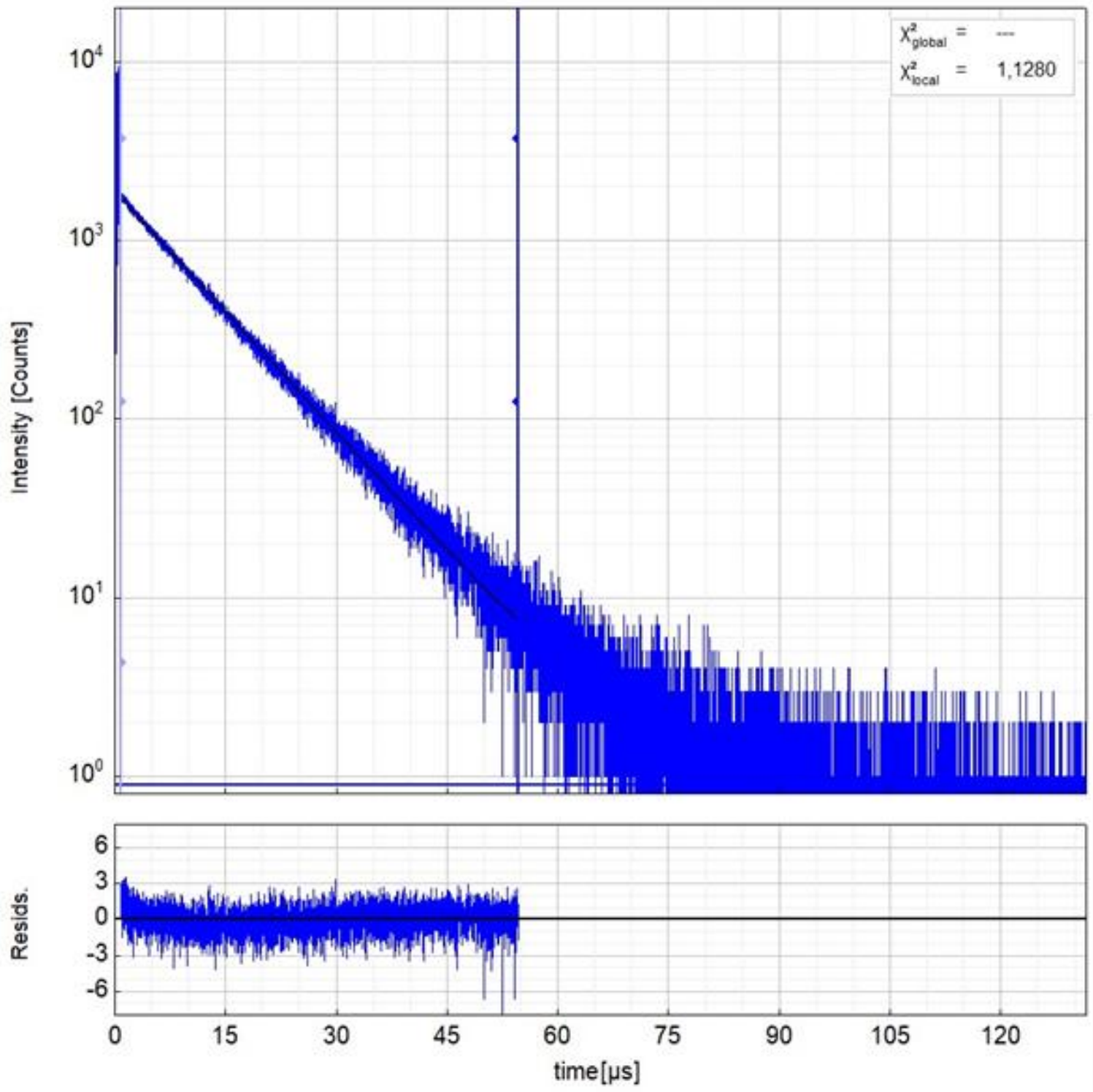

Parameter	Value	Δ	δ
A_1[kCnts/Chnl]	1,7229	±0,0032	0,2%
τ_1[ns]	9 646	±23	0,2%
I_1[kCnts]	2 077,3	±2,8	0,1%
A_{Rel1}[%]	100,0	---	---
I_{Rel1}[%]	100,0	---	---
$Bkgr_{Dec}$[kCnts]	0,0009	---	---
τ_{AvInt}[ns]	9 646	±23	0,2%
τ_{AvAmp}[ns]	9 646	±23	0,2%

Figure S-316. Left: Raw (experimental) time-resolved photoluminescence decay of **Pt(L_{NCO})AmPy** in a frozen glassy matrix of DCM/MeOH (v/v = 1:1) at 77 K, including the residuals (λ_{exc} = 376.7 nm, λ_{em} = 470 nm). Right: Fitting parameters including pre-exponential factors and confidence limits.

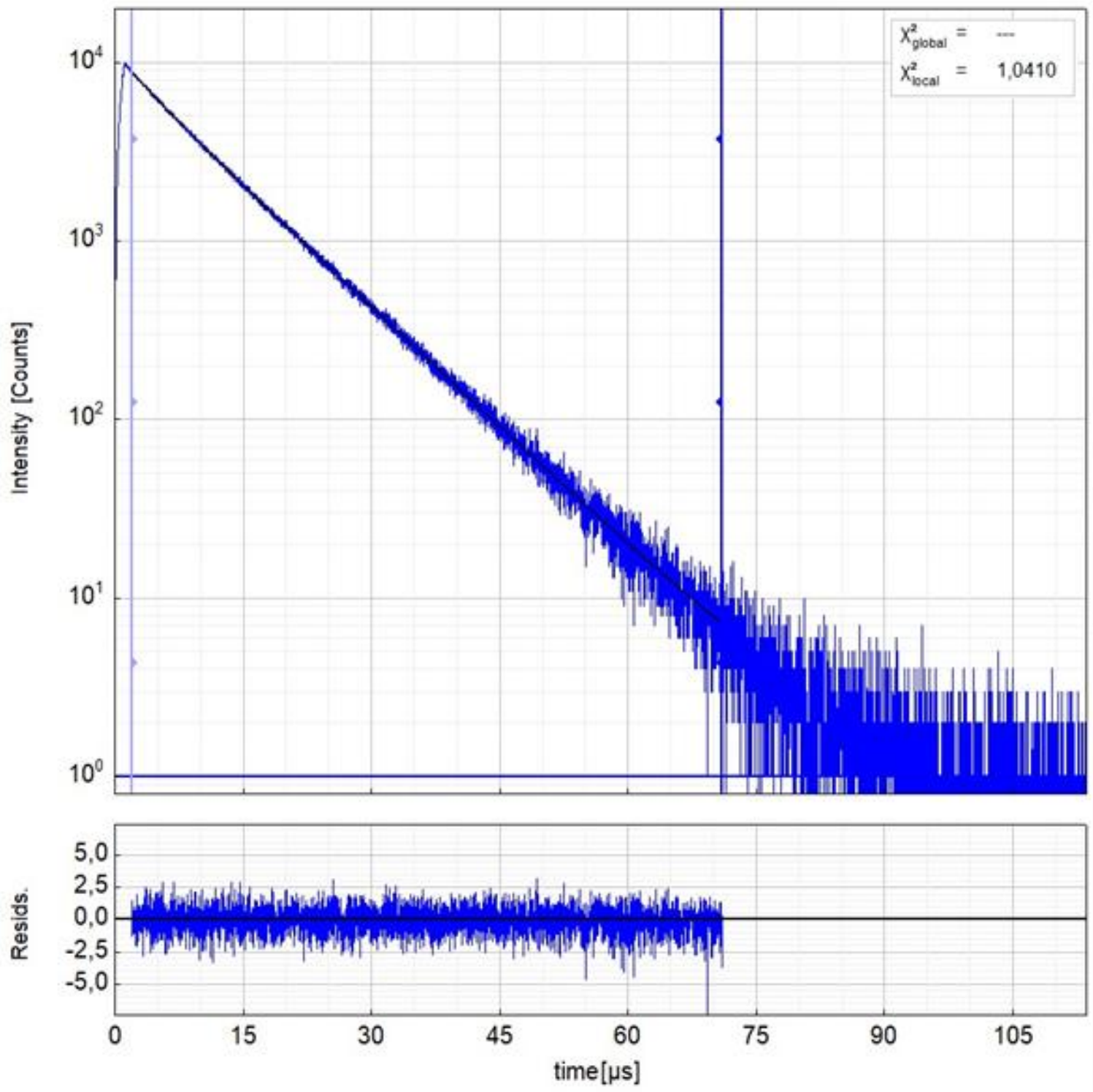

Parameter	Value	Δ	δ
A_1[kCnts/Chnl]	7,732	±0,039	0,5%
τ_1[ns]	9 681,7	±10,0	0,1%
I_1[kCnts]	4 679	±17	0,4%
A_{Rel1}[%]	87,3	±0,4	0,5%
I_{Rel1}[%]	94,5	±0,4	0,4%
A_2[kCnts/Chnl]	1,129	±0,035	3,0%
τ_2[ns]	3 930	±120	3,0%
I_2[kCnts]	277	±19	6,5%
A_{Rel2}[%]	12,8	±0,4	3,1%
I_{Rel2}[%]	5,6	±0,4	6,5%
$Bkgr_{Dec}$[kCnts]	0,0010	---	---
τ_{AvInt}[ns]	9 360,3	±5,0	0,1%
τ_{AvAmp}[ns]	8 948,3	±9,4	0,1%

Figure S-317. Left: Raw (experimental) time-resolved photoluminescence decay of **Pt(L_H)Cl** in liquid DCM at 298 K (Ar-purged), including the residuals (λ_{exc} = 376.7 nm, λ_{em} = 494 nm). Right: Fitting parameters including pre-exponential factors and confidence limits.

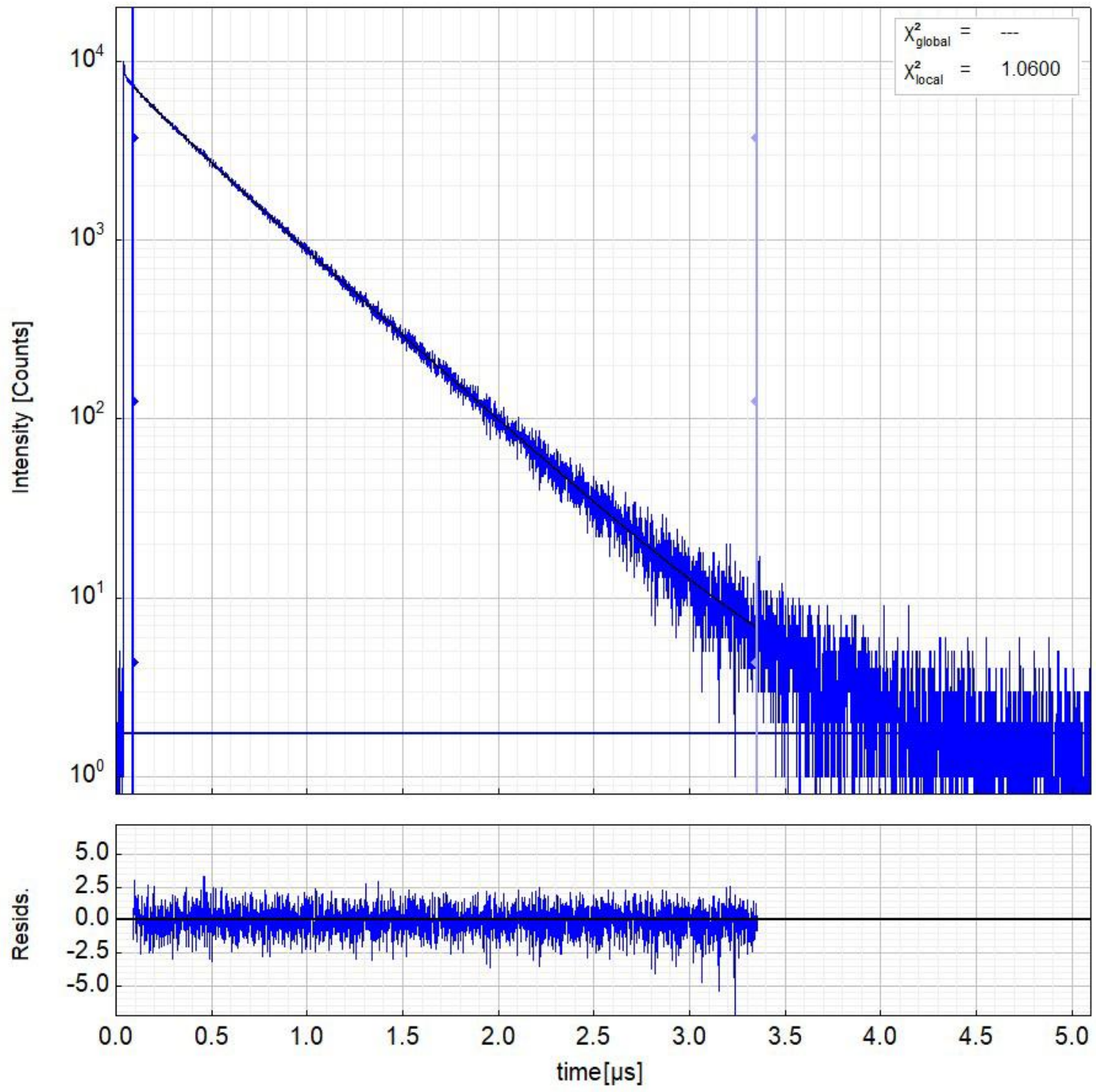

Parameter	Value	Δ	δ
A_1[kCnts/Chnl]	1,48	±0,17	11%
τ_1[ns]	144,6	±4,7	3,2%
I_1[kCnts]	416	±31	7,3%
A_{Rel1}[%]	27,9	±3,0	11%
I_{Rel1}[%]	45,4	±3,4	7,4%
A_2[kCnts/Chnl]	3,83	±0,15	3,9%
τ_2[ns]	67,1	±1,7	2,4%
I_2[kCnts]	501	±32	6,2%
A_{Rel2}[%]	72,2	±3,0	4,1%
I_{Rel2}[%]	54,7	±3,4	6,2%
$Bkgr_{Dec}$[kCnts]	0,0060	±0,0006	9,2%
τ_{AvInt}[ns]	102,23	±0,52	0,5%
τ_{AvAmp}[ns]	88,62	±0,37	0,4%

Figure S-318. Left: Raw (experimental) time-resolved photoluminescence decay of **Pt(L$_H$)Cl** in liquid DCM at 298 K (air-equilibrated), including the residuals (λ_{exc} = 376.7 nm, λ_{em} = 494 nm). Right: Fitting parameters including pre-exponential factors and confidence limits.

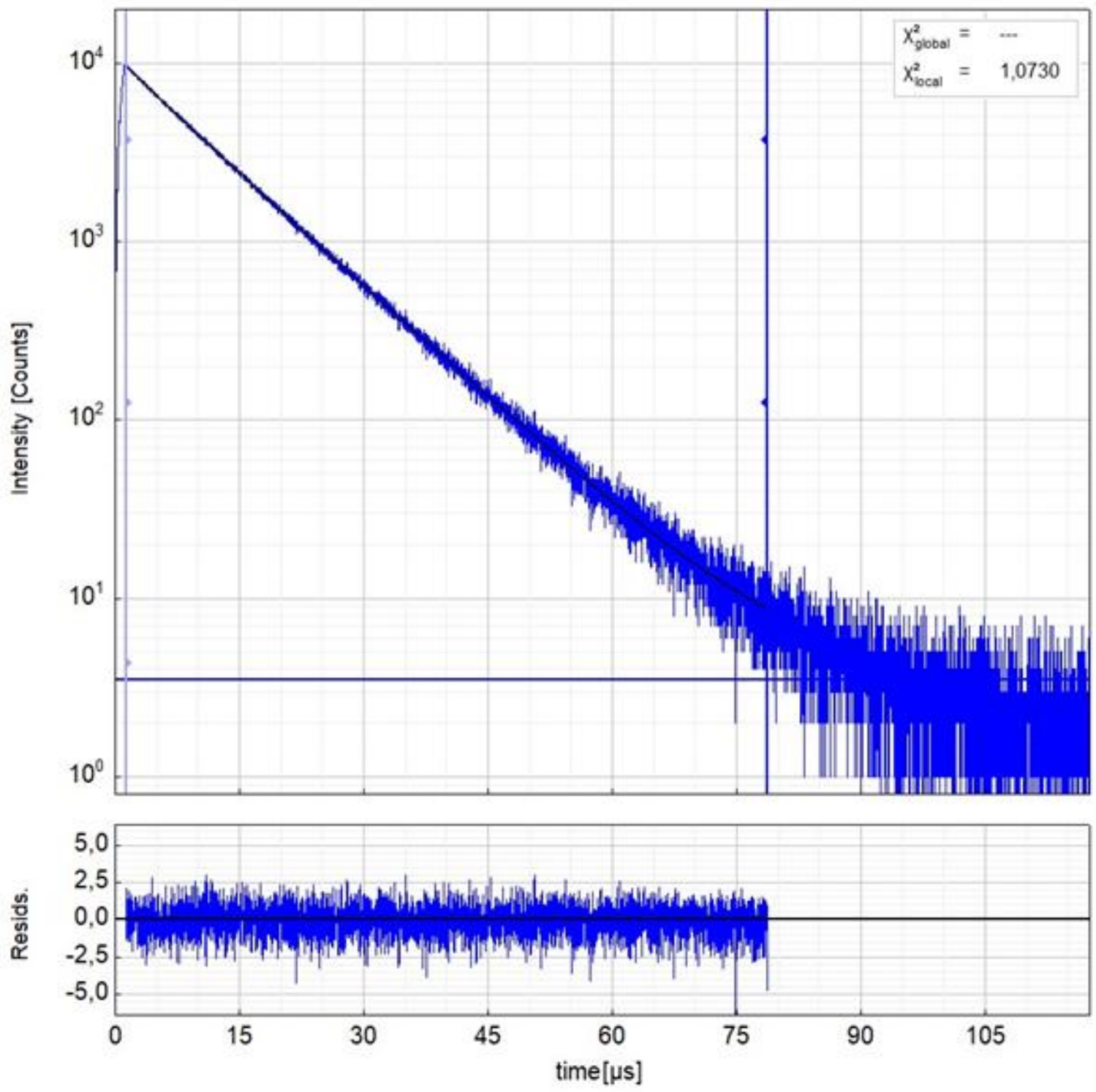

Parameter	Value	Δ	δ
A_1[kCnts/Chnl]	8,81	±0,12	1,3%
τ_1[ns]	10 422	±36	0,3%
I_1[kCnts]	5 739	±56	1,0%
A_{Rel1}[%]	90,9	±1,2	1,2%
I_{Rel1}[%]	95,5	±1,0	1,0%
A_2[kCnts/Chnl]	0,89	±0,11	12%
τ_2[ns]	4 940	±460	9,1%
I_2[kCnts]	273	±56	20%
A_{Rel2}[%]	9,2	±1,2	12%
I_{Rel2}[%]	4,6	±1,0	20%
$Bkgr_{Dec}$[kCnts]	0,0035	---	---
τ_{AvInt}[ns]	10 172,9	±9,1	0,1%
τ_{AvAmp}[ns]	9 922	±22	0,2%

Figure S-319. Left: Raw (experimental) time-resolved photoluminescence decay of **Pt(L$_H$)Cl** in a frozen glassy matrix of DCM/MeOH (v/v = 1:1) at 77 K, including the residuals (λ_{exc} = 376.7 nm, λ_{em} = 502 nm). Right: Fitting parameters including pre-exponential factors and confidence limits.

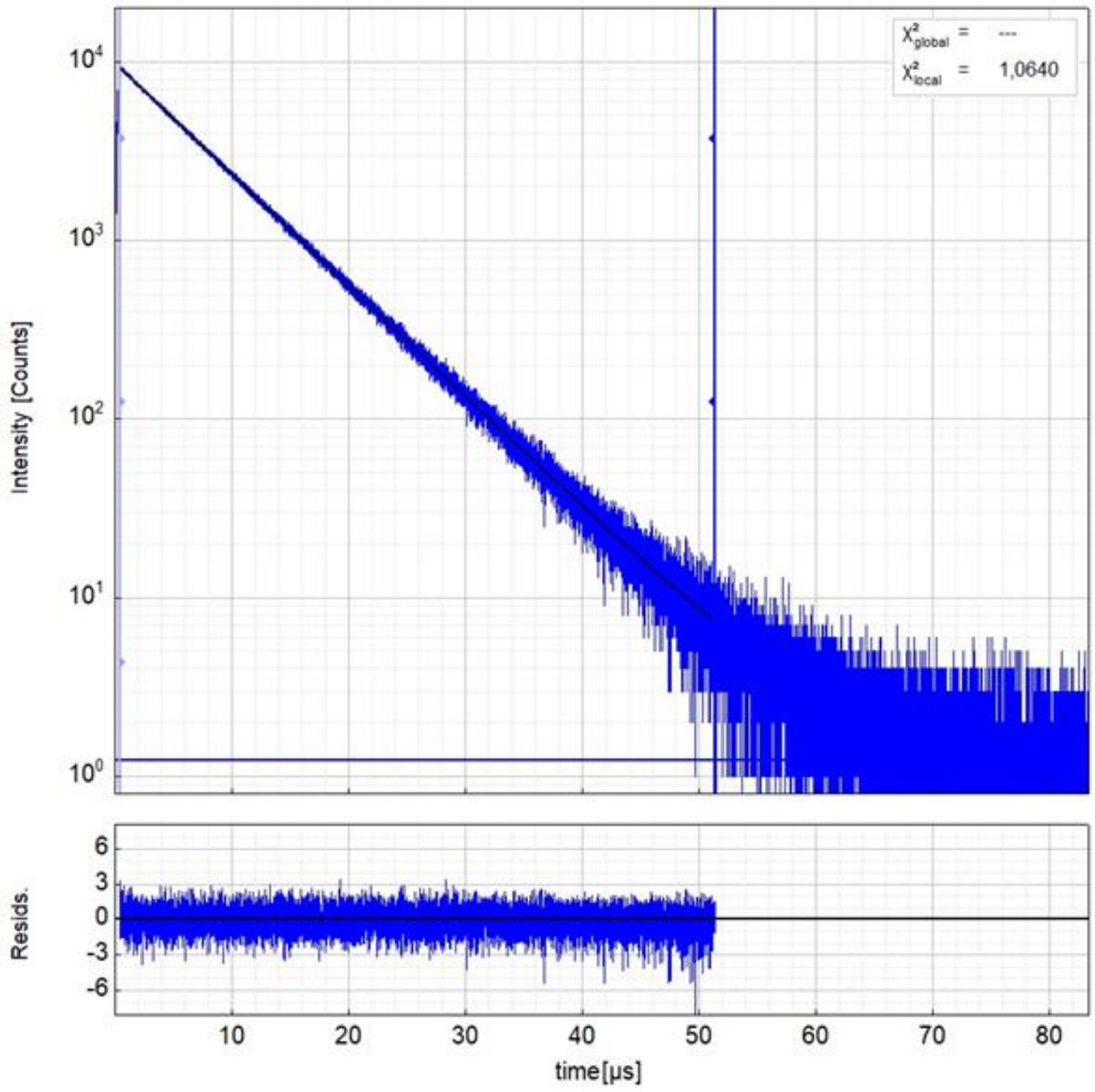

Parameter	Value	Δ	δ
A_1[kCnts/Chnl]	9,2540	±0,0091	0,1%
τ_1[ns]	6 960,4	±5,9	0,1%
I_1[kCnts]	16 103	±11	0,1%
A_{Rel1}[%]	100,0	---	---
I_{Rel1}[%]	100,0	---	---
$Bkgr_{Dec}$[kCnts]	0,0013	±0,0002	16%
τ_{AvInt}[ns]	6 960,4	±5,9	0,1%
τ_{AvAmp}[ns]	6 960,4	±5,9	0,1%

Figure S-320. Left: Raw (experimental) time-resolved photoluminescence decay of **Pt(L$_H$)PPh$_3$** in liquid DCM at 298 K (Ar-purged), including the residuals (λ_{exc} = 376.7 nm, λ_{em} = 491 nm). Right: Fitting parameters including pre-exponential factors and confidence limits.

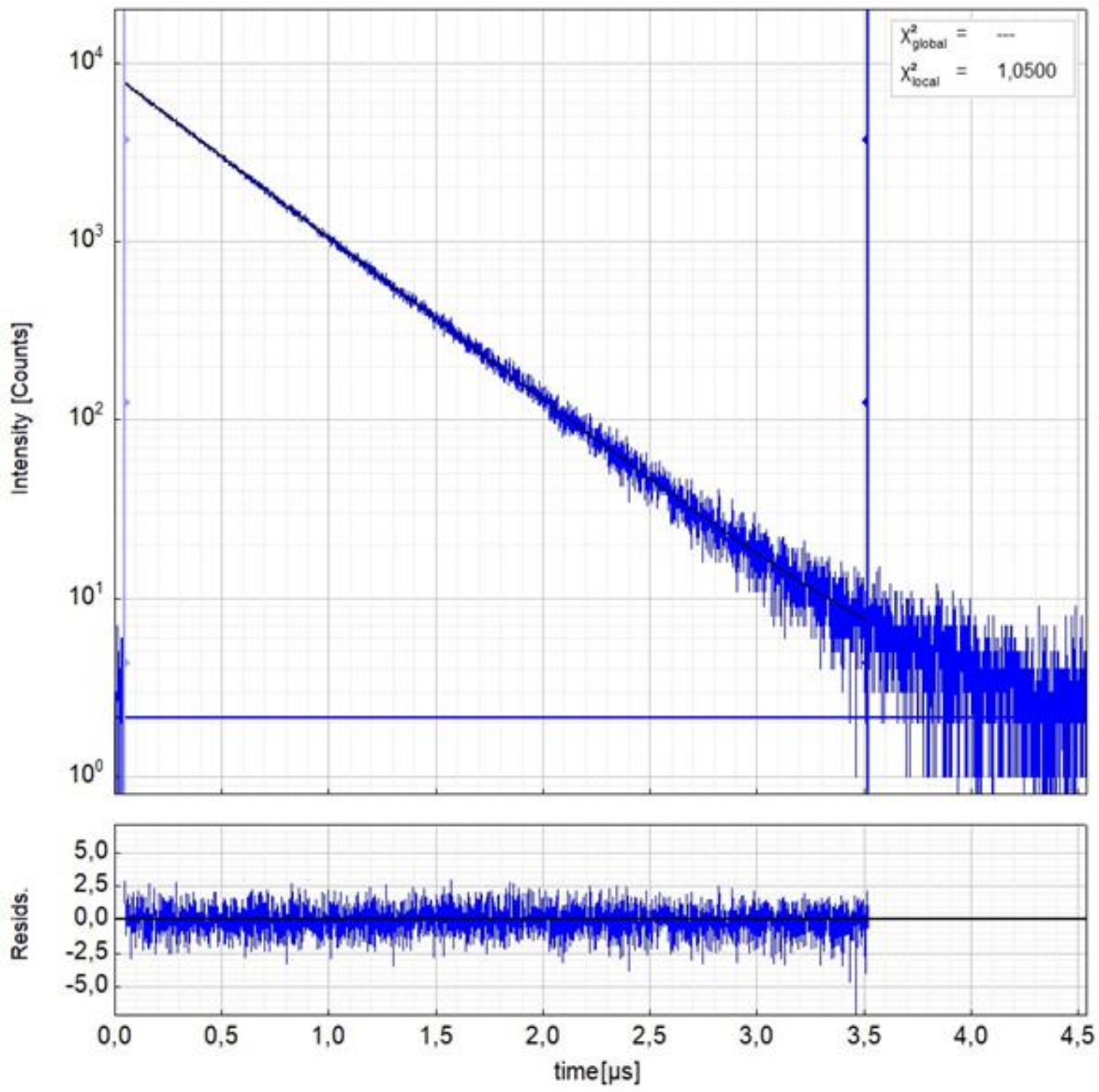

Parameter	Value	Δ	δ
A_1[kCnts/Chnl]	7,717	±0,017	0,2%
τ_1[ns]	476,52	±0,79	0,2%
I_1[kCnts]	3 677,0	±5,3	0,1%
A_{Rel1}[%]	100,0	---	---
I_{Rel1}[%]	100,0	---	---
$Bkgr_{Dec}$[kCnts]	0,0022	±0,0004	16%
τ_{AvInt}[ns]	476,52	±0,79	0,2%
τ_{AvAmp}[ns]	476,52	±0,79	0,2%

Figure S-321. Left: Raw (experimental) time-resolved photoluminescence decay of **Pt(L$_H$)PPh$_3$** in liquid DCM at 298 K (air-equilibrated), including the residuals (λ_{exc} = 376.7 nm, λ_{em} = 491 nm). Right: Fitting parameters including pre-exponential factors and confidence limits.

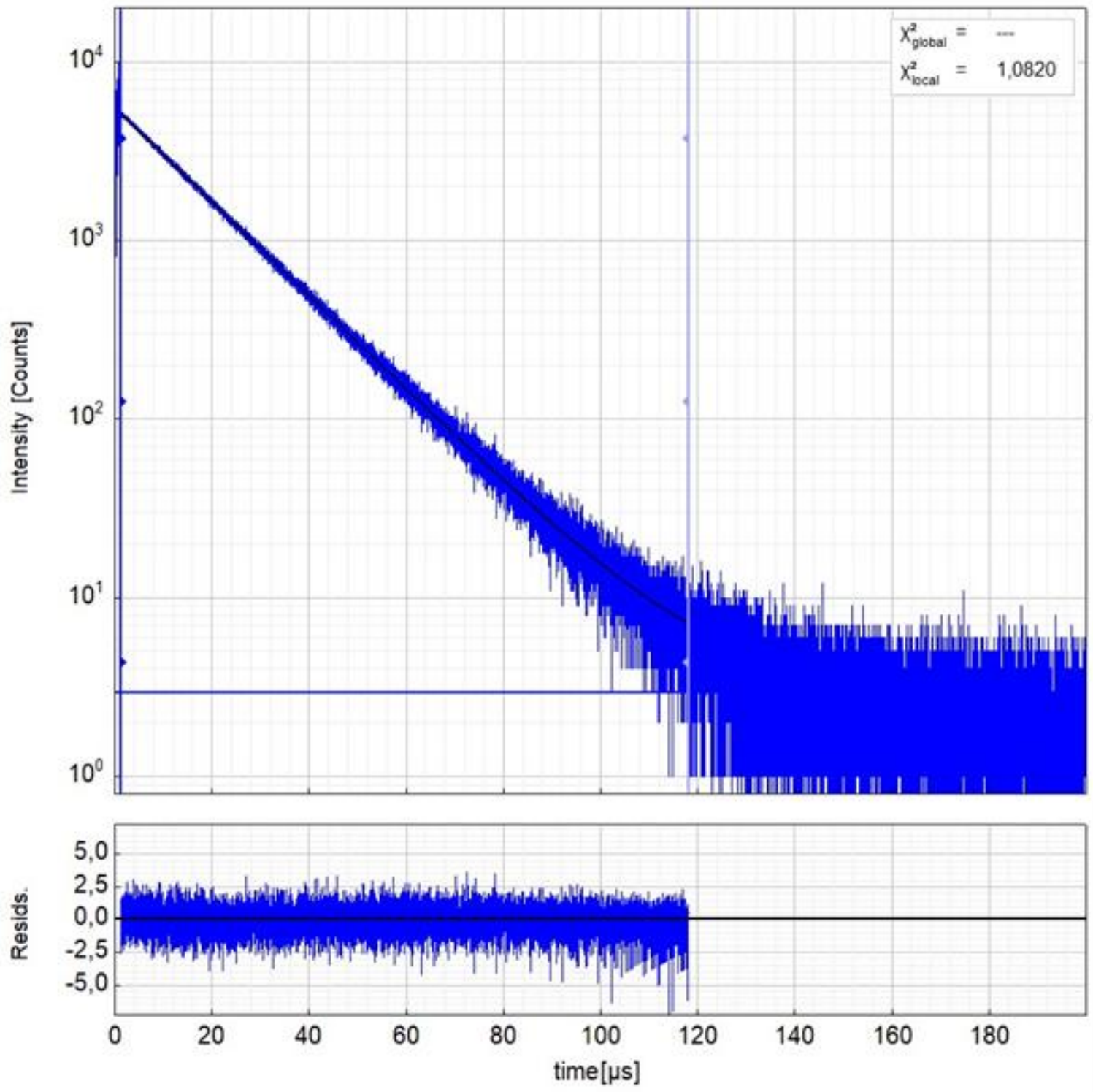

Parameter	Value	Δ	δ
A_1[kCnts/Chnl]	5,1787	±0,0061	0,1%
τ_1[ns]	16 433	±16	0,1%
I_1[kCnts]	10 637,3	±9,1	0,1%
A_{Rel1}[%]	100,0	---	---
I_{Rel1}[%]	100,0	---	---
$Bkgr_{Dec}$[kCnts]	0,0030	±0,0002	5,8%
τ_{AvInt}[ns]	16 433	±16	0,1%
τ_{AvAmp}[ns]	16 433	±16	0,1%

Figure S-322. Left: Raw (experimental) time-resolved photoluminescence decay of **Pt(L$_H$)PPh$_3$** in a frozen glassy matrix of DCM/MeOH (v/v = 1:1) at 77 K, including the residuals (λ_{exc} = 376.7 nm, λ_{em} = 491 nm). Right: Fitting parameters including pre-exponential factors and confidence limits.

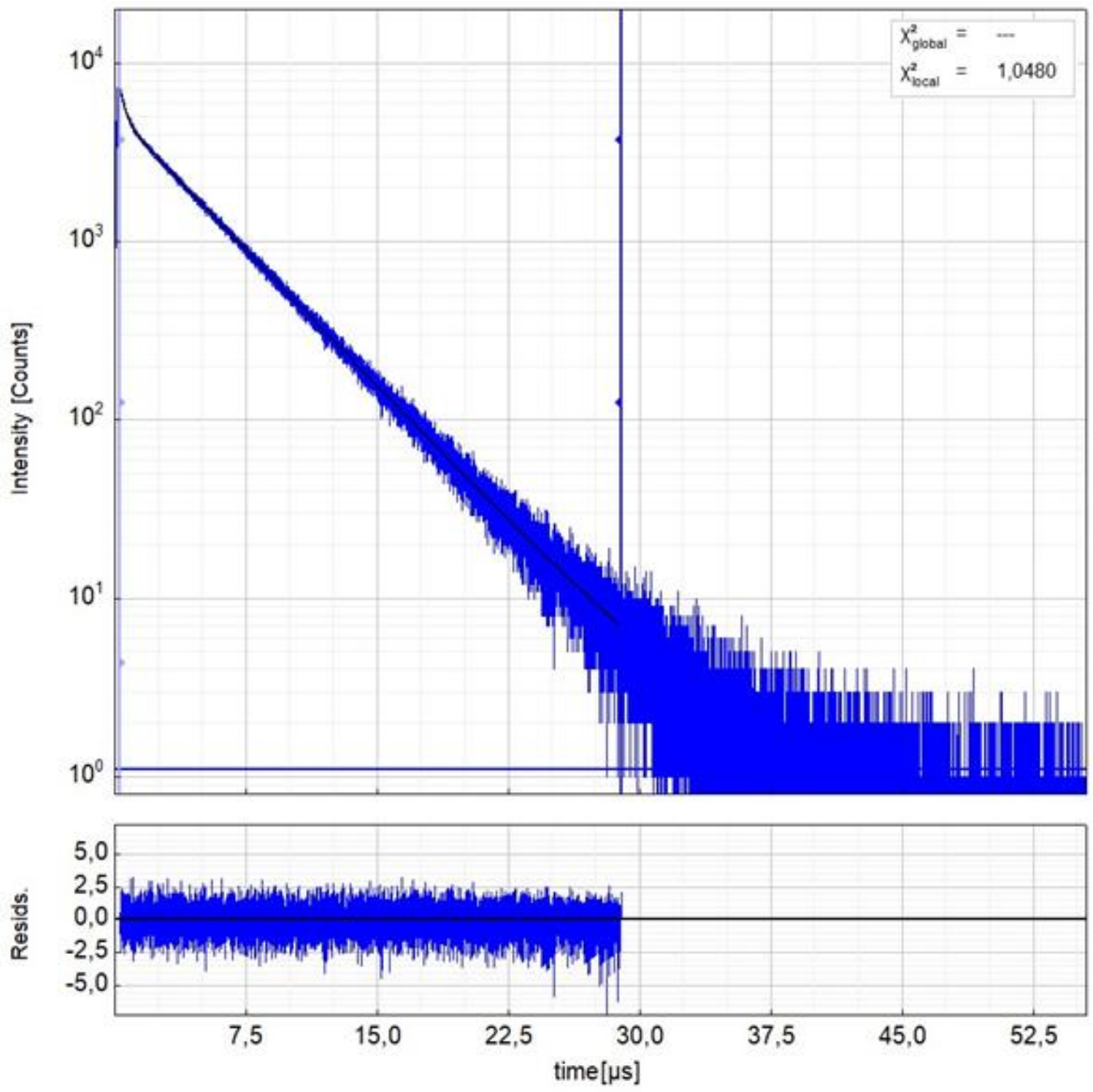

Parameter	Value	Δ	δ
A_1[kCnts/Chnl]	4,8409	±0,0089	0,2%
τ_1[ns]	4 263,2	±5,3	0,1%
I_1[kCnts]	10 319	±11	0,1%
A_{Rel1}[%]	68,2	±0,4	0,5%
I_{Rel1}[%]	96,3	±0,1	0,1%
A_2[kCnts/Chnl]	2,268	±0,033	1,4%
τ_2[ns]	352,2	±7,8	2,2%
I_2[kCnts]	399,2	±6,9	1,7%
A_{Rel2}[%]	31,9	±0,4	1,0%
I_{Rel2}[%]	3,8	±0,1	1,7%
$Bkgr_{Dec}$[kCnts]	0,0011	±0,0002	17%
τ_{AvInt}[ns]	4 117,5	±4,3	0,1%
τ_{AvAmp}[ns]	3 016	±15	0,5%

Figure S-323. Left: Raw (experimental) time-resolved photoluminescence decay of **Pt(L$_H$)AmPy** in liquid DCM at 298 K (Ar-purged), including the residuals (λ_{exc} = 376.7 nm, λ_{em} = 500 nm). Right: Fitting parameters including pre-exponential factors and confidence limits.

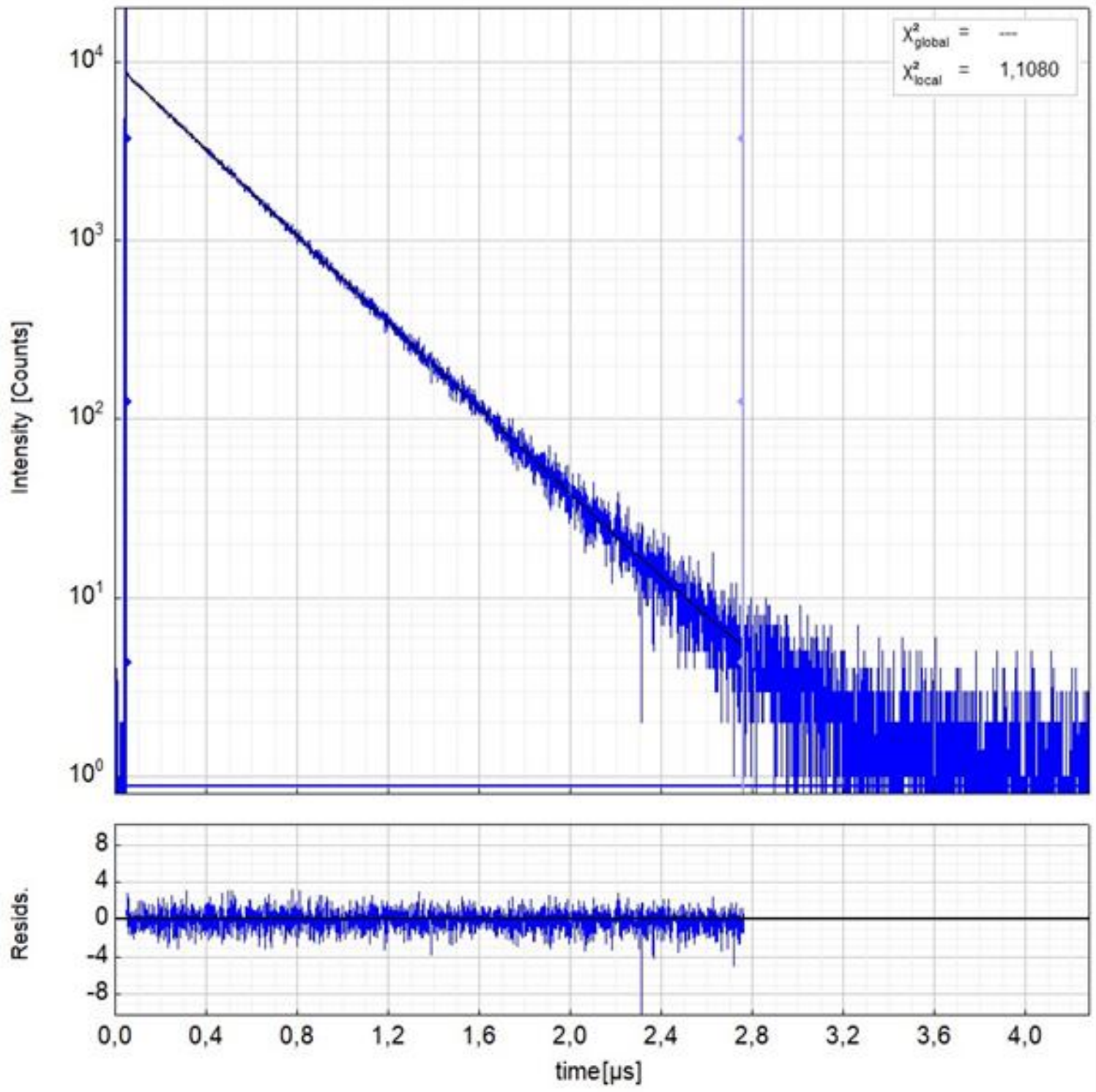

Parameter	Value	Δ	δ
A_1[kCnts/Chnl]	8,530	±0,019	0,2%
τ_1[ns]	358,88	±0,65	0,2%
I_1[kCnts]	3 061,0	±5,3	0,2%
A_{Rel1}[%]	100,0	---	---
I_{Rel1}[%]	100,0	---	---
$Bkgr_{Dec}$[kCnts]	0,0009	±0,0004	38%
τ_{AvInt}[ns]	358,88	±0,65	0,2%
τ_{AvAmp}[ns]	358,88	±0,65	0,2%

Figure S-324. Left: Raw (experimental) time-resolved photoluminescence decay of **Pt(L$_H$)AmPy** in liquid DCM at 298 K (air-equilibrated), including the residuals (λ_{exc} = 376.7 nm, λ_{em} = 500 nm). Right: Fitting parameters including pre-exponential factors and confidence limits.

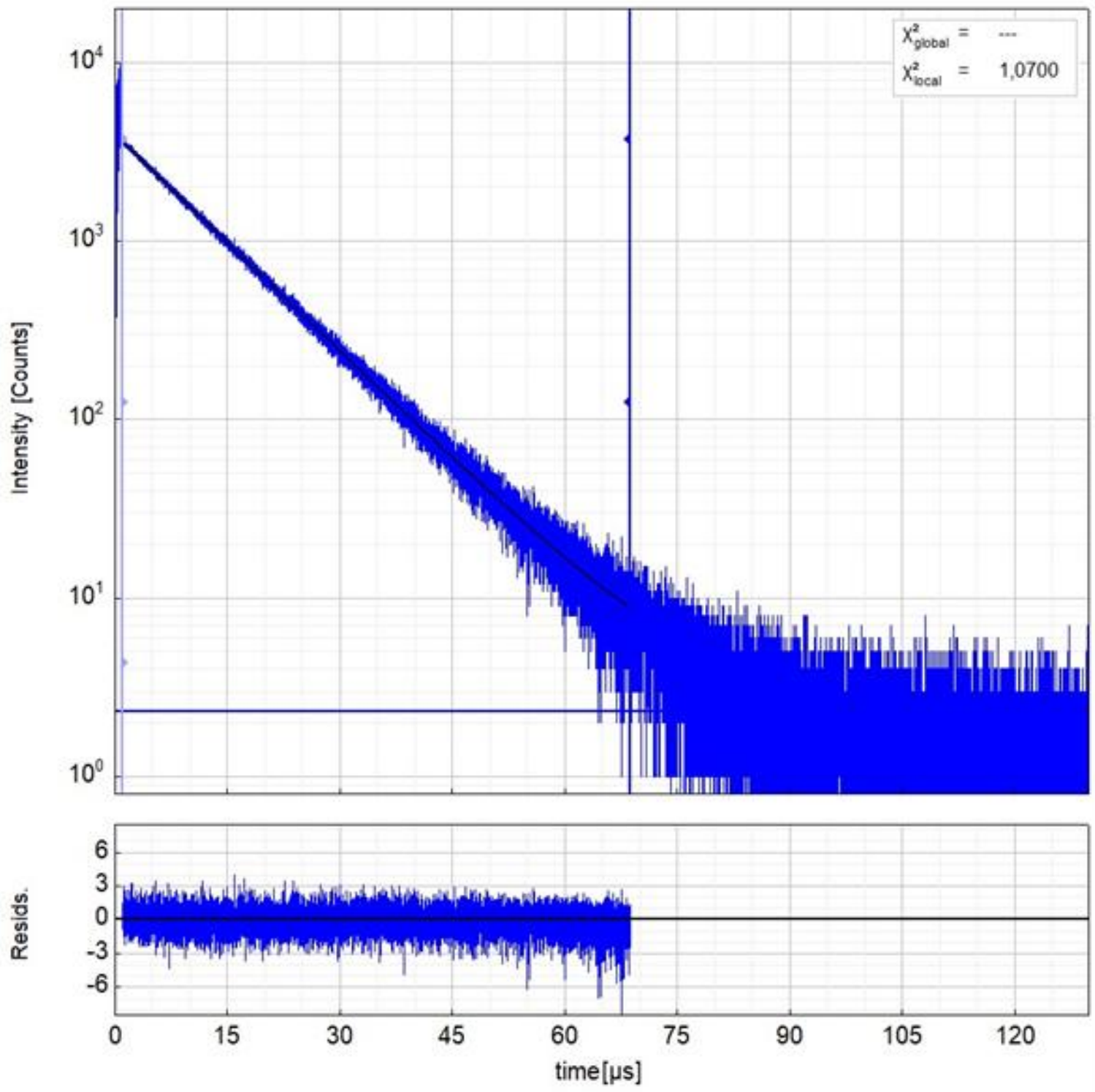

Parameter	Value	Δ	δ
A_1[kCnts/Chnl]	3,5827	±0,0045	0,1%
τ_1[ns]	10 713	±13	0,1%
I_1[kCnts]	9 594,7	±8,8	0,1%
A_{Rel1}[%]	100,0	---	---
I_{Rel1}[%]	100,0	---	---
$Bkgr_{Dec}$[kCnts]	0,0024	±0,0002	7,8%
τ_{AvInt}[ns]	10 713	±13	0,1%
τ_{AvAmp}[ns]	10 713	±13	0,1%

Figure S-325. Left: Raw (experimental) time-resolved photoluminescence decay of **Pt(L$_H$)AmPy** in a frozen glassy matrix of DCM/MeOH (v/v = 1:1) at 77 K, including the residuals (λ_{exc} = 376.7 nm, λ_{em} = 488 nm). Right: Fitting parameters including pre-exponential factors and confidence limits.

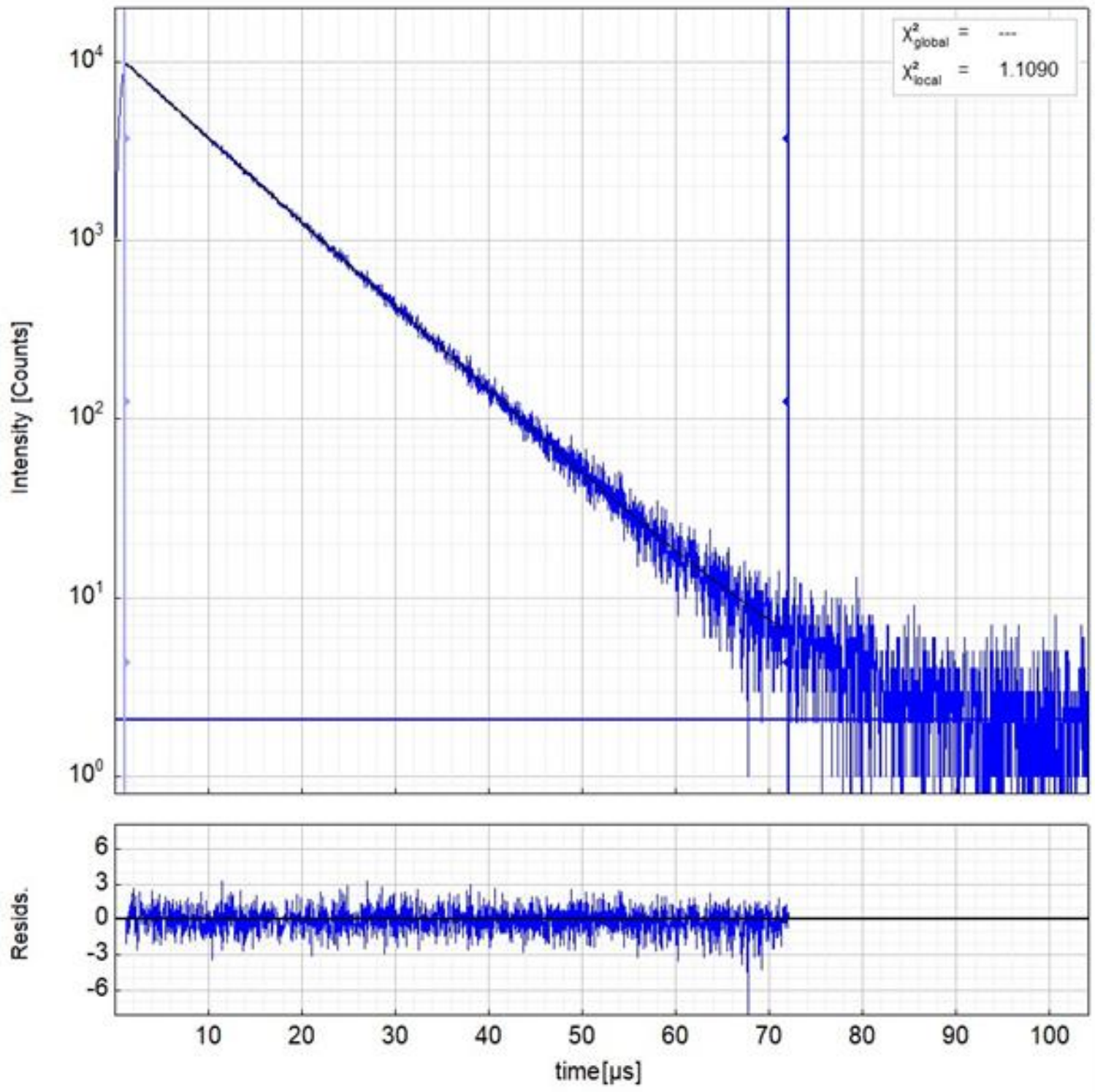

Parameter	Value	Δ	δ
A_1[kCnts/Chnl]	9.839	±0.017	0.2%
$τ_1$[ns]	9 187	±12	0.1%
I_1[kCnts]	2 824.4	±3.3	0.1%
A_{Rel1}[%]	100.0	---	---
I_{Rel1}[%]	100.0	---	---
$Bkgr_{Dec}$[kCnts]	0.0021	±0.0004	15%
$τ_{AvInt}$[ns]	9 187	±12	0.1%
$τ_{AvAmp}$[ns]	9 187	±12	0.1%

Figure S-326. Left: Raw (experimental) time-resolved photoluminescence decay of **Pt(L$_H$)NHC** in liquid DCM at 298 K (Ar-purged), including the residuals (λ_{exc} = 376.7 nm, λ_{em} = 492 nm). Right: Fitting parameters including pre-exponential factors and confidence limits.

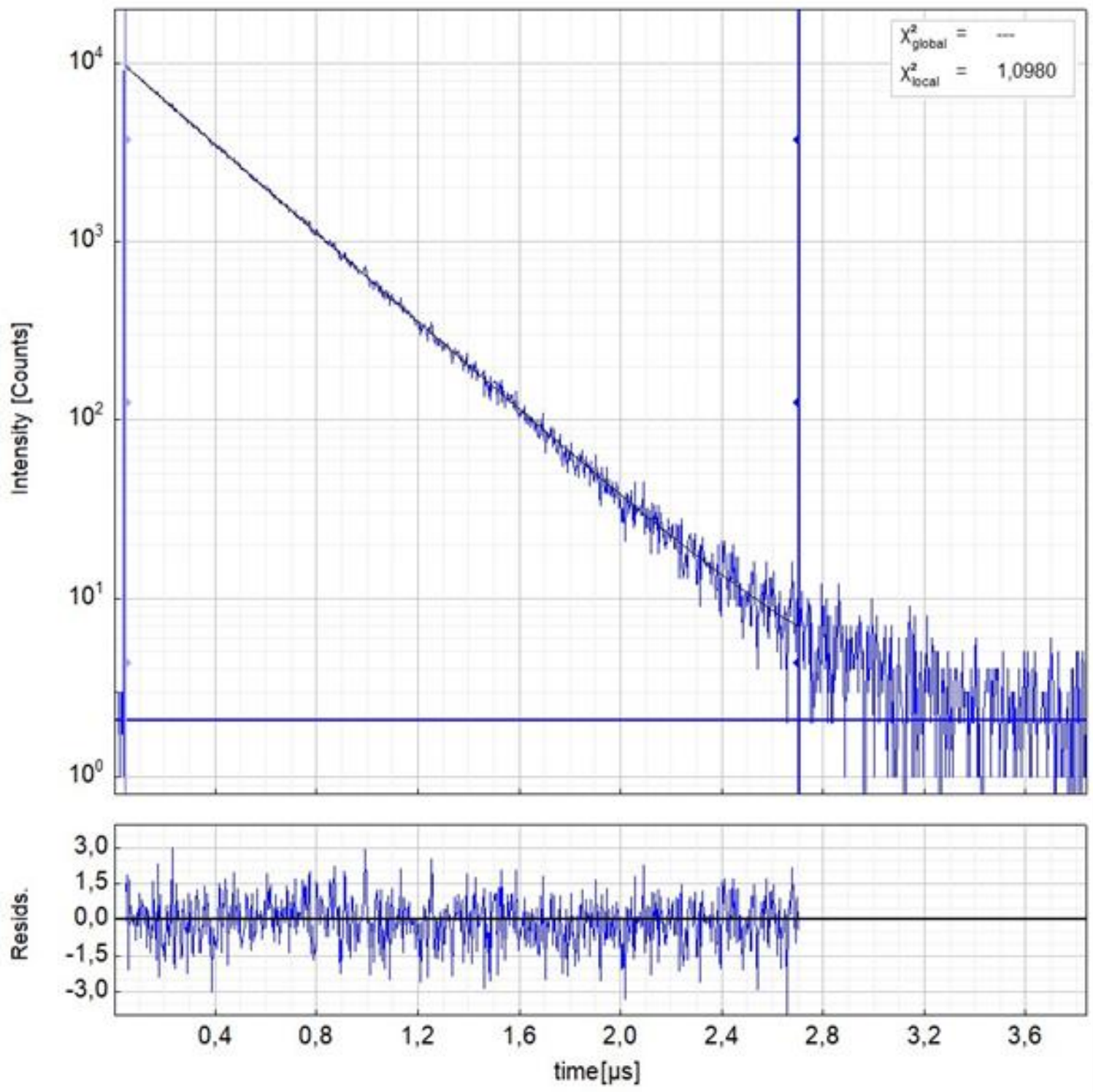

Parameter	Value	Δ	δ
A_1[kCnts/Chnl]	9,571	±0,031	0,3%
$τ_1$[ns]	349,81	±0,86	0,2%
I_1[kCnts]	837,0	±2,0	0,2%
A_{Rel1}[%]	100,0	---	---
I_{Rel1}[%]	100,0	---	---
$Bkgr_{Dec}$[kCnts]	0,0021	±0,0006	29%
$τ_{AvInt}$[ns]	349,81	±0,86	0,2%
$τ_{AvAmp}$[ns]	349,81	±0,86	0,2%

Figure S-327. Left: Raw (experimental) time-resolved photoluminescence decay of **Pt(L$_H$)NHC** in liquid DCM at 298 K (air-equilibrated), including the residuals (λ_{exc} = 376.7 nm, λ_{em} = 492 nm). Right: Fitting parameters including pre-exponential factors and confidence limits.

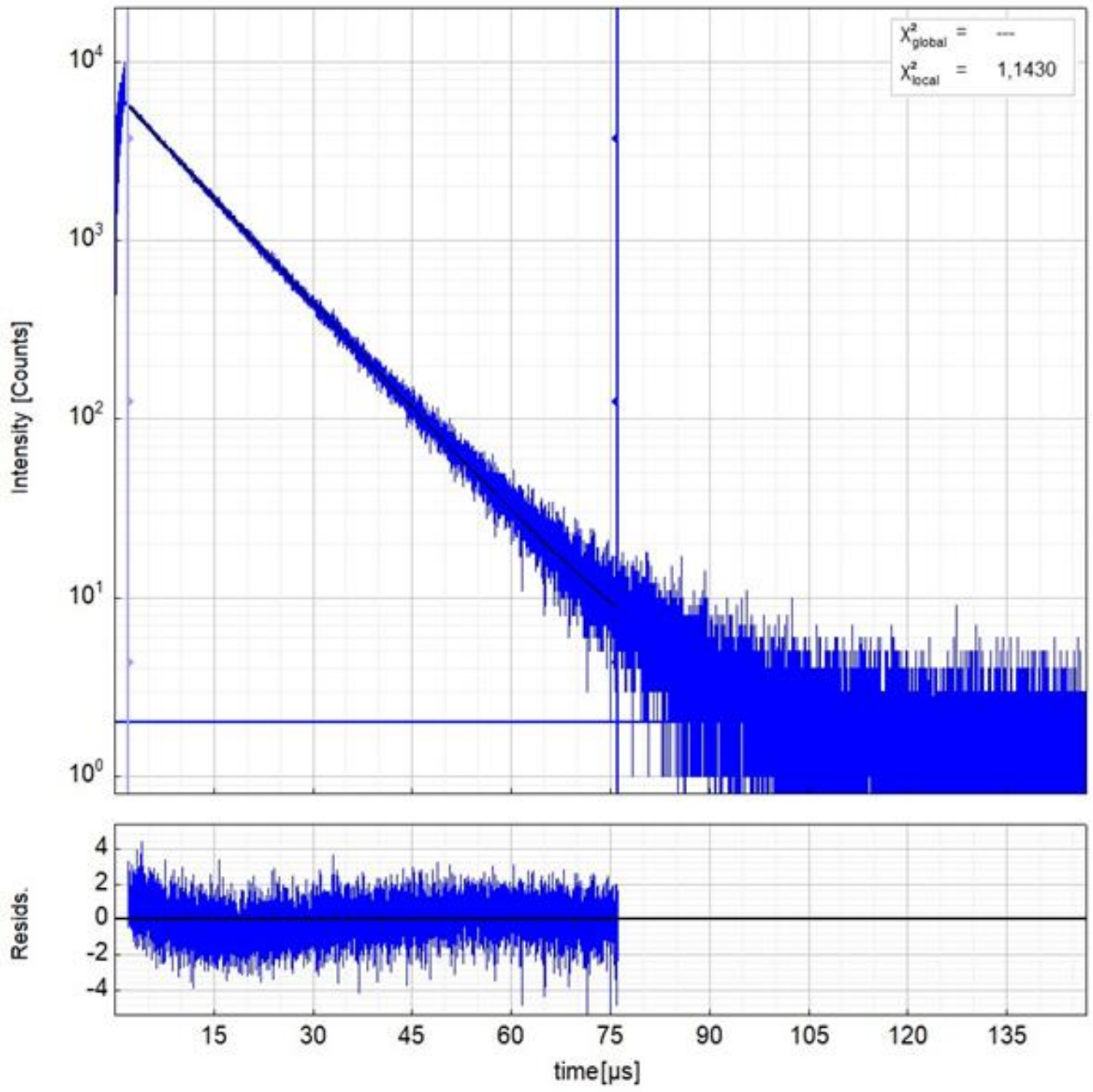

Parameter	Value	Δ	δ
A_1[kCnts/Chnl]	5,6638	±0,0069	0,1%
τ_1[ns]	11 000,8	±9,5	0,1%
I_1[kCnts]	7 788,3	±6,0	0,1%
A_{Rel1}[%]	100,0	---	---
I_{Rel1}[%]	100,0	---	---
$Bkgr_{Dec}$[kCnts]	0,0020	---	---
τ_{AvInt}[ns]	11 000,8	±9,5	0,1%
τ_{AvAmp}[ns]	11 000,8	±9,5	0,1%

Figure S-328. Left: Raw (experimental) time-resolved photoluminescence decay of **Pt(L_H)NHC** in a frozen glassy matrix of DCM/MeOH (v/v = 1:1) at 77 K, including the residuals (λ_{exc} = 376.7 nm, λ_{em} = 484 nm). Right: Fitting parameters including pre-exponential factors and confidence limits.

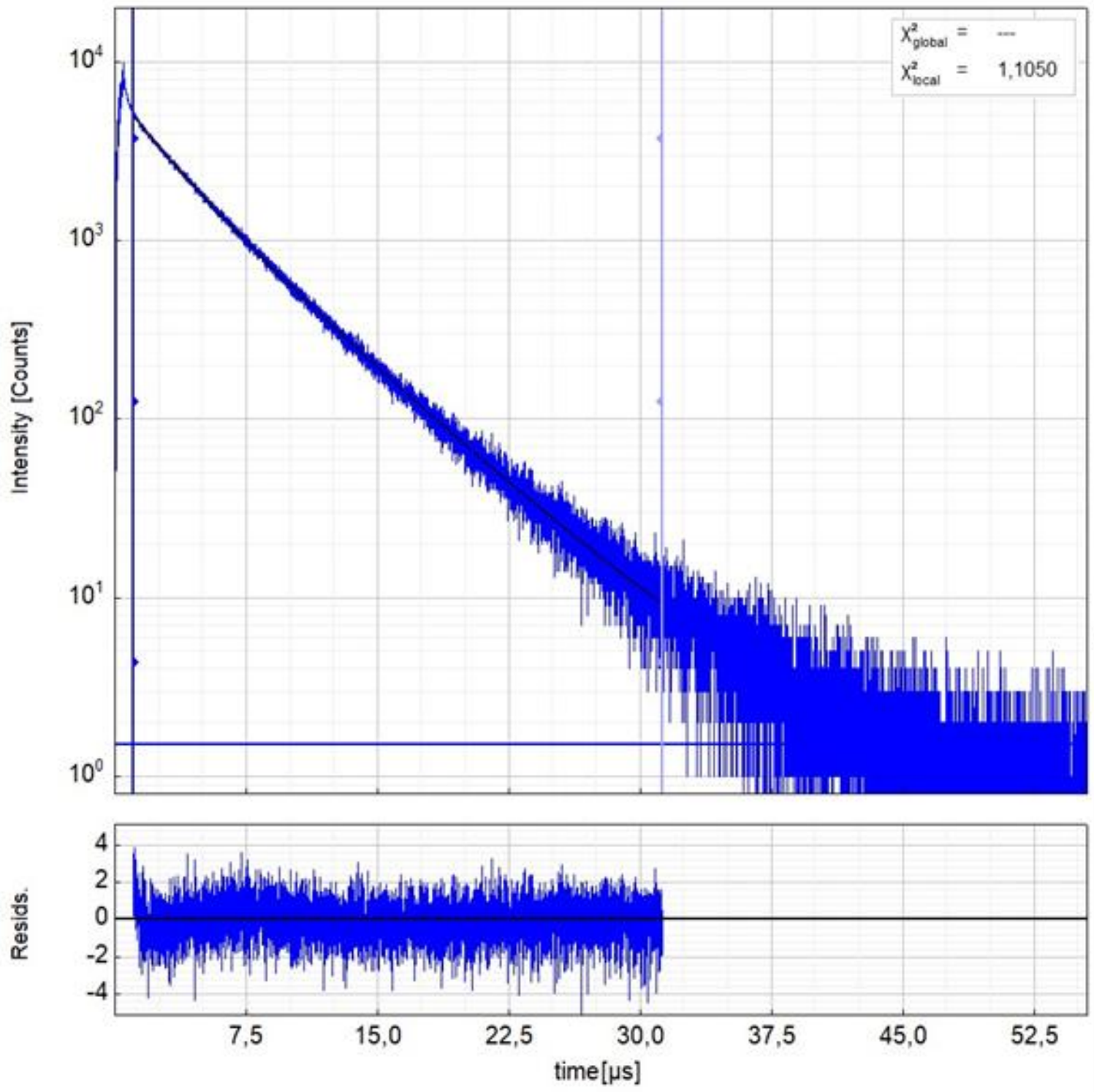

Parameter	Value	Δ	δ
A_1[kCnts/Chnl]	2,65	±0,22	8,0%
τ_1[ns]	5 178	±94	1,8%
I_1[kCnts]	3 430	±210	6,0%
A_{Rel1}[%]	53,5	±4,1	7,6%
I_{Rel1}[%]	68,4	±4,1	6,0%
A_2[kCnts/Chnl]	2,31	±0,20	8,3%
τ_2[ns]	2 750	±140	4,9%
I_2[kCnts]	1 590	±210	13%
A_{Rel2}[%]	46,6	±4,1	8,7%
I_{Rel2}[%]	31,7	±4,1	13%
$Bkgr_{Dec}$[kCnts]	0,0015	---	---
τ_{AvInt}[ns]	4 409,0	±6,4	0,1%
τ_{AvAmp}[ns]	4 046	±14	0,3%

Figure S-329. Left: Raw (experimental) time-resolved photoluminescence decay of **Pt(L_{tBu})Cl** in liquid DCM at 298 K (Ar-purged), including the residuals (λ_{exc} = 376.7 nm, λ_{em} = 505 nm). Right: Fitting parameters including pre-exponential factors and confidence limits.

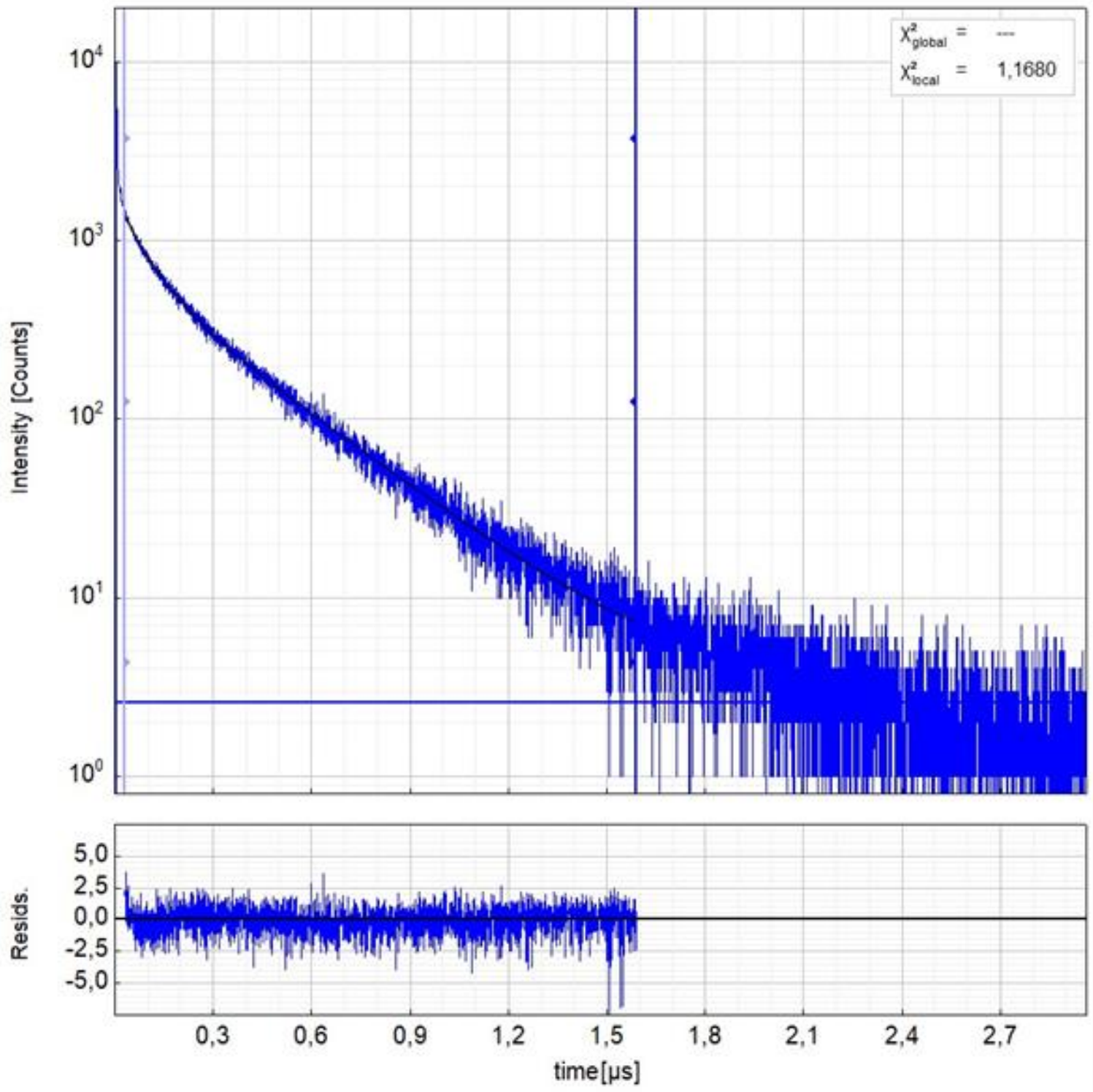

Parameter	Value	Δ	δ
A_1[kCnts/Chnl]	0,624	±0,020	3,1%
τ_1[ns]	316,9	±4,0	1,2%
I_1[kCnts]	385,7	±7,5	1,9%
A_{Rel1}[%]	46,8	±1,1	2,2%
I_{Rel1}[%]	77,3	±1,4	1,7%
A_2[kCnts/Chnl]	0,709	±0,014	1,9%
τ_2[ns]	81,9	±3,8	4,6%
I_2[kCnts]	113,4	±6,5	5,7%
A_{Rel2}[%]	53,3	±1,1	2,0%
I_{Rel2}[%]	22,8	±1,4	5,9%
$Bkgr_{Dec}$[kCnts]	0,0026	---	---
τ_{AvInt}[ns]	263,49	±0,76	0,3%
τ_{AvAmp}[ns]	191,8	±1,5	0,7%

Figure S-330. Left: Raw (experimental) time-resolved photoluminescence decay of **Pt(L_{tBu})Cl** in liquid DCM at 298 K (air-equilibrated), including the residuals (λ_{exc} = 376.7 nm, λ_{em} = 505 nm). Right: Fitting parameters including pre-exponential factors and confidence limits.

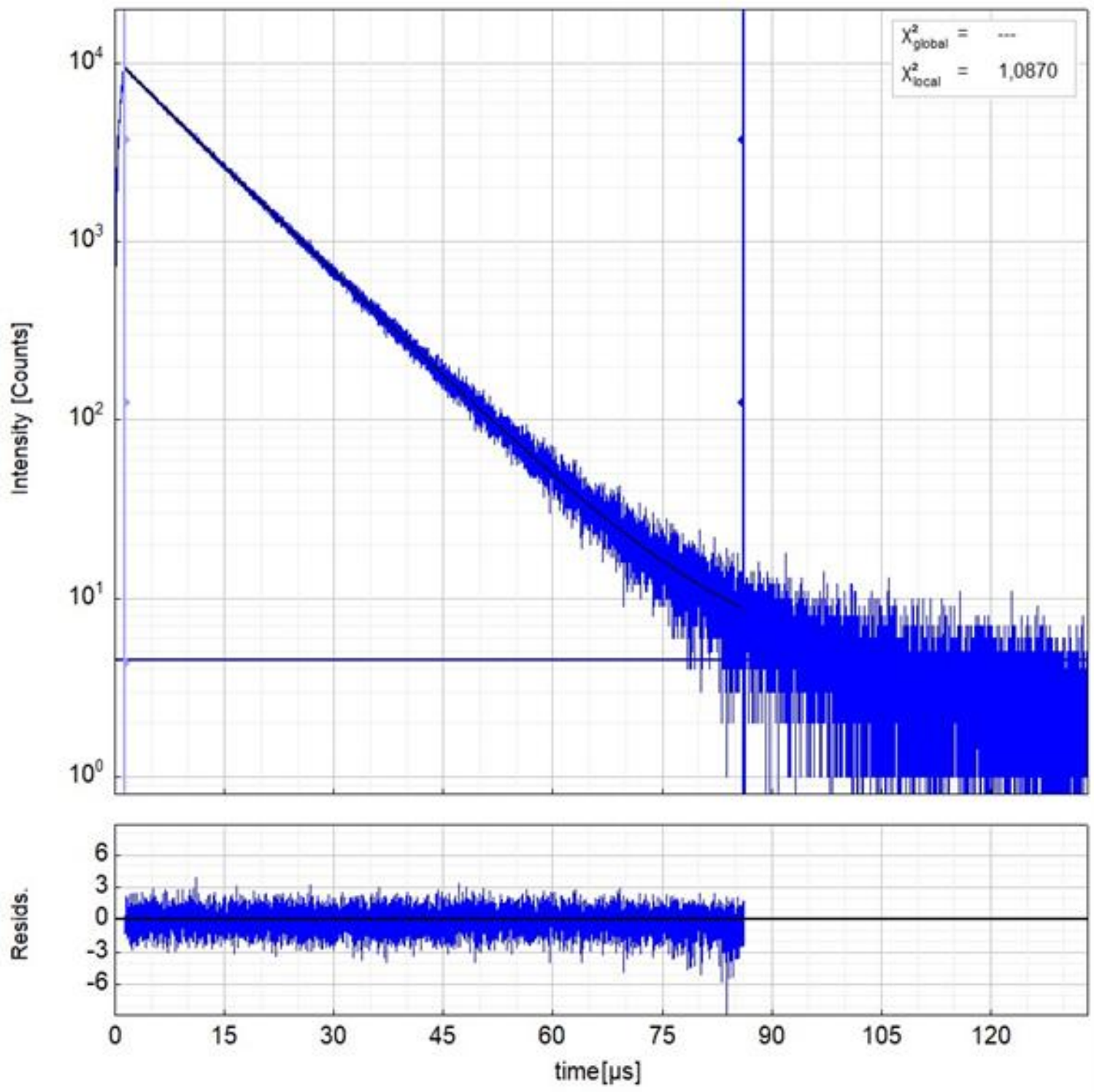

Parameter	Value	Δ	δ
A_1[kCnts/Chnl]	8,736	±0,081	0,9%
τ_1[ns]	11 132	±27	0,2%
I_1[kCnts]	12 156	±85	0,7%
A_{Rel1}[%]	93,3	±0,9	0,9%
I_{Rel1}[%]	96,5	±0,7	0,7%
A_2[kCnts/Chnl]	0,628	±0,079	12%
τ_2[ns]	5 680	±460	8,0%
I_2[kCnts]	446	±86	19%
A_{Rel2}[%]	6,8	±0,9	13%
I_{Rel2}[%]	3,6	±0,7	19%
$Bkgr_{Dec}$[kCnts]	0,0045	---	---
τ_{AvInt}[ns]	10 939,1	±7,5	0,1%
τ_{AvAmp}[ns]	10 767	±18	0,2%

Figure S-331. Left: Raw (experimental) time-resolved photoluminescence decay of **Pt(L_{tBu})Cl** in a frozen glassy matrix of DCM/MeOH (v/v = 1:1) at 77 K, including the residuals (λ_{exc} = 376.7 nm, λ_{em} = 500 nm). Right: Fitting parameters including pre-exponential factors and confidence limits.

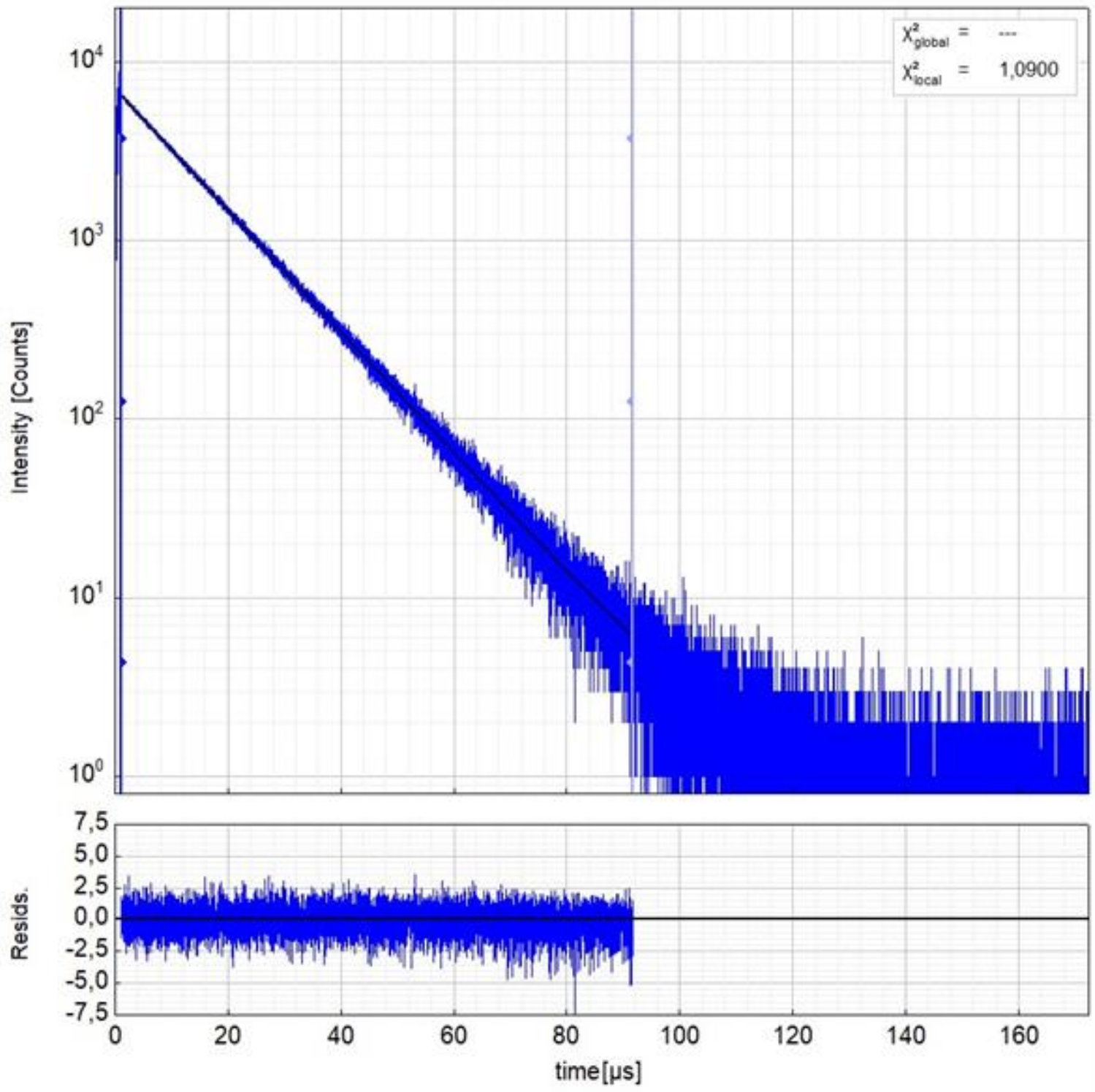

Parameter	Value	Δ	δ
A_1[kCnts/Chnl]	6,5054	±0,0028	0,0%
τ_1[ns]	12 784,8	±5,3	0,0%
I_1[kCnts]	10 396,1	±4,0	0,0%
A_{Rel1}[%]	100,0	---	---
I_{Rel1}[%]	100,0	---	---
$Bkgr_{Dec}$[kCnts]	0,0007	±0,0002	17%
τ_{AvInt}[ns]	12 784,8	±5,3	0,0%
τ_{AvAmp}[ns]	12 784,8	±5,3	0,0%

Figure S-332. Left: Raw (experimental) time-resolved photoluminescence decay of **Pt(L$_{tBu}$)PPh$_3$** in liquid DCM at 298 K (Ar-purged), including the residuals (λ_{exc} = 376.7 nm, λ_{em} = 500 nm). Right: Fitting parameters including pre-exponential factors and confidence limits.

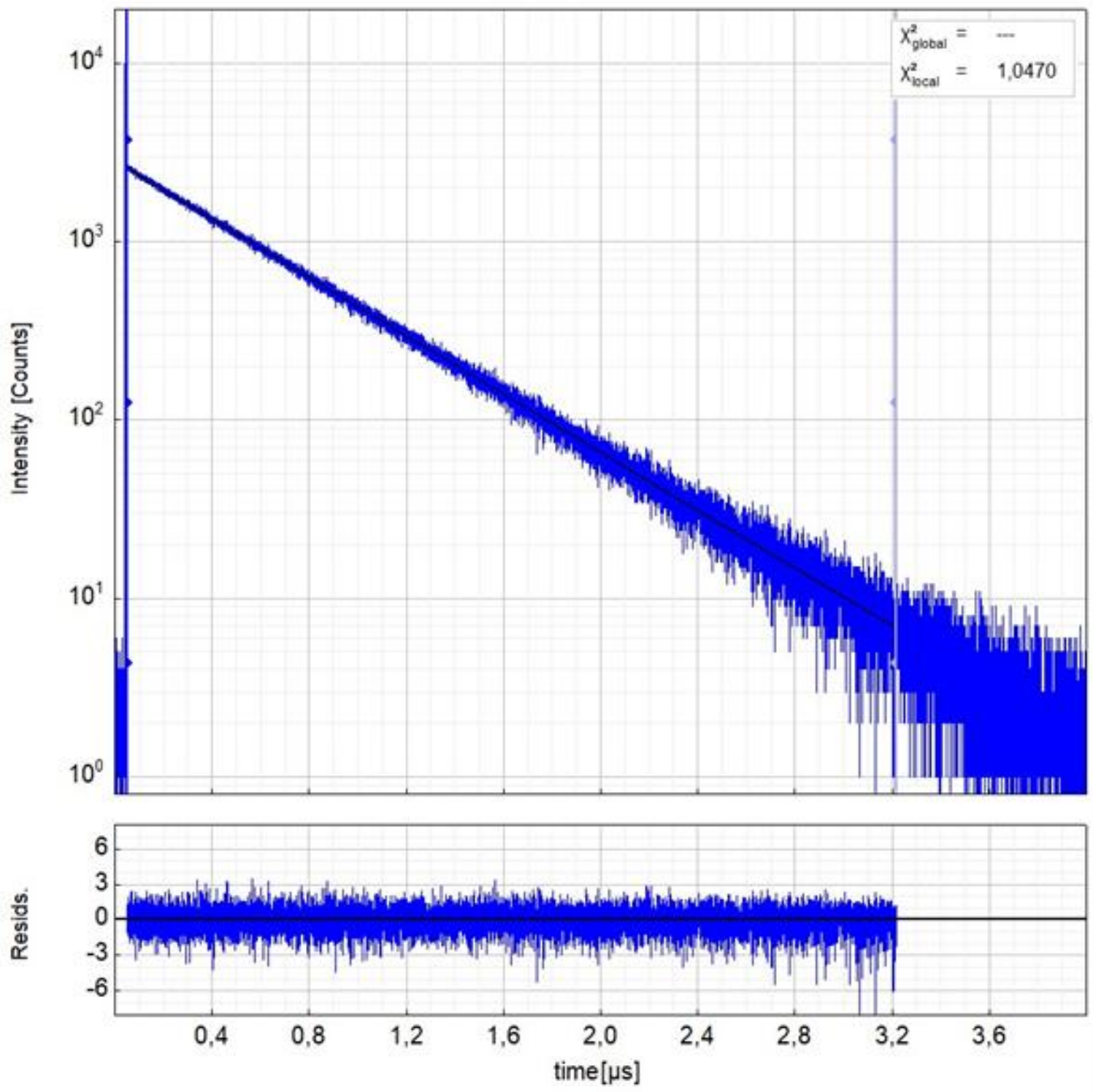

Parameter	Value	Δ	δ
A_1[kCnts/Chnl]	2,5898	±0,0042	0,2%
τ_1[ns]	530,21	±0,83	0,2%
I_1[kCnts]	5 492,5	±6,8	0,1%
A_{Rel1}[%]	100,0	---	---
I_{Rel1}[%]	100,0	---	---
$Bkgr_{Dec}$[kCnts]	0,0003	±0,0002	74%
τ_{AvInt}[ns]	530,21	±0,83	0,2%
τ_{AvAmp}[ns]	530,21	±0,83	0,2%

Figure S-333. Left: Raw (experimental) time-resolved photoluminescence decay of **Pt(L$_{tBu}$)PPh$_3$** in liquid DCM at 298 K (air-equilibrated), including the residuals (λ_{exc} = 376.7 nm, λ_{em} = 500 nm). Right: Fitting parameters including pre-exponential factors and confidence limits.

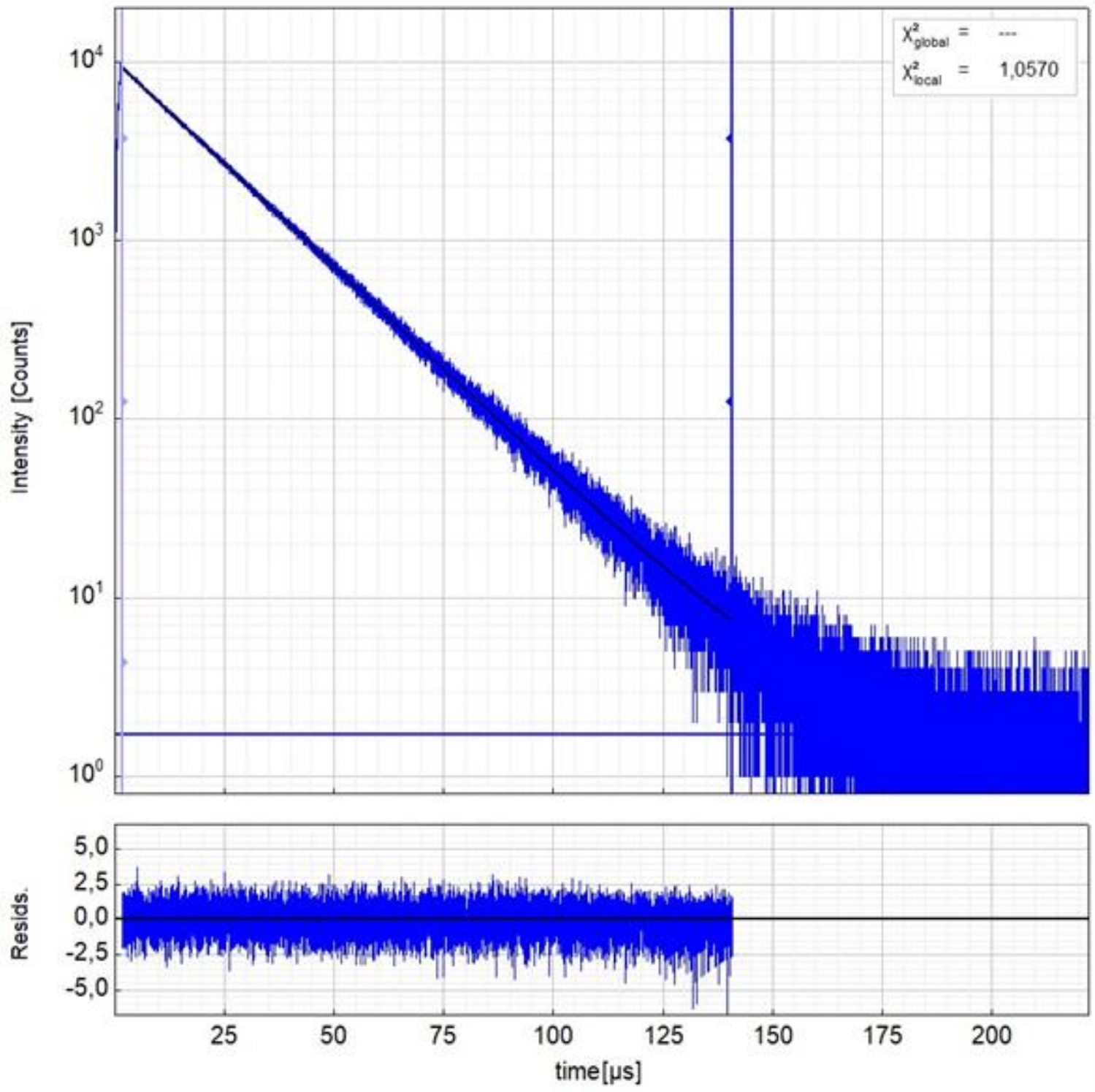

Parameter	Value	Δ	δ
A_1[kCnts/Chnl]	9,2468	±0,0084	0,1%
τ_1[ns]	18 842	±14	0,1%
I_1[kCnts]	21 777,9	±9,7	0,0%
A_{Rel1}[%]	100,0	---	---
I_{Rel1}[%]	100,0	---	---
$Bkgr_{Dec}$[kCnts]	0,0017	±0,0002	8,7%
τ_{AvInt}[ns]	18 842	±14	0,1%
τ_{AvAmp}[ns]	18 842	±14	0,1%

Figure S-334. Left: Raw (experimental) time-resolved photoluminescence decay of **Pt(L_{tBu})PPh_3** in a frozen glassy matrix of DCM/MeOH (v/v = 1:1) at 77 K, including the residuals (λ_{exc} = 376.7 nm, λ_{em} = 484 nm). Right: Fitting parameters including pre-exponential factors and confidence limits.

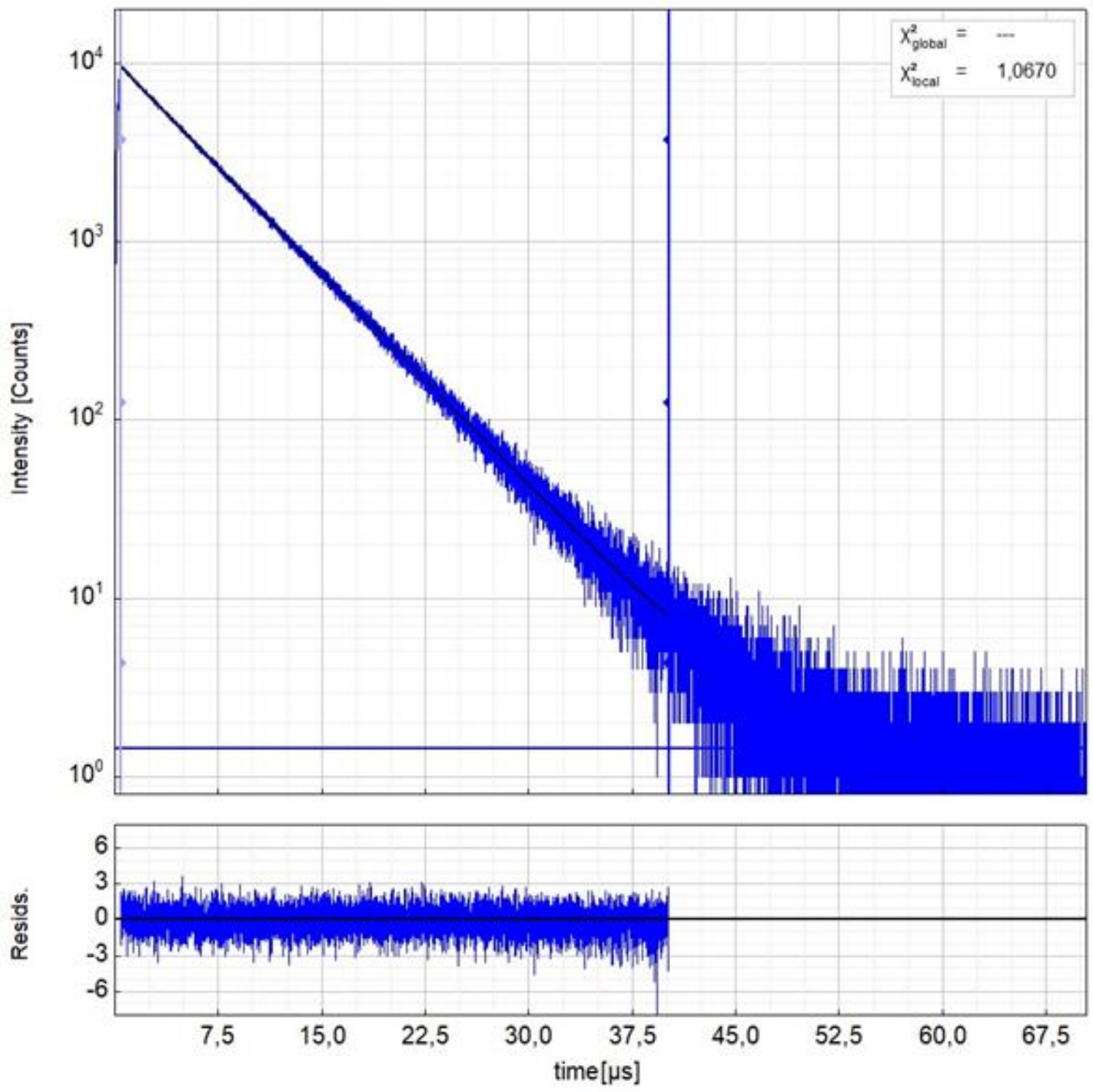

Parameter	Value	Δ	δ
A_1[kCnts/Chnl]	9,529	±0,011	0,1%
τ_1[ns]	5 443,4	±4,8	0,1%
I_1[kCnts]	12 967,1	±9,4	0,1%
A_{Rel1}[%]	100,0	---	---
I_{Rel1}[%]	100,0	---	---
$Bkgr_{Dec}$[kCnts]	0,0015	±0,0003	16%
τ_{AvInt}[ns]	5 443,4	±4,8	0,1%
τ_{AvAmp}[ns]	5 443,4	±4,8	0,1%

Figure S-335. Left: Raw (experimental) time-resolved photoluminescence decay of **Pt(L_{tBu})AmPy** in liquid DCM at 298 K (Ar-purged), including the residuals (λ_{exc} = 376.7 nm, λ_{em} = 504 nm). Right: Fitting parameters including pre-exponential factors and confidence limits.

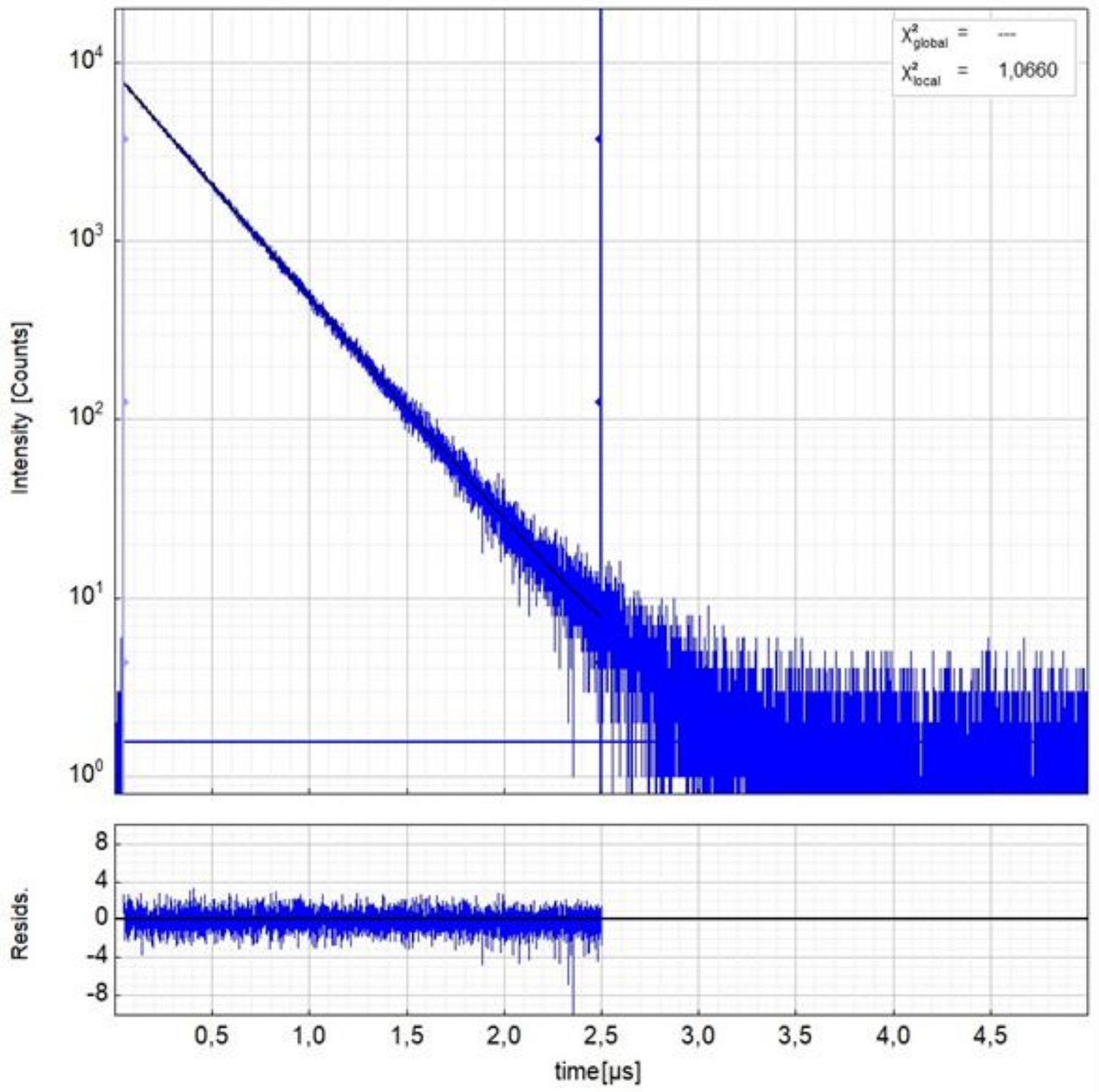

Parameter	Value	Δ	δ
A_1[kCnts/Chnl]	7,645	±0,013	0,2%
τ_1[ns]	344,89	±0,47	0,1%
I_1[kCnts]	5 272,8	±6,6	0,1%
A_{Rel1}[%]	100,0	---	---
I_{Rel1}[%]	100,0	---	---
$Bkgr_{Dec}$[kCnts]	0,0016	±0,0004	20%
τ_{AvInt}[ns]	344,89	±0,47	0,1%
τ_{AvAmp}[ns]	344,89	±0,47	0,1%

Figure S-336. Left: Raw (experimental) time-resolved photoluminescence decay of **Pt(L_{tBu})AmPy** in liquid DCM at 298 K (air-equilibrated), including the residuals (λ_{exc} = 376.7 nm, λ_{em} = 504 nm). Right: Fitting parameters including pre-exponential factors and confidence limits.

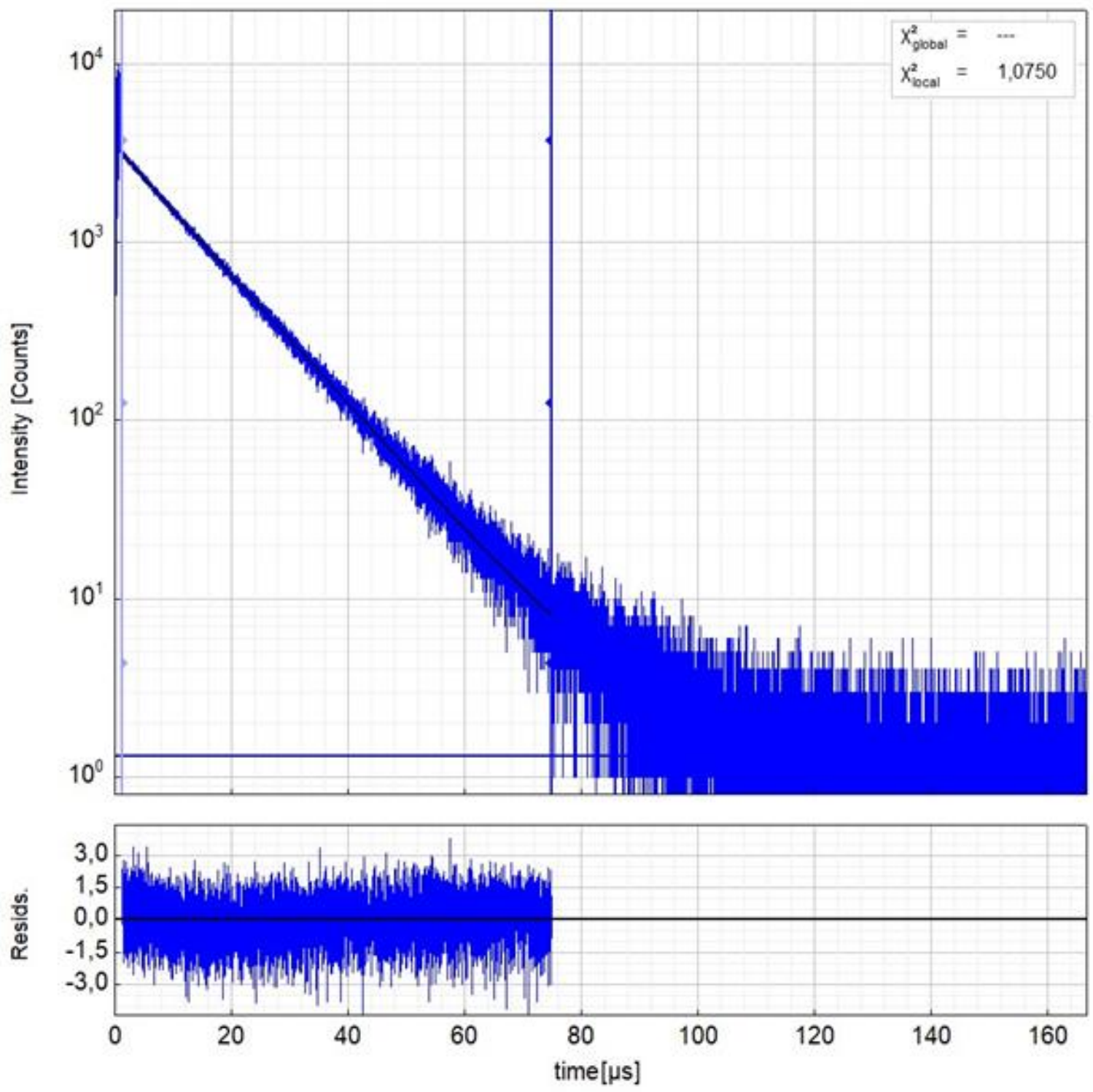

Parameter	Value	Δ	δ
A_1[kCnts/Chnl]	3,1025	±0,0036	0,1%
τ_1[ns]	11 998,7	±8,5	0,1%
I_1[kCnts]	4 653,2	±3,1	0,1%
A_{Rel1}[%]	100,0	---	---
I_{Rel1}[%]	100,0	---	---
$Bkgr_{Dec}$[kCnts]	0,0013	---	---
τ_{AvInt}[ns]	11 998,7	±8,5	0,1%
τ_{AvAmp}[ns]	11 998,7	±8,5	0,1%

Figure S-337. Left: Raw (experimental) time-resolved photoluminescence decay of **Pt(L_{tBu})AmPy** in a frozen glassy matrix of DCM/MeOH (v/v = 1:1) at 77 K, including the residuals (λ_{exc} = 376.7 nm, λ_{em} = 493 nm). Right: Fitting parameters including pre-exponential factors and confidence limits.

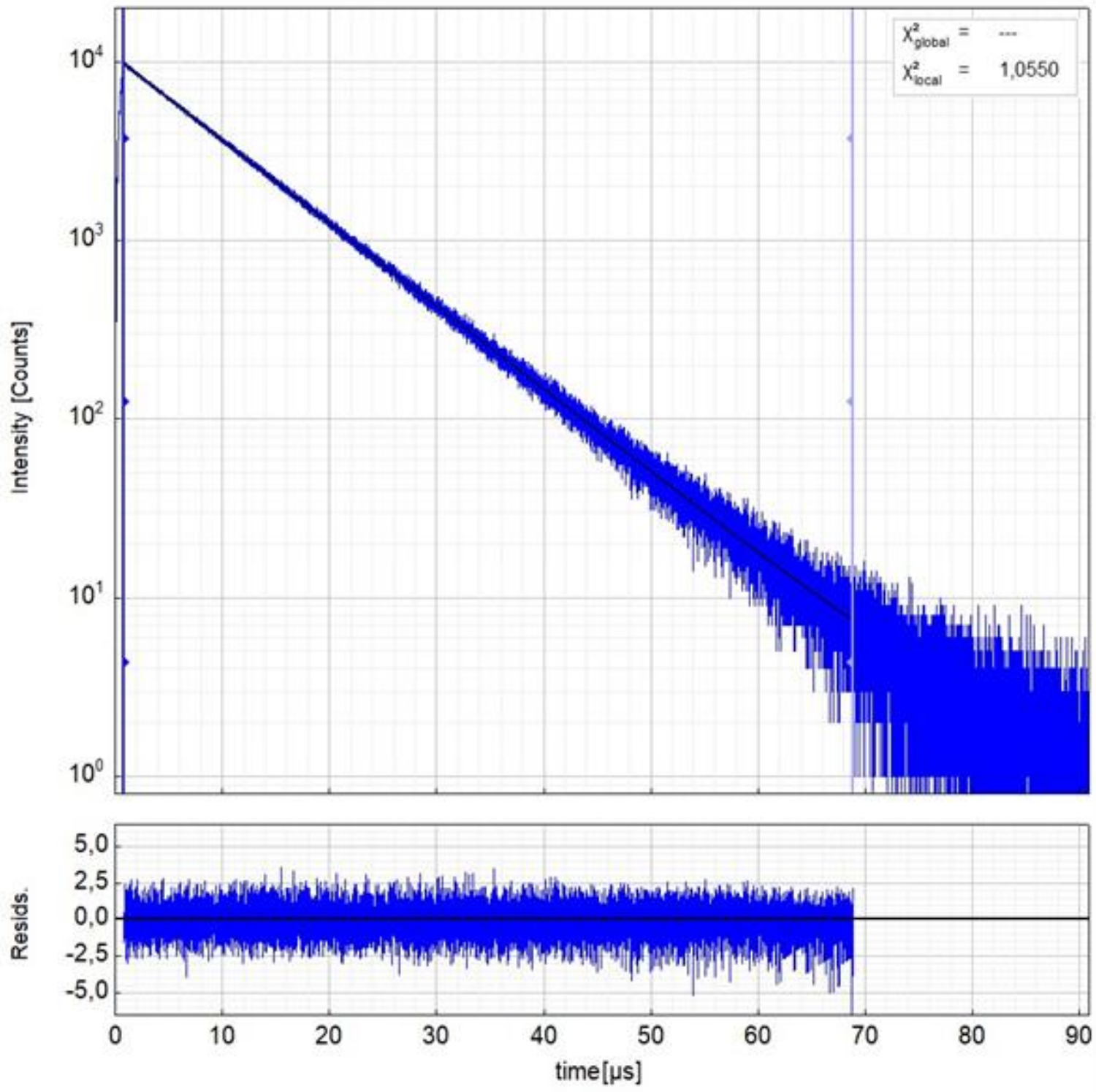

Parameter	Value	Δ	δ
A_1[kCnts/Chnl]	9,7168	±0,0079	0,1%
τ_1[ns]	9 335,9	±6,3	0,1%
I_1[kCnts]	22 679	±13	0,1%
A_{Rel1}[%]	100,0	---	---
I_{Rel1}[%]	100,0	---	---
$Bkgr_{Dec}$[kCnts]	0,0008	±0,0002	21%
τ_{AvInt}[ns]	9 335,9	±6,3	0,1%
τ_{AvAmp}[ns]	9 335,9	±6,3	0,1%

Figure S-338. Left: Raw (experimental) time-resolved photoluminescence decay of **Pt(L_{tBu})NHC** in liquid DCM at 298 K (Ar-purged), including the residuals (λ_{exc} = 376.7 nm, λ_{em} = 496 nm). Right: Fitting parameters including pre-exponential factors and confidence limits.

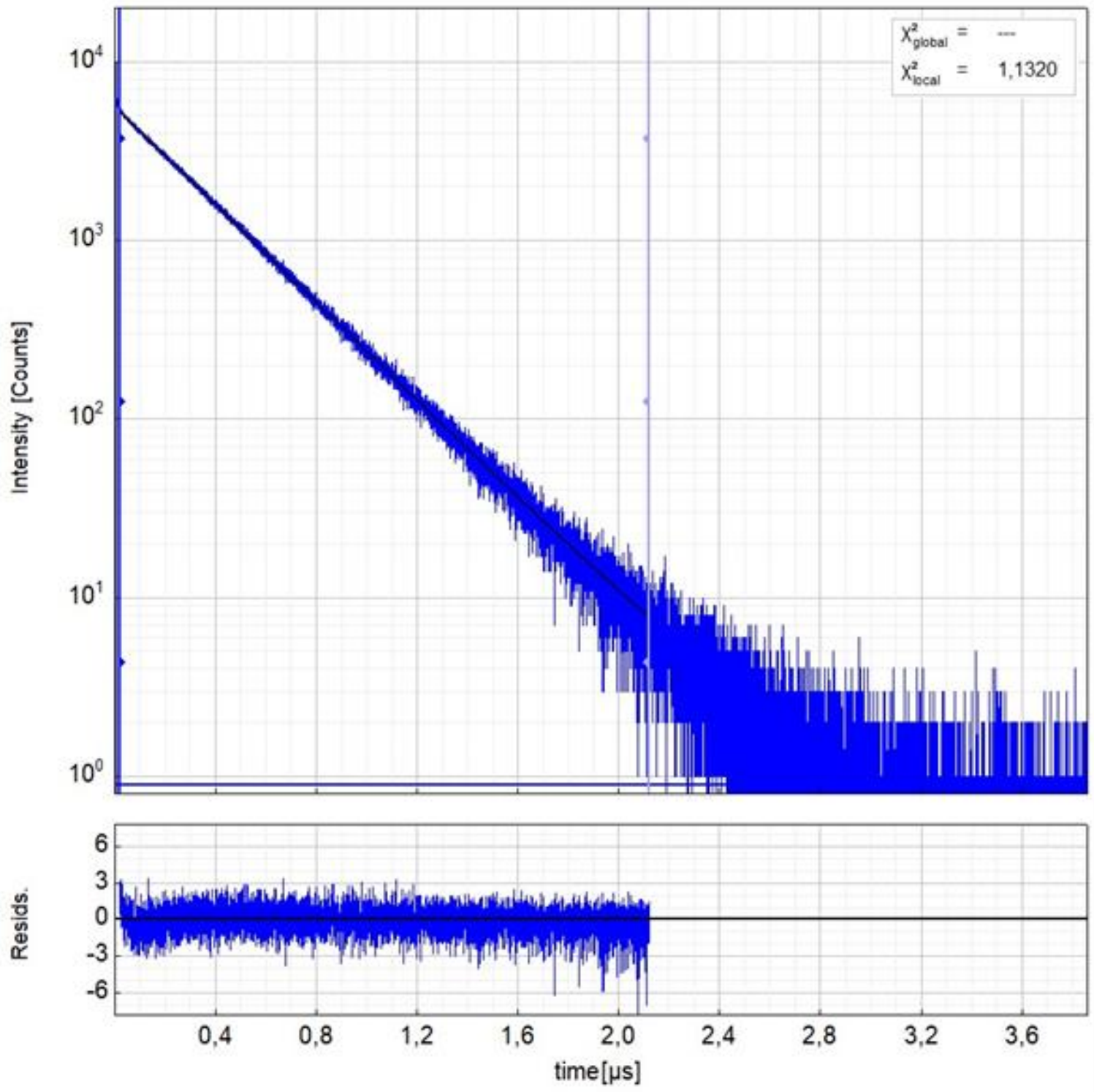

Parameter	Value	Δ	δ
A_1[kCnts/Chnl]	5,2437	±0,0059	0,1%
τ_1[ns]	316,49	±0,35	0,1%
I_1[kCnts]	6 482,7	±5,8	0,1%
A_{Rel1}[%]	100,0	---	---
I_{Rel1}[%]	100,0	---	---
$Bkgr_{Dec}$[kCnts]	0,0009	---	---
τ_{AvInt}[ns]	316,49	±0,35	0,1%
τ_{AvAmp}[ns]	316,49	±0,35	0,1%

Figure S-339. Left: Raw (experimental) time-resolved photoluminescence decay of **Pt(L_{tBu})NHC** in liquid DCM at 298 K (air-equilibrated), including the residuals (λ_{exc} = 376.7 nm, λ_{em} = 496 nm). Right: Fitting parameters including pre-exponential factors and confidence limits.

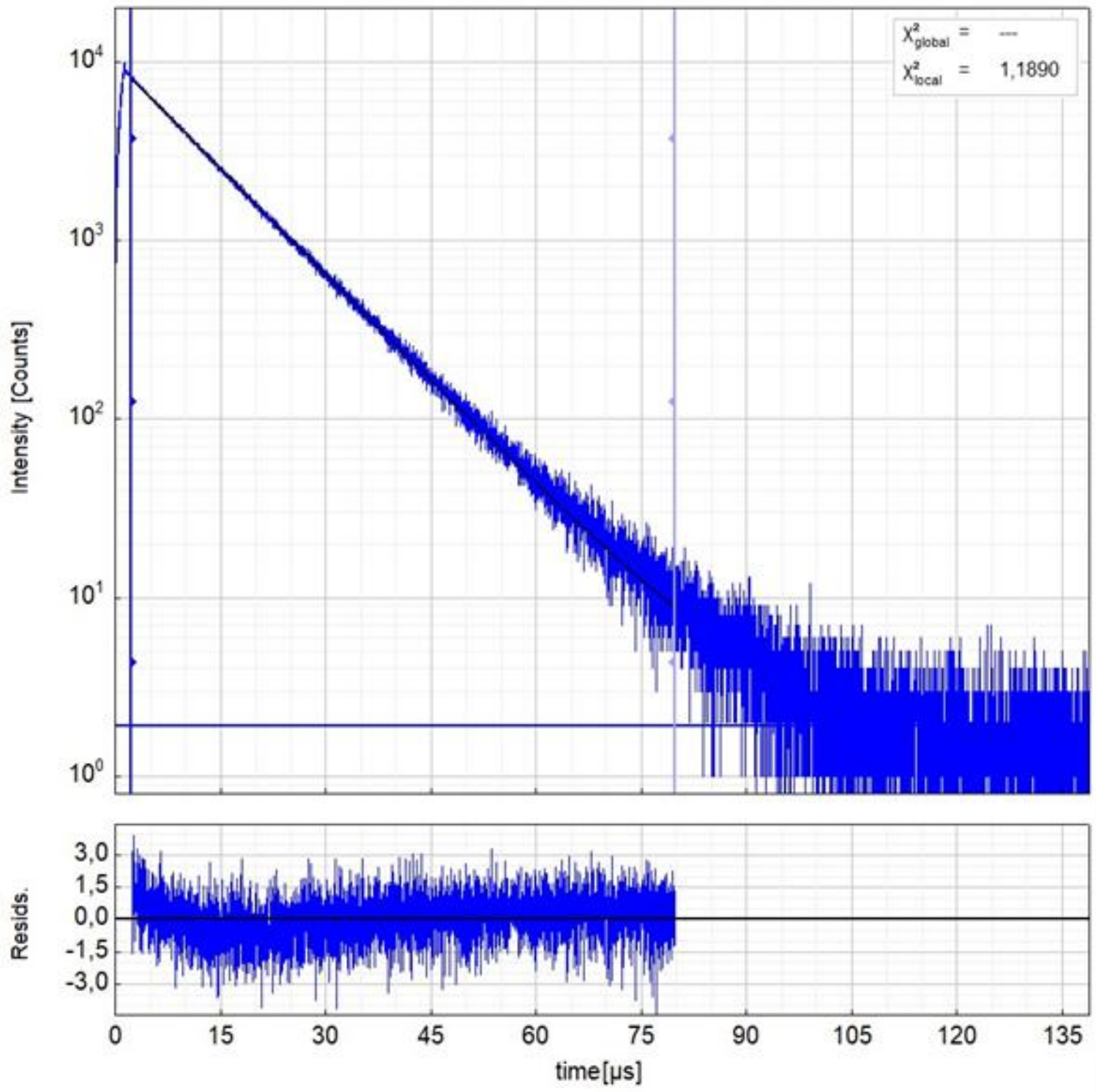

Parameter	Value	Δ	δ
A_1[kCnts/Chnl]	8,0076	±0,0089	0,1%
τ_1[ns]	10 983	±11	0,1%
I_1[kCnts]	5 496,6	±4,5	0,1%
A_{Rel1}[%]	100,0	---	---
I_{Rel1}[%]	100,0	---	---
$Bkgr_{Dec}$[kCnts]	0,0019	---	---
τ_{AvInt}[ns]	10 983	±11	0,1%
τ_{AvAmp}[ns]	10 983	±11	0,1%

Figure S-340. Left: Raw (experimental) time-resolved photoluminescence decay of **Pt(L_{tBu})NHC** in a frozen glassy matrix of DCM/MeOH (v/v = 1:1) at 77 K, including the residuals (λ_{exc} = 376.7 nm, λ_{em} = 486 nm). Right: Fitting parameters including pre-exponential factors and confidence limits.

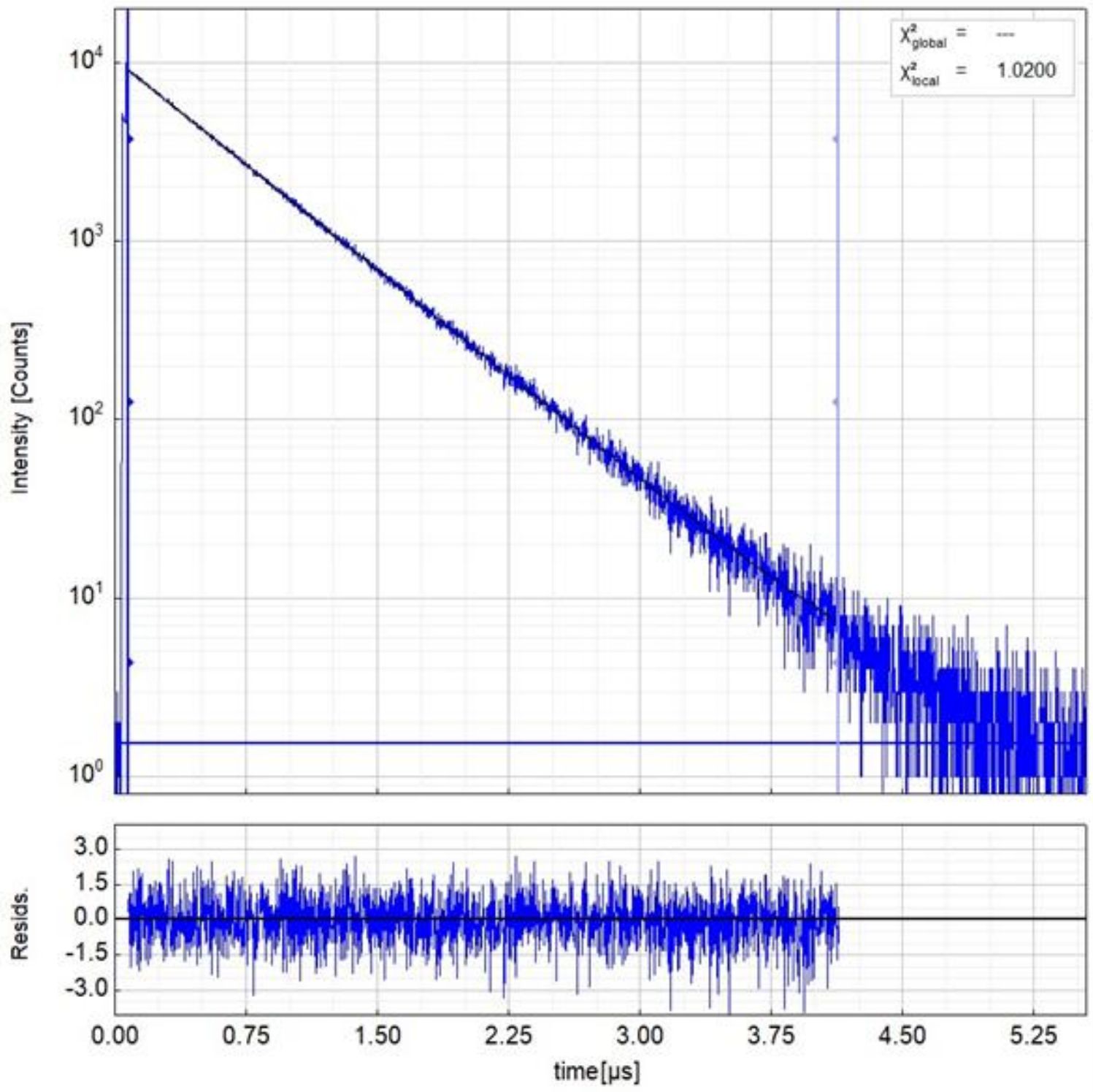

Parameter	Value	Δ	δ
A_1[kCnts/Chnl]	9.046	±0.017	0.2%
τ_1[ns]	551.20	±0.79	0.1%
I_1[kCnts]	2 493.1	±3.3	0.1%
A_{Rel1}[%]	100.0	---	---
I_{Rel1}[%]	100.0	---	---
$Bkgr_{Dec}$[kCnts]	0.0016	±0.0004	23%
τ_{AvInt}[ns]	551.20	±0.79	0.1%
τ_{AvAmp}[ns]	551.20	±0.79	0.1%

Figure S-341. Left: Raw (experimental) time-resolved photoluminescence decay of **Pt(L_{DiF})PPh$_3$** in liquid DCM at 298 K (Ar-purged), including the residuals (λ_{exc} = 376.7 nm, λ_{em} = 475 nm). Right: Fitting parameters including pre-exponential factors and confidence limits.

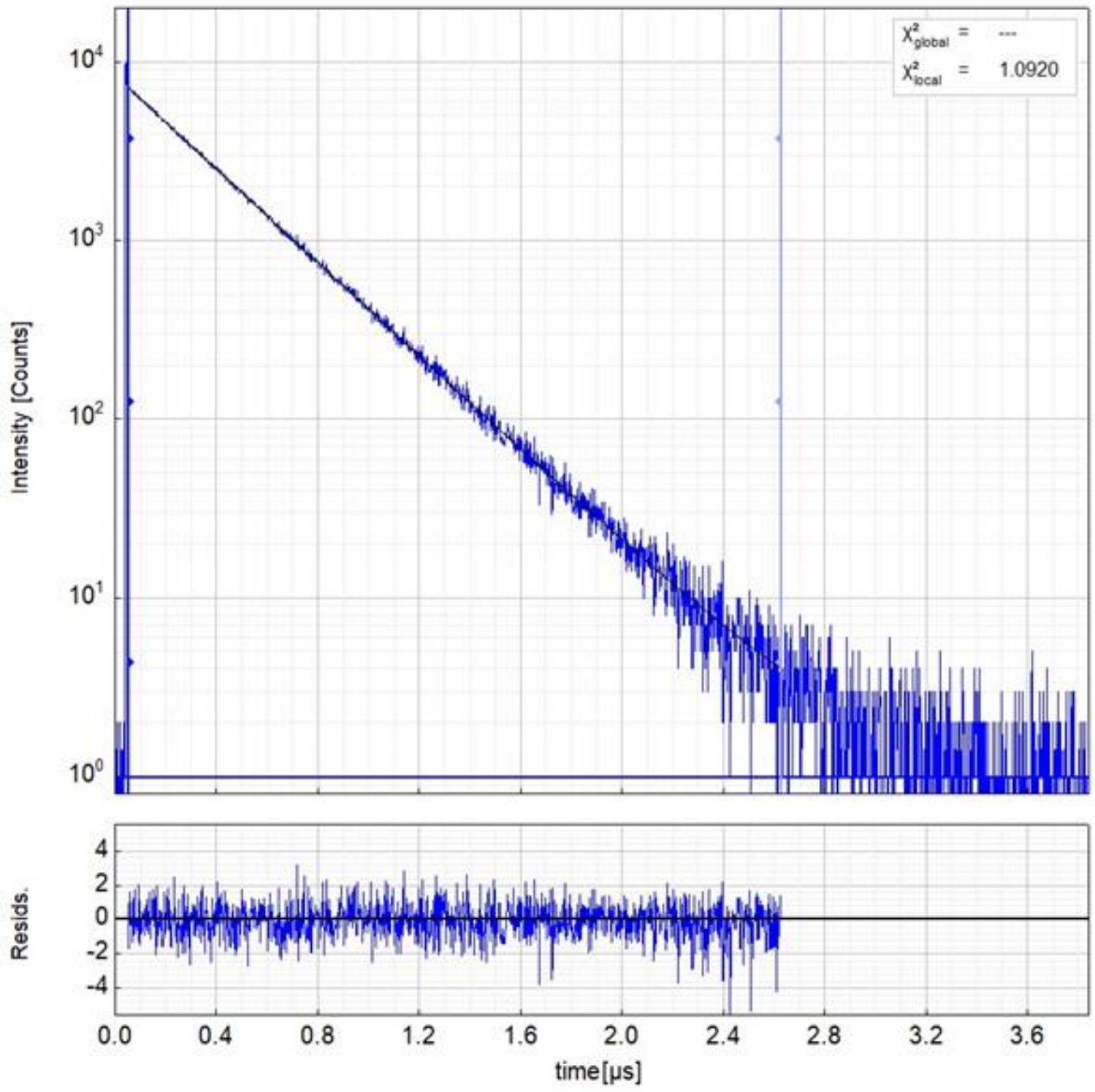

Parameter	Value	Δ	δ
A_1[kCnts/Chnl]	7.158	±0.019	0.3%
τ_1[ns]	331.71	±0.67	0.2%
I_1[kCnts]	1 187.1	±2.1	0.2%
A_{Rel1}[%]	100.0	---	---
I_{Rel1}[%]	100.0	---	---
$Bkgr_{Dec}$[kCnts]	0.0010	±0.0004	35%
τ_{AvInt}[ns]	331.71	±0.67	0.2%
τ_{AvAmp}[ns]	331.71	±0.67	0.2%

Figure S-342. Left: Raw (experimental) time-resolved photoluminescence decay of **Pt(L$_{DiF}$)PPh$_3$** in liquid DCM at 298 K (air-equilibrated), including the residuals (λ_{exc} = 376.7 nm, λ_{em} = 475 nm). Right: Fitting parameters including pre-exponential factors and confidence limits.

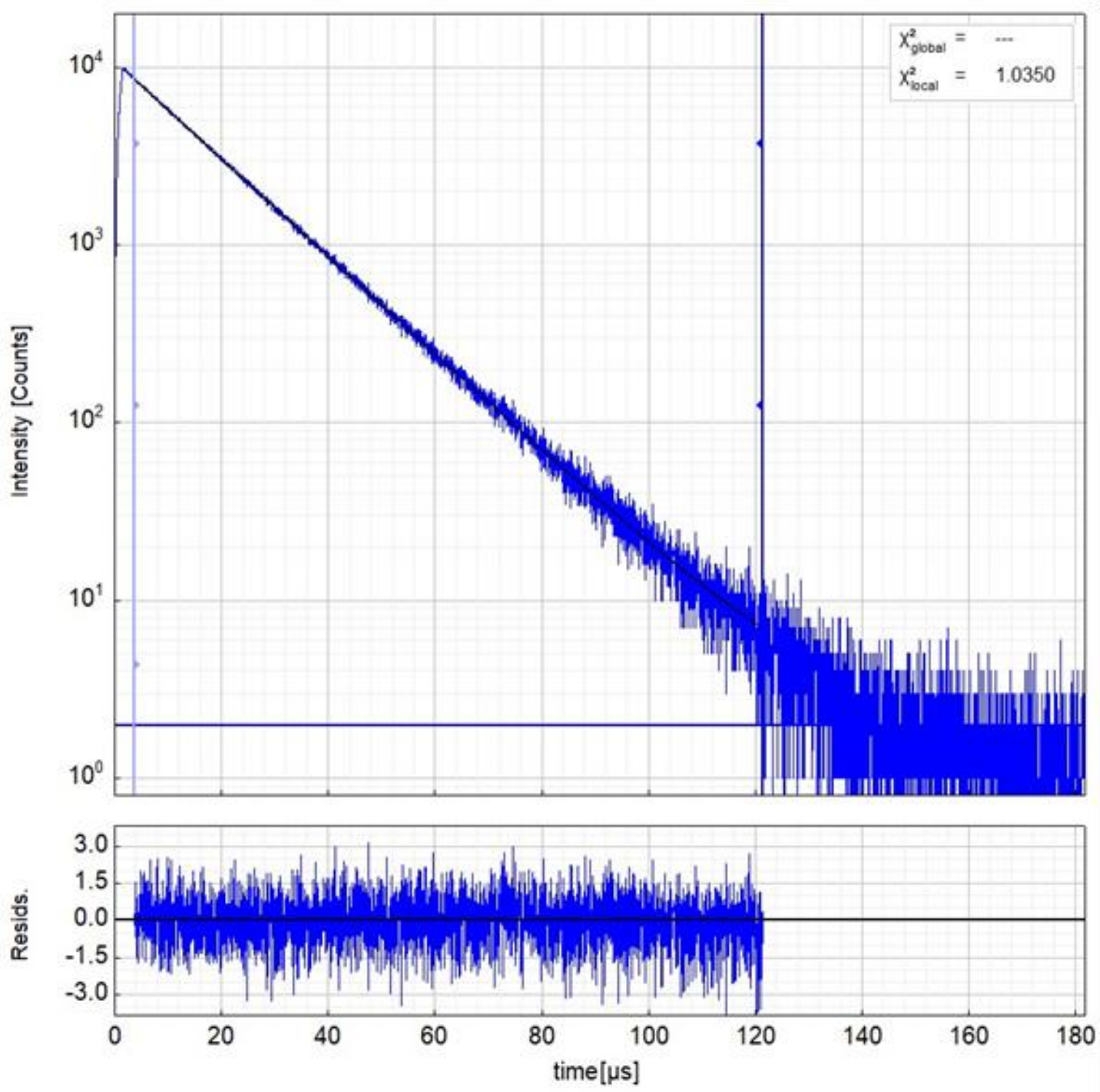

Parameter	Value	Δ	δ
A_1[kCnts/Chnl]	8.522	±0.013	0.1%
τ_1[ns]	15 810	±18	0.1%
I_1[kCnts]	4 210.0	±4.1	0.1%
A_{Rel1}[%]	100.0	---	---
I_{Rel1}[%]	100.0	---	---
$Bkgr_{Dec}$[kCnts]	0.0020	±0.0003	12%
τ_{AvInt}[ns]	15 810	±18	0.1%
τ_{AvAmp}[ns]	15 810	±18	0.1%

Figure S-343. Left: Raw (experimental) time-resolved photoluminescence decay of **Pt(L$_{DiF}$)PPh$_3$** in a frozen glassy matrix of DCM/MeOH (v/v = 1:1) at 77 K, including the residuals (λ_{exc} = 376.7 nm, λ_{em} = 470 nm). Right: Fitting parameters including pre-exponential factors and confidence limits.

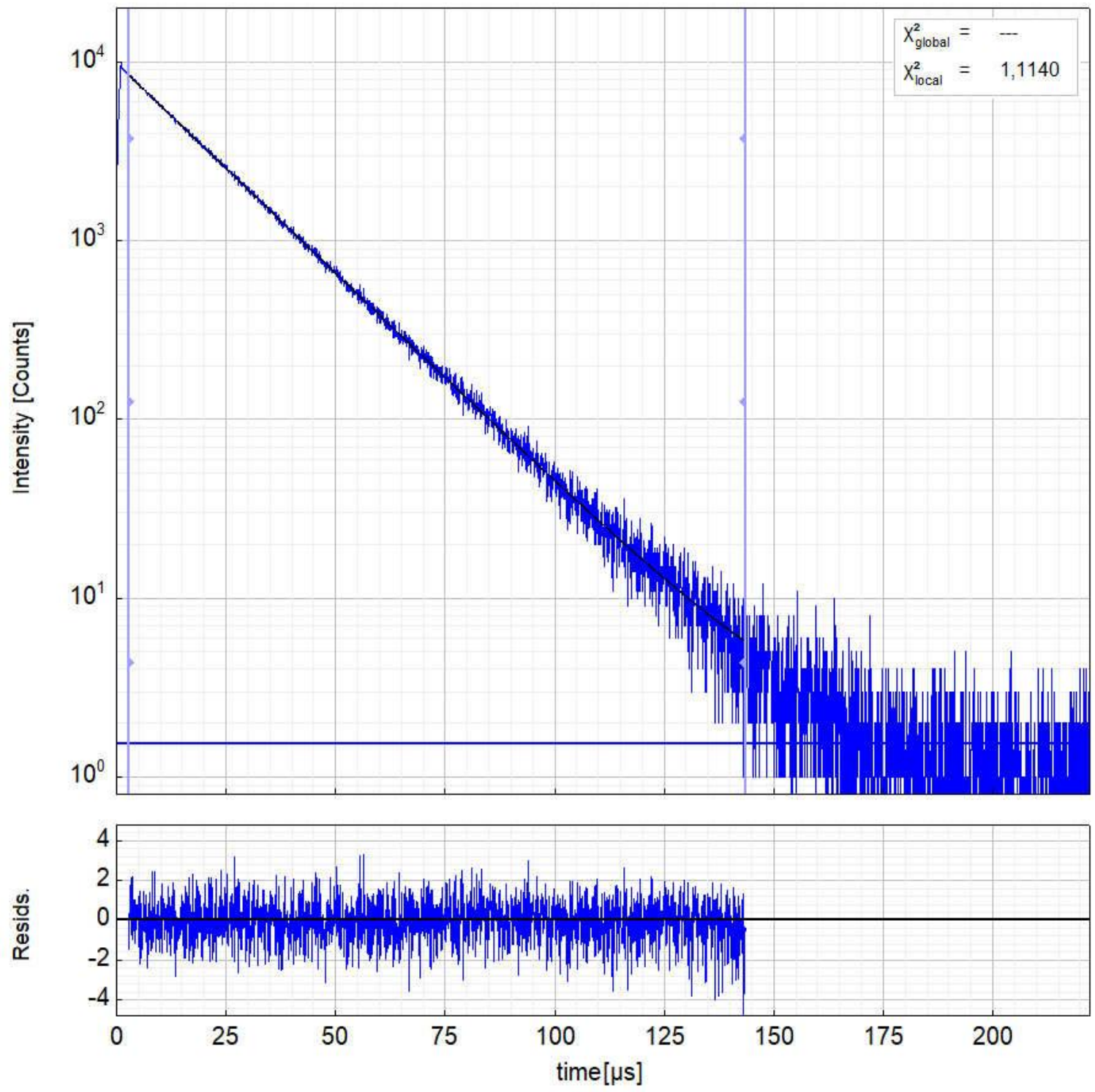

Parameter	Value	Δ	δ
A_1[kCnts/Chnl]	8,385	±0,015	0,2%
$τ_1$[ns]	18 494	±28	0,1%
I_1[kCnts]	2 423,0	±2,5	0,1%
$Bkgr_{Dec}$[kCnts]	0,0016	±0,0003	18%
$τ_{AvInt}$[ns]	18 494	±28	0,1%
$τ_{AvAmp}$[ns]	18 494	±28	0,1%

Figure S-344. Left: Raw (experimental) time-resolved photoluminescence decay of **Pt(L$_{mOMe}$)PPh$_3$** in liquid DCM at 298 K (Ar-purged), including the residuals ($λ_{exc}$ = 376.7 nm, $λ_{em}$ = 504 nm). Right: Fitting parameters including pre-exponential factors and confidence limits.

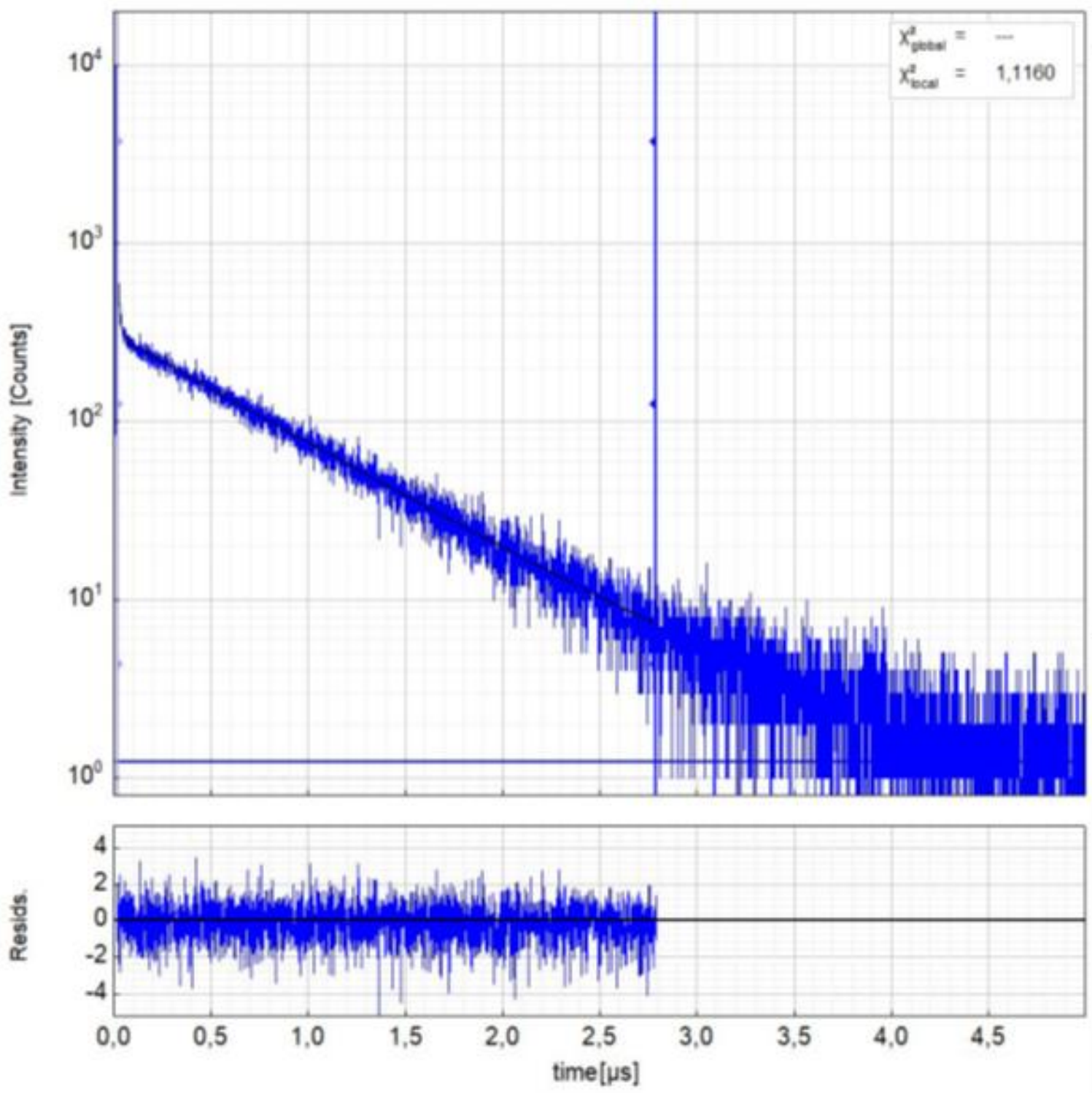

Parameter	Value	Δ	δ
A_1[kCnts/Chnl]	0,2981	±0,0012	0,4%
$τ_1$[ns]	713,2	±3,8	0,5%
I_1[kCnts]	212,57	±1,00	0,5%
A_{Rel1}[%]	36,7	±1,1	2,9%
I_{Rel1}[%]	97,7	±0,2	0,1%
A_2[kCnts/Chnl]	0,515	±0,023	4,3%
$τ_2$[ns]	10,01	±0,46	4,6%
I_2[kCnts]	5,15	±0,27	5,1%
A_{Rel2}[%]	63,4	±1,1	1,7%
I_{Rel2}[%]	2,4	±0,2	4,6%
$Bkgr_{Dec}$[kCnts]	0,0013	±0,0003	18%
$τ_{AvInt}$[ns]	696,6	±3,2	0,5%
$τ_{AvAmp}$[ns]	268,0	±7,6	2,8%

Figure S-345. Left: Raw (experimental) time-resolved photoluminescence decay of **Pt(L$_{mOMe}$)PPh$_3$** in liquid DCM at 298 K (air-equilibrated), including the residuals ($λ_{exc}$ = 376.7 nm, $λ_{em}$ = 500 nm). Right: Fitting parameters including pre-exponential factors and confidence limits.

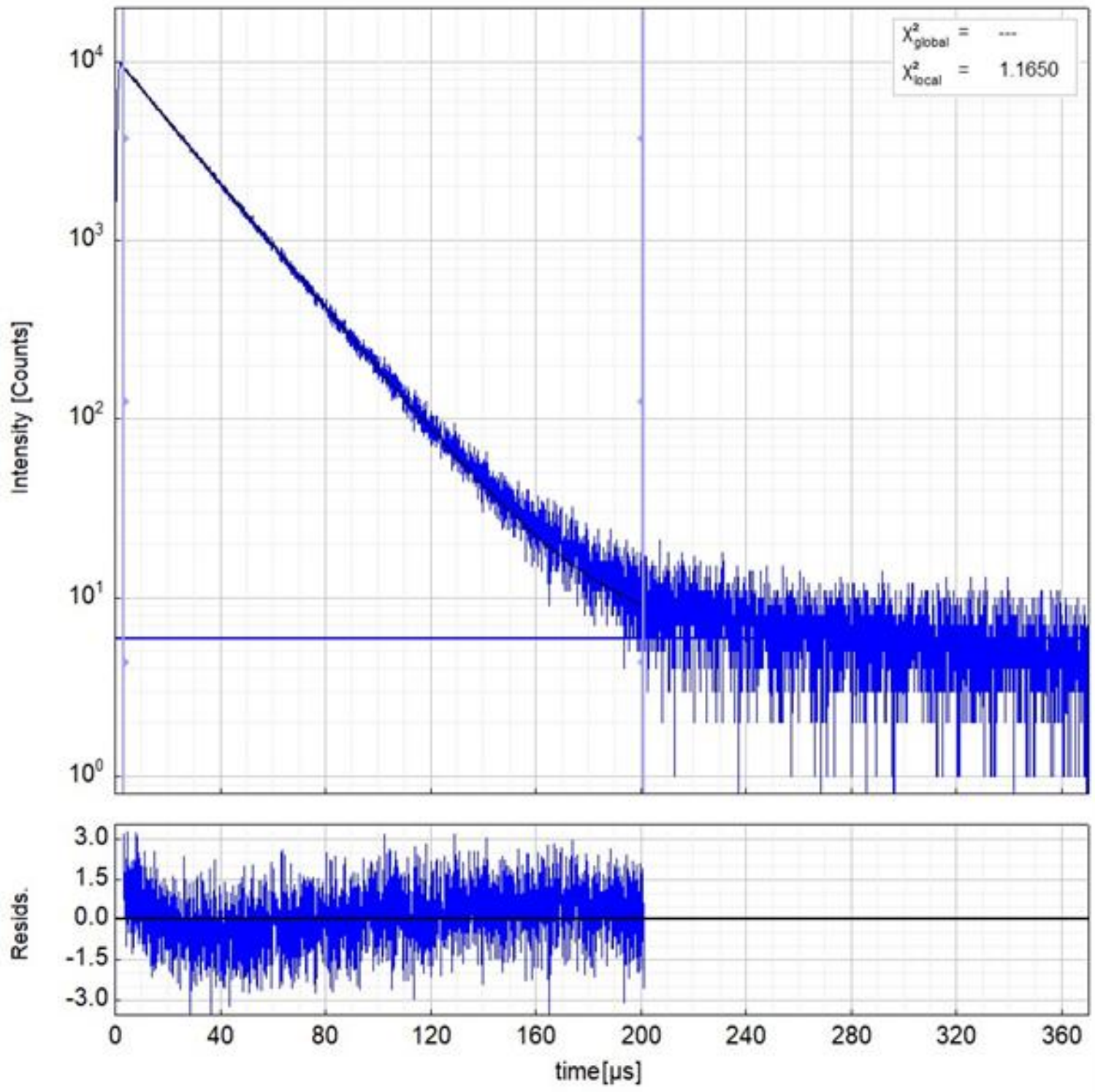

Parameter	Value	Δ	δ
A_1[kCnts/Chnl]	9.215	±0.017	0.2%
τ_1[ns]	24 787	±33	0.1%
I_1[kCnts]	3 568.9	±5.3	0.1%
A_{Rel1}[%]	100.0	---	---
I_{Rel1}[%]	100.0	---	---
$Bkgr_{Dec}$[kCnts]	0.0059	---	---
τ_{AvInt}[ns]	24 787	±33	0.1%
τ_{AvAmp}[ns]	24 787	±33	0.1%

Figure S-346. Left: Raw (experimental) time-resolved photoluminescence decay of **Pt(L$_{mOMe}$)PPh$_3$** in a frozen glassy matrix of DCM/MeOH (v/v = 1:1) at 77 K, including the residuals (λ_{exc} = 376.7 nm, λ_{em} = 500 nm). Right: Fitting parameters including pre-exponential factors and confidence limits.

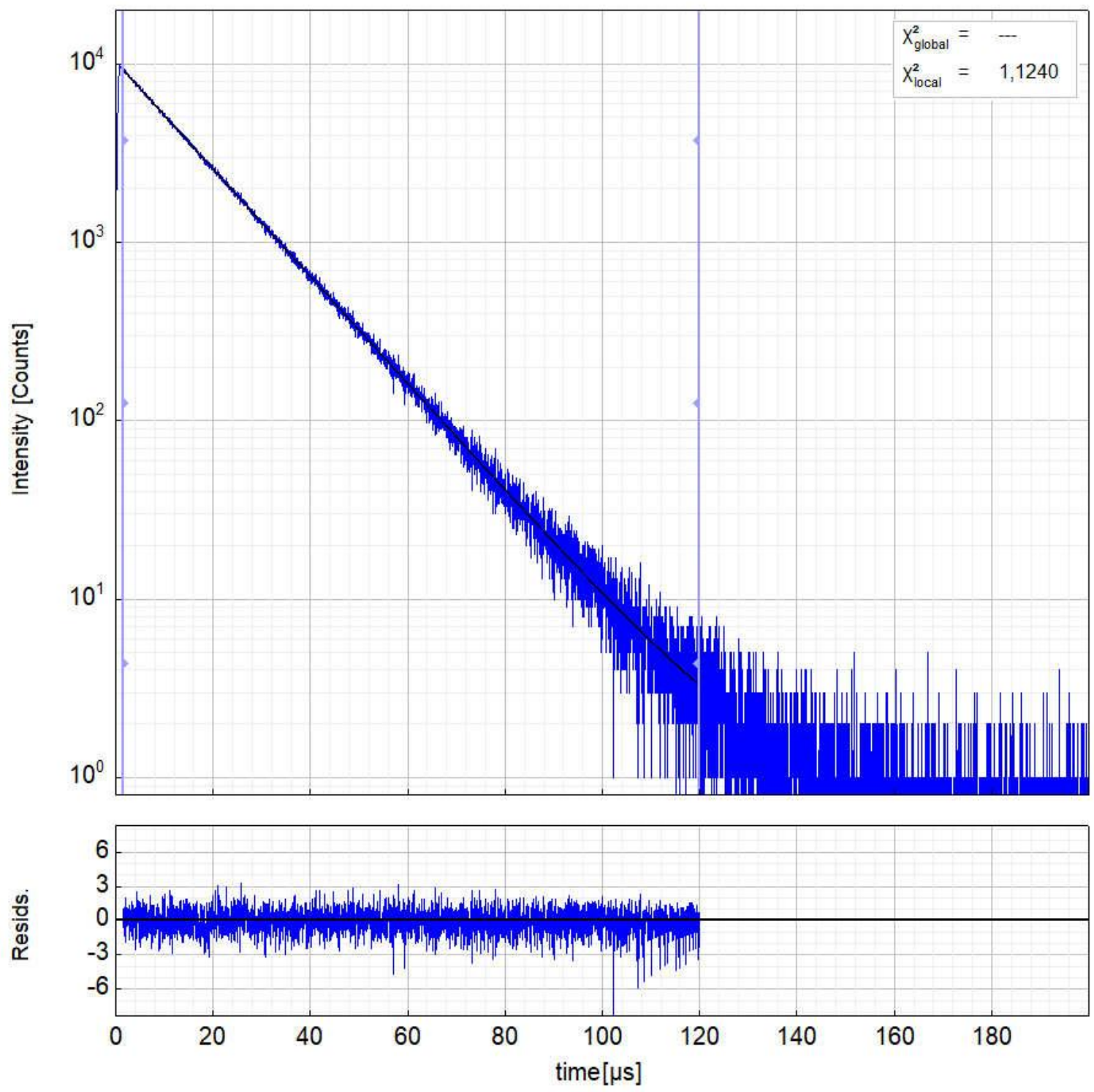

Parameter	Value	Δ	δ
A_1[kCnts/Chnl]	9,2910	±0,0081	0,1%
τ_1[ns]	14 431	±11	0,1%
I_1[kCnts]	4 189,7	±3,7	0,1%
$Bkgr_{Dec}$[kCnts]	0,0008	±0,0002	15%
τ_{AvInt}[ns]	14 431	±11	0,1%
τ_{AvAmp}[ns]	14 431	±11	0,1%

Figure S-347. Left: Raw (experimental) time-resolved photoluminescence decay of **Pt(L$_{mOMe}$)NHC** in liquid DCM at 298 K (Ar-purged), including the residuals (λ_{exc} = 376.7 nm, λ_{em} = 530 nm). Right: Fitting parameters including pre-exponential factors and confidence limits.

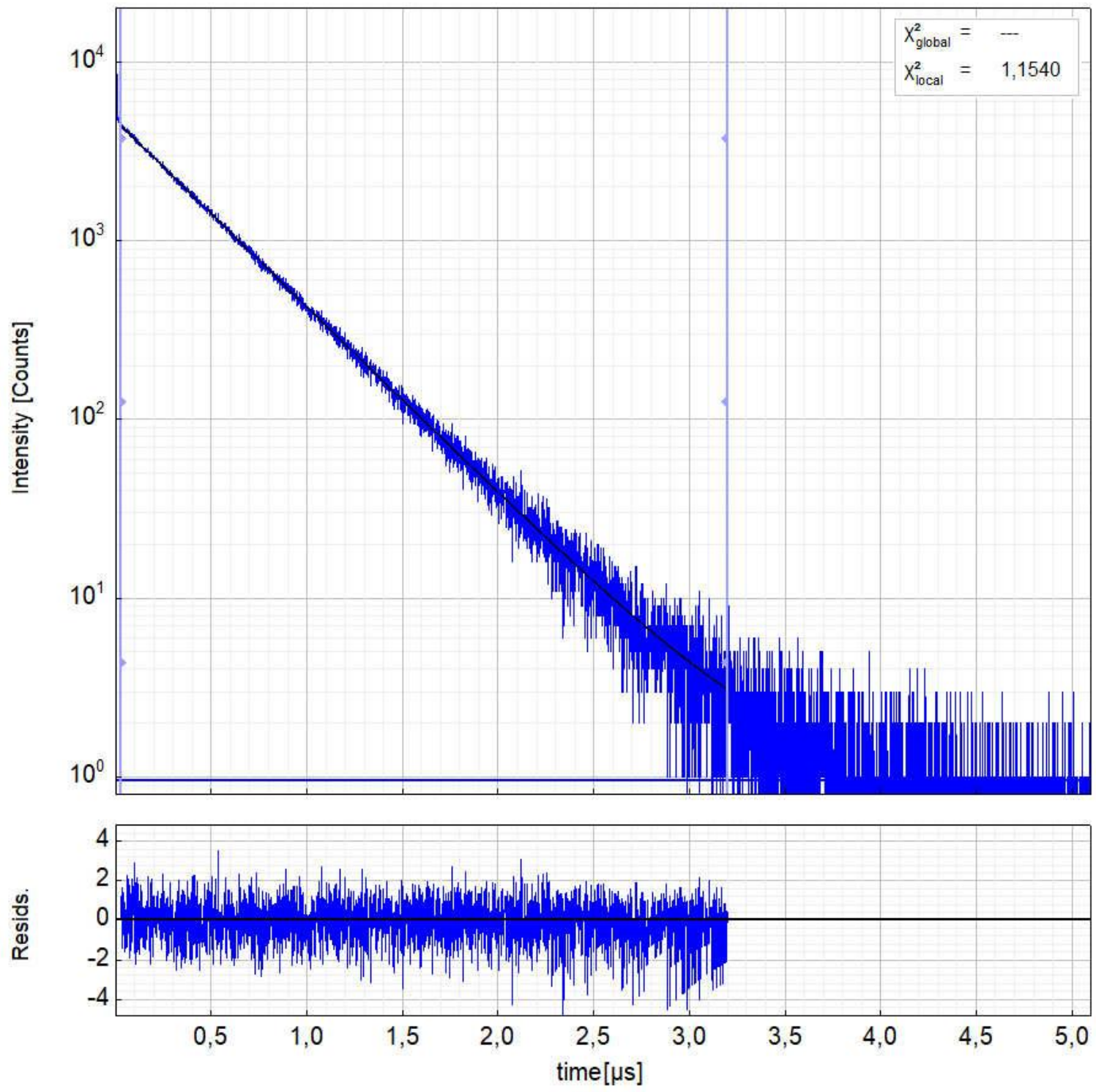

Parameter	Value	Δ	δ
A_1[kCnts/Chnl]	4,3866	±0,0072	0,2%
τ_1[ns]	415,57	±0,40	0,1%
I_1[kCnts]	1 822,9	±1,3	0,1%
$Bkgr_{Dec}$[kCnts]	0,0010	±0,0002	15%
τ_{AvInt}[ns]	415,57	±0,40	0,1%
τ_{AvAmp}[ns]	415,57	±0,40	0,1%

Figure S-348. Left: Raw (experimental) time-resolved photoluminescence decay of **Pt(L_{mOMe})NHC** in liquid DCM at 298 K (air-equilibrated), including the residuals (λ_{exc} = 376.7 nm, λ_{em} = 530 nm). Right: Fitting parameters including pre-exponential factors and confidence limits.

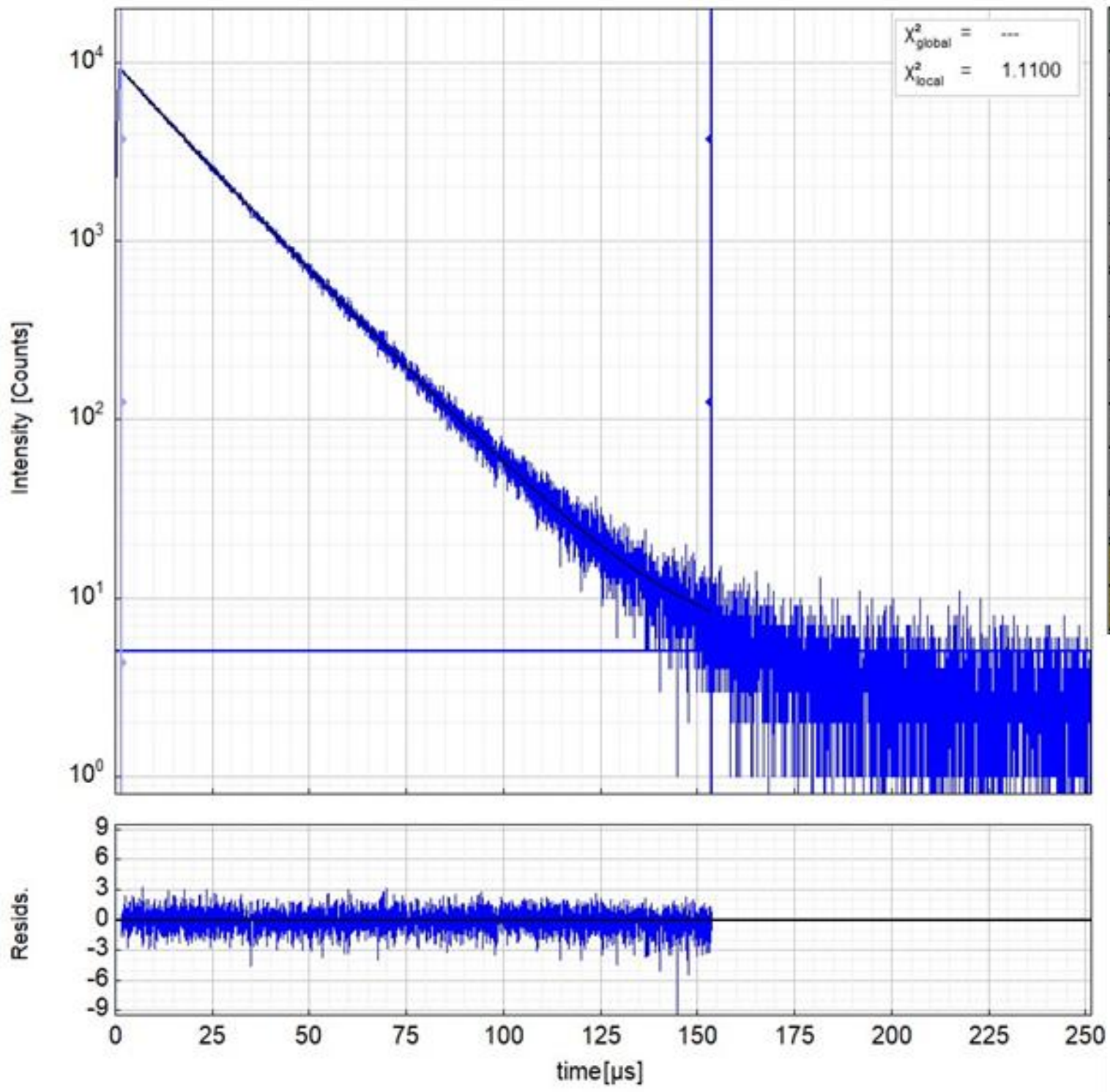

Parameter	Value	Δ	δ
A_1[kCnts/Chnl]	8.00	±0.11	1.3%
τ_1[ns]	19 634	±64	0.3%
I_1[kCnts]	4 903	±50	1.0%
A_{Rel1}[%]	87.9	±1.2	1.3%
I_{Rel1}[%]	92.9	±1.0	1.0%
A_2[kCnts/Chnl]	1.11	±0.11	9.4%
τ_2[ns]	10 900	±440	4.0%
I_2[kCnts]	378	±50	13%
A_{Rel2}[%]	12.2	±1.2	9.4%
I_{Rel2}[%]	7.2	±1.0	13%
$Bkgr_{Dec}$[kCnts]	0.0050	---	---
τ_{AvInt}[ns]	19 009	±15	0.1%
τ_{AvAmp}[ns]	18 569	±27	0.1%

Figure S-349. Left: Raw (experimental) time-resolved photoluminescence decay of **Pt(L_{mOMe})NHC** in a frozen glassy matrix of DCM/MeOH (v/v = 1:1) at 77 K, including the residuals (λ_{exc} = 376.7 nm, λ_{em} = 480 nm). Right: Fitting parameters including pre-exponential factors and confidence limits.

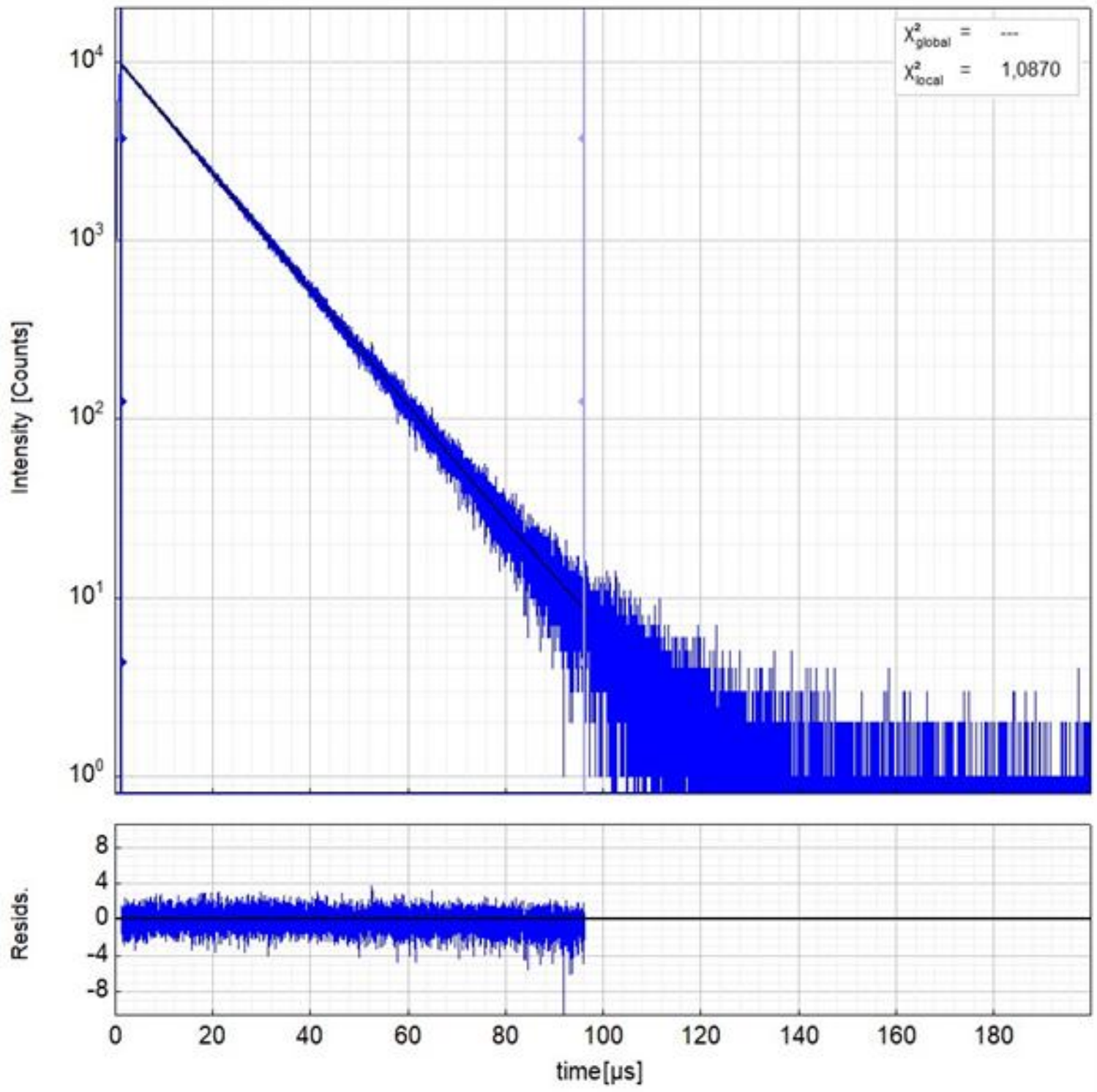

Parameter	Value	Δ	δ
A_1[kCnts/Chnl]	9,6136	±0,0092	0,1%
$τ_1$[ns]	13 338,9	±9,1	0,1%
I_1[kCnts]	16 030	±11	0,1%
A_{Rel1}[%]	100,0	---	---
I_{Rel1}[%]	100,0	---	---
$Bkgr_{Dec}$[kCnts]	0,0008	---	---
$τ_{AvInt}$[ns]	13 338,9	±9,1	0,1%
$τ_{AvAmp}$[ns]	13 338,9	±9,1	0,1%

Figure S-350. Left: Raw (experimental) time-resolved photoluminescence decay of **Pt(L_{pOMe})PPh_3** in liquid DCM at 298 K (Ar-purged), including the residuals ($λ_{exc}$ = 376.7 nm, $λ_{em}$ = 580 nm). Right: Fitting parameters including pre-exponential factors and confidence limits.

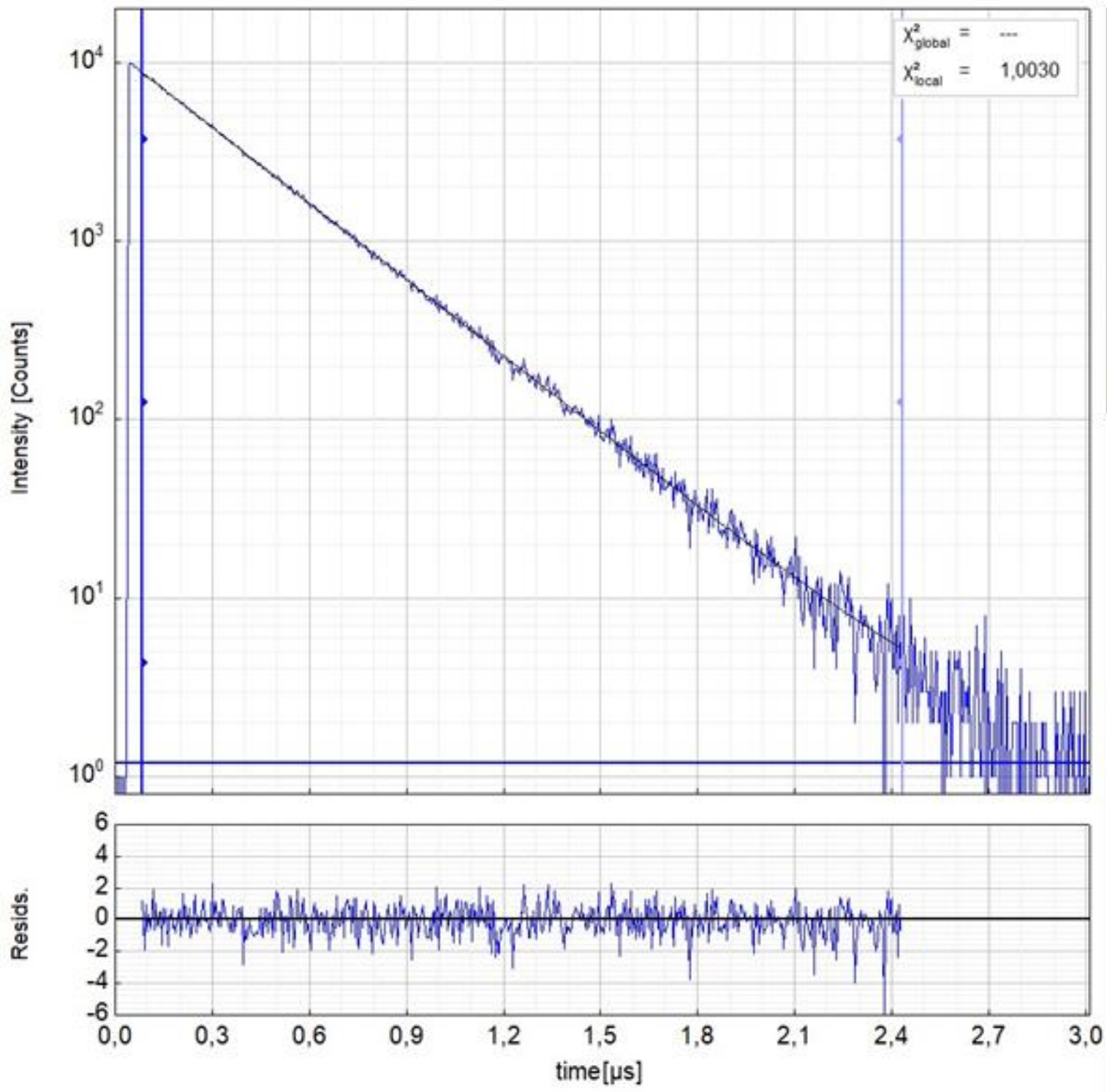

Parameter	Value	Δ	δ
A_1[kCnts/Chnl]	8,774	±0,028	0,3%
$τ_1$[ns]	305,21	±0,80	0,3%
I_1[kCnts]	669,5	±1,5	0,2%
A_{Rel1}[%]	100,0	---	---
I_{Rel1}[%]	100,0	---	---
$Bkgr_{Dec}$[kCnts]	0,0012	±0,0006	45%
$τ_{AvInt}$[ns]	305,21	±0,80	0,3%
$τ_{AvAmp}$[ns]	305,21	±0,80	0,3%

Figure S-351. Left: Raw (experimental) time-resolved photoluminescence decay of **Pt(L_{pOMe})PPh_3** in liquid DCM at 298 K (air-equilibrated), including the residuals ($λ_{exc}$ = 376.7 nm, $λ_{em}$ = 580 nm). Right: Fitting parameters including pre-exponential factors and confidence limits.

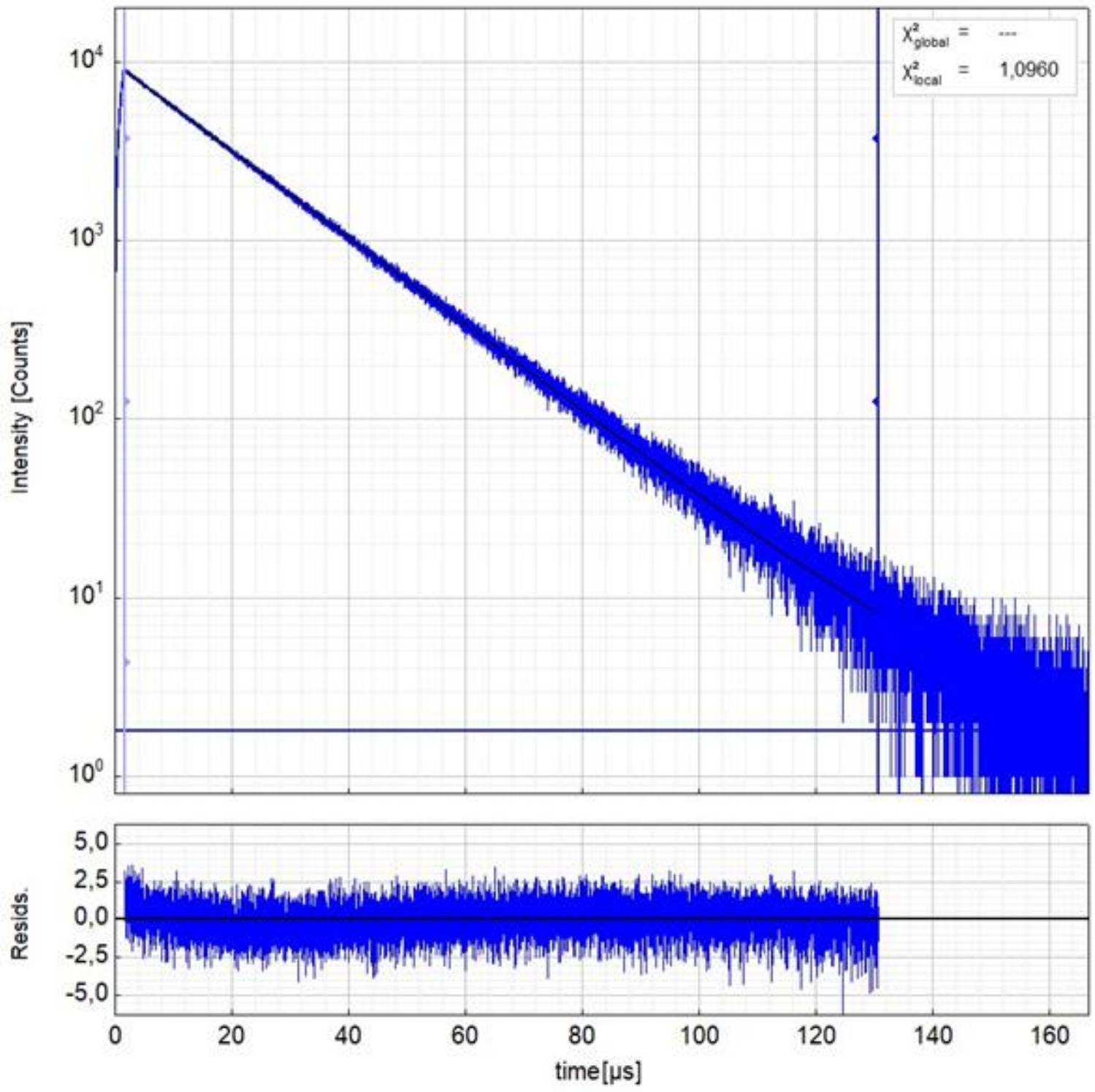

Parameter	Value	Δ	δ
A_1[kCnts/Chnl]	8,8353	±0,0082	0,1%
τ_1[ns]	17 840	±12	0,1%
I_1[kCnts]	19 703	±13	0,1%
A_{Rel1}[%]	100,0	---	---
I_{Rel1}[%]	100,0	---	---
$Bkgr_{Dec}$[kCnts]	0,0018	---	---
τ_{AvInt}[ns]	17 840	±12	0,1%
τ_{AvAmp}[ns]	17 840	±12	0,1%

Figure S-352. Left: Raw (experimental) time-resolved photoluminescence decay of **Pt(L_{pOMe})PPh_3** in a frozen glassy matrix of DCM/MeOH (v/v = 1:1) at 77 K, including the residuals (λ_{exc} = 376.7 nm, λ_{em} = 535 nm). Right: Fitting parameters including pre-exponential factors and confidence limits.

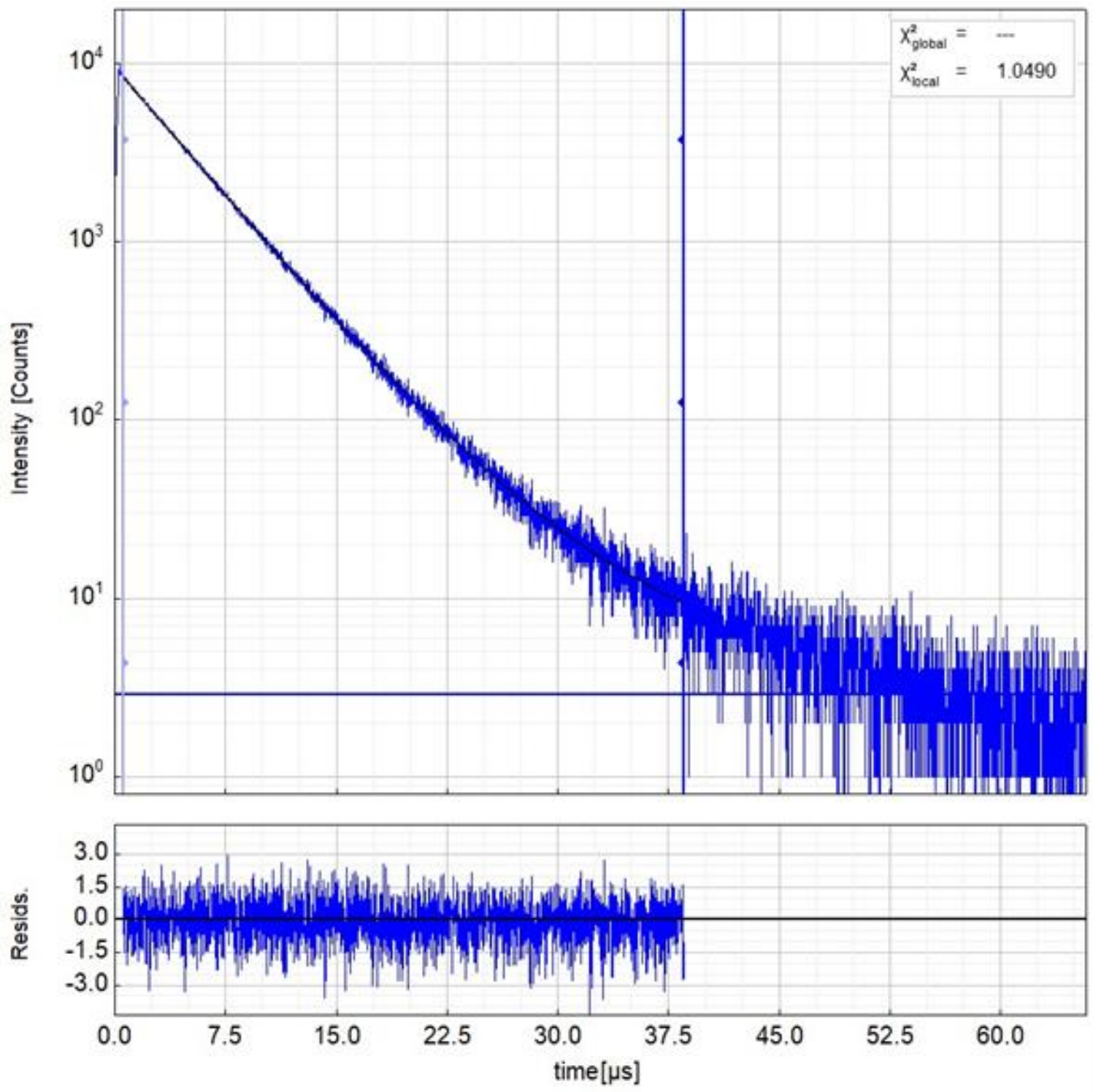

Parameter	Value	Δ	δ
A_1[kCnts/Chnl]	0.198	±0.066	33%
τ_1[ns]	10 400	±1 100	10.0%
I_1[kCnts]	128	±29	22%
A_{Rel1}[%]	2.4	±0.8	33%
I_{Rel1}[%]	5.4	±1.2	22%
A_2[kCnts/Chnl]	8.188	±0.058	0.7%
τ_2[ns]	4 428	±30	0.7%
I_2[kCnts]	2 266	±30	1.3%
A_{Rel2}[%]	97.7	±0.8	0.8%
I_{Rel2}[%]	94.7	±1.2	1.3%
$Bkgr_{Dec}$[kCnts]	0.0029	---	---
τ_{AvInt}[ns]	4 741	±14	0.3%
τ_{AvAmp}[ns]	4 566.3	±9.6	0.2%

Figure S-353. Left: Raw (experimental) time-resolved photoluminescence decay of **Pt(L_{mDMA})PPh_3** in liquid DCM at 298 K (Ar-purged), including the residuals (λ_{exc} = 376.7 nm, λ_{em} = 570 nm). Right: Fitting parameters including pre-exponential factors and confidence limits.

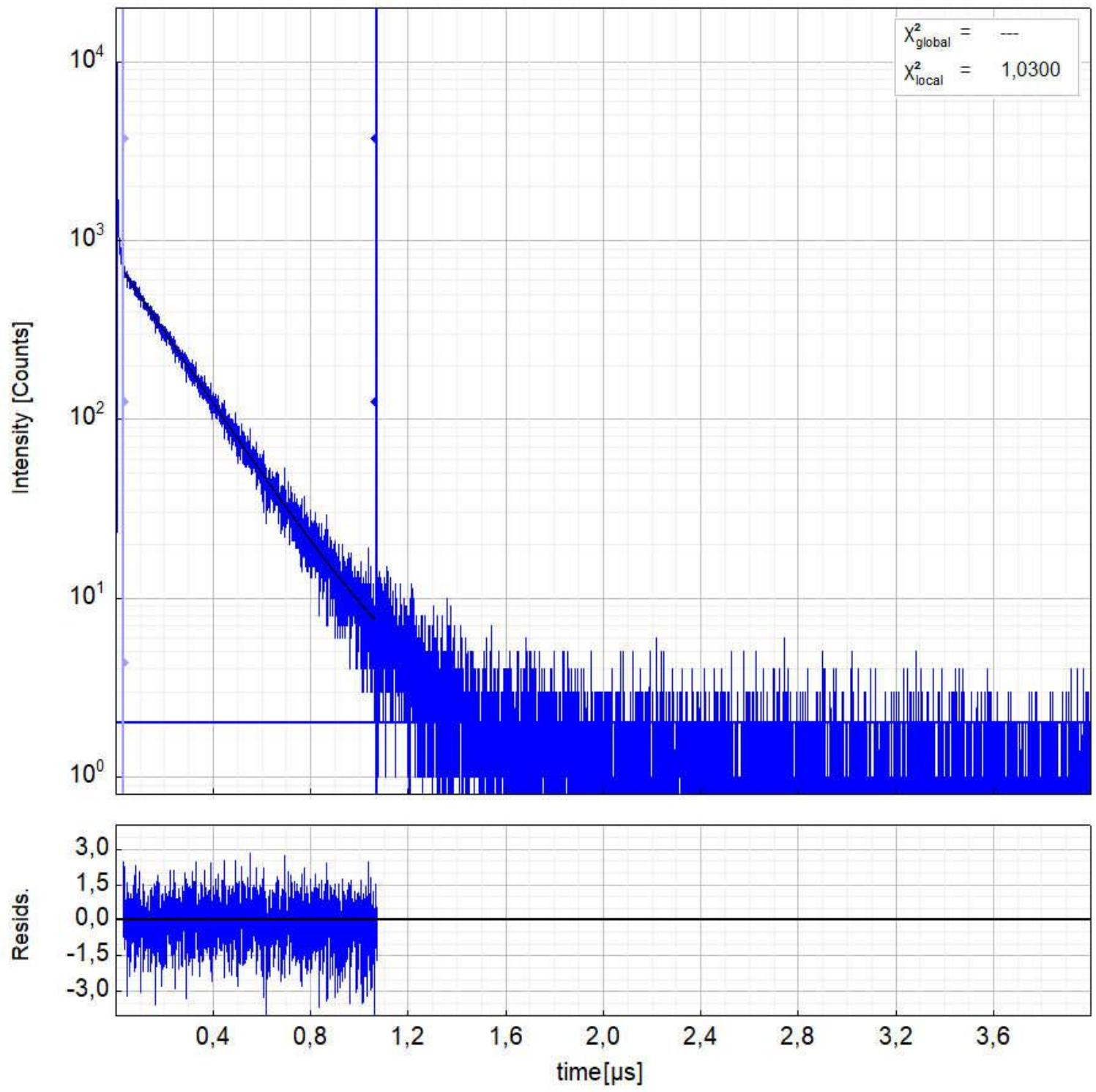

Parameter	Value	Δ	δ
A_1[kCnts/Chnl]	0,6706	±0,0026	0,4%
τ_1[ns]	215,6	±1,3	0,6%
I_1[kCnts]	289,1	±1,3	0,4%
A_{Rel1}[%]	100,0	---	---
I_{Rel1}[%]	100,0	---	---
$Bkgr_{Dec}$[kCnts]	0,0021	±0,0003	15%
τ_{AvInt}[ns]	215,6	±1,3	0,6%
τ_{AvAmp}[ns]	215,6	±1,3	0,6%

Figure S-354. Left: Raw (experimental) time-resolved photoluminescence decay of **Pt(L$_{mDMA}$)PPh$_3$** in liquid DCM at 298 K (air-equilibrated), including the residuals (λ_{exc} = 376.7 nm, λ_{em} = 570 nm). Right: Fitting parameters including pre-exponential factors and confidence limits.

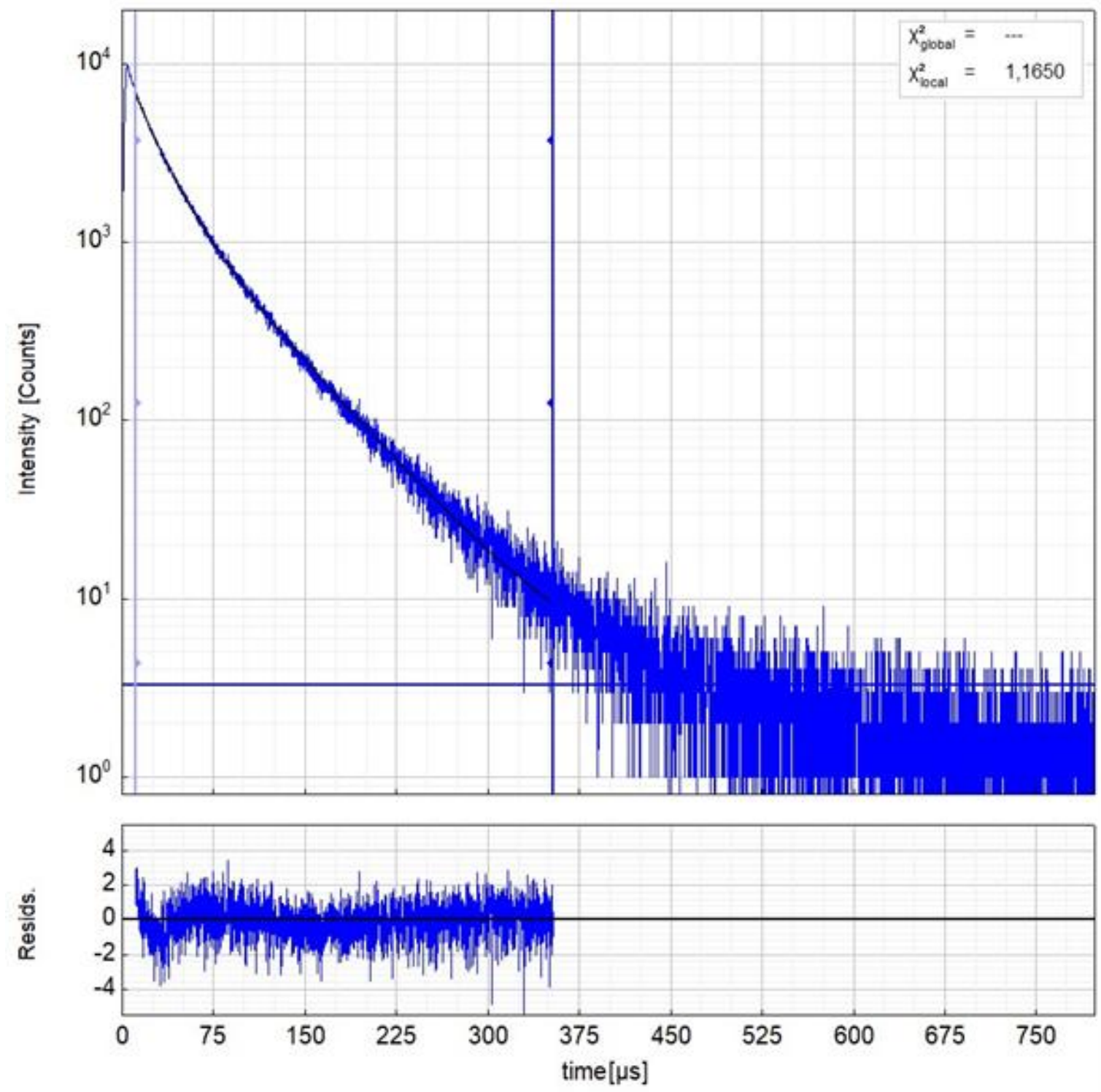

Parameter	Value	Δ	δ
A_1[kCnts/Chnl]	2,281	±0,062	2,7%
τ_1[ns]	57 800	±520	0,9%
I_1[kCnts]	1 030	±19	1,8%
A_{Rel1}[%]	34,6	±0,9	2,4%
I_{Rel1}[%]	59,1	±1,1	1,9%
A_2[kCnts/Chnl]	4,325	±0,053	1,2%
τ_2[ns]	21 150	±370	1,7%
I_2[kCnts]	715	±19	2,7%
A_{Rel2}[%]	65,5	±0,9	1,3%
I_{Rel2}[%]	41,0	±1,1	2,7%
$Bkgr_{Dec}$[kCnts]	0,0033	---	---
τ_{AvInt}[ns]	42 781	±93	0,2%
τ_{AvAmp}[ns]	33 800	±160	0,5%

Figure S-355. Left: Raw (experimental) time-resolved photoluminescence decay of **Pt(L$_{mDMA}$)PPh$_3$** in a frozen glassy matrix of DCM/MeOH (v/v = 1:1) at 77 K, including the residuals (λ_{exc} = 376.7 nm, λ_{em} = 535 nm). Right: Fitting parameters including pre-exponential factors and confidence limits.

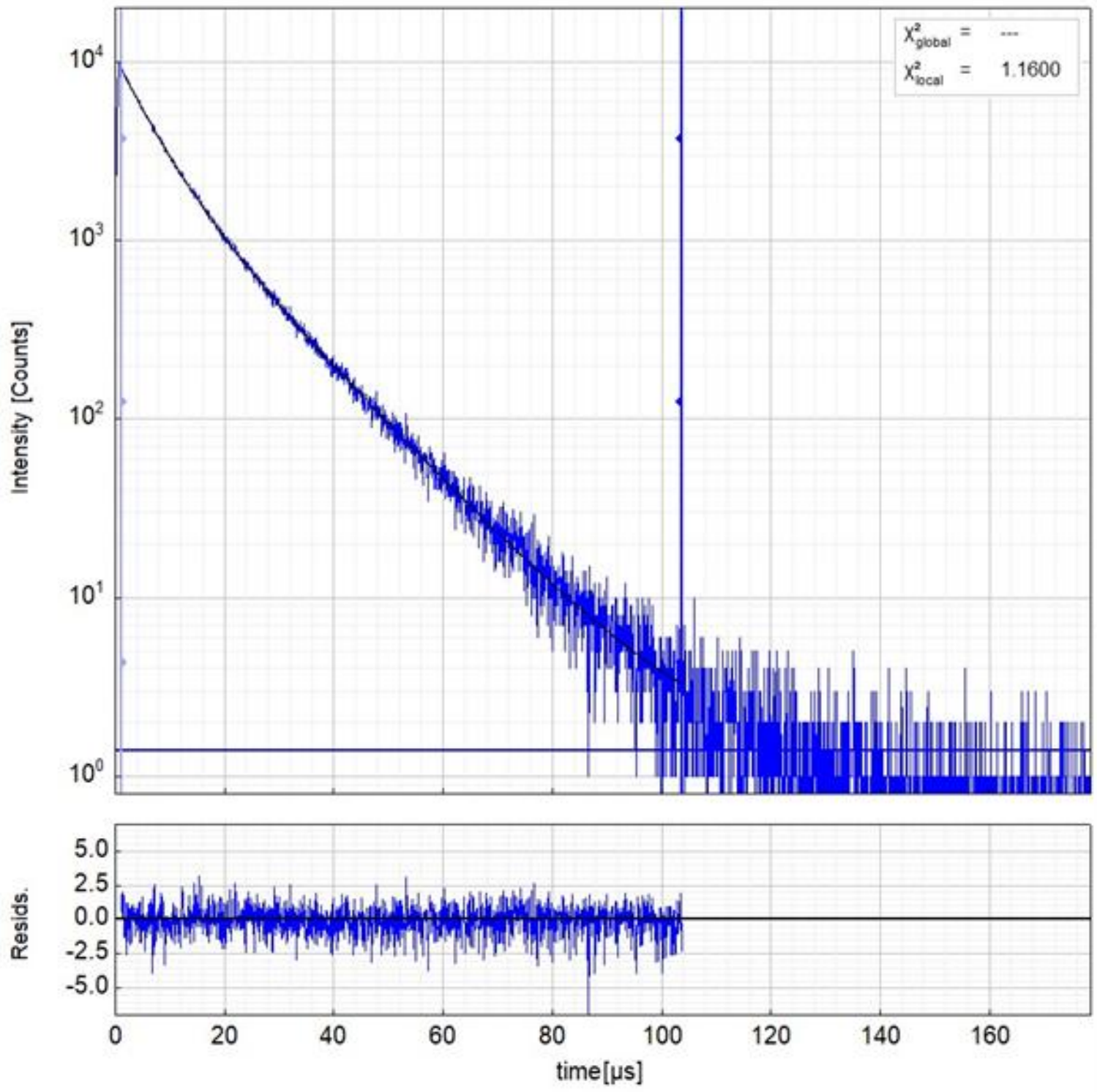

Parameter	Value	Δ	δ
A_1[kCnts/Chnl]	3.21	±0.11	3.4%
$τ_1$[ns]	13 820	±140	1.0%
I_1[kCnts]	693	±18	2.5%
A_{Rel1}[%]	34.9	±1.2	3.3%
I_{Rel1}[%]	56.1	±1.4	2.5%
A_2[kCnts/Chnl]	6.008	±0.099	1.6%
$τ_2$[ns]	5 800	±110	1.7%
I_2[kCnts]	544	±17	3.1%
A_{Rel2}[%]	65.2	±1.2	1.7%
I_{Rel2}[%]	44.0	±1.4	3.1%
$Bkgr_{Dec}$[kCnts]	0.0014	---	---
$τ_{AvInt}$[ns]	10 287	±23	0.2%
$τ_{AvAmp}$[ns]	8 586	±35	0.4%

Figure S-356. Left: Raw (experimental) time-resolved photoluminescence decay of **Pt(L$_{mDMA}$)NHC** in liquid DCM at 298 K (Ar-purged), including the residuals ($λ_{exc}$ = 376.7 nm, $λ_{em}$ = 502 nm). Right: Fitting parameters including pre-exponential factors and confidence limits.

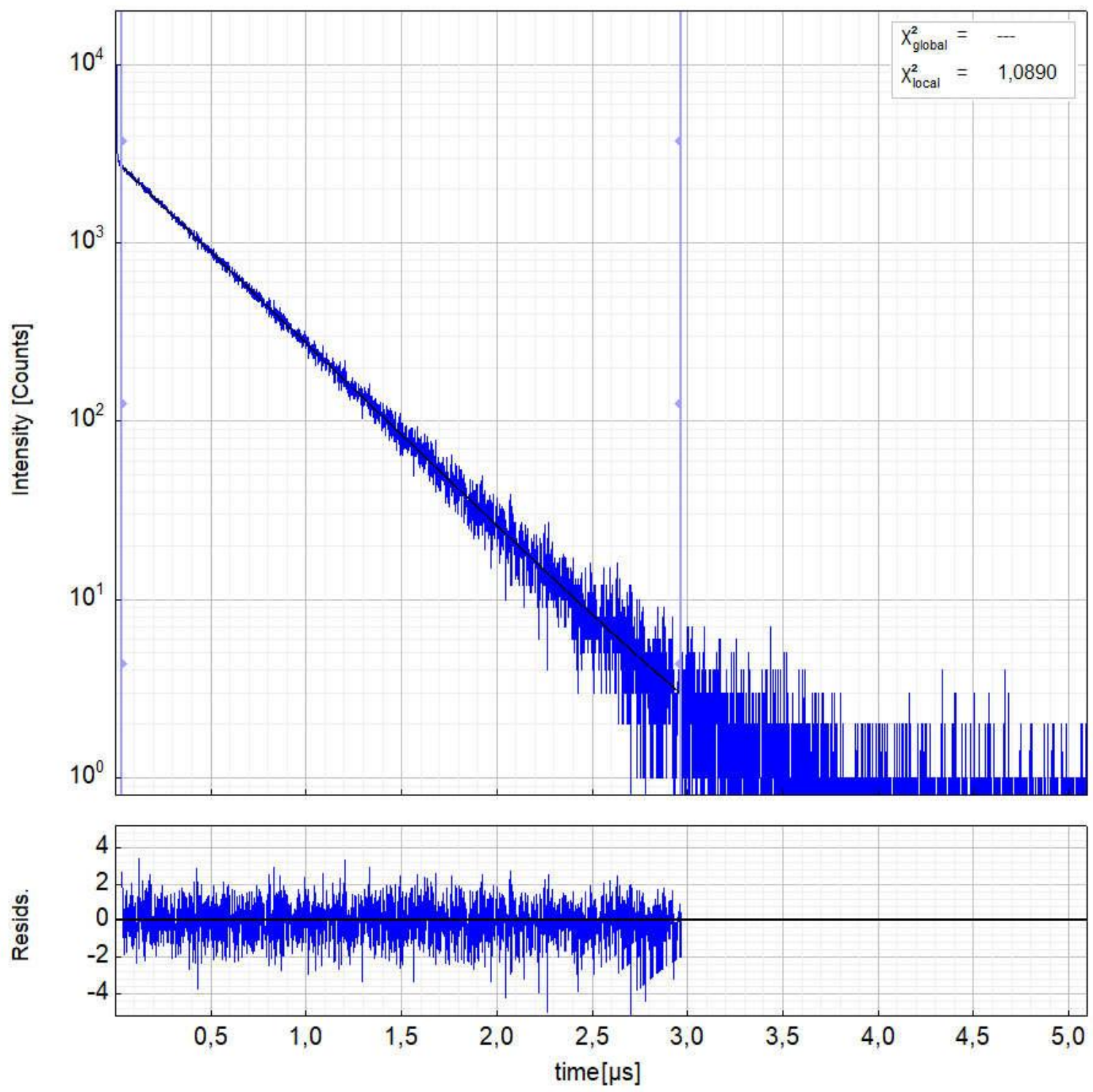

Parameter	Value	Δ	δ
A_1[kCnts/Chnl]	2,6649	±0,0061	0,2%
$τ_1$[ns]	423,1	±1,1	0,2%
I_1[kCnts]	1 127,5	±3,5	0,3%
$Bkgr_{Dec}$[kCnts]	0,0004	±0,0003	59%
$τ_{AvInt}$[ns]	423,1	±1,1	0,2%
$τ_{AvAmp}$[ns]	423,1	±1,1	0,2%

Figure S-357. Left: Raw (experimental) time-resolved photoluminescence decay of **Pt(L$_{mDMA}$)NHC** in liquid DCM at 298 K (air-equilibrated), including the residuals ($λ_{exc}$ = 376.7 nm, $λ_{em}$ = 502 nm). Right: Fitting parameters including pre-exponential factors and confidence limits.

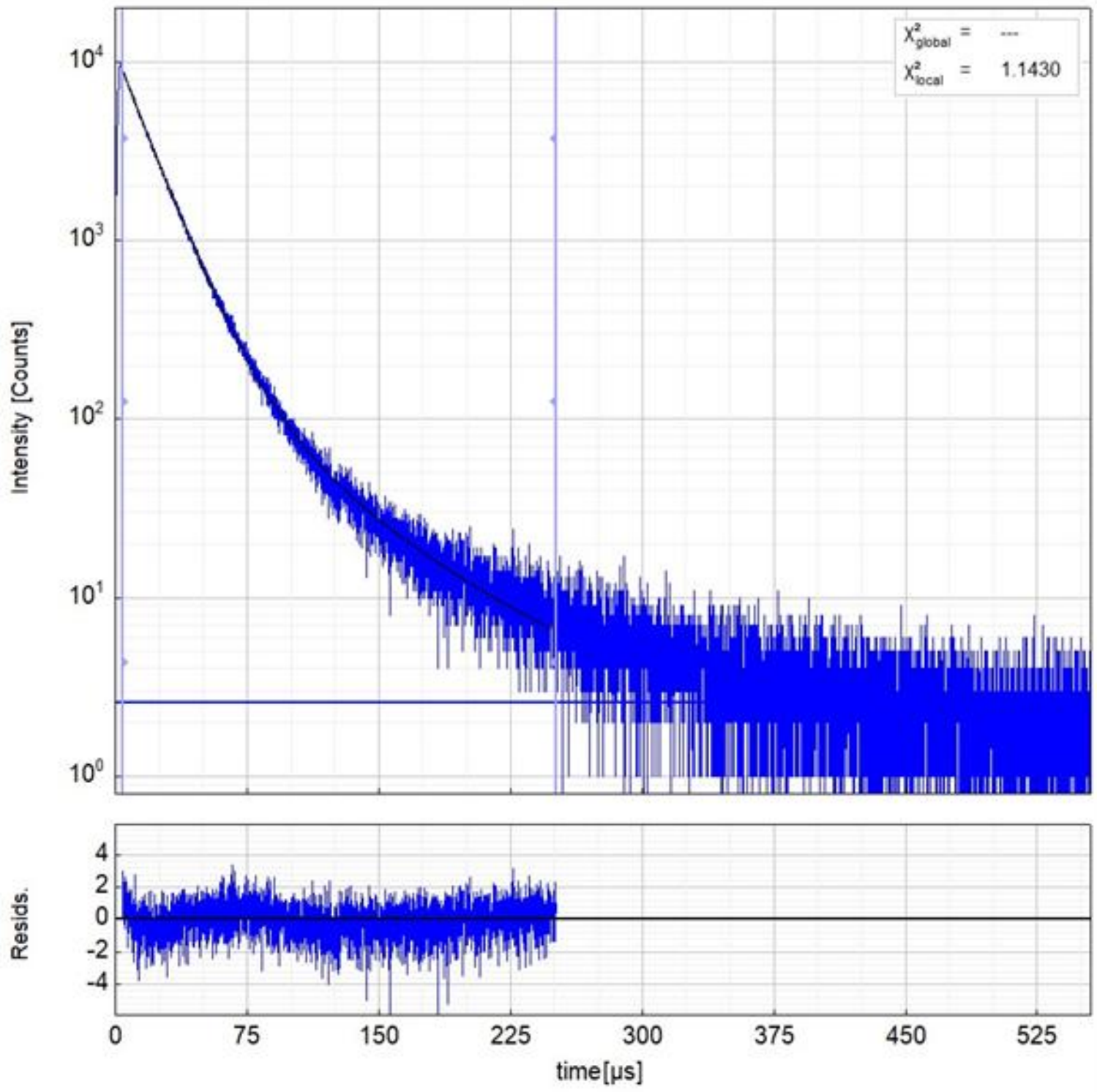

Parameter	Value	Δ	δ
A_1[kCnts/Chnl]	0.301	±0.020	6.6%
τ_1[ns]	57 000	±1 500	2.5%
I_1[kCnts]	268	±11	4.1%
A_{Rel1}[%]	3.5	±0.3	6.6%
I_{Rel1}[%]	10.8	±0.5	4.1%
A_2[kCnts/Chnl]	8.440	±0.027	0.3%
τ_2[ns]	16 809	±84	0.5%
I_2[kCnts]	2 217	±12	0.5%
A_{Rel2}[%]	96.6	±0.3	0.2%
I_{Rel2}[%]	89.3	±0.5	0.5%
$Bkgr_{Dec}$[kCnts]	0.0026	---	---
τ_{AvInt}[ns]	21 140	±69	0.3%
τ_{AvAmp}[ns]	18 191	±50	0.3%

Figure S-358. Left: Raw (experimental) time-resolved photoluminescence decay of **Pt(L_{mDMA})NHC** in a frozen glassy matrix of DCM/MeOH (v/v = 1:1) at 77 K, including the residuals (λ_{exc} = 376.7 nm, λ_{em} = 530 nm). Right: Fitting parameters including pre-exponential factors and confidence limits.

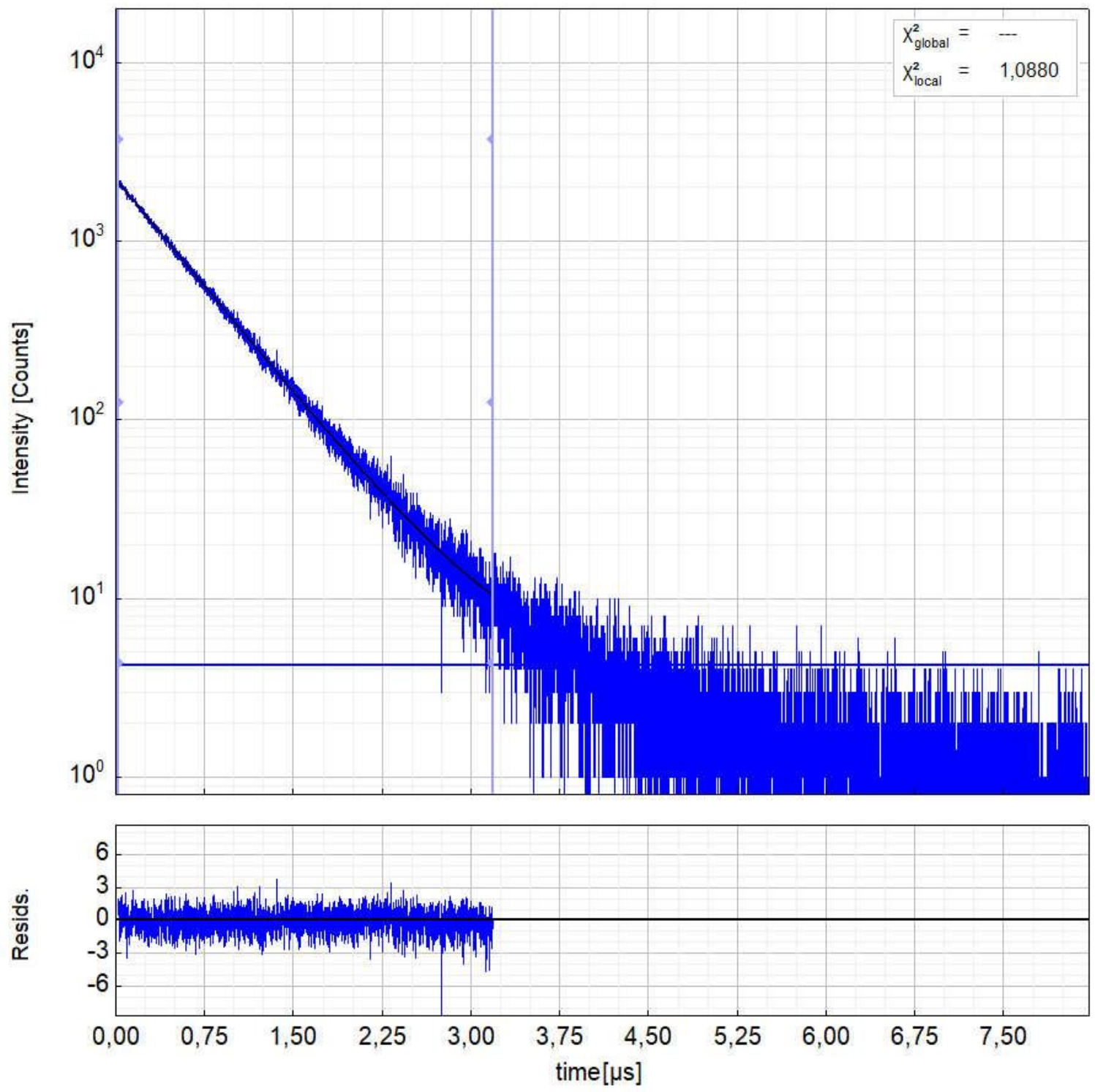

Parameter	Value	Δ	δ
A_1[kCnts/Chnl]	2,1316	±0,0064	0,3%
τ_1[ns]	542,4	±1,6	0,3%
I_1[kCnts]	1 156,1	±2,2	0,2%
A_{Rel1}[%]	100,0	---	---
I_{Rel1}[%]	100,0	---	---
$Bkgr_{Dec}$[kCnts]	0,0042	±0,0004	7,4%
τ_{AvInt}[ns]	542,4	±1,6	0,3%
τ_{AvAmp}[ns]	542,4	±1,6	0,3%

Figure S-359. Left: Raw (experimental) time-resolved photoluminescence decay of **Pt(L_{pDMA})PPh_3** in liquid DCM at 298 K (Ar-purged), including the residuals (λ_{exc} = 376.7 nm, λ_{em} = 700 nm). Right: Fitting parameters including pre-exponential factors and confidence limits.

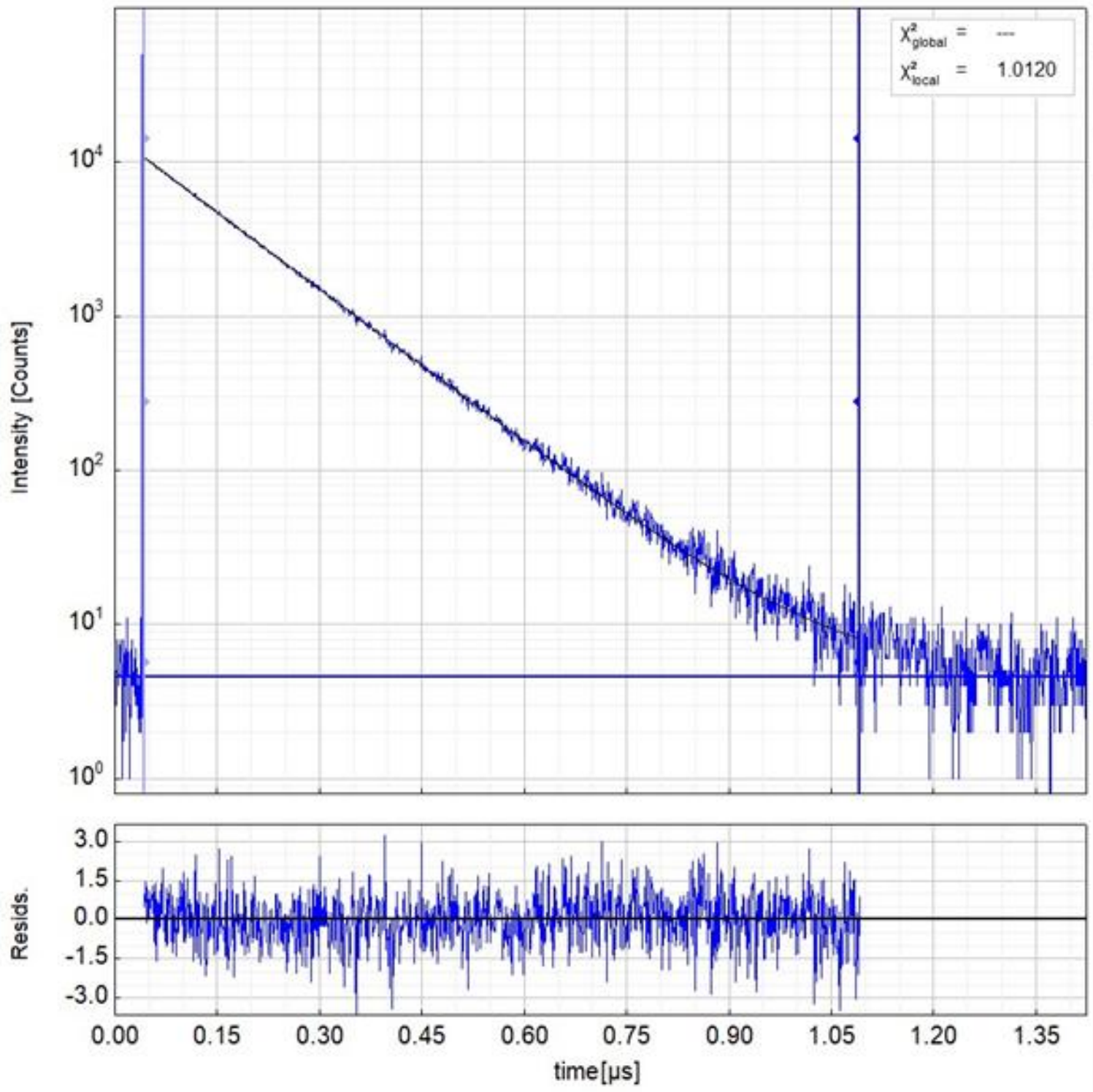

Parameter	Value	Δ	δ
A_1[kCnts/Chnl]	10.608	±0.012	0.1%
τ_1[ns]	130.78	±0.15	0.1%
I_1[kCnts]	1 387.2	±1.5	0.1%
A_{Rel1}[%]	100.0	---	---
I_{Rel1}[%]	100.0	---	---
$Bkgr_{Dec}$[kCnts]	0.0046	---	---
τ_{AvInt}[ns]	130.78	±0.15	0.1%
τ_{AvAmp}[ns]	130.78	±0.15	0.1%

Figure S-360. Left: Raw (experimental) time-resolved photoluminescence decay of **Pt(L$_{pDMA}$)PPh$_3$** in liquid DCM at 298 K (air-equilibrated), including the residuals (λ_{exc} = 376.7 nm, λ_{em} = 693 nm). Right: Fitting parameters including pre-exponential factors and confidence limits.

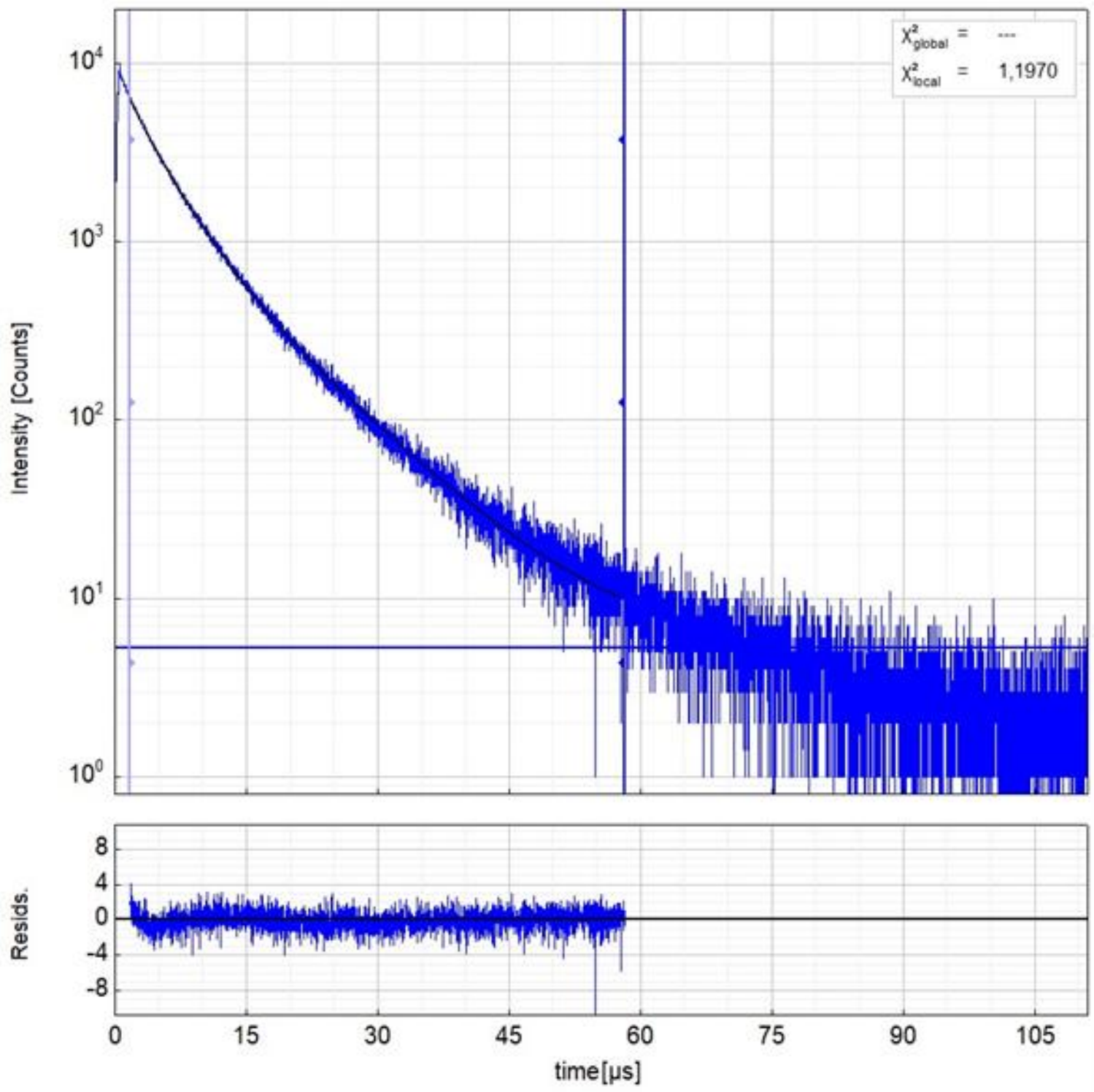

Parameter	Value	Δ	δ
A_1[kCnts/Chnl]	1,632	±0,077	4,7%
τ_1[ns]	9 610	±140	1,4%
I_1[kCnts]	980	±33	3,3%
A_{Rel1}[%]	26,5	±1,1	4,2%
I_{Rel1}[%]	47,6	±1,6	3,3%
A_2[kCnts/Chnl]	4,541	±0,063	1,4%
τ_2[ns]	3 803	±70	1,8%
I_2[kCnts]	1 080	±32	3,0%
A_{Rel2}[%]	73,6	±1,1	1,5%
I_{Rel2}[%]	52,5	±1,6	3,0%
$Bkgr_{Dec}$[kCnts]	0,0053	---	---
τ_{AvInt}[ns]	6 563	±19	0,3%
τ_{AvAmp}[ns]	5 337	±28	0,5%

Figure S-361. Left: Raw (experimental) time-resolved photoluminescence decay of **Pt(L$_{pDMA}$)PPh$_3$** in a frozen glassy matrix of DCM/MeOH (v/v = 1:1) at 77 K, including the residuals (λ_{exc} = 376.7 nm, λ_{em} = 700 nm). Right: Fitting parameters including pre-exponential factors and confidence limits.

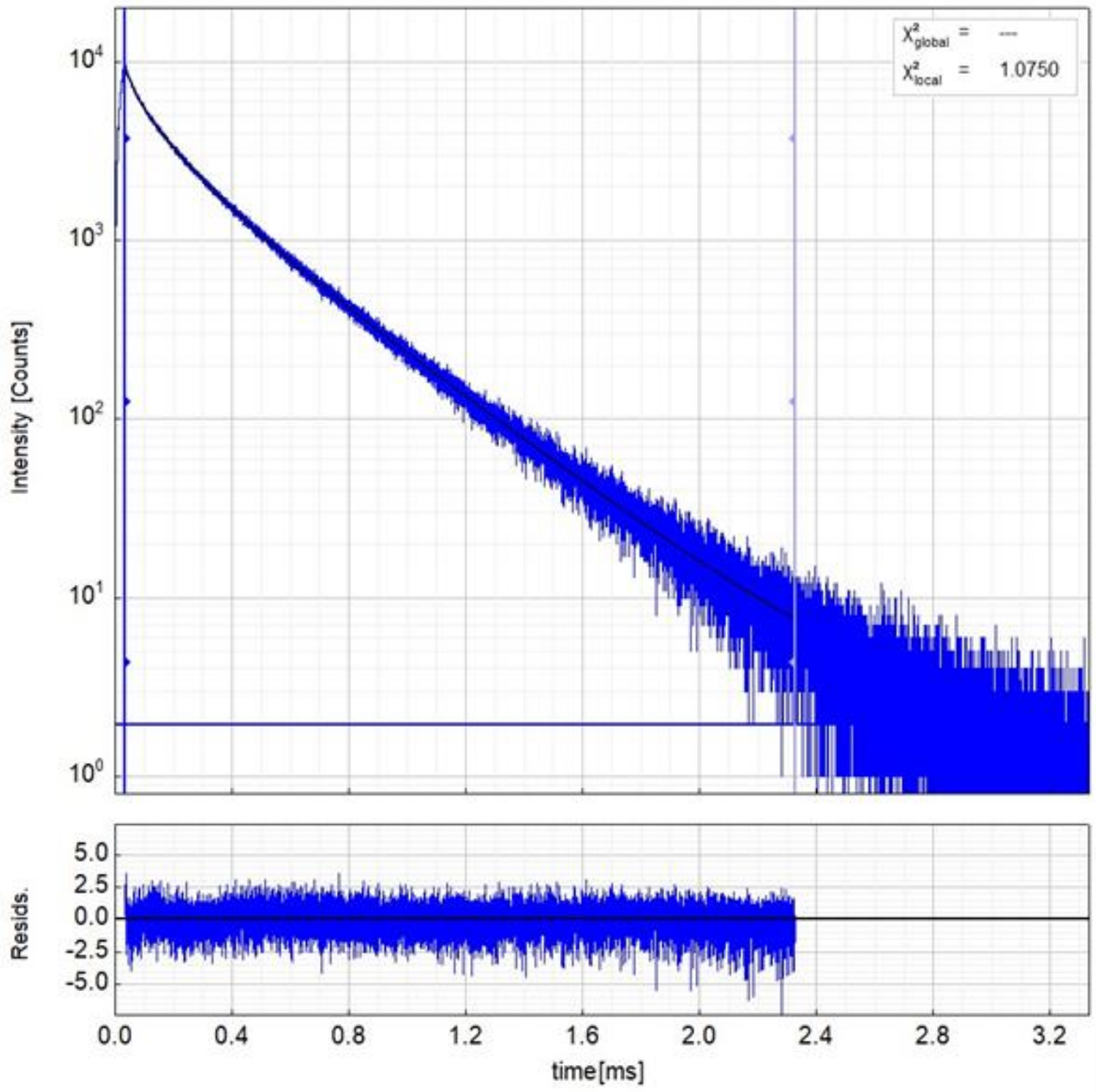

Parameter	Value	Δ	δ
A_1[kCnts/Chnl]	3.36	±0.17	4.9%
τ_1[ns]	359 100	±5 700	1.6%
I_1[kCnts]	9 410	±330	3.5%
A_{Rel1}[%]	36.6	±1.8	4.8%
I_{Rel1}[%]	66.6	±2.4	3.6%
A_2[kCnts/Chnl]	3.605	±0.065	1.8%
τ_2[ns]	146 400	±9 100	6.2%
I_2[kCnts]	4 130	±260	6.3%
A_{Rel2}[%]	39.3	±0.7	1.6%
I_{Rel2}[%]	29.2	±1.9	6.2%
A_3[kCnts/Chnl]	2.22	±0.15	6.6%
τ_3[ns]	35 300	±3 300	9.2%
I_3[kCnts]	610	±96	16%
A_{Rel3}[%]	24.2	±1.7	7.0%
I_{Rel3}[%]	4.4	±0.7	16%
$Bkgr_{Dec}$[kCnts]	0.0020	±0.0006	27%
τ_{AvInt}[ns]	283 070	±770	0.3%
τ_{AvAmp}[ns]	197 300	±1 200	0.6%

Figure S-362. Left: Raw (experimental) time-resolved photoluminescence decay of **Pd(L_{tBu})PPh_3** in a frozen glassy matrix of DCM/MeOH (v/v = 1:1) at 77 K, including the residuals (λ_{exc} = 376.7 nm, λ_{em} = 522 nm). Right: Fitting parameters including pre-exponential factors and confidence limits.

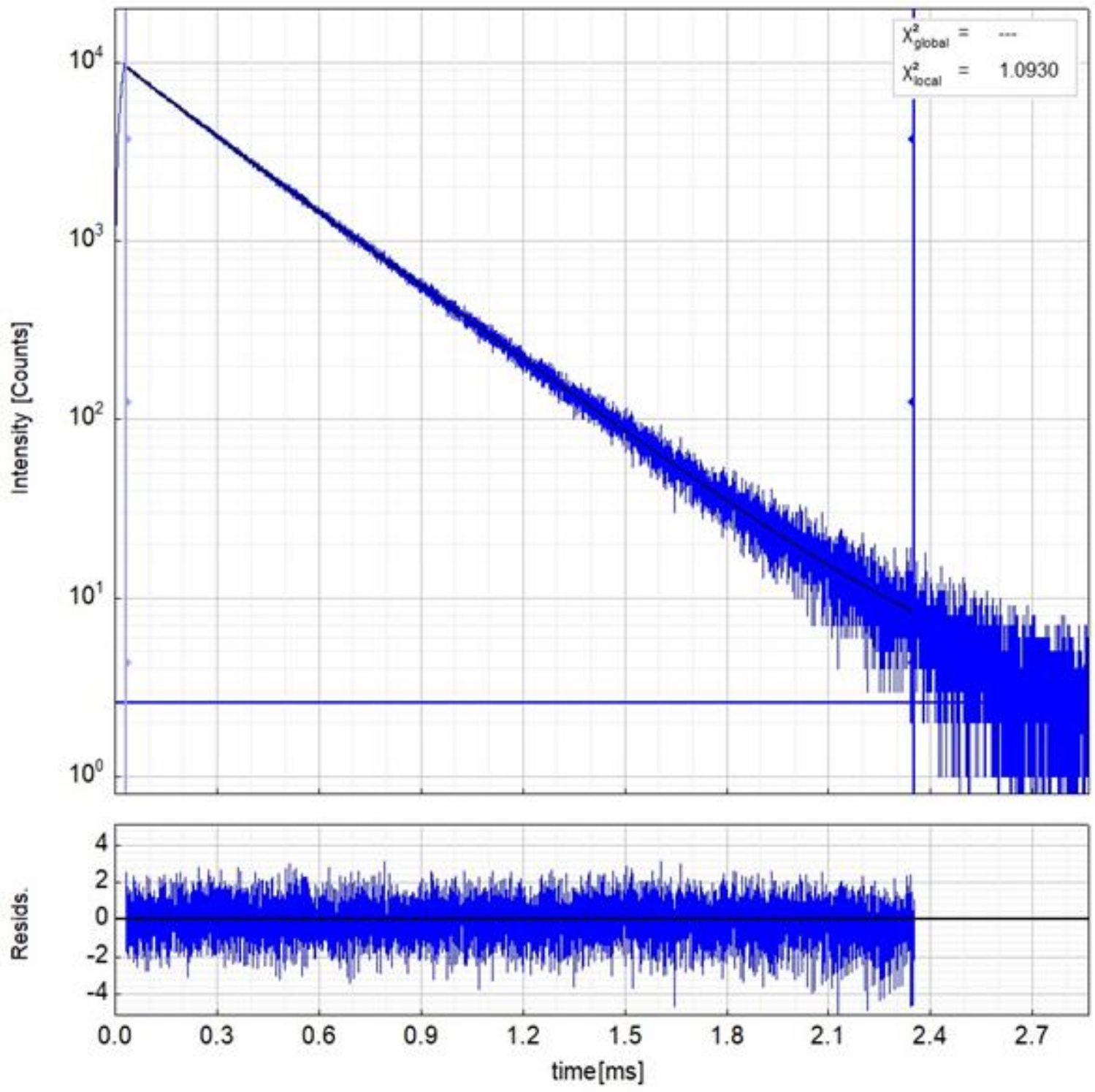

Parameter	Value	Δ	δ
A_1[kCnts/Chnl]	8.555	±0.080	0.9%
τ_1[ns]	317 580	±770	0.2%
I_1[kCnts]	10 613	±76	0.7%
A_{Rel1}[%]	90.1	±0.9	0.9%
I_{Rel1}[%]	94.6	±0.7	0.7%
A_2[kCnts/Chnl]	0.949	±0.078	8.2%
τ_2[ns]	164 900	±7 900	4.8%
I_2[kCnts]	612	±75	12%
A_{Rel2}[%]	10.0	±0.9	8.1%
I_{Rel2}[%]	5.5	±0.7	12%
$Bkgr_{Dec}$[kCnts]	0.0026	---	---
τ_{AvInt}[ns]	309 260	±210	0.1%
τ_{AvAmp}[ns]	302 330	±440	0.1%

Figure S-363. Left: Raw (experimental) time-resolved photoluminescence decay of **Pd(L_{tBu})NHC** in a frozen glassy matrix of DCM/MeOH (v/v = 1:1) at 77 K, including the residuals (λ_{exc} = 376.7 nm, λ_{em} = 465 nm). Right: Fitting parameters including pre-exponential factors and confidence limits.

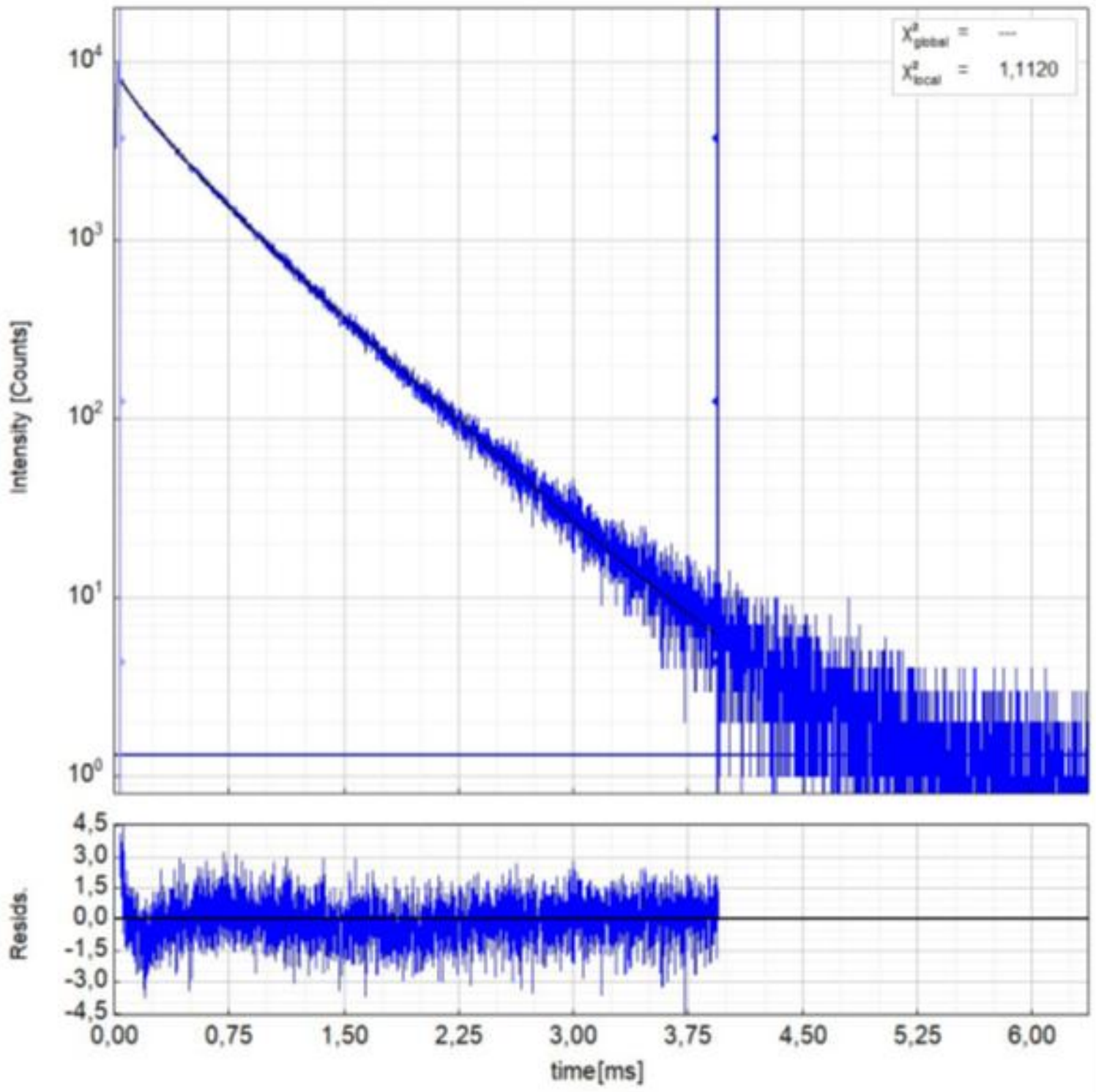

Parameter	Value	Δ	δ
A_1[kCnts/Chnl]	4,745	±0,068	1,4%
τ_1[ns]	566 600	±2 400	0,4%
I_1[kCnts]	2 625	±29	1,1%
A_{Rel1}[%]	61,6	±0,9	1,4%
I_{Rel1}[%]	78,3	±0,9	1,1%
A_2[kCnts/Chnl]	2,968	±0,067	2,2%
τ_2[ns]	252 200	±4 100	1,6%
I_2[kCnts]	731	±28	3,8%
A_{Rel2}[%]	38,5	±0,9	2,2%
I_{Rel2}[%]	21,8	±0,9	3,8%
$Bkgr_{Dec}$[kCnts]	0,0013	---	---
τ_{AvInt}[ns]	498 110	±650	0,1%
τ_{AvAmp}[ns]	445 600	±940	0,2%

Figure S-364. Left: Raw (experimental) time-resolved photoluminescence decay of **Pd(L_{mOMe})PPh_3** in a frozen glassy matrix of DCM/MeOH (v/v = 1:1) at 77 K, including the residuals (λ_{exc} = 376.7 nm, λ_{em} = 475 nm). Right: Fitting parameters including pre-exponential factors and confidence limits.

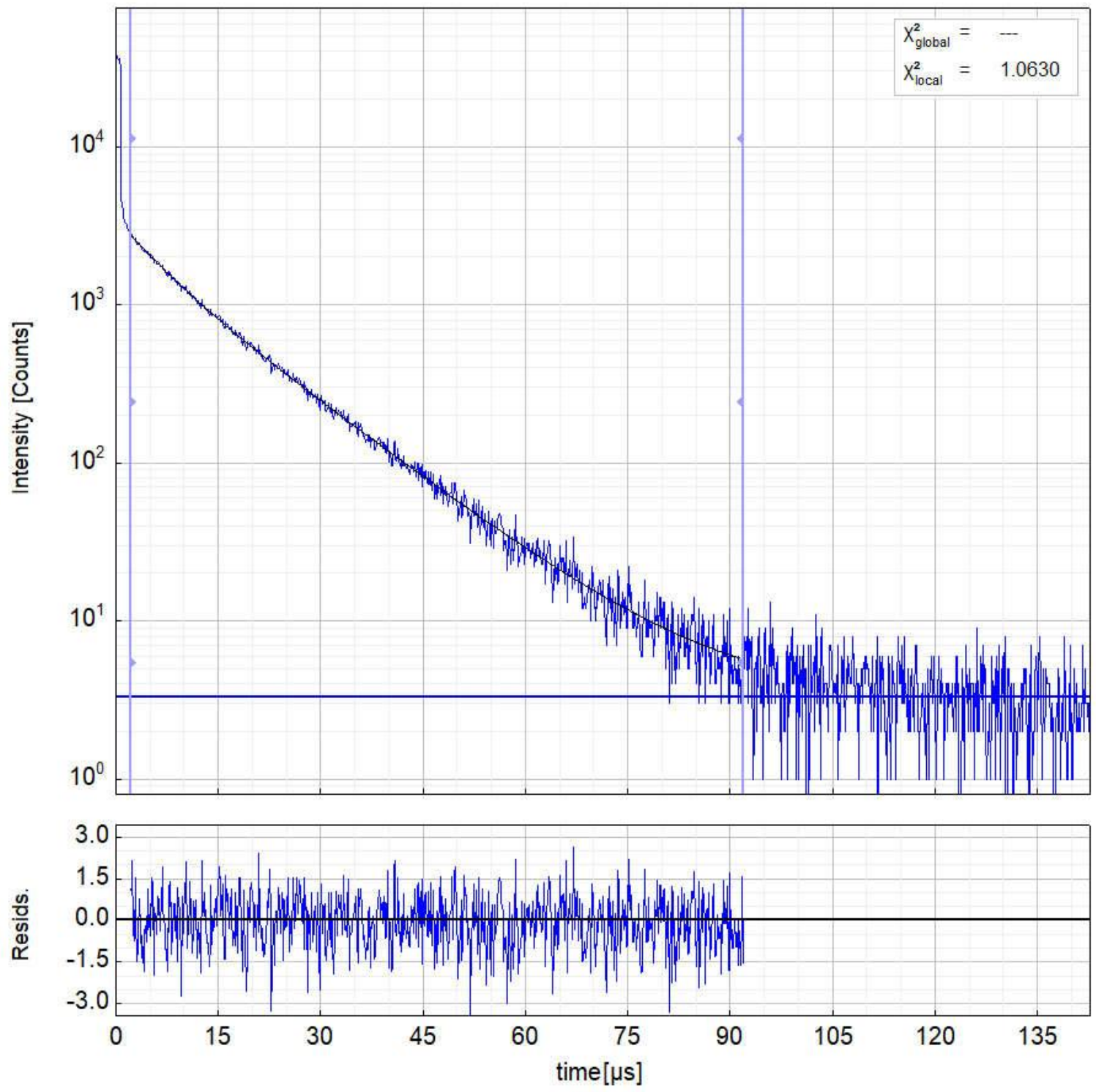

Parameter	Value	Δ	δ
A_1[kCnts/Chnl]	1.90	±0.16	8.3%
τ_1[ns]	13 460	±420	3.1%
I_1[kCnts]	199	±11	5.4%
A_{Rel1}[%]	68.6	±5.3	7.7%
I_{Rel1}[%]	84.7	±4.7	5.5%
A_2[kCnts/Chnl]	0.87	±0.14	16%
τ_2[ns]	5 320	±870	16%
I_2[kCnts]	36	±12	31%
A_{Rel2}[%]	31.5	±5.3	17%
I_{Rel2}[%]	15.4	±4.7	30%
$Bkgr_{Dec}$[kCnts]	0.0034	±0.0007	18%
τ_{AvInt}[ns]	12 207	±86	0.7%
τ_{AvAmp}[ns]	10 900	±190	1.7%

Figure S-365. Left: Raw (experimental) time-resolved photoluminescence decay of **Pd(L_{mOMe})NHC** in liquid DCM at 298 K (Ar-purged), including the residuals (λ_{exc} = 376.7 nm, λ_{em} = 502 nm). Right: Fitting parameters including pre-exponential factors and confidence limits.

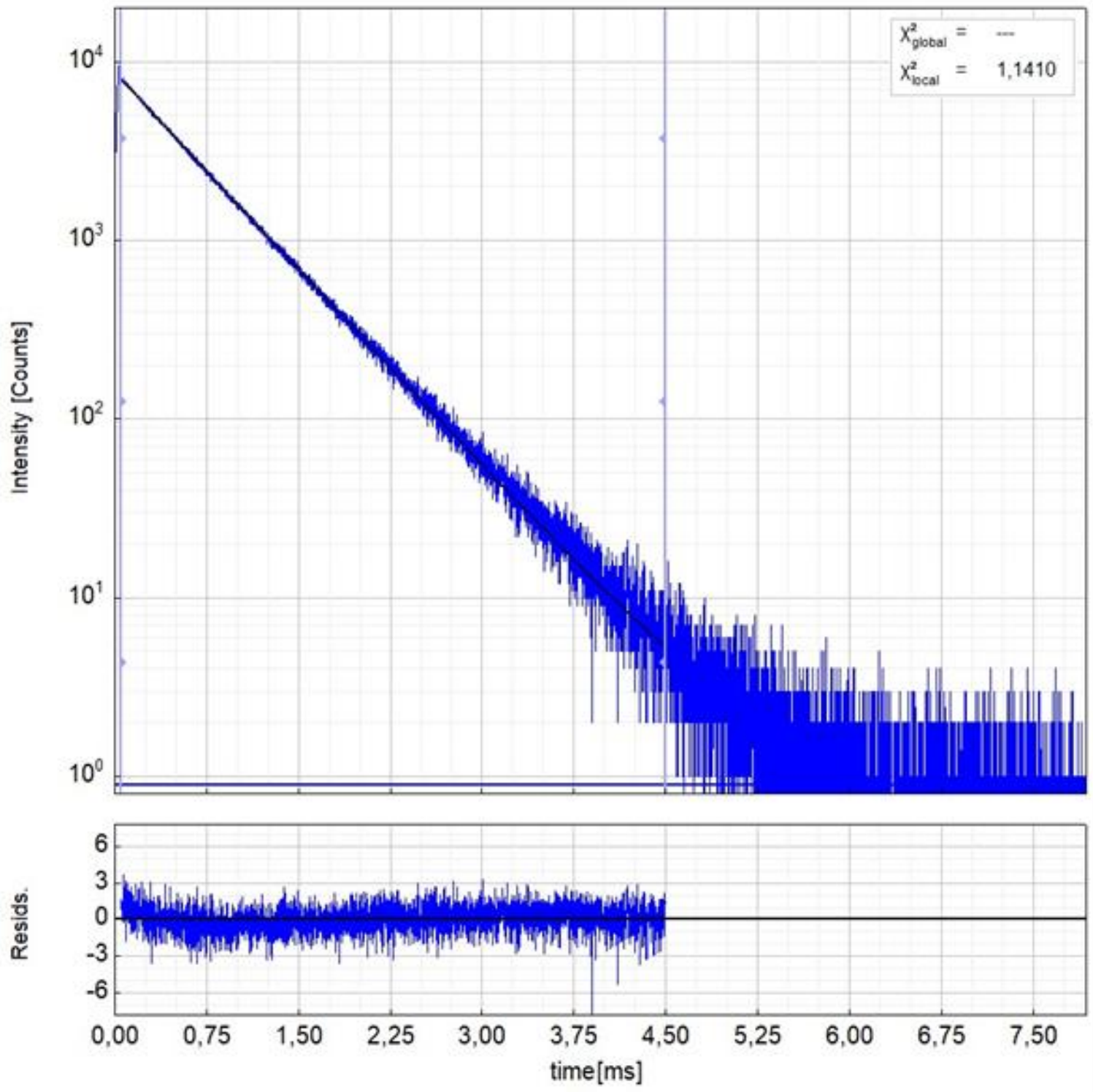

Parameter	Value	Δ	δ
A_1[kCnts/Chnl]	7,940	±0,013	0,2%
τ_1[ns]	592 550	±570	0,1%
I_1[kCnts]	4 594,1	±5,8	0,1%
A_{Rel1}[%]	100,0	---	---
I_{Rel1}[%]	100,0	---	---
$Bkgr_{Dec}$[kCnts]	0,0009	---	---
τ_{AvInt}[ns]	592 550	±570	0,1%
τ_{AvAmp}[ns]	592 550	±570	0,1%

Figure S-366. Left: Raw (experimental) time-resolved photoluminescence decay of **Pd(L_{mOMe})NHC** in a frozen glassy matrix of DCM/MeOH (v/v = 1:1) at 77 K, including the residuals (λ_{exc} = 376.7 nm, λ_{em} = 465 nm). Right: Fitting parameters including pre-exponential factors and confidence limits.

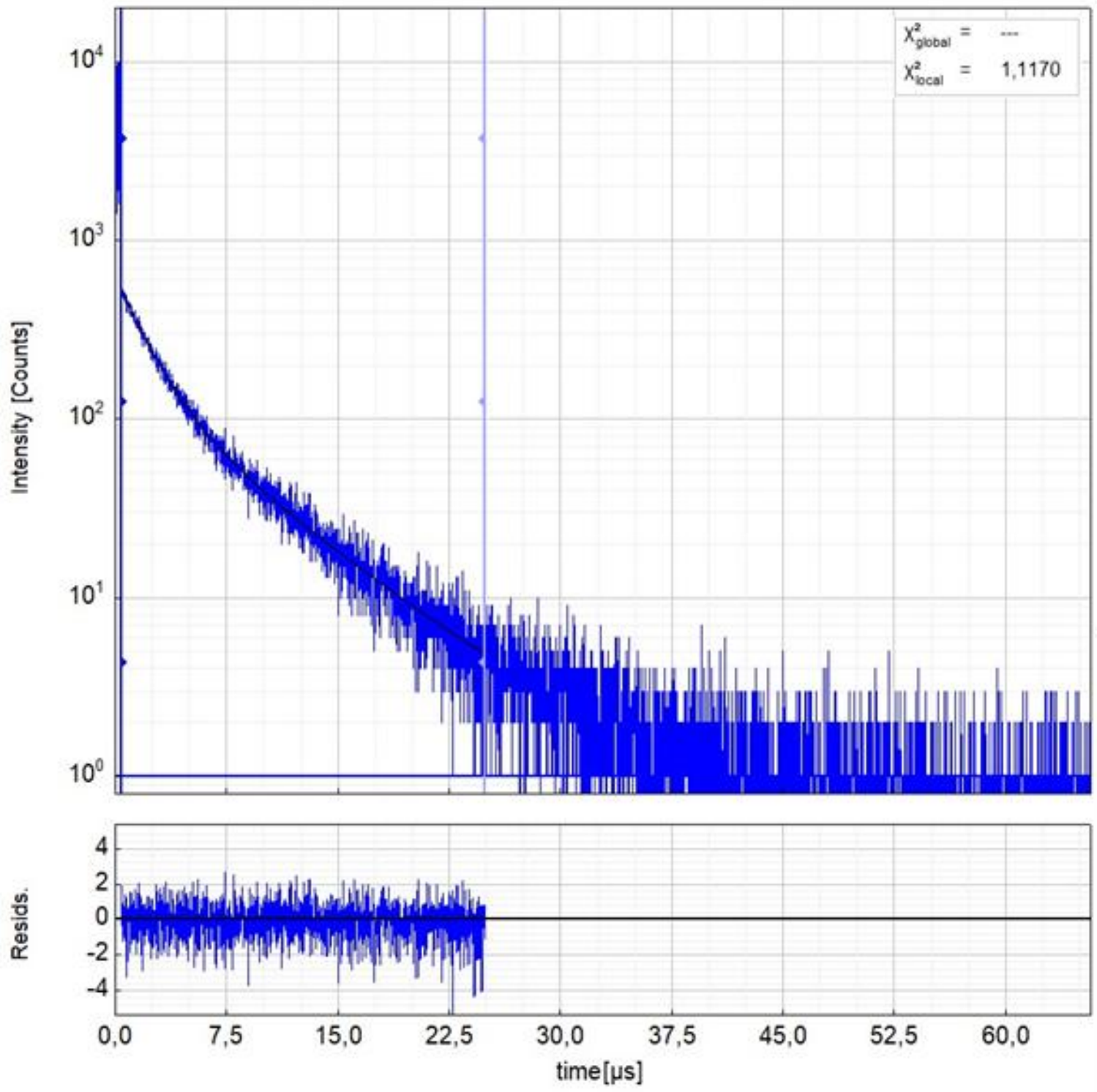

Parameter	Value	Δ	δ
A_1[kCnts/Chnl]	0,153	±0,011	6,6%
τ_1[ns]	6 660	±210	3,0%
I_1[kCnts]	63,4	±2,5	3,8%
A_{Rel1}[%]	29,0	±1,8	6,2%
I_{Rel1}[%]	58,9	±2,4	3,9%
A_2[kCnts/Chnl]	0,374	±0,011	2,8%
τ_2[ns]	1 900	±80	4,2%
I_2[kCnts]	44,4	±2,7	5,9%
A_{Rel2}[%]	71,1	±1,8	2,5%
I_{Rel2}[%]	41,2	±2,4	5,6%
$Bkgr_{Dec}$[kCnts]	0,0010	---	---
τ_{AvInt}[ns]	4 697	±53	1,1%
τ_{AvAmp}[ns]	3 277	±44	1,3%

Figure S-367. Left: Raw (experimental) time-resolved photoluminescence decay of **Pd(L_{mDMA})PPh_3** in liquid DCM at 298 K (Ar-purged), including the residuals (λ_{exc} = 376.7 nm, λ_{em} = 525 nm). Right: Fitting parameters including pre-exponential factors and confidence limits.

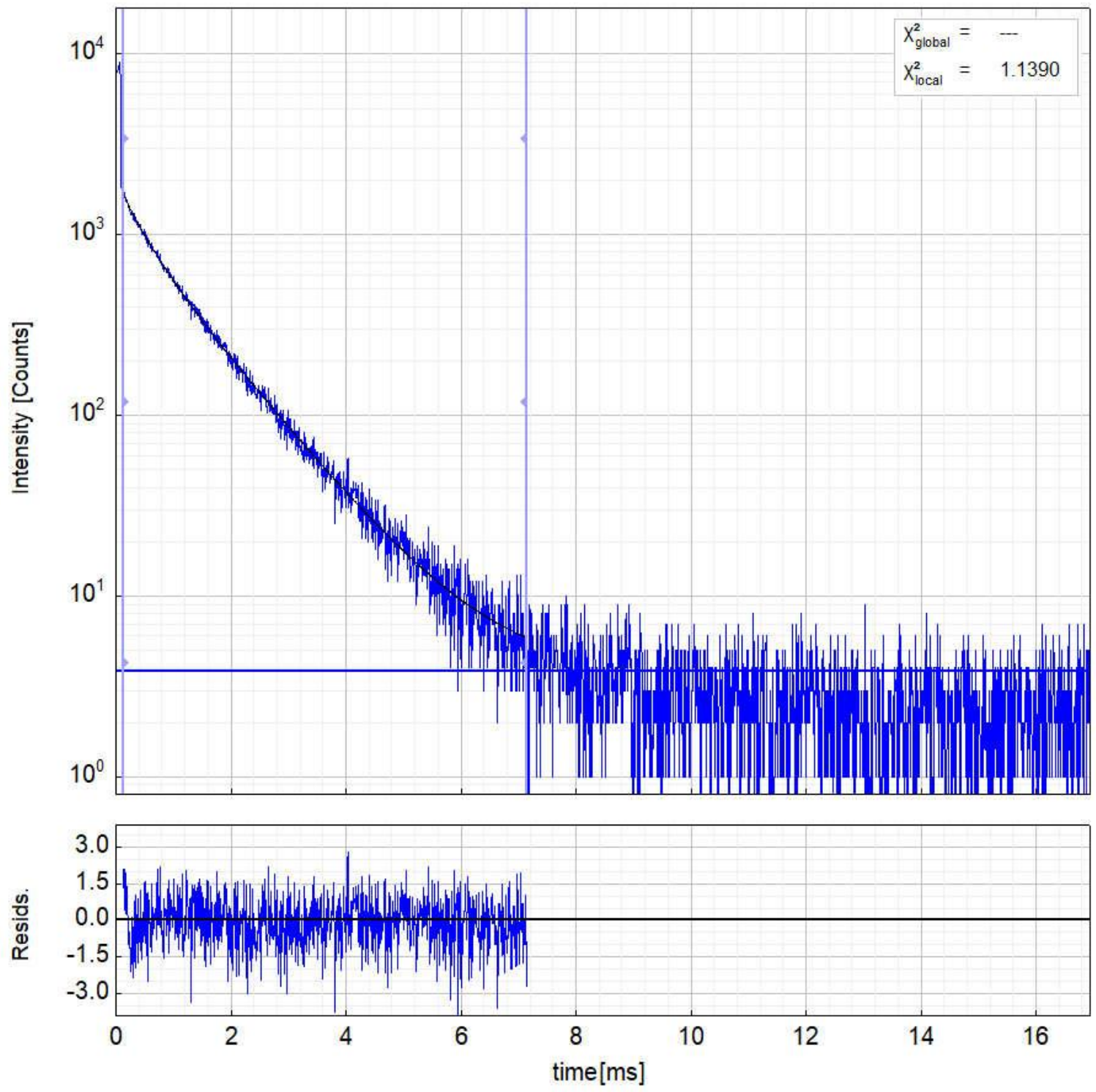

Parameter	Value	Δ	δ
A_1[kCnts/Chnl]	1.066	±0.098	9.2%
τ_1[ns]	1 125 000	±38 000	3.4%
I_1[kCnts]	146.3	±9.0	6.1%
A_{Rel1}[%]	63.9	±5.2	8.1%
I_{Rel1}[%]	83.6	±5.1	6.0%
A_2[kCnts/Chnl]	0.605	±0.080	13%
τ_2[ns]	390 000	±62 000	16%
I_2[kCnts]	28.8	±8.8	30%
A_{Rel2}[%]	36.2	±5.2	14%
I_{Rel2}[%]	16.5	±5.1	30%
$Bkgr_{Dec}$[kCnts]	0.0039	±0.0008	20%
τ_{AvInt}[ns]	1 004 000	±9 400	0.9%
τ_{AvAmp}[ns]	859 000	±12 000	1.4%

Figure S-368. Left: Raw (experimental) time-resolved photoluminescence decay of **Pd(L_{mDMA})PPh_3** in a frozen glassy matrix of DCM/MeOH (v/v = 1:1) at 77 K, including the residuals (λ_{exc} = 376.7 nm, λ_{em} = 530 nm). Right: Fitting parameters including pre-exponential factors and confidence limits.

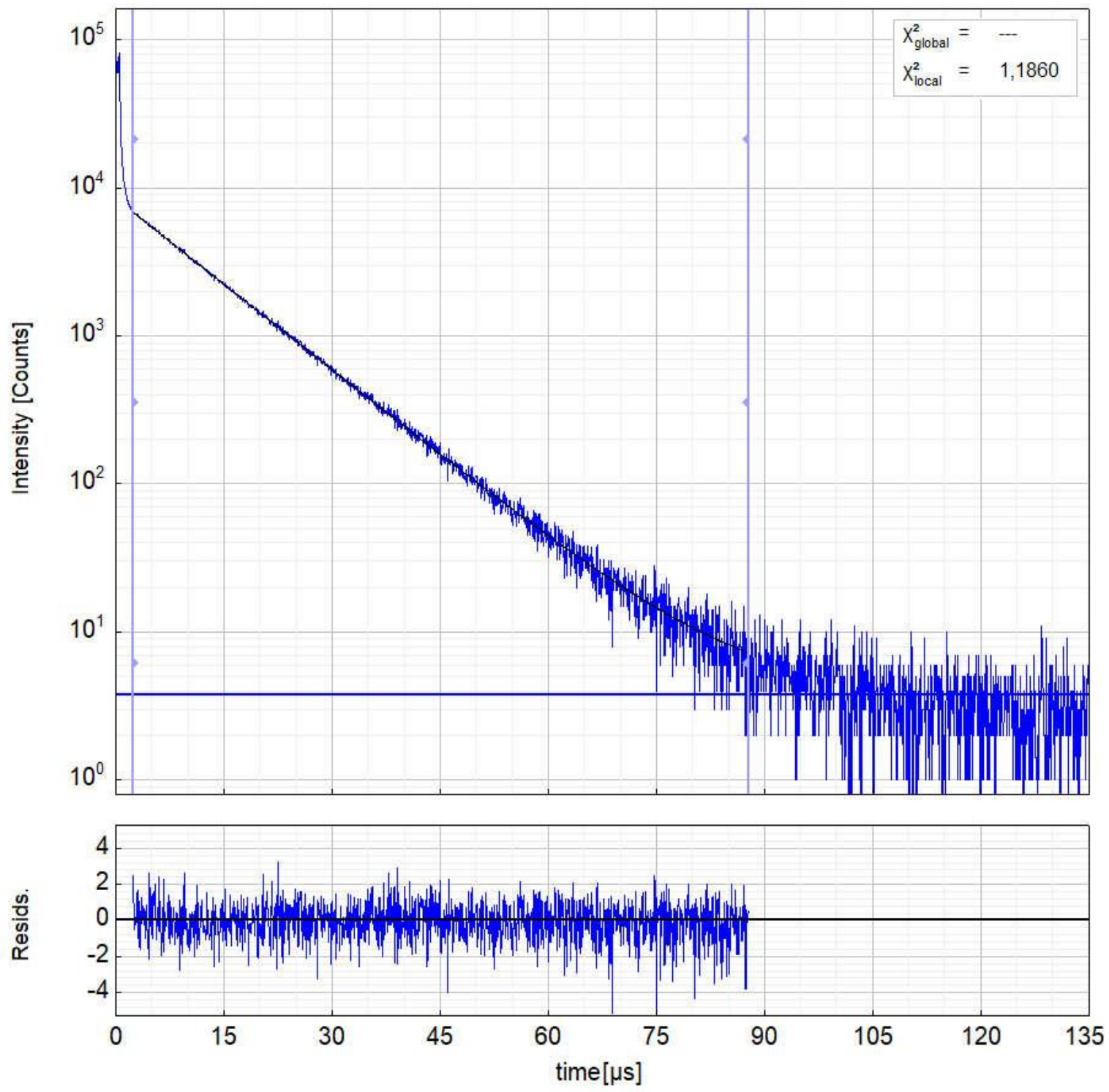

Parameter	Value	Δ	δ
A_1[kCnts/Chnl]	6,847	±0,016	0,2%
τ_1[ns]	11 259	±19	0,2%
I_1[kCnts]	1 204,5	±2,4	0,2%
A_{Rel1}[%]	100,0	---	---
I_{Rel1}[%]	100,0	---	---
$Bkgr_{Dec}$[kCnts]	0,0038	±0,0005	13%
τ_{AvInt}[ns]	11 259	±19	0,2%
τ_{AvAmp}[ns]	11 259	±19	0,2%

Figure S-369. Left: Raw (experimental) time-resolved photoluminescence decay of **Pd(L_{mDMA})NHC** in liquid DCM at 298 K (Ar-purged), including the residuals (λ_{exc} = 376.7 nm, λ_{em} = 504 nm). Right: Fitting parameters including pre-exponential factors and confidence limits.

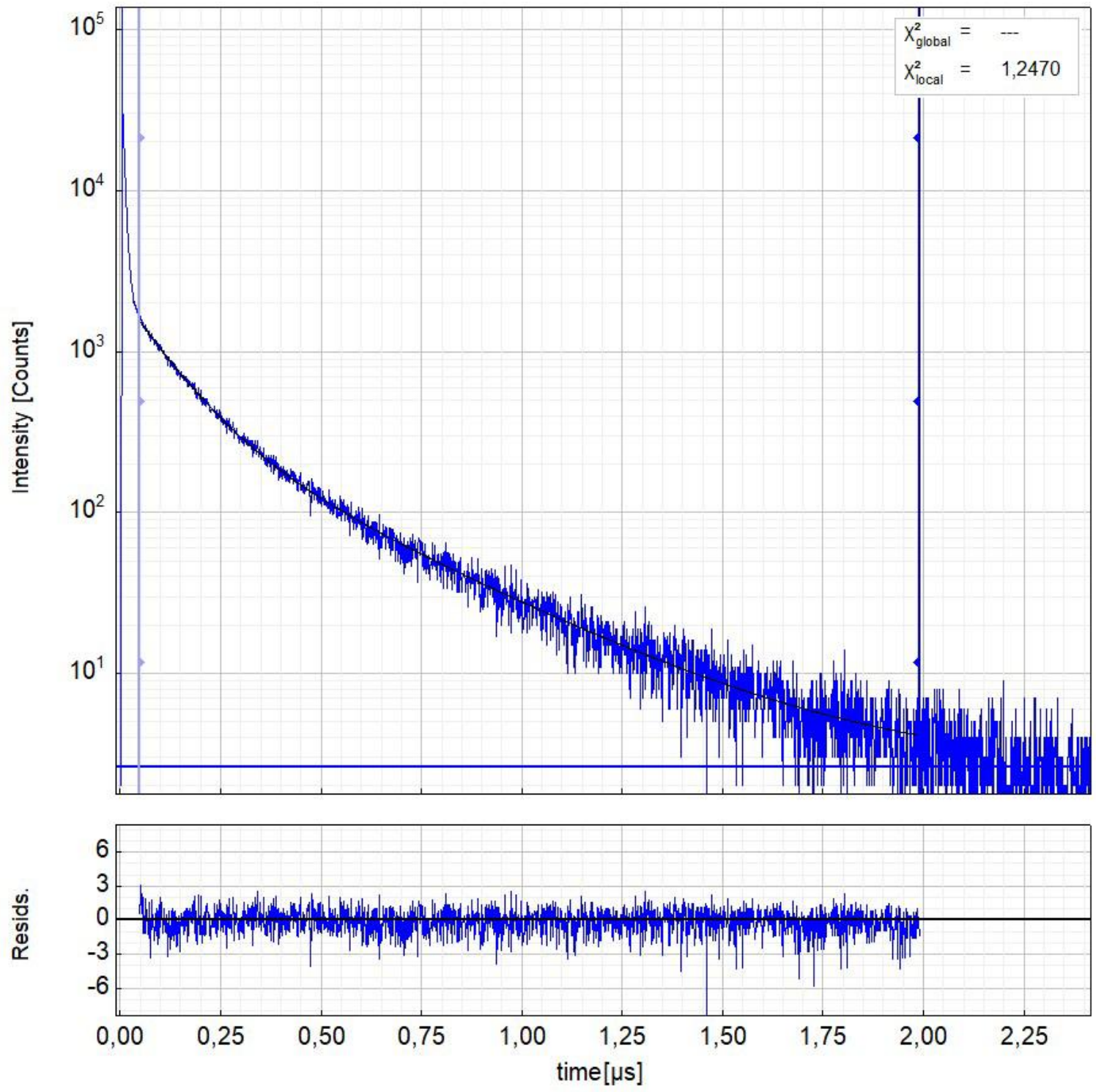

Parameter	Value	Δ	δ
A_1[kCnts/Chnl]	0,373	±0,017	4,4%
$τ_1$[ns]	352,8	±8,7	2,5%
I_1[kCnts]	131,4	±2,9	2,1%
A_{Rel1}[%]	24,1	±1,1	4,3%
I_{Rel1}[%]	51,4	±1,2	2,3%
A_2[kCnts/Chnl]	1,177	±0,020	1,7%
$τ_2$[ns]	105,7	±2,1	1,9%
I_2[kCnts]	124,3	±3,4	2,7%
A_{Rel2}[%]	76,0	±1,1	1,4%
I_{Rel2}[%]	48,7	±1,2	2,4%
$Bkgr_{Dec}$[kCnts]	0,0027	±0,0004	13%
$τ_{AvInt}$[ns]	232,7	±2,7	1,1%
$τ_{AvAmp}$[ns]	165,1	±1,6	1,0%

Figure S-370. Left: Raw (experimental) time-resolved photoluminescence decay of **Pd(L_{mDMA})NHC** in liquid DCM at 298 K (air-equilibrated), including the residuals (λ_{exc} = 376.7 nm, λ_{em} = 504 nm). Right: Fitting parameters including pre-exponential factors and confidence limits.

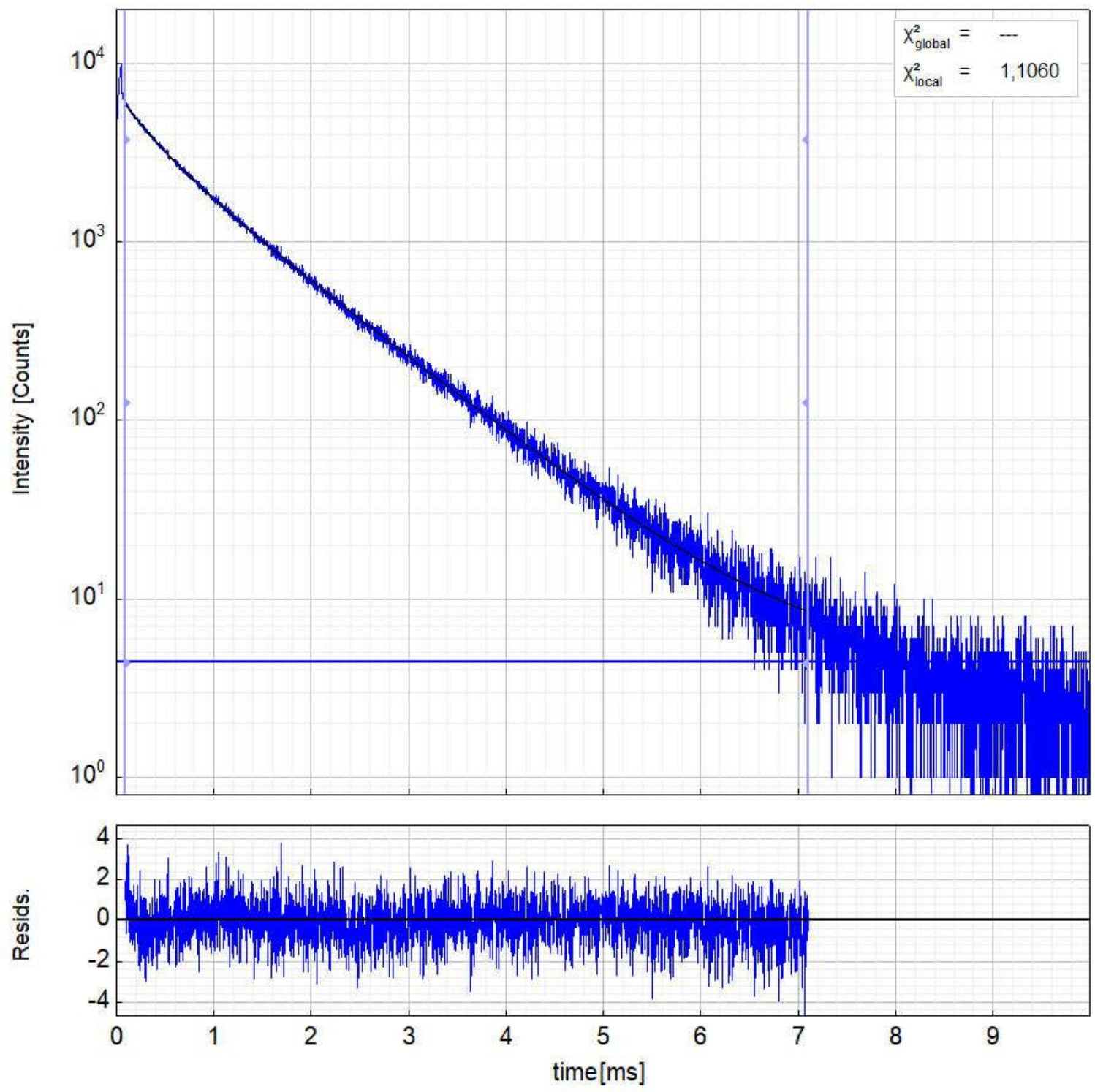

Parameter	Value	Δ	δ
A_1[kCnts/Chnl]	3.791	±0.057	1.5%
$τ_1$[ns]	1 025 900	±6 600	0.6%
I_1[kCnts]	1 899	±19	1.0%
A_{Rel1}[%]	64.9	±0.8	1.1%
I_{Rel1}[%]	84.0	±0.8	0.9%
A_2[kCnts/Chnl]	2.054	±0.034	1.6%
$τ_2$[ns]	364 000	±13 000	3.4%
I_2[kCnts]	365	±19	5.0%
A_{Rel2}[%]	35.2	±0.8	2.1%
I_{Rel2}[%]	16.1	±0.8	4.9%
$Bkgr_{Dec}$[kCnts]	0.0045	±0.0002	2.8%
$τ_{AvInt}$[ns]	919 300	±1 500	0.2%
$τ_{AvAmp}$[ns]	793 100	±3 200	0.4%

Figure S-371. Left: Raw (experimental) time-resolved photoluminescence decay of **Pd(L_{mDMA})NHC** in a frozen glassy matrix of DCM/MeOH (v/v = 1:1) at 77 K, including the residuals (λ_{exc} = 376.7 nm, λ_{em} = 520 nm). Right: Fitting parameters including pre-exponential factors and confidence limits.

Section S6: References

[1] M. M. Rahman, J. Zhang, Q. Zhao, J. Feliciano, E. Bisz, B. Dziuk, R. Lalancette, R. Szostak, M. Szostak, *Organometallics* **2022**, *41*, 2281.

[2] SAINT+ (includes Xprep and SADABS); Bruker AXS Inc., Madison, Wisconsin, USA, 2021.

[3] G. M. Sheldrick, *Acta Crystallogr*. **2002**, *64*, 112.

[4] I. J. Bruno, J. C. Cole, P. R. Edgington, M. Kessler, C. F. Macrae, P. McCabe, J. Pearson, R. Taylor, *Acta Crystallogr.* **2002**, *58*, 389.
